# African cichlid fishes: morphological data and taxonomic insights from a genus-level survey of supraneurals, pterygiophores, and vertebral counts (Ovalentaria, Blenniiformes, Cichlidae, Pseudocrenilabrinae)

**DOI:** 10.3897/BDJ.12.e130707

**Published:** 2024-10-18

**Authors:** Michael K. Oliver

**Affiliations:** 1 Yale Peabody Museum of Natural History, New Haven, United States of America Yale Peabody Museum of Natural History New Haven United States of America

**Keywords:** osteology, skeleton, meristic characters, pattern characters, fin supports, vertebrae, survey, radiograph, x-ray, microCT, Etroplinae, Ptychochrominae, Cichlinae, Lake Tanganyika, Lake Malawi, Haplochromini, Pseudocrenilabrini, new subtribe, Polycentridae, Pholidichthyidae

## Abstract

**Background:**

The iconic freshwater cichlid fishes (Cichlidae) comprise about 1750 validly named species and hundreds more that are known, but not yet described and named. Cichlids are an important source of protein for millions of people on several continents, are model organisms in studies of evolution, speciation, ecology, development, behaviour and physiology and are popular as aquarium fishes. Yet, comparative studies of cichlid internal anatomy are rare. Even their osteology has not been taxonomically surveyed. The cichlid postcranial skeleton has been especially neglected.

**New information:**

Here, I provide the first survey in cichlids of the considerable variation in numbers of vertebrae, supraneurals and dorsal- and anal-fin supports (pterygiophores), as well as the patterns with which the pterygiophores insert between the neural or haemal spines. The study includes some 1700 specimens of nearly 400 cichlid species. Focusing on the largest subfamily, the African cichlids or Pseudocrenilabrinae, the survey furnishes data from species in all but one of its 166 genera. Limited data from species in the other cichlid subfamilies (Etroplinae, Ptychochrominae and Cichlinae) and from the related leaffishes, Polycentridae, are also presented. Key examples of pterygiophore insertion patterns from throughout the range of variation are illustrated and discussed. Detailed analytical tables and all raw data are provided in supplementary files.

A bizarre specialisation in *Cyprichromis* is noted, evidently for the first time. Uniquely in this Lake Tanganyikan genus, five to seven anal pterygiophores are abdominal in position, located anterior to the anal fin and inserting toward or between successive pairs of pleural ribs.

Taxonomic changes: The most speciose tribe of African cichlids, currently known as Haplochromini, is correctly called Pseudocrenilabrini. Based chiefly on the molecular phylogenetic findings of other workers, I propose four pseudocrenilabrine subtribes, one occurring in rivers and three endemic to Lake Malawi. I also re-assign the Lake Tanganyikan tribe Tropheini as another subtribe of Pseudocrenilabrini, in line with numerous molecular studies placing tropheines firmly within this tribe. The remaining genera of Pseudocrenilabrini remain *incertae sedis* in this tribe pending clarification of their phylogenetic relationships.

The character complex here surveyed is a promising source of taxonomically and phylogenetically informative characteristics distinguishing or uniting cichlid taxa at multiple hierarchical levels, from species through subfamily. This reference set of novel character data can also provide information for palaeontological studies of African cichlids. These attributes are skeletal features potentially available for study in well preserved fossils and may help determine their correct taxonomic placement.

## Introduction

### Dedication

In memory of David Henry Eccles, MBE (7 August 1932–7 December 2021), extraordinary naturalist, polymath and mentor.

### History

“*The purposes of this study are to present data on the complete supraneural and pterygiophore insertion patterns of all known extant carangid species and to analyze these patterns for information useful in recognizing differences among these taxa that will aid in their identification or suggest specializations that might aid in hypothesizing intrafamilial relationships. Additionally, we hope that our findings will encourage others to explore the utility of this character complex in other groups of fishes” —* Springer and Smith-Vaniz (2008).

The Cichlidae contains some 1756 valid species of freshwater fishes in four subfamilies (Fricke et al. 2024a). The largest of these, encompassing all African cichlids and those of the Middle East, is the Pseudocrenilabrinae with 1161 described species — fully two-thirds of all cichlids. The actual number of pseudocrenilabrine species, however, is much higher. Turner et al. (2001) asked "How many species of cichlid fishes are there in African lakes?" but, after discussing all the problems, could not conclude more precisely than "...there are a great many cichlid species in Lakes Malawi, Tanganyika and Victoria". In Lake Malawi alone, far beyond the 413 species currently described (Oliver 2024), some 400–600 additional known or suspected species await description (Snoeks 2004; Konings 2016). In Lake Tanganyika, between 33 (Ronco et al. 2020) and 105 (Anonymous 2023) undescribed species are known or suspected. Further new species also continue to be collected and, eventually, formally described from satellite lakes of Lake Malawi (Genner et al. 2007, Turner et al. 2019, Turner et al. 2023), Lake Victoria itself (Seehausen 1999, Witte et al. 2007, de Zeeuw et al. 2010, de Zeeuw et al. 2013) and its satellite lakes (Mwanja 2004, Odhiambo et al. 2011a, Odhiambo et al. 2011b, Ogutu-Ohwayo et al. 2013), Lake Edward (Vranken et al. 2019, Vranken et al. 2020, Vranken et al. 2022, Vranken et al. 2024), Lake Mweru (Katongo et al. 2006) and from rivers (e.g. Lamboj 2009, Lamboj 2012, Lamboj et al. 2016, Schedel et al. 2018, Stauffer et al. 2021, Turner et al. 2021, Genner et al. 2022, Ibala Zamba et al. 2022, Lamboj and Koblmüller 2022). Thus, the number of valid African cichlid species eventually recognised is likely to surpass the current total of all species of Cichlidae.

Fossil African cichlids are also being discovered and named at an increasing pace and are currently assigned to 13 extinct genera (Přikryl et al. 2022, table 4). One reason that “[d]etermining the relationships of fossil cichlids is notoriously difficult” is the “lack of morphological datasets that contain characters normally preserved in fossils” (Přikryl et al. 2022).

The musculoskeletal morphology of cichlids, and especially pseudocrenilabrines, has been much studied in the past twenty-five years. These studies, however, have been confined primarily to the head region and to only a few species; for example, Albertson and Kocher (2001), Albertson et al. (2003), Kassam et al. (2004), Hulsey et al. (2007), Postl et al. (2008), le Pabic et al. (2009), Cooper et al. (2010), Cooper et al. (2011), Odhiambo et al. (2011), Parsons et al. (2011), Bird and Webb (2014), Tsuboi et al. (2014), Tsuboi et al. (2015), and Hu and Albertson (2021). Geometric morphometrics is often employed in these reports, which are concerned principally with the evolution of trophic specialisations. With a few exceptions, such as the recent study by Bucklow et al. (2024), the cichlid postcranial skeleton has been virtually ignored.

The current study attempts to fill this knowledge void by providing an extensive genus-level survey of certain postcranial skeletal features. These include both meristic characters (counts of vertebrae, supraneurals and proximal dorsal and anal pterygiophores) and pattern characters (specific insertion locations of pterygiophores relative to neural or haemal spines). This neglected character complex may be available for study in well-preserved fossils and, thus, may provide new characters informing taxonomic and phylogenetic studies of both living and fossil cichlids.

## Materials and methods

### Data sources

This study is based on data I recorded from radiographs, microCT image stacks and a few cleared-and-stained specimens. In all, data from 1705 specimens of 391 cichlid species are included, representing all tribes within the Pseudocrenilabrinae and exemplars of the other three cichlid subfamilies. Limited data from a few leaffishes (Polycentridae) are included for purposes of outgroup comparison. Authors of all species names are given in Suppl. material 12. The primary type specimens (holotype or lectotype) of 90 species are amongst the cichlid specimens included; their values are signalled in the supplementary tables by an asterisk. At least one imaged specimen was available from 165 of the 166 currently recognised genera of Pseudocrenilabrinae (Fricke et al. 2024b). Only *Enigmatochromis* Lamboj 2009 (Chromidotilapiini) is not represented; a radiograph of *E.lucanusi* is known to have been made at the American Museum of Natural History, but efforts to locate it were unsuccessful and the specimens were not available to be re-imaged.

I scanned older film radiographs on an Epson Perfection 4990 flatbed photo scanner at a resolution of 1200 pixels per inch (ppi) and displayed the enlarged images on a monitor while recording the raw meristic data and pterygiophore insertion patterns. For radiographs and microCT images available online, I enlarged the image on the monitor to fullscreen size and captured one or more screenshots, saved in .jpg format at 96 ppi. I later recorded meristic and pattern data, while displaying each image on the monitor at sufficient enlargement to enable seeing all required small details, notably the supraneurals (SN) and the posterior dorsal (DPt) and anal (APt) pterygiophores. If the specimen was not imaged with head to the left, I reversed the image. If the fish’s image was not approximately horizontal or adequately clear to permit reasonable certainty of interpretation, I rotated it or applied one or more digital enhancements in Corel PaintShop Pro X8. These techniques included sharpening with the unsharp mask, adjusting brightness and contrast, adding fill light, increasing image clarity and applying local tone mapping (LTM). LTM proved particularly helpful in bringing out details on low-contrast images. A few images, especially those created from microCT volumes, could not be made usably clear because the small posterior pterygiophores were inadequately resolved in the original image stack. I did not use such images.

### Recording data

I made all counts twice, following the conventions in Springer and Smith-Vaniz (2008). If the two results differed, I repeated the count until it was consistent. If a count appeared likely to be correct, but the contents of one or more dorsal insertion spaces (DIS) or interhaemal spaces (IHS) could not be absolutely confirmed, I indicated the uncertain entry with a question mark in the raw data and used that specimen’s data. If I could not arrive at a repeatable count with reasonable confidence, I excluded that specimen.

I recorded counts of precaudal, caudal and total vertebrae. Following Springer and Smith-Vaniz (2008): fig. 1 and cichlid authors such as Ibala Zamba et al. (2022), I included the compound terminal centrum (urostyle) in the count; it is, after all, a vertebra.

I entered all raw data on to a Microsoft Excel spreadsheet, which became the table in Suppl. material 11. The header row contains consecutive numbers from 1 to n (40, as it turned out), accommodating the maximum total number of vertebrae encountered in any of the studied specimens.

**Figure 1.**
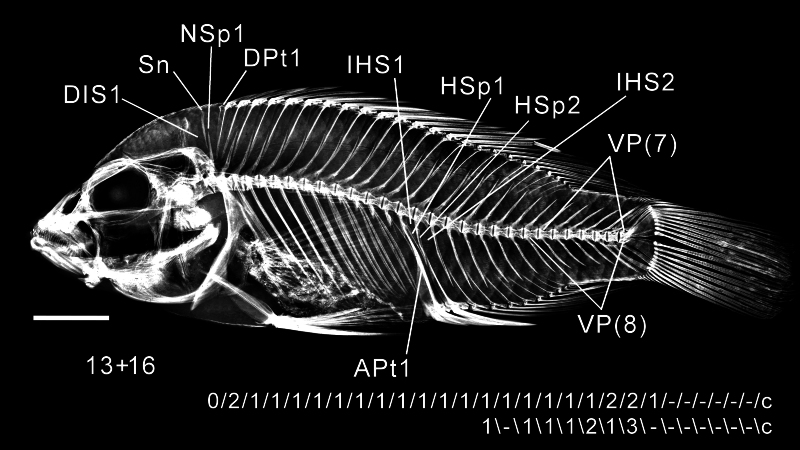
Guide to the structures and insertion spaces treated herein. Abbreviations: APt1, first anal pterygiophore; DIS1, first dorsal insertion space; DPt1, first dorsal (proximal) pterygiophore; HSp1,2, first and second haemal spines; IHS1,2, first and second interhaemal spaces; NSp1, first neural spine; Sn, supraneural; VP (7), the seven vertebrae posterior to the vertebrae participating in last occupied DIS; VP (8), the eight vertebrae posterior to the vertebrae participating in last occupied IHS. Two rows of numbers at lower right illustrate how the 13 precaudal + 16 caudal vertebrae (total 29) and their pterygiophore insertion patterns are recorded (see Suppl. material 11). Each supraneural is indicated by a 0; each solidus (/) represents a neural spine and each reverse solidus (\) represents a haemal spine; numerals represent number of pterygiophores in each insertion space; hyphens represent empty insertion spaces; c is the terminal half-centrum. Base image of *Astatotilapiacalliptera* (USNM 330613) copyright by AMNH, where specimen was x-rayed. Scale bar = 1 cm.

Two successive rows are allocated for each specimen. The number of cells in the upper row, starting from the left, equals the total count of precaudal plus caudal vertebrae, including the compound terminal centrum. For a specimen with a count of 13+16 = 29 vertebrae (Fig. 1), a “c” (for compound centrum) is placed in column 29 of both rows.

In the lower row, the first entry is placed below the cell of the first caudal vertebra — not the last precaudal vertebra as stated by Springer and Smith-Vaniz (2008), as that placement results in lower-row Pt entries misaligned with those of the upper row. Thus, in this example of a 13+16 count, a temporary “x” is placed in the second row column 14 (the precaudal count plus 1) to show where the contents of the first insertion space immediately anterior to the first APt is to be entered.

The entry for each cell records the contents of the corresponding insertion space anterior to that centrum’s neural or haemal spine. In column 1 of the upper row, an entry is made representing the first DIS in front of the first neural spine. A separate “0” is entered for each SN; a numeral is entered to indicate the number of pterygiophores. If more than one element is present in a space, the elements are separated by hyphens. For example, if the insertion space has two SN and two Pt, 0-0-2 is recorded. The contents of each DIS is similarly entered, as shown in Fig. 1. An empty insertion space is indicated with a dash (–). The contents of the IHS are likewise entered in the lower row.

Tribal assignments of genera in the Pseudocrenilabrinae follow Dunz and Schliewen (2013), except for the revised status of Tropheini assigned herein (see Taxon treatments below, which also includes designation of new subtribes within Pseudocrenilabrini). Generic names are those currently recognised as valid by Fricke et al. (2024c), even when these occasionally differ from the usual current practice of cichlid workers, notably for a number of nominal genera similar to *Haplochromis*.

The material examined is listed in Suppl. material 12.

### Limitations of data

**Figure 2.**
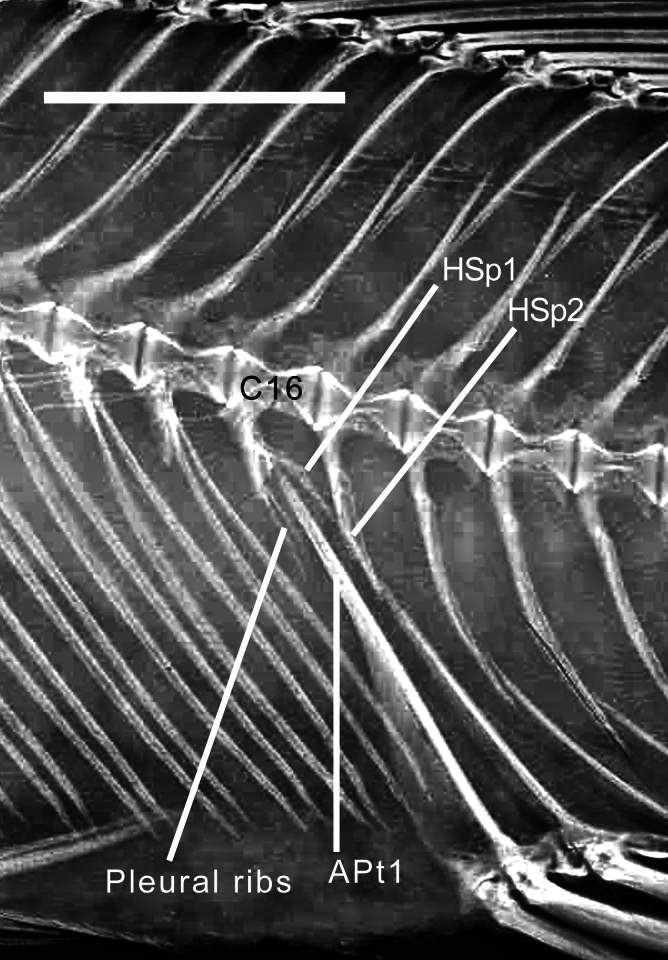
Example of an atypical first caudal vertebra with both a haemal spine and pleural ribs. As this is the vertebra bearing the first haemal spine, which together with the first anal pterygiophore imposes the posterior limit to the abdominal cavity, it is judged the first caudal vertebra. Abbreviations: APt1, first anal pterygiophore; C16, centrum 16; HSp1,2, first and second haemal spines. *Pseudotropheusjohannii* (AMNH 215563). Scale bar = 5 mm. Source image copyright by AMNH.

(1) In most cases, I have not examined the actual specimens in the source images (or have not seen them in many years). Therefore, I have usually relied on the reported identifications, except that I have updated them to current nomenclature as in Fricke et al. (2024c). It is likely that some misidentifications are included. For example, the syntype of *Rubricatochromisletourneuxi* (Hemichromini) has 17 caudal vertebrae, whereas the three non-type specimens so identified all have only 13 (Suppl. material 2). Similarly, for *Coptodonzillii* (Coptodonini) in AMNH 229645, the unusual variation in counts — total vertebrae 28–31 (Suppl. material 2), anal pterygiophores 8–12 (Suppl. material 5) — may indicate the presence of more than one species in the lot. More prosaically, some lots may simply have been misidentified.

(2) Source image quality is quite variable. For the purposes of this study, the best images available to me have usually been either radiographs on fine-grain film, made for me at AMNH some 50 years ago or recent digital radiographs. Availability of online radiographs and microCT images or image stacks has enabled far more comprehensive taxonomic coverage than I initially planned, but again, image quality is variable. I elected to include data from images for which I might not be certain of one or two counts. My rationale was that, in a first survey such as this, it is best to include as much diversity as possible, trusting that the specimens can be re-imaged with better resolution (or the images re-examined by younger eyes) in doubtful cases. Usually the area of uncertainty in fuzzy low-resolution or grossly under- or overexposed online images is in the numbers of posterior DPt or APt or their precise insertion.

(3) Counts of total vertebrae should all be correct, but opinions on location of the first caudal vertebra might differ by one for some specimens. The first caudal centrum is that bearing the first haemal spine, but this can be difficult to determine. Occasionally, the first caudal centrum bears pleural ribs as well as a haemal spine (Fig. 2). From some multi-specimen images, I include data from only those specimens with sharpest detail, as noted in Suppl. material 12. Although I have tried to avoid or eliminate errors in the data, some of course may still occur.

## Data resources

The data underpinning the analysis reported in this paper are deposited in the Dryad Data Repository at https://doi.org/10.5061/dryad.k3j9kd5hx.

## Taxon treatments

### Pseudocrenilabrini

Fowler, 1934

C834BC53-AAA4-5002-B4EB-F53FCE63E95A

Division Acanthopterygii Artedi, 1738
Subdivision Percomorphacea Wiley & Johnson, 2010
Series Ovalentaria Smith & Near in Wainwright et al. (2012)
Order Blenniiformes Bleeker, 1859 (*sensu* Near and Thacker (2024))
Family Cichlidae Bonaparte, 1835
Subfamily Pseudocrenilabrinae Fowler, 1934
Tribe Pseudocrenilabrini Fowler, 1934 (= Haplochromini Hoedeman, 1947)

#### Taxon discussion

Hoedeman’s proposal (in Hoedeman and de Jong 1947) of the subfamily name Haplochrominae and tribe name Haplochromini is valid according to Van der Laan et al. (2014):109. Nevertheless, as Dunz and Schliewen (2013) noticed (without formalising the correction), Pseudocrenilabrini Fowler, 1934 has priority as the senior synonym of the "haplochromine" tribe (because the type genera, *Pseudocrenilabrus* and *Haplochromis*, of both names are classified in this tribe). Therefore, **the older name Pseudocrenilabrini Fowler, 1934 has priority over Haplochromini and must be used for the "haplochromines**," despite the incorrect use of the junior synonym in numerous works, such as Trewavas (1983), Poll (1986), Eccles and Trewavas (1989), Salzburger et al. (2002), Takahashi (2003), Koblmüller et al. (2008), Takahashi and Koblmüller (2011), Henning and Meyer (2012), Meyer et al. (2015), Oliver (2016), Ahi and Sefc (2017), Altner et al. (2017), Schedel et al. (2019), Altner et al. (2020), Dierickx and Snooks (2020), Lein and Jordan (2021), Lichilín et al. (2021), Nam et al. (2021), Svardal et al. (2021), Astudillo-Clavijo et al. (2022), Kocher et al. (2022), Ricci et al. (2022), Singh et al. (2022), Tibihika et al. (2022), Duenser et al. (2023), Kundu et al. (2023), Muschick et al. (2023), Nakamura et al. (2023), Santos et al. (2023), Tuckett et al. (2023), Costa et al. (2024), and Ngoepe et al. (2024).

#### Infratribal groups of the Pseudocrenilabrini

Molecular phylogenetics has begun to clarify the composition and interrelationships of African cichlid subclades; e.g., Schwarzer et al. (2009), Sylvester et al. (2010), Dunz and Schliewen (2013), Meyer et al. (2015), Malinsky et al. (2018), Ronco et al. (2021). Even within the multitude of recently evolved pseudocrenilabrine species including the iconic species flocks of Lake Victoria and especially Lake Malawi, some monophyletic groups are becoming recognised (Blumer et al. 2024). Formal nomenclature, however, has lagged behind these advances, forcing now well corroborated subclades to be specified with awkward workarounds like “Lake Malawi flock group A” and “Lake Malawi flock group B” (Meyer et al. 1996) or "the so-called mbuna ... and the remaining 500 haplochromine species which are sometimes referred to as the non-mbuna" (Konings et al. 2024).

I take this opportunity to correct the status of a previously named tribe (Tropheini), formalise subtribal names for four pseudocrenilabrine subclades and list the remaining pseudocrenilabrine genera not yet phylogenetically placed.

### Pseudocrenilabrina

Fowler, 1934

90B08C91-D20A-5B99-AF0E-11772EDBAF71

#### Diagnosis

A distinct pseudocrenilabrine clade, the “*Pseudocrenilabrus* group” (Weiss et al. 2015, Schedel et al. 2019) is united by molecular characters discovered through analyses of both mitochondrial and nuclear DNA (Schedel et al. 2020). A single morphological synapomorphy further unites all members of the genera *Pseudocrenilabrus*, *Lufubuchromis* and *Palaeoplex*, namely possession at least in adult males of the “*Pseudocrenilabrus* blotch” (Schedel et al. 2020), a red or orange mark covering the distal tip of the soft anal fin. This fin lacks the ocellated egg-dummies of adult males in the Pseudotropheina and the various non-ocellated coloured spots or streaks of adult males in the Cyrtocarina and Rhamphochromina.

**Type genus**: *Pseudocrenilabrus* Fowler, 1934

**Included genera and additional species** as indicated by mitochondrial DNA data (Schedel et al. 2019): *Lufubuchromis* Schedel, Kupriyanov, Katongo & Schliewen, 2020; *Palaeoplex* Schedel, Kupriyanov, Katongo & Schliewen, 2020; and *Pseudocrenilabrus* Fowler, 1934; also *Haplochromismoeruensis* (Boulenger, 1899); *Orthochromismachadoi* (Poll, 1967); and four northern Zambian species of *Orthochromis*: *O.kalungwishiensis* (Greenwood & Kullander, 1994); *O.luongoensis* (Greenwood & Kullander, 1994); *O.katumbii* Schedel, Vreven, Manda, Abwe, Manda, & Schliewen, 2018; and *O.mporokoso* Schedel, Vreven, Manda, Abwe, Manda, & Schliewen, 2018.

#### Distribution

Widespread (*Pseudocrenilabrus* spp.) in northern, central, eastern and southern Africa; other species in northern Zambia, Lake Mweru and Angola (Cunene River).

#### Notes

Fowler (1934):462 proposed the subfamily name Pseudocrenilabrinae, whose stem Pseudocrenilabr- is therefore available for use in other family-group names, including subtribe, taking the same authorship and date. Tawil (2001) mentioned a tribe Pseudocrenilabrini and a subtribe Pseudocrenilabrina, without citing their author and date.

Vertebral and pterygiophore counts and most-frequent insertion patterns of the latter, are summarised in Tables 1, 2, but are not distinctive for this subtribe.

### Tropheina

Poll, 1986 (new rank)

7C43B893-DC32-5AD8-9BC9-D10FD85D7FE1

#### Diagnosis

According to Takahashi (2003): 379, “This tribe [now subtribe] is characterized exclusively by extensively granulated cycloid scales at midbody (granulations comprising irregularly arranged, variously shaped protrusions over almost entire exposed surface)”. Haefeli et al. (2024): 445 extended this diagnosis, noting the presence of “…additional scales with small ctenii along the midbody, increasing in numbers towards the ventral and posterior side”.

**Type genus**: *Tropheus* Boulenger 1898

**Included genera**: *Interochromis* Yamaoka, Hori, & Kuwamura, 1998; *Jabarichromis* Haefeli, Schedel, Ronco, Indermaur, & Salzburger, 2024; *Limnotilapia* Regan, 1920; *Lobochilotes* Boulenger, 1915; *Petrochromis* Boulenger, 1898; *Pseudosimochromis* Nelissen, 1977; *Shuja* Genner, Ngatunga, & Turner, 2022; *Simochromis* Boulenger, 1898; *Tropheus* Boulenger, 1898.

#### Distribution

Lake Tanganyika.

#### Notes

Multiple molecular phylogenetic studies have recovered the tropheines as a clade nested within Pseudocrenilabrini (Salzburger et al. 2005, Koblmüller et al. 2008, Takahashi and Koblmüller 2011, Dunz and Schliewen 2013, Meyer et al. 2015, Ronco et al. 2021). Thus it is logical to place the tropheines as subordinate within Pseudocrenilabrini and not as a separate tribe. Vertebral and pterygiophore counts and most-frequent insertion patterns of the latter, are summarised in Tables 1, 2, but are not distinctive for the Tropheina.

### Rhamphochromina

Oliver, 2024 new subtribe

C7743224-9D58-581E-ABAF-BDDF23BC009C

0B6816A1-6D72-42FC-9EEF-7D700DE324FB

#### Description

Sometimes called the Lake Malawi “pelagic subradiation”, this clade is distinguished most definitively by a large (> 3.5 million base pairs) inversion at astCal1.2 coordinates 4,788,189 to 8,315,641 bp on chromosome 20 in at least *Diplotaxodon* and *Rhamphochromis* spp.; the single *Pallidochromis* species has not yet been studied (Blumer et al. 2024: p. 24 of supplementary text). This inversion is absent in the other Lake Malawi subtribes, with one exception (see Notes, below). The subtribe is also distinguishable from other Lake Malawi "haplochromines" by the following, in combination: pelagic, mid-water to deep-living species with predatory facies preying on fishes or zooplankton (versus demersal or epibenthic species found over sandy, muddy or rocky substrates or habitats intermediate between these and with widely varying diet, often fishes or zooplankton, but also specialists on benthic invertebrates, epilithic or epiphytic algae or even fish scales and fins); all jaw teeth simple, unicuspid, those of the outer row widely spaced in most species (versus jaw teeth unicuspid, bicuspid or tricuspid, the outer jaw teeth usually more closely spaced); absence (loss) of the plesiomorphic dark vertical bars and usually also absence of a mid-lateral horizontal stripe, resulting in the flanks of individuals being plain silver, grey, blackish or countershaded with no distinct melanic markings, except for a thin mid-lateral stripe in *Rhamphochromisesox* and *R.* sp. “stripe” (versus distinct melanic patterns present, visible at least in the "fright pattern" and in females and non-breeding males; vertical bars usually present, often in addition to darker markings such as spots, longitudinal stripe(s) or an oblique stripe). Vertebral and pterygiophore counts and most-frequent insertion patterns of the latter, are summarised in Tables 1, 2, but do not separate members of Rhamphochromina from those of Cyrtocarina. However, non-overlapping counts of vertebrae posterior to those bracketing the last occupied dorsal insertion space separate Rhamphochromina from Pseudotropheina (10–13 and 6–9, respectively). Total vertebrae are 33–40 in Rhamphochromina versus 28–33 in Pseudotropheina, 29–35 in Cyrtocarina.

**Type genus**: *Rhamphochromis* Regan, 1922

**Included genera**: *Diplotaxodon* Trewavas, 1935; *Pallidochromis* Turner, 1994; *Rhamphochromis* Regan, 1922.

#### Distribution

Lakes Malawi, Malombe, Chilingali and Kingiri and the upper Shire River.

#### Notes

Turner et al. (2004) had already suggested, based on a mitochondrial phylogeny, that *Rhamphochromis* is monophyletic and that its sister group consists of *Diplotaxodon* plus *Pallidochromis*. The discovery by Blumer et al. (2024) of the shared chromosome 2 inversion largely confirms that suggestion, although *Pallidochromistokolosh* has not yet been examined. Subsequent to the divergence of *Rhamphochromis* and *Diplotaxodon* from their common ancestor, this inversion was apparently passed to the ancestor of the “benthic” subradiation (a part of Cyrtocarina) through hybridisation with a *Diplotaxodon* (Blumer et al. 2024). Thus the chromosome 20 inversion does not define a strictly monophyletic group. The other characteristics listed in the diagnosis, however, will distinguish Rhamphochromina from “benthic” species.

### Cyrtocarina

Oliver, 2024 new subtribe

2413D25B-1F80-56E6-88D2-7B6E2DB125B6

50DD6BC6-1F0D-423A-9B76-29BF12A3E78B

#### Diagnosis

A distinct clade within the Pseudocrenilabrini. No morphological synapomorphies have been discovered, including in the present study. Members of this subtribe can, however, be distinguished from those of Pseudotropheina by the squamation on the chest transitioning gradually (versus abruptly) in size to the larger scales of adjacent areas; by the ovaries of adult females which are of subequal size (versus with left ovary atrophied); and by the anal fin maculae of mature males, if not secondarily lost, being simple brightly coloured ovals or streaks not surrounded by narrow, transparent, depigmented rings (versus true oval ocellae, each surrounded by a depigmented ring that, at least to human observers, lends the marks a three-dimensional appearance resembling cichlid eggs). Cyrtocarins can be distinguished from members of the Rhamphochromina by expressing a melanin pattern including (although often not as the dominant elements of the pattern) from 4 to about 10 vertical dark bars on the flanks and caudal peduncle, often with a mid-lateral longitudinal stripe and variably a stripe on the upper flanks, at least as a "fright pattern" (versus no vertical bars and usually no mid-lateral stripe and with simple silvery, grey, blackish or countershaded pigmentation); oral teeth of outer row closely spaced (crowns of adjacent teeth separated by a tooth's width or less), their crowns unicuspid, bicuspid or rarely tricuspid (versus always unicuspid, widely spaced in most species); demersal or epibenthic over sand, mud, rocky or intermediate habitats (versus pelagic, mid-water to deep-living).

**Type genus**: *Cyrtocara* Boulenger, 1902

**Included genera**: *Alticorpus* Stauffer & McKaye, 1988; *Aristochromis* Trewavas, 1935; *Aulonocara* Regan, 1922; *Buccochromis* Eccles & Trewavas, 1989; *Caprichromis* Eccles & Trewavas, 1989; *Champsochromis* Boulenger, 1915; *Cheilochromis* Eccles & Trewavas, 1989; *Chilotilapia* Boulenger, 1908; *Copadichromis* Eccles & Trewavas, 1989; *Corematodus* Boulenger, 1897; *Ctenopharynx* Eccles & Trewavas, 1989; *Cyrtocara* Boulenger, 1902; *Dimidiochromis* Eccles & Trewavas, 1989; *Docimodus* Boulenger, 1897; *Exochochromis* Eccles & Trewavas, 1989; *Fossorochromis* Eccles & Trewavas, 1989; *Hemitaeniochromis* Eccles & Trewavas, 1989; *Hemitilapia* Boulenger, 1902; *Lethrinops* Regan, 1922; *Lichnochromis* Trewavas, 1935; *Mchenga* Stauffer & Konings, 2006; *Mylochromis* Regan, 1922; *Naevochromis* Eccles & Trewavas, 1989; *Nimbochromis* Eccles & Trewavas, 1989; *Nyassachromis* Eccles & Trewavas, 1989; *Otopharynx* Regan, 1920; *Placidochromis* Eccles & Trewavas, 1989; *Protomelas* Eccles & Trewavas, 1989; *Sciaenochromis* Eccles & Trewavas, 1989; *Stigmatochromis* Eccles & Trewavas, 1989; *Taeniochromis* Eccles & Trewavas, 1989; *Taeniolethrinops* Eccles & Trewavas, 1989; *Tramitichromis* Eccles & Trewavas, 1989; *Trematocranus* Trewavas, 1935; *Tyrannochromis* Eccles & Trewavas, 1989.

#### Distribution

Lake Malawi including its satellite lakes Chilingali and Chikukutu (Genner et al. 2007, Turner et al. 2019) and crater lakes Kingiri and Ilamba (Turner et al. 2019), as well as the upper Shire River, Lake Malombe and the middle Shire River.

#### Notes

Tawil (2001):78, 81, 83 mentioned a "sous-tribu des Cyrtocarina", but these mentions do not satisfy Code requirements for availability: for all new names published after 1999, Article 16.1 requires that they be indicated explicitly as intentionally new; Article 16.2 requires citation of the name of the type genus. Tawil's mentions of "Cyrtocarina" fail to satisfy these Articles. I had independently selected Cyrtocarina as the most logical and recognisable name for Lake Malawi’s large non-mbuna clade long before becoming aware of these invalid prior uses of this name.

Phylogenomic analysis of ultraconserved element (UCE) loci, based on a limited set of taxa, consistently resolved the Lake Malawi-endemic non-mbuna pseudocrenilabrines (other than *Rhamphochromis*) as a well-supported clade according to Hulsey et al. 2017 (also D. Hulsey in litt. 2-Oct-2022). However, the multiple single-nucleotide polymorphisms (SNPs) that would indicate monophyly at various UCE loci and would diagnose the subtribe have apparently not been specified, so are not yet usable diagnostic attributes.

There is evidence from chromosome inversions that introgression into some of the Cyrtocarina taxa from other lineages has occurred, including *Astatotilapia*, *Diplotaxodon* and, perhaps, *Pseudocrenilabrus* (Blumer et al. 2024 fig. 4; G. Turner, pers. comm.). Therefore, the evolution of this subtribe has been partly reticulate.

Vertebral and pterygiophore counts and most-frequent insertion patterns of the latter, are summarised in Tables 1, 2, but are not distinctive for this subtribe as a whole. Note that the genera *Diplotaxodon*, *Pallidochromis* and *Rhamphochromis*, previously classified with the above genera, are removed to a separate subtribe above.

### Pseudotropheina

Oliver, 2024 new subtribe

614F4285-C320-50CD-A23F-DE1C88CFED95

CFD4A72D-6606-47F1-B767-32C25B88D60F

#### Diagnosis

A distinct clade, the "mbuna," within the Pseudocrenilabrini. This subtribe is characterised in part by a single-nucleotide polymorphism (SNP) in a transcription factor, *irx1b*, of the WNT signalling pathway in the forebrain. In the Pseudotropheina (25 species screened in 11 genera), this nucleotide is fixed as T, versus C in both the Cyrtocarina (47 species in 25 genera) and Rhamphochromina (5 species representing all three genera) (details in Sylvester et al. (2010)). Despite repeated assertions, no definite morphological synapomorphies diagnosing the mbuna clade have been identified (see discussion in Oliver and Arnegard (2010)). However, several morphological characteristics can discriminate the members of this clade from those of the other two Lake Malawi subtribes, although not from certain riverine and Lake Victoria Pseudocrenilabrini. These attributes include: scales on the chest that are abruptly smaller than those of adjacent body regions (versus with gradual change in chest scale size); anal fin of males bearing true ocelli (prominent rounded yellow to orange spots bordered with a narrow transparent ring) in the posterior half of the fin (versus with variously-shaped coloured areas in anal fin that are never surrounded with a transparent ring); and left ovary atrophied (versus both ovaries of similar size; however, an atrophied left ovary is found also in some Rhamphochromina). The number of vertebrae posterior to those bracketing the last occupied dorsal insertion space completely separates the Pseudotropheina (with 6–9) from the Rhamphochromina (with 10–13), although members of these two subtribes are unlikely to be mistaken for each other.

**Type genus**: *Pseudotropheus* Regan, 1922

**Included genera**: *Abactochromis* Oliver & Arnegard, 2010; *Chindongo* Li, Konings, & Stauffer, 2016; *Cyathochromis* Trewavas, 1935; *Cynotilapia* Regan, 1922; *Genyochromis* Trewavas, 1935; *Gephyrochromis* Boulenger, 1901; *Iodotropheus* Oliver & Loiselle, 1972; *Labeotropheus* Ahl, 1926; *Labidochromis* Trewavas, 1935; *Maylandia* Meyer & Foerster, 1984; *Melanochromis* Trewavas, 1935; *Petrotilapia* Trewavas, 1935; *Pseudotropheus* Regan, 1922; *Tropheops* Trewavas, 1984.

#### Distribution

Lake Malawi and the upper Shire River (Ribbink et al. 1983).

#### Notes

Phylogenomic analysis, based on ultraconserved element (UCE) markers, also consistently resolves the mbuna as a highly-supported clade (McGee et al. 2016, Hulsey et al. 2017; D. Hulsey in litt. 2-Oct-2022), but the specific SNPs have evidently not been reported.

Hoedeman (in Hoedeman and de Jong (1947)) previously proposed a tribe Pseudotropheini, but this was published as a name only and, thus, under Article 13 of the Code is not available (Van der Laan et al. 2014: 109). Subsequently, Tawil (2001): 78, 81 mentioned a "sous-tribu des Pseudotropheina", but this too fails to satisfy certain requirements for availability: for all new names published after 1999, Article 16.1 requires that they be indicated explicitly as intentionally new; Article 16.2 requires citation of the name of the type genus. Tawil's uses of "Pseudotropheina" do not satisfy these requirements. I had independently selected Pseudotropheina as the most logical and recognisable name for Lake Malawi’s mbuna clade months before becoming aware of these invalid prior mentions of this name.

Vertebral and pterygiophore counts and most-frequent insertion patterns of the latter, are summarised in Tables 1, 2. Notably, the minimum number of anal pterygiophores (8) in the Pseudotropheina is lower than that of its larger sister clade Cyrtocarina in which the minimum is nine.

**Other Pseudocrenilabrini**

In addition to the above five subtribes, the Pseudocrenilabrini contains further genera whose phylogenetic positions within the tribe have not yet been resolved. These are:

1. The *Serranochromis* informal group (Eccles and Trewavas 1989, Greenwood 1993) with *Chetia* Trewavas, 1961; *Sargochromis* Regan, 1920; *Serranochromis* Regan, 1920; and *Pharyngochromis* Greenwood, 1979, all of which I consider to be genera *incertae sedis* in Pseudocrenilabrini, as no synapomorphy, whether morphological or molecular, has been identified;
2. Other nominal genera *incertae sedis* in Pseudocrenilabrini: *Allochromis* Greenwood, 1980; *Astatotilapia* Pellegrin, 1904; *Astatoreochromis* Pellegrin, 1904; *Ctenochromis* Pfeffer, 1893; *Cyclopharynx* Poll, 1948; most *Haplochromis* Hilgendorf, 1888 spp.; *Lithochromis* Lippitsch & Seehausen, 1998; *Mbipia* Lippitsch & Seehausen, 1998; *Neochromis* Regan, 1920; most *Orthochromis* Greenwood, 1954 spp.; *Paralabidochromis* Greenwood, 1956; *Pundamilia* Seehausen & Lippitsch, 1998; *Pyxichromis* Greenwood, 1980; *Schubotzia* Boulenger, 1914; *Schwetzochromis* Poll, 1948; and *Thoracochromis* Greenwood, 1979.

One hopes that further molecular phylogenetic studies will be able to elucidate the relationships of these genera, many of which may not be monophyletic. Eventually, if found to constitute a monophyletic group, many species in the *incertae sedis* genera could be placed in a pseudocrenilabrine subtribe Haplochromina Hoedeman 1947, which would be possible because *Haplochromis* and *Pseudocrenilabrus* would then be in different subtribes.

## Analysis

Within the African cichlids (Pseudocrenilabrinae), there is striking variation in the number of vertebrae, dorsal and anal pterygiophores and even supraneurals. Table 1 provides an overview of the ranges of these meristic counts observed within all tribes and subtribes of Pseudocrenilabrinae and, to a limited extent, within the other three cichlid subfamilies. Detailed species-level counts are presented in Suppl. materials 2, 3, 4, 5, 6, 7 (an introduction to all the data tables is provided in Suppl. material 1). Substantial variation also occurs in the insertion patterns of dorsal and anal pterygiophores between the neural and haemal spines, respectively. Selected features of these insertion patterns are summarised in Table 2 and are detailed at the species level in Suppl. materials 8, 9, 10. The complete raw data for all individual specimens are available in Suppl. material 11. The specimens I examined are listed in Suppl. material 12.

Molecular phylogenetic studies consistently (and, I would add, quite counterintuitively) identify the anguilliform, blenny-like Indo-Pacific reef fish genus *Pholidichthys*, with two species, as the sister clade of the Cichlidae, and the leaffishes (Polycentridae) as sister of this pair (Near and Thacker 2024). Therefore, in the results below, some description is provided of the relevant character states observed in those sister groups, especially the more cichlidlike polycentrids, along with those of cichlids.

**Figure 3.**
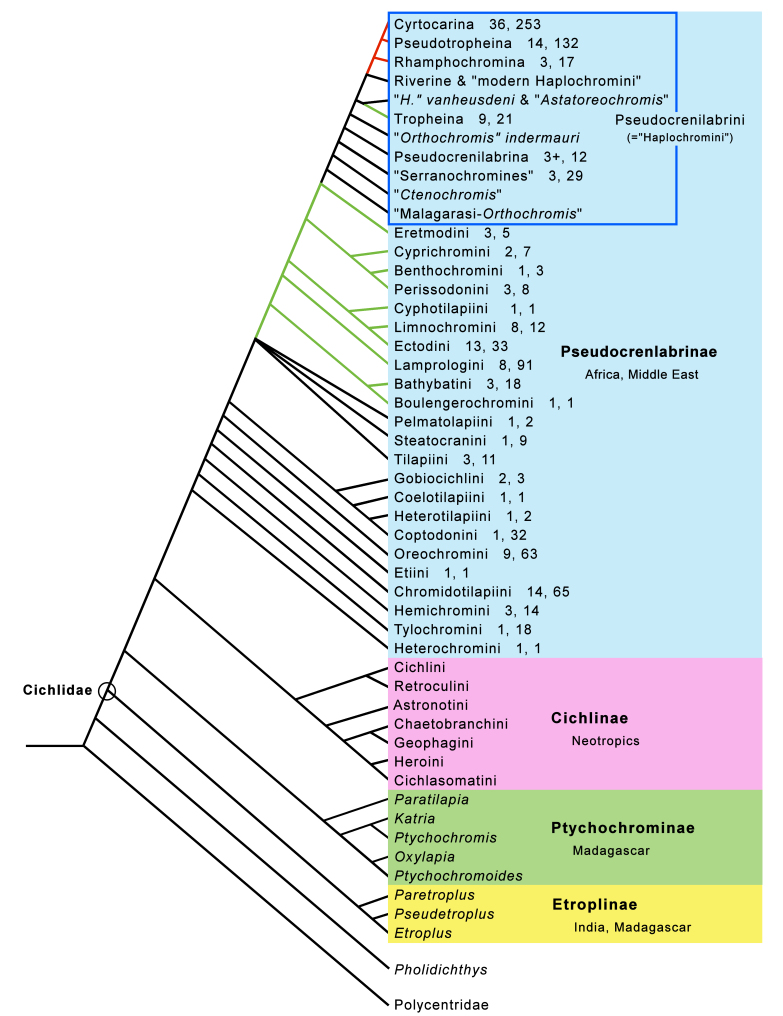
Cladogram attempting to summarise current hypotheses of the interrelationships of all cichlid subfamilies, tribes and subtribes (but, as Irisarri et al. (2018) noted, "While the monophyly of cichlid tribes is well established, their interrelationships remain hotly debated"). Green branches signify Lake Tanganyika tribes; terminal red branches, Lake Malawi subtribes of Pseudocrenilabrini. Pelmatochromines are included in Chromidotilapiini; *Trematocara* is included in Bathybatini. Numbers following tribal and subtribal names in Pseudocrenilabrinae indicate the number of currently valid genera and species in each clade. ”*H*” = “*Haplochromis*”. Compiled from trees in molecular and morphological phylogenetic studies, including Takahashi (2003), Sparks and Smith (2004), Stiassny and Sparks (2006), Smith et al. (2008), López-Fernández et al. (2010), Wainwright et al. (2012), Dunz and Schliewen (2013), Steele and López-Fernández (2014), Meyer et al. (2015), Ilves et al. (2018), Irisarri et al. (2018) Malinsky et al. 2018, Altner et al. (2020), Ronco et al. (2021), Schedel et al. (2019), and Blumer et al. (2024).

*Pholidichthys*, polycentrids, and cichlids together comprise the basal clade of the Order Blenniiformes *sensu* Near and Thacker (2024). A cladogram (Fig. 3) including *Pholidichthys*, polycentrids and all cichlid subfamilies, tribes and subtribes is given for reference in evaluating the data in the following sections.

### Vertebral counts

Counts of vertebrae are summarised in Table 1 and listed for each genus and species I examined in Suppl. material 2.

The nearest putative cichlid outgroup — apart from *Pholidichthys* — are the Asian and African leaffishes (Polycentridae). The few polycentrids I inspected, from three of the four genera, have 9–11 precaudal, 13–15 caudal and 22–26 total vertebrae, with three or four more caudal than precaudal centra (Table 1). The highly-specialised *Pholidichthys* has 22–26 precaudal, 48–56 caudal and 71–79 total vertebrae (Springer and Freihofer 1976).

Within the Cichlidae, the subfamily Etroplinae, with three genera found in Madagascar or India, is the most basal subclade. The nine specimens of these three genera that I examined have 12–16 precaudal, 14–19 caudal and 27–34 total vertebrae.

The Ptychochrominae (found in Madagascar), sister to the remaining two subfamilies, contains five genera. The 27 specimens I surveyed, including at least one specimen from each genus, have virtually identical ranges of vertebral numbers as in the Etroplinae: 12–16 precaudal, 14–19 caudal and 27–33 total vertebrae. The few Cichlinae (Neotropics) I examined have 12–20 precaudal, 11–19 caudal and 24–36 total vertebrae; these ranges likely underestimate the variation in this subfamily.

Low numbers of vertebrae are primitive for African cichlids (Pseudocrenilabrinae), as suggested by the low counts of vertebrae found in the Polycentridae, as well as in the Etroplinae and Ptychochrominae. Indeed, *Heterochromismultidens*, sister to all other members of the subfamily, has only 13 precaudal, 14 caudal and 27 total vertebrae in all 11 specimens examined. The other two pseudocrenilabrines that retain two supraneurals also have relatively compact vertebral columns; *Tylochromispolylepis* has 14–15 precaudal, 16–17 caudal and 31 total and *Etia* has 14 precaudal, 12 caudal and 26 total vertebrae.

The fewest precaudal vertebrae in African cichlids (Table 1) are found in certain Bathybatini and Lamprologini, both with a minimum of 11. The most occur in the Cyprichromini (21 found in one of five *Cyprichromiscoloratus* and three of five *C.pavo*). The fewest caudal vertebrae, 12, are found in certain Hemichromini, Chromidotilapiini, *Etia*, riverine Oreochromini and Coptodonini; the most, 25, in Ectodini, specifically in three of five *Enantiopusmelanogenys* examined. The next highest maximum is 22, in several tribes.

The shortest pseudocrenilabrine vertebral columns that I observed occur in the Hemichromini (25 total vertebrae in all four specimens of *Anomalochromisthomasi*) and Chromidotilapiini (25 in one of four specimens of ­*Divandualbimarginatus*).

A trend towards longer vertebral columns is evident in many tribes (Table 1). Total vertebral counts in the upper 30s occur in multiple tribes. I found a maximum of 40 in specimens of three different tribes: Bathybatini (in four of five *Bathybatesfasciatus*) and Cyprichromini (in one of five *Cyprichromispavo*), both from Lake Tanganyika and Pseudocrenilabrini, Rhamphochromina (in one of two *Rhamphochromisesox* of Lake Malawi).

Turner (1994): 378, 380 states that *Pallidochromis*, in the single specimen he checked, has only 15 + 17 = 32 vertebrae, as highlighted in the diagnosis of that new genus. The four *P.tokolosh* specimens available to me have 14–15 + 19 = 33–34 vertebrae (34 in three of the four), thus updating the diagnosis of *Pallidochromis*. Turner may not have included the urostyle in his count.

**Relative lengths of precaudal and caudal parts of the vertebral column.** I surveyed the relative lengths of pseudocrenilabrine precaudal versus caudal sections by tabulating the number of caudal minus precaudal centra (Table 1, Suppl. material 2). The greatest ranges of variation within a tribe occur in the Lamprologini (0–10) and Ectodini (1–11). Unusually long caudal relative to precaudal counts in the species I examined are seen in *Lamprologus* 'ornatipinnis congo' (caudal minus precaudal = 9 in four specimens and 10 in one) and *Lamprologus* 'ornatipinnis zambia' (caudal minus precaudal = 8 in one, 9 in six and 10 in two specimens). The lamprologines with the shortest caudal region relative to precaudal are *Chalinochromisbrichardi* with comparatively long precaudal section of 17 vertebrae and caudal minus precaudal = 0 in five of five specimens and *Variabilichromismoorii* also with a long precaudal section of 18 vertebrae and caudal minus precaudal = 0 in five and 1 in two specimens. The most extreme example of a relatively long caudal vertebral section that I found in any African cichlid, however, is in the ectodine *Enantiopusmelanogenys*, with 14 precaudal (five of five specimens), 24 (two of five) or 25 caudal (three of five) and 38–39 total vertebrae, for a caudal section 10 or 11 vertebrae longer than the precaudal count.

**Number of vertebrae posterior to last pterygiophore-hosting vertebrae.** I tallied how many vertebrae (including the terminal half-centrum) occur behind the most posterior dorsal insertion space with a pterygiophore and the corresponding count behind the last interhemal space with a pterygiophore (Table 1, Suppl. material 3). For brevity, these counts are referred to below as post-dorsal and post-anal vertebrae. These are not the same as the number of caudal vertebrae.

Amongst Polycentridae, *Afronandus* has six vertebrae behind the dorsal fin and eight behind the anal in the single specimen seen; *Monocirrhus* and *Polycentropsis* both have 3–4 post-dorsal and three post-anal vertebrae. Etropline cichlids have 4–6 post-dorsal and also 4–6 post-anal vertebrae. In the Ptychochrominae, there are 7–12 post-dorsal and 7–11 post-anal vertebrae. The Cichlinae I examined have 4–11 post-dorsal and 3–11 post-anal vertebrae. In the Pseudocrenilabrinae, the ranges are 4–13 vertebrae post-dorsally (4 in some Chromidotilapiini and Eretmodini, 13 in some Bathybatini and Pseudocrenilabrini, Rhamphochromina) and 5–14 post-anal vertebrae (5 in certain Chromidotilapiini, Coptodonini and Pelmatolapiini; 14 in Ectodini).

A few pseudocrenilabrine taxa are notable for having marked dorsal–versus–ventral asymmetry in these vertebral counts. To take one example, the four *Teleogrammabrichardi* (Chromidotilapiini) I examined have five (in three) or six (in one) post-dorsal, but nine (in two) or 10 (in two) post-anal vertebrae. Another example is *Asprotilapialeptura* (Ectodini), a species with an unusually long caudal peduncle, in which there are nine (in one), 10 (in three) or 11 (in one) post-dorsal vertebrae, but 13 (in one) or 14 (in four) post-anal centra. This asymmetry arises, of course, because the last anal-fin ray inserts markedly anterior to the last dorsal-fin ray in these species.

### Supraneurals

Counts of supraneurals are summarised in Table 1 and listed in detail in Suppl. material 8.

Supraneurals, called predorsals or predorsal interneurals in some older literature, are narrow bones in the sagittal plane between the supraoccipital crest and the spinous dorsal fin. They were long thought to be homologous with proximal dorsal pterygiophores (Smith and Bailey 1961). However, evidence from phylogenetic and ontogenetic comparison shows that supraneurals are not serial homologues of dorsal pterygiophores (Mabee 1988; Springer and Smith-Vaniz 2008) and are a distinct kind of structure.

*Pholidichthys* has no supraneurals (Springer and Freihofer 1976: fig. 9 and p. 38). Of the Polycentridae, *Monocirrhuspolyacanthus* has two (in two specimens) or three (in one) supraneurals. Equally, of the two *Polycentropsisabbreviata* available, one has two supraneurals and the other has three. The single *Afronandussheljuzhkoi* I saw has three supraneurals.

The Etroplinae consistently have two supraneurals in the nine specimens I saw, which (minimally) represent all three genera.

**Figure 4.**
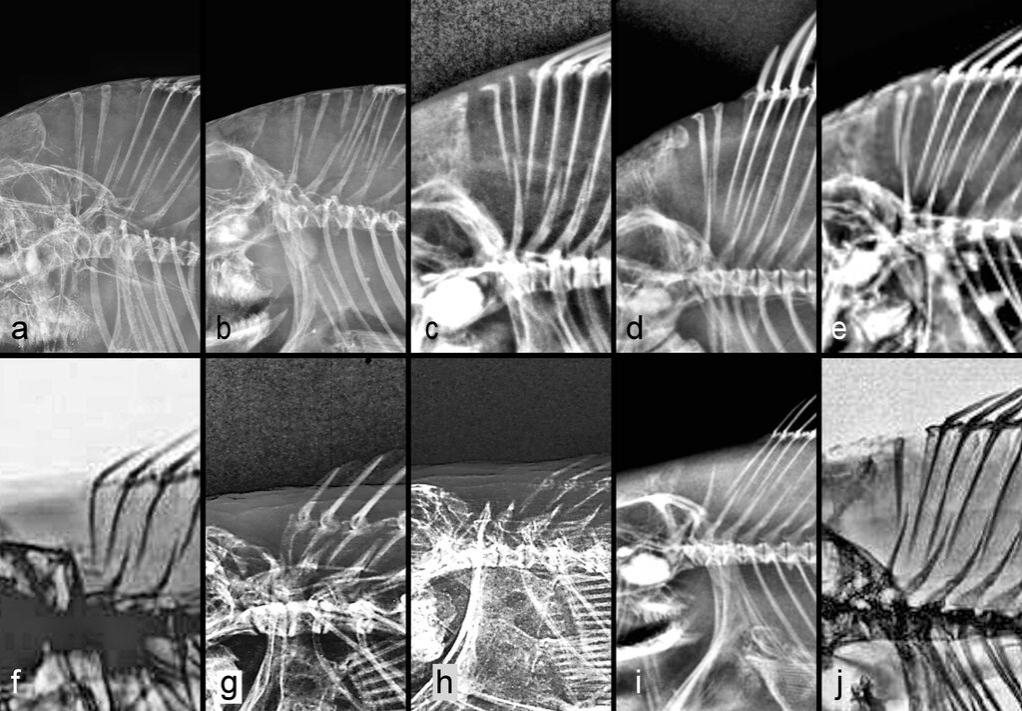
Examples of supraneural and pterygiophore insertion patterns in the first three dorsal insertion spaces in *Paratilapia* and species of pseudocrenilabrine Cichlidae. **a**, **b** *Paratilapiapolleni*, AMNH 11687 (Ptychochrominae), 0/0/2/ and 0/0-0/2/ (two of seven patterns observed in this species); **c** *Heterochromismultidens*, CU 88257 (Pseudocrenilabrinae, Heterochromini), 0/0-2/1/ (modal pattern); **d** *Tylochromispolylepis*, UNIBAS LID1 (Tylochromini), 0/0-2/1/ (modal pattern); **e** *Etianguti*, ZSM-PIS-029430 (Etiini), 0/0-2/1/ (modal pattern); **f** *Nanochromisnudiceps*, BMNH 1963.10.22.9 (Chromidotilapiini), –/2/1/ (one of two patterns observed in this species); **g** *Gobiocichlawonderi*, USNM 357037 (Gobiocichlini), –/2/1/ (modal pattern); **h** *Gobiocichlaethelwynnae*, USNM 229454, a paratype (Gobiocichlini), –/–/2/ (modal pattern); **i** *Bathybatesminor*, UNIBAS JEF7 (Bathybatini), –/1/2/ (modal pattern); **j** *Astatotilapiacalliptera*, BMNH 1893.11.15.1, lectotype (Pseudocrenilabrini), 0/2/1/ (modal pattern of this species and of this entire tribe). UNIBAS specimen images based on images on MorphoSource copyright by Fabrizia Ronco (see Suppl. material 12); used under CC BY-NC 4.0. Other source images copyright by the respective institutions of the specimens.

For the Ptychochrominae, exactly two supraneurals seem to have been reported (Stiassny and Sparks 2006, Sparks and Stiassny 2010), although the latter authors, in describing a new species *Ptychochromisernestmagnusi*, note that the “[p]osterior supraneural [is] often reduced in size *or absent*” (emphasis mine). *Katria* and *Oxylapia*, for each of which I examined a single specimen’s image, both have two supraneurals, but with different insertion patterns (Suppl. material 8). Likewise, in all three available specimens of *Ptychochromoidesbetsileanus*, I observed two supraneurals, but again with different insertion patterns. However, I found three supraneurals in 14 ptychochromine specimens — five of eleven *Paratilapiapolleni* (Fig. 4) and nine of eleven *Ptychochromisoligacanthus*. Thus, either a trend towards an increased number of supraneurals from two to three has occurred in the Ptychochrominae or the primitive number in the Ptychochrominae might be three, not two. Examination of additional specimens of Etroplinae as well as of Ptychochrominae might clarify matters. It is generally accepted that the primitive number of supraneurals in the Cichlidae is two (Cichocki 1976, Oliver 1984, Stiassny 1991, Schliewen and Stiassny 2003) and, furthermore, that the (only) derived condition with respect to that number is reduction to one or none, but the latter supposition needs to be revised.

Stiassny and Sparks (2006) recognised a ptychochromine clade composed of *Katria* and *Ptychochromis* based on their character 8. Uniquely in these two genera, “two supraneural elements are located anterior to the first neural spine”. I confirmed this pattern in the single *Katria* I was able to examine, but found it in only eight of eleven *P.oligacanthus*, the other three having a single supraneural anterior to the first neural spine and either one or two supraneurals (and two pterygiophores) between the first and second neural spines.

The Cichlinae I studied have two supraneurals most frequently, but some have none, one or three.

In the Pseudocrenilabrinae the number of supraneurals is primitively two (in *Heterochromis*, *Tylochromis* and *Etia*). As noted by Schliewen and Stiassny (2003), given the hypothesised phylogentic position of Hemichromini and Chromidotilapiini (the latter now including pelmatochromines), all with one supraneural, between *Tylochromis* and *Etia* (Fig. 3), it is uncertain whether the two supraneurals of *Etia* (Fig. 4e) are due to a reversal from one to two or whether the single supraneural of the intervening clades occurred independently of the reduction to one in the common ancestor of Oreochromini and the other more derived pseudocrenilabrines.

**Figure 5.**
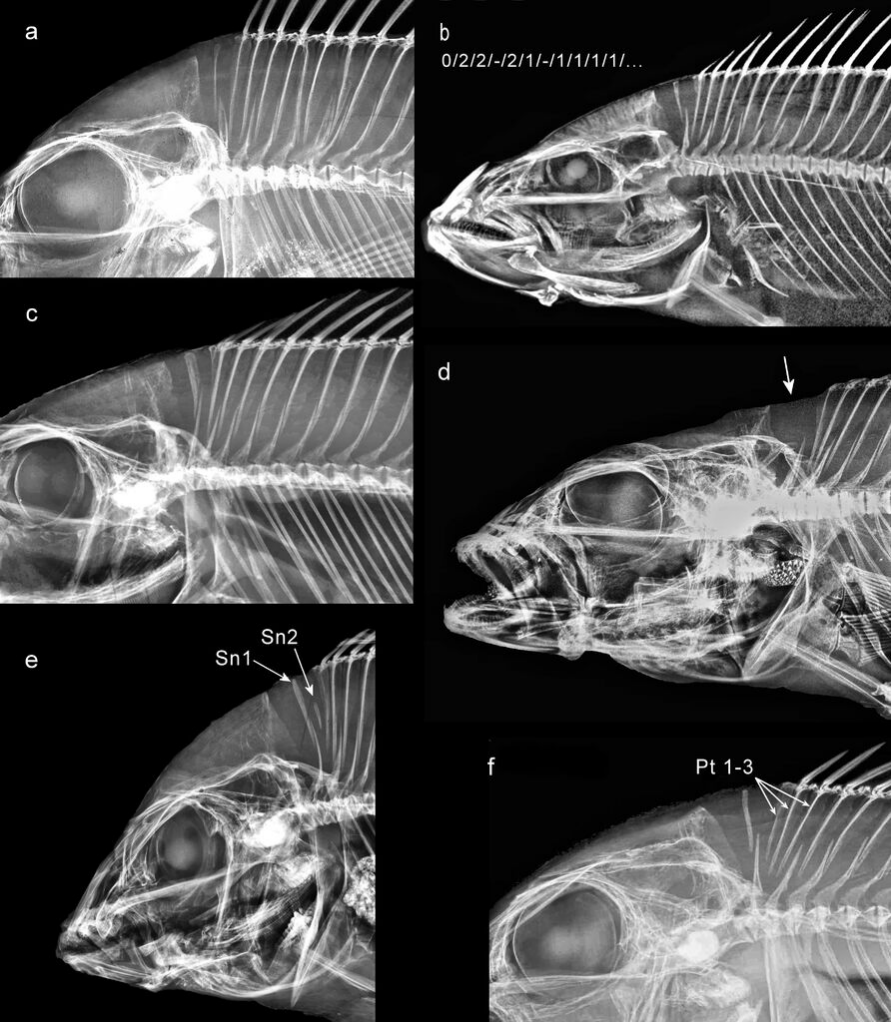
Examples of individual variant (non-modal for their species) insertion patterns of supraneural(s), anterior dorsal pterygiophores, or both, in Pseudocrenilabrini from Lake Malawi. **a** *Protomelasfenestratus*, AMNH 222022 (Cyrtocarina), with vertebral abnormality (centra 5+6 fused?), fourth dorsal fin spine supported by two pterygiophores and fifth insertion space with two pterygiophores; **b** *Exochochromisanagenys*, PSU 13376 (Cyrtocarina), unusual pattern of anterior pterygiophores with multiple occurrences of two or no pterygiophores per insertion space as indicated on image; **c** *Otopharynxovatus*, AMNH 31826 (Cyrtocarina), all anterior insertion spaces (after the first with its usual single supraneural) with a single pterygiophore, instead of the usual two in the second space; **d** *Sciaenochromisahli*, BMNH 1935.6.14.1639–1641 (Cyrtocarina), specimen with no supraneural (arrow points to expected location of a single supraneural); **e** *Trematocranusmicrostoma*, USNM 227923 (Cyrtocarina), specimen with a normal supraneural (Sn1) in first insertion space and a small atavistic second supraneural (Sn2) and two pterygiophores in second insertion space; **f** *Labidochromisvellicans*, YPM 014268 (Pseudotropheina), three pterygiophores (Pt 1–3) instead of normal two in second dorsal insertion space (note also that first pterygiophore lacks a fin spine). Source image of **b** courtesy of J. Stauffer; other source images copyright by the respective institutions of the specimens.

In most of the derived pseudocrenilabrines I examined, there is usually one supraneural (Table 2, Suppl. material 8). An exception is *Bathybates* (Bathybatini), in which some species (*B.graueri*, *B.vittatus*) have one, but others (*B.fasciatus*, *B.hornii*, *B.leo*, *B.minor*) have lost the supraneural, and one is variably present or absent in at least *B.ferox*. From the trees in figs. 1 and 3 of Kirchberger et al. (2012), it appears that both loss of the supraneural and its re-appearance may have occurred more than once in *Bathybates* (both of the other Bathybatini, *Hemibatesstenosoma* and *Trematocara* spp. have one supraneural). Supraneurals have also been independently lost several other times: in the Gobiocichlini, some Chromidotilapiini (17 of 17 *Limbochromisrobertsi*, two of four *Teleogrammabrichardi*, two of two *Nanochromisnudiceps*, two of two *N.parilus*), some Lamprologini (one of five *Telmatochromisdhonti*, three of five *T.vittatus*; but five of five *T.temporalis* have a supraneural) and rarely in Pseudocrenilabrini, Rhamphochromina (one of two *Rhamphochromisesox*) and Cyrtocarina (one of 16 *Sciaenochromisahli*, Fig. 5d).

I can also document the atavistic re-appearance of a second supraneural in a few pseudocrenilabrines. A small second supraneural occurs in one of two *Buccochromisnototaenia* (the holotype), one of four *Champsochromiscaeruleus*, one of three *Dimidiochromiskiwinge*, one of three *Docimodusjohnstoni* and one of nine *Trematocranusmicrostoma* (Fig. 5e) specimens examined. Intriguingly, amongst all pseudocrenilabrines other than *Heterochromis*, *Tylochromis* and *Etia* that I inspected, a second supraneural was found only in these five species, all of which belong to one of the three terminal pseudocrenilabrine subtribes, Cyrtocarina of Lake Malawi, phylogenetically distant from their basal two-supraneural relatives. I did not encounter any example in the sister of the Cyrtocarina, the Pseudotropheina (the mbuna of Lake Malawi). Kevrekidis et al. (2019) (also Kevrekidis 2020) did, however, find this two-supraneural state in some Tilapiini, remarking that “…on the order of 10% of the specimens of *Tilapiabaloni* Trewavas and Stewart, 1975 and *T.sparrmanii* Smith, 1840 studied show a reversion [from one] to two supraneural bones. In the specimens of *T.baloni* and *T.sparrmanii*, the second supraneural is much smaller than the first and placed posteroventrally to it” — exactly as I observed in the Lake Malawi taxa noted.

### Pterygiophore counts

**Relationship of pterygiophore count to number of fin rays.** In cichlids, the first two dorsal-fin spines are nearly always borne on two separate pterygiophores. The last two (uncommonly one or three) segmented rays are borne together on a single pterygiophore. Usually, therefore, there is one less dorsal pterygiophore than the total number of dorsal spines plus segmented rays.

In contrast to the dorsal fin, in the cichlid anal fin, the first two spines are nearly always borne together on a single pterygiophore that is clearly derived from the fusion of two separate cartilages (Penk et al. 2019:9), as observed by Kohno and Taki (1983) in *Oreochromisniloticus*. The plesiomorphic condition, in which the first two anal pterygiophores are separate struts, is seen in most specimens of the Indian etropline *Pseudetroplusmaculatus* in FMNH 17028. I also observed this condition in a cichline, *Chaetobranchopsisorbicularis* (UF 188930) and amongst pseudocrenilabrines in one specimen each of *Altolamprologus* 'compressiceps shell' (UNIBAS IRI7) and *Lamprologus* 'ornatipinnis zambia' (UNIBAS JDH6). The last anal pterygiophore typically supports both of the terminal two segmented rays, although rarely it may support one or three rays. Usually, therefore, in pseudocrenilabrines, there are two fewer anal pterygiophores than the total number of anal fin spines and soft rays.

**Dorsal pterygiophore count** (Table 1, Suppl. material 4). The Polycentridae I examined have 22–26 dorsal pterygiophores. In the basal cichlid subfamilies, the number ranges from 25–33 (Etroplinae) or 21–25 (Ptychochrominae). In the few Cichlinae I studied, the range is 21–32. The common ancestor of the African cichlids (Pseudocrenilabrinae) evidently had a relatively long dorsal fin and correspondingly numerous pterygiophores, judging by dorsal pterygiophore counts of 27–28 in *Heterochromis* and 28 in *Tylochromis* (represented by *T.polylepis*). *Etia* has 24 in all six available specimens. More-derived subclades have a wide range of counts, from a low of only 20 in the Bathybatini (in three of five *Trematocarazebra*) to a high of 32 found in Gobiocichlini (one of 14 *Gobiocichlawonderi*), Bathybatini (three of five *Bathybatesfasciatus*) and Perissodini (one of five *Plecodusparadoxus*).

**Longest consecutive series of a single dorsal pterygiophore per dorsal insertion space** (Table 1, Suppl. material 7). The Polycentridae examined have short series, only 4–10 DIS long. In etropline cichlids I found 15–18. The Ptychochrominae have 13–15, except for *Paratilapiapolleni*, in eleven specimens of which every count from eight to 13 is represented. Specimens of the seven tribes of Cichlinae have counts of 12–22, most commonly 13–16 (apart from one atypical *Cichlaorinocensis* with only six, the full series being interrupted by an empty DIS).

In the basal tribes of Pseudocrenilabrinae with two supraneurals, *Heterochromis* has 10–13 consecutive DIS with a single DPt each; *Tylochromispolylepis* has 15–17; *Etia* has 14 in all six specimens. The remaining groups have counts ranging from a low of 12 in a few tribes to a maximum of 28 in one of fourteen individuals of *Gobiocichlawonderi* (Gobiocichlini).

**Anal pterygiophores anterior to first haemal spine** (Table 1, Fig. 7, Suppl. material 5). In the Polycentridae, 1 - 3 pterygiophores insert in front of the first haemal spine. Within etropline cichlids, the single *Paretropluspolyactis* has two, the one *Etroplussuratensis* has five and the seven *Pseudetroplusmaculatus* (Fig. 7a) have 5–8; in the Ptychochrominae, there are one or two in this position. The Cichlinae I examined have one (usually) or two, except for *Hypselacaracoryphaenoides* (Heroini) in which four of five specimens have two, but one has none anterior to the first haemal spine.

In the Pseudocrenilabrinae, *Heterochromis* has one in ten specimens and two in one; *Tylochromispolylepis*, 1 (six specimens); and *Etia*, one in five and two in one. Therefore, it appears that the pseudocrenilabrine common ancestor most likely had a single anal pterygiophore in front of the first haemal spine or may likewise have variably had one or two. In the Pseudocrenilabrinae as a whole, one is the strongly modal number, but two is not uncommon and, more rarely, there is no pterygiophore anterior to the first haemal spine.

Increased numbers inserting anterior to the first haemal spine have arisen several times. In Lamprologini, the modal number is two, but counts of three occur in at least seven species and four occur in this position in *Altolamprologuscalvus* (Fig. 7h; five of five specimens), *Lamprologuscallipterus* (one of ten) and *Variabilichromismoorii* (Fig. 7e; seven of seven). In the Ectodini, one is the modal count, but two is common and three occurs in five species studied, in one of which, *Enantiopusmelanogenys*, it is found in five of five specimens.

**Figure 6.**
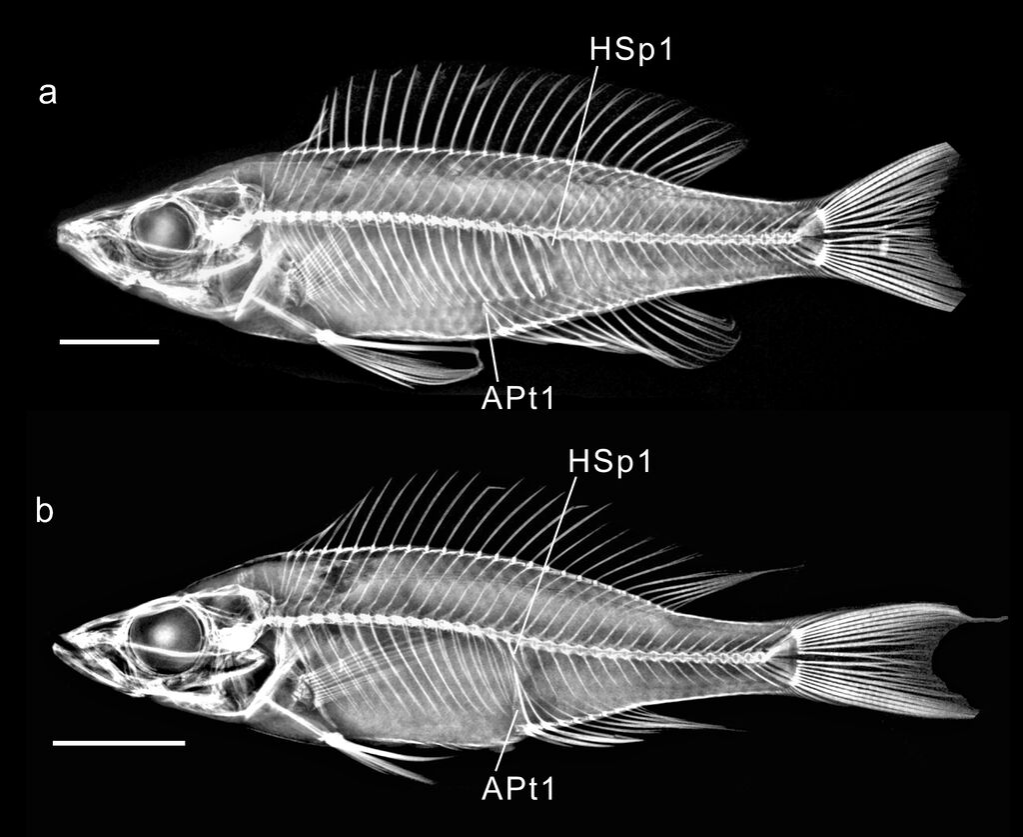
**a** *Cyprichromisleptosoma* (UNIBAS ITE9); **b** *Paracyprichromisnigripinnis* (UNIBAS GPD8). In **a**, note the forward location of multiple abdominal anal pterygiophores (seven in this individual) inserting towards consecutive pleural ribs, a unique autapomorphy of all *Cyprichromis* spp. In **b**, three anal pterygiophores insert anterior to and adjacent to the first haemal spine. Abbreviations: APt1, first anal pterygiophore; HSp1, first haemal spine. Figure derived from images on MorphoSource copyright by Fabrizia Ronco (see Suppl. material 12); used under CC BY-NC 4.0. Scale bars = 1 cm.

**Figure 7.**
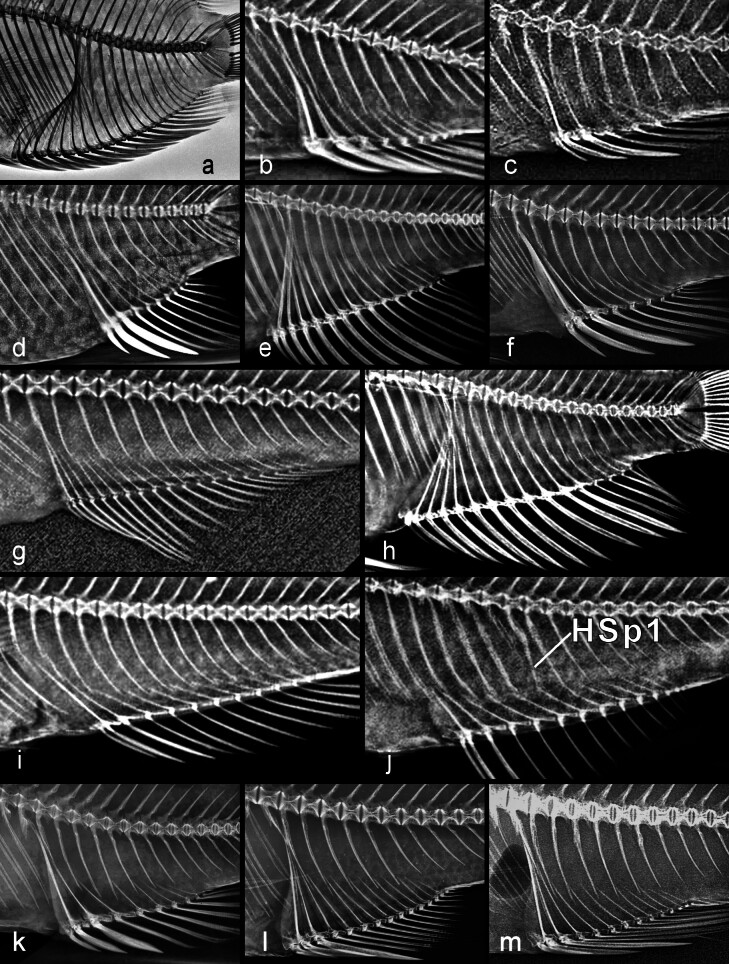
Examples of anal-fin pterygiophore insertion patterns in *Pseudetroplus* and in species of Pseudocrenilabrinae. **a** *Pseudetroplusmaculatus*, MCZ 4311 (Etroplinae), 5\–\2\1\2\1\1\2\2\2\1\ (5 Pt in first insertion space anterior to HSp1 [compare *Cyprichromis* condition in j below], 19 total Pt); **b** *Rubricatochromisletourneuxi*, CUMV 94558 (Hemichromini), 1\1\1\1\2\2\1\ (1 Pt anterior to HSp1, 9 total Pt); **c** *Congochromisrobustus*, RMCA 135706, holotype (Chromidotilapiini), 1\–\1\2\1\1\ (1 Pt anterior to HSp1, 6 total Pt, the lowest count found in any African cichlid); **d** *Sarotherodonmelanotheron*, UF 91814 (Oreochromini), 1\–\1\2\1\3\1\ (1 Pt anterior to HSp1, 9 total Pt); **e** *Variabilichromismoorii*, UNIBAS IYC8 (Lamprologini), 4\1\1\2\1\1\2\1\1\ (4 Pt anterior to HSp1, 14 total Pt); **f** *Abactochromislabrosus*, YPM 021602 (Pseudocrenilabrini, Pseudotropheina), 1\1\–\1\1\1\3\ (1 Pt anterior to HSp1, 8 total Pt; this total count is evidence that this species is correctly placed in the mbuna clade Pseudotropheina, as members of its sister clade Cyrtocarina invariably have ≥ 9 total anal Pt); **g** *Bathybatesfasciatus*, UNIBAS KYD1 (Bathybatini), 1\2\3\2\1\2\2\2\2\1\ (1 Pt anterior to HSp1, 18 total Pt, the highest count found in any African cichlid, seen only in this and a few other *Bathybates* spp.); **h** *Altolamprologuscalvus*, UNIBAS IOC5 (Lamprologini), 4\1\1\1\1\1\1\1\1\1\2\ (4 Pt anterior to HSp1, 15 total Pt); **i** *Xenotilapiasima*, UNIBAS LBE9 (Ectodini), 1\1\1\1\1\1\1\1\1\1\1\ (1 Pt anterior to HSp1, 11 total Pt); **j** *Cyprichromis* sp. ‘jumbo’, UNIBAS MOD8 (Cyprichromini), 2 1 1 1 – – \2\1\1\2\1\ (no Pt in first two insertion spaces anterior to HSp1; an underlined numeral indicates for each position the number of abdominal (precaudal) Pt inserting between successive pairs of pleural ribs [compare etropline condition in a above], 12 total Pt); **k** *Iodotropheussprengerae*, USNM 207015, paratype (Pseudocrenilabrini, Pseudotropheina), 1\1\–\1\–\1\2\2\ (1 Pt anterior to HSp1, 8 Pt total); **l** *Diplotaxodonargenteus*, AMNH 221945 (Pseudocrenilabrini, Rhamphochromina), 3\1\2\2\2\3\ (3 Pt anterior to HSp1, 13 total Pt); **m** *Pallidochromistokolosh*, YPM 026900 (Pseudocrenilabrini, Rhamphochromina), 2\1\1\2\1\2\3\ (2 Pt anterior to HSp1, 12 total Pt). UNIBAS specimen images based on images on MorphoSource copyright by Fabrizia Ronco (see Suppl. material 12); used under CC BY-NC 4.0. Other source images copyright by the respective institutions of the specimens.

In Cyprichromini the count ranges from 0 - 7, with a remarkable, unique arrangement in *Cyprichromis*. According to Barel et al. (1977):350, writing about Lake Victoria pseudocrenilabrines, “Topographically the first caudal vertebra is the one toward which the first anal pterygiophore points”. This is likely true for all pseudocrenilabrine cichlids, with the single known exception o*f Cyprichromis*. I found that all studied specimens of seven *Cyprichromis* species have an increased number (5, 6 or 7) of anal pterygiophores inserting anterior to the first haemal spine, all of which are separate, directed towards several consecutive precaudal vertebrae and inserting towards or between successive pairs of pleural ribs (Figs 6, 7j; Suppl. material 11). One specimen of *C.coloratus* (UNIBAS JEC7) even has more pterygiophores in front of (7) than behind (6) the first haemal spine. This striking character state of *Cyprichromis* is not found in its sister clade, *Paracyprichromis*, all examined species of which have 0 to 3 anterior pterygiophores that follow the normal pattern by inserting along the anterior edge of the first haemal spine (Fig. 6b; Suppl. material 11). Poll (1986):144–145 made no mention of this unique character state in *Cyprichromis*. It was also not mentioned by Takahashi (2004), who stated (p. 4): “Regarding *Cyprichromis*, only the elongated swim bladder [amongst characters he studied] is valid as a synapomorphy supporting the genus…”. To that character can now be added this second synapomorphy of abdominal anal pterygiophores, which may be somehow related to the previously known, exceptionally elongated swim bladder in this genus, which extends to above the anterior one-third of the anal fin.

Although the South Asian genus *Pseudetroplus* (Etroplinae) and other etroplines also have an increased number, 4–8, of anal pterygiophores anterior to the first haemal spine (Pethiyagoda et al. 2014; this study), this is a completely different condition from that seen in *Cyprichromis*. In etroplines, the anterior pterygiophores are differently configured, the tips of all 4–8 pointing towards the tip of the first haemal spine (Fig. 7a; Pethiyagoda et al. (2014): figs. 1A_1_–1A_3_).

In the Lake Malawi Pseudocrenilabrini, species of both Pseudotropheina and Cyrtocarina overall have a strongly modal number of 1 anal pterygiophore anterior to the first haemal spine (Suppl. material 5), although two occurs as a variant in many species. *Mchenga* sp. is an exception, with a strong mode of two (in 19 of 23 specimens). Apart from occasional individual variants with three throughout the riverine and lacustrine Pseudocrenilabrini, counts above two are consistently seen in *Diplotaxodon*. In this genus of Rhamphochromina, all studied species frequently have an elevated count with *D.argenteus* having two (in three) or three (in seven, Fig. 7l) specimens; *D.ecclesi* with three (in the holotype, the only specimen seen); *D.greenwoodi* with three (again in the holotype and only specimen examined); and *D.limnothrissa* with one (in two), three (in six) or four (in two). This strong trend towards numbers of pre-anal pterygiophores being increased above two is a newly-recognised derived attribute of *Diplotaxodon*. *Rhamphochromis* has been suspected to be closely related to *Diplotaxodon* and *Pallidochromis* (Turner et al. 2004). These three genera now constitute the subtribe Rhamphochromina. The five *Rhamphochromis* specimens total that I examined from three different species have 0 (in one), one (in one) or two (in three) pre-anal pterygiophores; thus, the trend towards increase seems to be absent in this genus. *Pallidochromistokolosh* (Fig. 7m) has two pre-anal pterygiophores in all four specimens examined.

**Anal pterygiophore total count** (Table 1, Suppl. material 5). The Polycentridae examined range from 9–24 anal pterygiophores. Amongst cichlids, the Etroplinae sampled (Fig. 7a) have 18–22; the Ptychochrominae, 9–12; and the Cichlinae, 8–21. In the Pseudocrenilabrinae, I found *Heterochromis* to have 10–11; *Tylochromispolylepis*, 8–9; and *Etia*, 9. The overall numbers in this subfamily range from a low of six in the holotype of *Congochromisrobustus* (Chromidotilapiini; Fig. 7c) to a high of 18 in several *Bathybates* spp. (Bathybatini; Fig. 7g).

Anal pterygiophore counts provide the solution to a vexing problem. Of the hundreds of pseudocrenilabrines endemic to Lake Malawi, in three endemic sister clades, only one species has been difficult to place with confidence in one or the other clade. *Abactochromislabrosus* (Trewavas 1935) was assigned to the mbuna group (i.e. Pseudotropheina) when first described and for several decades thereafter. Some writers, however (listed in Oliver and Arnegard (2010)), suspected it might instead belong in the "hap" or demersal non-mbuna group (i.e. Cyrtocarina). There has been no definitive resolution, as no known morphological character unambiguously distinguishes one group from the other; no (non-molecular) synapomorphy has been found for either clade (see discussion in Oliver and Arnegard (2010)). In the present study, I found that the total number of anal pterygiophores in the Pseudotropheina is 8–10 (mode 9); in the Cyrtocarina, it is 9–13 (mode 10). Of six *A.labrosus* specimens, five have eight (Fig. 7f) and one has nine. Not a single one of the 425 specimens of Cyrtocarina species I examined has as few as eight anal pterygiophores, whereas 62 of 219 Pseudotropheina specimens have eight (e.g. Fig. 7k). Although a morphological synapomorphy for the mbuna is still lacking, this finding is compelling evidence corroborating the placement of *Abactochromislabrosus* in the mbuna clade, Pseudotropheina, as was tentatively suggested by Oliver and Arnegard (2010).

### Pterygiophore insertion patterns

The insertion patterns of the dorsal and anal pterygiophores vary intraspecifically in most species. Nonetheless, some informative differences do occur between species, subtribes and tribes. There is a large range of variation in both vertebral column length and number of dorsal- and anal-fin spines and rays across pseudocrenilabrine clades. However, the long mid-section of the dorsal pterygiophore series is normally composed monotonously of a single pterygiophore in every insertion space. For this reason, in comparing patterns, I found it expedient to concentrate on the three areas with the most informative variation, namely the first three and last four dorsal insertion spaces (DIS) and the last four interhaemal spaces (IHS). The most frequent patterns found in each tribe and subtribe are summarised in Table 2, while Suppl. materials 8, 9, 10, respectively, display all insertion patterns found in these three areas for all species examined. The raw data, including the complete insertion patterns of all individual specimens, may be found in Suppl. material 11.

**First three occupied dorsal insertion spaces**: All three species of Polycentridae I inspected have insertion patterns of the first three DIS that I did not find in any cichlid (Suppl. material 8). One of two *Polycentropsisabbreviata* has 0/0-1/2/, a pattern found also in a few cichlid specimens; the other has 0/0/0-1/, seen in no cichlid, but also observed in one of two *Monocirrhuspolyacanthus*. The second *M.polyacanthus* with 0-0/0-1/1/ and the single *Afronandussheljuzhkoi* with –/0/0-1/ both have insertion patterns not seen in any cichlid.

In the Etroplinae, I observed two patterns. The more frequent one is 0/0-1/1/ seen in the single available *Etroplussuratensis*, five of seven *Pseudetroplusmaculatus* and the lone *Paretropluspolyactis*. Elsewhere in the Cichlidae, I found this pattern only in one of eleven *Paratilapiapolleni* (Ptychochrominae), two of three *Astronotusocellatus* (Cichlinae) and one of three *Dimidiochromiskiwinge* (Pseudocrenilabrini: Cyrtocarina). The other two *Ps.maculatus* have 0-0/1/1/, an arrangement not found in any other cichlid in this survey.

The Ptychochrominae display 13 different insertion patterns across 27 specimens. The single *Katriakatria* has 0-0/1/2/, observed in no other cichlid. *Oxylapiapolli*, again a single specimen, has 0/0/2/, found in two other ptychochromine species, but in no other cichlid. Eleven specimens of *Paratilapiapolleni* show seven different patterns; eleven of *Ptychochromisoligacanthus* have four patterns. Two of three *Ptychochromoidesbetsileanus* have 0/–/0-1/, another insertion pattern seen in no other cichlid.

In the Pseudocrenilabrinae, I found 20 different insertion patterns (counting, as always, every observed variant) for the first three DIS. These three spaces in pseudocrenilabrines may include a total of 0, 1 or 2 supraneurals (normally 1) and 2, 3 or 4 pterygiophores. Eight of the 20 pseudocrenilabrine patterns include an empty insertion space, which may occur in the first, second or third position. In one of these patterns, both first and second positions are empty (–/–/2/, in 9 of 11 *Gobiocichlaethelwynnae* (Fig. 4h).

Of the three genera retaining two supraneurals, *Heterochromis* has 0/0-2/1/ (in 10 specimens; Fig. 4c) or 0-0/2/1/ (in one); *Tylochromis* has 0/0-2/1/ (in six of six specimens; Fig. 4d); and *Etia* has 0-0/2/1/ (in five specimens; Fig. 4e) or 0/0-2/1/ (in one). Thus, all of these basal pseudocrenilabrines have two supraneurals, followed by two pterygiophores in the second insertion space and one in the third space, but some have both supraneurals before the first neural spine (in the first DIS), whereas others have the second supraneural after the first neural spine (in the second DIS). Notably, in the Neotropical subfamily Cichlinae, in five of the seven tribes, the few taxa and specimens I examined share the 0/0-2/1/ pattern. The excepted tribes are Astronotini and Cichlini (Suppl. material 8).

Current molecular phylogenetic hypotheses of pseudocrenilabrines place the Hemichromini and Chromidotilapiini, the latter including pelmatochromines, consecutively after both *Heterochromis* and *Tylochromis*, but basal to *Etia* (Fig. 3). Yet, unlike *Etia*, all members of Hemichromini and Chromidotilapiini have lost the second supraneural (and also the first supraneural in certain members of the latter tribe — at least *Nanochromisparilus*, one of two *N.nudiceps* and *Teleogrammabrichardi*; Suppl. material 8). As already noted above, this morphological evidence in isolation would suggest that *Etia* is misplaced and might belong immediately after *Heterochromis* and *Tylochromis*, a possibility discussed by Schliewen and Stiassny (2003). However, molecular phylogenetic evidence suggests otherwise (Dunz and Schliewen 2013).

The dominant 0/2/1/ insertion pattern, i.e. a single supraneural in the first dorsal insertion space, two pterygiophores in the second and one pterygiophore in the third, is found only in the Pseudocrenilabrinae and, I would argue, points to a clear-cut synapomorphy of all African cichlids more terminal in position than *Etia* (Table 2). The other 19 patterns found in this subfamily are either individual variants or more-apomorphic developments. Note, however, that the 0/2/1/ pattern is easily produced from patterns seen even in the Ptychochrominae and Cichlinae by the loss of one or two supraneurals. Thus, the loss of the second supraneural is the actual synapomorphy, as also noted, before the discovery of *Etia*, by Stiassny (1990) in her fig. 3 character 12.

Although 0/2/1/ is the dominant pseudocrenilabrine pattern (Fig. 4j), exceptional taxa with other patterns do exist (Suppl. material 8). This pattern is secondarily modified in Gobiocichlini by loss of the supraneural (Fig. 4g, h). Amongst Lake Tanganyika taxa, the Bathybatini examined all lack the 0/2/1/ pattern. *Bathybatesferox* (9 of 10 specimens), *B.graueri*, *B.vittatus*, *Hemibatesstenosoma*, *Trematocaraunimaculatum* and *T.zebra* all have 0/1/2/; in other words, the second pterygiophore has been displaced from the second to the third DIS (or, alternatively, the tip of the second neural spine in these taxa has shifted forward by one pterygiophore). In contrast, *Bathybatesfasciatus*, the single exceptional *B.ferox*, *B.hornii*, *B.leo*, *B.vittatus* and most *B.minor* (Fig. 4i) all appear to have lost the supraneural, giving the unusual pattern –/1/2/. In the Cyprichromini, all seven *Cyprichromis* spp. modally have 0/1/2/, whereas the three *Paracyprichromis* species studied have 0/2/1/. Most of the Lamprologini examined have 0/2/1/, but *Lamprologuscallipterus* has 0/1/2/ (9 of 10 specimens). In the Limnochromini, the only genus not showing the pattern 0/2/1/ is *Baileychromis*; *B.centropomoides* (4 of 5 specimens) has 0/1/2/.

Amongst Lake Malawi pseudocrenilabrines, all taxa examined in the Pseudotropheina and nearly all in the Cyrtocarina modally have the typical 0/2/1/ pattern (Suppl. material 8). The 0/1/2/ variant is more frequent in the Cyrtocarina (5.9%) than in the Pseudotropheina (2.7%). Of the open-water predators (Rhamphochromina), *Diplotaxodonargenteus* and *D.limnothrissa* modally have 0/1/2/ and 2 of 4 *Pallidochromistokolosh* specimens have 0/1/3/. *Rhamphochromis* also departs from the 0/2/1/ norm, with only one of five total specimens having this pattern (the holotype of *R.brevis*; a second specimen of this species has 0/1/2/). The single *R.woodi* I examined has 0/–/3/; the two *R.esox*, 0/1/2/ and –/2/1/. *Diplotaxodon* and *Pallidochromis* tend to have the second DIS empty (seen in 6 of 19 *Diplotaxodon* specimens of three species, in 2 of 4 *Pallidochromis* specimens and in no other Lake Malawi cichlid studied, except the *R.woodi* specimen already noted). This unusual shared tendency is consistent with *Diplotaxodon* and *Pallidochromis* being sister taxa (a possibility raised by Turner et al. (2004), based on a mitochondrial phylogeny) and with findings of Hulsey et al. 2013 (fig. 2), based on the complete mitochondrial genomes of these fishes.

**Last four occupied dorsal insertion spaces**: The few polycentrids I examined have four insertion patterns, three of which are also found in cichlids. The fourth, /2/3/2/2/ (in one of two *Monocirrhuspolyacanthus*), did not occur in any cichlid I examined.

In specimens of Etroplinae, I found four insertion patterns for the last four occupied DIS, all of which also occur in the Pseudocrenilabrinae.

In the Ptychochrominae, all eight patterns found are, again, also common to the Pseudocrenilabrinae.

The Cichlinae studied have a total of only eight distinct insertion patterns, the modal one being /1/2/2/2/ which I found in all tribes except Astronotini and Retroculini, the most basal two of this subfamily. In the few specimens examined from those two tribes only /2/1/2/1/ happened to occur, a pattern seen also in Cichlasomatini and Geophagini.

**Figure 8.**
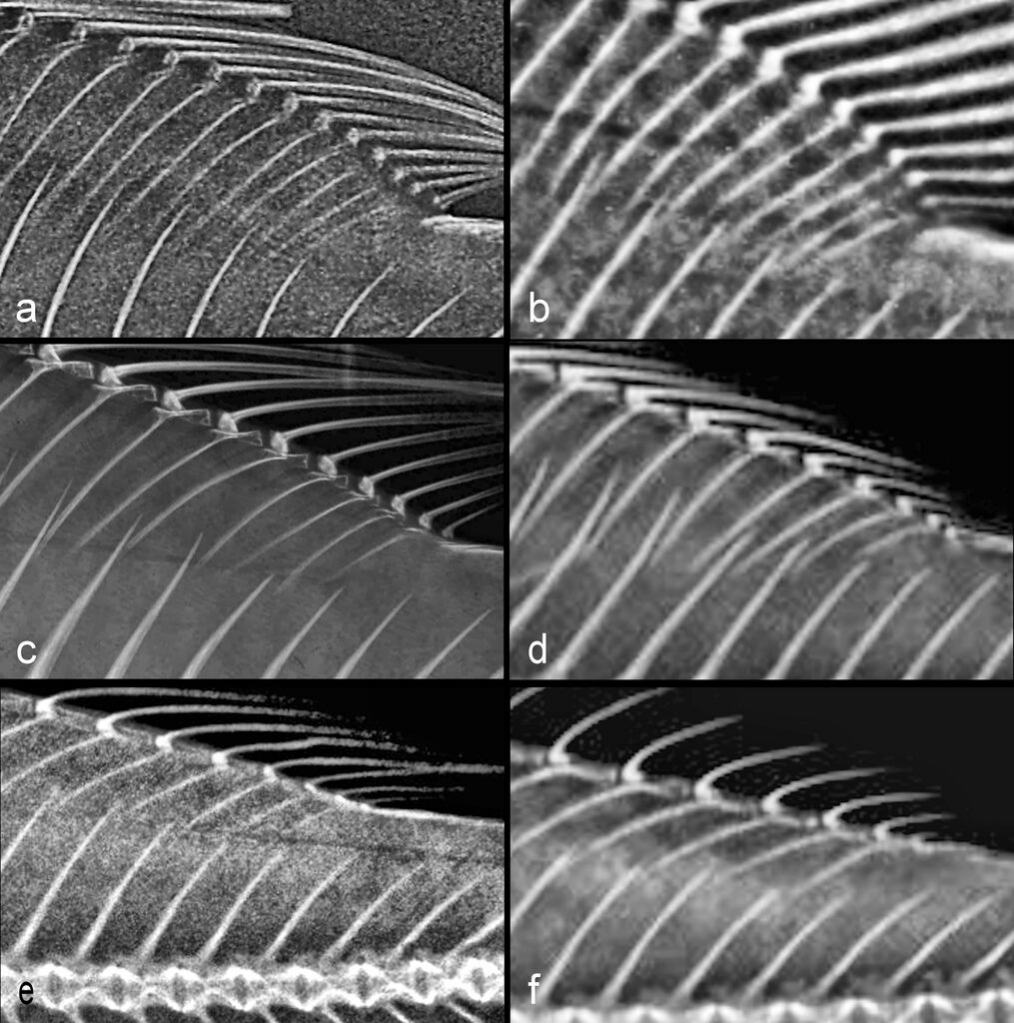
Examples of pterygiophore insertion patterns in the last four occupied dorsal insertion spaces in species of pseudocrenilabrine Cichlidae. **a** *Pelmatolapiamariae*, USNM 304008 (Pelmatolapiini), /2/2/2/3/ (9 total Pt, modal pattern); **b**,*Heterochromismultidens*, CU 88257 (Heterochromini), /2/2/2/2/ (8 total Pt, modal pattern); **c** *Dimidiochromiscompressiceps*, AMNH 31783 (Pseudocrenilabrini, Cyrtocarina), /2/1/2/2/ (7 total Pt, modal pattern); **d** *Trematocaraunimaculatum*, UNIBAS IXF2 (Bathybatini), /1/2/1/2/ (6 total Pt, modal pattern); **e** *Lestradeaperspicax*, UNIBAS IOI1 (Ectodini), /1/1/2/1/ (5 total Pt, modal pattern); **f** *Grammatotrialemairii*, UNIBAS JBC5 (Ectodini), /1/1/1/1/ (4 total Pt, variant pattern). UNIBAS specimen images based on images on MorphoSource copyright by Fabrizia Ronco (see Suppl. material 12); used under CC BY-NC 4.0. Other source images copyright by the respective institutions of the specimens.

In the Pseudocrenilabrinae, the insertion patterns of the last four occupied DIS are more varied than those of the first three DIS, as expected given the additional insertion space; I observed 37 different patterns in this subfamily. The total number of pterygiophores in these four DIS may be 4, 5, 6, 7, 8 or rarely 9 (Fig. 8). Only a single instance of an empty space in this region was seen, /1/–/1/2/ in one of five specimens of *Microdontochromistenuidentatus*.

Of the basal two-supraneural pseudocrenilabrines, *Heterochromis* has at least six different insertion patterns, the modal one (in four of eleven specimens; Fig. 8b) being /2/2/2/2/; *Tylochromispolylepis* has five patterns in six specimens; and *Etia* has /2/2/2/2/ in all six specimens.

In Lake Tanganyika, the most frequent pattern varies by tribe in a few cases (Table 2). In the Cyprichromini and Eretmodini, it is /1/2/1/2/; in the Ectodini, /1/1/2/1/ (Fig. 8e); those two patterns are almost equally common in the Lamprologini examined; in the Perissodini and Tropheina, it is /2/1/2/1/.

The two largest Lake Malawi pseudocrenilabrine clades differ strikingly in the most frequent insertion pattern of the last four DIS, unlike the situation with the first three DIS in these subtribes. In the Pseudotropheina, the commonest pattern is /1/2/2/1/ (in 39.7% of specimens, versus 17.1% of Cyrtocarina specimens); in the Cyrtocarina, the most frequent pattern is /2/1/2/2/ (in 35.5% of specimens, versus — strikingly — only 0.9% of Pseudotropheina specimens). Only one of each clade’s four most frequent patterns is amongst the other clade’s most common four (Table 3). Only 31 specimens of the third Lake Malawi clade, Rhamphochromina, were examined, but in these few, /1/2/2/1/ was the dominant pattern (in 51.6%).

**Last four occupied interhaemal spaces**: In the five specimens of Polycentridae I examined, there are four patterns, only one of which (\3\3\2\3\, in one of two *Monocirrhuspolyacanthus*) did not also occur in cichlids. Another pattern, \2\2\2\2\, found in both specimens of *Polycentropsisabbreviata*, occurs also in all four cichlid subfamilies. The total number of anal pterygiophores in the last four IHS of these five polycentrid specimens ranges from 5 (in *Afronandus*) to 11 (in one of two *Monocirrhus*). No cichlid I have studied has more than 9 pterygiophores in the last four spaces.

The nine specimens of Etroplinae have five patterns, all frequently seen in other subfamilies.

The 27 Ptychochrominae specimens have ten patterns, all occurring also in the Pseudocrenilabrinae and all but one seen in even the few Cichlinae studied.

In the Pseudocrenilabrinae, the pterygiophore insertion patterns of the last four occupied interhaemal spaces (Suppl. material 10) are, again, highly varied, with 39 different patterns found in this study. The total number of pterygiophores in these four IHS may be 4, 5, 6, 7, 8 or rarely 9 (for example, in a *Diplotaxodonargenteus* specimen with the formula \2\2\2\3\; Fig. 7l). Six of the 39 patterns in this subfamily include an empty insertion space which occurs in the first, or rarely the second, of these last four spaces, rarely if ever as the modal pattern of the species. Of the basal taxa with two supraneurals, *Heterochromis* has five patterns in 11 specimens; the modal pattern (in six of eleven) is \1\2\2\2\, which occurs also in the Ptychochrominae and is frequent in many pseudocrenilabrine clades. The six available *Etia* specimens have four different patterns. In *Tylochromispolylepis* the modal pattern (in four of six specimens) is \1\1\2\2\, again frequently seen throughout the Pseudocrenilabrinae. Few trends in insertion patterns in the last four IHS are discernible within this subfamily.

In the Lake Tanganyika fauna, variability and small sample sizes obscure any clear modal pattern in all but two clades. The Lamprologini appear to have a clearly modal pattern of \1\1\2\1\ in 44 of 107 specimens; in the Pseudocrenilabrini, Tropheina, the most frequent pattern is \1\2\1\2\, in 19 of 59 specimens. Several of the insertion patterns observed in the species of Bathybatini were rarely or never found in any other Lake Tanganyika tribe: \2\1\2\2\, \2\2\1\1\, \2\2\1\2\, \2\2\2\1\ and \2\2\2\2\. However, except for \2\2\1\1\, these patterns convergently re-appear in the Lake Malawi subtribe Cyrtocarina (but not at all in the mbuna subtribe Pseudotropheina, except for a single *Pseudotropheuslivingstonii* with \2\1\2\2\).

Within the Lake Malawi pseudocrenilabrines, the most frequently observed pattern in the Pseudotropheina is \1\1\2\2\ (in 33.8% of specimens, versus 8.2% of cyrtocarin specimens); in the Cyrtocarina, the most frequent pattern is \2\1\2\2\ (in 19.1% of specimens, versus only a single pseudotrophein specimen, 0.5%). It is remarkable that none of either clade’s four commonest patterns is amongst the other clade’s most common four (Table 4). In the Rhamphochromina, small sample size prevents making firm conclusions, but \2\2\2\1\ and \2\2\2\2\ occur more frequently than in the other two subtribes.

## Discussion

The results of this study demonstrate that taxonomically and phylogenetically significant differences occur within the African (and other) cichlids in the previously unexplored character complex here surveyed. Counts of precaudal, caudal and total vertebrae, as well as caudal minus precaudal vertebrae, reveal informative distinctions at multiple taxonomic levels. Dorsal and anal pterygiophore counts and their specific patterns of insertion between neural spines and haemal spines, respectively, provide further novel character information.

At the subfamily level, the Etroplinae has, for some time, been resolved through analysis of both morphological and molecular genetic characters, as the sister group of the other three subfamilies. Ptychochrominae is likewise resolved as the sister of the remaining two subfamilies, Cichlinae and Pseudocrenilabrinae (Fig. 3). Most vertebral counts (precaudal, caudal, total and caudal minus precaudal) do not differentiate Etroplinae from Ptychochrominae since the minimum and maximum counts are virtually identical in both. However, the two are completely differentiated by numbers of vertebrae posterior to the last occupied dorsal insertion space and interhaemal space (Suppl. material 3). Furthermore, the total number of anal pterygiophores and their number anterior to the first haemal spine also completely separate these two subfamilies. Moreover, the total number of dorsal pterygiophores, as well as the longest series of one pterygiophore per dorsal insertion space, both overlap by only one between the Etroplinae and Ptychochrominae. I was surprised to find that members of at least two ptychochromine genera commonly have as many as three supraneurals, because only two seem to have been reported previously in that subfamily (see Supraneurals, above).

In contrast to the two more basal cichlid subclades, the Cichlinae and Pseudocrenilabrinae as a whole overlap extensively with each other in ranges of all counts explored in this study. Within pseudocrenilabrines, however, some tribes differ strikingly from others in these characers.

Caudal vertebrae provide one intriguing intertribal comparison. Most African cichlid tribes, including the least-derived ones, have species with a minimum of 12–15 caudal vertebrae (and of course other species with more than the minimum). Nine tribes, though — all in Lake Tanganyika — have an increased minimum caudal vertebrae count. The Lamprologini and Eretmodini have at least 16, the Ectodini, Limnochromini and Cyprichromini at least 17 and the Boulengerochromini, Bathybatini, Benthochromini and Perissodini at least 18 caudal vertebrae (in these nine tribes, the maximum count of caudal vertebrae ranges from only 17 in Eretmodini to as many as 25 in Ectodini).

The maximum pseudocrenilabrine count of total (precaudal plus caudal) vertebrae I observed is 40, seen in three different tribes: Bathybatini, Cyprichromini and Pseudocrenilabrini, Rhamphochromina (see Vertebral counts, above).

The characters surveyed here occasionally prove useful even in distinguishing related genera within a tribe. The most remarkable example is *Cyprichromis*, all species of which have a unique arrangement of 5–7 anal pterygiophores anterior to the first haemal spine, separately inserting towards or between consecutive pairs of pleural ribs (see Anal pterygiophores anterior to first haemal spine, above). Species of *Paracyprichromis*, the sister of *Cyprichromis*, have 0–3 pterygiophores in front of the first haemal spine and these follow the usual, plesiomorphic pseudocrenilabrine pattern of inserting along the anterior edge of that spine.

I found few diagnostic overall differences in supraneurals, pterygiophores or vertebrae between the three Lake Malawi pseudocrenilabrine subtribes Pseudotropheina (the mbuna), Cyrtocarina (the demersal “non-mbuna” or "haps") and Rhamphochromina (the "pelagic subradiation"). The Pseudotropheina and Rhamphochromina do differ in number of vertebrae behind the last occupied DIS (6–9 and 10–13, respectively) and total number of anal pterygiophores (8–10 and 11–14, respectively; Table 1). In addition, the minimum number of anal pterygiophores proved to be only eight in Pseudotropheina, but nine in Cyrtocarina. This corroborates the tentative assignment of *Abactochromis* to the mbuna clade (Oliver and Arnegard 2010), as this enigmatic thick-lipped cichlid usually has only eight anal pterygiophores.

Examining differences between congeneric species was not a focus of this study, but some were noted incidentally. Few distinctions in any pterygiophore counts emerged between congeneric species I examined. One genus, however, *Gobiocichla* (Gobiocichlini), does have a dramatic difference between its two species, with non-overlapping counts of dorsal pterygiophores minus anal pterygiophores (Suppl. material 6). *Gobiocichlaethelwynnae* (11 specimens) has 16–18, mode 17, whereas *G.wonderi* (14 specimens) has 21–23, mode 21. Another intrageneric example, detailed above in Supraneurals, is the variation in supraneural count between species of *Bathybates* with some species consistently having one, others none and still others with one variably present or absent (supraneurals have been lost several times in Pseudocrenilabrinae, e.g. Fig. 4f–i and, conversely, the previously lost second supraneural sporadically re-appears, e.g. Fig. 5e).

More commonly in the African cichlids studied herein, no distinct intrageneric differences occur in these characters. For example, *Cyphotilapia* (Cyphotilapiini) is a genus with two nominal species, *C.frontosa* and *C.gibberosa*, the latter currently a synonym of the former. As radiographs under both names are available on Morphosource, I inspected five of each and found no evident distinction in either numbers of vertebrae and pterygiophores or insertion patterns of the latter structures.

Even though the number of total dorsal or anal pterygiophores is closely related to the externally countable number of spines plus segmented rays in the corresponding dorsal or anal fin, the pterygiophores provide much additional information. Their specific patterns of insertion between the neural and haemal spines are a rich source of novel character data. Especially varied and, thus, informative, are insertion patterns of the last four dorsal and anal pterygiophores. Different species with similar fin-ray counts, particularly from different tribes, may have different numbers of pterygiophores in those spaces. As an example, both *Pelmatolapiamariae* (Pelmatolapiini) and *Lestradeaperspicax* (Ectodini) have about 29 total dorsal-fin spines and rays, but the former has eight or nine pterygiophores (Fig. 8a), the latter only five or six (Fig. 8e), in the last four insertion spaces.

This paper has highlighted only a few of the comparisons possible using the dataset fully recorded in Suppl. material 11. The character complex surveyed in this exploratory study has the potential to contribute to distinguishing species in some tribes, to furnish novel characters for “total evidence” phenomic–genomic phylogenetic analyses and to help determine or corroborate the relationships of fossil cichlids.

## Supplementary Material

XML Treatment for Pseudocrenilabrini

XML Treatment for Pseudocrenilabrina

XML Treatment for Tropheina

XML Treatment for Rhamphochromina

XML Treatment for Cyrtocarina

XML Treatment for Pseudotropheina

AC989727-EF15-53F8-A022-9A1B6FBC4CF9 10.3897/BDJ.12.e130707.suppl1 Supplementary material 1 Introduction to Supplementary Tables Data type text Brief description Explanation of data presentation and sequence of taxa in all supplemental tables. File: oo_1134472.pdf https://binary.pensoft.net/file/1134472 Michael K. Oliver

1B2D5218-E516-5469-AB1F-1C5C0A074290 10.3897/BDJ.12.e130707.suppl2 Supplementary material 2 Table S1. Data type table, morphological, meristic, survey Brief description Frequency distribution of **vertebral counts** in Polycentridae (outgroup) and Cichlidae. File: oo_1134473.pdf https://binary.pensoft.net/file/1134473 Michael K. Oliver

2B89B0D7-532D-5864-8E06-9EE1961FF611 10.3897/BDJ.12.e130707.suppl3 Supplementary material 3 Table S2. Data type table, morphological, meristic, survey Brief description Frequency distribution of **number of vertebrae behind the last occupied dorsal insertion space and last occupied interhaemal space** in Polycentridae (outgroup) and Cichlidae. File: oo_1134474.pdf https://binary.pensoft.net/file/1134474 Michael K. Oliver

0142DD9A-EE55-5C7E-BD29-2F3F91DBE984 10.3897/BDJ.12.e130707.suppl4 Supplementary material 4 Table S3. Data type table, morphological, meristic, survey Brief description Frequency distribution of **total number of dorsal pterygiophores** in Polycentridae (outgroup) and Cichlidae. File: oo_1134476.pdf https://binary.pensoft.net/file/1134476 Michael K. Oliver

43A857ED-B304-5274-9B42-7615049835AC 10.3897/BDJ.12.e130707.suppl5 Supplementary material 5 Table S4. Data type table, morphological, meristic, survey Brief description Frequency distribution of (1) **number of anal pterygiophores anterior to first haemal spine** and (2) **total number of anal pterygiophores** in Polycentridae (outgroup) and Cichlidae. File: oo_1134478.pdf https://binary.pensoft.net/file/1134478 Michael K. Oliver

871064B0-13AB-543E-9832-8B0A53F708C5 10.3897/BDJ.12.e130707.suppl6 Supplementary material 6 Table S5. Data type table, morphological, meristic, survey Brief description Frequency distribution of **number of dorsal pterygiophores minus anal pterygiophores** in Polycentridae (outgroup) and Cichlidae. File: oo_1134480.pdf https://binary.pensoft.net/file/1134480 Michael K. Oliver

7635B465-7F0D-5EF9-9D28-6A6B32EAEFB9 10.3897/BDJ.12.e130707.suppl7 Supplementary material 7 Table S6. Data type table, morphological, meristic, survey Brief description Frequency distribution of **longest consecutive series of a single dorsal pterygiophore in each dorsal insertion space** in Polycentridae (outgroup) and Cichlidae. File: oo_1134481.pdf https://binary.pensoft.net/file/1134481 Michael K. Oliver

8629C422-C9EC-5417-A21E-EA8EBB9C16EE 10.3897/BDJ.12.e130707.suppl8 Supplementary material 8 Table S7. Data type table, morphological, meristic, survey Brief description Frequency distribution of **insertion patterns of supraneural(s) and pterygiophores in the first three dorsal insertion spaces** in Polycentridae (outgroup) and Cichlidae. File: oo_1134483.pdf https://binary.pensoft.net/file/1134483 Michael K. Oliver

39A9D59A-AA54-5982-BEFE-8161A57B1B83 10.3897/BDJ.12.e130707.suppl9 Supplementary material 9 Table S8. Data type table, morphological, meristic, survey Brief description Frequency distribution of **pterygiophore insertion patterns in the last four occupied dorsal insertion spaces** in Polycentridae (outgroup) and Cichlidae. File: oo_1134485.pdf https://binary.pensoft.net/file/1134485 Michael K. Oliver

DA68B1A9-7656-5810-87AD-41B6ACB191B7 10.3897/BDJ.12.e130707.suppl10 Supplementary material 10 Table S9. Data type table, morphological, meristic, survey Brief description Frequency distribution of **anal pterygiophore insertion patterns in the last four occupied interhaemal spaces** in Polycentridae (outgroup) and Cichlidae. File: oo_1134486.pdf https://binary.pensoft.net/file/1134486 Michael K. Oliver

38536764-FA81-556D-A95B-23B5A4603368 10.3897/BDJ.12.e130707.suppl11 Supplementary material 11 Table S10. Data type table, morphological, meristic, survey Brief description RAW SPECIMEN-LEVEL DATA obtained by this study and analysed various ways in the text and in Tables S1–S9. The data represent polycentrid and cichlid vertebral counts and supraneural (Sn) and pterygiophore (Pt) counts and insertion formulae, as modelled after table 10 of Springer & Smith-Vaniz (2008). Two rows represent each specimen, the upper (dorsal fin) having as many columns from left to right as there are total vertebrae, the lower (anal fin) with as many columns as caudal vertebrae and aligned with left end below the cell of the first caudal vertebra. All upper cells, except the last, which is the urostylar vertebra indicated by “c,” represent dorsal insertion spaces, starting with the #1 cell for the first dorsal insertion space to the left (craniad) of the first neural spine. The lower cells represent interhaemal spaces, except for the first (area to left of first haemal spine) and last (urostylar vertebra “c”). Other data are entered as follows: Each 0 indicates a supraneural (SN); other numerals represent the number of pterygiophores (Pt) in an insertion space. Hyphens connect entries with multiple SN or with both SN and Pt within a space; for example, 0-0-2 indicates that a space has two SN and two Pt. An empty insertion space is shown with a dash (–). Uniquely in *Cyprichromis*, numerals and dashes in grey-shaded cells in the anal-fin row indicate abdominal anal pterygiophores anterior to interhaemal space 1, thus inserting towards consecutive precaudal vertebrae with pleural ribs. File: oo_1143598.pdf https://binary.pensoft.net/file/1143598 Michael K. Oliver

34776E42-CFD1-5655-9F40-888A3C39C572 10.3897/BDJ.12.e130707.suppl12 Supplementary material 12 Specimens examined and information sources Data type Text Brief description A detailed listing of all specimens examined for this study, with links to those images that are available online. File: oo_1134489.pdf https://binary.pensoft.net/file/1134489 Michael K. Oliver

## Figures and Tables

**Table 1.** Summary of variation in counts of vertebrae and pterygiophores observed in Cichlidae by subfamily, tribe and subtribe. Data for Polycentridae (outgroup) are also given. Subfamilies and pseudocrenilabrine tribes are arranged in approximate order of estimated branching from basal to terminal (Fig. 3). Values for serranochromines exclude data from specimens of “*Chetia*? sp.”, which were likely misidentified. Pseudocrenilabrini *incertae sedis* includes all riverine and lacustrine genera and species not included in the other groupings within this tribe (see Taxon treatments). Numbers in parentheses indicate counts in atypical (malformed) specimens. See Suppl. materials 2, 3, 4, 5, 6, 7 for complete data on all counts of these structures in all species studied. **Abbreviations**: Ant, anterior; APt, anal pterygiophores; C–PreC, caudal minus precaudal; DIS, dorsal insertion space; DPt, dorsal pterygiophores; HSp1, first haemal spine; IHS, interhemal spaces; L., lake; post, posterior to; PreC, precaudal; Pt, pterygiophore.

FAMILY: Subfamily or Tribe / Subtribe	Vertebrae				Vertebrae post last occupied:			Anal pterygiophores		Total DPt minus total APt	Longest series 1 Pt per DIS
	PreC	Caudal	Total	C–PreC	DIS	IHS	Total DPt	Ant to HSp1	Total		
POLYCENTRIDAE	9–11	13–15	22–26	3–4	3–6	3–8	22–26	1–3	22–26	4–13	4–10
CICHLIDAE: Etroplinae	12–16	14–19	27–34	–1–4	4–5	4–6	25–33	4–8	18–22	5–12	15–18
Ptychochrominae	12–16	14–19	27–33	–2–5	7–12	7–11	21–25	1–2	9–12	10–15	8–15
Cichlinae	12–20	11–19	24–36	–5–5	4–11	3–11	21–32	1–2	8–21	6–21	(6) 12–22
Pseudocrenilabrinae:											
Heterochromini	13	14	27	1	5–6	6–7	27–28	1–2	10–11	17–18	10–13
Tylochromini	14–15	16–17	31	1–3	7–8	9–10	28	1	8–9	19–20	15–17
Hemichromini	12–15	12–17	25–29	–2–5	6–8	6–8	22–25	1–2	8–10	13–16	12–17
Chromidotilapiini	12–15	12–20	25–34	–3–7	4–8	5–10	23–30	0–2	6–12	13–19	(6) 13–25
Etiini	14	12	26	–2	6	6	24	1–2	9	15	14
Oreochromini											
Middle East	14–15	14–15	29–30	–1–1	7–9	7–9	23–25	1	9–10	14–16	14–15
Riverine (African)	13–18	12–16	26–33	–3–1	6–8	6–9	23–30	1–2	7–13	14–18	14–19
L. Barombi	13–15	14–15	27–29	–1–2	6–8	7–8	23–26	1–2	9–11	13–16	14–16
L. Tanganyika	16	15	31	–1	7	7–8	28–29	1–2	10–11	17–19	17
Gobiocichlini	17–18	15–19	32–37	–2–1	5–8	8–10	28–32	0–2	8–13	16–23	18–28
Coelotilapiini	15	13	28	–2	6	6	27	1	9	18	15
Heterotilapiini	15	13	28	–2	5–6	6	28–30	1–2	11–12	16–18	14–15
Coptodonini	14–16	12–15	27–31	–4–1	5–7	5–8	25–28	1–2	8–12	15–18	12–17
Tilapiini	13–15	13–14	26–28	–2–0	5–8	6–7	23–26	1–2	8–11	12–18	13–15
Steatocranini	15–16	14–16	30–31	–2–1	5–6	7–8	27–28	1	7–8	19–21	19
Pelmatolapiini	15	13	28	–2	6	5–6	28	1–2	11–12	16–17	15
Boulengerochromini	16	18–19	34–35	2–3	10	10–11	29–30	1	10–11	19–20	15–16
Bathybatini	11–18	18–22	30–40	3–8	9–13	9–12	20–32	1–2	8–18	9–14	12–17
Lamprologini	11–17	16–22	27–37	0–10	5–11	6–12	23–29	1–4	9–16	11–19	17–23
Cyphotilapiini	15–17	15–16	32	–2–0	7–8	8–9	26–27	1–2	8–9	17–19	16–20
Ectodini	13–17	17–25	32–39	1–11	7–11	9–14	24–31	1–3	8–12	12–19	(12) 19–26
Limnochromini	13–16	17–19	30–35	2–5	8–10	9–11	22–28	1–2	8–10	14–18	(13) 16–22
Cyprichromini	16–21	17–20	35–40	–3–4	10–12	11–13	26–30	0–7	8–14	14–19	18–23
Benthochromini	17–18	18–19	35–37	0–2	9–10	9–10	27–29	1	10–11	16–18	19–23
Perissodini	15–18	18–21	34–39	2–4	8–11	9–12	27–32	1–2	10–13	16–20	16–24
Eretmodini	13–14	16–17	29–30	2–4	4–5	8–9	26–27	1–2	8–9	18–19	19–21
Pseudocrenilabrini:											
Serranochromines	15–17	15–19	31–36	–1–3	7–10	8–10	25–29	1–3	9–12	15–19	17–19
Pseudocrenilabrina	12–14	14–18	26–31	1–5	5–8	6–10	23–26	1–2	8–10	14–17	15–18
Tropheina	13–16	15–18	29–34	–1–4	5–9	6–10	23–28	1–2	8–11	15–19	15–19
Cyrtocarina	12–16	15–20	29–35	0–7	6–11	7–12	23–31	0–2	9–13	13–18	12–20
Pseudotropheina	13–16	15–17	28–33	–1–4	6–9	7–10	22–27	1–3	8–10	14–19	(13–14) 16–21
Rhamphochromina	14–18	17–22	33–40	–1–5	10–13	10–13	24–31	0–4	11–14	11–19	14–21
*incertae sedis*	12–15	14–19	26–32	–1–7	5–11	7–12	21–27	1–3	8–11	12–19	(6–10) 14–20

**Table 2.** Summary of most-frequent insertion patterns of supraneurals and pterygiophores in first three dorsal insertion spaces (DIS) and of dorsal and anal pterygiophores in last four occupied DIS and interhaemal spaces, respectively, for Cichlidae by subfamily, tribe and subtribe. Data from Polycentridae (outgroup) are also given. Subfamilies and pseudocrenilabrine tribes are arranged in approximate order of estimated branching from basal to terminal (Fig. 3). Values for serranochromines exclude data from specimens of “*Chetia* ? sp.”, which were likely misidentified. Pseudocrenilabrini *incertae sedis* includes all riverine and lacustrine genera and species not included in the other groupings within this tribe (see Taxon treatments). See Suppl. materials 8, 9, 10 for complete data on all insertion patterns of all species studied. **Abbreviations**: DIS, dorsal insertion spaces; IHS, interhaemal spaces.

FAMILY: Subfamily or Tribe/Subtribe	Most-frequent insertion patterns		
	First 3 DIS	Last 4 occupied DIS	Last 4 occupied IHS
POLYCENTRIDAE	0/0/0-1/ (in 2 of 5)	2/2/2/3/ (in 2 of 5)	none > once
CICHLIDAE: Etroplinae	0/0-1/1/	/2/2/2/1/; /2/1/2/2/; /2/2/1/2/	\2\2\2\1\; \2\2\2\2\
Ptychochrominae	0/0/2/; 0-0/0-1/2/; 0-0/0-2/1/	/1/2/2/2/; /2/1/2/2/	\2\1\2\1\; \1\2\2\2\; \2\1\2\2\
Cichlinae	0/0-2/1/	/1/2/2/2/; /1/2/2/1/; /2/1/2/1/	\2\2\2\1\
Pseudocrenilabrinae:			
Heterochromini	0/0-2/1/	/1/2/2/2/	\1\2\2\2\
Tylochromini	0/0-2/1/	/1/2/2/3/	\1\1\2\2\
Hemichromini	0/2/1/	/2/2/2/1/	\1\2\2\1\
Chromidotilapiini	0/2/1/	/2/2/2/1/; /2/1/2/2/; /1/2/2/2/	\1\2\2\1\; \1\1\2\2\; \2\1\2\1\
Etiini	0-0/2/1/	/2/2/2/2/	\1\2\3\1\; \2\2\2\1\; \2\2\3\1\
Oreochromini			
Middle East	0/2/1/	/2/1/2/1/; /2/1/2/2/	\1\2\2\1\
Riverine (African)	0/2/1/	/1/2/2/2/	\1\2\2\2\
Lake Barombi	0/2/1/	/2/2/2/1/	\1\2\2\2\
Lake Tanganyika	0/2/1/	/2/2/2/1/	\1\2\2\2\
Gobiocichlini	–/2/1/; –/–/2/	/1/2/1/1/; /1/2/1/2/	\1\2\1\2\; \1\2\1\1\
Coelotilapiini	0/2/1/	/1/2/2/3/	\1\2\2\2\
Heterotilapiini	0/2/1/	/2/2/2/3/	\2\2\2\3\
Coptodonini	0/2/1/	/1/2/2/2/; /1/2/3/1/	\1\2\2\1\; \2\2\2\1\; \2\2\2\2\
Tilapiini	0/2/1/	/2/2/2/2/; /2/2/2/1/; /1/2/2/1/	\1\2\2\1\; \1\2\2\2\
Steatocranini	0/2/1/	/2/1/2/1/	\1\1\2\1\
Pelmatolapiini	0/2/1/	/2/2/2/3/	\2\2\2\2\
Boulengerochromini	0/2/1/	/2/2/2/2/; /2/2/2/1/	\1\2\2\2\
Bathybatini	0/1/2/	/2/1/2/2/; /1/2/1/1/; /1/2/2/1/	\2\1\2\2\; \2\2\1\2\; \2\2\2\1\
Lamprologini	0/2/1/	/1/1/2/1/; /1/2/1/2/; /1/1/2/2/	\1\1\2\1\; \1\2\1\1\
Cyphotilapiini	0/2/1/	/1/2/2/1/; /2/1/2/1/	\1\1\2\2\; \1\2\2\1\
Ectodini	0/2/1/	/1/1/2/1/; 1/1/1/2/	\1\1\1\2\; \1\1\2\1\
Limnochromini	0/2/1/	/1/1/2/1/; /1/2/1/2/	\1\2\1\2\; \1\1\2\1\; \1\1\2\2\
Cyprichromini	0/1/2/; 0/2/1/	/1/2/1/2/; /1/2/1/1/; /2/1/1/1/	\1\2\1\2\; \1\1\2\1\; \2\1\2\1\
Benthochromini	0/2/1/	/1/1/2/1/	none > once
Perissodini	0/2/1/	/2/1/2/1/	\2\1\2\2\; \1\1\2\2\; \2\1\2\1\
Eretmodini	0/2/1/	/1/2/1/2/	\1\1\2\1\
Pseudocrenilabrini			
Serranochromines	0/2/1/	/2/1/2/2/; /2/1/2/1/; /2/2/2/1/	\1\2\2\1\; \2\2\1\1\; \1\2\2\2\
Pseudocrenilabrina	0/2/1/	/1/2/2/1/	\2\1\2\1\; \1\1\2\2\; \1\2\2\1\
Tropheina	0/2/1/	/2/1/2/1/; /1/2/1/2/	\1\2\1\2\; \1\1\2\1\; \1\1\2\2\
Cyrtocarina	0/2/1/; 0/1/2/	/2/1/2/2/; /1/2/2/1/	\2\1\2\2\; \1\2\2\2\; \1\2\2\1\
Pseudotropheina	0/2/1/	/1/2/2/1/; /1/2/1/2/	\1\1\2\2\; \1\2\1\2\; \1\1\2\1\
Rhamphochromina	0/1/2; 0/–/3	/1/2/2/1/	no clear modal pattern
*incertae sedis*	0/2/1/	/1/2/1/2/; /1/2/2/1/; /2/1/2/1/	\1\2\1\2\; \2\1\2\2\; \1\2\2\2\

**Table 3.** Dorsal pterygiophores in the last four occupied dorsal insertion spaces: Comparison of the three Lake Malawi pseudocrenilabrine subtribes. For each subtribe, the four most frequent patterns are in **boldface** (number of specimens and percentage). For the Pseudotropheina and the Cyrtocarina, note that only one of the four most frequent patterns of either subtribe is amongst the other’s most common four. Furthermore, the most frequent cyrtocarin pattern is found in only one percent of pseudotropheins. Abbreviation: DIS, dorsal insertion spaces. All specimens and their insertion patterns, including less frequent ones, are shown in Suppl. material 9.

Last 4 occupied DIS	/1/1/2/2/	/1/2/1/2/	/1/2/2/1/	/1/2/2/2/	/2/1/2/1/	/2/1/2/2/	/2/1/2/3/
Pseudotropheina (n = 219)	**26**	**35**	**87**	7	**23**	2	0
	**11.9**%	**16.0**%	**39.7**%	3.2%	**10.5**%	0.9%	0.0%
Cyrtocarina (n = 425)	12	12	**78**	**49**	17	**162**	**34**
	2.6%	2.6%	**17.1**%	**10.7**%	3.7%	**35.5**%	**7.5**%
Rhamphochromina (n = 31)	**3**	**4**	**16**	1	0	**5**	2
	**9.7**%	**12.9**%	**51.6**%	3.2%	0.0%	**16.1**%	6.5%

**Table 4.** Anal pterygiophores in the last four occupied interhaemal spaces: comparison of the three Lake Malawi pseudocrenilabrine subtribes. For each subtribe, the four most frequent patterns are in **boldface** (number of specimens and percentage). Due to small sample sizes with tied counts, Rhamphochromina has five most frequent patterns. Note that none of the commonest patterns is the same in the large subtribes Pseudotropheina and Cyrtocarina. Abbreviation: IHS, interhaemal spaces. All specimens and their insertion patterns, including less frequent ones, are shown in Suppl. material 10.

Last 4 occupied IHS	\1\1\2\1\	\1\1\2\2\	\1\2\1\2\	\1\2\1\3\	\1\2\2\1\	\1\2\2\2\	\2\1\2\1\	\2\1\2\2\	\2\2\2\1\	\2\2\2\2\
Pseudotropheina (n = 219)	**26**	**74**	**38**	2	14	0	**20**	1	0	0
	**11.9**%	**33.8**%	**17.4**%	0.9%	6.4%	0.0%	**9.1**%	0.5%	0.0%	0.0%
Cyrtocarina (n = 425)	2	35	19	**52**	**69**	**70**	21	**81**	18	1
	0.5%	8.2%	4.5%	**12.2**%	**16.2**%	**16.5**%	4.6%	**19.1**%	4.2%	0.2%
Rhamphochromina (n = 31)	0	0	1	0	**4**	**4**	0	**5**	**4**	**4**
	0.0%	0.0%	3.2%	0.0%	**12.9**%	**12.9**%	0.0%	**16.1**%	**12.9**%	**12.9**%
