# Supplementary material for: African cichlid fishes: morphological data and taxonomic insights from a genus-level survey of supraneurals, pterygiophores, and vertebral counts (Ovalentaria, Blenniiformes, Cichlidae, Pseudocrenilabrinae)
Source: Biodivers Data J. 2024 Oct 18;12:e130707. doi: 10.3897/BDJ.12.e130707 (PMC11512106; doi:10.3897/BDJ.12.e130707)
Supplement: Supplementary material 1 — Introduction to Supplementary Tables [file bdj-12-e130707-s001.pdf]

Data presentation and Sequence of taxa in all supplementary tables:

After the few Polycentridae, the examined specimens of Cichlidae are listed by subfamily: Etroplinae, Ptychochrominae, Cichlinae (alphabetically by tribe), and Pseudocrenilabrinae. Within Pseudocrenilabrinae, yellow cells in the first column label geographical groups of taxa: first those of the Middle East, followed by African Riverine, and then lakes: Barombi Mbo, Fwa, Albert, the Edward–George system, Victoria and its satellite lakes, Kivu, Turkana, Tanganyika, and Malawi. Under each geographical heading, the taxa are listed alphabetically by tribe, then by genus and species. Note that, in agreement with current molecular phylogenetic hypotheses, “pelmatochromines” are included in Chromidotilapiini; *Trematocara* is included in Bathybatini; and the traditional Lake Tanganyika tribe Tropheini is a subtribe of Haplochromini. The species binomens follow Fricke et al. (2024).

For tribes with three or more species studied, the number of specimens with each count is totaled in a pink row. For Lake Malawi, the entire column totals for each of the three endemic subtribes, Cyrtocarina, Pseudotropheina, and Rhamphochromina, are repeated at the foot of the table for ease of comparison. Counts with an asterisk are from the primary type specimen (holotype or lectotype) of that species or of a species currently synonymized with it (see Appendix 2).
