# Supplementary material for: African cichlid fishes: morphological data and taxonomic insights from a genus-level survey of supraneurals, pterygiophores, and vertebral counts (Ovalentaria, Blenniiformes, Cichlidae, Pseudocrenilabrinae)
Source: Biodivers Data J. 2024 Oct 18;12:e130707. doi: 10.3897/BDJ.12.e130707 (PMC11512106; doi:10.3897/BDJ.12.e130707)
Supplement: Supplementary material 2 — Table S1. [file bdj-12-e130707-s002.pdf]

Table 1. Frequency distribution of vertebral counts (precaudal, caudal, total, and caudal minus precaudal)

Precaudal vertebrae

	9	10	11	12	13	14	15	16	17	18	19	20	21
**Polycentridae**													
Afronandus sheljuzhkoi			1										
Monocirrhus polyacanthus	1	1											
Polycentropsis abbreviata	1	1											
**Cichlidae:** Etroplinae													
India													
Etroplus suratensis								1					
Pseudetroplus maculatus				3	2	2							
Madagascar													
Paretroplus polyactis							1						
Ptychochrominae													
Madagascar													
Katria katria							1						
Oxylapia polli						1							
Paratilapia polleni				10	1								
Ptychochromis oligacanthus					1	10							
Ptychochromoides betsileanus							2	1					
Cichlinae													
Neotropics													
**Astronotini**													
Astronotus ocellatus							3						
**Chaetobranchini**													
Chaetobranchopsis orbicularis					1								
Chaetobranchus flavescens					4								
**Cichlasomatini**													
Aequidens tetramerus					1								
Andinoacara pulcher				1									
Bujurquina vittata					1								
Cichlasoma dimerus					1								
Cichlasoma taenia					1								
Krobia guianensis				1									
**Cichlini**													
Cichla ocellaris										1			
Cichla orinocensis										2	1		
**Geophagini**													
Biotodoma cupido						4							
Geophagus crassilabris						1							
Guianacara owroewefi					8								
Saxatilia frenata												1*	
Saxatilia lepidota										2			
**Heroini**													
Amphilophus citrinellus						1							
Hypselacara coryphaenoides						5							
**Retroculini**													
Retroculus lapidifer						1							
Pseudocrenilabrinae													
Middle East													
**Oreochromini**													
Iranocichla hormuzensis							2						
Tristramella sacra							1						
Tristramella simonis						2	2						
**Pseudocrenilabrini**													
Astatotilapia flaviijosephi				1	3								
**Oreochromini (M.E.) column totals**	–	–	–	–	–	2	5	–	–	–	–	–	–

Caudal vertebrae

	11	12	13	14	15	16	17	18	19	20	21	22	23	24	25
**Polycentridae**															
Afronandus sheljuzhkoi					1										
Monocirrhus polyacanthus			2												
Polycentropsis abbreviata			2												
**Cichlidae:** Etroplinae															
India															
Etroplus suratensis					1										
Pseudetroplus maculatus				3	4										
Madagascar															
Paretroplus polyactis									1						
Ptychochrominae															
Madagascar															
Katria katria						1									
Oxylapia polli									1						
Paratilapia polleni					10	1									
Ptychochromis oligacanthus				10	1										
Ptychochromoides betsileanus				1	1	1									
Cichlinae															
Neotropics															
**Astronotini**															
Astronotus ocellatus							3								
**Chaetobranchini**															
Chaetobranchopsis orbicularis				1											
Chaetobranchus flavescens			4												
**Cichlasomatini**															
Aequidens tetramerus			1												
Andinoacara pulcher			1												
Bujurquina vittata	1														
Cichlasoma dimerus		1													
Cichlasoma taenia			1												
Krobia guianensis			1												
**Cichlini**															
Cichla ocellaris								1							
Cichla orinocensis						1	–	2							
**Geophagini**															
Biotodoma cupido				4											
Geophagus crassilabris					1										
Guianacara owroewefi				8											
Saxatilia frenata					1*										
Saxatilia lepidota					2										
**Heroini**															
Amphilophus citrinellus							1								
Hypselacara coryphaenoides					5										
**Retroculini**															
Retroculus lapidifer									1						
Pseudocrenilabrinae															
Middle East															
**Oreochromini**															
Iranocichla hormuzensis				2											
Tristramella sacra					1										
Tristramella simonis				2	2										
**Pseudocrenilabrini**															
Astatotilapia flaviijosephi				3	1										
**Oreochromini (M.E.) column totals**	–	–	–	4	3	–	–	–	–	–	–	–	–	–	–

Total vertebrae

	22	23	24	25	26	27	28	29	30	31	32	33	34	35	36	37	38	39	40
**Polycentridae**																			
Afronandus sheljuzhkoi					1														
Monocirrhus polyacanthus	1	1																	
Polycentropsis abbreviata	1	1																	
**Cichlidae:** Etroplinae																			
India																			
Etroplus suratensis										1									
Pseudetroplus maculatus						5	1	1											
Madagascar																			
Paretroplus polyactis													1						
Ptychochrominae																			
Madagascar																			
Katria katria										1									
Oxylapia polli												1							
Paratilapia polleni						9	2												
Ptychochromis oligacanthus							11												
Ptychochromoides betsileanus									2	1									
Cichlinae																			
Neotropics																			
**Astronotini**																			
Astronotus ocellatus											3								
**Chaetobranchini**																			
Chaetobranchopsis orbicularis						1													
Chaetobranchus flavescens					4														
**Cichlasomatini**																			
Aequidens tetramerus					1														
Andinoacara pulcher				1															
Bujurquina vittata			1																
Cichlasoma dimerus				1															
Cichlasoma taenia					1														
Krobia guianensis				1															
**Cichlini**																			
Cichla ocellaris															1				
Cichla orinocensis														1	2				
**Geophagini**																			
Biotodoma cupido							4												
Geophagus crassilabris								1											
Guianacara owroewefi						8													
Saxatilia frenata														1*					
Saxatilia lepidota												2							
**Heroini**																			
Amphilophus citrinellus										1									
Hypselacara coryphaenoides								5											
**Retroculini**																			
Retroculus lapidifer												1							
Pseudocrenilabrinae																			
Middle East																			
**Oreochromini**																			
Iranocichla hormuzensis								2											
Tristramella sacra									1										
Tristramella simonis								4											
**Pseudocrenilabrini**																			
Astatotilapia flaviijosephi						4													
**Oreochromini (M.E.) column totals**	–	–	–	–	–	–	–	6	1	–	–	–	–	–	–	–	–	–	–

Caudal minus precaudal vertebrae

	−5	−4	−3	−2	−1	0	1	2	3	4	5	6	7	8	9	10	11
**Polycentridae**																	
Afronandus sheljuzhkoi										1							
Monocirrhus polyacanthus									1	1							
Polycentropsis abbreviata									1	1							
**Cichlidae:** Etroplinae																	
India																	
Etroplus suratensis					1												
Pseudetroplus maculatus						1	3	–	3								
Madagascar																	
Paretroplus polyactis										1							
Ptychochrominae																	
Madagascar																	
Katria katria							1										
Oxylapia polli											1						
Paratilapia polleni								1	9	1							
Ptychochromis oligacanthus						10	–	1									
Ptychochromoides betsileanus				1	–	1	1										
Cichlinae																	
Neotropics																	
**Astronotini**																	
Astronotus ocellatus								3									
**Chaetobranchini**																	
Chaetobranchopsis orbicularis							1										
Chaetobranchus flavescens						4											
**Cichlasomatini**																	
Aequidens tetramerus						1											
Andinoacara pulcher							1										
Bujurquina vittata				1													
Cichlasoma dimerus					1												
Cichlasoma taenia						1											
Krobia guianensis							1										
**Cichlini**																	
Cichla ocellaris						1											
Cichla orinocensis			1	–	–	2											
**Geophagini**																	
Biotodoma cupido						4											
Geophagus crassilabris							1										
Guianacara owroewefi							8										
Saxatilia frenata	1*																
Saxatilia lepidota			2														
**Heroini**																	
Amphilophus citrinellus									1								
Hypselacara coryphaenoides							5										
**Retroculini**																	
Retroculus lapidifer											1						
Pseudocrenilabrinae																	
Middle East																	
**Oreochromini**																	
Iranocichla hormuzensis					2												
Tristramella sacra						1											
Tristramella simonis					2	–	2										
**Pseudocrenilabrini**																	
Astatotilapia flaviijosephi							3	–	1								
**Oreochromini (M.E.) column totals**	–	–	–	–	4	1	2	–	–	–	–	–	–	–	–	–	–

Table 1 (continued). Frequency distribution of vertebral counts (precaudal, caudal, total, and caudal minus precaudal)

	Precaudal vertebrae												
	9	10	11	12	13	14	15	16	17	18	19	20	21
African riverine													
**Chromidotilapiini**													
Benitochromis batesii					6								
Benitochromis finleyi						1							
Chromidotilapia guntheri						2							
Chromidotilapia kingsleyae					4	2							
Congochromis robustus						1*							
Congochromis squamiceps						2							
Divandu albimarginatus				1	3								
Limbochromis robertsi					17								
Nanochromis nudiceps						2							
Nanochromis parilus						2							
Parananochromis gabonicus					1*								
Parananochromis longirostris					3	8							
Pelmatochromis buettikoferi							3						
Pelmatochromis ocellifer						1*							
Pelvicachromis pulcher						1							
Pelvicachromis taeniatus						1*							
Pterochromis congicus					2*								
Teleogramma brichardi					4								
Thysochromis ansorgii					3	1							
Wallaceochromis humilis							5*						
**Chromidotilapiini column totals**	–	–	–	1	43	24	8	–	–	–	–	–	–
**Coelotilapiini**													
Coelotilapia joka							1						
**Coptodonini**													
Coptodon discolor							2						
Coptodon tholloni						1							
Coptodon zillii						6	5	13					
**Coptodonini column totals**	–	–	–	–	–	7	7	13	–	–	–	–	–
**Etiini**													
Etia nguti						6							
**Gobiocichlini**													
Gobiocichla ethelwynnae									3	8			
Gobiocichla wonderi										14			
Paragobiocichla irvinei									4				
**Gobiocichlini column totals**	–	–	–	–	–	–	–	–	7	22	–	–	–
**Hemichromini**													
Anomalochromis thomasi				2	2								
Hemichromis elongatus							4						
Hemichromis fasciatus							1						
Rubricatochromis bimaculatus					5	1							
Rubricatochromis letourneuxi				1	3								
**Hemichromini column totals**	–	–	–	3	10	1	5	–	–	–	–	–	–
**Heterochromini**													
Heterochromis multidens					11								
**Heterotilapiini**													
Heterotilapia buettikoferi							5						
**Oreochromini**													
Danakilia franchettii					3	8	3						
Oreochromis jipe									3	1			
Oreochromis niloticus						1	–	2					
Oreochromis shiranus							5	1					
Sarotherodon galilaeus								2					
Sarotherodon melanotheron						4	2						
**Oreochromini (riverine) totals**	–	–	–	–	3	13	10	5	3	1	–	–	–
**Pelmatolapiini**													
Pelmatolapia mariae							6						

	Caudal vertebrae														
	11	12	13	14	15	16	17	18	19	20	21	22	23	24	25
African riverine															
**Chromidotilapiini**															
Benitochromis batesii			6												
Benitochromis finleyi			1												
Chromidotilapia guntheri			2												
Chromidotilapia kingsleyae		1	5												
Congochromis robustus			1*												
Congochromis squamiceps		1	–	1											
Divandu albimarginatus			4												
Limbochromis robertsi				17											
Nanochromis nudiceps			1	–	1										
Nanochromis parilus					2										
Parananochromis gabonicus				1*											
Parananochromis longirostris		7	4												
Pelmatochromis buettikoferi		3													
Pelmatochromis ocellifer		1*													
Pelvicachromis pulcher			1												
Pelvicachromis taeniatus			1*												
Pterochromis congicus			2*												
Teleogramma brichardi										4					
Thysochromis ansorgii			2	2											
Wallaceochromis humilis			1*	4											
**Chromidotilapiini column totals**	–	13	31	25	3	–	–	–	–	4	–	–	–	–	–
**Coelotilapiini**															
Coelotilapia joka			1												
**Coptodonini**															
Coptodon discolor			2												
Coptodon tholloni			1												
Coptodon zillii		1	13	7	3										
**Coptodonini column totals**	–	1	16	7	3	–	–	–	–	–	–	–	–	–	–
**Etiini**															
Etia nguti		6													
**Gobiocichlini**															
Gobiocichla ethelwynnae							8	3							
Gobiocichla wonderi								13	1						
Paragobiocichla irvinei					4										
**Gobiocichlini column totals**	–	–	–	–	4	–	8	16	1	–	–	–	–	–	–
**Hemichromini**															
Anomalochromis thomasi		2	2												
Hemichromis elongatus			4												
Hemichromis fasciatus			1												
Rubricatochromis bimaculatus			5	1											
Rubricatochromis letourneuxi			3	–	–	–	1								
**Hemichromini column totals**	–	2	15	1	–	–	1	–	–	–	–	–	–	–	–
**Heterochromini**															
Heterochromis multidens				11											
**Heterotilapiini**															
Heterotilapia buettikoferi			5												
**Oreochromini**															
Danakilia franchettii		2	9	3											
Oreochromis jipe					3	1									
Oreochromis niloticus			1	1	1										
Oreochromis shiranus				1	5										
Sarotherodon galilaeus			2												
Sarotherodon melanotheron		2	4												
**Oreochromini (riverine) totals**	–	4	16	5	9	1	–	–	–	–	–	–	–	–	–
**Pelmatolapiini**															
Pelmatolapia mariae			6												

	Total vertebrae																		
	22	23	24	25	26	27	28	29	30	31	32	33	34	35	36	37	38	39	40
African riverine																			
**Chromidotilapiini**																			
Benitochromis batesii					6														
Benitochromis finleyi						1													
Chromidotilapia guntheri						2													
Chromidotilapia kingsleyae					5	1													
Congochromis robustus						1*													
Congochromis squamiceps					1	–	1												
Divandu albimarginatus				1	3														
Limbochromis robertsi						17													
Nanochromis nudiceps							1	1											
Nanochromis parilus								2											
Parananochromis gabonicus						1*													
Parananochromis longirostris					10	1													
Pelmatochromis buettikoferi						3													
Pelmatochromis ocellifer					1*														
Pelvicachromis pulcher						1													
Pelvicachromis taeniatus						1*													
Pterochromis congicus					2*														
Teleogramma brichardi													4						
Thysochromis ansorgii					1	3													
Wallaceochromis humilis							1*	4											
**Chromidotilapiini column totals**	–	–	–	1	29	32	3	7	–	–	–	–	4	–	–	–	–	–	–
**Coelotilapiini**																			
Coelotilapia joka							1												
**Coptodonini**																			
Coptodon discolor							2												
Coptodon tholloni						1													
Coptodon zillii							9	13	–	2									
**Coptodonini column totals**	–	–	–	–	–	1	11	13	–	2	–	–	–	–	–	–	–	–	–
**Etiini**																			
Etia nguti					6														
**Gobiocichlini**																			
Gobiocichla ethelwynnae													1	9	1				
Gobiocichla wonderi															13	1			
Paragobiocichla irvinei											4								
**Gobiocichlini column totals**	–	–	–	–	–	–	–	–	–	–	4	–	1	9	14	1	–	–	–
**Hemichromini**																			
Anomalochromis thomasi				4															
Hemichromis elongatus							4												
Hemichromis fasciatus							1												
Rubricatochromis bimaculatus					4	2													
Rubricatochromis letourneuxi					3	–	–	1											
**Hemichromini column totals**	–	–	–	4	7	2	5	1	–	–	–	–	–	–	–	–	–	–	–
**Heterochromini**																			
Heterochromis multidens						11													
**Heterotilapiini**																			
Heterotilapia buettikoferi							5												
**Oreochromini**																			
Danakilia franchettii					1	11	2												
Oreochromis jipe											2	2							
Oreochromis niloticus						1	–	–	1	1									
Oreochromis shiranus									6										
Sarotherodon galilaeus								2											
Sarotherodon melanotheron						6													
**Oreochromini (riverine) totals**	–	–	–	–	1	18	2	2	7	1	2	2	–	–	–	–	–	–	–
**Pelmatolapiini**																			
Pelmatolapia mariae							6												

	Caudal minus precaudal vertebrae																
	−5	−4	−3	−2	−1	0	1	2	3	4	5	6	7	8	9	10	11
African riverine																	
**Chromidotilapiini**																	
Benitochromis batesii						6											
Benitochromis finleyi					1												
Chromidotilapia guntheri					2												
Chromidotilapia kingsleyae				1	1	4											
Congochromis robustus					1*												
Congochromis squamiceps				1	–	1											
Divandu albimarginatus						3	1										
Limbochromis robertsi							17										
Nanochromis nudiceps					1	–	1										
Nanochromis parilus							2										
Parananochromis gabonicus							1*										
Parananochromis longirostris				7	1	3											
Pelmatochromis buettikoferi			3														
Pelmatochromis ocellifer				1*													
Pelvicachromis pulcher					1												
Pelvicachromis taeniatus					1*												
Pterochromis congicus						2*											
Teleogramma brichardi													4				
Thysochromis ansorgii					1	1	2										
Wallaceochromis humilis				1*	4												
**Chromidotilapiini column totals**	–	–	3	11	14	20	24	–	–	–	–	–	4	–	–	–	–
**Coelotilapiini**																	
Coelotilapia joka				1													
**Coptodonini**																	
Coptodon discolor				2													
Coptodon tholloni					1												
Coptodon zillii		1	10	3	4	5	1										
**Coptodonini column totals**	–	1	10	5	5	5	1	–	–	–	–	–	–	–	–	–	–
**Etiini**																	
Etia nguti				6													
**Gobiocichlini**																	
Gobiocichla ethelwynnae					7	2	2										
Gobiocichla wonderi						13	1										
Paragobiocichla irvinei				4													
**Gobiocichlini column totals**	–	–	–	4	7	15	3	–	–	–	–	–	–	–	–	–	–
**Hemichromini**																	
Anomalochromis thomasi					2	–	2										
Hemichromis elongatus				4													
Hemichromis fasciatus				1													
Rubricatochromis bimaculatus					1	4	1										
Rubricatochromis letourneuxi						3	–	–	–	–	1						
**Hemichromini column totals**	–	–	–	5	3	7	3	–	–	–	1	–	–	–	–	–	–
**Heterochromini**																	
Heterochromis multidens							11										
**Heterotilapiini**																	
Heterotilapia buettikoferi				5													
**Oreochromini**																	
Danakilia franchettii			2	1	7	2	2										
Oreochromis jipe			1	2	1												
Oreochromis niloticus				1	2												
Oreochromis shiranus				1	–	5											
Sarotherodon galilaeus			2														
Sarotherodon melanotheron			2	–	4												
**Oreochromini (riverine) totals**	–	–	7	5	14	7	2	–	–	–	–	–	–	–	–	–	–
**Pelmatolapiini**																	
Pelmatolapia mariae				6													

Table 1 (continued). Frequency distribution of vertebral counts (precaudal, caudal, total, and caudal minus precaudal)

	Precaudal vertebrae												
	9	10	11	12	13	14	15	16	17	18	19	20	21
**Pseudocrenilabrini**													
Astatotilapia bloyeti				3	5								
Astatotilapia burtoni					6								
Astatotilapia calliptera					7*	2							
Astatotilapia swynnertoni					3								
Chetia flaviventris						1							
Chetia gracilis							1*						
Chetia ? sp.				5	3								
Ctenochromis pectoralis				2									
Ctenochromis scatebra					1*								
Haplochromis demeusii					1*								
Haplochromis fasciatus						6							
Haplochromis humilis						3							
Haplochromis moeruensis					2	2							
Haplochromis oligacanthus				1*									
Lufubuchromis relictus					1*								
Orthochromis machadoi					2								
Orthochromis malagaraziensis					2								
Orthochromis polyacanthus						2							
Orthochromis stormsi					9	2							
Palaeoplex palimpsest						1*							
Pharyngochromis acuticeps					2	4							
Pseudocrenilabrus multicolor				1	3								
Pseudocrenilabrus philander				1									
Sargochromis carlottae							1	1					
Sargochromis codringtonii							1	2*					
Sargochromis giardi							2						
Sargochromis greenwoodi								2*					
Serranochromis angusticeps								1					
Serranochromis longimanus								4*					
Serranochromis macrocephalus							1						
Serranochromis meridianus							1						
Serranochromis robustus								4	2				
Thoracochromis albolabris					1								
Thoracochromis wingatii				1	1								
**Pseudocrenilabrini (riverine) totals**	–	–	–	14	49	23	7	14	2	–	–	–	–
**Steatocranini**													
Steatocranus casuarius							2	1					
**Tilapiini**													
Chilochromis duponti							1						
Congolapia bilineata						2	1						
Tilapia busumana						4							
Tilapia sparrmanii					1*	3							
**Tilapiini column totals**	–	–	–	–	1	9	2	–	–	–	–	–	–
**Lake Barombi Mbo**													
**Oreochromini**													
Konia eisentrauti						5							
Myaka myaka						4							
Pungu maclareni							2*						
Stomatepia mariae					1								
Stomatepia pindu					2	2							
**Oreochromini (Barombi) totals**	–	–	–	–	3	11	2	–	–	–	–	–	–
**Lake Fwa**													
**Pseudocrenilabrini**													
Cyclopharynx schwetzi						12	2						
Schwetzochromis neodon						2							

	Caudal vertebrae														
	11	12	13	14	15	16	17	18	19	20	21	22	23	24	25
**Pseudocrenilabrini**															
Astatotilapia bloyeti						5	3								
Astatotilapia burtoni					5	1									
Astatotilapia calliptera					1	4	4*								
Astatotilapia swynnertoni					1	2									
Chetia flaviventris							1								
Chetia gracilis							1*								
Chetia ? sp.			3	5											
Ctenochromis pectoralis							2								
Ctenochromis scatebra							1*								
Haplochromis demeusii				1*											
Haplochromis fasciatus				6											
Haplochromis humilis						3									
Haplochromis moeruensis						2	2								
Haplochromis oligacanthus				1*											
Lufubuchromis relictus						1*									
Orthochromis machadoi							1	1							
Orthochromis malagaraziensis								2							
Orthochromis polyacanthus							1	1							
Orthochromis stormsi					1	8	2								
Palaeoplex palimpsest						1*									
Pharyngochromis acuticeps						5	1								
Pseudocrenilabrus multicolor				4											
Pseudocrenilabrus philander						1									
Sargochromis carlottae					1	1									
Sargochromis codringtonii						2	1*								
Sargochromis giardi						1	1								
Sargochromis greenwoodi						2*									
Serranochromis angusticeps									1						
Serranochromis longimanus							4*								
Serranochromis macrocephalus								1							
Serranochromis meridianus								1							
Serranochromis robustus								3	3						
Thoracochromis albolabris									1						
Thoracochromis wingatii						2									
**Pseudocrenilabrini (riverine) totals**	–	–	3	17	9	41	25	9	5	–	–	–	–	–	–
**Steatocranini**															
Steatocranus casuarius				1	–	2									
**Tilapiini**															
Chilochromis duponti			1												
Congolapia bilineata			1	2											
Tilapia busumana			4												
Tilapia sparrmanii			4*												
**Tilapiini column totals**	–	–	10	2	–	–	–	–	–	–	–	–	–	–	–
**Lake Barombi Mbo**															
**Oreochromini**															
Konia eisentrauti				1	4										
Myaka myaka					4										
Pungu maclareni				2*											
Stomatepia mariae					1										
Stomatepia pindu				3	1										
**Oreochromini (Barombi) totals**	–	–	–	6	10	–	–	–	–	–	–	–	–	–	–
**Lake Fwa**															
**Pseudocrenilabrini**															
Cyclopharynx schwetzi				1	12	1									
Schwetzochromis neodon								2							

	Total vertebrae																		
	22	23	24	25	26	27	28	29	30	31	32	33	34	35	36	37	38	39	40
**Pseudocrenilabrini**																			
Astatotilapia bloyeti							1	6	1										
Astatotilapia burtoni							5	1											
Astatotilapia calliptera							1	2	6*										
Astatotilapia swynnertoni							1	2											
Chetia flaviventris										1									
Chetia gracilis											1*								
Chetia ? sp.					8														
Ctenochromis pectoralis								2											
Ctenochromis scatebra									1*										
Haplochromis demeusii						1*													
Haplochromis fasciatus							6												
Haplochromis humilis									3										
Haplochromis moeruensis									4										
Haplochromis oligacanthus					1*														
Lufubuchromis relictus								1*											
Orthochromis machadoi									1	1									
Orthochromis malagaraziensis										2									
Orthochromis polyacanthus										1	1								
Orthochromis stormsi							1	7	2	1									
Palaeoplex palimpsest									1*										
Pharyngochromis acuticeps								1	5										
Pseudocrenilabrus multicolor					1	3													
Pseudocrenilabrus philander							1												
Sargochromis carlottae										2									
Sargochromis codringtonii										1	1	1*							
Sargochromis giardi										1	1								
Sargochromis greenwoodi											2*								
Serranochromis angusticeps														1					
Serranochromis longimanus												4*							
Serranochromis macrocephalus												1							
Serranochromis meridianus												1							
Serranochromis robustus													2	3	1				
Thoracochromis albolabris											1								
Thoracochromis wingatii							1	1											
**Pseudocrenilabrini (riverine) totals**	–	–	–	–	10	4	17	23	24	10	7	7	2	4	1	–	–	–	–
**Steatocranini**																			
Steatocranus casuarius									1	2									
**Tilapiini**																			
Chilochromis duponti							1												
Congolapia bilineata							3												
Tilapia busumana						4													
Tilapia sparrmanii					1*	3													
**Tilapiini column totals**	–	–	–	–	1	7	4	–	–	–	–	–	–	–	–	–	–	–	–
**Lake Barombi Mbo**																			
**Oreochromini**																			
Konia eisentrauti							1	4											
Myaka myaka								4											
Pungu maclareni								2*											
Stomatepia mariae							1												
Stomatepia pindu						1	3												
**Oreochromini (Barombi) totals**	–	–	–	–	–	1	5	10	–	–	–	–	–	–	–	–	–	–	–
**Lake Fwa**																			
**Pseudocrenilabrini**																			
Cyclopharynx schwetzi								12	2										
Schwetzochromis neodon											2								

	Caudal minus precaudal vertebrae																
	−5	−4	−3	−2	−1	0	1	2	3	4	5	6	7	8	9	10	11
**Pseudocrenilabrini**																	
Astatotilapia bloyeti									8	1	2						
Astatotilapia burtoni								5	1								
Astatotilapia calliptera								3	2	4*							
Astatotilapia swynnertoni								1	2								
Chetia flaviventris									1								
Chetia gracilis								1*									
Chetia ? sp.						3	–	5									
Ctenochromis pectoralis											2						
Ctenochromis scatebra										1*							
Haplochromis demeusii							1*										
Haplochromis fasciatus						6											
Haplochromis humilis								3									
Haplochromis moeruensis								2	–	2							
Haplochromis oligacanthus								1*									
Lufubuchromis relictus									1*								
Orthochromis machadoi										1	1						
Orthochromis malagaraziensis											2						
Orthochromis polyacanthus									1	1							
Orthochromis stormsi								2	8	1							
Palaeoplex palimpsest								1*									
Pharyngochromis acuticeps								4	1	1							
Pseudocrenilabrus multicolor							3	1									
Pseudocrenilabrus philander										1							
Sargochromis carlottae					1	–	1										
Sargochromis codringtonii						1	2*										
Sargochromis giardi							1	1									
Sargochromis greenwoodi						2*											
Serranochromis angusticeps									1								
Serranochromis longimanus							4*										
Serranochromis macrocephalus									1								
Serranochromis meridianus									1								
Serranochromis robustus							1	3	2								
Thoracochromis albolabris												1					
Thoracochromis wingatii									1	1							
**Pseudocrenilabrini (riverine) totals**	–	–	–	–	1	12	13	33	28	14	7	1	–	–	–	–	–
**Steatocranini**																	
Steatocranus casuarius				1	–	–	2										
**Tilapiini**																	
Chilochromis duponti				1													
Congolapia bilineata				1	–	2											
Tilapia busumana					4												
Tilapia sparrmanii					3	1*											
**Tilapiini column totals**	–	–	–	2	7	3	–	–	–	–	–	–	–	–	–	–	–
**Lake Barombi Mbo**																	
**Oreochromini**																	
Konia eisentrauti						1	4										
Myaka myaka							4										
Pungu maclareni					2*												
Stomatepia mariae								1									
Stomatepia pindu						2	1	1									
**Oreochromini (Barombi) totals**	–	–	–	–	2	3	9	2	–	–	–	–	–	–	–	–	–
**Lake Fwa**																	
**Pseudocrenilabrini**																	
Cyclopharynx schwetzi					1	1	11	1									
Schwetzochromis neodon										2							

Table 1 (continued). Frequency distribution of vertebral counts (precaudal, caudal, total, and caudal minus precaudal)

	Precaudal vertebrae												
	9	10	11	12	13	14	15	16	17	18	19	20	21
**Lake Albert**													
**Pseudocrenilabrini**													
Haplochromis avium				2	1								
Haplochromis loati					1								
**Lake Edward–George system**													
**Pseudocrenilabrini**													
Haplochromis akika					4*								
Haplochromis aquila					1*								
Haplochromis aureus						1*							
Haplochromis curvidens					1*								
Haplochromis falcatus					1*								
Haplochromis fuscus					1								
Haplochromis glaucus					1*								
Haplochromis gracilifur					1*								
Haplochromis kimondo					1*								
Haplochromis labiatus					1								
Haplochromis latifrons						1*							
Haplochromis limax					4								
Haplochromis mentatus						1*							
Haplochromis molossus						1*							
Haplochromis pappenheimi						1*							
Haplochromis paradoxus						1*							
Haplochromis pardus					1*								
Haplochromis pelagicus							1*						
Haplochromis pharyngalis						1*							
Haplochromis quasimodo						1*							
Haplochromis relictidens					1*								
Haplochromis rex					1*								
Haplochromis simba						1*							
Haplochromis squamipinnis					1*								
Haplochromis taurinus						1*							
Schubotzia eduardiana					9	1							
**Pseudocrenilabrini (Ed–Grg) totals**	–	–	–	–	29	11	1	–	–	–	–	–	–
**Lake Victoria & satellites**													
**Pseudocrenilabrini**													
Allochromis welcommei					1								
Astatoreochromis alluaudi						1							
Haplochromis chlorochrous					4								
Haplochromis cryptogramma				1	8	1							
Haplochromis fusiformis							1						
Haplochromis latifasciatus					4	2							
Haplochromis lividus					1	1							
Haplochromis nubilus					2*	1							
Haplochromis plutonius				1	3	1							
Lithochromis rubripinnis					1	3							
Lithochromis xanthopteryx					1	3							
Mbipia mbipi						1							
Neochromis nigricans					1								
Paralabidochromis victoriae					2								
Pundamilia igneopinnis						2							
Pundamilia pundamilia					4								
Pyxichromis parorthostoma					1								
**Pseudocrenilabrini (Victoria) totals**	–	–	–	2	33	16	1	–	–	–	–	–	–
**Lake Kivu**													
**Pseudocrenilabrini**													
Haplochromis astatodon					4*	2							
Haplochromis paucidens						2							

	Caudal vertebrae														
	11	12	13	14	15	16	17	18	19	20	21	22	23	24	25
**Lake Albert**															
**Pseudocrenilabrini**															
Haplochromis avium						1	2								
Haplochromis loati						1									
**Lake Edward–George system**															
**Pseudocrenilabrini**															
Haplochromis akika							4*								
Haplochromis aquila								1*							
Haplochromis aureus								1*							
Haplochromis curvidens							1*								
Haplochromis falcatus							1*								
Haplochromis fuscus							1								
Haplochromis glaucus							1*								
Haplochromis gracilifur							1*								
Haplochromis kimondo								1*							
Haplochromis labiatus							1								
Haplochromis latifrons							1*								
Haplochromis limax						3	1								
Haplochromis mentatus							1*								
Haplochromis molossus						1*									
Haplochromis pappenheimi							1*								
Haplochromis paradoxus						1*									
Haplochromis pardus							1*								
Haplochromis pelagicus							1*								
Haplochromis pharyngalis							1*								
Haplochromis quasimodo							1*								
Haplochromis relictidens							1*								
Haplochromis rex							1*								
Haplochromis simba								1*							
Haplochromis squamipinnis								1*							
Haplochromis taurinus							1*								
Schubotzia eduardiana						1	8	1							
**Pseudocrenilabrini (Ed–Grg) totals**	–	–	–	–	–	6	29	6	–	–	–	–	–	–	–
**Lake Victoria & satellites**															
**Pseudocrenilabrini**															
Allochromis welcommei								1							
Astatoreochromis alluaudi							1								
Haplochromis chlorochrous							1	3							
Haplochromis cryptogramma							2	6	2						
Haplochromis fusiformis								1							
Haplochromis latifasciatus						1	5								
Haplochromis lividus							2								
Haplochromis nubilus						1	2*								
Haplochromis plutonius							1	3	1						
Lithochromis rubripinnis						2	1	1							
Lithochromis xanthopteryx							3	1							
Mbipia mbipi						1									
Neochromis nigricans							1								
Paralabidochromis victoriae							1	1							
Pundamilia igneopinnis							1	1							
Pundamilia pundamilia						1	2	1							
Pyxichromis parorthostoma						1									
**Pseudocrenilabrini (Victoria) totals**	–	–	–	–	–	7	23	19	3	–	–	–	–	–	–
**Lake Kivu**															
**Pseudocrenilabrini**															
Haplochromis astatodon						5	1*								
Haplochromis paucidens					1	1									

	Total vertebrae																		
	22	23	24	25	26	27	28	29	30	31	32	33	34	35	36	37	38	39	40
**Lake Albert**																			
**Pseudocrenilabrini**																			
Haplochromis avium								3											
Haplochromis loati								1											
**Lake Edward–George system**																			
**Pseudocrenilabrini**																			
Haplochromis akika									4*										
Haplochromis aquila										1*									
Haplochromis aureus											1*								
Haplochromis curvidens									1*										
Haplochromis falcatus									1*										
Haplochromis fuscus									1										
Haplochromis glaucus									1*										
Haplochromis gracilifur									1*										
Haplochromis kimondo										1*									
Haplochromis labiatus									1										
Haplochromis latifrons										1*									
Haplochromis limax								3	1										
Haplochromis mentatus										1*									
Haplochromis molossus									1*										
Haplochromis pappenheimi										1*									
Haplochromis paradoxus									1*										
Haplochromis pardus									1*										
Haplochromis pelagicus											1*								
Haplochromis pharyngalis										1*									
Haplochromis quasimodo										1*									
Haplochromis relictidens									1*										
Haplochromis rex									1*										
Haplochromis simba											1*								
Haplochromis squamipinnis										1*									
Haplochromis taurinus										1*									
Schubotzia eduardiana									9	1									
**Pseudocrenilabrini (Ed–Grg) totals**	–	–	–	–	–	–	–	3	25	10	3	–	–	–	–	–	–	–	–
**Lake Victoria & satellites**																			
**Pseudocrenilabrini**																			
Allochromis welcommei										1									
Astatoreochromis alluaudi										1									
Haplochromis chlorochrous									1	3									
Haplochromis cryptogramma									1	8	1								
Haplochromis fusiformis										1									
Haplochromis latifasciatus									5	1									
Haplochromis lividus									1	1									
Haplochromis nubilus									3*										
Haplochromis plutonius										5									
Lithochromis rubripinnis									2	2									
Lithochromis xanthopteryx										4									
Mbipia mbipi									1										
Neochromis nigricans									1										
Paralabidochromis victoriae									1	1									
Pundamilia igneopinnis										1	1								
Pundamilia pundamilia								1	2	1									
Pyxichromis parorthostoma								1											
**Pseudocrenilabrini (Victoria) totals**	–	–	–	–	–	–	–	2	18	30	2	–	–	–	–	–	–	–	–
**Lake Kivu**																			
**Pseudocrenilabrini**																			
Haplochromis astatodon								3	3*										
Haplochromis paucidens								1	1										

	Caudal minus precaudal vertebrae																
	−5	−4	−3	−2	−1	0	1	2	3	4	5	6	7	8	9	10	11
**Lake Albert**																	
**Pseudocrenilabrini**																	
Haplochromis avium									1	–	2						
Haplochromis loati									1								
**Lake Edward–George system**																	
**Pseudocrenilabrini**																	
Haplochromis akika										4*							
Haplochromis aquila											1*						
Haplochromis aureus										1*							
Haplochromis curvidens										1*							
Haplochromis falcatus										1*							
Haplochromis fuscus										1							
Haplochromis glaucus										1*							
Haplochromis gracilifur										1*							
Haplochromis kimondo											1*						
Haplochromis labiatus										1							
Haplochromis latifrons									1*								
Haplochromis limax									3	1							
Haplochromis mentatus									1*								
Haplochromis molossus								1*									
Haplochromis pappenheimi									1*								
Haplochromis paradoxus								1*									
Haplochromis pardus										1*							
Haplochromis pelagicus								1*									
Haplochromis pharyngalis									1*								
Haplochromis quasimodo									1*								
Haplochromis relictidens										1*							
Haplochromis rex										1*							
Haplochromis simba										1*							
Haplochromis squamipinnis											1*						
Haplochromis taurinus									1*								
Schubotzia eduardiana								1	–	8	1						
**Pseudocrenilabrini (Ed–Grg) totals**	–	–	–	–	–	–	–	4	9	24	4	–	–	–	–	–	–
**Lake Victoria & satellites**																	
**Pseudocrenilabrini**																	
Allochromis welcommei											1						
Astatoreochromis alluaudi									1								
Haplochromis chlorochrous										1	3						
Haplochromis cryptogramma									1	1	6	1	1				
Haplochromis fusiformis									1								
Haplochromis latifasciatus									1	5							
Haplochromis lividus									1	1							
Haplochromis nubilus								1	–	2*							
Haplochromis plutonius									1	–	3	–	1				
Lithochromis rubripinnis								2	1	–	1						
Lithochromis xanthopteryx									3	–	1						
Mbipia mbipi								1									
Neochromis nigricans										1							
Paralabidochromis victoriae										1	1						
Pundamilia igneopinnis									1	1							
Pundamilia pundamilia									1	2	1						
Pyxichromis parorthostoma									1								
**Pseudocrenilabrini (Victoria) totals**	–	–	–	–	–	–	–	4	13	13	17	1	2	–	–	–	–
**Lake Kivu**																	
**Pseudocrenilabrini**																	
Haplochromis astatodon								2	3	1							
Haplochromis paucidens							1	1									

Table 1 (continued). Frequency distribution of vertebral counts (precaudal, caudal, total, and caudal minus precaudal)

Precaudal vertebrae

	9	10	11	12	13	14	15	16	17	18	19	20	21
Lake Turkana													
**Pseudocrenilabrini**													
Haplochromis rudolfianus					7	1							
Haplochromis turkanae					3								
Lake Tanganyika													
**Bathybatini**													
Bathybates fasciatus										5			
Bathybates ferox							10						
Bathybates graueri							5						
Bathybates hornii							1						
Bathybates leo								5					
Bathybates minor							5						
Bathybates vittatus								1					
Hemibates stenosoma								4					
Trematocara unimaculatum			2	3									
Trematocara zebra				5									
**Bathybatini column totals**	–	–	2	8	–	–	21	10	–	5	–	–	–
**Benthochromini**													
Benthochromis tricoti									2	3			
**Boulengerochromini**													
Boulengerochromis microlepis								7					
**Cyphotilapiini**													
Cyphotilapia frontosa							1	4					
Cyphotilapia gibberosa								4	1				
**Cyprichromini**													
Cyprichromis coloratus												4	1
Cyprichromis leptosoma									1	3	1		
Cyprichromis microlepidotus										2	3		
Cyprichromis pavo												2	3
Cyprichromis zonatus											5		
Cyprichromis sp. "dwarf jumbo"										2	–	3	
Cyprichromis sp. "jumbo"											1	4	
Paracyprichromis brieni								3	2				
Paracyprichromis nigripinnis								2	2				
Paracyprichromis sp. "brieni south"									5				
**Cyprichromini column totals**	–	–	–	–	–	–	–	5	10	7	10	13	4
**Ectodini**													
Asprotilapia leptura							2	3					
Aulonocranus dewindti						2	3						
Callochromis macrops						5							
Cardiopharynx schoutedeni								4	1				
Cunningtonia longiventralis								4	1				
Cyathopharynx furcifer								5					
Ectodus descampsii							4	1					
Enantiopus melanogenys						5							
Grammatotria lemairii						4	1						
Lestradea perspicax								2	3				
Microdontochromis tenuidentatus						5							
Ophthalmotilapia boops								4	1				
Xenotilapia sima					5								
**Ectodini column totals**	–	–	–	–	5	21	10	23	6	–	–	–	–
**Eretmodini**													
Eretmodus cyanostictus					1	4							
Eretmodus marksmithi					3	2							
Spathodus erythrodon					5								
Tanganicodus irsacae					5								
**Eretmodini column totals**	–	–	–	–	14	6	–	–	–	–	–	–	–

Caudal vertebrae

	11	12	13	14	15	16	17	18	19	20	21	22	23	24	25
Lake Turkana															
**Pseudocrenilabrini**															
Haplochromis rudolfianus						3	4	1							
Haplochromis turkanae						3									
Lake Tanganyika															
**Bathybatini**															
Bathybates fasciatus											1	4			
Bathybates ferox										9	1				
Bathybates graueri									4	1					
Bathybates hornii										1					
Bathybates leo										5					
Bathybates minor											4	1			
Bathybates vittatus										1					
Hemibates stenosoma									4						
Trematocara unimaculatum								3	2						
Trematocara zebra								5							
**Bathybatini column totals**	–	–	–	–	–	–	–	8	10	17	6	5	–	–	–
**Benthochromini**															
Benthochromis tricoti								2	3						
**Boulengerochromini**															
Boulengerochromis microlepis								6	1						
**Cyphotilapiini**															
Cyphotilapia frontosa						4	1								
Cyphotilapia gibberosa					1	4									
**Cyprichromini**															
Cyprichromis coloratus								3	2						
Cyprichromis leptosoma								2	3						
Cyprichromis microlepidotus							1	4							
Cyprichromis pavo								2	3						
Cyprichromis zonatus								2	3						
Cyprichromis sp. "dwarf jumbo"							1	2	2						
Cyprichromis sp. "jumbo"								1	4						
Paracyprichromis brieni									2	3					
Paracyprichromis nigripinnis								1	3						
Paracyprichromis sp. "brieni south"								2	3						
**Cyprichromini column totals**	–	–	–	–	–	–	2	19	25	3	–	–	–	–	–
**Ectodini**															
Asprotilapia leptura										1	2	2			
Aulonocranus dewindti							3	2							
Callochromis macrops								1	4						
Cardiopharynx schoutedeni								3	2						
Cunningtonia longiventralis								1	3	1					
Cyathopharynx furcifer							2	3							
Ectodus descampsii							3	2							
Enantiopus melanogenys														2	3
Grammatotria lemairii												1	4		
Lestradea perspicax									3	2					
Microdontochromis tenuidentatus											4	1			
Ophthalmotilapia boops									5						
Xenotilapia sima												5			
**Ectodini column totals**	–	–	–	–	–	–	8	12	17	4	6	9	4	2	3
**Eretmodini**															
Eretmodus cyanostictus						5									
Eretmodus marksmithi						2	3								
Spathodus erythrodon							5								
Tanganicodus irsacae							5								
**Eretmodini column totals**	–	–	–	–	–	7	13	–	–	–	–	–	–	–	–

Total vertebrae

	22	23	24	25	26	27	28	29	30	31	32	33	34	35	36	37	38	39	40
Lake Turkana																			
**Pseudocrenilabrini**																			
Haplochromis rudolfianus								2	5	1									
Haplochromis turkanae								3											
Lake Tanganyika																			
**Bathybatini**																			
Bathybates fasciatus																		1	4
Bathybates ferox														9	1				
Bathybates graueri													4	1					
Bathybates hornii														1					
Bathybates leo															5				
Bathybates minor															4	1			
Bathybates vittatus															1				
Hemibates stenosoma														4					
Trematocara unimaculatum									5										
Trematocara zebra									5										
**Bathybatini column totals**	–	–	–	–	–	–	–	–	10	–	–	–	4	15	11	1	–	1	4
**Benthochromini**																			
Benthochromis tricoti														1	2	2			
**Boulengerochromini**																			
Boulengerochromis microlepis													6	1					
**Cyphotilapiini**																			
Cyphotilapia frontosa											5								
Cyphotilapia gibberosa											5								
**Cyprichromini**																			
Cyprichromis coloratus																	2	3	
Cyprichromis leptosoma															2	3			
Cyprichromis microlepidotus															3	2			
Cyprichromis pavo																		4	1
Cyprichromis zonatus																2	3		
Cyprichromis sp. "dwarf jumbo"																3	2		
Cyprichromis sp. "jumbo"																	2	3	
Paracyprichromis brieni															5				
Paracyprichromis nigripinnis														3	1				
Paracyprichromis sp. "brieni south"														2	3				
**Cyprichromini column totals**	–	–	–	–	–	–	–	–	–	–	–	–	–	5	14	10	9	10	1
**Ectodini**																			
Asprotilapia leptura															1	4			
Aulonocranus dewindti											5								
Callochromis macrops											1	4							
Cardiopharynx schoutedeni													2	3					
Cunningtonia longiventralis														4	1				
Cyathopharynx furcifer												2	3						
Ectodus descampsii											2	3							
Enantiopus melanogenys																	2	3	
Grammatotria lemairii																5			
Lestradea perspicax															5				
Microdontochromis tenuidentatus														4	1				
Ophthalmotilapia boops															1	4			
Xenotilapia sima														5					
**Ectodini column totals**	–	–	–	–	–	–	–	–	–	–	8	9	5	16	9	13	2	3	–
**Eretmodini**																			
Eretmodus cyanostictus								1	4										
Eretmodus marksmithi									5										
Spathodus erythrodon									5										
Tanganicodus irsacae									5										
**Eretmodini column totals**	–	–	–	–	–	–	–	1	19	–	–	–	–	–	–	–	–	–	–

Caudal minus precaudal vertebrae

	−5	−4	−3	−2	−1	0	1	2	3	4	5	6	7	8	9	10	11
Lake Turkana																	
**Pseudocrenilabrini**																	
Haplochromis rudolfianus								1	2	4	1						
Haplochromis turkanae									3								
Lake Tanganyika																	
**Bathybatini**																	
Bathybates fasciatus									1	4							
Bathybates ferox											9	1					
Bathybates graueri										4	1						
Bathybates hornii											1						
Bathybates leo										5							
Bathybates minor												4	1				
Bathybates vittatus										1							
Hemibates stenosoma									4								
Trematocara unimaculatum												3	–	2			
Trematocara zebra												5					
**Bathybatini column totals**	–	–	–	–	–	–	–	–	5	14	11	13	1	2	–	–	–
**Benthochromini**																	
Benthochromis tricoti						1	3	1									
**Boulengerochromini**																	
Boulengerochromis microlepis								6	1								
**Cyphotilapiini**																	
Cyphotilapia frontosa						4	–	1									
Cyphotilapia gibberosa				1	–	4											
**Cyprichromini**																	
Cyprichromis coloratus			1	2	2												
Cyprichromis leptosoma					1	1	2	1									
Cyprichromis microlepidotus				1	2	2											
Cyprichromis pavo			2	1	2												
Cyprichromis zonatus					2	3											
Cyprichromis sp. "dwarf jumbo"			1	2	–	–	2										
Cyprichromis sp. "jumbo"				1	3	1											
Paracyprichromis brieni								2	–	3							
Paracyprichromis nigripinnis							1	1	2								
Paracyprichromis sp. "brieni south"							2	3									
**Cyprichromini column totals**	–	–	4	7	12	7	7	7	2	3	–	–	–	–	–	–	–
**Ectodini**																	
Asprotilapia leptura										1	2	–	2				
Aulonocranus dewindti								3	–	2							
Callochromis macrops										1	4						
Cardiopharynx schoutedeni							1	2	2								
Cunningtonia longiventralis							1	–	3	1							
Cyathopharynx furcifer							2	3									
Ectodus descampsii							1	2	2								
Enantiopus melanogenys																2	3
Grammatotria lemairii													1	–	4		
Lestradea perspicax								3	–	2							
Microdontochromis tenuidentatus													4	1			
Ophthalmotilapia boops								1	4								
Xenotilapia sima															5		
**Ectodini column totals**	–	–	–	–	–	–	5	14	11	7	6	–	7	1	9	2	3
**Eretmodini**																	
Eretmodus cyanostictus								4	1								
Eretmodus marksmithi								2	–	3							
Spathodus erythrodon										5							
Tanganicodus irsacae										5							
**Eretmodini column totals**	–	–	–	–	–	–	–	6	1	13	–	–	–	–	–	–	–

Table 1 (continued). Frequency distribution of vertebral counts (precaudal, caudal, total, and caudal minus precaudal)

Precaudal vertebrae

	9	10	11	12	13	14	15	16	17	18	19	20	21
**Lamprologini**													
Altolamprologus calvus					5								
Altolamprologus compressiceps					5								
Altolamp. 'compressiceps shell'				5									
Chalinochromis brichardi									5				
Chalinochromis cyanophleps								1	4				
Julidochromis ornatus							1	4					
Julidochromis regani								5					
Lamprologus callipterus						10							
Lamprologus 'ornatipinnis congo'				5									
Lamprologus 'ornatipinnis zambia'				9									
Lamprologus speciosus			3	2									
Lepidiolamprologus kendalli								5					
Lepidiolamprologus mimicus								5					
Lepidiolamprologus profundicola								5					
Neolamprologus tretocephalus						5							
Neolamprologus ventralis					1	4							
Telmatochromis dhonti						4	1						
Telmatochromis temporalis						1	4						
Telmatochromis vittatus								3	2				
Variabilichromis moorii								7					
**Lamprologini column totals**	–	–	3	21	11	24	6	35	11	–	–	–	–
**Limnochromini**													
Baileychromis centropomoides							5						
Gnathochromis permaxillaris							5						
Greenwoodochromis christyi						5							
Limnochromis auritus						2	3						
Reganochromis calliurus							2	3					
Tangachromis dhanisi					3	1							
Trematochromis benthicola						5							
Triglachromis otostigma						2	3						
**Limnochromini column totals**	–	–	–	–	3	15	18	3	–	–	–	–	–
**Oreochromini**													
Oreochromis tanganicae								5					
**Perissodini**													
Haplotaxodon microlepis									5				
Perissodus microlepis							1	4					
Plecodus paradoxus									1	4			
Xenochromis hecqui							4	1					
**Perissodini column totals**	–	–	–	–	–	–	5	5	6	4	–	–	–
**Pseudocrenilabrini: Tropheina**													
Interochromis loocki							5						
Jabarichromis pfefferi					2	3							
Limnotilapia dardennii								4					
Lobochilotes labiata							5						
Petrochromis horii								5					
Petrochromis polyodon							1	4					
Pseudosimochromis curvifrons							5						
Shuja horei					8								
Simochromis diagramma							3	2					
Tropheus annectens							4	1					
Tropheus brichardi						2	3						
Tropheus duboisi							5						
Tropheus moorii							10						
**Tropheina column totals**	–	–	–	–	10	5	41	16	–	–	–	–	–
**Tylochromini**													
Tylochromis polylepis						2	4						

Caudal vertebrae

	11	12	13	14	15	16	17	18	19	20	21	22	23	24	25
**Lamprologini**															
Altolamprologus calvus								5							
Altolamprologus compressiceps							2	3							
Altolamp. 'compressiceps shell'							1	4							
Chalinochromis brichardi							5								
Chalinochromis cyanophleps								1	4						
Julidochromis ornatus							4	1							
Julidochromis regani							3	2							
Lamprologus callipterus										9	1				
Lamprologus 'ornatipinnis congo'											4	1			
Lamprologus 'ornatipinnis zambia'										1	6	2			
Lamprologus speciosus						3	2								
Lepidiolamprologus kendalli								4	1						
Lepidiolamprologus mimicus									4	1					
Lepidiolamprologus profundicola										4	1				
Neolamprologus tretocephalus								4	1						
Neolamprologus ventralis								4	1						
Telmatochromis dhonti								5							
Telmatochromis temporalis							1	3	1						
Telmatochromis vittatus								3	2						
Variabilichromis moorii						5	2								
**Lamprologini column totals**	–	–	–	–	–	8	20	39	14	15	12	3	–	–	–
**Limnochromini**															
Baileychromis centropomoides								4	1						
Gnathochromis permaxillaris							1	4							
Greenwoodochromis christyi							3	2							
Limnochromis auritus								4	1						
Reganochromis calliurus									5						
Tangachromis dhanisi							3	1							
Trematochromis benthicola							4	1							
Triglachromis otostigma							2	3							
**Limnochromini column totals**	–	–	–	–	–	–	13	19	7	–	–	–	–	–	–
**Oreochromini**															
Oreochromis tanganicae					5										
**Perissodini**															
Haplotaxodon microlepis									4	1					
Perissodus microlepis								3	2						
Plecodus paradoxus										4	1				
Xenochromis hecqui								1	4						
**Perissodini column totals**	–	–	–	–	–	–	–	4	10	5	1	–	–	–	–
**Pseudocrenilabrini: Tropheina**															
Interochromis loocki						5									
Jabarichromis pfefferi						3	2								
Limnotilapia dardennii								4							
Lobochilotes labiata							5								
Petrochromis horii						3	2								
Petrochromis polyodon						4	1								
Pseudosimochromis curvifrons						4	1								
Shuja horei						2	6								
Simochromis diagramma						1	3	1							
Tropheus annectens					1	4									
Tropheus brichardi					5										
Tropheus duboisi					4	1									
Tropheus moorii					3	7									
**Tropheina column totals**	–	–	–	–	13	34	20	5	–	–	–	–	–	–	–
**Tylochromini**															
Tylochromis polylepis						4	2								

Total vertebrae

	22	23	24	25	26	27	28	29	30	31	32	33	34	35	36	37	38	39	40
**Lamprologini**																			
Altolamprologus calvus										5									
Altolamprologus compressiceps									2	3									
Altolamp. 'compressiceps shell'								1	4										
Chalinochromis brichardi													5						
Chalinochromis cyanophleps														2	3				
Julidochromis ornatus												5							
Julidochromis regani												3	2						
Lamprologus callipterus													9	1					
Lamprologus 'ornatipinnis congo'												4	1						
Lamprologus 'ornatipinnis zambia'											1	6	2						
Lamprologus speciosus						1	4												
Lepidiolamprologus kendalli													4	1					
Lepidiolamprologus mimicus														4	1				
Lepidiolamprologus profundicola															4	1			
Neolamprologus tretocephalus											4	1							
Neolamprologus ventralis											5								
Telmatochromis dhonti											4	1							
Telmatochromis temporalis											1	4							
Telmatochromis vittatus													2	2	1				
Variabilichromis moorii											5	2							
**Lamprologini column totals**	–	–	–	–	–	1	4	1	6	8	20	26	25	10	9	1	–	–	–
**Limnochromini**																			
Baileychromis centropomoides												4	1						
Gnathochromis permaxillaris											1	4							
Greenwoodochromis christyi										3	2								
Limnochromis auritus											1	4							
Reganochromis calliurus													2	3					
Tangachromis dhanisi									3		1								
Trematochromis benthicola										4	1								
Triglachromis otostigma											4	1							
**Limnochromini column totals**	–	–	–	–	–	–	–	–	3	7	10	13	3	3	–	–	–	–	–
**Oreochromini**																			
Oreochromis tanganicae										5									
**Perissodini**																			
Haplotaxodon microlepis															4	1			
Perissodus microlepis													4	1					
Plecodus paradoxus																1	3	1	
Xenochromis hecqui													5						
**Perissodini column totals**	–	–	–	–	–	–	–	–	–	–	–	–	9	1	4	2	3	1	–
**Pseudocrenilabrini: Tropheina**																			
Interochromis loocki										5									
Jabarichromis pfefferi									5										
Limnotilapia dardennii													4						
Lobochilotes labiata											5								
Petrochromis horii											3	2							
Petrochromis polyodon											5								
Pseudosimochromis curvifrons										4	1								
Shuja horei								2	6										
Simochromis diagramma											3	2							
Tropheus annectens										5									
Tropheus brichardi								2	3										
Tropheus duboisi									4	1									
Tropheus moorii									3	7									
**Tropheina column totals**	–	–	–	–	–	–	–	4	21	22	17	4	4	–	–	–	–	–	–
**Tylochromini**																			
Tylochromis polylepis										6									

Caudal minus precaudal vertebrae

	−5	−4	−3	−2	−1	0	1	2	3	4	5	6	7	8	9	10	11
**Lamprologini**																	
Altolamprologus calvus											5						
Altolamprologus compressiceps										2	3						
Altolamp. 'compressiceps shell'											1	4					
Chalinochromis brichardi						5											
Chalinochromis cyanophleps							1	3	1								
Julidochromis ornatus							4	–	1								
Julidochromis regani							3	2									
Lamprologus callipterus												9	1				
Lamprologus 'ornatipinnis congo'															4	1	
Lamprologus 'ornatipinnis zambia'														1	6	2	
Lamprologus speciosus										2	1	2					
Lepidiolamprologus kendalli								4	1								
Lepidiolamprologus mimicus									4	1							
Lepidiolamprologus profundicola										4	1						
Neolamprologus tretocephalus										4	1						
Neolamprologus ventralis										4	–	1					
Telmatochromis dhonti									1	4							
Telmatochromis temporalis								1	3	–	1						
Telmatochromis vittatus							1	3	1								
Variabilichromis moorii						5	2										
**Lamprologini column totals**	–	–	–	–	–	10	11	13	12	21	13	16	1	1	10	3	–
**Limnochromini**																	
Baileychromis centropomoides									4	1							
Gnathochromis permaxillaris								1	4								
Greenwoodochromis christyi									3	2							
Limnochromis auritus									3	1	1						
Reganochromis calliurus									3	2							
Tangachromis dhanisi										4							
Trematochromis benthicola									4	1							
Triglachromis otostigma								2	1	2							
**Limnochromini column totals**	–	–	–	–	–	–	–	3	22	13	1	–	–	–	–	–	–
**Oreochromini**																	
Oreochromis tanganicae					5												
**Perissodini**																	
Haplotaxodon microlepis								4	1								
Perissodus microlepis								3	1	1							
Plecodus paradoxus								3	2								
Xenochromis hecqui								1	–	4							
**Perissodini column totals**	–	–	–	–	–	–	–	11	4	5	–	–	–	–	–	–	–
**Pseudocrenilabrini: Tropheina**																	
Interochromis loocki							5										
Jabarichromis pfefferi								3	–	2							
Limnotilapia dardennii								4									
Lobochilotes labiata								5									
Petrochromis horii						3	2										
Petrochromis polyodon						4		1									
Pseudosimochromis curvifrons							4	1									
Shuja horei									2	6							
Simochromis diagramma						1	1	2	1								
Tropheus annectens					1		4										
Tropheus brichardi						3	2										
Tropheus duboisi						4	1										
Tropheus moorii						3	7										
**Tropheina column totals**	–	–	–	–	1	18	26	16	3	8	–	–	–	–	–	–	–
**Tylochromini**																	
Tylochromis polylepis							4	–	2								

Table 1 (continued). Frequency distribution of vertebral counts (precaudal, caudal, total, and caudal minus precaudal)

	Precaudal vertebrae													Caudal vertebrae															Total vertebrae																			Caudal minus precaudal vertebrae																
	9	10	11	12	13	14	15	16	17	18	19	20	21	11	12	13	14	15	16	17	18	19	20	21	22	23	24	25	22	23	24	25	26	27	28	29	30	31	32	33	34	35	36	37	38	39	40	−5	−4	−3	−2	−1	0	1	2	3	4	5	6	7	8	9	10	11
Lake Malawi																																																																	
**Pseudocrenilabrini: Cyrtocarina**																																																																	
Alticorpus mentale					1																	1																	1																					1					
Alticorpus peterdaviesi				1																		1																1																						1				
Aristochromis christyi						2																2																			2																			2						
Aulonocara nyassae					1																1																1																				1						
Aulonocara rostratum					1																	1																	1																					1					
Aulonocara stonemani					1*																1*																1*																				1*						
Buccochromis atritaeniatus						2*														2*																	2*																			2*							
Buccochromis heterotaenia							2*													2*																			2*																	2*								
Buccochromis nototaenia							2*													1*	1																			1*	1															1*	1							
Buccochromis oculatus							3													2	1																			2	1															2	1							
Buccochromis spectabilis							1																1																					1																	1							
Caprichromis orthognathus						4	1												1	2	2																	1	2	1	1														1	3	1							
Champsochromis caeruleus							3	1														1	3																				4														1	–	3						
Champsochromis spilorhynchus								2												2																			2															2									
Cheilochromis euchilus					2*	3												1	4*																	3*	2																1	2	2*							
Chilotilapia rhoadesii						4														4																	4																		4								
Copadichromis jacksoni							3													3																			3																	3								
Copadichromis quadrimaculatus							1												1																			1																	1									
Corematodus taeniatus					4	3													3	4																7																		3	–	4						
Ctenopharynx nitidus						5	11													10	6																			15	1														10	1	5							
Ctenopharynx pictus						1														1																	1																		1								
Cyrtocara moorii						6*														1	4	1*																	1	4	1*																1	4	1*						
Dimidiochromis compressiceps					4	8														8	4																	1	10	1																7	2	3					
Dimidiochromis kiwinge							3													3																			3																	3								
Docimodus evelynae							2*													2*																			2*																	2*								
Docimodus johnstoni						3														2	1																	2	1																		2	1						
Exochochromis anagenys					1	5	1*													6*	1																	6	1*																1*	5	–	1					
Fossorochromis rostratus						1															1																	1																				1							
Hemitaeniochromis urotaenia						1														1																	1																		1								
Hemitilapia oxyrhynchus						8	2												1	9																	1	7	2															3	7								
Lethrinops gossei					1*	1																2*																	1*	1																		1	1*						
Lethrinops lethrinus					3													1	2																1	2																			1	2							
Lethrinops polli					1*																1*																1*																				1*						
Lichnochromis acuticeps						1														1																	1																		1								
Mchenga cyclicos							1													1																			1																	1								
Mchenga inornata						1*	1															1	1*																			2*																	1	–	1*						
Mchenga sp.							3	20												1	10	12																		1	13	9											1	10	9	3								
Mylochromis formosus							1*	1													1	–	1*																			1	1*													1	–	–	1*							
Mylochromis gracilis							3*															2*	1																				2*	1																2*	1						
Mylochromis guentheri							2												1	1																		1	1															1	1									
Mylochromis lateristriga						4														4																	4																		4								
Mylochromis spilostichus							5	3*														7*	1																				4	4*														3*	4	1						
Mylochromis subocularis						2														1	1																1	1																	1	1							
Naevochromis chrysogaster						5*														5*																	5*																		5*								
Nimbochromis fuscotaeniatus						2												1		1																1	–	1															1	–	1							
Nimbochromis livingstonii						5	1														6																	5	1																	1	5								
Nimbochromis venustus						4														4																	4																		4								
Nyassachromis leuciscus						6*																5*	1																	5*	1																		5*	1						
Nyassachromis microcephalus							1	1*														1*	1																			2*															1*	–	1								
Nyassachromis nigritaeniatus							3	1														2	2																			1	3														1	1	2								
Nyassachromis purpurans							1													1																			1																	1								
Otopharynx argyrosoma							3*													1*	2																			1*	2															1*	2								
Otopharynx decorus								6*													3	3*																				3	3*														3	3*								
Otopharynx cf. *heterodon*						18	1													18	1																17	2																	1	17	1							
Otopharynx heterodon							8											1	6	1																	1	6	1														1	6	1									
Otopharynx lithobates						5*												1	4*																	1	4*																	1	4*								

Table 1 (continued). Frequency distribution of vertebral counts (precaudal, caudal, total, and caudal minus precaudal)

Precaudal vertebrae	9	10	11	12	13	14	15	16	17	18	19	20	21
Otopharynx ovatus						1	4*						
Otopharynx selenurus						1	5						
Otopharynx speciosus					2	2	1						
Otopharynx tetraspilus						3	5						
Otopharynx tetrastigma					8*	8							
Placidochromis hennydaviesae					1*								
Placidochromis johnstoni					2	2							
Placidochromis longimanus						4	24						
Placidochromis macrognathus					1	1							
Placidochromis milomo						2	2						
Protomelas annectens							2						
Protomelas fenestratus					4	27	5						
Protomelas insignis							2						
Protomelas kirkii					2*	1							
Protomelas labridens						1							
Protomelas ornatus					2*								
Protomelas spilopterus					1	3	2						
Protomelas taeniolatus						1							
Protomelas triaenodon						3							
Sciaenochromis ahli					11	5							
Stigmatochromis modestus					4*	1							
Stigmatochromis pholidophorus					2*	3							
Stigmatochromis pleurospilus					1*								
Stigmatochromis woodi					5	2*							
Taeniochromis holotaenia							1						
Taeniolethrinops praeorbitalis							1						
Tramitichromis brevis						1							
Trematocranus labifer						4							
Trematocranus microstoma					5	3	1						
Trematocranus placodon					3								
Tyrannochromis macrostoma						1							
Tyrannochromis nigriventer						1*							
**Cyrtocarina column totals**	**–**	**–**	**–**	**1**	**74**	**191**	**124**	**35**	**–**	**–**	**–**	**–**	**–**
**Pseudocrenilabrini: Pseudotropheina**													
Abactochromis labrosus					6*								
Chindongo bellicosus							4	2					
Chindongo minutus						1							
Cyathochromis obliquidens					1	3							
Cynotilapia afra							1						
Cynotilapia sp.						2	2						
Genyochromis mento						8	2						
Gephyrochromis lawsi							1						
Gephyrochromis moorii						2	2						
Iodotropheus sprengerae						6							
Labeotropheus fuelleborni						5	10						
Labeotropheus trewavasae								3					
Labidochromis caeruleus						1							
Labidochromis freibergi						1*							
Labidochromis joanjohnsonae					1*								
Labidochromis pallidus					5	18							
Labidochromis shiranus					1								
Labidochromis textilis						2*							
Labidochromis vellicans					5	25							
Maylandia aurora						1*							
Maylandia koningsi							1						
Maylandia lanisticola						1*							
Maylandia zebra							7	2					
Melanochromis auratus						2	4*						

Caudal vertebrae	11	12	13	14	15	16	17	18	19	20	21	22	23	24	25
Otopharynx ovatus							3*	2							
Otopharynx selenurus								4	2						
Otopharynx speciosus							2	3							
Otopharynx tetraspilus						7	1								
Otopharynx tetrastigma						4	11*	1							
Placidochromis hennydaviesae							1*								
Placidochromis johnstoni						1	1	2							
Placidochromis longimanus						2	25	1							
Placidochromis macrognathus							1	1							
Placidochromis milomo					1	3									
Protomelas annectens								2							
Protomelas fenestratus						12	24								
Protomelas insignis							2								
Protomelas kirkii						1	2*								
Protomelas labridens							1								
Protomelas ornatus							2*								
Protomelas spilopterus						3	3								
Protomelas taeniolatus							1								
Protomelas triaenodon							2	1							
Sciaenochromis ahli							4	12							
Stigmatochromis modestus							4*	1							
Stigmatochromis pholidophorus							2	3*							
Stigmatochromis pleurospilus								1*							
Stigmatochromis woodi							3*	3	1						
Taeniochromis holotaenia								1							
Taeniolethrinops praeorbitalis								1							
Tramitichromis brevis							1								
Trematocranus labifer							4								
Trematocranus microstoma						1	–	7	1						
Trematocranus placodon							1	2							
Tyrannochromis macrostoma								1							
Tyrannochromis nigriventer								1*							
**Cyrtocarina column totals**	**–**	**–**	**–**	**–**	**2**	**48**	**199**	**136**	**35**	**5**	**–**	**–**	**–**	**–**	**–**
**Pseudocrenilabrini: Pseudotropheina**															
Abactochromis labrosus					1	5*									
Chindongo bellicosus						4	2								
Chindongo minutus					1										
Cyathochromis obliquidens						2	2								
Cynotilapia afra						1									
Cynotilapia sp.					3	1									
Genyochromis mento						2	8								
Gephyrochromis lawsi						1									
Gephyrochromis moorii						1	3								
Iodotropheus sprengerae					2	4									
Labeotropheus fuelleborni					3	10	2								
Labeotropheus trewavasae						1	2								
Labidochromis caeruleus						1									
Labidochromis freibergi						1*									
Labidochromis joanjohnsonae							1*								
Labidochromis pallidus					5	17	1								
Labidochromis shiranus							1								
Labidochromis textilis						1*	1								
Labidochromis vellicans					2	25	3								
Maylandia aurora							1*								
Maylandia koningsi					1										
Maylandia lanisticola						1*									
Maylandia zebra					4	5									
Melanochromis auratus						3	3*								

Total vertebrae	22	23	24	25	26	27	28	29	30	31	32	33	34	35	36	37	38	39	40
Otopharynx ovatus											4*	1							
Otopharynx selenurus												5	1						
Otopharynx speciosus									2	–	2	1							
Otopharynx tetraspilus									3	4	1								
Otopharynx tetrastigma								1	9*	6									
Placidochromis hennydaviesae									1*										
Placidochromis johnstoni									1	3									
Placidochromis longimanus										5	23								
Placidochromis macrognathus										2									
Placidochromis milomo									3	1									
Protomelas annectens												2							
Protomelas fenestratus								1	9	26									
Protomelas insignis											2								
Protomelas kirkii									3*										
Protomelas labridens										1									
Protomelas ornatus									2*										
Protomelas spilopterus									3	2	1								
Protomelas taeniolatus										1									
Protomelas triaenodon										2	1								
Sciaenochromis ahli										15	1								
Stigmatochromis modestus									3*	2									
Stigmatochromis pholidophorus										4*	1								
Stigmatochromis pleurospilus										1*									
Stigmatochromis woodi									1	5*	1								
Taeniochromis holotaenia												1							
Taeniolethrinops praeorbitalis												1							
Tramitichromis brevis										1									
Trematocranus labifer										4									
Trematocranus microstoma									1	5	1	2							
Trematocranus placodon									1	2									
Tyrannochromis macrostoma											1								
Tyrannochromis nigriventer											1*								
**Cyrtocarina column totals**	**–**	**–**	**–**	**–**	**–**	**–**	**–**	**3**	**52**	**171**	**102**	**60**	**25**	**12**	**–**	**–**	**–**	**–**	**–**
**Pseudocrenilabrini: Pseudotropheina**																			
Abactochromis labrosus							1	5*											
Chindongo bellicosus										2	4								
Chindongo minutus								1											
Cyathochromis obliquidens									3	1									
Cynotilapia afra										1									
Cynotilapia sp.								1	3										
Genyochromis mento									1	8	1								
Gephyrochromis lawsi										1									
Gephyrochromis moorii										3	1								
Iodotropheus sprengerae								2	4										
Labeotropheus fuelleborni									6	9									
Labeotropheus trewavasae											1	2							
Labidochromis caeruleus									1										
Labidochromis freibergi									1*										
Labidochromis joanjohnsonae									1*										
Labidochromis pallidus								9	14										
Labidochromis shiranus									1										
Labidochromis textilis									1*	1									
Labidochromis vellicans								5	23	2									
Maylandia aurora										1*									
Maylandia koningsi									1										
Maylandia lanisticola									1*										
Maylandia zebra									3	5	1								
Melanochromis auratus										5	1*								

Caudal minus precaudal vertebrae	−5	−4	−3	−2	−1	0	1	2	3	4	5	6	7	8	9	10	11
Otopharynx ovatus								3*	1	1							
Otopharynx selenurus									4	1	1						
Otopharynx speciosus									1	4							
Otopharynx tetraspilus							4	4									
Otopharynx tetrastigma								3	6	6*	1						
Placidochromis hennydaviesae										1*							
Placidochromis johnstoni								1	1	–	2						
Placidochromis longimanus							2	22	3	1							
Placidochromis macrognathus									1	–	1						
Placidochromis milomo						1	1	2									
Protomelas annectens									2								
Protomelas fenestratus							5	6	22	3							
Protomelas insignis								2									
Protomelas kirkii								1	–	2*							
Protomelas labridens									1								
Protomelas ornatus										2*							
Protomelas spilopterus							1	3	1	1							
Protomelas taeniolatus									1								
Protomelas triaenodon									2	1							
Sciaenochromis ahli									4	1	11						
Stigmatochromis modestus									1	3*	1						
Stigmatochromis pholidophorus									2	1	2*						
Stigmatochromis pleurospilus											1*						
Stigmatochromis woodi									2*	1	3	1					
Taeniochromis holotaenia									1								
Taeniolethrinops praeorbitalis									1								
Tramitichromis brevis									1								
Trematocranus labifer									4								
Trematocranus microstoma								1	1	1	6						
Trematocranus placodon										1	2						
Tyrannochromis macrostoma										1							
Tyrannochromis nigriventer										1*							
**Cyrtocarina column totals**	**–**	**–**	**–**	**–**	**–**	**3**	**33**	**86**	**160**	**83**	**52**	**7**	**1**	**–**	**–**	**–**	**–**
**Pseudocrenilabrini: Pseudotropheina**																	
Abactochromis labrosus								1	5*								
Chindongo bellicosus						2	2	2									
Chindongo minutus							1										
Cyathochromis obliquidens								2	1	1							
Cynotilapia afra					1												
Cynotilapia sp.						2	1	1									
Genyochromis mento							1	2	7								
Gephyrochromis lawsi							1										
Gephyrochromis moorii							1	1	2								
Iodotropheus sprengerae							2	4									
Labeotropheus fuelleborni						3	7	3	2								
Labeotropheus trewavasae						1	1	1									
Labidochromis caeruleus								1									
Labidochromis freibergi								1*									
Labidochromis joanjohnsonae										1*							
Labidochromis pallidus							5	13	4	1							
Labidochromis shiranus										1							
Labidochromis textilis								1*	1								
Labidochromis vellicans							2	21	6	1							
Maylandia aurora									1*								
Maylandia koningsi						1											
Maylandia lanisticola								1*									
Maylandia zebra					1	4	4										
Melanochromis auratus							3	1*	2								

Table 1 (continued). Frequency distribution of vertebral counts (precaudal, caudal, total, and caudal minus precaudal)

	Precaudal vertebrae													Caudal vertebrae															Total vertebrae																			Caudal minus precaudal vertebrae																
	9	10	11	12	13	14	15	16	17	18	19	20	21	11	12	13	14	15	16	17	18	19	20	21	22	23	24	25	22	23	24	25	26	27	28	29	30	31	32	33	34	35	36	37	38	39	40	−5	−4	−3	−2	−1	0	1	2	3	4	5	6	7	8	9	10	11
Melanochromis chipokae							1*												1*																			1*																1*										
Melanochromis loriae							4*												4*																			4*																4*										
Melanochromis melanopterus							11												5	6																		5	6															5	6									
Melanochromis simulans							1												1																			1																1										
Melanochromis cf. *vermivorus*						1	1												1	1																		2																1		1								
Petrotilapia genalutea							1												1																			1																1										
Petrotilapia nigra						1														1																		1																		1								
Petrotilapia tridentiger							1												1																			1																1										
Petrotilapia sp.							4											1	3																		1	3															1	3										
Pseudotropheus elegans						1*														1*																		1*																		1*								
Pseudotropheus interruptus						1	1*												1*	1																		2*																1*	–	1								
Pseudotropheus johannii						9	24	4*										5	25*	7																	7	26	4*														2	5*	19	6	5								
Pseudotropheus livingstonii						6*	1												1	6*																	1	5*	1																2	5*								
Pseudotropheus lucerna							1													1																			1																1									
Tropheops tropheops							5	2										3	4																		1	6														2	1	4										
**Pseudotropheina column totals**	–	–	–	–	19	97	90	13	–	–	–	–	–	–	–	–	–	31	134	54	–	–	–	–	–	–	–	–	–	–	–	–	–	–	1	23	74	98	21	2	–	–	–	–	–	–	–	–	–	–	–	6	20	72	71	45	5	–	–	–	–	–	–	–
**Pseudocrenilabrini: Rhamphochromina**																																																																
Diplotaxodon argenteus								2*	4	4										4	6*																				6*	–	4										8	–	2*									
Diplotaxodon ecclesi								1*													1*																				1*														1*									
Diplotaxodon greenwoodi							1*														1*																			1*																1*								
Diplotaxodon limnothrissa									2	8										2	5	3																				2	7	1									2	5	1	2									
Pallidochromis tokolosh						1	3															4																		1	3																	3	1						
Rhamphochromis brevis								1	1*													1*	1																				2*													1*	–	1							
Rhamphochromis esox										2														1	1																					1	1									1	1							
Rhamphochromis woodi									1														1																					1													1								
**Rhamphochromina column totals**	–	–	–	–	–	1	4	4	8	14	–	–	–	–	–	–	–	–	–	6	13	8	2	1	1	–	–	–	–	–	–	–	–	–	–	–	–	–	–	2	10	2	13	2	–	1	1	–	–	–	–	2	13	1	6	3	5	1	–	–	–	–	–	–
**Cyrtocarina column totals**	–	–	–	1	74	191	124	35	–	–	–	–	–	–	–	–	–	2	48	199	136	35	5	–	–	–	–	–	–	–	–	–	–	–	–	3	52	171	102	60	25	12	–	–	–	–	–	–	–	–	–	–	3	33	86	160	83	52	7	1	–	–	–	–
**Pseudotropheina column totals**	–	–	–	–	19	97	90	13	–	–	–	–	–	–	–	–	–	31	134	54	–	–	–	–	–	–	–	–	–	–	–	–	–	–	1	23	74	98	21	2	–	–	–	–	–	–	–	–	–	–	–	6	20	72	71	45	5	–	–	–	–	–	–	–
**Rhamphochromina column totals**	–	–	–	–	–	1	4	4	8	14	–	–	–	–	–	–	–	–	–	6	13	8	2	1	1	–	–	–	–	–	–	–	–	–	–	–	–	–	–	2	10	2	13	2	–	1	1	–	–	–	–	2	13	1	6	3	5	1	–	–	–	–	–	–
