# Supplementary material for: African cichlid fishes: morphological data and taxonomic insights from a genus-level survey of supraneurals, pterygiophores, and vertebral counts (Ovalentaria, Blenniiformes, Cichlidae, Pseudocrenilabrinae)
Source: Biodivers Data J. 2024 Oct 18;12:e130707. doi: 10.3897/BDJ.12.e130707 (PMC11512106; doi:10.3897/BDJ.12.e130707)
Supplement: Supplementary material 3 — Table S2. [file bdj-12-e130707-s003.pdf]

Table 2. Frequency distribution of counts of vertebrae behind last occupied dorsal insertion space and interhemal space

	Vertebrae (including urostylar) posterior to those of last occupied DIS												Vertebrae (including urostylar) posterior to those of last occupied IHS												
	3	4	5	6	7	8	9	10	11	12	13	?	3	4	5	6	7	8	9	10	11	12	13	14	?
Polycentridae																									
Afronandus sheljuzhkoi				1														1							
Monocirrhus polyacanthus	1	1											2												
Polycentropsis abbreviata	1	1											2												
Cichlidae: Etroplinae																									
**India**																									
Etroplus suratensis			1												1										
Pseudetroplus maculatus		1	6											1	5	1									
**Madagascar**																									
Paretroplus polyactis				1												1									
Ptychochrominae																									
**Madagascar**																									
Katria katria									1											1					
Oxylapia polli										1											1				
Paratilapia polleni						9	2										4	7							
Ptychochromis oligacanthus					10	1											10	1							
Ptychochromoides betsileanus						2	1											2	–	1					
Cichlinae																									
**Neotropics**																									
**Astronotini**																									
Astronotus ocellatus			3													3									
**Chaetobranchini**																									
Chaetobranchopsis orbicularis		1											1												
Chaetobranchus flavescens			4												4										
**Cichlasomatini**																									
Aequidens tetramerus			1													1									
Andinoacara pulcher			1													1									
Bujurquina vittata			1													1									
Cichlasoma dimerus			1												1										
Cichlasoma taenia		1													1										
Krobia guianensis				1												1									
**Cichlini**																									
Cichla ocellaris									1												1				
Cichla orinocensis								1	2										1	1	1				
**Geophagini**																									
Biotodoma cupido					3	1											2	2							
Geophagus crassilabris					1													1							
Guianacara owroewefi				3	5												6	2							
Saxatilia frenata					1*													1*							
Saxatilia lepidota				2														2							
**Heroini**																									
Amphilophus citrinellus					1												1								
Hypselacara coryphaenoides			1	4												5									
**Retroculini**																									
Retroculus lapidifer							1														1				
Pseudocrenilabrinae																									
**Middle East**																									
**Oreochromini**																									
Iranocichla hormuzensis					2												2								
Tristramella sacra							1												1						
Tristramella simonis						4												4							
**Oreochromini (M.E.) column totals**	**–**	**–**	**–**	**–**	**2**	**4**	**1**	**–**	**–**	**–**	**–**	**–**	**–**	**–**	**–**	**–**	**2**	**4**	**1**	**–**	**–**	**–**	**–**	**–**	**–**
**Pseudocrenilabrini**																									
Astatotilapia flaviijosephi					2	2											2	2							
**African riverine**																									
**Chromidotilapiini**																									
Benitochromis batesii				6												2	4								
Benitochromis finleyi				1													1								
Chromidotilapia guntheri			1	1												2									

	Vertebrae (including urostylar) posterior to those of last occupied DIS												Vertebrae (including urostylar) posterior to those of last occupied IHS												
	3	4	5	6	7	8	9	10	11	12	13	?	3	4	5	6	7	8	9	10	11	12	13	14	?
Chromidotilapia kingsleyae			1	7	1											2	7								
Congochromis robustus			1*														1*								
Congochromis squamiceps			1	1												1	1								
Divandu albimarginatus			1	3												2	2								
Limbochromis robertsi				17												2	15								
Nanochromis nudiceps			2														2								
Nanochromis parilus		2													1	1									
Parananochromis gabonicus					1*													1*							
Parananochromis longirostris			1	10												4	7								
Pelmatochromis buettikoferi				3												1	2								
Pelmatochromis ocellifer				1*												1*									
Pelvicachromis pulcher				1												1									
Pelvicachromis taeniatus				1*												1*									
Pterochromis congicus					2*												2*								
Teleogramma brichardi			3	1															2	2					
Thysochromis ansorgii				4												3	1								
Wallaceochromis humilis				4*	1												2*	3							
**Chromidotilapiini column totals**	**–**	**2**	**11**	**61**	**5**	**–**	**–**	**–**	**–**	**–**	**–**	**–**	**–**	**–**	**1**	**23**	**47**	**4**	**2**	**2**	**–**	**–**	**–**	**–**	**–**
**Coelotilapiini**																									
Coelotilapia joka				1												1									
**Coptodonini**																									
Coptodon discolor				2												2									
Coptodon tholloni				1													1								
Coptodon zillii			1	10	13										1	9	13	1							
**Coptodonini column totals**	**–**	**–**	**1**	**13**	**13**	**–**	**–**	**–**	**–**	**–**	**–**	**–**	**–**	**–**	**1**	**11**	**14**	**1**	**–**	**–**	**–**	**–**	**–**	**–**	**–**
**Etiini**																									
Etia nguti				6												6									
**Gobiocichlini**																									
Gobiocichla ethelwynnae					9	2												10	1						
Gobiocichla wonderi			1	10	3													2	10	2					
Paragobiocichla irvinei				2	2													4							
**Gobiocichlini colulmn totals**	**–**	**–**	**1**	**12**	**14**	**2**	**–**	**–**	**–**	**–**	**–**	**–**	**–**	**–**	**–**	**–**	**–**	**16**	**11**	**2**	**–**	**–**	**–**	**–**	**–**
**Hemichromini**																									
Anomalochromis thomasi				3	1											3	1								
Hemichromis elongatus					4												3	1							
Hemichromis fasciatus					1												1								
Rubricatochromis bimaculatus				6												4	2								
Rubricatochromis letourneuxi				2	1	1										2	1	1							
**Hemichromini column totals**	**–**	**–**	**–**	**11**	**7**	**1**	**–**	**–**	**–**	**–**	**–**	**–**	**–**	**–**	**–**	**9**	**8**	**2**	**–**	**–**	**–**	**–**	**–**	**–**	**–**
**Heterochromini**																									
Heterochromis multidens			1	10												1	10								
**Heterotilapiini**																									
Heterotilapia buettikoferi			3	2												5									
**Oreochromini**																									
Danakilia franchettii				2	12												8	6							
Oreochromis jipe				1	2	1											1	2	1						
Oreochromis niloticus				1	1	1											2	1							
Oreochromis shiranus					6												3	3							
Sarotherodon galilaeus				2												1	1								
Sarotherodon melanotheron				5	1											3	3								
**Oreochromini (riverine) totals**	**–**	**–**	**–**	**11**	**22**	**2**	**–**	**–**	**–**	**–**	**–**	**–**	**–**	**–**	**–**	**4**	**18**	**12**	**1**	**–**	**–**	**–**	**–**	**–**	**–**
**Pelmatolapiini**																									
Pelmatolapia mariae				4											1	5									
**Pseudocrenilabrini**																									
Astatotilapia bloyeti						8												6	2						
Astatotilapia burtoni				1	1	3	1										2	4							
Astatotilapia calliptera					2	7*											1	5	3*						
Astatotilapia swynnertoni					2	1											2	1							
Chetia flaviventris						1													1						
Chetia gracilis						1*													1*						
Chetia ? sp.			2	4	2											2	4	–	–	–	–	–	–	–	2

	Vertebrae (including urostylar) posterior to those of last occupied DIS												Vertebrae (including urostylar) posterior to those of last occupied IHS												
	3	4	5	6	7	8	9	10	11	12	13	?	3	4	5	6	7	8	9	10	11	12	13	14	?
Ctenochromis pectoralis					2													2							
Ctenochromis scatebra						1*													1*						
Haplochromis demeusii					1*													1*							
Haplochromis fasciatus					5	1												6							
Haplochromis humilis						3												2	1						
Haplochromis moeruensis						4												1	3						
Haplochromis oligacanthus					1*												1*								
Lufubuchromis relictus						1*													1*						
Orthochromis machadoi					1	1												1	–	1					
Orthochromis malagaraziensis					2												1	–	1						
Orthochromis polyacanthus				1	–	1												1	–	1					
Orthochromis stormsi			1	4	6												1	8	2						
Palaeoplex palimpsest						1*													1*						
Pharyngochromis acuticeps						3	3											1	3	2					
Pseudocrenilabrus multicolor			1	3												2	2								
Pseudocrenilabrus philander					1													1							
Sargochromis carlottae					1	1												1	1						
Sargochromis codringtonii					1	1	1*											1	2*						
Sargochromis giardi						2												1	1						
Sargochromis greenwoodi						2*													2*						
Serranochromis angusticeps								1												1					
Serranochromis longimanus							4*												3*	1					
Serranochromis macrocephalus						1													1						
Serranochromis meridianus							1												1						
Serranochromis robustus							4	2												6					
Thoracochromis albolabris								1												1					
Thoracochromis wingatii					1	1												2							
**Pseudocrenilabrini (riverine) totals**	**–**	**–**	**4**	**13**	**29**	**45**	**14**	**4**	**–**	**–**	**–**	**–**	**–**	**–**	**–**	**4**	**14**	**45**	**31**	**13**	**–**	**–**	**–**	**–**	**2**
**Steatocranini**																									
Steatocranus casuarius			1	2													1	2							
**Tilapiini**																									
Chilochromis duponti				1													1								
Congolapia bilineata					3												3								
Tilapia busumana			1	2	–	1										3	1								
Tilapia sparrmanii				4*												3*	1								
**Tilapiini column totals**	**–**	**–**	**1**	**7**	**3**	**1**	**–**	**–**	**–**	**–**	**–**	**–**	**–**	**–**	**–**	**6**	**6**	**–**	**–**	**–**	**–**	**–**	**–**	**–**	**–**
**Lake Barombi Mbo**																									
**Oreochromini**																									
Konia eisentrauti				1	4												1	4							
Myaka myaka					1	3												4							
Pungu maclareni					2*												2*								
Stomatepia mariae						1												1							
Stomatepia pindu					2	2											2	2							
**Oreochromini (Barombi) totals**	**–**	**–**	**–**	**1**	**9**	**6**	**–**	**–**	**–**	**–**	**–**	**–**	**–**	**–**	**–**	**–**	**5**	**11**	**–**	**–**	**–**	**–**	**–**	**–**	**–**
**Lake Fwa**																									
**Pseudocrenilabrini**																									
Cyclopharynx schwetzi				1	9	4											1	12	1						
Schwetzochromis neodon						2												1	1						
**Lake Albert**																									
**Pseudocrenilabrini**																									
Haplochromis avium						1	2												2	1					
Haplochromis loati						1												1							
**Lake Edward–George system**																									
**Pseudocrenilabrini**																									
Haplochromis akika						2	2*											1	3*						
Haplochromis aquila								1*												1*					
Haplochromis aureus								1*												1*					
Haplochromis curvidens							1*												1*						
Haplochromis falcatus							1*												1*						
Haplochromis fuscus						1												1							
Haplochromis glaucus							1*												1*						

	Vertebrae (including urostylar) posterior to those of last occupied DIS												Vertebrae (including urostylar) posterior to those of last occupied IHS												
	3	4	5	6	7	8	9	10	11	12	13	?	3	4	5	6	7	8	9	10	11	12	13	14	?
Haplochromis gracilifur							1*												1*						
Haplochromis kimondo							1*												1*						
Haplochromis labiatus						1												1							
Haplochromis latifrons								1*												1*					
Haplochromis limax						4											1	3							
Haplochromis mentatus							1*												1*						
Haplochromis molossus							1*												1*						
Haplochromis pappenheimi								1*												1*					
Haplochromis paradoxus						1*													1*						
Haplochromis pardus						1*													1*						
Haplochromis pelagicus						1*														1*					
Haplochromis pharyngalis							1*												1*						
Haplochromis quasimodo							1*													1*					
Haplochromis relictidens							1*												1*						
Haplochromis rex						1*													1*						
Haplochromis simba								1*												1*					
Haplochromis squamipinnis							1*												1*						
Haplochromis taurinus							1*												1*						
Schubotzia eduardiana						4	6											1	9						
**Pseudocrenilabrini (Ed–Grg) totals**	**–**	**–**	**–**	**–**	**–**	**16**	**20**	**5**	**–**	**–**	**–**	**–**	**–**	**–**	**–**	**–**	**1**	**7**	**26**	**7**	**–**	**–**	**–**	**–**	**–**
**Lake Victoria & satellites**																									
**Pseudocrenilabrini**																									
Allochromis welcommei							1												1						
Astatoreochromis alluaudi							1												1						
Haplochromis chlorochrous							1	2	1										1	1	2				
Haplochromis cryptogramma								6	4											4	5	1			
Haplochromis fusiformis								1													1				
Haplochromis latifasciatus						3	3											1	5						
Haplochromis lividus						1	1												2						
Haplochromis nubilus						3*												1	2*						
Haplochromis plutonius							1	3	1											3	2				
Lithochromis rubripinnis						2	2											2	2						
Lithochromis xanthopteryx						1	3											1	2	1					
Mbipia mbipi						1												1							
Neochromis nigricans					1													1							
Paralabidochromis victoriae							2												2						
Pundamilia igneopinnis							1	1											1	1					
Pundamilia pundamilia						3	1											3	1						
Pyxichromis parorthostoma						1											1								
**Pseudocrenilabrini (Victoria) totals**	**–**	**–**	**–**	**–**	**1**	**15**	**17**	**13**	**6**	**–**	**–**	**–**	**–**	**–**	**–**	**–**	**1**	**10**	**20**	**10**	**10**	**1**	**–**	**–**	**–**
**Lake Kivu**																									
**Pseudocrenilabrini**																									
Haplochromis astatodon						2	4*											2*	4						
Haplochromis paucidens					1	1												1	1						
**Lake Turkana**																									
**Pseudocrenilabrini**																									
Haplochromis rudolfianus						2	6											2	6						
Haplochromis turkanae						1	2											1	2						
**Lake Tanganyika**																									
**Bathybatini**																									
Bathybates fasciatus											5											5			
Bathybates ferox								2	8											10					
Bathybates graueri									4	1										4	1				
Bathybates hornii									1												1				
Bathybates leo									4	1										4	1				
Bathybates minor											5									1	4				
Bathybates vittatus										1											1				
Hemibates stenosoma									4												4				
Trematocara unimaculatum							2	3											1	4					
Trematocara zebra								2	3												1	4			
**Bathybatini column totals**	**–**	**–**	**–**	**–**	**–**	**–**	**2**	**7**	**24**	**3**	**10**	**–**	**–**	**–**	**–**	**–**	**–**	**–**	**1**	**23**	**13**	**9**	**–**	**–**	**–**

	Vertebrae (including urostylar) posterior to those of last occupied DIS												Vertebrae (including urostylar) posterior to those of last occupied IHS												
	3	4	5	6	7	8	9	10	11	12	13	?	3	4	5	6	7	8	9	10	11	12	13	14	?
**Benthochromini**																									
Benthochromis tricoti							4	1											1	4					
**Boulengerochromini**																									
Boulengerochromis microlepis								7												1	6				
**Cyphotilapiini**																									
Cyphotilapia frontosa					1	4													5						
Cyphotilapia gibberosa					2	3												3	2						
**Cyprichromini**																									
Cyprichromis coloratus									4	1													5		
Cyprichromis leptosoma									5													5			
Cyprichromis microlepidotus								2	3												3	2			
Cyprichromis pavo									5													3	2		
Cyprichromis zonatus									5													3	2		
Cyprichromis sp. "dwarf jumbo"								3	1	1												3	2		
Cyprichromis sp. "jumbo"									5														5		
Paracyprichromis brieni								1	4													3	2		
Paracyprichromis nigripinnis								2	2												1	2	1		
Paracyprichromis sp. "brieni south"								1	4												1	4			
**Cyprichromini column totals**	**–**	**–**	**–**	**–**	**–**	**–**	**–**	**9**	**38**	**2**	**–**	**–**	**–**	**–**	**–**	**–**	**–**	**–**	**–**	**–**	**5**	**25**	**19**	**–**	**–**
**Ectodini**																									
Asprotilapia leptura							1	3	1														1	4	
Aulonocranus dewindti						1	4													4	1				
Callochromis macrops					1	4														1	4				
Cardiopharynx schoutedeni							5													3	2				
Cunningtonia longiventralis							1	4												1	4				
Cyathopharynx furcifer						1	4												2	3					
Ectodus descampsii						3	2												1	4					
Enantiopus melanogenys						1	1	3											2	3					
Grammatotria lemairii								3	2													4	1		
Lestradea perspicax							4	2													5	1			
Microdontochromis tenuidentatus							1	3	1												1	4			
Ophthalmotilapia boops							2	3													5				
Xenotilapia sima							5														4	1			
**Ectodini column totals**	**–**	**–**	**–**	**–**	**1**	**10**	**30**	**21**	**4**	**–**	**–**	**–**	**–**	**–**	**–**	**–**	**–**	**–**	**5**	**19**	**26**	**10**	**2**	**4**	**–**
**Eretmodini**																									
Eretmodus cyanostictus		2	3															5							
Eretmodus marksmithi			5																5						
Spathodus erythrodon			5																5						
Tanganicodus irsacae		3	2																5						
**Eretmodini column totals**	**–**	**5**	**15**	**–**	**–**	**–**	**–**	**–**	**–**	**–**	**–**	**–**	**–**	**–**	**–**	**–**	**–**	**5**	**15**	**–**	**–**	**–**	**–**	**–**	**–**
**Lamprologini**																									
Altolamprologus calvus				4	1											1	4								
Altolamprologus compressiceps				1	4												3	2							
Altolamp. 'compressiceps shell'				1	4												3	2							
Chalinochromis brichardi					5													4	1						
Chalinochromis cyanophleps						1	4												1	4					
Julidochromis ornatus				3	2													5							
Julidochromis regani					4	1												1	4						
Lamprologus callipterus						4	6												6	4					
Lamprologus 'ornatipinnis congo'							4	1												5					
Lamprologus 'ornatipinnis zambia'						1	4	4												7	1	1			
Lamprologus speciosus				5													5								
Lepidiolamprologus kendalli						3	2												3	2					
Lepidiolamprologus mimicus							4	1												3	2				
Lepidiolamprologus profundicola							1	3	1												5				
Neolamprologus tretocephalus						5													2	3					
Neolamprologus ventralis						4	1												1	4					
Telmatochromis dhonti					5													4	1						
Telmatochromis temporalis			1	–	3	1										1	–	3	1						
Telmatochromis vittatus						4	1												3	2					
Variabilichromis moorii					5	2											3	3	1						

	Vertebrae (including urostylar) posterior to those of last occupied DIS												Vertebrae (including urostylar) posterior to those of last occupied IHS												
	3	4	5	6	7	8	9	10	11	12	13	?	3	4	5	6	7	8	9	10	11	12	13	14	?
**Lamprologini column totals**	**–**	**–**	**1**	**14**	**33**	**26**	**27**	**9**	**1**	**–**	**–**	**–**	**–**	**–**	**–**	**2**	**18**	**24**	**24**	**34**	**8**	**1**	**–**	**–**	**–**
**Limnochromini**																									
Baileychromis centropomoides							5													4	1				
Gnathochromis permaxillaris							5													5					
Greenwoodochromis christyi						1	4												2	3					
Limnochromis auritus							3	2												1	4				
Reganochromis calliurus						1	4														5				
Tangachromis dhanisi						1	3												1	2	1				
Trematochromis benthicola						3	2												3	2					
Triglachromis otostigma							4	1											5						
**Limnochromini column totals**	**–**	**–**	**–**	**–**	**–**	**6**	**30**	**3**	**–**	**–**	**–**	**–**	**–**	**–**	**–**	**–**	**–**	**–**	**11**	**17**	**11**	**–**	**–**	**–**	**–**
**Oreochromini**																									
Oreochromis tanganicae					5												1	4							
**Perissodini**																									
Haplotaxodon microlepis							1	3	1												3	2			
Perissodus microlepis						1	4												1	3	1				
Plecodus paradoxus						1	4												1	4					
Xenochromis hecqui						1	4												5						
**Perissodonini column totals**	**–**	**–**	**–**	**–**	**–**	**3**	**13**	**3**	**1**	**–**	**–**	**–**	**–**	**–**	**–**	**–**	**–**	**–**	**7**	**7**	**4**	**2**	**–**	**–**	**–**
**Pseudocrenilabrini: Tropheina**																									
Interochromis loocki					1	4												1	4						
Jabarichromis pfefferi					3	2												1	4						
Limnotilapia dardennii							4													4					
Lobochilotes labiata						5													2	3					
Petrochromis horii					1	4												2	3						
Petrochromis polyodon						5													5						
Pseudosimochromis curvifrons					3	2												5							
Shuja horei					2	6												1	7						
Simochromis diagramma						5												2	2	1					
Tropheus annectens				4	1												3	2							
Tropheus brichardi				5													5								
Tropheus duboisi				4	1												4	1							
Tropheus moorii			1	5	4											1	5	4							
**Tropheina column totals**	**–**	**–**	**1**	**18**	**16**	**33**	**4**	**–**	**–**	**–**	**–**	**–**	**–**	**–**	**–**	**1**	**17**	**19**	**27**	**8**	**–**	**–**	**–**	**–**	**–**
**Tylochromini**																									
Tylochromis polylepis					3	3													3	3					
**Lake Malawi**																									
**Pseudocrenilabrini: Cyrtocarina**																									
Alticorpus mentale									1													1			
Alticorpus peterdaviesi								1													1				
Aristochromis christyi							1	1												1	1				
Aulonocara nyassae							1													1					
Aulonocara rostratum								1													1				
Aulonocara stonemani							1*														1*				
Buccochromis atritaeniatus								2*												2*					
Buccochromis heterotaenia							2*													2*					
Buccochromis nototaenia						1*	–	1												2*					
Buccochromis oculatus								3													3				
Buccochromis spectabilis							1														1				
Caprichromis orthognathus						3	2												3	2					
Champsochromis caeruleus							1	3													4				
Champsochromis spilorhynchus							2													2					
Cheilochromis euchilus						3*	2											1	2*	2					
Chilotilapia rhoadesii						2	2												4						
Copadichromis jacksoni						3													3						
Copadichromis quadrimaculatus						1												1							
Corematodus taeniatus						1	6												2	5					
Ctenopharynx nitidus						10	6												5	11					
Ctenopharynx pictus						1													1						
Cyrtocara moorii						3	3*												4	2*					
Dimidiochromis compressiceps							7	5											2	10					

	Vertebrae (including urostylar) posterior to those of last occupied DIS												Vertebrae (including urostylar) posterior to those of last occupied IHS												
	3	4	5	6	7	8	9	10	11	12	13	?	3	4	5	6	7	8	9	10	11	12	13	14	?
Dimidiochromis kiwinge							3													3					
Docimodus evelynae						1	1*												1	1*					
Docimodus johnstoni							2	1												2	1				
Exochochromis anagenys							7*													6	1*				
Fossorochromis rostratus							1												1						
Hemitaeniochromis urotaenia							1												1						
Hemitilapia oxyrhynchus						7	3											2	7	1					
Lethrinops gossei							1*	1												2*					
Lethrinops lethrinus						2	1												2	1					
Lethrinops polli							1*													1*					
Lichnochromis acuticeps							1												1						
Mchenga cyclicos								1													1				
Mchenga inornata							2*													2*					
Mchenga sp.						2	14	7										1	4	14	4				
Mylochromis formosus						1	1*												1	1*					
Mylochromis gracilis							1*	–	2											2*	1				
Mylochromis guentheri						2													1	1					
Mylochromis lateristriga						3	1												4						
Mylochromis spilostichus						1	5*	2												5	3*				
Mylochromis subocularis							2												1	1					
Naevochromis chrysogaster						2	3*											1	2	2*					
Nimbochromis fuscotaeniatus						1	1												1	1					
Nimbochromis livingstonii						1	5												2	4					
Nimbochromis venustus						2	2											1	3						
Nyassachromis leuciscus						1*	5												1*	5					
Nyassachromis microcephalus						1*	1												1*	1					
Nyassachromis nigritaeniatus							2	2											1	3					
Nyassachromis purpurans							1													1					
Otopharynx argyrosoma							2*	1											1*	2					
Otopharynx decorus							3	3*												5*	1				
Otopharynx cf. *heterodon*						16	3											2	16	1					
Otopharynx heterodon					2	5	1											3	5						
Otopharynx lithobates					1	4*												1	4*						
Otopharynx ovatus						4*	1												3*	2					
Otopharynx selenurus							3	3											1	4	1				
Otopharynx speciosus						2	1	2										1	1	2	1				
Otopharynx tetraspilus						7	1											5	3						
Otopharynx tetrastigma					1	12*	3											2	13*	1					
Placidochromis hennydaviesae							1*													1*					
Placidochromis johnstoni						1	3												3	1					
Placidochromis longimanus						1	22	5											4	22	2				
Placidochromis macrognathus							2													2					
Placidochromis milomo					1	3												4							
Protomelas annectens							1	1												2					
Protomelas fenestratus					5	19	12											10	25	1					
Protomelas insignis						2													2						
Protomelas kirkii						3*													3*						
Protomelas labridens						1												1							
Protomelas ornatus						2												1	1						
Protomelas spilopterus					1	4	1												5	1					
Protomelas taeniolatus						1													1						
Protomelas triaenodon						2	1												2	1					
Sciaenochromis ahli						1	15												3	12	1				
Stigmatochromis modestus					1*	4												2*	3						
Stigmatochromis pholidophorus						2*	2	1											3*	2					
Stigmatochromis pleurospilus												1*													1*
Stigmatochromis woodi						1	4*	–	–	–	–	2						1	3*	2	–	–	–	–	1
Taeniochromis holotaenia				1													1								
Taeniolethrinops praeorbitalis								1													1				
Tramitichromis brevis							1											1							
Trematocranus labifer							4												2	2					

Table 2 (continued). Frequency distribution of counts of vertebrae behind last occupied dorsal insertion space and interhemal space

	Vertebrae (including urostylar) posterior to those of last occupied DIS												Vertebrae (including urostylar) posterior to those of last occupied IHS												
	3	4	5	6	7	8	9	10	11	12	13	?	3	4	5	6	7	8	9	10	11	12	13	14	?
Trematocranus microstoma						2	7												6	3					
Trematocranus placodon						2	1											1	1	1					
Tyrannochromis macrostoma						1														1					
Tyrannochromis nigriventer							1*													1*					
**Cyrtocarina column totals**	**–**	**–**	**–**	**1**	**12**	**157**	**201**	**48**	**3**	**–**	**–**	**3**	**–**	**–**	**–**	**–**	**1**	**42**	**175**	**174**	**30**	**1**	**–**	**–**	**2**
**Pseudocrenilabrini: Pseudotropheina**																									
Abactochromis labrosus					3	3*												4*	2						
Chindongo bellicosus					2	4												3	3						
Chindongo minutus					1												1								
Cyathochromis obliquidens						4												1	3						
Cynotilapia afra						1													1						
Cynotilapia sp.					3	1											1	3							
Genyochromis mento					2	8												1	9						
Gephyrochromis lawsi					1													1							
Gephyrochromis moorii					1	3												1	3						
Iodotropheus sprengerae					4	2											2	3	1						
Labeotropheus fuelleborni					6	8	1											6	9						
Labeotropheus trewavasae						2	1												2	1					
Labidochromis caeruleus					1													1							
Labidochromis freibergi						1*												1*							
Labidochromis joanjohnsonae						1*													1*						
Labidochromis pallidus					12	11												13	10						
Labidochromis shiranus					1														1						
Labidochromis textilis						2*													2*						
Labidochromis vellicans					22	7	–	–	–	–	–	1					2	21	7						
Maylandia aurora						1*													1*						
Maylandia koningsi						1												1*							
Maylandia lanisticola					1*													1*							
Maylandia zebra					5	4													9						
Melanochromis auratus					2	4*												1	4	1*					
Melanochromis chipokae						1*												1*							
Melanochromis loriae					2*	2												2*	2						
Melanochromis melanopterus					2	8	1											4	7						
Melanochromis simulans						1												1							
Melanochromis cf. *vermivorus*						2													2						
Petrotilapia genalutea					1													1							
Petrotilapia nigra						1													1						
Petrotilapia tridentiger						1													1						
Petrotilapia sp.					2	2												4							
Pseudotropheus elegans					1*														1*						
Pseudotropheus interruptus					1*	1													2*						
Pseudotropheus johannii				2	25	10*											3	25	9*						
Pseudotropheus livingstonii						6*	1												7*						
Pseudotropheus lucerna							1													1					
Tropheops tropheops				1	4	2											2	5							
**Pseudotropheina column totals**	**–**	**–**	**–**	**3**	**105**	**105**	**5**	**–**	**–**	**–**	**–**	**1**	**–**	**–**	**–**	**–**	**11**	**105**	**100**	**3**	**–**	**–**	**–**	**–**	**–**
**Pseudocrenilabrini: Rhamphochromina**																									
Diplotaxodon argenteus								1	6*	3										2	7*	1			
Diplotaxodon ecclesi									1*												1*				
Diplotaxodon greenwoodi									1*												1*				
Diplotaxodon limnothrissa								2	4	4										1	5	4			
Pallidochromis tokolosh									1	2	1										1	3			
Rhamphochromis brevis								1*	1												2*				
Rhamphochromis esox										2													2		
Rhamphochromis woodi										1													1		
**Rhamphochromina column totals**	**–**	**–**	**–**	**–**	**–**	**–**	**–**	**4**	**14**	**12**	**1**	**–**	**–**	**–**	**–**	**–**	**–**	**–**	**–**	**3**	**17**	**8**	**3**	**–**	**–**
**Cyrtocarina column totals**	**–**	**–**	**–**	**1**	**12**	**157**	**201**	**48**	**3**	**–**	**–**	**3**	**–**	**–**	**–**	**–**	**1**	**42**	**175**	**174**	**30**	**1**	**–**	**–**	**2**
**Pseudotropheina column totals**	**–**	**–**	**–**	**3**	**105**	**105**	**5**	**–**	**–**	**–**	**–**	**1**	**–**	**–**	**–**	**–**	**11**	**105**	**100**	**3**	**–**	**–**	**–**	**–**	**–**
**Rhamphochromina column totals**	**–**	**–**	**–**	**–**	**–**	**–**	**–**	**4**	**14**	**12**	**1**	**–**	**–**	**–**	**–**	**–**	**–**	**–**	**–**	**3**	**17**	**8**	**3**	**–**	**–**
