# Supplementary material for: African cichlid fishes: morphological data and taxonomic insights from a genus-level survey of supraneurals, pterygiophores, and vertebral counts (Ovalentaria, Blenniiformes, Cichlidae, Pseudocrenilabrinae)
Source: Biodivers Data J. 2024 Oct 18;12:e130707. doi: 10.3897/BDJ.12.e130707 (PMC11512106; doi:10.3897/BDJ.12.e130707)
Supplement: Supplementary material 4 — Table S3. [file bdj-12-e130707-s004.pdf]

Table 4. Frequency distributions of counts of anal pterygiophores anterior to HSp1, and of total anal pterygiophores

	Anal pterygiophores anterior to HSp1									Total number of anal pterygiophores																		
	0	1	2	3	4	5	6	7	8	6	7	8	9	10	11	12	13	14	15	16	17	18	19	20	21	22	24	?
Polycentridae																												
Afronandus sheljuzhkoi			1										1															
Monocirrhus polyacanthus				2																						1	1	
Polycentropsis abbreviata		1	1																	1	1							
Cichlidae: Etroplinae																												
**India**																												
Etroplus suratensis						1																				1		
Pseudetroplus maculatus					2	2	2	–	1													2	3	2				
**Madagascar**																												
Paretroplus polyactis			1																						1			
Ptychochrominae																												
**Madagascar**																												
Katria katria			1										1															
Oxylapia polli			1										1															
Paratilapia polleni		10	1											1	8	2												
Ptychochromis oligacanthus		11											8	3														
Ptychochromoides betsileanus		2	1											2	1													
Cichlinae																												
**Neotropics**																												
**Astronotini**																												
Astronotus ocellatus		3																		3								
**Chaetobranchini**																												
Chaetobranchopsis orbicularis			1																						1			
Chaetobranchus flavescens		4														4												
**Cichlasomatini**																												
Aequidens tetramerus		1										1																
Andinoacara pulcher		1											1															
Bujurquina vittata		1										1																
Cichlasoma dimerus		1												1														
Cichlasoma taenia		1												1														
Krobia guianensis		1											1															
**Cichlini**																												
Cichla ocellaris			1													1												
Cichla orinocensis		2	1													2	1											
**Geophagini**																												
Biotodoma cupido		3	1										2	2														
Geophagus crassilabris		1										1																
Guianacara owroewefi		8											8															
Saxatilia frenata		1*												1*														
Saxatilia lepidota		1												1	1													
**Heroini**																												
Amphilophus citrinellus			1															1										
Hypselacara coryphaenoides	1	–	4														5											
**Retroculini**																												
Retroculus lapidifer		1										1																
Pseudocrenilabrinae																												
**Middle East**																												
**Oreochromini**																												
Iranocichla hormuzensis		2											2															
Tristramella sacra		1												1														
Tristramella simonis		4											4															
**Oreochromini (M.E.) column totals**	–	**7**	–	–	–	–	–	–	–	–	–	–	**6**	**1**	–	–	–	–	–	–	–	–	–	–	–	–	–	–
**Pseudocrenilabrini**																												
Astatotilapia flavijosephi		4											2	2														
**African riverine**																												
**Chromidotilapiini**																												
Benitochromis batesii	1	5									1	4	1															
Benitochromis finleyi		1										1																
Chromidotilapia guntheri	1	1										2																
Chromidotilapia kingsleyae		9										6	2	1														
Congochromis robustus		1*								1*																		
Congochromis squamiceps		2										2																
Divandu albimarginatus		4										1	2	1														
Limbochromis robertsi	1	13	3									1	14	2														
Nanochromis nudiceps		2									1	–	1															
Nanochromis parilus		2											2															
Parananochromis gabonicus		1*										1*																
Parananochromis longirostris		9	2									11																

	Anal pterygiophores anterior to HSp1									Total number of anal pterygiophores																		
	0	1	2	3	4	5	6	7	8	6	7	8	9	10	11	12	13	14	15	16	17	18	19	20	21	22	24	?
Pelmatochromis buettikoferi		3											3															
Pelmatochromis ocellifer		1*											1*															
Pelvicachromis pulcher		1										1																
Pelvicachromis taeniatus		1*										1*																
Pterochromis congicus		2											1	1														
Teleogramma brichardi		4													2	2												
Thysochromis ansorgii		4											3	1														
Wallaceochromis humilis	1*	4										5*																
**Chromidotilapiini column totals**	**4**	**70**	**5**	**–**	**–**	**–**	**–**	**–**	**–**	**1**	**2**	**36**	**30**	**6**	**2**	**2**	**–**	**–**	**–**	**–**	**–**	**–**	**–**	**–**	**–**	**–**	**–**	**–**
**Coelotilapiini**																												
Coelotilapia joka		1											1															
**Coptodonini**																												
Coptodon discolor		2												2														
Coptodon tholloni		1												1														
Coptodon zillii		23	1									2	7	8	6	1												
**Coptodonini column totals**	**–**	**26**	**1**	**–**	**–**	**–**	**–**	**–**	**–**	**–**	**–**	**2**	**7**	**11**	**6**	**1**	**–**	**–**	**–**	**–**	**–**	**–**	**–**	**–**	**–**	**–**	**–**	**–**
**Etiini**																												
Etia nguti		5	1										6															
**Gobiocichlini**																												
Gobiocichla ethelwynnae	2	7	2												3	7	1											
Gobiocichla wonderi		14										1	3	9	1													
Paragobiocichla irvinei		4										4																
**Gobiocichlini colulmn totals**	**2**	**25**	**2**	**–**	**–**	**–**	**–**	**–**	**–**	**–**	**–**	**5**	**3**	**9**	**4**	**7**	**1**	**–**	**–**	**–**	**–**	**–**	**–**	**–**	**–**	**–**	**–**	**–**
**Hemichromini**																												
Anomalochromis thomasi		4											4															
Hemichromis elongatus		4												4														
Hemichromis fasciatus			1											1														
Rubricatochromis bimaculatus		9										2	6	1														
Rubricatochromis letourneuxi		3	1									1	2	1														
**Hemichromini column totals**	**–**	**20**	**2**	**–**	**–**	**–**	**–**	**–**	**–**	**–**	**–**	**3**	**12**	**7**	**–**	**–**	**–**	**–**	**–**	**–**	**–**	**–**	**–**	**–**	**–**	**–**	**–**	**–**
**Heterochromini**																												
Heterochromis multidens		10	1											10	1													
**Heterotilapiini**																												
Heterotilapia buettikoferi		4	1												3	2												
**Oreochromini**																												
Danakilia franchettii		12	2								2	9	3															
Oreochromis jipe		2	2													3	1											
Oreochromis niloticus		3												1	2													
Oreochromis shiranus		5	1												2	4												
Sarotherodon galilaeus			2											2														
Sarotherodon melanotheron		6											4	2														
**Oreochromini (riverine) totals**	**–**	**28**	**7**	**–**	**–**	**–**	**–**	**–**	**–**	**–**	**2**	**9**	**7**	**5**	**4**	**7**	**1**	**–**	**–**	**–**	**–**	**–**	**–**	**–**	**–**	**–**	**–**	**–**
**Pelmatolapiini**																												
Pelmatolapia mariae		2	4												4	2												
**Pseudocrenilabrini**																												
Astatotilapia bloyeti		5	3										1	5	2													
Astatotilapia burtoni		6												4	2													
Astatotilapia calliptera		6*	3										7*	2														
Astatotilapia swynnertoni		2	1											2	1													
Chetia flaviventris		1												1														
Chetia gracilis		1*													1*													
Chetia ? sp.		8											4	2	–	–	–	–	–	–	–	–	–	–	–	–	–	2
Ctenochromis pectoralis		2												2														
Ctenochromis scatebra			1*										1*															
Haplochromis demeusii		1*										1*																
Haplochromis fasciatus		6										6																
Haplochromis humilis		2	1										1	2														
Haplochromis moeruensis		4										1	3															
Haplochromis oligacanthus		1*									1*																	
Lufubuchromis relictus			1*										1*															
Orthochromis machadoi		2											2															
Orthochromis malagaraziensis		1	1											1	1													
Orthochromis polyacanthus		2										2																
Orthochromis stormsi		11										9	2															
Palaeoplex palimpsest		1*												1*														
Pharyngochromis acuticeps		5	1									1	5															
Pseudocrenilabrus multicolor		3	1										2	2														
Pseudocrenilabrus philander		1											1															
Sargochromis carlottae			2												2													
Sargochromis codringtonii		3*												1	2*													

	Anal pterygiophores anterior to HSp1									Total number of anal pterygiophores																		
	0	1	2	3	4	5	6	7	8	6	7	8	9	10	11	12	13	14	15	16	17	18	19	20	21	22	24	?
Sargochromis giardi		1	–	1										1	1													
Sargochromis greenwoodi		2*											1	1*														
Serranochromis angusticeps		1														1												
Serranochromis longimanus		4*													4*													
Serranochromis macrocephalus		1												1														
Serranochromis meridianus		1													1													
Serranochromis robustus		6													5	1												
Thoracochromis albolabris		1											1															
Thoracochromis wingatii		1	1										2															
**Pseudocrenilabrini (riverine) totals**	**–**	**92**	**16**	**1**	**–**	**–**	**–**	**–**	**–**	**–**	**1**	**20**	**34**	**28**	**22**	**2**	**–**	**–**	**–**	**–**	**–**	**–**	**–**	**–**	**–**	**–**	**–**	**2**
**Steatocranini**																												
Steatocranus casuarius		3									2	1																
**Tilapiini**																												
Chilochromis duponti		1											1															
Congolapia bilineata		3										2	1															
Tilapia busumana		4										2	2															
Tilapia sparrmanii		3*	1											3	1*													
**Tilapiini column totals**	**–**	**11**	**1**	**–**	**–**	**–**	**–**	**–**	**–**	**–**	**–**	**4**	**4**	**3**	**1**	**–**	**–**	**–**	**–**	**–**	**–**	**–**	**–**	**–**	**–**	**–**	**–**	**–**
**Lake Barombi Mbo**																												
**Oreochromini**																												
Konia eisentrauti		5											3	2														
Myaka myaka		3	1											3	1													
Pungu maclareni		2*												2*														
Stomatepia mariae		1												1														
Stomatepia pindu		4												3	1													
**Oreochromini (Barombi) totals**	**–**	**15**	**1**	**–**	**–**	**–**	**–**	**–**	**–**	**–**	**–**	**–**	**3**	**11**	**2**	**–**	**–**	**–**	**–**	**–**	**–**	**–**	**–**	**–**	**–**	**–**	**–**	**–**
**Lake Fwa**																												
**Pseudocrenilabrini**																												
Cyclopharynx schwetzi		10	4										13	1														
Schwetzochromis neodon		2												2														
**Lake Albert**																												
**Pseudocrenilabrini**																												
Haplochromis avium		1	2									3																
Haplochromis loati		1												1														
**Lake Edward–George system**																												
**Pseudocrenilabrini**																												
Haplochromis akika		1	3*											3*	1													
Haplochromis aquila		1*												1*														
Haplochromis aureus			1*											1*														
Haplochromis curvidens			1*											1*														
Haplochromis falcatus			1*												1*													
Haplochromis fuscus		1												1														
Haplochromis glaucus		1*												1*														
Haplochromis gracilifur		1*											1*															
Haplochromis kimondo		1*												1*														
Haplochromis labiatus		1												1														
Haplochromis latifrons		1*											1*															
Haplochromis limax		3	1											3	1													
Haplochromis mentatus		1*												1*														
Haplochromis molossus		1*											1*															
Haplochromis pappenheimi		1*											1*															
Haplochromis paradoxus		1*											1*															
Haplochromis pardus		1*												1*														
Haplochromis pelagicus		1*												1*														
Haplochromis pharyngalis		1*												1*														
Haplochromis quasimodo		1*												1*														
Haplochromis relictidens		1*											1*															
Haplochromis rex		1*												1*														
Haplochromis simba		1*												1*														
Haplochromis squamipinnis		1*												1*														
Haplochromis taurinus			1*											1*														
Schubotzia eduardiana		8	2										5	5														
**Pseudocrenilabrini (Ed–Grg) totals**	**–**	**31**	**10**	**–**	**–**	**–**	**–**	**–**	**–**	**–**	**–**	**–**	**11**	**27**	**3**	**–**	**–**	**–**	**–**	**–**	**–**	**–**	**–**	**–**	**–**	**–**	**–**	**–**
**Lake Victoria & satellites**																												
**Pseudocrenilabrini**																												
Allochromis welcommei			1												1													
Astatoreochromis alluaudi		1													1													
Haplochromis chlorochrous		3	1										3	1														
Haplochromis cryptogramma		8	2										10															
Haplochromis fusiformis		1												1														

	Anal pterygiophores anterior to HSp1									Total number of anal pterygiophores																		
	0	1	2	3	4	5	6	7	8	6	7	8	9	10	11	12	13	14	15	16	17	18	19	20	21	22	24	?
Haplochromis latifasciatus		6											1	5														
Haplochromis lividus		1	1											2														
Haplochromis nubilus		1*	2											2	1*													
Haplochromis plutonius		4	1										2	3														
Lithochromis rubripinnis		4												3	1													
Lithochromis xanthopteryx		3	1										1	2	1													
Mbipia mbipi		1											1															
Neochromis nigricans		1												1														
Paralabidochromis victoriae			2											2														
Pundamilia igneopinnis		1	1											2														
Pundamilia pundamilia		3	1										1	3														
Pyxichromis parorthostoma				1									1															
**Pseudocrenilabrini (Victoria) totals**	**–**	**38**	**13**	**1**	**–**	**–**	**–**	**–**	**–**	**–**	**–**	**–**	**20**	**27**	**5**	**–**	**–**	**–**	**–**	**–**	**–**	**–**	**–**	**–**	**–**	**–**	**–**	**–**
**Lake Kivu**																												
**Pseudocrenilabrini**																												
Haplochromis astatodon		5*	1										2	4*														
Haplochromis paucidens		2											2															
**Lake Turkana**																												
**Pseudocrenilabrini**																												
Haplochromis rudolfianus		8												8														
Haplochromis turkanae		2	1									1	2															
**Lake Tanganyika**																												
**Bathybatini**																												
Bathybates fasciatus		5																				5						
Bathybates ferox		10																			9	1						
Bathybates graueri		5																1	4									
Bathybates hornii		1																		1								
Bathybates leo		3	2																	2	3							
Bathybates minor		5																			1	4						
Bathybates vittatus		1																		1								
Hemibates stenosoma		4															1	3										
Trematocara unimaculatum		5												3	2													
Trematocara zebra		5										1	4															
**Bathybatini column totals**	**–**	**44**	**2**	**–**	**–**	**–**	**–**	**–**	**–**	**–**	**–**	**1**	**4**	**3**	**2**	**–**	**1**	**4**	**4**	**4**	**13**	**10**	**–**	**–**	**–**	**–**	**–**	**–**
**Benthochromini**																												
Benthochromis tricoti		5												1	4													
**Boulengerochromini**																												
Boulengerochromis microlepis		7												3	4													
**Cyphotilapiini**																												
Cyphotilapia frontosa		3	2									4	1															
Cyphotilapia gibberosa		1	4									2	3															
**Cyprichromini**																												
Cyprichromis coloratus							3	2								2	3											
Cyprichromis leptosoma						1	3	1								3	2											
Cyprichromis microlepidotus							3	2								1	3	1										
Cyprichromis pavo							1	4									3	2										
Cyprichromis zonatus						1	3	1								2	2	1										
Cyprichromis sp. "dwarf jumbo"							2	3								2	2	1										
Cyprichromis sp. "jumbo"						1	2	2								3	2											
Paracyprichromis brieni	2	–	–	3										2	3													
Paracyprichromis nigripinnis				4								2	2															
Paracyprichromis sp. "brieni south"			3	2										5														
**Cyprichromini column totals**	**2**	**–**	**3**	**9**	**–**	**3**	**17**	**15**	**–**	**–**	**–**	**2**	**2**	**7**	**3**	**13**	**17**	**5**	**–**	**–**	**–**	**–**	**–**	**–**	**–**	**–**	**–**	**–**
**Ectodini**																												
Asprotilapia leptura		1	3	1									4	1														
Aulonocranus dewindti		4	1										1	4														
Callochromis macrops		5										4	1															
Cardiopharynx schoutedeni		4	1											4	1													
Cunningtonia longiventralis		1	4										1	4														
Cyathopharynx furcifer		5												4	1													
Ectodus descampsii		1	4										2	3														
Enantiopus melanogenys				5																	1	4						
Grammatotria lemairii			4	1											1	4												
Lestradea perspicax			5	1										1	5													
Microdontochromis tenuidentatus		4	1										1	2	1	1												
Ophthalmotilapia boops		1	2	2											5													
Xenotilapia sima		5													3	2												
**Ectodini column totals**	**–**	**31**	**25**	**10**	**–**	**–**	**–**	**–**	**–**	**–**	**–**	**4**	**10**	**23**	**17**	**7**	**–**	**–**	**–**	**–**	**1**	**4**	**–**	**–**	**–**	**–**	**–**	**–**

	Anal pterygiophores anterior to HSp1									Total number of anal pterygiophores																		
	0	1	2	3	4	5	6	7	8	6	7	8	9	10	11	12	13	14	15	16	17	18	19	20	21	22	24	?
**Eretmodini**																												
Eretmodus cyanostictus		4	1									2	3															
Eretmodus marksmithi		5										5																
Spathodus erythrodon		5										5																
Tanganicodus irsacae		1										4	1															
**Eretmodini column totals**	**–**	**19**	**1**	**–**	**–**	**–**	**–**	**–**	**–**	**–**	**–**	**16**	**4**	**–**	**–**	**–**	**–**	**–**	**–**	**–**	**–**	**–**	**–**	**–**	**–**	**–**	**–**	**–**
**Lamprologini**																												
Altolamprologus calvus					5														3	2								
Altolamprologus compressiceps				5													2	3										
Altolamp. 'compressiceps shell'		1	3	1												1	2	2										
Chalinochromis brichardi			4	1										1	3	1												
Chalinochromis cyanophleps			5												4	1												
Julidochromis ornatus		1	4												5													
Julidochromis regani		2	3										3	1	1													
Lamprologus callipterus			1	8	1												2	7	1									
Lamprologus 'ornatipinnis congo'		5														4	1											
Lamprologus 'ornatipinnis zambia'		9													1	8												
Lamprologus speciosus			4	1											1	4												
Lepidiolamprologus kendalli			4	1											1	4												
Lepidiolamprologus mimicus			4	1											1	4												
Lepidiolamprologus profundicola			5												1	4												
Neolamprologus tretocephalus		5												5														
Neolamprologus ventralis		2	3											4	1													
Telmatochromis dhonti		4	1												4	1												
Telmatochromis temporalis		5													4	1												
Telmatochromis vittatus			5												5													
Variabilichromis moorii					7												3	4										
**Lamprologini column totals**	**–**	**34**	**46**	**18**	**13**	**–**	**–**	**–**	**–**	**–**	**–**	**–**	**3**	**11**	**32**	**33**	**10**	**16**	**4**	**2**	**–**	**–**	**–**	**–**	**–**	**–**	**–**	**–**
**Limnochromini**																												
Baileychromis centropomoides		4	1										3	2														
Gnathochromis permaxillaris		3	2									1	3	1														
Greenwoodochromis christyi		3	2										5															
Limnochromis auritus		5											5															
Reganochromis calliurus			5										3	2														
Tangachromis dhanisi		3	1									3	1															
Trematochromis benthicola		1	4									4	1															
Triglachromis otostigma		5											5															
**Limnochromini column totals**	**–**	**24**	**15**	**–**	**–**	**–**	**–**	**–**	**–**	**–**	**–**	**8**	**26**	**5**	**–**	**–**	**–**	**–**	**–**	**–**	**–**	**–**	**–**	**–**	**–**	**–**	**–**	**–**
**Oreochromini**																												
Oreochromis tanganicae		3	2											3	2													
**Perissodini**																												
Haplotaxodon microlepis		4	1											4	1													
Perissodus microlepis		2	3											2	3													
Plecodus paradoxus		5															5											
Xenochromis hecqui		5													5													
**Perissodini column totals**	**–**	**16**	**4**	**–**	**–**	**–**	**–**	**–**	**–**	**–**	**–**	**–**	**–**	**6**	**9**	**–**	**5**	**–**	**–**	**–**	**–**	**–**	**–**	**–**	**–**	**–**	**–**	**–**
**Pseudocrenilabrini: Tropheina**																												
Interochromis loocki		5										3	2															
Jabarichromis pfefferi		5										2	3															
Limnotilapia dardennii		4										1	3															
Lobochilotes labiata		4	1									5																
Petrochromis horii		1	4									1	3	1														
Petrochromis polyodon		3	2									1	4															
Pseudosimochromis curvifrons		5											3	2														
Shuja horei		8										8																
Simochromis diagramma		5											5															
Tropheus annectens		5											2	3														
Tropheus brichardi		4	1											5														
Tropheus duboisi		5											5															
Tropheus moorii		10												8	2													
**Tropheina column totals**	**–**	**64**	**8**	**–**	**–**	**–**	**–**	**–**	**–**	**–**	**–**	**21**	**30**	**19**	**2**	**–**	**–**	**–**	**–**	**–**	**–**	**–**	**–**	**–**	**–**	**–**	**–**	**–**
**Tylochromini**																												
Tylochromis polylepis		6										3	3															

	Anal pterygiophores anterior to HSp1									Total number of anal pterygiophores																		
	0	1	2	3	4	5	6	7	8	6	7	8	9	10	11	12	13	14	15	16	17	18	19	20	21	22	24	?
**Lake Malawi**																												
**Pseudocrenilabrini: Cyrtocarina**																												
Alticorpus mentale		1													1													
Alticorpus peterdaviesi		1												1														
Aristochromis christyi		1	1												1	1												
Aulonocara nyassae		1											1															
Aulonocara rostratum		1												1														
Aulonocara stonemani		1*											1*															
Buccochromis atritaeniatus		1	1*											2*														
Buccochromis heterotaenia		2*													2*													
Buccochromis nototaenia		2*													2*													
Buccochromis oculatus		3												1	2													
Buccochromis spectabilis		1													1													
Caprichromis orthognathus		5												3	2													
Champsochromis caeruleus		4													4													
Champsochromis spilorhynchus		2												2														
Cheilochromis euchilus		5*												5*														
Chilotilapia rhoadesii		4												1	3													
Copadichromis jacksoni		2	1														3											
Copadichromis quadrimaculatus			1													1												
Corematodus taeniatus		4	3											2	5													
Ctenopharynx nitidus		13	3											11	5													
Ctenopharynx pictus		1												1														
Cyrtocara moorii		4	2*											2	4*													
Dimidiochromis compressiceps		11	1												3	8	1											
Dimidiochromis kiwinge		3													2	1												
Docimodus evelynae		2*													2*													
Docimodus johnstoni		3												2	1													
Exochochromis anagenys		5*	2											1*	6													
Fossorochromis rostratus			1												1													
Hemitaeniochromis urotaenia		1												1														
Hemitilapia oxyrhynchus		8	2											2	8													
Lethrinops gossei		1	1*											2*														
Lethrinops lethrinus		2	1										1	2														
Lethrinops polli		1*												1*														
Lichnochromis acuticeps			1											1														
Mchenga cyclicos		1											1															
Mchenga inornata		2*												2*														
Mchenga sp.		4	19											3	16	4												
Mylochromis formosus		1	1*											2*														
Mylochromis gracilis		2	1*												3*													
Mylochromis guentheri		2											1	1														
Mylochromis lateristriga		3	1											1	3													
Mylochromis spilostichus		7*	1												8*													
Mylochromis subocularis		2											2															
Naevochromis chrysogaster		4*	1											3*	2													
Nimbochromis fuscotaeniatus		2												1	1													
Nimbochromis livingstonii		6												4	2													
Nimbochromis venustus		3	1												4													
Nyassachromis leuciscus		6*												2*	4													
Nyassachromis microcephalus		2*												1	1*													
Nyassachromis nigritaeniatus		2	2											4														
Nyassachromis purpurans		1												1														
Otopharynx argyrosoma		3*												3*														
Otopharynx decorus		5*	1											3	3*													
Otopharynx cf. *heterodon*		14	5										11	8														
Otopharynx heterodon		7	1										6	2														
Otopharynx lithobates		2	3*										4*	1														
Otopharynx ovatus		4*	1											1	4*													
Otopharynx selenurus		6												2	4													
Otopharynx speciosus		5												4	1													
Otopharynx tetraspilus		3	5											4	4													
Otopharynx tetrastigma		14	2*											10*	6													
Placidochromis hennydaviesae		1*											1*															
Placidochromis johnstoni		4												2	2													
Placidochromis longimanus		24	4										10	18														
Placidochromis macrognathus		1	1											2														
Placidochromis milomo		3	1											2	2													
Protomelas annectens		2												2														
Protomelas fenestratus	1	31	4										3	32	1													

	Anal pterygiophores anterior to HSp1									Total number of anal pterygiophores																		
	0	1	2	3	4	5	6	7	8	6	7	8	9	10	11	12	13	14	15	16	17	18	19	20	21	22	24	?
Protomelas insignis		1	1											1	1													
Protomelas kirkii		3*											2*	1														
Protomelas labridens		1													1													
Protomelas ornatus		1*	1											2														
Protomelas spilopterus		5	1											4	2													
Protomelas taeniolatus			1											1														
Protomelas triaenodon		2	1											1	2													
Sciaenochromis ahli		16												15	1													
Stigmatochromis modestus		4	1*											2	3*													
Stigmatochromis pholidophorus		4*	1											2	2	1*												
Stigmatochromis pleurospilus		1*																										1*
Stigmatochromis woodi		7*												4	2*	–	–	–	–	–	–	–	–	–	–	–	–	1
Taeniochromis holotaenia		1													1													
Taeniolethrinops praeorbitalis			1											1														
Tramitichromis brevis		1												1														
Trematocranus labifer		4												3	1													
Trematocranus microstoma		8	1											3	6													
Trematocranus placodon		3											1	2														
Tyrannochromis macrostoma		1												1														
Tyrannochromis nigriventer		1*													1*													
**Cyrtocarina column totals**	**1**	**339**	**85**	**–**	**–**	**–**	**–**	**–**	**–**	**–**	**–**	**–**	**45**	**209**	**149**	**16**	**4**	**–**	**–**	**–**	**–**	**–**	**–**	**–**	**–**	**–**	**–**	**2**
**Pseudocrenilabrini: Pseudotropheina**																												
Abactochromis labrosus		6*										5*	1															
Chindongo bellicosus		3	3									2	3	1														
Chindongo minutus			1										1															
Cyathochromis obliquidens		4											4															
Cynotilapia afra		1											1															
Cynotilapia sp.		3	1										3	1														
Genyochromis mento		8	2									1	8	–	–	–	–	–	–	–	–	–	–	–	–	–	–	1
Gephyrochromis lawsi		1											1															
Gephyrochromis moorii		3	1										4															
Iodotropheus sprengerae		5	1									4	1	1														
Labeotropheus fuelleborni		13	2									4	11															
Labeotropheus trewavasae		3										1	2															
Labidochromis caeruleus		1										1																
Labidochromis freibergi			1*									1*																
Labidochromis joanjohnsonae			1*										1*															
Labidochromis pallidus		20	3									20	3															
Labidochromis shiranus		1											1															
Labidochromis textilis		1	1*									1*	1															
Labidochromis vellicans		24	6									7	22	–	–	–	–	–	–	–	–	–	–	–	–	–	–	1
Maylandia aurora		1*											1*															
Maylandia koningsi		1										1																
Maylandia lanisticola		1*											1*															
Maylandia zebra		6	2	1								1	8															
Melanochromis auratus		6*										3*	3															
Melanochromis chipokae		1*											1*															
Melanochromis loriae		4*										2	2*															
Melanochromis melanopterus		9	2									3	6	1	–	–	–	–	–	–	–	–	–	–	–	–	–	1
Melanochromis simulans		1											1															
Melanochromis cf. *vermivorus*		1	1										1	–	–	–	–	–	–	–	–	–	–	–	–	–	–	1
Petrotilapia genalutea		1											1															
Petrotilapia nigra		1											1															
Petrotilapia tridentiger		1											1															
Petrotilapia sp.		4											4															
Pseudotropheus elegans		1*											1*															
Pseudotropheus interruptus		2*										1*	1															
Pseudotropheus johannii		31*	6									3	33*	1														
Pseudotropheus livingstonii		6	1*										6*	1														
Pseudotropheus lucerna		1										1																
Tropheops tropheops		7											7															
**Pseudotropheina column totals**	**–**	**183**	**35**	**1**	**–**	**–**	**–**	**–**	**–**	**–**	**–**	**62**	**147**	**6**	**–**	**–**	**–**	**–**	**–**	**–**	**–**	**–**	**–**	**–**	**–**	**–**	**–**	**4**
**Pseudocrenilabrini: Rhamphochromina**																												
Diplotaxodon argenteus			3*	7												7*	2	1										
Diplotaxodon ecclesi				1*													1*											
Diplotaxodon greenwoodi				1*												1*												
Diplotaxodon limnothrissa		2	–	6	2										2	5	3											
Pallidochromis tokolosh			4													3	1											
Rhamphochromis brevis	1*	–	1												1*	–	1											
Rhamphochromis esox			2												1	1												

	Anal pterygiophores anterior to HSp1									Total number of anal pterygiophores																		
	0	1	2	3	4	5	6	7	8	6	7	8	9	10	11	12	13	14	15	16	17	18	19	20	21	22	24	?
Rhamphochromis woodi		1													1													
**Rhamphochromina column totals**	**1**	**3**	**10**	**15**	**2**	**–**	**–**	**–**	**–**	**–**	**–**	**–**	**–**	**–**	**5**	**17**	**8**	**1**	**–**	**–**	**–**	**–**	**–**	**–**	**–**	**–**	**–**	**–**
**Cyrtocarina column totals**	**1**	**339**	**85**	**–**	**–**	**–**	**–**	**–**	**–**	**–**	**–**	**–**	**45**	**209**	**149**	**16**	**4**	**–**	**–**	**–**	**–**	**–**	**–**	**–**	**–**	**–**	**–**	**2**
**Pseudotropheina column totals**	**–**	**183**	**35**	**1**	**–**	**–**	**–**	**–**	**–**	**–**	**–**	**62**	**147**	**6**	**–**	**–**	**–**	**–**	**–**	**–**	**–**	**–**	**–**	**–**	**–**	**–**	**–**	**4**
**Rhamphochromina column totals**	**1**	**3**	**10**	**15**	**2**	**–**	**–**	**–**	**–**	**–**	**–**	**–**	**–**	**–**	**5**	**17**	**8**	**1**	**–**	**–**	**–**	**–**	**–**	**–**	**–**	**–**	**–**	**–**
