# Supplementary material for: African cichlid fishes: morphological data and taxonomic insights from a genus-level survey of supraneurals, pterygiophores, and vertebral counts (Ovalentaria, Blenniiformes, Cichlidae, Pseudocrenilabrinae)
Source: Biodivers Data J. 2024 Oct 18;12:e130707. doi: 10.3897/BDJ.12.e130707 (PMC11512106; doi:10.3897/BDJ.12.e130707)
Supplement: Supplementary material 5 — Table S4. [file bdj-12-e130707-s005.pdf]

Table 5. Frequency distribution of number of dorsal pterygiophores minus number of anal pterygiophores

	Total dorsal pterygiophores minus total anal pterygiophores																					
	2	4	5	6	7	8	9	10	11	12	13	14	15	16	17	18	19	20	21	22	23	?
Polycentridae																						
Afronandus sheljuzhkoi											1											
Monocirrhus polyacanthus	1	1																				
Polycentropsis abbreviata					1	1																
Cichlidae: Etroplinae																						
**India**																						
Etroplus suratensis								1														
Pseudetroplus maculatus			2	1	3	–	1															
**Madagascar**																						
Paretroplus polyactis										1												
Ptychochrominae																						
**Madagascar**																						
Katria katria											1											
Oxylapia polli												1										
Paratilapia polleni								5	4	2												
Ptychochromis oligacanthus												8	3									
Ptychochromoides betsileanus												2	1									
Cichlinae																						
**Neotropics**																						
**Astronotini**																						
Astronotus ocellatus													3									
**Chaetobranchini**																						
Chaetobranchopsis orbicularis				1																		
Chaetobranchus flavescens											4											
**Cichlasomatini**																						
Aequidens tetramerus														1								
Andinoacara pulcher												1										
Bujurquina vittata											1											
Cichlasoma dimerus											1											
Cichlasoma taenia													1									
Krobia guianensis												1										
**Cichlini**																						
Cichla ocellaris																	1					
Cichla orinocensis																1	2					
**Geophagini**																						
Biotodoma cupido												2	2									
Geophagus crassilabris															1							
Guianacara owroewefi													8									
Saxatilia frenata																			1*			
Saxatilia lepidota																	1	1				
**Heroini**																						
Amphilophus citrinellus																						
Hypselacara coryphaenoides												5										
**Retroculini**																						
Retroculus lapidifer																1						
Pseudocrenilabrinae																						
**Middle East**																						
**Oreochromini**																						
Iranocichla hormuzensis														2								
Tristramella sacra												1										
Tristramella simonis												2	2									
**Oreochromini (M.E.) column totals**	–	–	–	–	–	–	–	–	–	–	–	**3**	**2**	**2**	–	–	–	–	–	–	–	–
**Pseudocrenilabrini**																						
Astatotilapia flaviijosephi										2	2											

	Total dorsal pterygiophores minus total anal pterygiophores																					
	2	4	5	6	7	8	9	10	11	12	13	14	15	16	17	18	19	20	21	22	23	?
**African riverine**																						
**Chromidotilapiini**																						
Benitochromis batesii													1	5								
Benitochromis finleyi														1								
Chromidotilapia guntheri															1	1						
Chromidotilapia kingsleyae													5	4								
Congochromis robustus																1*						
Congochromis squamiceps														2								
Divandu albimarginatus													1	2	1							
Limbochromis robertsi												1	13	2	1							
Nanochromis nudiceps															2							
Nanochromis parilus																2						
Parananochromis gabonicus													1*									
Parananochromis longirostris													3	7	1							
Pelmatochromis buettikoferi														3								
Pelmatochromis ocellifer														1*								
Pelvicachromis pulcher														1								
Pelvicachromis taeniatus														1*								
Pterochromis congicus											1	1*										
Teleogramma brichardi															1	3						
Thysochromis ansorgii												2	1	1								
Wallaceochromis humilis																4*	1					
**Chromidotilapiini column totals**	**–**	**–**	**–**	**–**	**–**	**–**	**–**	**–**	**–**	**–**	**1**	**4**	**25**	**30**	**7**	**11**	**1**	**–**	**–**	**–**	**–**	**–**
**Coelotilapiini**																						
Coelotilapia joka																1						
**Coptodonini**																						
Coptodon discolor															2							
Coptodon tholloni													1									
Coptodon zillii													2	8	10	4						
**Coptodonini column totals**	**–**	**–**	**–**	**–**	**–**	**–**	**–**	**–**	**–**	**–**	**–**	**–**	**3**	**8**	**12**	**4**	**–**	**–**	**–**	**–**	**–**	**–**
**Etiini**																						
Etia nguti													6									
**Gobiocichlini**																						
Gobiocichla ethelwynnae														2	6	3						
Gobiocichla wonderi																			10	3	1	
Paragobiocichla irvinei																		1	3			
**Gobiocichlini column totals**	**–**	**–**	**–**	**–**	**–**	**–**	**–**	**–**	**–**	**–**	**–**	**–**	**–**	**2**	**6**	**3**	**–**	**1**	**13**	**3**	**1**	**–**
**Hemichromini**																						
Anomalochromis thomasi											1	3										
Hemichromis elongatus													4									
Hemichromis fasciatus														1								
Rubricatochromis bimaculatus												2	4									
Rubricatochromis letourneuxi											1	1	2									
**Hemichromini column totals**	**–**	**–**	**–**	**–**	**–**	**–**	**–**	**–**	**–**	**–**	**2**	**6**	**10**	**1**	**–**	**–**	**–**	**–**	**–**	**–**	**–**	**–**
**Heterochromini**																						
Heterochromis multidens															5	6						
**Heterotilapiini**																						
Heterotilapia buettikoferi														1	1	3						
**Oreochromini**																						
Danakilia franchettii												3	6	5								
Oreochromis jipe															3	1						
Oreochromis niloticus												1	–	2								
Oreochromis shiranus													4	2								
Sarotherodon galilaeus																2						
Sarotherodon melanotheron													3	3								
**Oreochromini (riverine) totals**	**–**	**–**	**–**	**–**	**–**	**–**	**–**	**–**	**–**	**–**	**–**	**4**	**13**	**12**	**3**	**3**	**–**	**–**	**–**	**–**	**–**	**–**

	Total dorsal pterygiophores minus total anal pterygiophores																					
	2	4	5	6	7	8	9	10	11	12	13	14	15	16	17	18	19	20	21	22	23	?
**Pelmatolapiini**																						
Pelmatolapia mariae														2	4							
**Pseudocrenilabrini**																						
Astatotilapia bloyeti										2	5	1										
Astatotilapia burtoni										1	3	2										
Astatotilapia calliptera												3	6*									
Astatotilapia swynnertoni											3											
Chetia flaviventris													1									
Chetia gracilis														1*								
Chetia ? sp.												7	–	–	–	–	–	–	–	–	–	1
Ctenochromis pectoralis												2										
Ctenochromis scatebra													1*									
Haplochromis demeusii														1*								
Haplochromis fasciatus														3	3							
Haplochromis humilis													2	1								
Haplochromis moeruensis													2	1	1							
Haplochromis oligacanthus												1*										
Lufubuchromis relictus													1*									
Orthochromis machadoi														1	1							
Orthochromis malagaraziensis													1	1								
Orthochromis polyacanthus																1	1					
Orthochromis stormsi															8	3						
Palaeoplex palimpsest													1*									
Pharyngochromis acuticeps												1	3	2								
Pseudocrenilabrus multicolor												4										
Pseudocrenilabrus philander												1										
Sargochromis carlottae													1	1								
Sargochromis codringtonii														1	1*	1						
Sargochromis giardi														1	1							
Sargochromis greenwoodi															1*	–	1					
Serranochromis angusticeps															1							
Serranochromis longimanus													1	3*								
Serranochromis macrocephalus																1						
Serranochromis meridianus														1								
Serranochromis robustus															3	3						
Thoracochromis albolabris														1								
Thoracochromis wingatii												2										
**Pseudocrenilabrini (riverine) totals**	**–**	**–**	**–**	**–**	**–**	**–**	**–**	**–**	**–**	**3**	**11**	**24**	**20**	**19**	**20**	**9**	**2**	**–**	**–**	**–**	**–**	**1**
**Steatocranini**																						
Steatocranus casuarius																	1	1	1			
**Tilapiini**																						
Chilochromis duponti															1							
Congolapia bilineata														2	1							
Tilapia busumana												1	–	–	1	2						
Tilapia sparrmanii										1*	–	3										
**Tilapiini column totals**	**–**	**–**	**–**	**–**	**–**	**–**	**–**	**–**	**–**	**1**	**–**	**4**	**–**	**2**	**3**	**2**	**–**	**–**	**–**	**–**	**–**	**–**
**Lake Barombi Mbo**																						
**Oreochromini**																						
Konia eisentrauti													1	4								
Myaka myaka												1	2	1								
Pungu maclareni													2*									
Stomatepia mariae												1										
Stomatepia pindu											1	3										
**Oreochromini (Barombi) totals**	**–**	**–**	**–**	**–**	**–**	**–**	**–**	**–**	**–**	**–**	**1**	**5**	**5**	**5**	**–**	**–**	**–**	**–**	**–**	**–**	**–**	**–**

	Total dorsal pterygiophores minus total anal pterygiophores																					
	2	4	5	6	7	8	9	10	11	12	13	14	15	16	17	18	19	20	21	22	23	?
**Lake Fwa**																						
**Pseudocrenilabrini**																						
Cyclopharynx schwetzi													4	10								
Schwetzochromis neodon															2							
**Lake Albert**																						
**Pseudocrenilabrini**																						
Haplochromis avium											1	1	1									
Haplochromis loati											1											
**Lake Edward–George system**																						
**Pseudocrenilabrini**																						
Haplochromis akika											3*	1										
Haplochromis aquila												1*										
Haplochromis aureus												1*										
Haplochromis curvidens												1*										
Haplochromis falcatus										1*												
Haplochromis fuscus												1										
Haplochromis glaucus											1*											
Haplochromis gracilifur												1*										
Haplochromis kimondo												1*										
Haplochromis labiatus												1										
Haplochromis latifrons												1*										
Haplochromis limax											2	2										
Haplochromis mentatus												1*										
Haplochromis molossus												1*										
Haplochromis pappenheimi												1*										
Haplochromis paradoxus													1*									
Haplochromis pardus												1*										
Haplochromis pelagicus														1*								
Haplochromis pharyngalis												1*										
Haplochromis quasimodo												1*										
Haplochromis relictidens												1*										
Haplochromis rex												1*										
Haplochromis simba												1*										
Haplochromis squamipinnis												1*										
Haplochromis taurinus												1*										
Schubotzia eduardiana											1	8	1									
**Pseudocrenilabrini (Ed–Grg) totals**	**–**	**–**	**–**	**–**	**–**	**–**	**–**	**–**	**–**	**1**	**7**	**30**	**2**	**1**	**–**	**–**	**–**	**–**	**–**	**–**	**–**	**–**
**Lake Victoria & satellites**																						
**Pseudocrenilabrini**																						
Allochromis welcommei											1											
Astatoreochromis alluaudi												1										
Haplochromis chlorochrous											2	2										
Haplochromis cryptogramma										1	4	5										
Haplochromis fusiformis													1									
Haplochromis latifasciatus											2	4										
Haplochromis lividus												2										
Haplochromis nubilus											1*	2										
Haplochromis plutonius											1	4										
Lithochromis rubripinnis											1	3										
Lithochromis xanthopteryx												3	1									
Mbipia mbipi													1									
Neochromis nigricans													1									
Paralabidochromis victoriae											1	1										
Pundamilia igneopinnis												1	1									
Pundamilia pundamilia												4										
Pyxichromis parorthostoma													1									
**Pseudocrenilabrini (Victoria) totals**	**–**	**–**	**–**	**–**	**–**	**–**	**–**	**–**	**–**	**1**	**13**	**32**	**6**	**–**	**–**	**–**	**–**	**–**	**–**	**–**	**–**	**–**

	Total dorsal pterygiophores minus total anal pterygiophores																					
	2	4	5	6	7	8	9	10	11	12	13	14	15	16	17	18	19	20	21	22	23	?
**Lake Kivu**																						
**Pseudocrenilabrini**																						
Haplochromis astatodon											4*	2										
Haplochromis paucidens													2									
**Lake Turkana**																						
**Pseudocrenilabrini**																						
Haplochromis rudolfianus										1	4	3										
Haplochromis turkanae											2	1										
**Lake Tanganyika**																						
**Bathybatini**																						
Bathybates fasciatus											2	3										
Bathybates ferox									8	2												
Bathybates graueri									4	–	1											
Bathybates hornii										1												
Bathybates leo									2	2	1											
Bathybates minor							3	2														
Bathybates vittatus										1												
Hemibates stenosoma											2	2										
Trematocara unimaculatum									2	3												
Trematocara zebra									3	1	1											
**Bathybatini column totals**	**–**	**–**	**–**	**–**	**–**	**–**	**3**	**2**	**19**	**10**	**7**	**5**	**–**	**–**	**–**	**–**	**–**	**–**	**–**	**–**	**–**	**–**
**Benthochromini**																						
Benthochromis tricoti														1	2	2						
Boulengerochromini																						
Boulengerochromis microlepis																	5	2				
**Cyphotilapiini**																						
Cyphotilapia frontosa															1	1	3					
Cyphotilapia gibberosa																5						
**Cyprichromini**																						
Cyprichromis coloratus														5								
Cyprichromis leptosoma												2	3									
Cyprichromis microlepidotus												1	3	1								
Cyprichromis pavo													1	3	1							
Cyprichromis zonatus													3	2								
Cyprichromis sp. "dwarf jumbo"													3	2								
Cyprichromis sp. "jumbo"													1	3	1							
Paracyprichromis brieni													1	1	3							
Paracyprichromis nigripinnis															2	1	1					
Paracyprichromis sp. "brieni south"														1	4							
**Cyprichromini column totals**	**–**	**–**	**–**	**–**	**–**	**–**	**–**	**–**	**–**	**–**	**–**	**3**	**15**	**18**	**11**	**1**	**1**	**–**	**–**	**–**	**–**	**–**
**Ectodini**																						
Asprotilapia leptura																2	3					
Aulonocranus dewindti												3	2									
Callochromis macrops															1	4						
Cardiopharynx schoutedeni														3	2							
Cunningtonia longiventralis														2	3							
Cyathopharynx furcifer												1	–	3	1							
Ectodus descampsii													2	3								
Enantiopus melanogenys										3	2											
Grammatotria lemairii													2	3								
Lestradea perspicax														1	5							
Microdontochromis tenuidentatus												1	1	3								
Ophthalmotilapia boops													1	4								
Xenotilapia sima													3	2								
**Ectodini column totals**	**–**	**–**	**–**	**–**	**–**	**–**	**–**	**–**	**–**	**3**	**2**	**5**	**11**	**24**	**12**	**6**	**3**	**–**	**–**	**–**	**–**	**–**

	Total dorsal pterygiophores minus total anal pterygiophores																					
	2	4	5	6	7	8	9	10	11	12	13	14	15	16	17	18	19	20	21	22	23	?
**Eretmodini**																						
Eretmodus cyanostictus																4	1					
Eretmodus marksmithi																	5					
Spathodus erythrodon																	5					
Tanganicodus irsacae																1	4					
**Eretmodini column totals**	**–**	**–**	**–**	**–**	**–**	**–**	**–**	**–**	**–**	**–**	**–**	**–**	**–**	**–**	**–**	**5**	**15**	**–**	**–**	**–**	**–**	**–**
**Lamprologini**																						
Altolamprologus calvus									3	2												
Altolamprologus compressiceps										4	1											
Altolamp. 'compressiceps shell'									2	2	1											
Chalinochromis brichardi															1	4						
Chalinochromis cyanophleps															2	3						
Julidochromis ornatus															5							
Julidochromis regani															1	1	3					
Lamprologus callipterus										1	8	1										
Lamprologus 'ornatipinnis congo'										1	4											
Lamprologus 'ornatipinnis zambia'										1	7	1										
Lamprologus speciosus									4	1												
Lepidiolamprologus kendalli														4	1							
Lepidiolamprologus mimicus														4	1							
Lepidiolamprologus profundicola														3	2							
Neolamprologus tretocephalus														1	4							
Neolamprologus ventralis												1	3	1								
Telmatochromis dhonti												1	3	1								
Telmatochromis temporalis													1	3	1							
Telmatochromis vittatus														1	3	1						
Variabilichromis moorii												4	3									
**Lamprologini column totals**	**–**	**–**	**–**	**–**	**–**	**–**	**–**	**–**	**9**	**12**	**21**	**8**	**10**	**18**	**21**	**9**	**3**	**–**	**–**	**–**	**–**	**–**
**Limnochromini**																						
Baileychromis centropomoides													1	4								
Gnathochromis permaxillaris														1	4							
Greenwoodochromis christyi													3	2								
Limnochromis auritus														3	2							
Reganochromis calliurus															1	4						
Tangachromis dhanisi												2	1	1								
Trematochromis benthicola															5							
Triglachromis otostigma												1	4									
**Limnochromini column totals**	**–**	**–**	**–**	**–**	**–**	**–**	**–**	**–**	**–**	**–**	**–**	**3**	**9**	**11**	**12**	**4**	**–**	**–**	**–**	**–**	**–**	**–**
Oreochromini																						
Oreochromis tanganicae															2	1	2					
**Perissodini**																						
Haplotaxodon microlepis																3	2					
Perissodus microlepis															2	2	–	1				
Plecodus paradoxus																4	1					
Xenochromis hecqui														4	1							
**Perissodini column totals**	**–**	**–**	**–**	**–**	**–**	**–**	**–**	**–**	**–**	**–**	**–**	**–**	**–**	**4**	**3**	**9**	**3**	**1**	**–**	**–**	**–**	**–**
**Pseudocrenilabrini: Tropheina**																						
Interochromis loocki															1	4						
Jabarichromis pfefferi														4	1							
Limnotilapia dardennii																2	2					
Lobochilotes labiata																	5					
Petrochromis horii															1	–	4					
Petrochromis polyodon															3	2						
Pseudosimochromis curvifrons														1	4							
Shuja horei													1	6	1							
Simochromis diagramma																4	1					
Tropheus annectens															3	2						

	Total dorsal pterygiophores minus total anal pterygiophores																					
	2	4	5	6	7	8	9	10	11	12	13	14	15	16	17	18	19	20	21	22	23	?
Tropheus brichardi													2	3								
Tropheus duboisi															5							
Tropheus moorii														8	2							
**Tropheina column totals**	**–**	**–**	**–**	**–**	**–**	**–**	**–**	**–**	**–**	**–**	**–**	**–**	**3**	**22**	**21**	**14**	**12**	**–**	**–**	**–**	**–**	**–**
**Tylochromini**																						
Tylochromis polylepis																	2	4				
**Lake Malawi**																						
**Pseudocrenilabrini: Cyrtocarina**																						
Alticorpus mentale											1											
Alticorpus peterdaviesi												1										
Aristochromis christyi													1	1								
Aulonocara nyassae														1								
Aulonocara rostratum													1									
Aulonocara stonemani														1*								
Buccochromis atritaeniatus													1	1*								
Buccochromis heterotaenia														2*								
Buccochromis nototaenia														1	1*							
Buccochromis oculatus													1	2								
Buccochromis spectabilis															1							
Caprichromis orthognathus													2	3								
Champsochromis caeruleus															2	2						
Champsochromis spilorhynchus																2						
Cheilochromis euchilus												1*	4									
Chilotilapia rhoadesii												1	3									
Copadichromis jacksoni													1	2								
Copadichromis quadrimaculatus													1									
Corematodus taeniatus												4	3									
Ctenopharynx nitidus														5	11							
Ctenopharynx pictus														1								
Cyrtocara moorii												1	2	3*								
Dimidiochromis compressiceps											3	9										
Dimidiochromis kiwinge													2	1								
Docimodus evelynae														1*	1							
Docimodus johnstoni												1	–	2								
Exochochromis anagenys													3	3	1*							
Fossorochromis rostratus													1									
Hemitaeniochromis urotaenia													1									
Hemitilapia oxyrhynchus												2	5	3								
Lethrinops gossei													2*									
Lethrinops lethrinus												1	2									
Lethrinops polli													1*									
Lichnochromis acuticeps													1									
Mchenga cyclicos															1							
Mchenga inornata															1	1*						
Mchenga sp.													4	12	7							
Mylochromis formosus																2*						
Mylochromis gracilis												1	1	–	1*							
Mylochromis guentheri														1	–	1						
Mylochromis lateristriga													3	1								
Mylochromis spilostichus															4	4*						
Mylochromis subocularis														1	1							
Naevochromis chrysogaster													5*									
Nimbochromis fuscotaeniatus													2									
Nimbochromis livingstonii												1	4	1								
Nimbochromis venustus												2	2									
Nyassachromis leuciscus													3	2	1*							
Nyassachromis microcephalus															2*							

	Total dorsal pterygiophores minus total anal pterygiophores																					
	2	4	5	6	7	8	9	10	11	12	13	14	15	16	17	18	19	20	21	22	23	?
Nyassachromis nigritaeniatus															4							
Nyassachromis purpurans															1							
Otopharynx argyrosoma														1*	2							
Otopharynx decorus															1	5*						
Otopharynx cf. *heterodon*														5	13	1						
Otopharynx heterodon															7	1						
Otopharynx lithobates														1	4*							
Otopharynx ovatus														4*	–	1						
Otopharynx selenurus														5	1							
Otopharynx speciosus												1	2	2								
Otopharynx tetraspilus													7	1								
Otopharynx tetrastigma											1	7	6*	2								
Placidochromis hennydaviesae													1*									
Placidochromis johnstoni												2	2									
Placidochromis longimanus													7	17	4							
Placidochromis macrognathus													2									
Placidochromis milomo														4								
Protomelas annectens														1	1							
Protomelas fenestratus												1	4	21	10							
Protomelas insignis															2							
Protomelas kirkii													3*									
Protomelas labridens												1										
Protomelas ornatus													2*									
Protomelas spilopterus												1	3	2								
Protomelas taeniolatus														1								
Protomelas triaenodon													2	1								
Sciaenochromis ahli												2	12	2								
Stigmatochromis modestus													4*	1								
Stigmatochromis pholidophorus													3*	2								
Stigmatochromis pleurospilus																						1*
Stigmatochromis woodi												2*	3	–	–	–	–	–	–	–	–	2
Taeniochromis holotaenia															1							
Taeniolethrinops praeorbitalis														1								
Tramitichromis brevis													1									
Trematocranus labifer												1	3									
Trematocranus microstoma												3	2	4								
Trematocranus placodon													2	1								
Tyrannochromis macrostoma														1								
Tyrannochromis nigriventer													1*									
**Cyrtocarina column totals**	**–**	**–**	**–**	**–**	**–**	**–**	**–**	**–**	**–**	**–**	**5**	**46**	**134**	**131**	**86**	**20**	**–**	**–**	**–**	**–**	**–**	**3**
**Pseudocrenilabrini: Pseudotropheina**																						
Abactochromis labrosus													5*	1								
Chindongo bellicosus															3	2	1					
Chindongo minutus														1								
Cyathochromis obliquidens													3	1								
Cynotilapia afra														1								
Cynotilapia sp.													2	2								
Genyochromis mento														7	3							
Gephyrochromis lawsi															1							
Gephyrochromis moorii															4							
Iodotropheus sprengerae												2	–	2	2							
Labeotropheus fuelleborni													1	8	4	2						
Labeotropheus trewavasae															2	1						
Labidochromis caeruleus															1							
Labidochromis freibergi														1*								
Labidochromis joanjohnsonae														1*								
Labidochromis pallidus												1	1	16	5							

Table 5 (continued). Frequency distribution of number of dorsal pterygiophores minus number of anal pterygiophores

	Total dorsal pterygiophores minus total anal pterygiophores																					
	2	4	5	6	7	8	9	10	11	12	13	14	15	16	17	18	19	20	21	22	23	?
Labidochromis shiranus														1								
Labidochromis textilis														2*								
Labidochromis vellicans													7	17	5	–	–	–	–	–	–	1
Maylandia aurora														1*								
Maylandia koningsi														1								
Maylandia lanisticola														1*								
Maylandia zebra														2	6	1						
Melanochromis auratus														1	2	3*						
Melanochromis chipokae														1*								
Melanochromis loriae															1*	3						
Melanochromis melanopterus														2	6	2	–	–	–	–	–	1
Melanochromis simulans														1								
Melanochromis cf. vermivorus														1	–	–	–	–	–	–	–	1
Petrotilapia genalutea															1							
Petrotilapia nigra														1								
Petrotilapia tridentiger															1							
Petrotilapia sp.														1	3							
Pseudotropheus elegans															1*							
Pseudotropheus interruptus														1	–	1*						
Pseudotropheus johannii														6	27*	4						
Pseudotropheus livingstonii													1	4*	2							
Pseudotropheus lucerna																1						
Tropheops tropheops															5	2						
**Pseudotropheina column totals**	**–**	**–**	**–**	**–**	**–**	**–**	**–**	**–**	**–**	**–**	**–**	**3**	**20**	**85**	**85**	**22**	**1**	**–**	**–**	**–**	**–**	**3**
**Pseudocrenilabrini: Rhamphochromina**																						
Diplotaxodon argenteus											1	6*	2	1								
Diplotaxodon ecclesi											1*											
Diplotaxodon greenwoodi											1*											
Diplotaxodon limnothrissa													4	4	1	–	–	–	–	–	–	1
Pallidochromis tokolosh									1	–	1	2										
Rhamphochromis brevis													1	–	–	1*						
Rhamphochromis esox																	2					
Rhamphochromis woodi															1							
**Rhamphochromina column totals**	**–**	**–**	**–**	**–**	**–**	**–**	**–**	**–**	**1**	**–**	**4**	**8**	**7**	**5**	**2**	**1**	**2**	**–**	**–**	**–**	**–**	**1**
**Cyrtocarina column totals**	**–**	**–**	**–**	**–**	**–**	**–**	**–**	**–**	**–**	**–**	**5**	**46**	**134**	**131**	**86**	**20**	**–**	**–**	**–**	**–**	**–**	**3**
**Pseudotropheina column totals**	**–**	**–**	**–**	**–**	**–**	**–**	**–**	**–**	**–**	**–**	**–**	**3**	**20**	**85**	**85**	**22**	**1**	**–**	**–**	**–**	**–**	**3**
**Rhamphochromina column totals**	**–**	**–**	**–**	**–**	**–**	**–**	**–**	**–**	**1**	**–**	**4**	**8**	**7**	**5**	**2**	**1**	**2**	**–**	**–**	**–**	**–**	**1**
