# Supplementary material for: African cichlid fishes: morphological data and taxonomic insights from a genus-level survey of supraneurals, pterygiophores, and vertebral counts (Ovalentaria, Blenniiformes, Cichlidae, Pseudocrenilabrinae)
Source: Biodivers Data J. 2024 Oct 18;12:e130707. doi: 10.3897/BDJ.12.e130707 (PMC11512106; doi:10.3897/BDJ.12.e130707)
Supplement: Supplementary material 6 — Table S5. [file bdj-12-e130707-s006.pdf]

Table 6. Frequency distribution of counts of longest series of one pterygiophore per dorsal insertion space

	Longest consecutive series of 1 Pt per DIS																								
	4	5	6	7	8	9	10	11	12	13	14	15	16	17	18	19	20	21	22	23	24	25	26	27	28
Polycentridae																									
Afronandus sheljuzhkoi		1																							
Monocirrhus polyacanthus	1	–	–	1																					
Polycentropsis abbreviata		1	–	–	–	–	1																		
Cichlidae: Etroplinae																									
**India**																									
Etroplus suratensis														1											
Pseudetroplus maculatus												4	2	1											
**Madagascar**																									
Paretroplus polyactis															1										
Ptychochrominae																									
**Madagascar**																									
Katria katria											1														
Oxylapia polli											1														
Paratilapia polleni					1	1	1	2	3	3															
Ptychochromis oligacanthus											7	4													
Ptychochromoides betsileanus										2	1														
Cichlinae																									
**Neotropics**																									
**Astronotini**																									
Astronotus ocellatus												1	2												
**Chaetobranchini**																									
Chaetobranchopsis orbicularis												1													
Chaetobranchus flavescens											3	1													
**Cichlasomatini**																									
Aequidens tetramerus											1														
Andinoacara pulcher										1															
Bujurquina vittata										1															
Cichlasoma dimerus										1															
Cichlasoma taenia											1														
Krobia guianensis									1																
**Cichlini**																									
Cichla ocellaris										1															
Cichla orinocensis			1	–	–	–	–	–	–	–	1	1													
**Geophagini**																									
Biotodoma cupido												4													
Geophagus crassilabris													1												
Guianacara owroewefi										1	7														
Saxatilia frenata																			1*						
Saxatilila lepidota																	1	1							
**Heroini**																									
Amphilophus citrinellus															1										
Hypselacara coryphaenoides													5												
**Retroculini**																									
Retroculus lapidifer															18										
Pseudocrenilabrinae																									
**Middle East**																									
**Oreochromini**																									
Iranocichla hormuzensis											1	1													
Tristramella sacra												1													
Tristramella simonis												4													
**Oreochromini (M.E.) column totals**	**–**	**–**	**–**	**–**	**–**	**–**	**–**	**–**	**–**	**–**	**1**	**6**	**–**	**–**	**–**	**–**	**–**	**–**	**–**	**–**	**–**	**–**	**–**	**–**	**–**
**Pseudocrenilabrini**																									
Astatotilapia flaviijosephi											3	1													
**African riverine**																									
**Chromidotilapiini**																									
Benitochromis batesii											6														
Benitochromis finleyi												1													
Chromidotilapia guntheri												2													
Chromidotilapia kingsleyae			1	–	–	–	–	–	–	4	4														
Congochromis robustus													1*												

	Longest consecutive series of 1 Pt per DIS																								
	4	5	6	7	8	9	10	11	12	13	14	15	16	17	18	19	20	21	22	23	24	25	26	27	28
Congochromis squamiceps													2												
Divandu albimarginatus										4															
Limbochromis robertsi												17													
Nanochromis nudiceps													1	–	–	1									
Nanochromis parilus																1	1								
Parananochromis gabonicus												1*													
Parananochromis longirostris										1	9	1													
Pelmatochromis buettikoferi											3														
Pelmatochromis ocellifer											1*														
Pelvicachromis pulcher												1													
Pelvicachromis taeniatus													1*												
Pterochromis congicus										2*															
Teleogramma brichardi																				2	1	1			
Thysochromis ansorgii											4														
Wallaceochromis humilis												2	2*	1											
**Chromidotilapiini column totals**	**–**	**–**	**1**	**–**	**–**	**–**	**–**	**–**	**–**	**11**	**27**	**25**	**7**	**1**	**–**	**2**	**1**	**–**	**–**	**2**	**1**	**1**	**–**	**–**	**–**
**Coelotilapiini**																									
Coelotilapia joka												1													
**Coptodonini**																									
Coptodon discolor										1	–	1													
Coptodon tholloni											1														
Coptodon zillii									1	2	3	6	9	3											
**Coptodonini column totals**	**–**	**–**	**–**	**–**	**–**	**–**	**–**	**–**	**1**	**3**	**4**	**8**	**9**	**3**	**–**	**–**	**–**	**–**	**–**	**–**	**–**	**–**	**–**	**–**	**–**
**Etiini**																									
Etia nguti											6														
**Gobiocichlini**																									
Gobiocichla ethelwynnae																		7	2	2					
Gobiocichla wonderi																					1	10	2	–	1
Paragobiocichla irvinei															1	3									
**Gobiocichlini column totals**	**–**	**–**	**–**	**–**	**–**	**–**	**–**	**–**	**–**	**–**	**–**	**–**	**–**	**–**	**1**	**3**	**–**	**7**	**2**	**2**	**1**	**10**	**2**	**–**	**1**
**Hemichromini**																									
Anomalochromis thomasi									3	1															
Hemichromis elongatus										2	2														
Hemichromis fasciatus										1															
Rubricatochromis bimaculatus											5	1													
Rubricatochromis letourneuxi										1	2			1											
**Hemichromini column totals**	**–**	**–**	**–**	**–**	**–**	**–**	**–**	**–**	**3**	**5**	**9**	**1**	**–**	**1**	**–**	**–**	**–**	**–**	**–**	**–**	**–**	**–**	**–**	**–**	**–**
**Heterochromini**																									
Heterochromis multidens							1	–	7	3															
**Heterotilapiini**																									
Heterotilapia buettikoferi											3	2													
**Oreochromini**																									
Danakilia franchettii											10	3	1												
Oreochromis jipe															3	1									
Oreochromis niloticus											1	–	–	2											
Oreochromis shiranus													5	1											
Sarotherodon galilaeus													2												
Sarotherodon melanotheron											3	3													
**Oreochromini (riverine) totals**	**–**	**–**	**–**	**–**	**–**	**–**	**–**	**–**	**–**	**–**	**14**	**6**	**8**	**3**	**3**	**1**	**–**	**–**	**–**	**–**	**–**	**–**	**–**	**–**	**–**
**Pelmatolapiini**																									
Pelmatolapia mariae												6													
**Pseudocrenilabrini**																									
Astatotilapia bloyeti													6	2											
Astatotilapia burtoni												3	3												
Astatotilapia calliptera													2*	7											
Astatotilapia swynnertoni													1	1	1										
Chetia flaviventris														1											
Chetia gracilis															1*										
Chetia ? sp.										3	4	1													
Ctenochromis pectoralis													1	1											
Ctenochromis scatebra													1*												
Haplochromis demeusii											1*														
Haplochromis fasciatus											3	3													

	Longest consecutive series of 1 Pt per DIS																								
	4	5	6	7	8	9	10	11	12	13	14	15	16	17	18	19	20	21	22	23	24	25	26	27	28
Haplochromis humilis													2	1											
Haplochromis moeruensis													1	2	1										
Haplochromis oligacanthus			1*																						
Lufubuchromis relictus												1*													
Orthochromis machadoi															2										
Orthochromis malagaraziensis															1	–	1								
Orthochromis polyacanthus																2									
Orthochromis stormsi														10	1										
Palaeoplex palimpsest														1*											
Pharyngochromis acuticeps												3	3												
Pseudocrenilabrus multicolor												1	3												
Pseudocrenilabrus philander													1												
Sargochromis carlottae														1	1										
Sargochromis codringtonii														2	1*										
Sargochromis giardi														1	1										
Sargochromis greenwoodi														2*											
Serranochromis angusticeps														1											
Serranochromis longimanus														1*	3										
Serranochromis macrocephalus															1										
Serranochromis meridianus															1										
Serranochromis robustus														2	3	1									
Thoracochromis albolabris													1												
Thoracochromis wingatii													2												
**Pseudocrenilabrini (riverine) totals**	**–**	**–**	**1**	**–**	**–**	**–**	**–**	**–**	**–**	**3**	**8**	**12**	**27**	**36**	**18**	**3**	**1**	**–**	**–**	**–**	**–**	**–**	**–**	**–**	**–**
**Steatocranini**																									
Steatocranus casuarius																3									
**Tilapiini**																									
Chilochromis duponti												1													
Congolapia bilineata											1	2													
Tilapia busumana										2	2														
Tilapia sparrmanii											3*	1													
**Tilapiini column totals**	**–**	**–**	**–**	**–**	**–**	**–**	**–**	**–**	**–**	**2**	**6**	**4**	**–**	**–**	**–**	**–**	**–**	**–**	**–**	**–**	**–**	**–**	**–**	**–**	**–**
**Lake Barombi Mbo**																									
**Oreochromini**																									
Konia eisentrauti												2	3												
Myaka myaka												4													
Pungu maclareni													2*												
Stomatepia mariae											1														
Stomatepia pindu											2	2													
**Oreochromini (Barombi) totals**	**–**	**–**	**–**	**–**	**–**	**–**	**–**	**–**	**–**	**–**	**3**	**8**	**5**	**–**	**–**	**–**	**–**	**–**	**–**	**–**	**–**	**–**	**–**	**–**	**–**
**Lake Fwa**																									
**Pseudocrenilabrini**																									
Cyclopharynx schwetzi													6	8											
Schwetzochromis neodon															2										
**Lake Albert**																									
**Pseudocrenilabrini**																									
Haplochromis avium													3												
Haplochromis loati													1												
**Lake Edward–George system**																									
**Pseudocrenilabrini**																									
Haplochromis akika													3*	1											
Haplochromis aquila													1*												
Haplochromis aureus														1*											
Haplochromis curvidens													1*												
Haplochromis falcatus													1*												
Haplochromis fuscus													1												
Haplochromis glaucus							1*																		
Haplochromis gracilifur													1*												
Haplochromis kimondo														1*											
Haplochromis labiatus														1											
Haplochromis latifrons													1*												
Haplochromis limax												1	3												
Haplochromis mentatus														1*											

	Longest consecutive series of 1 Pt per DIS																								
	4	5	6	7	8	9	10	11	12	13	14	15	16	17	18	19	20	21	22	23	24	25	26	27	28
Haplochromis molossus													1*												
Haplochromis pappenheimi													1*												
Haplochromis paradoxus													1*												
Haplochromis pardus													1*												
Haplochromis pelagicus															1*										
Haplochromis pharyngalis														1*											
Haplochromis quasimodo														1*											
Haplochromis relictidens													1*												
Haplochromis rex													1*												
Haplochromis simba													1*												
Haplochromis squamipinnis														1*											
Haplochromis taurinus														1*											
Schubotzia eduardiana													6	4											
**Pseudocrenilabrini (Ed–Grg) totals**	–	–	–	–	–	–	**1**	–	–	–	–	**1**	**25**	**13**	**1**	–	–	–	–	–	–	–	–	–	–
**Lake Victoria & satellites**																									
**Pseudocrenilabrini**																									
Allochromis welcommei														1											
Astatoreochromis alluaudi														1											
Haplochromis chlorochrous												3	1												
Haplochromis cryptogramma												6	4												
Haplochromis fusiformis															1										
Haplochromis latifasciatus												1	5												
Haplochromis lividus													1	–	1										
Haplochromis nubilus													1*	2											
Haplochromis plutonius												1	2	2											
Lithochromis rubripinnis													1	3											
Lithochromis xanthopteryx													3	1											
Mbipia mbipi														1											
Neochromis nigricans														1											
Paralabidochromis victoriae													1	1											
Pundamilia igneopinnis													1	–	1										
Pundamilia pundamilia													1	3											
Pyxichromis parorthostoma													1												
**Pseudocrenilabrini (Victoria) totals**	–	–	–	–	–	–	–	–	–	–	–	**11**	**22**	**16**	**3**	–	–	–	–	–	–	–	–	–	–
**Lake Kivu**																									
**Pseudocrenilabrini**																									
Haplochromis astatodon												2	3*	1											
Haplochromis paucidens													1	1											
**Lake Turkana**																									
**Pseudocrenilabrini**																									
Haplochromis rudolfianus													5	3											
Haplochromis turkanae													3												
**Lake Tanganyika**																									
**Bathybatini**																									
Bathybates fasciatus												2	3												
Bathybates ferox										2	7	1													
Bathybates graueri										4	1														
Bathybates hornii									1																
Bathybates leo											3	1	1												
Bathybates minor									3	2															
Bathybates vittatus											1														
Hemibates stenosoma													3	1											
Trematocara unimaculatum											3	2													
Trematocara zebra											4	1													
**Bathybatini column totals**	–	–	–	–	–	–	–	–	**4**	**8**	**19**	**7**	**7**	**1**	–	–	–	–	–	–	–	–	–	–	–
**Benthochromini**																									
Benthochromis tricoti																1	–	–	2	2					
**Boulengerochromini**																									
Boulengerochromis microlepis												6	1												
**Cyphotilapiini**																									
Cyphotilapia frontosa													1	–	2	2									
Cyphotilapia gibberosa																4	1								

	Longest consecutive series of 1 Pt per DIS																								
	4	5	6	7	8	9	10	11	12	13	14	15	16	17	18	19	20	21	22	23	24	25	26	27	28
**Cyprichromini**																									
Cyprichromis coloratus																		3	1	1					
Cyprichromis leptosoma															1	3	1								
Cyprichromis microlepidotus																2	3								
Cyprichromis pavo																		3	1	1					
Cyprichromis zonatus																	1	4							
Cyprichromis sp. "dwarf jumbo"																	4	1							
Cyprichromis sp. "jumbo"																	1	3	1						
Paracyprichromis brieni																	5								
Paracyprichromis nigripinnis																2	2								
Paracyprichromis sp. "brieni south"															1	1	2	1							
**Cyprichromini column totals**	**–**	**–**	**–**	**–**	**–**	**–**	**–**	**–**	**–**	**–**	**–**	**–**	**–**	**–**	**2**	**8**	**19**	**15**	**3**	**2**	**–**	**–**	**–**	**–**	**–**
**Ectodini**																									
Asprotilapia leptura																			2	2	1				
Aulonocranus dewindti																4	1								
Callochromis macrops																		4	1						
Cardiopharynx schoutedeni																		1	4						
Cunningtonia longiventralis																		1	3	1					
Cyathopharynx furcifer																	2	3							
Ectodus descampsii																1	4								
Enantiopus melanogenys																						1	4		
Grammatotria lemairii																				2	2	1			
Lestradea perspicax																			3	3					
Microdontochromis tenuidentatus																	1	1	2	–	1				
Ophthalmotilapia boops									1	–	–	–	–	–	–	–	–	2	1	1					
Xenotilapia sima																			1	3	1				
**Ectodini column totals**	**–**	**–**	**–**	**–**	**–**	**–**	**–**	**–**	**1**	**–**	**–**	**–**	**–**	**–**	**–**	**5**	**8**	**12**	**17**	**12**	**5**	**2**	**4**	**–**	**–**
**Eretmodini**																									
Eretmodus cyanostictus																1	3	1							
Eretmodus marksmithi																2	3								
Spathodus erythrodon																1	4								
Tanganicodus irsacae																	2	3							
**Eretmodini column totals**	**–**	**–**	**–**	**–**	**–**	**–**	**–**	**–**	**–**	**–**	**–**	**–**	**–**	**–**	**–**	**4**	**12**	**4**	**–**	**–**	**–**	**–**	**–**	**–**	**–**
**Lamprologini**																									
Altolamprologus calvus																2	3								
Altolamprologus compressiceps															1	1	3								
Altolamp. 'compressiceps shell'															1	4									
Chalinochromis brichardi																		2	3						
Chalinochromis cyanophleps																		2	3						
Julidochromis ornatus																	2	1	2						
Julidochromis regani																		1	4						
Lamprologus callipterus																5	4	1							
Lamprologus 'ornatipinnis congo'																1	3	1							
Lamprologus 'ornatipinnis zambia'																2	4	3							
Lamprologus speciosus														4	1										
Lepidiolamprologus kendalli																	3	2							
Lepidiolamprologus mimicus																	4	1							
Lepidiolamprologus profundicola																	2	3							
Neolamprologus tretocephalus															5										
Neolamprologus ventralis																2	3								
Telmatochromis dhonti																		5							
Telmatochromis temporalis																		2	3						
Telmatochromis vittatus																		2	2	1					
Variabilichromis moorii															2	5									
**Lamprologini column totals**	**–**	**–**	**–**	**–**	**–**	**–**	**–**	**–**	**–**	**–**	**–**	**–**	**–**	**4**	**10**	**22**	**31**	**26**	**17**	**1**	**–**	**–**	**–**	**–**	**–**
**Limnochromini**																									
Baileychromis centropomoides																4	1								
Gnathochromis permaxillaris																4	1								
Greenwoodochromis christyi													1	–	4										
Limnochromis auritus															1	4									
Reganochromis calliurus																	2	1	2						
Tangachromis dhanisi										1	–	–	–	2	1										
Trematochromis benthicola														1	4										

Table 6 (continued). Frequency distribution of counts of longest series of one pterygiophore per dorsal insertion space

	Longest consecutive series of 1 Pt per DIS																								
	4	5	6	7	8	9	10	11	12	13	14	15	16	17	18	19	20	21	22	23	24	25	26	27	28
Triglachromis otostigma																1	3	1							
**Limnochromini column totals**	**–**	**–**	**–**	**–**	**–**	**–**	**–**	**–**	**–**	**1**	**–**	**–**	**1**	**3**	**10**	**13**	**7**	**2**	**2**	**–**	**–**	**–**	**–**	**–**	**–**
**Oreochromini**																									
Oreochromis tanganicae														5											
**Perissodini**																									
Haplotaxodon microlepis																	4	1							
Perissodus microlepis													1	1	–	2	–	1							
Plecodus paradoxus																				4	1				
Xenochromis hecqui																	4	1							
**Perissodini column totals**	**–**	**–**	**–**	**–**	**–**	**–**	**–**	**–**	**–**	**–**	**–**	**–**	**1**	**1**	**–**	**2**	**8**	**3**	**–**	**4**	**1**	**–**	**–**	**–**	**–**
**Pseudocrenilabrini: Tropheina**																									
Interochromis loocki														3	2										
Jabarichromis pfefferi													1	4											
Limnotilapia dardennii																4									
Lobochilotes labiata														2	3										
Petrochromis horii															1	4									
Petrochromis polyodon														1	4										
Pseudosimochromis curvifrons															4	1									
Shuja horei													1	7											
Simochromis diagramma												1	–	–	3	1									
Tropheus annectens															5										
Tropheus brichardi														1	3	1									
Tropheus duboisi															2	3									
Tropheus moorii															3	7									
**Tropheina column totals**	**–**	**–**	**–**	**–**	**–**	**–**	**–**	**–**	**–**	**–**	**–**	**1**	**2**	**18**	**30**	**21**	**–**	**–**	**–**	**–**	**–**	**–**	**–**	**–**	**–**
**Tylochromini**																									
Tylochromis polylepis												1	4	1											
**Lake Malawi**																									
**Pseudocrenilabrini: Cyrtocarina**																									
Alticorpus mentale												1													
Alticorpus peterdaviesi													1												
Aristochromis christyi															2										
Aulonocara nyassae												1													
Aulonocara rostratum													1												
Aulonocara stonemani													1*												
Buccochromis atritaeniatus														2*											
Buccochromis heterotaenia															2*										
Buccochromis nototaenia															2*										
Buccochromis oculatus														1	2										
Buccochromis spectabilis														1											
Caprichromis orthognathus											1	–	2	–	2										
Champsochromis caeruleus															2	1	1								
Champsochromis spilorhynchus															1	1									
Cheilochromis euchilus													4*	1											
Chilotilapia rhoadesii												1	1	2											
Copadichromis jacksoni															3										
Copadichromis quadrimaculatus																1									
Corematodus taeniatus													4	3											
Ctenopharynx nitidus														11	4	1									
Ctenopharynx pictus													1												
Cyrtocara moorii													1	3	2*										
Dimidiochromis compressiceps													5	5	2										
Dimidiochromis kiwinge														1	2										
Docimodus evelynae														1*	–	1									
Docimodus johnstoni													2	–	1										
Exochochromis anagenys									1	–	–	–	2	3	1*										
Fossorochromis rostratus														1											
Hemitilapia oxyrhynchus												1	4	5											
Hemitaeniochromis urotaenia													1												
Lethrinops gossei												1	–	1*											
Lethrinops lethrinus												3													
Lethrinops polli													1*												
Lichnochromis acuticeps												1													

	Longest consecutive series of 1 Pt per DIS																								
	4	5	6	7	8	9	10	11	12	13	14	15	16	17	18	19	20	21	22	23	24	25	26	27	28
Mchenga cyclicos														1											
Mchenga inornata														2*											
Mchenga sp.													2	9	11	1									
Mylochromis formosus															2*										
Mylochromis gracilis															1*	2									
Mylochromis guentheri														2											
Mylochromis lateristriga													4												
Mylochromis spilostichus															5*	3									
Mylochromis subocularis													2												
Naevochromis chrysogaster														5*											
Nimbochromis fuscotaeniatus												1	–	1											
Nimbochromis livingstonii														5	1										
Nimbochromis venustus													1	3											
Nyassachromis leuciscus													2	4*											
Nyassachromis microcephalus														1	1*										
Nyassachromis nigritaeniatus														2	2										
Nyassachromis purpurans														1											
Otopharynx argyrosoma														2*	1										
Otopharynx decorus														1	2	3*									
Otopharynx cf. *heterodon*													2	14	3										
Otopharynx heterodon													1	5	2										
Otopharynx lithobates													1	4*											
Otopharynx ovatus														2	3*										
Otopharynx selenurus														4	2										
Otopharynx speciosus													5												
Otopharynx tetraspilus													2	6											
Otopharynx tetrastigma										1	–	1	8*	6											
Placidochromis hennydaviesae												1*													
Placidochromis johnstoni													3	1											
Placidochromis longimanus													4	14	10										
Placidochromis macrognathus											1	–	1												
Placidochromis milomo													3	1											
Protomelas annectens														1	1										
Protomelas fenestratus									2	–	–	1	16	16	1										
Protomelas insignis														2											
Protomelas kirkii												1	2*												
Protomelas labridens														1											
Protomelas ornatus												1	1*												
Protomelas spilopterus													1	4	1										
Protomelas taeniolatus														1											
Protomelas triaenodon														3											
Sciaenochromis ahli												2	7	7											
Stigmatochromis modestus													3*	2											
Stigmatochromis pholidophorus													5*												
Stigmatochromis pleurospilus													1*												
Stigmatochromis woodi												1	2	4*											
Taeniochromis holotaenia															1										
Taeniolethrinops praeorbitalis													1												
Tramitichromis brevis													1												
Trematocranus labifer													3	1											
Trematocranus microstoma													4	5											
Trematocranus placodon													3												
Tyrannochromis macrostoma														1											
Tyrannochromis nigriventer														1*											
**Cyrtocarina column totals**	**–**	**–**	**–**	**–**	**–**	**–**	**–**	**–**	**3**	**1**	**2**	**18**	**122**	**186**	**78**	**14**	**1**	**–**	**–**	**–**	**–**	**–**	**–**	**–**	**–**
**Pseudocrenilabrini: Pseudotropheina**																									
Abactochromis labrosus													2	4*											
Chindongo bellicosus															1	5									
Chindongo minutus														1											
Cyathochromis obliquidens													2	2											
Cynotilapia afra															1										
Cynotilapia sp.														2	2										
Genyochromis mento														1	9										

	Longest consecutive series of 1 Pt per DIS																								
	4	5	6	7	8	9	10	11	12	13	14	15	16	17	18	19	20	21	22	23	24	25	26	27	28
Gephyrochromis lawsi																1									
Gephyrochromis moorii															4										
Iodotropheus sprengerae														2	4										
Labeotropheus fuelleborni											1	–	–	4	8	2									
Labeotropheus trewavasae															1	2									
Labidochromis caeruleus															1										
Labidochromis freibergi															1*										
Labidochromis joanjohnsonae														1*											
Labidochromis pallidus										1	–	–	–	15	7										
Labidochromis shiranus															1										
Labidochromis textilis														1*	1										
Labidochromis vellicans														11	17	2									
Maylandia aurora															1*										
Maylandia koningsi															1										
Maylandia lanisticola														1*											
Maylandia zebra															6	2	1								
Melanochromis auratus															1	1*	4								
Melanochromis chipokae																1*									
Melanochromis loriae															3	1*									
Melanochromis melanopterus														1	4	6									
Melanochromis simulans																1									
Melanochromis cf. *vermivorus*														1	1										
Petrotilapia genalutea																1									
Petrotilapia nigra															1										
Petrotilapia tridentiger															1										
Petrotilapia sp.														1	1	2									
Pseudotropheus elegans																		1*							
Pseudotropheus interruptus															1*	1									
Pseudotropheus johannii															10	26*	–	1							
Pseudotropheus livingstonii														2*	5										
Pseudotropheus lucerna															1										
Tropheops tropheops															2	5									
**Pseudotropheina column totals**	**–**	**–**	**–**	**–**	**–**	**–**	**–**	**–**	**–**	**1**	**1**	**–**	**4**	**50**	**97**	**59**	**5**	**2**	**–**	**–**	**–**	**–**	**–**	**–**	**–**
**Pseudocrenilabrini: Rhamphochromina**																									
Diplotaxodon argenteus												2	6*	2											
Diplotaxodon ecclesi													1*												
Diplotaxodon greenwoodi												1*													
Diplotaxodon limnothrissa													6	4											
Pallidochromis tokolosh											2	2													
Rhamphochromis brevis														1	–	1*									
Rhamphochromis esox															1	–	–	1							
Rhamphochromis woodi														1											
**Rhamphochromina column totals**	**–**	**–**	**–**	**–**	**–**	**–**	**–**	**–**	**–**	**–**	**2**	**5**	**13**	**8**	**1**	**1**	**–**	**1**	**–**	**–**	**–**	**–**	**–**	**–**	**–**
**Cyrtocarina column totals**	**–**	**–**	**–**	**–**	**–**	**–**	**–**	**–**	**3**	**1**	**2**	**18**	**122**	**186**	**78**	**14**	**1**	**–**	**–**	**–**	**–**	**–**	**–**	**–**	**–**
**Pseudotropheina column totals**	–	–	–	–	–	–	–	–	–	**1**	**1**	**0**	**4**	**50**	**97**	**59**	**5**	**2**	–	–	–	–	–	–	–
**Rhamphochromina column totals**	**–**	**–**	**–**	**–**	**–**	**–**	**–**	**–**	**–**	**–**	**2**	**5**	**13**	**8**	**1**	**1**	**–**	**1**	**–**	**–**	**–**	**–**	**–**	**–**	**–**
