# Supplementary material for: African cichlid fishes: morphological data and taxonomic insights from a genus-level survey of supraneurals, pterygiophores, and vertebral counts (Ovalentaria, Blenniiformes, Cichlidae, Pseudocrenilabrinae)
Source: Biodivers Data J. 2024 Oct 18;12:e130707. doi: 10.3897/BDJ.12.e130707 (PMC11512106; doi:10.3897/BDJ.12.e130707)
Supplement: Supplementary material 8 — Table S7. [file bdj-12-e130707-s008.pdf]

Table 8. Frequency distribution of pterygiophore insertion patterns of last 4 occupied dorsal insertion spaces

	/1/1/1/1/	/1/–/1/2/	1/1/1/2/	/1/1/2/1/	/1/1/2/2/	/1/1/2/3/	/1/1/3/1/	/1/2/1/1/	/1/2/1/2/	/1/2/1/3/	/1/2/1/4/	/1/2/2/1/	/1/2/2/2/	/1/2/2/3/	/1/2/3/1/	/1/2/3/2/	/1/2/3/3/	/1/3/1/1/	/1/3/2/1/	/1/3/2/2/	/2/1/1/1/	/2/1/1/2/	/2/1/1/3/	/2/1/2/1/	/2/1/2/2/	/2/1/2/3/	/2/1/3/1/	/2/1/3/2/	/2/2/1/1/	/2/2/1/2/	/2/2/1/3/	/2/2/2/1/	/2/2/2/2/	/2/2/2/3/	/2/2/3/1/	/2/2/3/2/	/2/3/2/1/	/2/3/2/2/	?
Polycentridae																																						
Afronandus sheljuzhkoi																													1									
Monocirrhus polyacanthus																																	1	–	–	–	1	
Polycentropsis abbreviata																																	1	–	1			
Cichlidae: Etroplinae																																						
**India**																																						
Etroplus suratensis																																	1					
Pseudetroplus maculatus																								2	–	–	–	–	2	–	3							
**Madagascar**																																						
Paretroplus polyactis																															1							
Ptychochrominae																																						
**Madagascar**																																						
Katria katria											1																											
Oxylapia polli																								1															
Paratilapia polleni								1	2	–	–	5	–	–	–	–	–	–	–	–	2																	
Ptychochromis oligacanthus							1	–	–	–	1	–	–	–	–	–	–	–	–	–	–	–	2	2	–	–	–	1	2	–	2							
Ptychochromoides betsileanus											1	1	–	–	–	–	–	–	–	–	–	–	–	1															
Cichlinae																																						
**Neotropics**																																						
**Astronotini**																																						
Astronotus ocellatus																							3																
**Chaetobranchini**																																						
Chaetobranchopsis orbicularis																															1							
Chaetobranchus flavescens												3	–	–	–	–	–	–	–	–	–	–	–	–	–	–	–	–	–	–	–	1						
**Cichlasomatini**																																						
Aequidens tetramerus											1																											
Andinoacara pulcher											1																											
Bujurquina vittata																							1															
Cichlasoma dimerus											1																											
Cichlasoma taenia																							1															
Krobia guianensis												1																										
**Cichlini**																																						
Cichla ocellaris																																1						
Cichla orinocensis												1	–	–	–	–	–	–	–	–	–	–	–	–	–	–	–	–	–	–	–	2						
**Geophagini**																																						
Biotodoma cupido												1	–	–	–	–	–	–	–	–	–	–	–	–	–	–	–	–	–	–	2	1						
Geophagus crassilabris																															1							
Guianacara owroewefi											2	1	–	–	–	–	–	–	–	–	–	–	1	–	1	–	–	–	–	2	–	1						
Saxatilia frenata																													1*									
Saxatilia lepidota											1	–	–	–	–	–	–	–	–	–	–	–	–	–	–	–	–	–	–	–	1							
**Heroini**																																						
Amphilophus citrinellus																																1						
Hypselacara coryphaenoides											1	4																										
**Retroculini**																																						
Retroculus lapidifer																							1															
Pseudocrenilabrinae																																						
**Middle East**																																						
**Oreochromini**																																						
Iranocichla hormuzensis						1	–	–	–	–	1																										
Tristramella sacra																								1															

Table 8 (continued). Frequency distribution of pterygiophore insertion patterns of last 4 occupied dorsal insertion spaces

	/1/1/1/1/	/1/–/1/2/	1/1/1/2/	/1/1/2/1/	/1/1/2/2/	/1/1/2/3/	/1/1/3/1/	/1/2/1/1/	/1/2/1/2/	/1/2/1/3/	/1/2/1/4/	/1/2/2/1/	/1/2/2/2/	/1/2/2/3/	/1/2/3/1/	/1/2/3/2/	/1/2/3/3/	/1/3/1/1/	/1/3/2/1/	/1/3/2/2/	/2/1/1/1/	/2/1/1/2/	/2/1/1/3/	/2/1/2/1/	/2/1/2/2/	/2/1/2/3/	/2/1/3/1/	/2/1/3/2/	/2/2/1/1/	/2/2/1/2/	/2/2/1/3/	/2/2/2/1/	/2/2/2/2/	/2/2/2/3/	/2/2/3/1/	/2/2/3/2/	/2/3/2/1/	/2/3/2/2/	?
Tristramella simonis																								2	1	–	–	–	–	1									
**Oreochromini (M.E.) column totals**	–	–	–	–	–	–	1	–	–	–	–	1	–	–	–	–	–	–	–	–	–	–	–	2	2	–	–	–	–	1	–	–	–	–	–	–	–	–	–
**Pseudocrenilabrini**																																							
Astatotilapia flavijosephi										2	–	1	–	–	–	–	–	–	–	–	–	–	–	1															
**African riverine**																																							
**Chromidotilapiini**																																							
Benitochromis batesii																										2	–	–	–	–	–	1	3						
Benitochromis finleyi																																1							
Chromidotilapia guntheri																			1	–	–	–	–	–	–	–	–	–	–	–	1								
Chromidotilapia kingsleyae												1	2	–	1	–	–	–	–	–	–	–	–	–	–	–	–	–	–	–	–	2	2			1			
Congochromis robustus																								1*															
Congochromis squamiceps													1	–	–	–	–	–	–	–	–	–	–	1															
Divandu albimarginatus														2	–	1	–	–	–	–	–	–	–	–	–	–	–	–	–	–	–	–	–	–	1				
Limbochromis robertsi																									4	1	–	–	–	–	–	10	1	–	1				
Nanochromis nudiceps									1	–	–	–	–	–	–	–	–	–	–	–	–	–	–	–	–	–	–	–	–	–	–	–	–	–	–	–	–	–	1
Nanochromis parilus												1	–	–	–	–	–	–	–	–	–	–	–	–	–	–	–	–	1										
Parananochromis gabonicus													1*																										
Parananochromis longirostris													1	–	–	–	–	–	1	–	–	–	–	–	2	1	1	–	–	–	–	1	4						
Pelmatochromis buettikoferi													2	–	–	–	–	–	–	–	–	–	–	–	–	–	–	–	–	–	–	1							
Pelmatochromis ocellifer																																		1*					
Pelvicachromis pulcher																																1							
Pelvicachromis taeniatus													1*																										
Pterochromis congicus																										1	–	–	–	–	–	–	1*						
Teleogramma brichardi																									4	–	–	–	–	–	–	8	1	–	1				
Thysochromis ansorgii												2	1	–	–	–	–	–	–	–	–	–	–	–	–	–	–	–	–	–	–	1							
Wallaceochromis humilis												1	1	–	–	–	–	–	–	–	–	–	–	–	1	–	–	–	–	1	–	–	1						
**Chromidotilapiini column totals**	–	–	–	–	–	–	–	–	1	–	–	5	10	2	1	1	–	–	2	–	–	–	–	2	11	5	1	–	1	1	1	26	13	1	3	1	–	–	1
**Coelotilapiini**																																							
Coelotilapia joka														1																									
**Coptodonini**																																							
Coptodon discolor																																	1	–	1				
Coptodon tholloni															1																								
Coptodon zillii													3	1	1	1	–	–	–	–	–	–	–	–	2	1	–	–	–	–	–	5	6	1	1				
**Coptodonini column totals**	–	–	–	–	–	–	–	–	–	–	–	–	3	1	2	1	–	–	–	–	–	–	–	–	2	1	–	–	–	–	–	5	7	1	2	–	–	–	–
**Etiini**																																							
Etia nguti																																	6						
**Gobiocichlini**																																							
Gobiocichla ethelwynnae									3	–	–	1	–	–	–	–	–	–	–	–	–	–	–	6	1														
Gobiocichla wonderi				3	2	–	–	8	1																														
Paragobiocichla irvinei										1	–	1	–	–	–	–	–	–	–	–	–	–	–	1	1														
**Gobiocichlini column totals**	–	–	–	3	2	–	–	8	4	1	–	2	–	–	–	–	–	–	–	–	–	–	–	7	2	–	–	–	–	–	–	–	–	–	–	–	–	–	–
**Hemichromini**																																							
Anomalochromis thomasi													2	–	–	–	–	–	–	–	–	–	–	–	–	–	–	–	–	1	–	–	–	1					
Hemichromis elongatus																									2	–	–	–	–	–	–	2							
Hemichromis fasciatus																																1							
Rubricatochromis bimaculatus													1	–	–	–	–	–	–	–	–	–	–	–	1	–	–	–	–	1	–	2	1						
Rubricatochromis letourneuxi					1	–	–	–	–	–	–	–	–	–	–	–	–	–	–	–	–	–	–	–	–	–	–	–	–	–	–	1	2						
**Hemichromini column totals**	–	–	–	–	1	–	–	–	–	–	–	–	3	–	–	–	–	–	–	–	–	–	–	–	3	–	–	–	–	2	–	6	3	1	–	–	–	–	–
**Heterochromini**																																							
Heterochromis multidens																	2	–	–	1	–	–	–	–	–	–	–	–	–	–	–	–	4	2	1	1			
**Heterotilapiini**																																							
Heterotilapia buettikoferi																																		2	1	–	1		

Table 8 (continued). Frequency distribution of pterygiophore insertion patterns of last 4 occupied dorsal insertion spaces

	|1/1/1/1|	|1/–/1/2|	1/1/1/2|	|1/1/2/1|	|1/1/2/2|	|1/1/2/3|	|1/1/3/1|	|1/2/1/1|	|1/2/1/2|	|1/2/1/3|	|1/2/1/4|	|1/2/2/1|	|1/2/2/2|	|1/2/2/3|	|1/2/3/1|	|1/2/3/2|	|1/2/3/3|	|1/3/1/1|	|1/3/2/1|	|1/3/2/2|	|2/1/1/1|	|2/1/1/2|	|2/1/1/3|	|2/1/2/1|	|2/1/2/2|	|2/1/2/3|	|2/1/3/1|	|2/1/3/2|	|2/2/1/1|	|2/2/1/2|	|2/2/1/3|	|2/2/2/1|	|2/2/2/2|	|2/2/2/3|	|2/2/3/1|	|2/2/3/2|	|2/3/2/1|	|2/3/2/2|	?
**Oreochromini**																																							
Danakilia franchettii													1	1	–	–	–	–	–	–	–	–	–	–	2	–	3	–	–	–	–	7							
Oreochromis jipe													3	–	–	–	–	–	–	–	–	–	–	–	–	–	–	–	–	–	–	1							
Oreochromis niloticus												1	–	–	–	–	–	–	–	–	–	–	–	–	–	–	–	–	–	–	–	–	2						
Oreochromis shiranus													3	–	1	–	–	–	–	–	–	–	–	–	–	1	–	–	–	–	–	1							
Sarotherodon galilaeus																1	–	–	–	–	–	–	–	–	–	–	–	–	–	–	–	–	–	–	–	–	1		
Sarotherodon melanotheron													1	–	–	–	–	–	–	–	–	–	–	–	–	1	–	–	–	–	–	1	2	–	1				
**Oreochromini (riverine) totals**	**–**	**–**	**–**	**–**	**–**	**–**	**–**	**–**	**–**	**–**	**–**	**1**	**8**	**1**	**1**	**1**	**–**	**–**	**–**	**–**	**–**	**–**	**–**	**–**	**2**	**2**	**3**	**–**	**–**	**–**	**–**	**10**	**4**	**–**	**1**	**–**	**1**	**–**	**–**
**Pelmatolapiini**																																							
Pelmatolapia mariae																																		5	–	1			
**Pseudocrenilabrini**																																							
Astatotilapia bloyeti					3	–	–	–	2	–	–	2	–	–	–	–	–	–	–	–	–	–	–	1															
Astatotilapia burtoni						1	–	–	–	–	–	–	4	–	–	–	–	–	–	–	–	–	–	1															
Astatotilapia calliptera					1	–	–	–	2	1	–	4	–	–	–	–	–	–	–	–	–	–	–	1*															
Astatotilapia swynnertoni					1	–	–	–	1	1																													
Chetia flaviventris																													1										
Chetia gracilis																																1*							
Chetia ? sp.												1	–	–	–	–	–	–	–	–	–	–	–	–	–	1	–	1	–	–	–	3	2						
Ctenochromis pectoralis												1	–	–	–	–	–	–	–	–	–	–	–	1															
Ctenochromis scatebra																								1*															
Haplochromis demeusii																																	1*						
Haplochromis fasciatus												1	–	–	–	–	–	–	1	–	–	–	–	–	–	1	–	–	–	–	–	1	2						
Haplochromis humilis													1	–	–	–	–	–	–	–	–	–	–	–	2														
Haplochromis moeruensis					1	–	–	–	–	1	–	–	1	–	–	–	–	–	–	–	–	–	–	1															
Haplochromis oligacanthus										1																													
Lufubuchromis relictus																									1*														
Orthochromis machadoi												1	1																										
Orthochromis malagaraziensis					1	–	–	–	–	–	–	–	–	–	–	–	–	–	–	–	–	1																	
Orthochromis polyacanthus									1	–	–	–	–	–	–	–	–	–	–	–	–	–	–	1															
Orthochromis stormsi													3	–	–	–	–	–	–	–	–	–	–	4	3	–	–	–	1										
Palaeoplex palimpsest										1*																													
Pharyngochromis acuticeps													1	–	–	–	–	–	–	–	–	–	–	2	1	–	2												
Pseudocrenilabrus multicolor												2	2																										
Pseudocrenilabrus philander												1																											
Sargochromis carlottae																									1	–	–	–	–	–	–	1							
Sargochromis codringtonii																										1*	–	–	–	–	–	2							
Sargochromis giardi																														1	–	–	1						
Sargochromis greenwoodi												1*	1																										
Serranochromis angusticeps																									1														
Serranochromis longimanus																								1	1	–	–	–	–	1*									
Serranochromis macrocephalus												1																											
Serranochromis meridianus																									1														
Serranochromis robustus												1	1	–	–	–	–	–	–	–	–	–	–	4															
Thoracochromis albolabris																									1														
Thoracochromis wingatii					1	–	–	–	–	–	–	–	–	–	–	–	–	–	–	–	1																		
**Pseudocrenilabrini (riverine) totals**	**–**	**–**	**–**	**–**	**8**	**1**	**–**	**–**	**6**	**5**	**–**	**16**	**15**	**–**	**–**	**–**	**–**	**–**	**1**	**–**	**1**	**1**	**–**	**18**	**13**	**3**	**2**	**1**	**2**	**2**	**–**	**8**	**6**	**–**	**–**	**–**	**–**	**–**	**–**
**Steatocranini**																																							
Steatocranus casuarius																								2	1														
**Tilapiini**																																							
Chilochromis duponti																																1							
Congolapia bilineata												1	–	–	–	–	–	–	–	–	–	–	–	–	–	–	–	1	–	–	–	–	1						
Tilapia busumana																																1	3						

Table 8 (continued). Frequency distribution of pterygiophore insertion patterns of last 4 occupied dorsal insertion spaces

	/1/1/1/1/	/1/–/1/2/	1/1/1/2/	/1/1/2/1/	/1/1/2/2/	/1/1/2/3/	/1/1/3/1/	/1/2/1/1/	/1/2/1/2/	/1/2/1/3/	/1/2/1/4/	/1/2/2/1/	/1/2/2/2/	/1/2/2/3/	/1/2/3/1/	/1/2/3/2/	/1/2/3/3/	/1/3/1/1/	/1/3/2/1/	/1/3/2/2/	/2/1/1/1/	/2/1/1/2/	/2/1/1/3/	/2/1/2/1/	/2/1/2/2/	/2/1/2/3/	/2/1/3/1/	/2/1/3/2/	/2/2/1/1/	/2/2/1/2/	/2/2/1/3/	/2/2/2/1/	/2/2/2/2/	/2/2/2/3/	/2/2/3/1/	/2/2/3/2/	/2/3/2/1/	/2/3/2/2/	?
Tilapia sparrmanii												2	–	–	–	–	–	–	–	–	–	–	–	–	–	–	1	–	–	–	–	1*							
**Tilapiini column totals**	–	–	–	–	–	–	–	–	–	–	–	**3**	–	–	–	–	–	–	–	–	–	–	–	–	–	–	**1**	**1**	–	–	–	**3**	**4**	–	–	–	–	–	–
**Lake Barombi Mbo**																																							
**Oreochromini**																																							
Konia eisentrauti												1	1	–	–	–	–	–	–	–	–	–	–	–	–	–	–	–	–	–	–	3							
Myaka myaka																			1	–	–	–	–	–	–	–	–	1	–	–	–	–	1						
Pungu maclareni																									1*	–	–	–	–	–	–	1							
Stomatepia mariae																																	1						
Stomatepia pindu														1	–	–	–	–	–	–	–	–	–	–	–	–	–	1	–	–	–	1	1						
**Oreochromini (Barombi) totals**	–	–	–	–	–	–	–	–	–	–	–	**1**	**1**	**1**	–	–	–	–	**1**	–	–	–	–	–	**1**	–	–	**2**	–	–	–	**5**	**3**	–	–	–	–	–	–
**Lake Fwa**																																							
**Pseudocrenilabrini**																																							
Cyclopharynx schwetzi										3	–	–	6	–	1	–	–	–	–	–	–	–	–	2	1	–	1												
Schwetzochromis neodon																									2														
**Lake Albert**																																							
**Pseudocrenilabrini**																																							
Haplochromis avium				1	1	–	–	–	1																														
Haplochromis loati					1																																		
**Lake Edward–George system**																																							
**Pseudocrenilabrini**																																							
Haplochromis akika									1	–	–	2*	–	–	–	–	–	–	–	–	–	–	–	1															
Haplochromis aquila										1*																													
Haplochromis aureus												1*																											
Haplochromis curvidens										1*																													
Haplochromis falcatus												1*																											
Haplochromis fuscus																								1															
Haplochromis glaucus					1*																																		
Haplochromis gracilifur									1*																														
Haplochromis kimondo									1*																														
Haplochromis labiatus									1																														
Haplochromis latifrons												1*																											
Haplochromis limax												1	1	–	–	–	–	–	–	–	–	–	–	2															
Haplochromis mentatus									1*																														
Haplochromis molossus									1*																														
Haplochromis pappenheimi									1*																														
Haplochromis paradoxus												1*																											
Haplochromis pardus																								1*															
Haplochromis pelagicus																								1*															
Haplochromis pharyngalis									1*																														
Haplochromis quasimodo												1*																											
Haplochromis relictidens												1*																											
Haplochromis rex																								1*															
Haplochromis simba																								1*															
Haplochromis squamipinnis												1*																											
Haplochromis taurinus									1*																														
Schubotzia eduardiana					1	–	–	–	4	–	–	3	–	–	–	–	–	–	–	–	–	–	–	2															
**Pseudocrenilabrini (Ed–Grg) totals**	–	–	–	–	**2**	–	–	–	**13**	**2**	–	**13**	**1**	–	–	–	–	–	–	–	–	–	–	**10**	–	–	–	–	–	–	–	–	–	–	–	–	–	–	–
**Lake Victoria & satellites**																																							
**Pseudocrenilabrini**																																							
Allochromis welcommei												1																											
Astatoreochromis alluaudi										1																													
Haplochromis chlorochrous									2	–	–	1	–	–	–	–	–	–	–	–	–	1																	

Table 8 (continued). Frequency distribution of pterygiophore insertion patterns of last 4 occupied dorsal insertion spaces

	/1/1/1/1/	/1/–/1/2/	1/1/1/2/	/1/1/2/1/	/1/1/2/2/	/1/1/2/3/	/1/1/3/1/	/1/2/1/1/	/1/2/1/2/	/1/2/1/3/	/1/2/1/4/	/1/2/2/1/	/1/2/2/2/	/1/2/2/3/	/1/2/3/1/	/1/2/3/2/	/1/2/3/3/	/1/3/1/1/	/1/3/2/1/	/1/3/2/2/	/2/1/1/1/	/2/1/1/2/	/2/1/1/3/	/2/1/2/1/	/2/1/2/2/	/2/1/2/3/	/2/1/3/1/	/2/1/3/2/	/2/2/1/1/	/2/2/1/2/	/2/2/1/3/	/2/2/2/1/	/2/2/2/2/	/2/2/2/3/	/2/2/3/1/	/2/2/3/2/	/2/3/2/1/	/2/3/2/2/	?
Haplochromis cryptogramma					2	–	–	1	1	–	–	3	–	–	–	–	–	–	–	–	–	–	–	3															
Haplochromis fusiformis												1																											
Haplochromis latifasciatus									2	–	–	–	–	–	–	–	–	–	–	–	–	–	–	4															
Haplochromis lividus					1	–	–	–	–	–	–	–	–	–	–	–	–	–	–	–	–	–	–	1															
Haplochromis nubilus									1	–	–	1	–	–	–	–	–	–	–	–	–	–	–	1*															
Haplochromis plutonius						2	–	–	1	–	–	1	–	–	–	–	–	–	–	–	–	–	–	1															
Lithochromis rubripinnis									3	–	–	1																											
Lithochromis xanthopteryx									1	–	–	–	–	–	–	–	–	–	–	–	–	–	–	3															
Mbipia mbipi												1																											
Neochromis nigricans																								1															
Paralabidochromis victoriae									2																														
Pundamilia igneopinnis										1	–	1																											
Pundamilia pundamilia									2	–	–	2																											
Pyxichromis parorthostoma													1																										
**Pseudocrenilabrini (Victoria) totals**	**–**	**–**	**–**	**–**	**3**	**2**	**–**	**1**	**15**	**2**	**–**	**13**	**1**	**–**	**–**	**–**	**–**	**–**	**–**	**–**	**–**	**1**	**–**	**14**	**–**	**–**	**–**	**–**	**–**	**–**	**–**	**–**	**–**	**–**	**–**	**–**	**–**	**–**	**–**
**Lake Kivu**																																							
**Pseudocrenilabrini**																																							
Haplochromis astatodon					1	–	–	–	2*	1	–	1	–	–	–	–	–	–	–	–	–	–	–	1															
Haplochromis paucidens												1	–	–	–	–	–	–	–	–	–	–	–	1															
**Lake Turkana**																																							
**Pseudocrenilabrini**																																							
Haplochromis rudolfianus					2	–	–	–	3	1	–	2																											
Haplochromis turkanae					2	–	–	1																															
**Lake Tanganyika**																																							
**Bathybatini**																																							
Bathybates fasciatus													1	–	–	–	–	–	–	–	–	–	–	–	2	–	–	–	1	1									
Bathybates ferox									1	–	–	1	–	–	–	–	–	–	–	–	–	–	–	2	3	–	–	–	–	1									
Bathybates graueri								2	–	–	–	–	–	–	–	–	–	–	–	–	–	–	–	–	1	–	–	–	2										
Bathybates hornii																																1							
Bathybates leo								1	–	–	–	–	–	–	–	–	–	–	–	–	–	–	–	–	3	–	–	–	1										
Bathybates minor												2	–	–	–	–	–	–	–	–	–	–	–	3															
Bathybates vittatus																														1									
Hemibates stenosoma												3	–	–	–	–	–	–	–	–	–	–	–	–	–	–	–	–	–	–	–	–	1						
Trematocara unimaculatum								2	3																														
Trematocara zebra			1	1	–	–	–	1	1																														
**Bathybatini column totals**	**–**	**–**	**1**	**1**	**–**	**–**	**–**	**6**	**5**	**–**	**–**	**6**	**1**	**–**	**–**	**–**	**–**	**–**	**–**	**–**	**–**	**–**	**–**	**5**	**9**	**–**	**–**	**–**	**4**	**3**	**–**	**1**	**1**	**–**	**–**	**–**	**–**	**–**	**–**
**Benthochromini**																																							
Benthochromis tricoti				3	–	–	–	–	–	–	–	1	–	–	–	–	–	–	–	–	1																		
**Boulengerochromini**																																							
Boulengerochromis microlepis														1	–	–	–	–	–	–	–	–	–	–	–	1	–	–	–	–	–	2	3						
**Cyphotilapiini**																																							
Cyphotilapia frontosa												1	1	–	–	–	–	–	–	–	–	–	–	2	1														
Cyphotilapia gibberosa									1	1	–	2	–	–	–	–	–	–	–	–	–	–	–	1															
**Cyprichromini**																																							
Cyprichromis coloratus				1	–	–	–	3	1																														
Cyprichromis leptosoma								1	1	–	–	–	–	–	–	–	–	–	–	–	2	1																	
Cyprichromis microlepidotus					1	–	–	–	3	–	–	–	–	–	–	–	–	–	–	–	–	1																	
Cyprichromis pavo				1	–	–	–	–	1	–	–	–	–	–	–	–	–	–	–	–	3																		
Cyprichromis zonatus								2	3																														
Cyprichromis sp. "dwarf jumbo"								1	1	–	–	–	–	–	–	–	–	–	–	–	2	1																	
Cyprichromis sp. "jumbo"								2	–	–	–	–	–	–	–	–	–	–	–	–	3																		
Paracyprichromis brieni								1	3	–	–	–	–	–	–	–	–	–	–	–	–	–	–	1															

Table 8 (continued). Frequency distribution of pterygiophore insertion patterns of last 4 occupied dorsal insertion spaces

	/1/1/1/1/	/1/–/1/2/	1/1/1/2/	/1/1/2/1/	/1/1/2/2/	/1/1/2/3/	/1/1/3/1/	/1/2/1/1/	/1/2/1/2/	/1/2/1/3/	/1/2/1/4/	/1/2/2/1/	/1/2/2/2/	/1/2/2/3/	/1/2/3/1/	/1/2/3/2/	/1/2/3/3/	/1/3/1/1/	/1/3/2/1/	/1/3/2/2/	/2/1/1/1/	/2/1/1/2/	/2/1/1/3/	/2/1/2/1/	/2/1/2/2/	/2/1/2/3/	/2/1/3/1/	/2/1/3/2/	/2/2/1/1/	/2/2/1/2/	/2/2/1/3/	/2/2/2/1/	/2/2/2/2/	/2/2/2/3/	/2/2/3/1/	/2/2/3/2/	/2/3/2/1/	/2/3/2/2/	?
Paracyprichromis nigripinnis								1	2	–	–	–	–	–	–	–	–	–	–	–	1																		
Paracyprichromis sp. "brieni south"					1	–	–	–	1	–	–	–	–	–	–	–	–	–	–	–	–	1																	
**Cyprichromini column totals**	**–**	**–**	**–**	**2**	**2**	**–**	**–**	**11**	**16**	**–**	**–**	**–**	**–**	**–**	**–**	**–**	**–**	**–**	**–**	**–**	**11**	**4**	**–**	**1**	**–**	**–**	**–**	**–**	**–**	**–**	**–**	**–**	**–**	**–**	**–**	**–**	**–**	**–**	**–**
**Ectodini**																																							
Asprotilapia leptura			2	3																																			
Aulonocranus dewindti			1	3	–	–	–	1																															
Callochromis macrops			1	4																																			
Cardiopharynx schoutedeni			1	4																																			
Cunningtonia longiventralis			3	2																																			
Cyathopharynx furcifer				3	1	–	–	1																															
Ectodus descampsii				3	–	–	–	2																															
Enantiopus melanogenys			3	2																																			
Grammatotria lemairii	1	–	4																																				
Lestradea perspicax				5	1																																		
Microdontochromis tenuidentatus	1	1	3																																				
Ophthalmotilapia boops			1	2	1	–	–	1																															
Xenotilapia sima	1	–	3	1																																			
**Ectodini column totals**	**3**	**1**	**22**	**32**	**3**	**–**	**–**	**5**	**–**	**–**	**–**	**–**	**–**	**–**	**–**	**–**	**–**	**–**	**–**	**–**	**–**	**–**	**–**	**–**	**–**	**–**	**–**	**–**	**–**	**–**	**–**	**–**	**–**	**–**	**–**	**–**	**–**	**–**	**–**
**Eretmodini**																																							
Eretmodus cyanostictus								2	2	–	–	–	–	–	–	–	–	–	–	–	–	1																	
Eretmodus marksmithi									3	–	–	–	–	–	–	–	–	–	–	–	–	2																	
Spathodus erythrodon									4	–	–	–	–	–	–	–	–	–	–	–	–	1																	
Tanganicodus irsacae								3	2																														
**Eretmodini column totals**	**–**	**–**	**–**	**–**	**–**	**–**	**–**	**5**	**11**	**–**	**–**	**–**	**–**	**–**	**–**	**–**	**–**	**–**	**–**	**–**	**–**	**4**	**–**	**–**	**–**	**–**	**–**	**–**	**–**	**–**	**–**	**–**	**–**	**–**	**–**	**–**	**–**	**–**	**–**
**Lamprologini**																																							
Altolamprologus calvus									1	–	–	3	–	–	–	–	–	–	–	–	–	–	–	1															
Altolamprologus compressiceps					4	–	–	–	1																														
Altolamp. 'compressiceps shell'					4	–	–	–	1																														
Chalinochromis brichardi								1	4																														
Chalinochromis cyanophleps					2	–	–	–	3																														
Julidochromis ornatus								3	1	–	–	–	–	–	–	–	–	–	–	–	–	1																	
Julidochromis regani				1	3	–	–	–	1																														
Lamprologus callipterus				6	–	–	–	–	4																														
Lamprologus 'ornatipinnis congo'			1	3	–	–	–	1																															
Lamprologus 'ornatipinnis zambia'			4	3	1	–	–	1																															
Lamprologus speciosus				1	1	–	–	3																															
Lepidiolamprologus kendalli									2	–	–	1	–	–	–	–	–	–	–	–	–	1	–	1															
Lepidiolamprologus mimicus												1	–	–	–	–	–	–	–	–	–	–	–	4															
Lepidiolamprologus profundicola									2	–	–	–	–	–	–	–	–	–	–	–	–	2	–	1															
Neolamprologus tretocephalus								1	–	–	–	–	–	–	–	–	–	–	–	–	–	–	–	1	3														
Neolamprologus ventralis				2	2	–	–	1																															
Telmatochromis dhonti				4	–	–	–	1																															
Telmatochromis temporalis				2	1	–	–	2																															
Telmatochromis vittatus				1	1	–	–	2	1																														
Variabilichromis moorii					1	–	–	–	1	–	–	–	–	–	–	–	–	–	–	–	–	–	–	–	5														
**Lamprologini column totals**	**–**	**–**	**5**	**23**	**20**	**–**	**–**	**16**	**22**	**–**	**–**	**5**	**–**	**–**	**–**	**–**	**–**	**–**	**–**	**–**	**–**	**4**	**–**	**8**	**8**	**–**	**–**	**–**	**–**	**–**	**–**	**–**	**–**	**–**	**–**	**–**	**–**	**–**	**–**
**Limnochromini**																																							
Baileychromis centropomoides				4	–	–	–	1																															
Gnathochromis permaxillaris					2	–	–	–	3																														
Greenwoodochromis christyi					1	–	–	1	1	–	–	2																											
Limnochromis auritus					2	–	–	–	3																														
Reganochromis calliurus				1	–	–	–	3	1																														

Table 8 (continued). Frequency distribution of pterygiophore insertion patterns of last 4 occupied dorsal insertion spaces

	/1/1/1/1/	/1/–/1/2/	1/1/1/2/	/1/1/2/1/	/1/1/2/2/	/1/1/2/3/	/1/1/3/1/	/1/2/1/1/	/1/2/1/2/	/1/2/1/3/	/1/2/1/4/	/1/2/2/1/	/1/2/2/2/	/1/2/2/3/	/1/2/3/1/	/1/2/3/2/	/1/2/3/3/	/1/3/1/1/	/1/3/2/1/	/1/3/2/2/	/2/1/1/1/	/2/1/1/2/	/2/1/1/3/	/2/1/2/1/	/2/1/2/2/	/2/1/2/3/	/2/1/3/1/	/2/1/3/2/	/2/2/1/1/	/2/2/1/2/	/2/2/1/3/	/2/2/2/1/	/2/2/2/2/	/2/2/2/3/	/2/2/3/1/	/2/2/3/2/	/2/3/2/1/	/2/3/2/2/	?
Tangachromis dhanisi				2	1	–	–	1																															
Trematochromis benthicola												3	2																										
Triglachromis otostigma	1	–	3	1																																			
**Limnochromini column totals**	**1**	**–**	**3**	**8**	**6**	**–**	**–**	**6**	**8**	**–**	**–**	**5**	**2**	**–**	**–**	**–**	**–**	**–**	**–**	**–**	**–**	**–**	**–**	**–**	**–**	**–**	**–**	**–**	**–**	**–**	**–**	**–**	**–**	**–**	**–**	**–**	**–**	**–**	**–**
**Oreochromini**																																							
Oreochromis tanganicae																1	–	–	–	–	–	–	–	–	–	–	1	–	–	–	–	2	1						
**Perissodini**																																							
Haplotaxodon microlepis																								3	2														
Perissodus microlepis									1	–	–	–	1	–	–	–	–	–	–	–	–	–	–	1	2														
Plecodus paradoxus																								5															
Xenochromis hecqui					1	–	–	–	2	–	–	1	–	–	–	–	–	–	–	–	–	–	–	1															
**Perissodini column totals**	**–**	**–**	**–**	**–**	**1**	**–**	**–**	**–**	**3**	**–**	**–**	**1**	**1**	**–**	**–**	**–**	**–**	**–**	**–**	**–**	**–**	**–**	**–**	**10**	**4**	**–**	**–**	**–**	**–**	**–**	**–**	**–**	**–**	**–**	**–**	**–**	**–**	**–**	**–**
**Pseudocrenilabrini: Tropheina**																																							
Interochromis loocki													1	–	–	–	–	–	–	–	–	–	–	–	4														
Jabarichromis pfefferi										1	–	–	–	–	–	–	–	–	–	–	–	1	–	3															
Limnotilapia dardennii																								3	1														
Lobochilotes labiata												2	–	–	–	–	–	–	–	–	–	–	–	–	–	–	–	–	–	3									
Petrochromis horii													1	–	–	–	–	–	–	–	–	–	–	–	4														
Petrochromis polyodon																								4	1														
Pseudosimochromis curvifrons													2	–	–	–	–	–	–	–	–	–	–	3															
Shuja horei									4	–	–	3	–	–	–	–	–	–	–	–	–	–	–	1															
Simochromis diagramma												1	–	–	–	–	–	–	–	–	–	–	–	1	3														
Tropheus annectens				3	–	–	–	1	–	–	–	–	–	–	–	–	–	–	–	–	–	–	1																
Tropheus brichardi									1	–	–	1	–	–	–	–	–	–	–	–	–	–	–	3															
Tropheus duboisi									3	–	–	–	–	–	–	–	–	–	–	–	–	–	–	2															
Tropheus moorii				1	–	–	–	–	4	–	–	–	–	–	–	–	–	–	–	–	–	1	–	4															
**Tropheina column totals**	**–**	**–**	**–**	**4**	**–**	**–**	**–**	**1**	**12**	**1**	**–**	**7**	**4**	**–**	**–**	**–**	**–**	**–**	**–**	**–**	**–**	**2**	**1**	**24**	**13**	**–**	**–**	**–**	**–**	**3**	**–**	**–**	**–**	**–**	**–**	**–**	**–**	**–**	**–**
**Tylochromini**																																							
Tylochromis polylepis														2	–	–	–	–	–	–	–	–	–	–	–	–	1	1	–	–	–	1	1						
**Lake Malawi**																																							
**Pseudocrenilabrini: Cyrtocarina**																																							
Alticorpus mentale																									1														
Alticorpus peterdaviesi										1																													
Aristochromis christyi														1	–	–	–	–	–	–	–	–	–	–	–	–	–	–	–	–	–	1							
Aulonocara nyassae												1																											
Aulonocara rostratum																									1														
Aulonocara stonemani																																1*							
Buccochromis atritaeniatus										1	1*																												
Buccochromis heterotaenia																																2*							
Buccochromis nototaenia												1*	–	–	–	–	–	–	–	–	–	–	–	–	1														
Buccochromis oculatus													1	–	–	–	–	–	–	–	–	–	–	–	–	–	2												
Buccochromis spectabilis												1																											
Caprichromis orthognathus												1	–	–	–	–	–	–	–	–	–	–	–	–	2	–	1	–	–	–	–	1							
Champsochromis caeruleus												2	1	–	–	–	–	–	–	–	–	–	–	–	–	1													
Champsochromis spilorhynchus												1	–	–	–	–	–	–	–	–	–	–	–	–	1														
Cheilochromis euchilus										1	–	–	–	–	–	–	–	–	–	–	–	–	–	1*	1	–	1	–	–	–	–	1							
Chilotilapia rhoadesii							1	–	–	–	–	–	1	–	–	–	–	–	–	–	–	–	–	–	2														
Copadichromis jacksoni													2	–	–	–	–	–	–	–	–	–	–	–	–	–	–	–	–	–	–	1							
Copadichromis quadrimaculatus										1																													
Corematodus taeniatus													2	–	–	–	–	–	–	–	–	–	–	–	3	–	1	–	–	–	–	1							
Ctenopharynx nitidus												4	1	–	–	–	–	–	–	–	–	–	–	–	6	4	–	–	–	–	–	1							
Ctenopharynx pictus												1																											

Table 8 (continued). Frequency distribution of pterygiophore insertion patterns of last 4 occupied dorsal insertion spaces

	/1/1/1/1/	/1/–/1/2/	1/1/1/2/	/1/1/2/1/	/1/1/2/2/	/1/1/2/3/	/1/1/3/1/	/1/2/1/1/	/1/2/1/2/	/1/2/1/3/	/1/2/1/4/	/1/2/2/1/	/1/2/2/2/	/1/2/2/3/	/1/2/3/1/	/1/2/3/2/	/1/2/3/3/	/1/3/1/1/	/1/3/2/1/	/1/3/2/2/	/2/1/1/1/	/2/1/1/2/	/2/1/1/3/	/2/1/2/1/	/2/1/2/2/	/2/1/2/3/	/2/1/3/1/	/2/1/3/2/	/2/2/1/1/	/2/2/1/2/	/2/2/1/3/	/2/2/2/1/	/2/2/2/2/	/2/2/2/3/	/2/2/3/1/	/2/2/3/2/	/2/3/2/1/	/2/3/2/2/	?
Cyrtocara moorii								1	–	–	–	–	–	–	–	–	–	–	–	–	–	–	–	2	3*														
Dimidiochromis compressiceps					1	–	–	–	–	1	–	1	3	–	–	–	–	–	–	–	–	–	–	–	6														
Dimidiochromis kiwinge												1	–	–	–	–	–	–	–	–	–	–	–	–	2														
Docimodus evelynae																									1*	–	–	–	–	1									
Docimodus johnstoni												1	–	–	–	–	–	–	–	–	–	–	–	1	1														
Exochochromis anagenys												1													2	2						1*	1						
Fossorochromis rostratus																									1														
Hemitaeniochromis urotaenia																									1														
Hemitilapia oxyrhynchus										1	–	4	–	–	–	–	–	–	–	–	–	–	–	–	4	1													
Lethrinops gossei							1	–	–	–	–	–	1*																										
Lethrinops lethrinus					1	–	–	–	–	–	–	–	–	–	–	–	–	–	–	–	–	–	–	–	2														
Lethrinops polli																											1*												
Lichnochromis acuticeps									1																														
Mchenga cyclicos																									1														
Mchenga inornata						1*	–	–	1																														
Mchenga sp.					1	–	1	–	1	–	–	5	2	–	–	–	–	–	–	–	–	–	–	–	11	–	2												
Mylochromis formosus					1	–	–	–	–	–	–	1*																											
Mylochromis gracilis							1	–	–	–	–	2*																											
Mylochromis guentheri																									1	–	–	–	–	–	–	1							
Mylochromis lateristriga												3	–	–	–	–	–	–	–	–	–	–	–	–	–	1													
Mylochromis spilostichus									2	–	–	4*	1	–	–	–	–	–	–	–	–	–	–	–	1														
Mylochromis subocularis												1	–	–	–	–	–	–	–	–	–	–	–	–	1														
Naevochromis chrysogaster										1	–	–	2*	–	–	–	–	–	–	–	–	–	–	–	2														
Nimbochromis fuscotaeniatus																									2														
Nimbochromis livingstonii										2	–	–	3	–	–	–	–	–	–	–	–	–	–	–	–	–	1												
Nimbochromis venustus													2	–	–	–	–	–	–	–	–	–	–	–	2														
Nyassachromis leuciscus					1	–	–	–	2	–	–	1*	–	–	–	–	–	–	–	–	–	–	–	–	1	–	1												
Nyassachromis microcephalus												2*																											
Nyassachromis nigritaeniatus											1	–	–	–	–	–	–	–	–	–	–	–	–	–	1	2													
Nyassachromis purpurans												1																											
Otopharynx argyrosoma																									2*	1													
Otopharynx decorus					1	–	–	–	–	1	–	1	–	–	–	–	–	–	–	–	–	–	–	–	2	1*													
Otopharynx cf. *heterodon*					1	–	–	–	–	–	–	–	2	1	–	–	–	–	–	–	–	–	–	–	9	4	2												
Otopharynx heterodon												1	1	–	–	–	–	–	–	–	–	–	–	–	4	1	–	1											
Otopharynx lithobates									1	–	–	–	–	–	–	–	–	–	–	–	–	–	–	–	4*														
Otopharynx ovatus												1	2	–	–	–	–	–	–	–	–	–	–	–	–	–	2*												
Otopharynx selenurus					1	–	–	–	–	–	–	–	–	–	–	–	–	–	–	–	–	–	1	–	3	–	–	–	–	–	1								
Otopharynx speciosus												2	–	–	–	–	–	–	–	–	–	–	–	–	3														
Otopharynx tetraspilus													1	–	–	–	–	–	–	–	–	–	–	–	6	–	–	–	–	1									
Otopharynx tetrastigma										2	–	1	–	–	–	–	–	–	–	–	–	–	–	5	5*	1	1	–	–	1									
Placidochromis hennydaviesae																									1*														
Placidochromis johnstoni						1	–	–	–	–	–	–	–	–	–	–	–	–	–	–	–	–	–	–	2	–	–	–	–	–	–	1							
Placidochromis longimanus										11	–	2	3	–	–	–	–	–	–	–	–	–	1	2	8	–	1												
Placidochromis macrognathus					1	–	–	–	–	–	–	–	–	–	–	–	–	–	–	–	–	–	–	–	1														
Placidochromis milomo																										3	–	1											
Protomelas annectens																									2														
Protomelas fenestratus							1	–	–	1	–	3	2	–	–	–	–	–	–	–	–	–	2	1	15	5	3	–	–	–	–	3							
Protomelas insignis												1	1																										
Protomelas kirkii												1												2*															
Protomelas labridens																								1															
Protomelas ornatus												1	–	–	–	–	–	–	–	–	–	–	–	–	1*														
Protomelas spilopterus										1	–	–	1	–	–	–	–	–	–	–	–	–	–	1	2	–	–	–	–	–	–	1							

Table 8 (continued). Frequency distribution of pterygiophore insertion patterns of last 4 occupied dorsal insertion spaces

	|1/1/1/1|	|1/–/1/2|	|1/1/1/2|	|1/1/2/1|	|1/1/2/2|	|1/1/2/3|	|1/1/3/1|	|1/2/1/1|	|1/2/1/2|	|1/2/1/3|	|1/2/1/4|	|1/2/2/1|	|1/2/2/2|	|1/2/2/3|	|1/2/3/1|	|1/2/3/2|	|1/2/3/3|	|1/3/1/1|	|1/3/2/1|	|1/3/2/2|	|2/1/1/1|	|2/1/1/2|	|2/1/1/3|	|2/1/2/1|	|2/1/2/2|	|2/1/2/3|	|2/1/3/1|	|2/1/3/2|	|2/2/1/1|	|2/2/1/2|	|2/2/1/3|	|2/2/2/1|	|2/2/2/2|	|2/2/2/3|	|2/2/3/1|	|2/2/3/2|	|2/3/2/1|	|2/3/2/2|	?
Protomelas taeniolatus																									1														
Protomelas triaenodon																									2	–	–	–	–	–	–	1							
Sciaenochromis ahli										1	–	1	5	–	–	–	–	–	–	–	–	–	–	1	6	–	1	–	–	–	–	1							
Stigmatochromis modestus												1*	–	–	–	–	–	–	–	–	–	–	–	–	2	1	–	–	–	–	–	1							
Stigmatochromis pholidophorus												1	–	–	1*	–	–	–	–	–	–	–	–	–	–	3													
Stigmatochromis pleurospilus																																							1*
Stigmatochromis woodi													3*	–	–	–	–	–	–	–	–	–	–	–	2	–	–	–	–	–	–	–	–	–	–	–	–	–	2
Taeniochromis holotaenia																									1														
Taeniolethrinops praeorbitalis												1																											
Tramitichromis brevis																									1														
Trematocranus labifer													1	–	–	–	–	–	–	–	–	–	–	–	3														
Trematocranus microstoma										1	–	3	2	–	–	–	–	–	–	–	–	–	–	–	2	1													
Trematocranus placodon												1	–	–	–	–	–	–	–	–	–	–	–	–	2														
Tyrannochromis macrostoma								1																															
Tyrannochromis nigriventer																									1*														
**Cyrtocarina column totals**	**–**	**–**	**–**	**–**	**9**	**2**	**5**	**2**	**8**	**27**	**2**	**62**	**46**	**2**	**1**	**–**	**–**	**–**	**–**	**–**	**–**	**–**	**4**	**17**	**157**	**32**	**20**	**2**	**–**	**3**	**1**	**19**	**1**	**–**	**–**	**–**	**–**	**–**	**3**
**Pseudocrenilabrini: Pseudotropheina**																																							
Abactochromis labrosus					2*	–	–	–	1	–	–	3	–																										
Chindongo bellicosus									–	2	–	2	1	–	–	–	–	–	–	–	–	–	–	1															
Chindongo minutus													1																										
Cyathochromis obliquidens									1	–	–	–	–	–	–	–	–	–	–	–	–	–	–	2	–	–	–	–	1										
Cynotilapia afra												1																											
Cynotilapia sp.									1	1	–	2																											
Genyochromis mento									2	1	–	4	–	–	–	–	–	1	–	–	–	–	–	2															
Gephyrochromis lawsi												1																											
Gephyrochromis moorii										2	–	–	–	–	–	–	–	–	–	–	–	–	–	2															
Iodotropheus sprengerae					2	–	–	1	–	–	–	3																											
Labeotropheus fuelleborni					2	–	–	–	3	–	–	5	3	–	–	–	–	–	–	–	–	–	–	2															
Labeotropheus trewavasae									1	–	–	2																											
Labidochromis caeruleus												1																											
Labidochromis freibergi					1*																																		
Labidochromis joanjohnsonae													1*																										
Labidochromis pallidus					4	–	–	–	5	3	–	11																											
Labidochromis shiranus												1																											
Labidochromis textilis									2*																														
Labidochromis vellicans					5	–	–	–	7	1	–	13	–	–	–	–	–	–	–	–	–	–	–	3	–	–	–	–	–	–	–	–	–	–	–	–	–	–	1
Maylandia aurora												1*																											
Maylandia koningsi					1																																		
Maylandia lanisticola																								1*															
Maylandia zebra						2	–	–	1	2	–	3	–	–	–	–	–	–	–	–	–	–	–	1															
Melanochromis auratus					1	1	–	–	–	1	–	3*																											
Melanochromis chipokae					1*																																		
Melanochromis loriae										2	–	1*	–	–	–	–	–	–	–	–	–	–	–	–	1														
Melanochromis melanopterus					1	1	–	–	–	–	–	6	–	–	1	–	–	–	–	–	–	–	–	2															
Melanochromis simulans					1																																		
Melanochromis cf. *vermivorus*									1	–	–	1																											
Petrotilapia genalutea												1																											
Petrotilapia nigra												1																											
Petrotilapia tridentiger												1																											
Petrotilapia sp.										2	–	2																											
Pseudotropheus elegans																								1*															
Pseudotropheus interruptus					1	–	–	–	–	–	–	–	–	–	–	–	–	–	–	–	–	–	–	1*															

Table 8 (continued). Frequency distribution of pterygiophore insertion patterns of last 4 occupied dorsal insertion spaces

	/1/1/1/1/	/1/–/1/2/	1/1/1/2/	/1/1/2/1/	/1/1/2/2/	/1/1/2/3/	/1/1/3/1/	/1/2/1/1/	/1/2/1/2/	/1/2/1/3/	/1/2/1/4/	/1/2/2/1/	/1/2/2/2/	/1/2/2/3/	/1/2/3/1/	/1/2/3/2/	/1/2/3/3/	/1/3/1/1/	/1/3/2/1/	/1/3/2/2/	/2/1/1/1/	/2/1/1/2/	/2/1/1/3/	/2/1/2/1/	/2/1/2/2/	/2/1/2/3/	/2/1/3/1/	/2/1/3/2/	/2/2/1/1/	/2/2/1/2/	/2/2/1/3/	/2/2/2/1/	/2/2/2/2/	/2/2/2/3/	/2/2/3/1/	/2/2/3/2/	/2/3/2/1/	/2/3/2/2/	?
Pseudotropheus johannii					4	1	–	–	9*	6	–	14	–	–	–	–	–	–	–	–	–	–	–	3															
Pseudotropheus livingstonii									1	1	–	3	–	–	–	–	–	–	–	–	–	–	–	1*	1														
Pseudotropheus lucerna										1																													
Tropheops tropheops						1	–	–	–	2	–	2	1	–	–	–	–	–	–	–	–	–	–	1															
**Pseudotropheina column totals**	**–**	**–**	**–**	**–**	**26**	**6**	**–**	**1**	**35**	**27**	**–**	**88**	**7**	**–**	**1**	**–**	**–**	**1**	**–**	**–**	**–**	**–**	**–**	**23**	**2**	**–**	**–**	**–**	**1**	**–**	**–**	**–**	**–**	**–**	**–**	**–**	**–**	**–**	**1**
**Pseudocrenilabrini: Rhamphochromina**																																							
Diplotaxodon argenteus									1	–	–	6*	–	–	–	–	–	–	–	–	–	–	–	–	2	1													
Diplotaxodon ecclesi												1*																											
Diplotaxodon greenwoodi												1*																											
Diplotaxodon limnothrissa					1	–	–	–	3	–	–	4	–	–	–	–	–	–	–	–	–	–	–	–	2														
Pallidochromis tokolosh												1	1	–	–	–	–	–	–	–	–	–	–	–	1	1													
Rhamphochromis brevis					1*	–	–	–	–	–	–	1																											
Rhamphochromis esox												2																											
Rhamphochromis woodi					1																																		
**Rhamphochromina column totals**	**–**	**–**	**–**	**–**	**3**	**–**	**–**	**–**	**4**	**–**	**–**	**16**	**1**	**–**	**–**	**–**	**–**	**–**	**–**	**–**	**–**	**–**	**–**	**–**	**5**	**2**	**–**	**–**	**–**	**–**	**–**	**–**	**–**	**–**	**–**	**–**	**–**	**–**	**–**
**Cyrtocarina column totals**	**–**	**–**	**–**	**–**	**9**	**2**	**5**	**2**	**8**	**27**	**2**	**62**	**46**	**2**	**1**	**–**	**–**	**–**	**–**	**–**	**–**	**–**	**4**	**17**	**157**	**32**	**20**	**2**	**–**	**3**	**1**	**19**	**1**	**–**	**–**	**–**	**–**	**–**	**3**
**Pseudotropheina column totals**	**–**	**–**	**–**	**–**	**26**	**6**	**–**	**1**	**35**	**27**	**–**	**88**	**7**	**–**	**1**	**–**	**–**	**1**	**–**	**–**	**–**	**–**	**–**	**23**	**2**	**–**	**–**	**–**	**1**	**–**	**–**	**–**	**–**	**–**	**–**	**–**	**–**	**–**	**1**
**Rhamphochromina column totals**	**–**	**–**	**–**	**–**	**3**	**–**	**–**	**–**	**4**	**–**	**–**	**16**	**1**	**–**	**–**	**–**	**–**	**–**	**–**	**–**	**–**	**–**	**–**	**–**	**5**	**2**	**–**	**–**	**–**	**–**	**–**	**–**	**–**	**–**	**–**	**–**	**–**	**–**	**–**
