# Supplementary material for: African cichlid fishes: morphological data and taxonomic insights from a genus-level survey of supraneurals, pterygiophores, and vertebral counts (Ovalentaria, Blenniiformes, Cichlidae, Pseudocrenilabrinae)
Source: Biodivers Data J. 2024 Oct 18;12:e130707. doi: 10.3897/BDJ.12.e130707 (PMC11512106; doi:10.3897/BDJ.12.e130707)
Supplement: Supplementary material 9 — Table S8. [file bdj-12-e130707-s009.pdf]

Table 9. Frequency distribution of pterygiophore insertion patterns of last 4 occupied interhemal spaces

	|–/1/2/2|	|–/1/2/3|	|–/2/1/2|	|–/2/1/3|	|–/2/2/2|	|1/1/1/1|	|1/1/1/2|	|1/1/1/3|	|1/1/2/1|	|1/–/2/2|	|1/1/2/2|	|1/1/2/3|	|1/1/3/1|	|1/1/3/2|	|1/2/1/1|	|1/2/1/2|	|1/2/1/3|	|1/2/1/4|	|1/2/2/1|	|1/2/2/2|	|1/2/2/3|	|1/2/3/1|	|2/1/1/1|	|2/1/1/2|	|2/1/1/3|	|2/1/2/1|	|2/1/2/2|	|2/1/2/3|	|2/1/3/1|	|2/1/3/2|	|2/2/1/1|	|2/2/1/2|	|2/2/1/3|	|2/2/2/1|	|2/2/2/2|	|2/2/2/3|	|2/2/3/1|	|2/2/3/2|	|3/1/2/3|	|3/3/2/3|	?
Polycentridae																																									
Afronandus sheljuzhkoi									1																																
Monocirrhus polyacanthus																																						1	–	1	
Polycentropsis abbreviata																																			2						
Cichlidae: Etroplinae																																									
**India**																																									
Etroplus suratensis																					1																				
Pseudetroplus maculatus																												1	–	–	–	–	–	4	2						
**Madagascar**																																									
Paretroplus polyactis																																			1						
Ptychochrominae																																									
**Madagascar (Ptychochrominae)**																																									
Katria katria																			1																						
Oxylapia polli															1																										
Paratilapia polleni																			1	3	–	–	–	–	–	3	2	–	–	–	1	–	–	–	1						
Ptychochromis oligacanthus											1	–	–	–	1	–	–	–	2	2	–	–	–	–	–	3	2														
Ptychochromoides betsileanus																											1	–	–	–	–	–	–	1	–	1					
Cichlinae																																									
**Neotropics**																																									
**Astronotini**																																									
Astronotus ocellatus											1	–	–	–	–	1	–	–	–	–	–	–	–	–	–	–	1														
**Chaetobranchini**																																									
Chaetobranchopsis orbicularis																																		1							
Chaetobranchus flavescens																				1	–	–	–	–	–	–	–	2	–	–	–	–	–	–	1						
**Cichlasomatini**																																									
Aequidens tetramerus															1																										
Andinoacara pulcher											1																														
Bujurquina vittata																			1																						
Cichlasoma dimerus																										1															
Cichlasoma taenia									1																																
Krobia guianensis																			1																						
Cichla ocellaris																																			1						
Cichla orinocensis																																		2	1						
**Geophagini**																																									
Biotodoma cupido											1	–	–	–	–	–	–	–	–	–	–	–	–	–	–	–	–	–	–	–	–	–	–	3							
Geophagus crassilabris									1																																
Guianacara owroewefi											1	2	–	–	–	2	–	–	3																						
Saxatilia frenata																1*																									
Saxatilia lepidota																											1	–	–	–	–	–	–	1							
**Heroini**																																									
Amphilophus citrinellus												1																													
Hypselacara coryphaenoides																				2	–	–	–	–	–	–	3														
**Retroculini**																																									
Retroculus lapidifer									1																																
Pseudocrenilabrinae																																									
**Middle East**																																									
**Oreochromini**																																									
Iranocichla hormuzensis																			1	–	–	–	–	–	–	–	–	–	–	–	–	–	–	1							

Table 9 (continued). Frequency distribution of pterygiophore insertion patterns of last 4 occupied interhemal spaces

	/–/1/2/2/	/–/1/2/3/	/–/2/1/2/	/–/2/1/3/	/–/2/2/2/	/1/1/1/1/	/1/1/1/2/	/1/1/1/3/	/1/1/2/1/	/1/–/2/2/	/1/1/2/2/	/1/1/2/3/	/1/1/3/1/	/1/1/3/2/	/1/2/1/1/	/1/2/1/2/	/1/2/1/3/	/1/2/1/4/	/1/2/2/1/	/1/2/2/2/	/1/2/2/3/	/1/2/3/1/	/2/1/1/1/	/2/1/1/2/	/2/1/1/3/	/2/1/2/1/	/2/1/2/2/	/2/1/2/3/	/2/1/3/1/	/2/1/3/2/	/2/2/1/1/	/2/2/1/2/	/2/2/1/3/	/2/2/2/1/	/2/2/2/2/	/2/2/2/3/	/2/2/3/1/	/2/2/3/2/	/3/1/2/3/	/3/3/2/3/	?
**Pseudocrenilabrini**																																									
Astatotilapia flavijosephi																			1	2	–	–	–	–	–	–	–	–	–	–	–	–	–	1							
**African riverine**																																									
**Chromidotilapiini**																																									
Benitochromis batesii											2	–	–	–	–	2	–	–	2																						
Benitochromis finleyi											1																														
Chromidotilapia guntheri																			2																						
Chromidotilapia kingsleyae											2	1	–	–	–	–	–	–	2	–	–	–	–	–	–	1	1	–	1	–	–	–	–	1							
Congochromis robustus															1*																										
Congochromis squamiceps											1	–	–	–	–	–	–	–	1																						
Divandu albimarginatus											1	1	–	–	–	–	–	–	1	1																					
Limbochromis robertsi									1	–	1	–	–	–	–	2	–	–	7	–	–	–	–	–	–	5	–	–	–	–	1										
Nanochromis nudiceps									1	–	1																														1
Nanochromis parilus											1	–	–	–	–	–	–	–	–	–	–	–	1																		
Parananochromis gabonicus											1*																														
Parananochromis longirostris											2	2	–	–	–	–	1	–	4	–	–	–	–	–	–	2															
Pelmatochromis buettikoferi																					1	–	–	–	–	–	–	1	–	–	–	–	–	1							
Pelmatochromis ocellifer																				1*																					
Pelvicachromis pulcher																										1															
Pelvicachromis taeniatus															1*																										
Pterochromis congicus																					1	–	–	–	–	–	–	–	–	–	–	–	–	1*							
Teleogramma brichardi																										4															
Thysochromis ansorgii																			3	–	–	–	–	–	–	–	–	–	–	–	1										
Wallaceochromis humilis											2*	–	–	–	1	–	–	–	1	–	–	–	–	–	–	1															
**Chromidotilapiini column totals**	**–**	**–**	**–**	**–**	**–**	**–**	**–**	**–**	**2**	**–**	**15**	**4**	**–**	**–**	**3**	**4**	**1**	**–**	**23**	**2**	**2**	**–**	**1**	**–**	**–**	**14**	**1**	**1**	**1**	**–**	**2**	**–**	**–**	**3**	**–**	**–**	**–**	**–**	**–**	**–**	**1**
**Coelotilapiini**																																									
Coelotilapia joka																				1																					
**Coptodonini**																																									
Coptodon discolor																																		2							
Coptodon tholloni																					1																				
Coptodon zillii																4	–	–	5	1	–	–	–	–	–	–	1	1	1	–	–	–	–	3	5	2	1				
**Coptodonini column totals**	**–**	**–**	**–**	**–**	**–**	**–**	**–**	**–**	**–**	**–**	**–**	**–**	**–**	**–**	**–**	**4**	**–**	**–**	**5**	**1**	**1**	**–**	**–**	**–**	**–**	**–**	**1**	**1**	**1**	**–**	**–**	**–**	**–**	**5**	**5**	**2**	**1**	**–**	**–**	**–**	**–**
**Etiini**																																									
Etia nguti																				2	–	1	–	–	–	–	–	–	–	–	–	–	–	2	–	–	1				
**Gobiocichlini**																																									
Gobiocichla ethelwynnae									1	–	1	–	–	–	2	5	1	–	–	1																					
Gobiocichla wonderi									1	–	–	–	–	–	4	3	–	–	1	–	–	–	–	2	–	3															
Paragobiocichla irvinei											2	–	–	–	–	–	–	–	1	–	–	–	–	–	–	1															
**Gobiocichlini column totals**	**–**	**–**	**–**	**–**	**–**	**–**	**–**	**–**	**2**	**–**	**3**	**–**	**–**	**–**	**6**	**8**	**1**	**–**	**2**	**1**	**–**	**–**	**–**	**2**	**–**	**4**	**–**	**–**	**–**	**–**	**–**	**–**	**–**	**–**	**–**	**–**	**–**	**–**	**–**	**–**	**–**
**Hemichromini**																																									
Anomalochromis thomasi																			2	–	–	–	–	–	–	–	1	–	–	–	–	–	–	1							
Hemichromis elongatus																				1	–	–	–	–	–	–	1	–	–	–	–	–	–	1	–	–	–	–	1		
Hemichromis fasciatus																																		1							
Rubricatochromis bimaculatus																			4	–	–	–	–	–	–	1	1														
Rubricatochromis letourneuxi																1	–	–	2							1															
**Hemichromini column totals**	**–**	**–**	**–**	**–**	**–**	**–**	**–**	**–**	**–**	**–**	**–**	**–**	**–**	**–**	**–**	**1**	**–**	**–**	**8**	**1**	**–**	**–**	**–**	**–**	**–**	**2**	**3**	**–**	**–**	**–**	**–**	**–**	**–**	**3**	**–**	**–**	**–**	**–**	**1**	**–**	**–**
**Heterochromini**																																									
Heterochromis multidens														1	–	–	–	–	–	6	1	–	–	–	–	–	2	–	–	–	1										
**Heterotilapiini**																																									
Heterotilapia buettikoferi																																			3	1	–	1			

Table 9 (continued). Frequency distribution of pterygiophore insertion patterns of last 4 occupied interhemal spaces

	|–|1|2|2|	|–|1|2|3|	|–|2|1|2|	|–|2|1|3|	|–|2|2|2|	|1|1|1|1|	|1|1|1|2|	|1|1|1|3|	|1|1|2|1|	|1|–|2|2|	|1|1|2|2|	|1|1|2|3|	|1|1|3|1|	|1|1|3|2|	|1|2|1|1|	|1|2|1|2|	|1|2|1|3|	|1|2|1|4|	|1|2|2|1|	|1|2|2|2|	|1|2|2|3|	|1|2|3|1|	|2|1|1|1|	|2|1|1|2|	|2|1|1|3|	|2|1|2|1|	|2|1|2|2|	|2|1|2|3|	|2|1|3|1|	|2|1|3|2|	|2|2|1|1|	|2|2|1|2|	|2|2|1|3|	|2|2|2|1|	|2|2|2|2|	|2|2|2|3|	|2|2|3|1|	|2|2|3|2|	|3|1|2|3|	|3|3|2|3|	?
Oreochromis niloticus																				2	–	1																			
Oreochromis shiranus																			1	1	–	1	–	–	–	–	–	–	–	–	–	–	–	2	1						
Sarotherodon galilaeus																						1	–	–	–	–	–	–	–	–	–	–	–	–	1						
Sarotherodon melanotheron																				1	1	–	–	–	–	–	–	2	1	1											
**Oreochromini (riverine) totals**	**–**	**–**	**–**	**–**	**1**	**–**	**–**	**–**	**–**	**–**	**1**	**1**	**–**	**–**	**–**	**–**	**1**	**–**	**3**	**10**	**1**	**3**	**–**	**–**	**–**	**1**	**–**	**4**	**1**	**1**	**–**	**–**	**–**	**4**	**3**	**–**	**–**	**–**	**–**	**–**	**–**
**Pelmatolapiini**																																									
Pelmatolapia mariae																												1	–	–	–	–	–	–	3	1	1				
**Pseudocrenilabrini**																																									
Astatotilapia bloyeti											1	–	–	–	–	1	1	–	2	1	–	–	–	–	–	–	2														
Astatotilapia burtoni																			2	2	–	–	–	–	–	–	2														
Astatotilapia calliptera									2	–	1	–	–	–	–	3*	1	–	2																						
Astatotilapia swynnertoni											1	–	–	–	–	–	–	–	1	–	–	–	–	–	–	–	–	–	–	–	–	–	–	1							
Chetia flaviventris																																1									
Chetia gracilis																				1*																					
Chetia ? sp.																				1	–	–	–	–	–	–	2	–	–	–	–	–	1	2	–	–	–	–	–	–	2
Ctenochromis pectoralis																			1	–	–	–	–	–	–	–	1														
Ctenochromis scatebra																											1*														
Haplochromis demeusii											1*																														
Haplochromis fasciatus		1	–	–	1	–	–	–	–	–	3	–	–	–	–	–	–	–	1																						
Haplochromis humilis																			2	–	–	–	–	–	–	–	–	–	1												
Haplochromis moeruensis											2	–	–	–	–	–	–	–	1	–	–	–	–	–	–	–	1														
Haplochromis oligacanthus			1*																																						
Lufubuchromis relictus																	1*																								
Orthochromis machadoi											2																														
Orthochromis malagaraziensis																							1	1																	
Orthochromis polyacanthus	1	–	–	–	–	–	–	–	–	–	–	–	–	–	1																										
Orthochromis stormsi					1	–	–	–	4	–	1	–	–	–	–	2	–	–	2	–	–	–	–	1																	
Palaeoplex palimpsest												1*																													
Pharyngochromis acuticeps					1	–	–	–	–	–	1	–	–	–	–	1	–	–	1	1	–	–	–	–	–	–	–	–	–	–	–	–	–	1							
Pseudocrenilabrus multicolor																				1	–	–	–	–	–	3															
Pseudocrenilabrus philander																			1																						
Sargochromis carlottae																				1	–	–	–	–	–	–	–	1													
Sargochromis codringtonii																				3*																					
Sargochromis giardi																			1	–	–	–	–	–	–	–	–	–	–	–	–	–	–	1							
Sargochromis greenwoodi																				1	–	–	–	–	–	–	1*														
Serranochromis angusticeps																															1										
Serranochromis longimanus																															3*	–	–	–	1						
Serranochromis macrocephalus																															1										
Serranochromis meridianus																			1																						
Serranochromis robustus																			4	–	–	–	–	–	–	–	–	–	–	–	1	1									
Thoracochromis albolabris																										1															
Thoracochromis wingatii																								1	–	1															
**Pseudocrenilabrini (riverine) totals**	**1**	**1**	**1**	**–**	**3**	**–**	**–**	**–**	**6**	**–**	**13**	**1**	**–**	**–**	**1**	**7**	**3**	**–**	**22**	**12**	**–**	**–**	**1**	**3**	**–**	**5**	**10**	**1**	**1**	**–**	**6**	**2**	**1**	**5**	**1**	**–**	**–**	**–**	**–**	**–**	**2**
**Steatocranini**																																									
Steatocranus casuarius									2	–	1																														
**Tilapiini**																																									
Chilochromis duponti																				1																					
Congolapia bilineata																			1	1	–	–	–	–	–	1															
Tilapia busumana																			2	1	–	–	–	–	–	–	–	–	–	–	–	–	–	1							
Tilapia sparrmanii																			1	1*	–	–	–	–	–	–	–	1	–	–	–	–	–	1							

Table 9 (continued). Frequency distribution of pterygiophore insertion patterns of last 4 occupied interhemal spaces

	|–/1/2/2/	|–/1/2/3/	|–/2/1/2/	|–/2/1/3/	|–/2/2/2/	|1/1/1/1/	|1/1/1/2/	|1/1/1/3/	|1/1/2/1/	|1/–/2/2/	|1/1/2/2/	|1/1/2/3/	|1/1/3/1/	|1/1/3/2/	|1/2/1/1/	|1/2/1/2/	|1/2/1/3/	|1/2/1/4/	|1/2/2/1/	|1/2/2/2/	|1/2/2/3/	|1/2/3/1/	|2/1/1/1/	|2/1/1/2/	|2/1/1/3/	|2/1/2/1/	|2/1/2/2/	|2/1/2/3/	|2/1/3/1/	|2/1/3/2/	|2/2/1/1/	|2/2/1/2/	|2/2/1/3/	|2/2/2/1/	|2/2/2/2/	|2/2/2/3/	|2/2/3/1/	|2/2/3/2/	|3/1/2/3/	|3/3/2/3/	?
**Lake Barombi Mbo**																																									
**Oreochromini**																																									
Konia eisentrauti																				2	–	–	–	–	–	–	1	1	–	–	–	–	–	1							
Myaka myaka																				2	–	–	–	–	–	–	–	–	–	–	–	–	–	1	1						
Pungu maclareni																				1*	–	1																			
Stomatepia mariae																																			1						
Stomatepia pindu																				1	–	1	–	–	–	–	–	–	–	–	–	–	–	–	1	–	1				
**Oreochromini (Barombi) totals**	**–**	**–**	**–**	**–**	**–**	**–**	**–**	**–**	**–**	**–**	**–**	**–**	**–**	**–**	**–**	**–**	**–**	**–**	**–**	**6**	**–**	**2**	**–**	**–**	**–**	**–**	**1**	**1**	**–**	**–**	**–**	**–**	**–**	**2**	**3**	**–**	**1**	**–**	**–**	**–**	**–**
**Lake Fwa**																																									
**Pseudocrenilabrini**																																									
Cyclopharynx schwetzi					1	–	–	–	–	–	1	–	–	–	–	–	–	–	4	5	–	1	–	–	–	–	2														
Schwetzochromis neodon											1	–	–	–	1																										
**Lake Albert**																																									
**Pseudocrenilabrini**																																									
Haplochromis avium									2	–	–	–	–	–	–	–	–	–	1																						
Haplochromis loati																											1														
**Lake Edward–George system**																																									
**Pseudocrenilabrini**																																									
Haplochromis akika											1	–	–	–	–	2*	–	–	–	–	–	–	–	–	–	–	1														
Haplochromis aquila																											1*														
Haplochromis aureus																										1*															
Haplochromis curvidens																											1*														
Haplochromis falcatus																1*																									
Haplochromis fuscus																1																									
Haplochromis glaucus																				1*																					
Haplochromis gracilifur																										1*															
Haplochromis kimondo																															1*										
Haplochromis labiatus																										1															
Haplochromis latifrons																										1*															
Haplochromis limax																1	–	–	1	1	–	–	–	–	–	1															
Haplochromis mentatus																			1*																						
Haplochromis molossus																1*																									
Haplochromis pappenheimi																1*																									
Haplochromis paradoxus											1*																														
Haplochromis pardus											1*																														
Haplochromis pelagicus																											1*														
Haplochromis pharyngalis																											1*														
Haplochromis quasimodo																	1*																								
Haplochromis relictidens									1*																																
Haplochromis rex																1*																									
Haplochromis simba																			1*																						
Haplochromis squamipinnis									1*																																
Haplochromis taurinus																1*																									
Schubotzia eduardiana									2	–	–	–	–	–	–	1	–	–	1	–	–	–	–	–	–	2	4														
**Pseudocrenilabrini (Ed–Grg) totals**	**–**	**–**	**–**	**–**	**–**	**–**	**–**	**–**	**4**	**–**	**3**	**–**	**–**	**–**	**–**	**10**	**1**	**–**	**4**	**2**	**–**	**–**	**–**	**–**	**–**	**7**	**9**	**–**	**–**	**–**	**1**	**–**	**–**	**–**	**–**	**–**	**–**	**–**	**–**	**–**	**–**
**Lake Victoria & satellites**																																									
**Pseudocrenilabrini**																																									
Allochromis welcommei																											1														
Astatoreochromis alluaudi																																1									
Haplochromis chlorochrous									1	–	1	–	–	–	–	–	–	–	1	–	–	–	–	1																	

Table 9 (continued). Frequency distribution of pterygiophore insertion patterns of last 4 occupied interhemal spaces

	|–/1/2/2|	|–/1/2/3|	|–/2/1/2|	|–/2/1/3|	|–/2/2/2|	|1/1/1/1|	|1/1/1/2|	|1/1/1/3|	|1/1/2/1|	|1/–/2/2|	|1/1/2/2|	|1/1/2/3|	|1/1/3/1|	|1/1/3/2|	|1/2/1/1|	|1/2/1/2|	|1/2/1/3|	|1/2/1/4|	|1/2/2/1|	|1/2/2/2|	|1/2/2/3|	|1/2/3/1|	|2/1/1/1|	|2/1/1/2|	|2/1/1/3|	|2/1/2/1|	|2/1/2/2|	|2/1/2/3|	|2/1/3/1|	|2/1/3/2|	|2/2/1/1|	|2/2/1/2|	|2/2/1/3|	|2/2/2/1|	|2/2/2/2|	|2/2/2/3|	|2/2/3/1|	|2/2/3/2|	|3/1/2/3|	|3/3/2/3|	?
Haplochromis lividus																				2																					
Haplochromis nubilus																1	–	–	–	2*																					
Haplochromis plutonius											2	–	–	–	–	–	–	–	1	2																					
Lithochromis rubripinnis																			2	–	–	–	–	–	–	1	1														
Lithochromis xanthopteryx									1	–	1	–	–	–	–	1	–	–	1																						
Mbipia mbipi																							1																		
Neochromis nigricans																			1																						
Paralabidochromis victoriae															1	–	–	–	–	–	–	–	–	–	–	–	1														
Pundamilia igneopinnis																										1	1														
Pundamilia pundamilia																1	–	–	1	–	–	–	–	–	–	2															
Pyxichromis parorthostoma									1																																
**Pseudocrenilabrini (Victoria) totals**	**–**	**–**	**–**	**–**	**–**	**–**	**–**	**–**	**3**	**–**	**7**	**–**	**–**	**–**	**1**	**7**	**–**	**–**	**10**	**9**	**–**	**–**	**1**	**2**	**–**	**6**	**5**	**–**	**–**	**–**	**–**	**1**	**–**	**–**	**–**	**–**	**–**	**–**	**–**	**–**	**–**
**Lake Kivu**																																									
**Pseudocrenilabrini**																																									
Haplochromis astatodon											1	–	–	–	1*	1	–	–	1	1	–	–	–	–	–	–	1														
Haplochromis paucidens											1	–	–	–	–	–	–	–	1																						
**Lake Turkana**																																									
**Pseudocrenilabrini**																																									
Haplochromis rudolfianus											2	–	–	–	–	1	–	–	–	2	–	–	–	–	–	2	1														
Haplochromis turkanae									1	–	1	–	–	–	–	1																									
**Lake Tanganyika**																																									
**Bathybatini**																																									
Bathybates fasciatus																											1	–	–	–	–	–	–	4							
Bathybates ferox																			1	3	–	–	–	–	–	–	3	–	–	–	–	3									
Bathybates graueri																			2	–	–	–	–	–	–	2	–	–	–	–	–	1									
Bathybates hornii																											1														
Bathybates leo																			1	1	–	–	–	–	–	–	–	–	–	–	–	2	–	1							
Bathybates minor																										1	–	–	–	–	2	–	–	–	2						
Bathybates vittatus																											1														
Hemibates stenosoma																																		1	3						
Trematocara unimaculatum									1	–	–	–	–	–	1	–	–	–	–	–	–	–	–	–	–	1	–	–	–	–	2										
Trematocara zebra							1	–	–	–	–	–	–	–	1	2	–	–	–	–	–	–	–	–	–	1															
**Bathybatini column totals**	**–**	**–**	**–**	**–**	**–**	**–**	**1**	**–**	**1**	**–**	**–**	**–**	**–**	**–**	**2**	**2**	**–**	**–**	**4**	**4**	**–**	**–**	**–**	**–**	**–**	**5**	**6**	**–**	**–**	**–**	**4**	**6**	**–**	**6**	**5**	**–**	**–**	**–**	**–**	**–**	**–**
**Benthochromini**															1	1	–	–	1	–	–	–	–	–	–	1	–	–	–	–	1										
Benthochromis tricoti															1	1	–	–	1	–	–	–	–	–	–	1	–	–	–	–	1										
**Boulengerochromini**																																									
Boulengerochromis microlepis																			1	3	–	–	–	–	–	–	–	1	–	–	–	1	–	–	1						
**Cyphotilapiini**																																									
Cyphotilapia frontosa			1	–	–	–	–	–	–	–	3	–	–	–	–	1																									
Cyphotilapia gibberosa											1	–	–	–	–	1	–	–	3																						
**Cyprichromini**																																									
Cyprichromis coloratus									1	–	–	–	–	–	–	3	–	–	–	–	–	–	–	1																	
Cyprichromis leptosoma			1	–	–	–	–	–	–	–	–	–	–	–	–	1	–	–	–	–	–	–	–	–	–	3															
Cyprichromis microlepidotus									1	–	–	–	–	–	1	1	–	–	–	–	–	–	–	–	–	2															
Cyprichromis pavo																			1	–	–	–	–	–	–	4															
Cyprichromis zonatus												1	–	–	–	1	1	–	2																						
Cyprichromis sp. "dwarf jumbo"									1	–	–	–	–	–	2	2																									
Cyprichromis sp. "jumbo"						1	–	–	1	–	–	–	–	–	–	1	–	–	–	–	–	–	1	1																	
Paracyprichromis brieni											1	–	–	–	1	2	–	–	–	–	–	–	–	–	–	–	1														
Paracyprichromis nigripinnis						1	–	–	3																																

Table 9 (continued). Frequency distribution of pterygiophore insertion patterns of last 4 occupied interhemal spaces

	|–/1/2/2|	|–/1/2/3|	|–/2/1/2|	|–/2/1/3|	|–/2/2/2|	|1/1/1/1|	|1/1/1/2|	|1/1/1/3|	|1/1/2/1|	|1/–/2/2|	|1/1/2/2|	|1/1/2/3|	|1/1/3/1|	|1/1/3/2|	|1/2/1/1|	|1/2/1/2|	|1/2/1/3|	|1/2/1/4|	|1/2/2/1|	|1/2/2/2|	|1/2/2/3|	|1/2/3/1|	|2/1/1/1|	|2/1/1/2|	|2/1/1/3|	|2/1/2/1|	|2/1/2/2|	|2/1/2/3|	|2/1/3/1|	|2/1/3/2|	|2/2/1/1|	|2/2/1/2|	|2/2/1/3|	|2/2/2/1|	|2/2/2/2|	|2/2/2/3|	|2/2/3/1|	|2/2/3/2|	|3/1/2/3|	|3/3/2/3|	?
**Ectodini**																																									
Asprotilapia leptura							2	–	–	–	–	–	–	–	–	–	–	–	–	–	–	–	3																		
Aulonocranus dewindti											1	–	–	–	–	2	–	–	1	–	–	–	–	–	–	1															
Callochromis macrops							1	–	3	–	1																														
Cardiopharynx schoutedeni									2	–	–	–	–	–	–	–	–	–	–	–	–	–	–	3																	
Cunningtonia longiventralis															3	1	–	–	–	–	–	–	–	–	–	1															
Cyathopharynx furcifer									1	–	–	–	–	–	–	2	–	–	–	–	–	–	–	–	–	2															
Ectodus descampsii									2	–	1	–	–	–	1	1																									
Enantiopus melanogenys							1	–	3	–	–	–	–	–	1																										
Grammatotria lemairii							5																																		
Lestradea perspicax									2	–	2	–	–	–	–	1	–	–	–	–	–	–	–	1																	
Microdontochromis tenuidentatus						2	2	–	1																																
Ophthalmotilapia boops								1	–	–	1	–	–	–	–	–	1	–	1	–	–	–	–	–	–	1															
Xenotilapia sima						2	3																																		
**Ectodini column totals**	**–**	**–**	**–**	**–**	**–**	**4**	**14**	**1**	**14**	**–**	**6**	**–**	**–**	**–**	**5**	**7**	**1**	**–**	**2**	**–**	**–**	**–**	**3**	**4**	**–**	**5**	**–**	**–**	**–**	**–**	**–**	**–**	**–**	**–**	**–**	**–**	**–**	**–**	**–**	**–**	**–**
**Eretmodini**																																									
Eretmodus cyanostictus							1	–	1	–	1	–	–	–	–	1	–	–	1																						
Eretmodus marksmithi									4	–	1																														
Spathodus erythrodon							1	–	4																																
Tanganicodus irsacae							1	–	2	–	1	–	–	–	1																										
**Eretmodini column totals**	**–**	**–**	**–**	**–**	**–**	**–**	**3**	**–**	**11**	**–**	**3**	**–**	**–**	**–**	**1**	**1**	**–**	**–**	**1**	**–**	**–**	**–**	**–**	**–**	**–**	**–**	**–**	**–**	**–**	**–**	**–**	**–**	**–**	**–**	**–**	**–**	**–**	**–**	**–**	**–**	**–**
**Lamprologini**																																									
Altolamprologus calvus							1	–	2	–	1	–	–	–	1																										
Altolamprologus compressiceps									2	–	2	–	–	–	1																										
Altolamp. 'compressiceps shell'							1	1	2	–	1																														
Chalinochromis brichardi									3	–	–	–	–	–	2																										
Chalinochromis cyanophleps									3	–	–	–	–	–	1	–	–	–	–	–	–	–	–	1																	
Julidochromis ornatus							1	–	4																																
Julidochromis regani						1	3	–	–	–	1																														
Lamprologus callipterus									2	–	2	–	–	–	6																										
Lamprologus 'ornatipinnis congo'									3	–	–	–	–	–	2																										
Lamprologus 'ornatipinnis zambia'								1	6	–	–	–	–	–	2																										
Lamprologus speciosus							1	–	1	–	–	–	–	–	1																										
Lepidiolamprologus kendalli											2	–	–	–	–	3																									
Lepidiolamprologus mimicus											2	–	–	–	1	2																									
Lepidiolamprologus profundicola															1	3	–	–	1																						
Neolamprologus tretocephalus									3	–	2																														
Neolamprologus ventralis							2	1	2																																
Telmatochromis dhonti							2	–	3																																
Telmatochromis temporalis							1	–	2	–	–	–	–	–	2																										
Telmatochromis vittatus							1	–	4																																
Variabilichromis moorii									3	–	–	–	–	–	1	1	–	–	–	–	–	–	–	1	–	1															
**Lamprologini column totals**	**–**	**–**	**–**	**–**	**–**	**1**	**13**	**3**	**45**	**–**	**13**	**–**	**–**	**–**	**21**	**9**	**–**	**–**	**1**	**–**	**–**	**–**	**–**	**2**	**–**	**1**	**–**	**–**	**–**	**–**	**–**	**–**	**–**	**–**	**–**	**–**	**–**	**–**	**–**	**–**	**–**
**Limnochromini**																																									
Baileychromis centropomoides									2	–	1	–	–	–	–	1	–	–	–	–	–	–	–	1																	
Gnathochromis permaxillaris							1	–	–	–	1	–	–	–	–	3																									
Greenwoodochromis christyi															2	3																									
Limnochromis auritus											2	–	–	–	–	1	–	–	2																						
Reganochromis calliurus							1	–	1	–	1	–	–	–	1	1																									
Tangachromis dhanisi							1	–	1	–	–	–	–	–	–	1	–	–	1																						

Table 9 (continued). Frequency distribution of pterygiophore insertion patterns of last 4 occupied interhemal spaces

	|–/1/2/2|	|–/1/2/3|	|–/2/1/2|	|–/2/1/3|	|–/2/2/2|	|1/1/1/1|	|1/1/1/2|	|1/1/1/3|	|1/1/2/1|	|1/–/2/2|	|1/1/2/2|	|1/1/2/3|	|1/1/3/1|	|1/1/3/2|	|1/2/1/1|	|1/2/1/2|	|1/2/1/3|	|1/2/1/4|	|1/2/2/1|	|1/2/2/2|	|1/2/2/3|	|1/2/3/1|	|2/1/1/1|	|2/1/1/2|	|2/1/1/3|	|2/1/2/1|	|2/1/2/2|	|2/1/2/3|	|2/1/3/1|	|2/1/3/2|	|2/2/1/1|	|2/2/1/2|	|2/2/1/3|	|2/2/2/1|	|2/2/2/2|	|2/2/2/3|	|2/2/3/1|	|2/2/3/2|	|3/1/2/3|	|3/3/2/3|	?
**Oreochromini**																																									
Oreochromis tanganicae																				2	–	–	–	–	–	–	1	–	–	–	–	–	–	1	1						
**Perissodini**																																									
Haplotaxodon microlepis											1	–	–	–	–	–	–	–	1	1	–	–	–	–	–	1	1														
Perissodus microlepis											2	–	–	–	–	–	1	–	–	1	–	–	–	–	–	–	1														
Plecodus paradoxus											1	–	–	–	–	1	–	–	–	–	–	–	–	–	–	–	3														
Xenochromis hecqui																			2	–	–	–	–	–	–	3															
**Perissodini column totals**	**–**	**–**	**–**	**–**	**–**	**–**	**–**	**–**	**–**	**–**	**4**	**–**	**–**	**–**	**–**	**1**	**1**	**–**	**3**	**2**	**–**	**–**	**–**	**–**	**–**	**4**	**5**	**–**	**–**	**–**	**–**	**–**	**–**	**–**	**–**	**–**	**–**	**–**	**–**	**–**	**–**
**Pseudocrenilabrini: Tropheina**																																									
Interochromis loocki							1	–	1	–	–	–	–	–	–	3																									
Jabarichromis pfefferi							1	–	2	–	2																														
Limnotilapia dardennii															1	2	–	–	–	–	–	–	–	–	–	1															
Lobochilotes labiata											2	–	–	–	2	1																									
Petrochromis horii											1	–	–	–	–	1	–	–	3																						
Petrochromis polyodon											1	–	–	–	1	1	–	–	1	–	–	–	–	–	–	1															
Pseudosimochromis curvifrons									2	–	–	–	–	–	–	1	–	–	1	–	–	–	–	–	–	1															
Shuja horei							1	–	5	–	1	–	–	–	1																										
Simochromis diagramma									2	–	2	–	–	–	1																										
Tropheus annectens											1	–	–	–	–	1	–	–	1	–	–	–	–	–	–	2															
Tropheus brichardi									1	–	2	–	–	–	–	2																									
Tropheus duboisi									2	–	–	–	–	–	–	2	–	–	–	–	–	–	–	–	–	1															
Tropheus moorii											1	–	–	–	2	5	–	–	1	–	–	–	–	–	–	1															
**Tropheina column totals**	**–**	**–**	**–**	**–**	**–**	**–**	**3**	**–**	**15**	**–**	**13**	**–**	**–**	**–**	**8**	**19**	**–**	**–**	**7**	**–**	**–**	**–**	**–**	**–**	**–**	**7**	**–**	**–**	**–**	**–**	**–**	**–**	**–**	**–**	**–**	**–**	**–**	**–**	**–**	**–**	**–**
**Tylochromini**																																									
Tylochromis polylepis											4	–	–	–	–	–	1	–	1																						
**Lake Malawi**																																									
**Pseudocrenilabrini: Cyrtocarina**																																									
Alticorpus mentale																					1																				
Alticorpus peterdaviesi																				1																					
Aristochromis christyi																												1	1												
Aulonocara nyassae																			1																						
Aulonocara rostratum																	1																								
Aulonocara stonemani																				1*																					
Buccochromis atritaeniatus																											1*	–	–	–	–	–	–	1							
Buccochromis heterotaenia																				1*	–	–	–	–	–	–	1														
Buccochromis nototaenia																			1	–	–	–	–	–	–	–	–	1*													
Buccochromis oculatus																						1	–	–	–	–	1	1													
Buccochromis spectabilis												1																													
Caprichromis orthognathus																	1	–	2	2																					
Champsochromis caeruleus																			1	–	–	–	–	–	–	–	3														
Champsochromis spilorhynchus											1	–	–	–	–	–	–	–	1																						
Cheilochromis euchilus												1	–	–	–	–	–	–	1	3*																					
Chilotilapia rhoadesii																											2	–	–	–	–	–	–	2							
Copadichromis jacksoni																	1		–	1	–	–	–	–	–	–	–	–	–	–	–	–	1								
Copadichromis quadrimaculatus																											1														
Corematodus taeniatus											1	–	1	–	–	–	1	–	1	–	–	–	–	–	–	–	–	2	–	–	–	–	–	1							
Ctenopharynx nitidus											1	2	–	–	–	2	4	–	1	–	–	–	–	–	–	1	5														
Ctenopharynx pictus																											1														
Cyrtocara moorii											2	–	–	–	–	–	–	–	–	–	–	–	–	–	–	1	3*														
Dimidiochromis compressiceps																	1	–	2	2	–	–	–	–	–	–	3	1	1	–	–	–	1	1							

Table 9 (continued). Frequency distribution of pterygiophore insertion patterns of last 4 occupied interhemal spaces

	|–|1|2|2|	|–|1|2|3|	|–|2|1|2|	|–|2|1|3|	|–|2|2|2|	|1|1|1|1|	|1|1|1|2|	|1|1|1|3|	|1|1|2|1|	|1|–|2|2|	|1|1|2|2|	|1|1|2|3|	|1|1|3|1|	|1|1|3|2|	|1|2|1|1|	|1|2|1|2|	|1|2|1|3|	|1|2|1|4|	|1|2|2|1|	|1|2|2|2|	|1|2|2|3|	|1|2|3|1|	|2|1|1|1|	|2|1|1|2|	|2|1|1|3|	|2|1|2|1|	|2|1|2|2|	|2|1|2|3|	|2|1|3|1|	|2|1|3|2|	|2|2|1|1|	|2|2|1|2|	|2|2|1|3|	|2|2|2|1|	|2|2|2|2|	|2|2|2|3|	|2|2|3|1|	|2|2|3|2|	|3|1|2|3|	|3|3|2|3|	?
Exochochromis anagenys												2	–	–	–	–	2*	–	–	2	–	–	–	–	–	–	–	–	–	–	–	–	–	1							
Fossorochromis rostratus																1																									
Hemitaeniochromis urotaenia																			1																						
Hemitilapia oxyrhynchus																	1	–	–	4	–	–	–	–	–	–	2	–	–	–	–	1	1	1							
Lethrinops gossei																			1	–	–	–	–	–	–	–	1*														
Lethrinops lethrinus											1	–	–	–	–	–	–	–	–	1	–	–	–	–	–	–	1														
Lethrinops polli																											1*														
Lichnochromis acuticeps																			1																						
Mchenga cyclicos											1																														
Mchenga inornata											1*	–	–	–	–	1																									
Mchenga sp.											2	1	–	–	–	–	3	–	4	5	–	–	–	–	–	–	4	1	–	–	–	–	–	3							
Mylochromis formosus																1	–	–	–	–	–	–	–	–	–	1*															
Mylochromis gracilis																	1	–	–	–	–	–	–	–	–	–	1	–	–	–	–	1*									
Mylochromis guentheri																1	1																								
Mylochromis lateristriga																			1	2	–	–	–	–	–	–	1														
Mylochromis spilostichus																	1	–	1	2*	–	–	–	–	1	–	3														
Mylochromis subocularis									1	–	1																														
Naevochromis chrysogaster																	2*	–	2	1																					
Nimbochromis fuscotaeniatus																	1	–	–	1																					
Nimbochromis livingstonii																	2	–	–	1	–	–	–	–	–	–	3														
Nimbochromis venustus																				1	–	–	–	–	–	–	1	–	–	–	–	–	–	2							
Nyassachromis leuciscus												1	–	–	–	1	–	–	1*	2	–	–	–	–	–	–	1														
Nyassachromis microcephalus											1	–	–	–	–	–	–	–	–	1*																					
Nyassachromis nigritaeniatus											1	–	–	–	–	–	1	–	1	–	–	–	–	–	–	1															
Nyassachromis purpurans											1																														
Otopharynx argyrosoma											1	–	–	–	–	1	–	–	1*																						
Otopharynx decorus											1	–	–	–	–	1	1	–	1*	–	–	–	–	–	–	2															
Otopharynx cf. *heterodon*				1	–	–	–	–	1	–	3	2	–	–	–	–	2	–	5	2	–	–	–	–	–	3															
Otopharynx heterodon				1	–	–	–	–	–	–	3	–	–	–	–	1	–	–	2	–	–	–	–	–	–	1															
Otopharynx lithobates											2*	–	–	–	–	–	–	–	–	–	–	–	–	–	–	2	1														
Otopharynx ovatus											1	–	–	–	–	–	–	–	–	1	1	–	–	–	–	–	–	–	–	–	–	–	–	2*							
Otopharynx selenurus																			1	2	–	–	–	–	–	1	–	–	1	–	–	1									
Otopharynx speciosus																			2	1	–	–	–	–	–	–	1	–	1												
Otopharynx tetraspilus																			4	2	–	–	–	–	–	–	2														
Otopharynx tetrastigma																1	1	–	4	3	–	–	–	–	–	–	5*	1	1												
Placidochromis hennydaviesae											1*																														
Placidochromis johnstoni																				2	–	–	–	–	–	1	–	1													
Placidochromis longimanus								1	–	–	4	1	–	–	–	1	4	–	3	5	–	–	–	–	2	1	6														
Placidochromis macrognathus																1	–	–	–	1																					
Placidochromis milomo											1	–	1	–	–	–	–	–	–	–	–	–	–	–	–	–	–	1	–	–	–	–	–	1							
Protomelas annectens																			1	–	–	–	–	–	1																
Protomelas fenestratus											2	2	–	–	–	5	7	–	7	1	–	–	–	–	1	1	10														
Protomelas insignis																			1	–	–	–	–	–	–	–	–	1													
Protomelas kirkii																			1	–	–	–	–	–	–	1	1														
Protomelas labridens																										1															
Protomelas ornatus																				1	–	–	–	–	–	1*															
Protomelas spilopterus																		1	1	3	–	–	–	–	–	–	1														
Protomelas taeniolatus																											1														
Protomelas triaenodon																				2	–	–	–	–	–	–	–	–	–	–	–	–	1								
Sciaenochromis ahli																	6	–	3	3	–	–	–	–	–	–	4														

Table 9 (continued). Frequency distribution of pterygiophore insertion patterns of last 4 occupied interhemal spaces

	|–/1/2/2|	|–/1/2/3|	|–/2/1/2|	|–/2/1/3|	|–/2/2/2|	|1/1/1/1|	|1/1/1/2|	|1/1/1/3|	|1/1/2/1|	|1/–/2/2|	|1/1/2/2|	|1/1/2/3|	|1/1/3/1|	|1/1/3/2|	|1/2/1/1|	|1/2/1/2|	|1/2/1/3|	|1/2/1/4|	|1/2/2/1|	|1/2/2/2|	|1/2/2/3|	|1/2/3/1|	|2/1/1/1|	|2/1/1/2|	|2/1/1/3|	|2/1/2/1|	|2/1/2/2|	|2/1/2/3|	|2/1/3/1|	|2/1/3/2|	|2/2/1/1|	|2/2/1/2|	|2/2/1/3|	|2/2/2/1|	|2/2/2/2|	|2/2/2/3|	|2/2/3/1|	|2/2/3/2|	|3/1/2/3|	|3/3/2/3|	?
Stigmatochromis woodi																1	3*	–	–	2	–	–	–	–	–	–	–	–	–	–	–	–	–	–	–	–	–	–	–	–	1
Taeniochromis holotaenia																			1																						
Taeniolethrinops praeorbitalis																											1														
Tramitichromis brevis																	1																								
Trematocranus labifer																			1	2	–	–	–	–	–	–	1														
Trematocranus microstoma																	1	–	1	1	–	–	–	–	–	–	3	–	–	–	–	1	–	2							
Trematocranus placodon											1	–	–	–	–	–	–	–	–	–	–	–	–	–	–	2															
Tyrannochromis macrostoma																			1																						
Tyrannochromis nigriventer																	1*																								
**Cyrtocarina column totals**	**–**	**–**	**–**	**2**	**–**	**–**	**–**	**1**	**2**	**–**	**35**	**14**	**2**	**–**	**–**	**19**	**52**	**1**	**69**	**70**	**2**	**1**	**–**	**–**	**5**	**21**	**81**	**12**	**5**	**–**	**–**	**6**	**4**	**18**	**1**	**–**	**–**	**–**	**–**	**–**	**2**
**Pseudocrenilabrini: Pseudotropheina**																																									
Abactochromis labrosus							1	2	1*	–	1	–	–	–	–	–	1																								
Chindongo bellicosus									2	–	3	–	–	–	–	1																									
Chindongo minutus																			1																						
Cyathochromis obliquidens											2	–	–	–	1	–	–	–	–	–	–	–	–	–	–	1															
Cynotilapia afra											1																														
Cynotilapia sp.											2	–	–	–	–	–	–	–	2																						
Genyochromis mento											1	–	–	–	2	2	–	–	1	–	–	–	–	–	–	3	–	–	–	–	–	–	–	–	–	–	–	–	–	–	1
Gephyrochromis lawsi																			1																						
Gephyrochromis moorii											1	–	–	–	–	2	–	–	–	–	–	–	–	–	–	1															
Iodotropheus sprengerae	1	–	–	–	–	–	–	–	3	–	2																														
Labeotropheus fuelleborni							1	1	–	–	11	–	–	–	1	1																									
Labeotropheus trewavasae									1	–	2																														
Labidochromis caeruleus									1																																
Labidochromis freibergi															1*																										
Labidochromis joanjohnsonae																1*																									
Labidochromis pallidus			1	–	–	–	2	–	4	1	5	–	–	–	2	8																									
Labidochromis shiranus											1																														
Labidochromis textilis											1	–	–	–	–	1*																									
Labidochromis vellicans				1	–	–	2	3	3	–	8	–	–	–	2	5	–	–	2	–	–	–	–	1	–	3															
Maylandia aurora											1*																														
Maylandia koningsi									1																																
Maylandia lanisticola																			1*																						
Maylandia zebra								1	2	–	1	–	–	–	–	3	–	–	1	–	–	–	–	–	–	1															
Melanochromis auratus	1	–	–	–	–	–	–	1	1	–	2*	–	–	–	–	–	1																								
Melanochromis chipokae									1*																																
Melanochromis loriae			1	–	–	–	–	–	–	–	1	–	–	–	–	1*	–	–	1																						
Melanochromis melanopterus									2	–	3	1	–	–	1	3	–	–	–	–	–	–	–	–	–	1															
Melanochromis simulans																										1															
Melanochromis cf. *vermivorus*									1	–	1																														
Petrotilapia genalutea																1																									
Petrotilapia nigra											1																														
Petrotilapia tridentiger											1																														
Petrotilapia sp.																3	–	–	–	–	–	–	–	–	–	1															
Pseudotropheus elegans											1*																														
Pseudotropheus interruptus											2*																														
Pseudotropheus johannii				1	–	–	1	4	3	–	14*	–	–	–	2	4	–	–	2		–	–	–	–	–	6															
Pseudotropheus livingstonii											3	–	–	–	–	2	–	–	1	–	–	–	–	–	–	–	1														
Pseudotropheus lucerna											1																														
Tropheops tropheops								1	–	–	1	1	–	–	1	–	–	–	1	–	–	–	–	–	–	2															

Table 9 (continued). Frequency distribution of pterygiophore insertion patterns of last 4 occupied interhemal spaces

	|–/1/2/2|	|–/1/2/3|	|–/2/1/2|	|–/2/1/3|	|–/2/2/2|	|1/1/1/1|	|1/1/1/2|	|1/1/1/3|	|1/1/2/1|	|1/–/2/2|	|1/1/2/2|	|1/1/2/3|	|1/1/3/1|	|1/1/3/2|	|1/2/1/1|	|1/2/1/2|	|1/2/1/3|	|1/2/1/4|	|1/2/2/1|	|1/2/2/2|	|1/2/2/3|	|1/2/3/1|	|2/1/1/1|	|2/1/1/2|	|2/1/1/3|	|2/1/2/1|	|2/1/2/2|	|2/1/2/3|	|2/1/3/1|	|2/1/3/2|	|2/2/1/1|	|2/2/1/2|	|2/2/1/3|	|2/2/2/1|	|2/2/2/2|	|2/2/2/3|	|2/2/3/1|	|2/2/3/2|	|3/1/2/3|	|3/3/2/3|	?
**Pseudocrenilabrini: Rhamphochromina**																																									
Diplotaxodon argenteus																				1	1	–	–	–	–	–	1*	1	–	–	–	–	1	1	3	1					
Diplotaxodon ecclesi																																			1*						
Diplotaxodon greenwoodi																											1*														
Diplotaxodon limnothrissa																1	–	–	2	2	1	1	–	–	–	–	2	–	–	–	–	–	–	1							
Pallidochromis tokolosh																				1	–	–	–	–	–	–	–	1	–	–	–	–	–	2							
Rhamphochromis brevis																											1*	–	1												
Rhamphochromis esox																			2																						
Rhamphochromis woodi																												1													
**Rhamphochromina column totals**	**–**	**–**	**–**	**–**	**–**	**–**	**–**	**–**	**–**	**–**	**–**	**–**	**–**	**–**	**–**	**1**	**–**	**–**	**4**	**4**	**2**	**1**	**–**	**–**	**–**	**–**	**5**	**3**	**1**	**–**	**–**	**–**	**1**	**4**	**4**	**1**	**–**	**–**	**–**	**–**	**–**
**Cyrtocarina column totals**	**–**	**–**	**–**	**2**	**–**	**–**	**–**	**1**	**2**	**–**	**35**	**14**	**2**	**–**	**–**	**19**	**52**	**1**	**69**	**70**	**2**	**1**	**–**	**–**	**5**	**21**	**81**	**12**	**5**	**–**	**–**	**6**	**4**	**18**	**1**	**–**	**–**	**–**	**–**	**–**	**2**
**Pseudotropheina column totals**	**2**	**–**	**2**	**2**	**–**	**–**	**7**	**13**	**26**	**1**	**74**	**2**	**–**	**–**	**13**	**38**	**2**	**–**	**14**	**–**	**–**	**–**	**–**	**1**	**–**	**20**	**1**	**–**	**–**	**–**	**–**	**–**	**–**	**–**	**–**	**–**	**–**	**–**	**–**	**–**	**1**
**Rhamphochromina column totals**	**–**	**–**	**–**	**–**	**–**	**–**	**–**	**–**	**–**	**–**	**–**	**–**	**–**	**–**	**–**	**1**	**–**	**–**	**4**	**4**	**2**	**1**	**–**	**–**	**–**	**–**	**5**	**3**	**1**	**–**	**–**	**–**	**1**	**4**	**4**	**1**	**–**	**–**	**–**	**–**	**–**
