# Supplementary material for: African cichlid fishes: morphological data and taxonomic insights from a genus-level survey of supraneurals, pterygiophores, and vertebral counts (Ovalentaria, Blenniiformes, Cichlidae, Pseudocrenilabrinae)
Source: Biodivers Data J. 2024 Oct 18;12:e130707. doi: 10.3897/BDJ.12.e130707 (PMC11512106; doi:10.3897/BDJ.12.e130707)
Supplement: Supplementary material 11 — Table S10. [file bdj-12-e130707-s011.pdf]

Species, Museum, Catalog number / ID<br>Vertebral count	1	2	3	4	5	6	7	8	9	10	11	12	13	14	15	16	17	18	19	20	21	22	23	24	25	26	27	28	29	30	31	32	33	34	35	36	37	38	39	40
POLYCENTRIDAE																																								
Afronandus sheljuzhkoi USNM 372183	–	0	0-1	2	1	1	1	1	1	–	1	1	1	1	2	1	2	2	1	2	–	–	–	–	–	c														
11+15												2	1	1	1	1	2	1	–	–	–	–	–	–	–	c														
Monocirrhus polyacanthus UF 120003	0-0	0-1	1	2	1	1	1	1	–	1	1	2	1	2	2	2	3	2?	2	–	–	c																		
9+13										3	2	2	2	2	2	3	3	2	3?	–	–	c																		
USNM 103840	0	0	0-1	2	1	1	1	1	1	1	1	2	1	2	2	2	2	2	3	–	–	–	c																	
10+13											3	2	2	2	2	2	2	2	3	2	–	–	c																	
Polycentropsis abbreviata CUMV 93208	0	0-1	2	1	1	1	1	1	–	1	1	1	1	2	1	2	2	3	2	–	–	c																		
9+13										2	1	2	1	1	2	2	2	2	2	–	–	c																		
USNM 302514	0	0	0-1	2	1	1	1	1	1	1	1	1	1	1	2	2	2	2	3	–	–	–	c	3	1	13														
CICHLIDAE: Etroplinae																																								
India																																								
Etroplus suratensis TCWC 11237.06	0	0-1	1	1	1	1	1	1	1	1	1	1	1	1	1	1	1	1	2	1	2	1	2	2	2	3	–	–	–	–	c									
16+15																	5	2	2	2	1	2	1	2	2	3	–	–	–	–	c									
Pseudetroplus maculatus FMNH 17028 L1	0	0-1	1	1	1	1	1	1	1	1	1	1	1	1	1	1	1	1	2	2	2	2	1	–	–	–	–	c												
14+14															6	2	1	2	2	1	2	3	–	–	–	–	–	c												
L2	0	0-1	1	1	1	1	1	1	1	1	1	1	1	1	1	1	2	1	2	2	1	2	–	–	–	–	c													
13+14														8	–	1	2	1	2	2	2	1	–	–	–	–	c													
L3	0	0-1	1	1	1	1	1	1	1	1	1	1	1	1	1	1	2	1	2	1	2	2	–	–	–	–	c													
12+15													5	1	2	1	2	1	2	2	2	2	–	–	–	–	c													
R1	0	0-1	1	1	1	1	1	1	1	1	1	1	1	1	1	1	2	1	2	2	2	1	–	–	–	–	c													
13+14														6	2	1	1	2	2	2	2	2	–	–	–	–	c													
R2	0-0	1	1	1	1	1	1	1	1	1	1	1	1	1	1	1	1	2	2	2	1	2	–	–	–	–	c													
12+15													4	1	2	1	2	1	2	2	2	1	–	–	–	–	c													
R3	0-0	1	1	1	1	1	1	1	1	1	1	1	1	1	1	1	2	1	2	1	2	2	–	–	–	–	c													
12+15													4	1	2	1	2	1	2	2	2	1	–	–	–	–	c													
MCZ 4311	0	0-1	1	1	1	1	1	1	1	1	1	1	1	1	1	1	1	1	1	2	1	2	2	2	1	–	–	–	c											
14+15															5	–	2	1	2	1	1	2	2	2	1	–	–	–	c											
Madagascar																																								
Paretroplus polyactis FMNH 6156	0	0-1	1	1	1	1	1	1	1	1	1	1	1	1	1	1	1	1	1	2	1	2	2	1	2	2	2	1	–	–	–	–	–	c						
15+19																2	1	2	1	1	1	2	2	1	2	2	2	2	–	–	–	–	–	c						
Ptychochrominae																																								
Madagascar																																								
Katria katria UMMZ 250284	0-0	1	2	1	1	1	1	1	1	1	1	1	1	1	1	1	1	2	2	1	–	–	–	–	–	–	–	–	–	–	c									
15+16																2	1	1	2	2	1	–	–	–	–	–	–	–	–	–	c									
Oxylapia polli UMMZ 250283	0	0	2	1	1	1	1	1	1	1	1	1	1	1	1	1	1	2	1	2	2	–	–	–	–	–	–	–	–	–	–	–	c							
14+19															2	–	1	1	1	2	1	1	–	–	–	–	–	–	–	–	–	–	c							
Paratilapia polleni AMNH 11687 a	0	0	2	2	1	1	1	1	1	1	1	1	1	1	1	1	2	1	3	–	–	–	–	–	–	–	c													
12+15													1	1	2	1	2	2	2	–	–	–	–	–	–	–	c													
b	0	0-1	1	2	1	1	1	1	1	1	1	1	1	1	1	1	2	2	2	–	–	–	–	–	–	–	c													
12+15													1	1	1	2	2	1	2	2	–	–	–	–	–	–	c													
c	0	0	3	1	1	1	1	1	1	1	1	1	1	1	2	1	2	1	2	–	–	–	–	–	–	–	c													
12+15													1	1	2	1	2	2	1	–	–	–	–	–	–	–	c													
d	0	0	2	2	1	1	1	1	1	1	1	1	1	1	1	2	1	2	2	–	–	–	–	–	–	–	c													
12+15													1	1	2	1	2	2	2	–	–	–	–	–	–	–	c													
e	0	0-0	2	2	2	1	1	1	1	1	1	1	1	1	1	2	1	3	–	–	–	–	–	–	–	–	c													
12+15													1	1	2	1	2	2	2	–	–	–	–	–	–	–	c													
AMNH 11689 left	0	0-0	2	1	1	1	1	1	1	1	1	1	1	1	1	1	2	1	1	2	–	–	–	–	–	–	–	c												
13+15														2	1	2	2	1	2	1	–	–	–	–	–	–	–	c												
right upper	0	0-0	2	1	1	1	1	1	1	1	1	1	1	1	1	1	2	2	2	–	–	–	–	–	–	–	–	c												
12+16													1	1	1	2	2	2	1	1	–	–	–	–	–	–	–	c												
right lower	0	0-0-1	2	1	1	1	1	1	1	1	1	1	1	1	1	2	1	1	2	–	–	–	–	–	–	–	c													
12+15													1	1	2	1	2	1	2	1	–	–	–	–	–	–	c													
AMNH 18842	0	0-1	2	1	1	1	1	1	1	1	1	1	1	1	1	1	2	2	2	–	–	–	–	–	–	–	c													
12+15													1	1	1	2	2	2	2	–	–	–	–	–	–	–	c													
AMNH 11709	0	0-0-1	2	1	1	1	1	1	1	1	1	1	–	2	1	1	2	2	2	–	–	–	–	–	–	–	c													
12+15													1	1	1	2	2	1	2	2	–	–	–	–	–	–	c													
	0	0-1	3	1	1	1	1	1	1	1	1	–	1	1	2	1	2	2	2	–	–	–	–	–	–	–	c													
12+15													1	1	2	1	2	1	2	1	–	–	–	–	–	–	c													
Ptychochromis oligacanthus YPM 023177	0-0	0-1	2	1	1	1	1	1	1	1	1	1	1	1	1	1	1	2	1	2	1	–	–	–	–	–	–	c												
14+14															1	1	1	2	1	2	1	–	–	–	–	–	–	c												
UMMZ 237497 L1	0-0	0-1	2	1	1	1	1	1	1	1	1	1	1	1	1	1	1	2	1	2	2	–	–	–	–	–	–	c												
14+14															1	1	1	2	1	2	1	–	–	–	–	–	–	c												
L2	0-0	0-2	1	1	1	1	1	1	1	1	1	1	1	1	1	1	1	2	1	2	1	–	–	–	–	–	–	c												
14+14															1	1	1	2	1	2	1	–	–	–	–	–	–	c												

Species, Museum, Catalog number / ID Vertebral count	1	2	3	4	5	6	7	8	9	10	11	12	13	14	15	16	17	18	19	20	21	22	23	24	25	26	27	28	29	30	31	32	33	34	35	36	37	38	39	40
L3	0	0-0-2	1	1	1	1	1	1	1	1	1	1	1	1	1	1	2	1	2	2	1	–	–	–	–	–	–	c												
14+14															1	1	1	2	1	2	2	–	–	–	–	–	–	c												
L4	0-0	0-2	1	1	1	1	1	1	1	1	1	1	1	1	1	1	1	2	2	1	1	–	–	–	–	–	–	c												
14+14															1	1	1	1	2	2	1	–	–	–	–	–	–	c												
L5	0-0	0-2	1	1	1	1	1	1	1	1	1	1	1	1	1	1	2	1	2	1	1	–	–	–	–	–	–	c												
14+14															1	1	2	1	2	1	1	–	–	–	–	–	–	c												
R1	0	0-2	1	1	1	1	1	1	1	1	1	1	1	1	1	1	2	1	2	2	–	–	–	–	–	–	–	c												
13+15														1	–	1	2	1	2	2	–	–	–	–	–	–	–	c												
R2	0-0	0-1	2	1	1	1	1	1	1	1	1	1	1	1	1	1	1	2	2	2	1	–	–	–	–	–	–	c												
14+14															1	1	1	1	2	2	2	–	–	–	–	–	–	c												
R3	0	0-2	1	1	1	1	1	1	1	1	1	1	1	1	1	1	1	2	2	2	1	–	–	–	–	–	–	c												
14+14															1	1	1	1	1	2	2	–	–	–	–	–	–	c												
R4	0-0	0-1	2	1	1	1	1	1	1	1	1	1	1	1	1	1	1	2	2	1	2	–	–	–	–	–	–	c												
14+14															1	1	1	1	2	2	2	–	–	–	–	–	–	c												
R5	0-0	0-2	1	1	1	1	1	1	1	1	1	1	1	1	1	1	1	2	2	1	2	–	–	–	–	–	–	c												
14+14															1	1	1	1	2	2	1	–	–	–	–	–	–	c												
Ptychochromoides betsileanus UMMZ 238114	0	–	0-1	2	1	1	1	1	1	1	1	1	1	1	1	1	1	2	1	2	2	1	–	–	–	–	–	–	–	c										
16+14																	1	2	2	2	2	1	–	–	–	–	–	–	–	c										
	0	–	0-1	2	1	1	1	1	1	1	1	1	1	1	1	1	1	2	1	2	2	2	–	–	–	–	–	–	–	–	c									
15+16																1	–	2	2	2	3	–	–	–	–	–	–	–	–	–	c									
	0	0	2	1	1	1	1	1	1	1	1	1	1	1	1	1	1	2	2	1	2	2	–	–	–	–	–	–	–	c										
15+15																2	–	2	2	1	2	2	–	–	–	–	–	–	–	c										
Cichlinae																																								
**Neotropics**																																								
**Astronotini**																																								
Astronotus ocellatus FMNH 85660	0	0-1	1	1	1	1	1	1	1	1	1	1	1	1	1	1	2	1	1	2	1	2	1	2	1	2	1	–	–	–	–	c								
15+17																1	1	2	1	1	2	2	1	2	1	2	–	–	–	–	–	c								
FMNH 105301	0	0-1	1	1	1	1	1	1	1	1	1	1	1	1	1	1	1	2	1	2	1	2	1	2	1	2	1	–	–	–	–	c								
15+17																1	1	2	1	1	2	2	1	1	2	2	–	–	–	–	–	c								
	0	0-0-1	1	1	1	1	1	1	1	1	1	1	1	1	1	1	1	2	1	2	1	2	1	2	1	2	1	–	–	–	–	c								
15+17																1	1	2	1	1	2	1	2	1	2	2	–	–	–	–	–	c								
**Chaetobranchini**																																								
Chaetobranchopsis orbicularis UF 188930	0	0-2	1	1	1	1	1	1	1	1	1	1	1	1	1	1	1	2	1	2	2	2	1	–	–	–	c													
13+14														2	**3**	2	2	2	1	2	2	2	2	1	–	–	c													
Chaetobranchus flavescens FMNH 101872	0	0-2	1	1	1	1	1	1	1	1	1	1	1	1	1	1	1	2	2	2	2	–	–	–	–	c														
13+13														1	1	1	2	1	2	2	2	–	–	–	–	c														
	–	0-2	1	1	1	1	1	1	1	1	1	1	1	1	1	1	2	1	2	2	2	–	–	–	–	c														
13+13														1	1	1	1	2	2	2	2	–	–	–	–	c														
FMNH 111427	0	0-2	1	1	1	1	1	1	1	1	1	1	1	1	1	1	2	1	2	2	2	–	–	–	–	c														
13+13														1	1	1	1	2	1	2	3	–	–	–	–	c														
	0	0-2	1	1	1	1	1	1	1	1	1	1	1	1	1	1	2	1	2	2	2	–	–	–	–	c														
13+13														1	1	1	1	2	1	2	3	–	–	–	–	c														
**Cichlasomatini**																																								
Aequidens tetramerus CAS SU 17302	0	0-2	1	1	1	1	1	1	1	1	1	1	1	1	1	1	2	1	2	2	1	–	–	–	–	c														
13+13														1	1	1	1	2	1	1	–	–	–	–	–	c														
Andinoacara pulcher ANSP 195366	0	0-2	1	1	1	1	1	1	1	1	1	1	1	1	1	2	1	2	2	1	–	–	–	–	c															
12+13													1	1	1	1	1	2	2	–	–	–	–	–	c															
Bujurquina vittata ANSP 124129	0	0-2	1	1	1	1	1	1	1	1	1	1	1	1	1	2	1	2	1	–	–	–	–	c																
13+11														1	1	1	2	2	1?	–	–	–	–	c																
Cichlasoma dimerus ANSP 124090	0	0-2	1	1	1	1	1	1	1	1	1	1	1	1	1	2	1	2	2	1	–	–	–	–	c															
13+12														1	1	2	2	1	2	1	–	–	–	–	c															
Cichlasoma taenia ANSP 76395	0	0-2	1	1	1	1	1	1	1	1	1	1	1	1	1	1	2	1	2	1	2	1	–	–	–	c														
13+13														1	1	1	2	1	1	2?	1?	–	–	–	–	c														
Krobia guianensis ANSP 179496	0	0-2	1	1	1	1	1	1	1	1	1	1	1	1	2	1	2	2	2	–	–	–	–	–	c															
12+13													1	1	1	1	2	2	1	–	–	–	–	–	c															
**Cichlini**																																								
Cichla ocellaris UF 244243	0	0-1	2	1	1	1	1	1	1	1	1	1	1	1	1	1	2	1	1	2	2	2	2	2	2	–	–	–	–	–	–	–	–	–	–	c				
18+18																			2	–	2	2	2	2	2	–	–	–	–	–	–	–	–	–	–	c				
Cichla orinocensis FMNH 103505 top of 3	0	0-1	2	1	1	1	1	1	1	–	1	2	1	1	1	1	1	1	2	2	1	2	2	2	2	–	–	–	–	–	–	–	–	–	–	c				
18+18																			1	1	2	2	2	2	2	1	–	–	–	–	–	–	–	–	–	c				
mid of 3	0	0-1	2	1	1	1	1	1	1	1	1	1	1	1	1	1	1	2	1	2	2	1	2	2	2	–	–	–	–	–	–	–	–	–	c					
19+16																				2	2	1	2	2	2	1	–	–	–	–	–	–	–	–	c					
low of 3	0	0-1	2	1	1	1	1	1	1	1	1	1	1	1	1	1	1	1	2	2	1	2	2	2	2	–	–	–	–	–	–	–	–	–	–	c				
18+18																			1	1	2	2	2	2	2	–	–	–	–	–	–	–	–	–	–	c				

Species, Museum, Catalog number / ID<br>Vertebral count	1	2	3	4	5	6	7	8	9	10	11	12	13	14	15	16	17	18	19	20	21	22	23	24	25	26	27	28	29	30	31	32	33	34	35	36	37	38	39	40
**Geophagini**																																								
Biotodoma cupido UF 100668	0	0-2	1	1	1	1	1	1	1	1	1	1	1	1	1	1	1	2	2	2	1	–	–	–	–	–	–	c												
14+14															1	1	1	2	2	2	1	–	–	–	–	–	–	c												
FMNH 53843	0	0-2	1	1	1	1	1	1	1	1	1	1	1	1	1	1	1	2	2	2	2	–	–	–	–	–	–	c												
14+14															1	1	1	2	2	2	1	–	–	–	–	–	–	c												
	0	0-2	1	1	1	1	1	1	1	1	1	1	1	1	1	1	1	2	2	2	–	–	–	–	–	–	–	c												
14+14															1	1	2	2	2	1	–	–	–	–	–	–	–	c												
	0	0-2	1	1	1	1	1	1	1	1	1	1	1	1	1	1	1	2	2	2	1	–	–	–	–	–	–	c												
14+14															2	1	1	1	2	2	–	–	–	–	–	–	–	c												
Geophagus crassilabris UF 240894	0-0	2	1	1	1	1	1	1	1	1	1	1	1	1	1	1	1	1	2	2	2	1	–	–	–	–	–	–	c											
14+15															1	1	1	1	1	2	1	–	–	–	–	–	–	–	c											
Guianacara owroewefi FMNH 117023 L1	0	0-2	1	1	1	1	1	1	1	1	1	1	1	1	1	1	2	2	1	3	–	–	–	–	–	–	c													
13+14														1	1	1	1	2	1	2	–	–	–	–	–	–	c													
L2	0	0-2	1	1	1	1	1	1	1	1	1	1	1	1	1	2	1	2	2	2	–	–	–	–	–	–	c													
13+14														1	1	1	1	2	3	–	–	–	–	–	–	–	c													
NOT USED - Unclear R1																																								
R2	0	0-2	1	1	1	1	1	1	1	1	1	1	1	1	1	1	2	1	2	3	–	–	–	–	–	–	c													
13+14														1	1	1	1	2	3	–	–	–	–	–	–	–	c													
R3	0	0-2	1	1	1	1	1	1	1	1	1	1	1	1	1	1	2	1	2	2	1	–	–	–	–	–	c													
13+14														1	1	1	1	1	2	2	–	–	–	–	–	–	c													
FMNH 117029 L1	0	0-2	1	1	1	1	1	1	1	1	1	1	1	1	1	1	2	2	1	3	–	–	–	–	–	–	c													
13+14														1	1	1	1	2	2	1	–	–	–	–	–	–	c													
L2	0	0-2	1	1	1	1	1	1	1	1	1	1	1	1	1	1	2	2	2	2	–	–	–	–	–	–	c													
13+14														1	1	1	1	2	2	1	–	–	–	–	–	–	c													
R1	0	0-2	1	1	1	1	1	1	1	1	1	1	1	1	1	1	2	1	2	2	1	–	–	–	–	–	c													
13+14														1	1	1	1	2	1	2	–	–	–	–	–	–	c													
R2	0	0-2	1	1	1	1	1	1	1	1	1	1	1	1	1	1	2	2	1	2	1	–	–	–	–	–	c													
13+14														1	1	1	1	2	2	1	–	–	–	–	–	–	c													
Saxatilia frenata USNM 1111 holotype	–	2	1	1	1	1	1	1	1	1	1	1	1	1	1	1	1	1	1	1	1	1	1	1	2	2	1	2	–	–	–	–	–	–	c					
20+15																					1	1	2	1	2	1	2	–	–	–	–	–	–	–	c					
Saxatilia lepidota NHMUK 1935.6.4.416–419 1	–	2	1	1	1	1	1	1	1	1	1	1	1	1	1	1	1	1	1	1	1	1	2	1	2	2	1	–	–	–	–	–	c							
(paratypes of *Crenicichla edithae* =*S.lepidota*) 18+15																			1	1	1	1	2	2	2	1	–	–	–	–	–	–	c							
2	–	2	1	1	1	1	1	1	1	1	1	1	1	1	1	1	1	1	1	1	1	1	1	2	2	2	1	–	–	–	–	–	c							
18+15																			1	1	1	2	1	2	2	–	–	–	–	–	–	–	c							
**Heroini**																																								
Amphilophus citrinellus USNM 44180	0	0-2	1	1	1	1	1	1	1	1	1	1	1	1	1	1	1	1	1	1	2	2	2	2	–	–	–	–	–	–	c									
14+17															2	–	1	1	1	2	1	1	2	3	–	–	–	–	–	–	c									
Hypselacara coryphaenoides USNM 206097	0	0-2	1	1	1	1	1	1	1	1	1	1	1	1	1	1	1	1	2	1	2	2	2	–	–	–	–	–	c											
(holotype of *Chuco axelrodi* = *H. coryph.*) 14+15															2	1	1	1	1	2	1	2	2	–	–	–	–	–	c											
USNM 206098 1	0	0-2	1	1	1	1	1	1	1	1	1	1	1	1	1	1	1	1	2	1	1	2	2	1	–	–	–	–	c											
(paratypes of *Chuco axelrodi* = *H. coryph.*) 14+15															–	3	1	1	1	1	2	2	2	–	–	–	–	–	c											
2	0	0-2	1	1	1	1	1	1	1	1	1	1	1	1	1	1	1	1	2	1	2	2	2	–	–	–	–	–	c											
14+15															2	1	1	1	1	1	2	2	2	–	–	–	–	–	c											
3	0	0-2	1	1	1	1	1	1	1	1	1	1	1	1	1	1	1	1	2	1	2	2	2	–	–	–	–	–	c											
14+15															2	1	1	1	1	2	1	2	2	–	–	–	–	–	c											
4	0	0-2	1	1	1	1	1	1	1	1	1	1	1	1	1	1	1	1	2	1	2	2	2	–	–	–	–	–	c											
14+15															2	1	1	1	1	2	1	2	2	–	–	–	–	–	c											
**Retroculini**																																								
Retroculus lapidifer USNM 152111	0	0-2	1	1	1	1	1	1	1	1	1	1	1	1	1	1	1	1	1	1	2	1	2	1	–	–	–	–	–	–	–	–	c							
(holotype of *R. boulengeri* = *R. lapidifer*) 14+19															1	–	1	1	1	1	2	1	–	–	–	–	–	–	–	–	–	–	c							
Pseudocrenilabrinae																																								
**Middle East**																																								
**Oreochromini**																																								
Iranocichla hormuzensis NHMUK 1981.1.12.1-2 paratypes	0	1	2	1	1	1	1	1	1	1	1	1	1	1	1	1	1	2	1	1	3	1	–	–	–	–	–	–	c											
15+14																1	–	1	2	2	2	1	–	–	–	–	–	–	c											
	0	2	1	1	1	1	1	1	1	1	1	1	1	1	1	1	1	2	1	2	2	1	–	–	–	–	–	–	c											
15+14																1	–	2	1	2	2	1	–	–	–	–	–	–	c											
Tristramella sacra UMMZ 213421	0	2	1	1	1	1	1	1	1	1	1	1	1	1	1	1	1	2	1	2	2	–	–	–	–	–	–	–	–	c										
15+15																1	2	1	2	1	3	–	–	–	–	–	–	–	–	c										
Tristramella simonis AMNH 225653 1	0	2	1	1	1	1	1	1	1	1	1	1	1	1	1	1	1	2	2	1	2	–	–	–	–	–	–	–	c											
15+14																1	–	2	1	2	3	–	–	–	–	–	–	–	c											
2	0	2	1	1	1	1	1	1	1	1	1	1	1	1	1	1	1	2	1	2	1	–	–	–	–	–	–	–	c											
14+15															1	–	2	1	2	2	1	–	–	–	–	–	–	–	c											
3	0	2	1	1	1	1	1	1	1	1	1	1	1	1	1	1	1	2	1	2	1	–	–	–	–	–	–	–	c											
14+15															1	–	2	1	2	2	1	–	–	–	–	–	–	–	c											

Species, Museum, Catalog number / ID<br>Vertebral count	1	2	3	4	5	6	7	8	9	10	11	12	13	14	15	16	17	18	19	20	21	22	23	24	25	26	27	28	29	30	31	32	33	34	35	36	37	38	39	40
4	0	2	1	1	1	1	1	1	1	1	1	1	1	1	1	1	1	2	1	2	2	–	–	–	–	–	–	–	c											
15+14																1	2	1	2	2	1	–	–	–	–	–	–	–	c											
**Pseudocrenilabrini**																																								
Astatotilapia flavijosephi NHMUK 1898.12.5.5-6	0	2	1	1	1	1	1	1	1	1	1	1	1	1	1	1	2	1	3	–	–	–	–	–	–	–	c													
paralectotypes 13+14														1	1	1	2	2	2	–	–	–	–	–	–	–	c													
	0	2	1	1	1	1	1	1	1	1	1	1	1	1	1	1	2	1	3	–	–	–	–	–	–	–	c													
12+15													1	–	2	1	1	2	2	1	–	–	–	–	–	–	c													
NHMUK 1949.9.16.398	0	2	1	1	1	1	1	1	1	1	1	1	1	1	1	1	2	1	2	1	–	–	–	–	–	–	c													
13+14														1	1	1	2	2	2	1	–	–	–	–	–	–	c													
	0	2	1	1	1	1	1	1	1	1	1	1	1	1	1	1	1	2	2	1	–	–	–	–	–	–	c													
13+14														1	1	1	2	2	2	–	–	–	–	–	–	–	c													
African Riverine																																								
**Chromidotilapiini**																																								
Benitochromis batesii NHMUK 1904.2.29.57–59 L1	0	2	1	1	1	1	1	1	1	1	1	1	1	1	1	1	2	2	2	1	–	–	–	–	–	c														
13+13														1	1	1	1	2	2	–	–	–	–	–	–	c														
L2	0	2	1	1	1	1	1	1	1	1	1	1	1	1	1	1	2	2	2	2	–	–	–	–	–	c														
13+13														1	1	1	2	1	2	–	–	–	–	–	–	c														
L3	0	2	1	1	1	1	1	1	1	1	1	1	1	1	1	1	2	2	2	2	–	–	–	–	–	c														
13+13														1	1	1	1	2	2	1	–	–	–	–	–	c														
R1	0	2	1	1	1	1	1	1	1	1	1	1	1	1	1	1	2	1	2	3	–	–	–	–	–	c														
13+13														1	1	1	1	2	2	–	–	–	–	–	–	c														
R2	0	2	1	1	1	1	1	1	1	1	1	1	1	1	1	1	2	2	2	2	–	–	–	–	–	c														
13+13														–	1	1	1	2	2	1	–	–	–	–	–	c														
R3	0	2	1	1	1	1	1	1	1	1	1	1	1	1	1	1	2	1	2	3	–	–	–	–	–	c														
13+13														1	1	1	2	1	2	–	–	–	–	–	–	c														
Benitochromis finleyi USNM 205627	0	2	1	1	1	1	1	1	1	1	1	1	1	1	1	1	1	2	2	2	1	–	–	–	–	–	c													
14+13															1	1	1	1	2	2	–	–	–	–	–	–	c													
Chromidotilapia guntheri NHMUK 1959.8.18.178	0	2	1	1	1	1	1	1	1	1	1	1	1	1	1	1	1	2	2	1	3	–	–	–	–	–	c													
14+13															1	–	1	1	2	2	1	–	–	–	–	–	c													
NHMUK 1961.10.18.6	0	2	1	1	1	1	1	1	1	1	1	1	1	1	1	1	1	2	1	3	2	1	–	–	–	–	c													
14+13															–	1	1	1	2	2	1	–	–	–	–	–	c													
Chromidotilapia kingsleyae CUMV 96466	0	2	1	1	1	1	1	1	1	1	1	1	1	1	1	1	2	2	3	2	–	–	–	–	–	c														
13+13														1	1	1	2	2	2	1	–	–	–	–	–	c														
NHMUK 1867.5.3.1 paralectotype	0	2	1	1	1	1	1	1	1	1	1	1	1	1	1	1	2	2	2	2	1	–	–	–	–	–	c													
14+13															1	1	2	1	3	1	–	–	–	–	–	–	c													
NHMUK 1912.4.1.517-525 top row L	0	2	1	1	1	1	1	1	1	1	1	1	1	1	1	1	2	2	2	1?	–	–	–	–	–	c														
13+13														1	1	1	2	2	1	–	–	–	–	–	–	c														
top row R	0	2	1	1	1	1	1	1	2	–	1	1	1	1	1	2	1	2	2	2	–	–	–	–	–	c														
13+13														1	1	1	1	2	2	–	–	–	–	–	–	c														
middle row mid	0	2	1	1	1	1	1	1	1	1	1	1	1	1	1	1	2	2	2	2	–	–	–	–	–	c														
14+12															1	2	1	2	2	–	–	–	–	–	–	c														
middle row R	0	2	1	1	1	1	1	1	1	1	1	1	1	1	1	2	1	2	2	2	–	–	–	–	–	c														
13+13														1	1	1	1	2	3	–	–	–	–	–	–	c														
lower row L	0	2	1	1	1	1	1	1	1	1	1	1	1	1	1	2	1	2	3	1	–	–	–	–	–	c														
13+13														1	1	1	2	2	1	–	–	–	–	–	–	c														
lower row mid	0	2	1	1	1	1	1	1	1	1	1	1	1	1	1	2	1	2	2	1	–	–	–	–	c															
13+12														1	1	2	1	2	1?	–	–	–	–	–	c															
lower row R	0	2	1	1	1	1	1	1	1	1	1	1	1	1	1	2	2	2	2?	–	–	–	–	–	–	c														
13+13?														1	1	1	1	2	2?	–	–	–	–	–	–	c														
Congochromis robustus RMCA 135706 holotype	0	2	1	1	1	1	1	1	1	1	1	1	1	1	1	1	1	1	2	1	2	1	–	–	–	–	c													
14+13															1	–	1	2	1	1	–	–	–	–	–	–	c													
Congochromis squamiceps CUMV 96716	0	2	1	1	1	1	1	1	1	1	1	1	1	1	1	1	1	1	2	2	2	–	–	–	–	c														
14+12															1	1	1	1	2	2?	–	–	–	–	–	c														
NHMUK 1902.4.14.11 syntype	0	2	1	1	1	1	1	1	1	1	1	1	1	1	1	1	1	1	2	1	2	1	–	–	–	–	–	c												
14+14															1	–	1	1	2	2	1	–	–	–	–	–	–	c												
Divandu albimarginatus AMNH 232347	0	2	1	1	1	1	1	1	1	1	1	1	1	1	1	2	1	2	3	2	–	–	–	–	–	c														
13+13														1	1	1	1	2	3	–	–	–	–	–	–	c														
	0	2	1	1	1	1	1	1	1	1	1	1	1	1	1	2	2	2	3	1	–	–	–	–	c															
12+13													1	1	1	1	2	2	2	–	–	–	–	–	c															
	0	2	1	1	1	1	1	1	1	1	1	1	1	1	1	2	1	2	2	3	–	–	–	–	–	c														
13+13														1	1	1	1	2	2	–	–	–	–	–	–	c														
	0	2	1	1	1	1	1	1	1	1	1	1	1	1	1	2	1	2	2	3	–	–	–	–	–	c														
13+13														1	1	1	1	2	2	1	–	–	–	–	–	c														

**Species, Museum, Catalog number / ID**<br>**Vertebral count**	1	2	3	4	5	6	7	8	9	10	11	12	13	14	15	16	17	18	19	20	21	22	23	24	25	26	27	28	29	30	31	32	33	34	35	36	37	38	39	40
Limbochromis robertsi USNM 214090 1	0	2	1	1	1	1	1	1	1	1	1	1	1	1	1	1	1	2	2	3	1	–	–	–	–	–	c													
13+14														–	1	1	1	1	2	2	–	–	–	–	–	–	c													
2	0	2	1	1	1	1	1	1	1	1	1	1	1	1	1	1	1	2	2	2	1	–	–	–	–	–	c													
13+14														1	1	1	1	2	2	1	–	–	–	–	–	–	c													
3	0	2	1	1	1	1	1	1	1	1	1	1	1	1	1	1	1	2	2	2	2?	–	–	–	–	–	c													
13+14														2	–	1	1	2	1	2	–	–	–	–	–	–	c													
4	0	2	1	1	1	1	1	1	1	1	1	1	1	1	1	1	1	2	2	2	1?	–	–	–	–	–	c													
13+14														2	–	1	1	2	2	1	–	–	–	–	–	–	c													
5	0	2	1	1	1	1	1	1	1	1	1	1	1	1	1	1	1	2	2	2	1?	–	–	–	–	–	c													
13+14														2	–	1	2	1	2	1	–	–	–	–	–	–	c													
6	0	2	1	1	1	1	1	1	1	1	1	1	1	1	1	1	1	2	2	2	1	–	–	–	–	–	c													
13+14														1	1	1	1	2	2	1	–	–	–	–	–	–	c													
7	0	2	1	1	1	1	1	1	1	1	1	1	1	1	1	1	1	2	2	2	1?	–	–	–	–	–	c													
13+14														1	1	1	2	1	2	1	–	–	–	–	–	–	c													
8	0	2	1	1	1	1	1	1	1	1	1	1	1	1	1	1	1	2	2	2	1	–	–	–	–	–	c													
13+14														1	1	1	2	1	2	1	–	–	–	–	–	–	c													
9	0	2	1	1	1	1	1	1	1	1	1	1	1	1	1	1	1	2	1	2	2	–	–	–	–	–	c													
13+14														1	1	1	1	2	2	1	–	–	–	–	–	–	c													
10	0	2	1	1	1	1	1	1	1	1	1	1	1	1	1	1	1	2	1	2	2	–	–	–	–	–	c													
13+14														1	1	1	1	2	2	1	–	–	–	–	–	–	c													
11	0	2	1	1	1	1	1	1	1	1	1	1	1	1	1	1	1	2	2	2	1	–	–	–	–	–	c													
13+14														1	1	1	1	2	2	1	–	–	–	–	–	–	c													
12	0	2	1	1	1	1	1	1	1	1	1	1	1	1	1	1	1	2	1	2	2	–	–	–	–	–	c													
13+14														1	1	1	1	2	2	1	1	–	–	–	–	–	c													
13	0	2	1	1	1	1	1	1	1	1	1	1	1	1	1	1	1	2	2	2	1	–	–	–	–	–	c													
13+14														1	1	1	1	2	2	1	–	–	–	–	–	–	c													
14	0	2	1	1	1	1	1	1	1	1	1	1	1	1	1	1	1	2	1	2	2	–	–	–	–	–	c													
13+14														1	1	1	1	2	1	2	–	–	–	–	–	–	c													
15	0	2	1	1	1	1	1	1	1	1	1	1	1	1	1	1	1	2	1	2	3	–	–	–	–	–	c													
13+14														1	1	1	2	1	1	2	1	–	–	–	–	–	c													
16	0	2	1	1	1	1	1	1	1	1	1	1	1	1	1	1	1	2	2	2	1	–	–	–	–	–	c													
13+14														1	1	1	2	1	2	1	–	–	–	–	–	–	c													
17	0	2	1	1	1	1	1	1	1	1	1	1	1	1	1	1	1	2	2	2	1	–	–	–	–	–	c													
13+14														1	1	1	2	1	2	1	–	–	–	–	–	–	c													
Nanochromis nudiceps CUMV 88260	0?	2	1	1	1	1	1	1	1	1	1	1	1	1	1	1	1	1	2	1	2	1	–	–	–	–	c													
14+13															1	1	1	1	2	1	–	–	–	–	–	–	c													
NHMUK 1963.10.22.9	–	2	1	1	1	1	1	1	1	1	1	1	1	1	1	1	1	1	1	1	1	2	1	2	–	–	–	–	c											
14+15															1	–	1	1	1	1	2	2	–	–	–	–	–	–	c											
Nanochromis parilus NHMUK 1977.1.11.33-34	–	2	1	1	1	1	1	1	1	1	1	1	1	1	1	1	1	1	1	1	1	1	2	2	1	–	–	–	c											
14+15															1	–	1	–	1	1	2	1	1	1?	–	–	–	–	c											
	–	2	1	1	1	1	1	1	1	1	1	1	1	1	1	1	1	1	1	1	1	2	2	1	1	–	–	–	c											
14+15															1	–	1	–	1	1	1	2	2	–	–	–	–	–	c											
Parananochromis gabonicus NHMUK 1967.10.12.57 Holotype	0	2	1	1	1	1	1	1	1	1	1	1	1	1	1	1	1	2	2	2	–	–	–	–	–	–	c													
13+14														1	1	1	1	2	2	–	–	–	–	–	–	–	c													
Parananochromis longirostris CUMV 80766	0	2	1	1	1	1	1	1	1	1	1	1	1	1	1	1	2	2	2	2	–	–	–	–	–	c														
14+12															2	–	2	1	2	1	–	–	–	–	–	c														
AMNH 230709 1	0	2	1	1	1	1	1	1	1	1	1	1	1	1	1	1	2	2	2	2	–	–	–	–	–	c														
14+12															1	1	2	1	3	–	–	–	–	–	–	c														
2	0	2	1	1	1	1	1	1	1	1	1	1	1	1	1	1	2	2	2	2	–	–	–	–	–	c														
14+12															1	1	1	2	3	–	–	–	–	–	–	c														
3	0	2	1	1	1	1	1	1	1	1	1	1	1	1	1	2	1	2	2	2	–	–	–	–	–	c														
14+12															1	1	2	1	2	1	–	–	–	–	–	c														
L1	0	2	1	1	1	1	1	1	1	1	1	1	1	1	1	1	2	1	3	1	–	–	–	–	–	c														
13+13														1	1	1	1	2	2	–	–	–	–	–	–	c														
L2	0	2	1	1	1	1	1	1	1	1	1	1	1	1	1	1	1	2	1	2	2	–	–	–	–	–	c													
14+13															1	1	1	2	2	1	–	–	–	–	–	–	c													
L3	0	2	1	1	1	1	1	1	1	1	1	1	1	1	1	1	2	1	2	3	–	–	–	–	–	c														
14+12															1	1	1	2	2	1	–	–	–	–	–	c														
R1	0	2	1	1	1	1	1	1	1	1	1	1	1	1	1	1	2	2	2	2	–	–	–	–	–	c														
14+12															1	1	1	2	3	–	–	–	–	–	–	c														
R2	0	2	1	1	1	1	1	1	1	1	1	1	1	1	1	1	2	2	2	1?	–	–	–	–	–	c														
13+13														1	1	1	2	2	1	–	–	–	–	–	–	c														
R3	0	2	1	1	1	1	1	1	1	1	1	1	1	1	1	1	2	1	3	2	1?	–	–	–	–	c														
14+12															1	1	1	2	2	1	–	–	–	–	–	c														
lowest	0	2	1	1	1	1	1	1	1	1	1	1	1	1	1	1	2	1	2	2	–	–	–	–	–	c														
13+13														2	–	1	1	2	2?	–	–	–	–	–	–	c														

Species, Museum, Catalog number / ID<br>Vertebral count	1	2	3	4	5	6	7	8	9	10	11	12	13	14	15	16	17	18	19	20	21	22	23	24	25	26	27	28	29	30	31	32	33	34	35	36	37	38	39	40
Pelmatochromis buettikoferi CUMV 97836	0	2	1	1	1	1	1	1	1	1	1	1	1	1	1	1	2	1	2	2	2	–	–	–	–	–	c													
15+12																1	1	2	2	2	1	–	–	–	–	–	c													
NHMUK 1910.11.28.11–12	0	2	1	1	1	1	1	1	1	1	1	1	1	1	1	1	2	2	2	2	1	–	–	–	–	–	c													
15+12																1	2	1	2	3	–	–	–	–	–	–	c													
	0	2	1	1	1	1	1	1	1	1	1	1	1	1	1	1	2	1	2	2	2	–	–	–	–	–	c													
15+12																1	1	2	2	3	–	–	–	–	–	–	c													
Pelmatochromis ocellifer NHMUK 1898.7.9.16 holotype	0	2	1	1	1	1	1	1	1	1	1	1	1	1	1	1	2	2	2	3	–	–	–	–	–	c														
14+12															1	1	1	2	2	2	–	–	–	–	–	c														
Pelvicachromis pulcher YPM 009680	0	2	1	1	1	1	1	1	1	1	1	1	1	1	1	1	1	2	2	2	1	–	–	–	–	–	c													
14+13															1	–	1	2	1	2	1	–	–	–	–	–	c													
Pelvicachromis taeniatus NHMUK 1901.1.28.21 holotype	0	2	1	1	1	1	1	1	1	1	1	1	1	1	1	1	1	1	2	2	2	–	–	–	–	–	c													
14+13															1	1	1	1	2	1	1	–	–	–	–	–	c													
Pterochromis congicus NHMUK 1897.9.30.12 holotype	0	2	1	1	1	1	1	1	1	1	1	1	1	1	1	2	2	2	2	–	–	–	–	–	–	c														
13+13														1	1	2	2	2	1	–	–	–	–	–	–	c														
NHMUK 1901.12.21.77	0	2	1	1	1	1	1	1	1	1	1	1	1	1	1	2	1	2	3	–	–	–	–	–	–	c														
13+13														1	1	1	2	2	3	–	–	–	–	–	–	c														
Teleogramma brichardi USNM 331312 1	1	1	1	1	1	1	1	1	1	1	1	1	1	1	1	1	1	1	1	1	1	1	1	1	2	1	2	–	–	–	–	–	c							
13+20														1	–	1	1	1	1	1	1	2	1	2	–	–	–	–	–	–	–	–	c							
2	1	1	1	1	1	1	1	1	1	1	1	1	1	1	1	1	1	1	1	1	1	1	1	1	1	2	1	1	–	–	–	–	c							
13+20														1	–	1	1	1	1	1	2	1	2	–	–	–	–	–	–	–	–	–	c							
3	–	2	1	1	1	1	1	1	1	1	1	1	1	1	1	1	1	1	1	1	1	1	1	1	1	2	1	1	–	–	–	–	c							
13+20														1	–	1	1	1	1	1	2	1	2	–	–	–	–	–	–	–	–	–	c							
4	–	2	1	1	1	1	1	1	1	1	1	1	1	1	1	1	1	1	1	1	1	1	1	1	1	2	1	2	–	–	–	–	c							
13+20														1	–	1	1	1	1	1	1	2	1	2	–	–	–	–	–	–	–	–	c							
Thysochromis ansorgii YPM 014283	0	2	1	1	1	1	1	1	1	1	1	1	1	1	1	1	2	1	2	2	2	–	–	–	–	–	c													
14+13															1	1	1	2	2	1	1	–	–	–	–	–	c													
NHMUK 1912.2.2.10-12 syntypes upper	0	2	1	1	1	1	1	1	1	1	1	1	1	1	1	1	2	1	2	2	1	–	–	–	–	–	c													
13+14														1	1	1	1	1	2	2	1	–	–	–	–	–	c													
mid	0	2	1	1	1	1	1	1	1	1	1	1	1	1	1	1	2	1	2	2	1	–	–	–	–	–	c													
13+14														1	1	1	1	2	2	1	–	–	–	–	–	–	c													
lower	0	2	1	1	1	1	1	1	1	1	1	1	1	1	1	1	2	2	2	1	–	–	–	–	–	c														
13+13														1	1	1	1	2	2	1	–	–	–	–	–	c														
Wallaceochromis humilis AMNH 97496	0	2	1	1	1	1	1	1	1	1	1	1	1	1	1	1	1	2	1	2	2	2	–	–	–	–	–	–	c											
15+14																1	1	2	1	2	1	–	–	–	–	–	–	–	c											
	0	2	1	1	1	1	1	1	1	1	1	1	1	1	1	1	1	2	1	2	1	2	2	–	–	–	–	–	c											
15+14																1	1	1	1	2	2	–	–	–	–	–	–	–	c											
	0	2	1	1	1	1	1	1	1	1	1	1	1	1	1	1	1	1	1	2	2	1	2	–	–	–	–	–	c											
15+14																1	1	1	1	2	1	1	–	–	–	–	–	–	c											
	0	2	1	1	1	1	1	1	1	1	1	1	1	1	1	1	1	1	2	1	2	2	1	–	–	–	–	–	c											
15+14																1	1	1	2	2	1	–	–	–	–	–	–	–	c											
NHMUK 1915.4.13.44 holotype	0	2	1	1	1	1	1	1	1	1	1	1	1	1	1	1	1	1	2	2	2	2	–	–	–	–	–	c												
15+13																–	2	1	1	2	2	–	–	–	–	–	–	c												
**Coelotilapiini**																																								
Coelotilapia joka NHMUK 1970.1.5.1	0	2	1	1	1	1	1	1	1	1	1	1	1	1	1	1	1	2	1	2	2	3	–	–	–	–	–	c												
15+13																1	–	1	1	2	2	2	–	–	–	–	–	c												
**Coptodonini**																																								
Coptodon discolor NHMUK 1903.4.24.33-35 middle	0	1	2	1	1	1	1	1	1	1	1	1	1	1	1	1	2	1	2	2	2	2	–	–	–	–	–	c												
syntypes 15+13																1	1	1	2	2	2	1	–	–	–	–	–	c												
bottom	0	2	1	1	1	1	1	1	1	1	1	1	1	1	1	1	1	2	2	2	3	1	–	–	–	–	–	c												
15+13																1	1	1	2	2	2	1	–	–	–	–	–	c												
Coptodon tholloni NHMUK 1896.5.5.33-35	0	2	1	1	1	1	1	1	1	1	1	1	1	1	1	1	2	1	2	3	1	–	–	–	–	–	c													
14+13															1	1	1	2	2	3	–	–	–	–	–	–	c													
Coptodon zillii NHMUK 1932.12.16.776–798 L1	0	2	1	1	1	1	1	1	1	1	1	1	1	1	1	1	1	1	2	1	2	3?	–	–	–	–	–	–	c											
16+13																	1	1	1	2	2	2	2	–	–	–	–	–	c											
L2	0	2	1	1	1	1	1	1	1	1	1	1	1	1	1	1	1	1	2	2	2	3	–	–	–	–	–	–	c											
16+13																	1	1	2	2	1	3	1	–	–	–	–	–	c											
L3	0	2	1	1	1	1	1	1	1	1	1	1	1	1	1	1	1	1	2	2	2	2	2	–	–	–	–	c												
16+12																	1	1	1	2	2	2	2	–	–	–	–	c												
L4	0	2	1	1	1	1	1	1	1	1	1	1	1	1	1	1	1	1	1	2	2	2	2	–	–	–	–	–	c											
16+13																	1	1	1	2	2	2	2	–	–	–	–	–	c											
L5	0	2	1	1	1	1	1	1	1	1	1	1	1	1	1	1	1	1	2	2	2	2	1	–	–	–	–	–	c											
16+13																	1	1	2	1	2	3	–?	–	–	–	–	–	c											
R1	0	2	1	1	1	1	1	1	1	1	1	1	1	1	1	1	1	1	1	2	2	3	1	–	–	–	–	–	c											
16+13																	2	–	1	2	2	3?	1?	–	–	–	–	–	c											
R2	0	2	1	1	1	1	1	1	1	1	1	1	1	1	1	1	1	2	1	2	3	1	–	–	–	–	–	–	c											
16+13																	1	1	2	2	2	3?	–	–	–	–	–	–	c											
R3	–	0-2	1	1	1	1	1	1	1	1	1	1	1	1	1	1	1	1	2	2	2	2	–?	–	–	–	–	–	c											
16+13																	1	1	1	2	2	2	1?	–	–	–	–	–	c											

Species, Museum, Catalog number / ID<br>Vertebral count	1	2	3	4	5	6	7	8	9	10	11	12	13	14	15	16	17	18	19	20	21	22	23	24	25	26	27	28	29	30	31	32	33	34	35	36	37	38	39	40
R4	0	2	1	1	1	1	1	1	1	1	1	1	1	1	2	–	1	1	2	2	2	2	1?	–	–	–	–	–	c											
16+13																	1	1	2	2	2	2	1	–	–	–	–	–	c											
R5	0	2	1	1	1	1	1	1	1	1	1	1	1	1	1	1	1	1	2	2	2	2	1	–	–	–	–	–	c											
16+13																	1	1	2	2	2	2	–	–	–	–	–	–	c											
USNM 229645 1	0	2	1	1	1	1	1	1	1	1	1	1	1	1	1	1	1	1	1	2	2	1	2	2?	–	–	–	–	–	–	c									
16+15																	1	–	2	1	1	2	2	1	–	–	–	–	–	–	c									
2	0	2	1	1	1	1	1	1	1	1	1	1	1	1	1	1	1	2	1	2	2	2	–	–	–	–	–	c												
15+13																1	1	1	2	1	2	1	–	–	–	–	–	c												
3	0	2	1	1	1	1	1	1	1	1	1	1	1	1	1	1	1	2	1	2	2	2	–	–	–	–	–	–	c											
15+14																1	1	1	2	1	2	1	–	–	–	–	–	–	c											
4	0	2	1	1	1	1	1	1	1	1	1	1	1	1	1	1	2	1	2	3	2	–	–	–	–	–	–	c												
15+13																1	1	2	1	2	2	–	–	–	–	–	–	c												
5	0	2	1	1	1	1	1	1	1	1	1	1	1	1	1	2	1	1	2	2	2	–	–	–	–	–	–	c												
14+14															1	–	1	1	2	2	1	–	–	–	–	–	–	c												
6	0	2	1	1	1	1	1	1	1	1	1	1	1	1	1	1	1	2	1	2	2	2	–	–	–	–	–	c												
15+13																1	1	1	1	2	2	1	–	–	–	–	–	c												
7	0	2	1	1	1	1	1	1	1	1	1	1	1	1	1	1	2	1	2	2	2	1	–	–	–	–	–	c												
14+14															1	–	1	2	1	2	1	–	–	–	–	–	–	c												
8	0	2	1	1	1	1	1	1	1	1	1	1	1	1	1	1	1	2	2	2	2	1	–	–	–	–	–	c												
14+14															1	–	1	2	1	2	2	1	–	–	–	–	–	c												
9	0	2	1	1	1	1	1	1	1	1	1	1	1	1	1	1	1	2	2	2	2	2	–	–	–	–	–	–	c											
14+15															1	–	1	1	2	2	2	–	–	–	–	–	–	–	c											
10	0	2	1	1	1	1	1	1	1	1	1	1	1	1	1	1	1	1	2	1	2	1	2	2	–	–	–	–	–	–	c									
16+15																	1	–	2	1	2	1	2	1	–	–	–	–	–	–	c									
11	0	2	1	1	1	1	1	1	1	1	1	1	1	1	1	2	1	2	2	2	2	–	–	–	–	–	–	c												
14+14															1	–	1	2	2	2	1	–	–	–	–	–	–	c												
12	0	2	1	1	1	1	1	1	1	1	1	1	1	1	1	1	1	1	2	1	2	2	2	–	–	–	–	–	c											
16+13																	1	1	1	2	2	2	3	–	–	–	–	–	c											
13	0	2	1	1	1	1	1	1	1	1	1	1	1	1	1	1	2	1	2	2	3	–?	–	–	–	–	–	c												
14+14															1	–	1	2	2	2	2	–	–	–	–	–	–	c												
14	0	2	1	1	1	1	1	1	1	1	1	1	1	1	1	1	1	1	2	2	2	2	–	–	–	–	–	–	c											
15+14																1	–	2	1	2	2	1	–	–	–	–	–	–	c											
**Etiini**																																								
Etia nguti UMMZ 248260	0-0	2	1	1	1	1	1	1	1	1	1	1	1	1	1	1	2	2	2	2	–	–	–	–	–	c														
14+12															1	–	2	2	3	1	–	–	–	–	–	c														
ZSM-PIS-029430_20140129_121137	0-0	2	1	1	1	1	1	1	1	1	1	1	1	1	1	1	2	2	2	2	–	–	–	–	–	c														
14+12															1	1	2	2	2	1	–	–	–	–	–	c														
ZSM-PIS-029430_20140129_121735	0-0	2	1	1	1	1	1	1	1	1	1	1	1	1	1	1	2	2	2	2	–	–	–	–	–	c														
14+12															2	–	1	2	3	1	–	–	–	–	–	c														
UAMZ F8702 81.9 mm SL	0-0	2	1	1	1	1	1	1	1	1	1	1	1	1	1	1	2	2	2	2	–	–	–	–	–	c														
14+12															1	1	1	2	2	2	–	–	–	–	–	c														
68.4 mm SL	0	0-2	1	1	1	1	1	1	1	1	1	1	1	1	1	1	2	2	2	2	–	–	–	–	–	c														
14+12															1	1	1	2	2	2	–	–	–	–	–	c														
63.8 mm SL	0-0?	2?	1	1	1	1	1	1	1	1	1	1	1	1	1	1	2	2	2	2	–	–	–	–	–	c														
14+12															1	1	2	2	2	1	–	–	–	–	–	c														
**Gobiocichlini**																																								
Gobiocichla ethelwynnae USNM 229454 4	–	–	2	1	1	1	1	1	1	1	1	1	1	1	1	1	1	1	1	1	1	1	1	1	2	1	2	1?	–	–	–	–	–	–	c					
(fish 1-3 not used) 17+18																		–	1	1	1	2	1	1	2	1	2	–	–	–	–	–	–	–	c					
5	–	–	2	1	1	1	1	1	1	1	1	1	1	1	1	1	1	1	1	1	1	1	1	1	2	1	2	1	–	–	–	–	–	–	c					
18+17																			1	1	1	1	2	1	1	2	1?	–	–	–	–	–	–	–	c					
6	–	–	2	1	1	1	1	1	1	1	1	1	1	1	1	1	1	1	1	1	1	1	1	1	2	1	2	–?	–	–	–	–	–	–	c					
17+18																		–	2	1	1	1	1	1	2	1	2	–	–	–	–	–	–	–	c					
7	–	–	2	1	1	1	1	1	1	1	1	1	1	1	1	1	1	1	1	1	1	1	1	1	2	1	2	1	–	–	–	–	–	–	–	c				
18+18																			1	1	1	2	1	1	2	1	2	–	–	–	–	–	–	–	–	c				
8	–	1	1	1	1	1	1	1	1	1	1	1	1	1	1	1	1	1	1	1	1	1	1	1	2	1	2	1?	–	–	–	–	–	–	c					
18+17																			1	2	–	1	2	1	2	1	1	–	–	–	–	–	–	–	c					
9	–	–	2	1	1	1	1	1	1	1	1	1	1	1	1	1	1	1	1	1	1	1	1	1	1	2	1	2	–	–	–	–	–	–	c					
18+17																			2	1	–	2	1	1	2	1	1	–	–	–	–	–	–	–	c					
10	–	–	2	1	1	1	1	1	1	1	1	1	1	1	1	1	1	1	1	1	1	1	1	1	1	2	1	2	–?	–	–	–	–	–	c					
18+17																			2	1	–	1	2	1	1	2	2	–	–	–	–	–	–	–	c					
11	–	1	1	1	1	1	1	1	1	1	1	1	1	1	1	1	1	1	1	1	1	1	1	1	2	2	1	–	–	–	–	–	–	c						
17+17																		1	1	1	1	1	1	2	2	2	–	–	–	–	–	–	–	c						
12	–	–	2	1	1	1	1	1	1	1	1	1	1	1	1	1	1	1	1	1	1	1	1	1	2	1	2	1	–	–	–	–	–	–	c					
18+17																			1	1	1	2	1	1	2	1	2	–	–	–	–	–	–	–	c					
13	–	–	2	1	1	1	1	1	1	1	1	1	1	1	1	1	1	1	1	1	1	1	1	1	2	1	2	1	–	–	–	–	–	–	c					
18+17																			1	1	1	1	2	1	2	1	2	–	–	–	–	–	–	–	c					
15	–	–	2	1	1	1	1	1	1	1	1	1	1	1	1	1	1	1	1	1	1	1	1	1	2	1	2	2	–	–	–	–	–	–	c					
18+17																			1	1	1	1	2	1	2	1	3	–	–	–	–	–	–	–	c					

Species, Museum, Catalog number / ID<br>Vertebral count	1	2	3	4	5	6	7	8	9	10	11	12	13	14	15	16	17	18	19	20	21	22	23	24	25	26	27	28	29	30	31	32	33	34	35	36	37	38	39	40
Gobiocichla wonderi USNM 357037 1	–	2	1	1	1	1	1	1	1	1	1	1	1	1	1	1	1	1	1	1	1	1	1	1	1	1	1	2	1	1	–	–	–	–	–	c				
18+18																			1	–	1	1	1	1	2	1	–	–	–	–	–	–	–	–	–	c				
2	–	2	1	1	1	1	1	1	1	1	1	1	1	1	1	1	1	1	1	1	1	1	1	1	1	1	1	2	1	1	–	–	–	–	–	c				
18+18																			1	–	1	1	1	1	2	1	1	–	–	–	–	–	–	–	–	c				
3	–	2	1	1	1	1	1	1	1	1	1	1	1	1	1	1	1	1	1	1	1	1	1	1	1	1	1	2	2	–	–	–	–	–	–	c				
18+18																			1	–	1	1	1	1	2	1	1	–	–	–	–	–	–	–	–	c				
4	–	2	1	1	1	1	1	1	1	1	1	1	1	1	1	1	1	1	1	1	1	1	1	1	1	1	2	1	2	–	–	–	–	–	–	c				
18+18																			1	–	1	1	1	1	2	1	2	–	–	–	–	–	–	–	–	c				
5	–	2	1	1	1	1	1	1	1	1	1	1	1	1	1	1	1	1	1	1	1	1	1	1	1	1	1	2	1	1	–	–	–	–	–	c				
18+18																			1	–	1	1	1	1	2	1	1	–	–	–	–	–	–	–	–	c				
6	–	2	1	1	1	1	1	1	1	1	1	1	1	1	1	1	1	1	1	1	1	1	1	1	1	1	1	2	1	1	–	–	–	–	–	c				
18+18																			1	–	1	1	1	1	1	2	1	1	–	–	–	–	–	–	–	c				
7	–	2	1	1	1	1	1	1	1	1	1	1	1	1	1	1	1	1	1	1	1	1	1	1	1	1	1	2	1	1	–	–	–	–	–	c				
18+18																			1	–	1	1	1	1	2	1	2	–	–	–	–	–	–	–	–	c				
8	–	2	1	1	1	1	1	1	1	1	1	1	1	1	1	1	1	1	1	1	1	1	1	1	1	1	1	1	2	1	–	–	–	–	–	c				
18+18																			1	–	1	1	1	2	1	2	1?	–	–	–	–	–	–	–	–	c				
9	–	2	1	1	1	1	1	1	1	1	1	1	1	1	1	1	1	1	1	1	1	1	1	1	1	1	1	2	1	1	–	–	–	–	–	–	c			
18+19																			1	–	1	1	1	2	1	1	2	–	–	–	–	–	–	–	–	–	c			
10	–	2	1	1	1	1	1	1	1	1	1	1	1	1	1	1	1	1	1	1	1	1	1	1	1	1	1	2	1	1	–	–	–	–	–	c				
18+18																			1	–	1	1	1	1	2	1	2	–	–	–	–	–	–	–	–	c				
11	–	1	2	1	1	1	1	1	1	1	1	1	1	1	1	1	1	1	1	1	1	1	1	1	1	1	1	1	2	1	–	–	–	–	–	c				
18+18																			1	–	1	1	1	1	2	2	1	–	–	–	–	–	–	–	–	c				
12	–	2	1	1	1	1	1	1	1	1	1	1	1	1	1	1	1	1	1	1	1	1	1	1	1	1	1	1	2	1	–	–	–	–	–	c				
18+18																			1	–	1	1	1	2	1	1	2	–	–	–	–	–	–	–	–	c				
13	–	2	1	1	1	1	1	1	1	1	1	1	1	1	1	1	1	1	1	1	1	1	1	1	1	1	1	2	1	1	–	–	–	–	–	c				
18+18																			1	–	1	1	1	2	1	2	1	–	–	–	–	–	–	–	–	c				
14	1	1	1	1	1	1	1	1	1	1	1	1	1	1	1	1	1	1	1	1	1	1	1	1	1	1	1	1	2	2	–	–	–	–	–	c				
18+18																			1	–	1	1	1	1	2	1	2	1	–	–	–	–	–	–	–	c				
Paragobiocichla irvinei NHMUK 1943.7.24.1-4 syntypes	–	2	1	1	1	1	1	1	1	1	1	1	1	1	1	1	1	1	1	1	1	2	1	2	2	1	–	–	–	–	–	c								
17+15																		1	–	1	1	1	2	2	–	–	–	–	–	–	–	c								
	–	2	1	1	1	1	1	1	1	1	1	1	1	1	1	1	1	1	1	1	1	2	1	2	2	–	–	–	–	–	–	c								
17+15																		1	–	1	1	2	2	1	–	–	–	–	–	–	–	c								
	–	2	1	1	1	1	1	1	1	1	1	1	1	1	1	1	1	1	1	1	2	1	2	1	3	–	–	–	–	–	–	c								
17+15																		1	–	1	2	1	2	1	–	–	–	–	–	–	–	c								
	–	2	1	1	1	1	1	1	1	1	1	1	1	1	1	1	1	1	1	1	1	2	2	1	2	1	–	–	–	–	–	c								
17+15																		1	–	1	1	1	2	2	–	–	–	–	–	–	–	c								
**Hemichromini**																																								
Anomalochromis thomasi CUMV 97804	0	2	1	1	1	1	1	1	1	1	1	1	1	1	2	2	2	3	–	–	–	–	–	–	c															
12+13													1	1	2	1	2	2	–	–	–	–	–	–	c															
NHMUK 1981.6.19.104	0	2	1	1	1	1	1	1	1	1	1	1	1	1	1	2	2	1	2	–	–	–	–	–	c															
13+12														1	1	2	2	2	1	–	–	–	–	–	c															
NHMUK 1981.6.19.118	0	2	1	1	1	1	1	1	1	1	1	1	1	1	2	1	2	2	2	–	–	–	–	–	c															
13+12														1	2	1	2	2	1	–	–	–	–	–	c															
NHMUK 1981.6.19.124	0	2	1	1	1	1	1	1	1	1	1	1	1	1	2	1	2	2	2	–	–	–	–	–	c															
12+13													1	–	2	1	2	2	1	–	–	–	–	–	c															
Hemichromis elongatus CUMV 92323 top	0	2	1	1	1	1	1	1	1	1	1	1	1	1	1	2	1	2	2	2	1	–	–	–	–	–	–	c												
15+13																1	3	1	2	3	–	–	–	–	–	–	–	c												
mid left	0	2	1	1	1	1	1	1	1	1	1	1	1	1	1	1	2	2	1	2	2	–	–	–	–	–	–	c												
15+13																1	2	2	2	2	1	–	–	–	–	–	–	c												
mid right	0	2	1	1	1	1	1	1	1	1	1	1	1	1	1	1	2	2	1	2	2	–	–	–	–	–	–	c												
15+13																1	2	2	1	2	2	–	–	–	–	–	–	c												
bottom	0	2	1	1	1	1	1	1	1	1	1	1	1	1	1	2	1	2	2	2	1	–	–	–	–	–	–	c												
15+13																1	2	1	2	2	2	–	–	–	–	–	–	c												
Hemichromis fasciatus CUMV 88248	0	2	1	1	1	1	1	1	1	1	1	1	1	1	1	2	1	2	2	2	1	–	–	–	–	–	–	c												
15+13																2	1	2	2	2	1	–	–	–	–	–	–	c												
Rubricatochromis bimaculatus CUMV 88263 L1	0	2	1	1	1	1	1	1	1	1	1	1	1	1	1	1	2	1	2	2	–	–	–	–	–	c														
13+13														1	1	1	1	2	2	1	–	–	–	–	–	c														
L2	0	2	1	1	1	1	1	1	1	1	1	1	1	1	1	1	2	2	1	2	–	–	–	–	–	c														
13+13														1	1	1	1	2	2	1	–	–	–	–	–	c														
L3	0	2	1	1	1	1	1	1	1	1	1	1	1	1	1	1	2	2	2	1	–	–	–	–	–	c														
13+13														1	1	1	2	2	1	–	–	–	–	–	–	c														
R1	0	2	1	1	1	1	1	1	1	1	1	1	1	1	1	1	2	2	2	2	–	–	–	–	–	c														
13+13														1	1	1	2	1	2	1	–	–	–	–	–	c														
R2	0	2	1	1	1	1	1	1	1	1	1	1	1	1	1	1	2	1	2	2	2?	–	–	–	–	–	c													
13+14														1	1	1	2	1	2	2	–	–	–	–	–	–	c													
R3	0	2	1	1	1	1	1	1	1	1	1	1	1	1	1	1	1	2	2	2	1?	–	–	–	–	–	c													
14+13															1	1	1	1	2	2	1	–	–	–	–	–	c													

Species, Museum, Catalog number / ID Vertebral count	1	2	3	4	5	6	7	8	9	10	11	12	13	14	15	16	17	18	19	20	21	22	23	24	25	26	27	28	29	30	31	32	33	34	35	36	37	38	39	40
Rubricatochromis letourneuxi NHMUK 1898.2.15.1 syntype	0	2	1	1	1	1	1	1	1	1	1	1	1	1	1	1	1	1	1	2	2	–	–	–	–	–	–	–	c											
12+17													2	–	–	1	1	2	1	2	1	–	–	–	–	–	–	–	c											
CUMV 94558 big L1	0	2	1	1	1	1	1	1	1	1	1	1	1	1	1	1	2	2	2	2	–	–	–	–	–	c														
13+13														1	1	1	1	2	2	1	–	–	–	–	–	c														
L2	0	2	1	1	1	1	1	1	1	1	1	1	1	1	1	2	2	2	1?	–	–	–	–	–	–	c														
13+13														1	1	1	2	1	2	–?	–	–	–	–	–	c														
L4	0	2	1	1	1	1	1	1	1	1	1	1	1	1	1	1	2	2	2	2?	–	–	–	–	–	c														
13+13														1	1	1	1	2	2	1	–	–	–	–	–	c														
**Heterochromini**																																								
Heterochromis multidens CUMV 88258	0	0-2	1	1	1	1	1	1	1	1	1	1	2	1	1	2	2	1	3	2	2	–	–	–	–	–	c													
13+14														2	–	1	2	1	2	2	–	–	–	–	–	–	c													
AMNH 239640	0	0-2	1	1	1	1	1	1	1	1	1	1	1	1	2	1	2	2	2	2	2	–	–	–	–	–	c													
13+14														1	1	1	1	2	2	2	–	–	–	–	–	–	c													
	0	0-2	1	1	1	1	1	1	1	1	1	1	1	1	2	1	2	2	2	2	2	–	–	–	–	–	c													
13+14														1	1	1	1	2	2	2	–	–	–	–	–	–	c													
CUMV 88257 L1	0	0-2	1	1	1	1	1	1	1	1	1	1	1	1	1	2	2	2	2	2	3	–	–	–	–	–	c													
13+14														1	1	1	1	2	2	3	–	–	–	–	–	–	c													
L2	0-0	2	1	1	1	1	1	1	1	1	1	1	1	1	2	1	2	2	2	3	2	–	–	–	–	–	c													
13+14														1	1	1	2	1	2	2	–	–	–	–	–	–	c													
L3	0	0-2	1	1	1	1	1	1	1	1	1	1	1	1	2	1	2	2	2	3	1	–	–	–	–	–	c													
13+14														1	1	1	1	2	2	1	1	–	–	–	–	–	c													
R1	0	0-2	1	1	1	1	1	1	1	1	1	1	1	1	2	1	2	2	2	2	3	–	–	–	–	–	c													
13+14														1	1	1	1	2	2	2	–	–	–	–	–	–	c													
R2	0	0-2	1	1	1	1	1	1	1	1	1	1	1	1	2	1	2	2	2	2	2	–	–	–	–	–	c													
13+14														1	1	1	1	2	2	2	–	–	–	–	–	–	c													
CUMV 89064	0	0-2	1	1	1	1	1	1	1	1	1	1	1	1	2	1	2	1	2	2	2	2	–	–	–	–	c													
13+14														1	–	2	1	1	3	2	–	–	–	–	–	–	c													
	0	0-2	1	1	1	1	1	1	1	1	1	1	1	1	1	2	2	1	2	3	3	–	–	–	–	–	c													
13+14														1	1	1	1	2	2	2	–	–	–	–	–	–	c													
	0	0-2	1	1	1	1	1	1	1	1	1	1	1	1	1	2	2	1	2	3	3	–	–	–	–	–	c													
13+14														1?	1?	1	1	2	2	2	–	–	–	–	–	–	c													
**Heterotilapiini**																																								
Heterotilapia buettikoferi AMNH 59425	0	2	1	1	1	1	1	1	1	1	1	1	1	1	1	1	2	1	3	2	2	3	1	–	–	–	–	c												
15+13																1	1	1	2	2	2	3	–	–	–	–	–	c												
	0	2	1	1	1	1	1	1	1	1	1	1	1	1	1	1	1	2	2	2	2	3	–	–	–	–	–	c												
15+13																1	1	1	2	2	2	2	–	–	–	–	–	c												
	0	2	1	1	1	1	1	1	1	1	1	1	1	1	1	1	1	2	2	2	2	3	–	–	–	–	–	c												
15+13																1	1	1	2	2	3	2	–	–	–	–	–	c												
	0	2	1	1	1	1	1	1	1	1	1	1	1	1	1	1	2	1	2	2	3	2	1	–	–	–	–	c												
15+13																1	1	1	2	2	2	2	–	–	–	–	–	c												
	0	2	1	1	1	1	1	1	1	1	1	1	1	1	1	1	2	1	2	2	3	2	1	–	–	–	–	c												
15+13																2	–	1	2	2	2	2	–	–	–	–	–	c												
**Oreochromini**																																								
Danakilia franchettii AMNH 223754 L1	0	2	1	1	1	1	1	1	1	1	1	1	1	1	1	1	2	2	2	1	–	–	–	–	–	–	c													
14+13															1	–	2	2	2	1	–	–	–	–	–	–	c													
L2	0	2	1	1	1	1	1	1	1	1	1	1	1	1	1	1	1	2	2	2	1	–	–	–	–	–	–	c												
14+14															2	–	1	1	2	2	–	–	–	–	–	–	–	c												
L3	0	2	1	1	1	1	1	1	1	1	1	1	1	1	1	1	1	2	2	2	1	–	–	–	–	–	c													
15+12																1	1	2	2	2	–	–	–	–	–	–	c													
L4	0	2	1	1	1	1	1	1	1	1	1	1	1	1	1	1	2	2	2	1	–	–	–	–	–	–	c													
14+13															1	–	2	2	2	–	–	–	–	–	–	–	c													
L5	0	2	1	1	1	1	1	1	1	1	1	1	1	1	1	1	2	2	2	1	–	–	–	–	–	–	c													
14+13															1	1	2	2	2	–	–	–	–	–	–	–	c													
L6	0	2	1	1	1	1	1	1	1	1	1	1	1	1	1	1	1	2	1	3	1	–	–	–	–	–	c													
14+13															1	1	1	2	2	2	–	–	–	–	–	–	c													
R1	0	2	1	1	1	1	1	1	1	1	1	1	1	1	1	1	2	2	2	1	–	–	–	–	–	–	c													
13+14														1	–	1	2	1	3	–	–	–	–	–	–	–	c													
R2	0	2	1	1	1	1	1	1	1	1	1	1	1	1	1	1	2	1	3	1	–	–	–	–	–	–	c													
14+13															2	–	1	2	2	2	–	–	–	–	–	–	c													
R3	0	2	1	1	1	1	1	1	1	1	1	1	1	1	1	1	2	2	2	1	–	–	–	–	–	–	c													
13+14														1	–	2	1	2	2	1	–	–	–	–	–	–	c													
R4	0	2	1	1	1	1	1	1	1	1	1	1	1	1	1	1	2	2	3	–	–	–	–	–	–	c														
13+13														1	–	1	2	2	2	–	–	–	–	–	–	c														
R5	0	2	1	1	1	1	1	1	1	1	1	1	1	1	1	1	2	1	2	2	–	–	–	–	–	–	c													
15+12																1	1	2	2	1	–	–	–	–	–	–	c													
R6	0	2	1	1	1	1	1	1	1	1	1	1	1	1	1	1	1	1	2	2	2	–	–	–	–	–	–	c												
15+13																1	1	1	2	3	–	–	–	–	–	–	–	c												

Species, Museum, Catalog number / ID<br>Vertebral count	1	2	3	4	5	6	7	8	9	10	11	12	13	14	15	16	17	18	19	20	21	22	23	24	25	26	27	28	29	30	31	32	33	34	35	36	37	38	39	40
NHMUK 1931.4.15.1–4 syntypes top	0	2	1	1	1	1	1	1	1	1	1	1	1	1	1	1	2	1	3	1	–	–	–	–	–	–	c													
14+13															1	1	2	2	2	–	–	–	–	–	–	–	c													
3rd from top	0	2	1	1	1	1	1	1	1	1	1	1	1	1	1	1	2	1	2	2	–	–	–	–	–	–	c													
14+13															1	1	2	1	2	1	–	–	–	–	–	–	c													
Oreochromis jipe NHMUK 1952.2.26.7-9 L2	0	2	1	1	1	1	1	1	1	1	1	1	1	1	1	1	1	1	1	1	2	1	2	2	2	–	–	–	–	–	–	c								
17+15																		1	2	1	2	2	2	2	–	–	–	–	–	–	–	c								
L3	0	2	1	1	1	1	1	1	1	1	1	1	1	1	1	1	1	1	1	1	2	1	2	2	2	1?	–	–	–	–	–	c								
17+15																		2	1	2	1	2	2	2	1	–	–	–	–	–	–	c								
L4	0	2	1	1	1	1	1	1	1	1	1	1	1	1	1	1	1	1	1	1	1	2	1	2	2	2	–	–	–	–	–	–	c							
18+15																			1	2	1	2	1	2	3	–	–	–	–	–	–	–	c							
L5	0	2	1	1	1	1	1	1	1	1	1	1	1	1	1	1	1	1	1	1	2	1	2	2	2	–	–	–	–	–	–	–	c							
17+16																		2	1	1	2	1	2	3	–	–	–	–	–	–	–	–	c							
Oreochromis niloticus NHMUK 1920.5.26.121–123	0	2	1	1	1	1	1	1	1	1	1	1	1	1	1	1	1	1	1	2	2	2	2	–	–	–	–	–	–	–	c									
16+15																	1	1	2	1	2	2	2	–	–	–	–	–	–	–	c									
	0	2	1	1	1	1	1	1	1	1	1	1	1	1	1	1	1	1	1	2	2	2	2	–	–	–	–	–	–	c										
16+14																	1	1	2	1	2	3	1	–	–	–	–	–	–	c										
	0	2	1	1	1	1	1	1	1	1	1	1	1	1	1	1	2	1	2	2	1?	–	–	–	–	–	c													
14+13															1	2	1	2	2	2	–	–	–	–	–	–	c													
Oreochromis shiranus AMNH 31872 a	0	2	1	1	1	1	1	1	1	1	1	1	1	1	1	1	1	1	2	1	2	2	2	–	–	–	–	–	–	c										
15+15																1	2	1	2	1	2	2	1	–	–	–	–	–	–	c										
b	0	2	1	1	1	1	1	1	1	1	1	1	1	1	1	1	1	1	2	2	2	2	1	–	–	–	–	–	–	c										
15+15																1	1	2	1	2	3	1	–	–	–	–	–	–	–	c										
c	0	2	1	1	1	1	1	1	1	1	1	1	1	1	1	1	1	1	2	1	2	2	2	–	–	–	–	–	–	c										
15+15																1	1	2	1	2	2	2	1	–	–	–	–	–	–	c										
d	0	2	1	1	1	1	1	1	1	1	1	1	1	1	1	1	1	1	1	2	1	2	3	–	–	–	–	–	–	c										
16+14																	2	2	1	2	2	2	1	–	–	–	–	–	–	c										
e	0	2	1	1	1	1	1	1	1	1	1	1	1	1	1	1	1	1	2	1	2	3	1	–	–	–	–	–	–	c										
15+15																1	2	1	2	2	2	2	–	–	–	–	–	–	–	c										
f	0	2	1	1	1	1	1	1	1	1	1	1	1	1	1	1	1	1	2	1	2	2	2	–	–	–	–	–	–	c										
15+15																1	1	2	1	2	2	2	–	–	–	–	–	–	–	c										
Sarotherodon galilaeus NHMUK 1972.1.27.4–5	0	2	1	1	1	1	1	1	1	1	1	1	1	1	1	1	1	1	2	1	2	3	2	–	–	–	–	–	c											
16+13																	2	–	1	1	2	3	1	–	–	–	–	–	c											
	0	2	1	1	1	1	1	1	1	1	1	1	1	1	1	1	1	1	2	2	3	2	1	–	–	–	–	–	c											
16+13																	2	–	2	2	2	2	–	–	–	–	–	–	c											
Sarotherodon melanotheron UF 91814	0	2	1	1	1	1	1	1	1	1	1	1	1	1	1	1	1	2	2	3	1	–	–	–	–	–	c													
14+13															1	–	1	2	1	3	1	–	–	–	–	–	c													
NHMUK 1972.2.18.17-22 1	0	2	1	1	1	1	1	1	1	1	1	1	1	1	1	1	2	1	2	3	–	–	–	–	–	–	c													
14+13															1	–	2	1	2	3	–	–	–	–	–	–	c													
2	0	2	1	1	1	1	1	1	1	1	1	1	1	1	1	1	1	2	2	2	2	–	–	–	–	–	c													
15+12																1	1	2	1	3	2	–	–	–	–	–	c													
3	0	2	1	1	1	1	1	1	1	1	1	1	1	1	1	1	2	2	2	2	1	–	–	–	–	–	c													
14+13															1	–	1	2	2	3	–	–	–	–	–	–	c													
4	0	2	1	1	1	1	1	1	1	1	1	1	1	1	1	1	2	1	2	2	2	–	–	–	–	–	c													
14+13															1	–	2	1	2	3	–	–	–	–	–	–	c													
vertical	0	2	1	1	1	1	1	1	1	1	1	1	1	1	1	1	1	2	2	2	2	–	–	–	–	–	c													
15+12																1	2	1	2	2	2	–	–	–	–	–	c													
**Pelmatolapiini**																																								
Pelmatolapia mariae USNM 304008 1																																								
Not used: Can't resolve posterior Pt's																																								
2	0	2	1	1	1	1	1	1	1	1	1	1	1	1	1	1	1	2	2	2	2	3	–	–	–	–	–	c												
15+13																2	–	1	2	2	2	2	–	–	–	–	–	c												
3	0	2	1	1	1	1	1	1	1	1	1	1	1	1	1	1	1	2	2	2	2	3	–	–	–	–	–	c												
15+13																2	–	1	2	2	2	2	–	–	–	–	–	c												
4	0	2	1	1	1	1	1	1	1	1	1	1	1	1	1	1	1	2	2	2	2	3	–	–	–	–	–	c												
15+13																1	–	2	1	2	2	3	1	–	–	–	–	c												
5	0	2	1	1	1	1	1	1	1	1	1	1	1	1	1	1	1	2	2	2	2	3	–	–	–	–	–	c												
15+13																1	1	1	2	2	2	3	–	–	–	–	–	c												
6	0	2	1	1	1	1	1	1	1	1	1	1	1	1	1	1	1	2	2	2	2	3	–	–	–	–	–	c												
15+13																2	–	1	2	1	2	3	–	–	–	–	–	c												
7	0	2	1	1	1	1	1	1	1	1	1	1	1	1	1	1	1	2	2	2	3	2	–	–	–	–	–	c												
15+13																2	–	1	2	2	2	2	–	–	–	–	–	c												
**Pseudocrenilabrini**																																								
Astatotilapia bloyeti NHMUK 1898.2.15.2 syntype	0	2	1	1	1	1	1	1	1	1	1	1	1	1	1	1	1	1	1	2	2	–	–	–	–	–	–	–	c											
12+17													1	–	1	1	1	2	1	2	2	–	–	–	–	–	–	–	c											
NHMUK 1969.2.11.372–387 L1	0	2	1	1	1	1	1	1	1	1	1	1	1	1	1	1	1	1	2	1	2	–	–	–	–	–	–	–	c											
13+16														2	–	1	1	1	1	2	2	–	–	–	–	–	–	–	c											
L2	0	2	1	1	1	1	1	1	1	1	1	1	1	1	1	1	1	1	2	2	1	–	–	–	–	–	–	–	c											
12+17													2	–	–	1	1	2	1	3	–	–	–	–	–	–	–	–	c											

Species, Museum, Catalog number / ID<br>Vertebral count	1	2	3	4	5	6	7	8	9	10	11	12	13	14	15	16	17	18	19	20	21	22	23	24	25	26	27	28	29	30	31	32	33	34	35	36	37	38	39	40
L3	0	2	1	1	1	1	1	1	1	1	1	1	1	1	1	1	1	1	2	1	2	1	–	–	–	–	–	–	–	c										
13+17														2	–	–	1	2	1	2	2?	–	–	–	–	–	–	–	–	c										
R1 too blurred to use																																								
R2	0	2	1	1	1	1	1	1	1	1	1	1	1	1	1	1	1	1	2	2	–	–	–	–	–	–	–	c												
12+16													1	–	1	1	1	2	1	2	–	–	–	–	–	–	–	c												
R3	0	2	1	1	1	1	1	1	1	1	1	1	1	1	1	1	1	1	1	2	2?	–	–	–	–	–	–	–	c											
13+16														1	1	1	1	1	2	2	2	–	–	–	–	–	–	–	c											
R4	0	2	1	1	1	1	1	1	1	1	1	1	1	1	1	1	1	1	2	2	1	–	–	–	–	–	–	–	c											
13+16														1	1	1	1	1	2	2	1	–	–	–	–	–	–	–	c											
bottom center	0	2	1	1	1	1	1	1	1	1	1	1	1	1	1	1	1	1	2	1	2	–	–	–	–	–	–	–	c											
13+16														1	1	1	1	1	2	2	1	–	–	–	–	–	–	–	c											
Astatotilapia burtoni NHMUK 1950.4.1.2176-2200 L1	0	2	1	1	1	1	1	1	1	1	1	1	1	1	1	1	1	1	2	1	2	1	–	–	–	–	–	c												
13+15														1	1	1	1	1	2	2	2	–	–	–	–	–	–	c												
L2	0	2	1	1	1	1	1	1	1	1	1	1	1	1	1	1	1	2	3	–	–	–	–	–	–	–	–	c												
13+15														1	1	1	2	1	2	2	–	–	–	–	–	–	–	c												
L3	0	2	1	1	1	1	1	1	1	1	1	1	1	1	1	1	1	1	2	2	2	–	–	–	–	–	–	c												
13+15?														1	1	1	1	1	2	2	1	–	–	–	–	–	–	c												
R1	0	2	1	1	1	1	1	1	1	1	1	1	1	1	1	1	1	1	2	2	2	–	–	–	–	–	–	–	c											
13+16														1	1	1	1	1	2	2	1	–	–	–	–	–	–	–	c											
R2	0	2	1	1	1	1	1	1	1	1	1	1	1	1	1	1	1	2	2	2	–	–	–	–	–	–	–	c												
13+15														1	1	1	1	2	2	2	–	–	–	–	–	–	–	c												
R3	0	2	1	1	1	1	1	1	1	1	1	1	1	1	1	1	1	2	2	2	–	–	–	–	–	–	–	c												
13+15														1	1	1	2	1	2	2	–	–	–	–	–	–	–	c												
Astatotilapia calliptera USNM 330613	0	2	1	1	1	1	1	1	1	1	1	1	1	1	1	1	1	1	1	2	2	–	–	–	–	–	–	–	c											
13+16														1	1	–	1	1	2	1	2	–	–	–	–	–	–	–	c											
	0	2	1	1	1	1	1	1	1	1	1	1	1	1	1	1	1	1	1	2	2	1	–	–	–	–	–	–	c											
13+16														1	–	1	1	1	2	1	3	–	–	–	–	–	–	–	c											
	0	2	1	1	1	1	1	1	1	1	1	1	1	1	1	1	1	1	1	2	2	1	–	–	–	–	–	–	–	c										
13+17														1	–	1	1	1	1	2	2	–	–	–	–	–	–	–	–	c										
	0	2	1	1	1	1	1	1	1	1	1	1	1	1	1	1	1	1	1	2	1	2	–	–	–	–	–	–	–	c										
13+17														2	–	–	1	1	1	2	2	1	–	–	–	–	–	–	–	c										
	0	2	1	1	1	1	1	1	1	1	1	1	1	1	1	1	1	1	1	2	2	1	–	–	–	–	–	–	–	c										
13+17														2	–	–	1	1	2	1	2	–	–	–	–	–	–	–	–	c										
right singleton	0	2	1	1	1	1	1	1	1	1	1	1	1	1	1	1	1	1	1	2	2	1	–	–	–	–	–	–	–	c										
14+16															1	1	1	1	1	1	2	1	–	–	–	–	–	–	–	c										
NHMUK 1921.9.6.84-93 top	0	2	1	1	1	1	1	1	1	1	1	1	1	1	1	1	1	1	1	2	1	2	–	–	–	–	–	–	–	c										
14+16															2	–	–	1	1	2	2	1	–	–	–	–	–	–	–	c										
NHMUK 1893.11.15.1-4 Lectotype	0	2	1	1	1	1	1	1	1	1	1	1	1	1	1	1	1	1	2	1	2	1	–	–	–	–	–	–	–	c										
13+17														1	–	1	1	1	2	1	2	–	–	–	–	–	–	–	–	c										
NHMUK 1893.1.17.4 a paralectotype	0	2	1	1	1	1	1	1	1	1	1	1	1	1	1	1	1	1	2	1	3	–	–	–	–	–	–	c												
13+15														1	1	1	1	1	1	2	1	–	–	–	–	–	–	c												
Astatotilapia swynnertoni NHMUK 1907.7.2.21–23 syntypes	0	2	1	1	1	1	1	1	1	1	1	1	1	1	1	1	1	1	2	1	2	–	–	–	–	–	–	c												
13+15														2	–	1	1	1	2	2	1	–	–	–	–	–	–	c												
	0	1	1	1	1	1	1	1	1	1	1	1	1	1	1	1	1	1	2	1	3	–	–	–	–	–	–	–	c											
13+16														1	1	1	1	1	1	2	2	–	–	–	–	–	–	–	c											
	0	2	1	1	1	1	1	1	1	1	1	1	1	1	1	1	1	1	1	1	2	2	–	–	–	–	–	–	c											
13+16														1	1	–	1	1	2	2	2	1	–	–	–	–	–	–	c											
Chetia flaviventris UMMZ 251518	0	2	1	1	1	1	1	1	1	1	1	1	1	1	1	1	1	1	1	2	2	1	1	–	–	–	–	–	–	–	c									
14+17															1	–	1	1	2	2	1	2	–	–	–	–	–	–	–	–	c									
Chetia gracilis NHMUK 1984.2.6.147 Holotype	0	2	1	1	1	1	1	1	1	1	1	1	1	1	1	1	1	1	1	1	2	2	2	1?	–	–	–	–	–	–	–	c								
15+17																1	1	1	1	1	2	2	2	–	–	–	–	–	–	–	–	c								
Chetia ? sp. AMNH 8334 a	0	2	1	1	1	1	1	1	1	1	1	1	1	1	1	2	1	2	3	–	–	–	–	–	–	c														
12+14													1	–	1	2	1	2	2	–	–	–	–	–	–	c														
b	0	2	1	1	1	1	1	1	1	1	1	1	1	1	1	2	1	2	2	1	–	–	–	–	–	c														
12+14													1	–	1	1	2	2	2	–	–	–	–	–	–	c														
d	0	2	1	1	1	1	1	1	1	1	1	1	1	1	1	1	2	2	2	1	–	–	–	–	–	c														
12+14													1	1	1	2	2	2	?	–	–	–	–	–	–	c														
e	0	2	1	1	1	1	1	1	1	1	1	1	1	1	1	1	2	1	3	2	–	–	–	–	–	c														
13+13														1	1	1	2	2	2	1	–	–	–	–	–	c														
f	0	2	1	1	1	1	1	1	1	1	1	1	1	1	1	2	2	2	2	–	–	–	–	–	–	c														
12+14													1	–	1	2	1	2	2	–	–	–	–	–	–	c														
g	0	2	1	1	1	1	1	1	1	1	1	1	1	1	1	1	1	2	2	2	1?	–	–	–	–	c														
12+14													1	1	1	1	2	2	?	?	–	–	–	–	–	c														
h	0	2	1	1	1	1	1	1	1	1	1	1	1	1	1	1	2	2	2	1	–	–	–	–	–	c														
13+13														1	1	1	2	1	3	–?	–	–	–	–	–	c														
i	0	2	1	1	1	1	1	1	1	1	1	1	1	1	1	1	2	2	2	2	–	–	–	–	–	c														
13+13														1	1	1	2	2	2	1	–	–	–	–	–	c														

Species, Museum, Catalog number / ID<br>Vertebral count	1	2	3	4	5	6	7	8	9	10	11	12	13	14	15	16	17	18	19	20	21	22	23	24	25	26	27	28	29	30	31	32	33	34	35	36	37	38	39	40
Ctenochromis pectoralis NHMUK 1899.2.27.1 paralecto	0	2	1	1	1	1	1	1	1	1	1	1	1	1	1	1	1	1	1	2	2	1	–	–	–	–	–	–	c											
12+17													1	–	1	–	1	2	1	2	2	–	–	–	–	–	–	–	c											
1 ex NHMUK 2021.7.15.1-3	0	2	1	1	1	1	1	1	1	1	1	1	1	1	1	1	1	1	2	1	2	1	–	–	–	–	–	–	c											
12+17													1	–	1	1	1	1	2	2	1	–	–	–	–	–	–	–	c											
Ctenochromis scatebra NHMUK 2021.7.15.4 holotype	0	2	1	1	1	1	1	1	1	1	1	1	1	1	1	1	1	1	2	1	2	1	–	–	–	–	–	–	–	c										
13+17														2	–	–	2	1	2	2	–	–	–	–	–	–	–	–	–	c										
Haplochromis demeusii NHMUK 1899.6.28.25 holotype	0	2	1	1	1	1	1	1	1	1	1	1	1	1	1	1	2	2	2	2	–	–	–	–	–	–	c													
13+14														1	1	1	1	2	2	–	–	–	–	–	–	–	c													
Haplochromis fasciatus NHMUK 1898.12.12.1–6 L1	0	2	1	1	1	1	1	1	1	1	1	1	1	1	1	1	1	2	2	2	2	–	–	–	–	–	–	c												
syntypes 14+14															1	1	1	1	2	2	–	–	–	–	–	–	–	c												
L2	0	2	1	1	1	1	1	1	1	1	1	1	1	1	1	1	2	1	3	2	1	–	–	–	–	–	–	c												
14+14															1	1	1	2	2	1	–	–	–	–	–	–	–	c												
L3	0	2	1	1	1	1	1	1	1	1	1	1	1	1	1	1	2	1	2	3	–	–	–	–	–	–	–	c												
14+14?															1	1	1	1	2	2	–	–	–	–	–	–	–	c												
R1	0	2	1	1	1	1	1	1	1	1	1	1	1	1	1	1	1	2	2	2	2	–	–	–	–	–	–	c												
14+14															1	1	–	1	2	3	–	–	–	–	–	–	–	c												
R2	0	2	1	1	1	1	1	1	1	1	1	1	1	1	1	1	1	2	2	2	1	–	–	–	–	–	–	c												
14+14															1	1	–	2	2	2	–	–	–	–	–	–	–	c												
R3	0	2	1	1	1	1	1	1	1	1	1	1	1	1	1	1	2	1	2	2	1?	–	–	–	–	–	–	c												
14+14															1	1	1	1	2	2?	–	–	–	–	–	–	–	c												
Haplochromis humilis NHMUK 1937.4.22.110-111	0	2	1	1	1	1	1	1	1	1	1	1	1	1	1	1	1	1	1	2	2	2	–	–	–	–	–	–	–	c										
14+16															1	–	1	1	2	1	3	1	–	–	–	–	–	–	–	c										
	0	2	1	1	1	1	1	1	1	1	1	1	1	1	1	1	1	1	2	1	2	2	–	–	–	–	–	–	–	c										
14+16															1	1	1	1	1	2	2	1	–	–	–	–	–	–	–	c										
NHMUK 1937.4.22.109	0	2	1	1	1	1	1	1	1	1	1	1	1	1	1	1	1	1	2	1	2	2	–	–	–	–	–	–	–	c										
14+16															2	–	1	1	2	2	1	–	–	–	–	–	–	–	–	c										
Haplochromis moeruensis NHMUK 1920.5.26.148–153	0	2	1	1	1	1	1	1	1	1	1	1	1	1	1	1	1	1	2	1	2	1	–	–	–	–	–	–	–	c										
L1 13+17														1	–	1	–	2	1	2	2	–	–	–	–	–	–	–	–	c										
L2	0	2	1	1	1	1	1	1	1	1	1	1	1	1	1	1	1	1	1	2	2	2	–	–	–	–	–	–	–	c										
13+17														1	–	1	1	1	1	2	2?	–	–	–	–	–	–	–	–	c										
R1	0	2	1	1	1	1	1	1	1	1	1	1	1	1	1	1	1	1	1	1	2	2	–	–	–	–	–	–	–	c										
14+16															1	1	–	1	1	2	2	1	–	–	–	–	–	–	–	c										
R2	0	2	1	1	1	1	1	1	1	1	1	1	1	1	1	1	1	1	1	2	1	3	–	–	–	–	–	–	–	c										
14+16															1	1	–	1	1	2	2	–	–	–	–	–	–	–	–	c										
Haplochromis oligacanthus NHMUK 1920.7.12.48 holotype	0	2	1	1	1	1	1	1	–	1	1	1	2	–	1	1	2	1	3	–	–	–	–	–	–	c														
12+14													1	–	1	–	2	1	2	–	–	–	–	–	–	c														
Lufubuchromis relictus ZSM 47494 holotype	0	2	1	1	1	1	1	1	1	1	1	1	1	1	1	1	1	2	1	2	2	–	–	–	–	–	–	–	c											
13+16														2	–	–	1	2	1	3	–	–	–	–	–	–	–	–	c											
Orthochromis machadoi NHMUK 1972.9.27.90–91	0	2	1	1	1	1	1	1	1	1	1	1	1	1	1	1	1	1	1	1	2	2	2	–	–	–	–	–	–	–	c									
13+18														1	1	–	1	1	1	2	2	–	–	–	–	–	–	–	–	–	c									
	0	2	1	1	1	1	1	1	1	1	1	1	1	1	1	1	1	1	1	1	2	2	1	–	–	–	–	–	–	c										
13+17														1	1	–	1	1	1	2	2	–	–	–	–	–	–	–	–	c										
Orthochromis malagaraziensis CUMV 95549	0	2	1	1	1	1	1	1	1	1	1	1	1	1	1	1	1	1	1	1	1	1	2	2	–	–	–	–	–	–	c									
13+18														1	–	1	1	1	1	1	2	1	1	1?	–	–	–	–	–	–	c									
NHMUK 1937.12.16.1	0	2	1	1	1	1	1	1	1	1	1	1	1	1	1	1	1	1	1	1	2	1	1	2	–	–	–	–	–	–	c									
13+18														2	–	–	1	1	2	1	1	2	–	–	–	–	–	–	–	–	c									
Orthochromis polyacanthus NHMUK 1920.5.26.137	0	2	1	1	1	1	1	1	1	1	1	1	1	1	1	1	1	1	1	1	1	2	1	2	1	–	–	–	–	–	c									
14+17															1	–	1	–	1	1	2	1	1	–	–	–	–	–	–	–	c									
	0	2	1	1	1	1	1	1	1	1	1	1	1	1	1	1	1	1	1	1	1	2	1	2	–	–	–	–	–	–	–	c								
14+18															1	–	1	1	–	1	2	2	–	–	–	–	–	–	–	–	–	c								
Orthochromis stormsi NHMUK 1977.1.11.5–24 L1	0	2	1	1	1	1	1	1	1	1	1	1	1	1	1	1	1	1	1	2	2	2	–	–	–	–	–	–	c											
13+16														1	–	1	1	1	1	2	1	–	–	–	–	–	–	–	c											
L2	0	2	1	1	1	1	1	1	1	1	1	1	1	1	1	1	1	1	1	2	2	2	–	–	–	–	–	–	c											
13+16														1	–	1	–	1	2	1	2	–	–	–	–	–	–	–	c											
L3	0	2	1	1	1	1	1	1	1	1	1	1	1	1	1	1	1	1	1	2	1	2	1	–	–	–	–	–	c											
13+16														1	–	1	–	2	1	1	2	–	–	–	–	–	–	–	c											
L4	0	2	1	1	1	1	1	1	1	1	1	1	1	1	1	1	1	1	1	2	2	1	1	–	–	–	–	–	c											
13+16														1	–	1	1	1	1	2	1	–	–	–	–	–	–	–	c											
R1	0	2	1	1	1	1	1	1	1	1	1	1	1	1	1	1	1	1	1	2	1	2	1	–	–	–	–	–	c											
13+16														1	–	1	–	1	1	2	2	–	–	–	–	–	–	–	c											
R2	0	**3**	1	1	1	1	1	1	1	1	1	1	1	1	1	1	1	1	1	2	1	2	2	–	–	–	–	c												
13+15														1	1	–	1	–	2	2	2	–	–	–	–	–	–	c												
R3	0	2	1	1	1	1	1	1	1	1	1	1	1	1	1	1	1	1	1	2	1	2	1	–	–	–	–	–	c											
13+16														1	–	1	–	1	2	1	2	–	–	–	–	–	–	–	c											
R4	0	2	1	1	1	1	1	1	1	1	1	1	1	1	1	1	1	1	1	2	1	2	2	–	–	–	–	–	–	c										
13+17														1	–	–	1	1	1	2	2	1	–	–	–	–	–	–	–	c										
NHMUK 1975.6.20.662-663	0	2	1	1	1	1	1	1	1	1	1	1	1	1	1	1	1	1	1	2	2	2	–	–	–	–	–	–	c											
13+16														1	–	1	1	1	1	2	1	–	–	–	–	–	–	–	c											

Species, Museum, Catalog number / ID<br>Vertebral count	1	2	3	4	5	6	7	8	9	10	11	12	13	14	15	16	17	18	19	20	21	22	23	24	25	26	27	28	29	30	31	32	33	34	35	36	37	38	39	40
	0	2	1	1	1	1	1	1	1	1	1	1	1	1	1	1	1	1	1	2	1	2	2	–	–	–	–	–	–	c										
14+16															1	1	–	1	2	2	1	–	–	–	–	–	–	–	–	c										
NHMUK 1992.10.9.1-2	0	2	1	1	1	1	1	1	1	1	1	1	1	1	1	1	1	1	1	1	2	1	2	1	–	–	–	–	–	–	c									
14+17															1	–	1	1	1	1	2	1	–	–	–	–	–	–	–	–	c									
Palaeoplex palimpsest ZSM 47492 holotype	0	2	1	1	1	1	1	1	1	1	1	1	1	1	1	1	1	1	1	2	1	3	–	–	–	–	–	–	–	c										
14+16															1	1	1	1	1	2	3	–	–	–	–	–	–	–	–	c										
Pharyngochromis acuticeps NHMUK 1908.12.11.5	0	2	1	1	1	1	1	1	1	1	1	1	1	1	1	1	1	1	2	1	3	1	–	–	–	–	–	–	–	c										
14+16															1	–	1	1	1	2	2	1	–	–	–	–	–	–	–	c										
NHMUK 1937.4.22.99-108 upper	0	2	1	1	1	1	1	1	1	1	1	1	1	1	1	1	1	1	2	2	2	–	–	–	–	–	–	–	–	c										
14+16															1	1	–	2	2	2	1	–	–	–	–	–	–	–	–	c										
2nd	0	2	1	1	1	1	1	1	1	1	1	1	1	1	1	1	1	2	1	2	1	–	–	–	–	–	–	–	–	c										
13+17														1	–	1	1	2	2	2	–	–	–	–	–	–	–	–	–	c										
3rd	0	2	1	1	1	1	1	1	1	1	1	1	1	1	1	1	1	2	1	3	1	–	–	–	–	–	–	–	c											
13+16														1	1	1	–	2	2	2	–	–	–	–	–	–	–	–	c											
lower left	0	2	1	1	1	1	1	1	1	1	1	1	1	1	1	1	1	1	2	1	2	1?	–	–	–	–	–	–	–	c										
14+16															1	1	1	1	2	1	2	–	–	–	–	–	–	–	–	c										
lower right	0	2	1	1	1	1	1	1	1	1	1	1	1	1	1	1	1	2	1	2	2?	–	–	–	–	–	–	–	–	c										
14+16															2	–	1	1	2	2	–	–	–	–	–	–	–	–	–	c										
Pseudocrenilabrus multicolor NHMUK 1964.7.1.89–108 L1	0	1	2	1	1	1	1	1	1	1	1	1	1	1	1	1	1	1	2	2	2?	–	–	–	–	–	c													
13+14														1	1	1	1	2	2	2?	–	–	–	–	–	–	c													
L2	0	2	1	1	1	1	1	1	1	1	1	1	1	1	1	1	1	1	2	2	2?	–	–	–	–	–	c													
13+14														2	1	–	1	2	1	2?	1?	–	–	–	–	–	c													
R1	0	2	1	1	1	1	1	1	1	1	1	1	1	1	1	1	1	1	2	2	1	–	–	–	–	–	c													
13+14														1	1	1	2	1	2	1	–	–	–	–	–	–	c													
R2	0	2	1	1	1	1	1	1	1	1	1	1	1	1	1	1	1	1	2	2	1?	–	–	–	–	c														
12+14													1	–	1	1	2	1	2	1	–	–	–	–	–	c														
Pseudocrenilabrus philander CUMV 91164	0	2	1	1	1	1	1	1	1	1	1	1	1	1	1	1	1	1	2	2	1	–	–	–	–	–	–	c												
12+16													1	–	1	1	1	2	2	1	–	–	–	–	–	–	–	c												
Sargochromis carlottae NHMUK 1910.5.31.65-66	0	2	1	1	1	1	1	1	1	1	1	1	1	1	1	1	1	1	1	2	1	2	2	–	–	–	–	–	–	–	c									
15+16																2	–	1	2	1	2	3	–	–	–	–	–	–	–	–	c									
	0	2	1	1	1	1	1	1	1	1	1	1	1	1	1	1	1	1	1	1	2	2	2	1	–	–	–	–	–	–	c									
16+15																	2	1	1	1	2	2	2	–	–	–	–	–	–	–	c									
Sargochromis codringtonii NHMUK 1908.11.6.32 lectotype	0	2	1	1	1	1	1	1	1	1	1	1	1	1	1	1	1	1	1	1	2	1	2	3	–	–	–	–	–	–	–	–	c							
16+17																	1	1	1	1	1	2	2	2	–	–	–	–	–	–	–	–	c							
paralectotype	0	2	1	1	1	1	1	1	1	1	1	1	1	1	1	1	1	1	1	2	1	2	2	2	1	–	–	–	–	–	–	c								
16+16																	1	1	1	1	1	2	2	2	–	–	–	–	–	–	–	c								
USNM 309604	0	2	1	1	1	1	1	1	1	1	1	1	1	1	1	1	1	1	1	2	2	2	1	–	–	–	–	–	–	–	c									
15+16																1	1	1	1	2	2	2	–	–	–	–	–	–	–	–	c									
Sargochromis giardi NHMUK 1907.6.29.181-182	0	2	1	1	1	1	1	1	1	1	1	1	1	1	1	1	1	1	1	2	2	2	2	–	–	–	–	–	–	–	c									
15+16																1	1	1	1	2	2	2	1	–	–	–	–	–	–	–	c									
	0	2	1	1	1	1	1	1	1	1	1	1	1	1	1	1	1	1	1	1	2	2	1	2	–	–	–	–	–	–	–	c								
15+17																3	–	–	1	1	2	2	1	–	–	–	–	–	–	–	–	c								
Sargochromis greenwoodi NHMUK 1910.5.31.53 holotype	0	2	1	1	1	1	1	1	1	1	1	1	1	1	1	1	1	1	1	2	1	2	2	1	–	–	–	–	–	–	–	c								
16+16																	1	1	1	2	1	2	2	–	–	–	–	–	–	–	–	c								
NHMUK 1910.5.31.55 paratype	0	2	1	1	1	1	1	1	1	1	1	1	1	1	1	1	1	1	1	2	1	2	2	2	–	–	–	–	–	–	–	c								
16+16																	1	–	1	1	2	2	2	–	–	–	–	–	–	–	–	c								
Serranochromis angusticeps NHMUK 1905.11.10.27-30	0	2	1	1	1	1	1	1	1	1	1	1	1	1	1	1	1	1	1	2	1	2	1	2	2	–	–	–	–	–	–	–	–	–	c					
16+19																	1	1	1	2	1	2	2	1	1	–	–	–	–	–	–	–	–	–	c					
Serranochromis longimanus NHMUK 1910.5.31.62–64 Lectotype	0	1	2	1	1	1	1	1	1	1	1	1	1	1	1	1	1	1	1	1	2	2	1	2	–	–	–	–	–	–	–	–	c							
16+17																	1	2	1	1	2	2	1	1	–	–	–	–	–	–	–	–	c							
paralectotype	0	2	1	1	1	1	1	1	1	1	1	1	1	1	1	1	1	1	1	1	2	1	2	2	–	–	–	–	–	–	–	–	c							
16+17																	1	1	1	2	2	2	1	1	–	–	–	–	–	–	–	–	c							
paralectotype	0	2	1	1	1	1	1	1	1	1	1	1	1	1	1	1	1	1	1	1	2	1	2	2	–	–	–	–	–	–	–	–	c							
16+17																	1	1	2	1	2	2	1?	1?	–	–	–	–	–	–	–	–	c							
paralectotype	0	2	1	1	1	1	1	1	1	1	1	1	1	1	1	1	1	1	1	1	2	1	2	1	–	–	–	–	–	–	–	–	c							
16+17																	1	1	1	2	2	2	2	–	–	–	–	–	–	–	–	–	c							
Serranochromis macrocephalus NHMUK 1920.5.26.164	0	2	1	1	1	1	1	1	1	1	1	1	1	1	1	1	1	1	1	1	2	1	2	2	1	–	–	–	–	–	–	–	c							
15+18																1	–	1	1	1	2	2	1	1	–	–	–	–	–	–	–	–	c							
Serranochromis meridianus NHMUK 1967.4.14.1	0	2	1	1	1	1	1	1	1	1	1	1	1	1	1	1	1	1	1	1	2	1	2	2	–	–	–	–	–	–	–	–	c							
15+18																1	–	1	1	2	1	2	2	1	–	–	–	–	–	–	–	–	c							
Serranochromis robustus AMNH 31877	0	2	1	1	1	1	1	1	1	1	1	1	1	1	1	1	1	1	1	2	1	2	1	2	1	–	–	–	–	–	–	–	–	c						
16+18																	1	1	1	2	1	2	2	1	–	–	–	–	–	–	–	–	–	c						
AMNH 31879	0	2	1	1	1	1	1	1	1	1	1	1	1	1	1	1	1	1	1	1	2	1	1	2	2	1	–	–	–	–	–	–	–	–	–	c				
17+19																		1	1	1	1	2	1	2	2	1	–	–	–	–	–	–	–	–	–	c				
NHMUK 1935.6.14.2330-2337 L5	0	2	1	1	1	1	1	1	1	1	1	1	1	1	1	1	1	1	1	1	1	2	2	1	2	1	–	–	–	–	–	–	–	–	c					
17+18																		1	1	2	1	1	2	2	1	–	–	–	–	–	–	–	–	–	c					
L6	0	2	1	1	1	1	1	1	1	1	1	1	1	1	1	1	1	1	1	2	1	2	1	2	1	–	–	–	–	–	–	–	–	c						
16+18																	1	1	1	1	2	2	1	2	–	–	–	–	–	–	–	–	–	c						

Species, Museum, Catalog number / ID — Vertebral count	1	2	3	4	5	6	7	8	9	10	11	12	13	14	15	16	17	18	19	20	21	22	23	24	25	26	27	28	29	30	31	32	33	34	35	36	37	38	39	40
R1	0	2	1	1	1	1	1	1	1	1	1	1	1	1	1	1	1	1	1	1	2	1	2	1	2	1	–	–	–	–	–	–	–	–	c					
16+19																	1	–	2	1	1	1	2	2	1?	–	–	–	–	–	–	–	–	–	c					
R2	0	2	1	1	1	1	1	1	1	1	1	1	1	1	1	1	1	1	1	1	2	1	2	2	2	–	–	–	–	–	–	–	–	–	c					
16+19																	1	–	1	2	1	2	2	1	1	–	–	–	–	–	–	–	–	–	c					
Thoracochromis albolabris NHMUK 1972.9.27.89	0	2	1	1	1	1	1	1	1	1	1	1	1	1	1	1	1	1	2	1	2	2	–	–	–	–	–	–	–	–	–	c								
13+19														1	–	1	1	–	2	1	2	1	–	–	–	–	–	–	–	–	–	c								
Thoracochromis wingatii MCZ 148133	0	2	1	1	1	1	1	1	1	1	1	1	1	1	1	1	1	1	2	1	1	1	–	–	–	–	–	–	c											
13+16														1	1	–	1	2	1	1	2	–	–	–	–	–	–	–	c											
UMMZ 166656 1st	0-1	2	1	1	1	1	1	1	1	1	1	1	1	1	1	1	1	1	2	2	–	–	–	–	–	–	–	c												
12+16													2	–	–	1	2	1	2	1	–	–	–	–	–	–	–	c												
**Steatocranini**																																								
Steatocranus casuarius USNM 331489 1 (small)	0	2	1	1	1	1	1	1	1	1	1	1	1	1	1	1	1	1	1	1	1	2	1	2	2	–	–	–	–	–	c									
15+16																1	–	–	1	1	1	2	1	–	–	–	–	–	–	–	c									
2	0	2	1	1	1	1	1	1	1	1	1	1	1	1	1	1	1	1	1	1	1	2	1	2	1	–	–	–	–	c										
16+14																	1	–	1	1	1	2	1	–	–	–	–	–	–	c										
3	0	2	1	1	1	1	1	1	1	1	1	1	1	1	1	1	1	1	1	1	1	2	1	2	1	–	–	–	–	–	c									
15+16																1	–	–	1	1	1	2	2	–	–	–	–	–	–	–	c									
**Tilapiini**																																								
Chilochromis duponti CUMV 96467	0	2	1	1	1	1	1	1	1	1	1	1	1	1	1	1	1	2	2	2	2	1	–	–	–	–	–	c												
15+13																1	1	1	2	2	2	–	–	–	–	–	–	c												
Congolapia bilineata AMNH 238673	0	2	1	1	1	1	1	1	1	1	1	1	1	1	1	1	1	2	1	3	2	–	–	–	–	–	–	c												
15+13																1	1	1	2	2	2	–	–	–	–	–	–	c												
	0	2	1	1	1	1	1	1	1	1	1	1	1	1	1	1	2	1	2	2	1	–	–	–	–	–	–	c												
14+14															1	–	1	2	1	2	1	–	–	–	–	–	–	c												
	0	2	1	1	1	1	1	1	1	1	1	1	1	1	1	1	1	2	2	2	2	–	–	–	–	–	–	c												
14+14															1	–	1	1	2	2	1	–	–	–	–	–	–	c												
Tilapia busumana NHMUK 1942.12.30.52-61 L1	0	2	1	1	1	1	1	1	1	1	1	1	1	1	1	1	2	2	2	2	2	–	–	–	–	–	c													
14+13															1	–	1	1	2	2	1?	–	–	–	–	–	c													
L2	0	2	1	1	1	1	1	1	1	1	1	1	1	1	1	1	2	2	2	2	2	–	–	–	–	–	c													
14+13															1	–	1	2	2	2	1?	–	–	–	–	–	c													
middle column 1	0	2	1	1	1	1	1	1	1	1	1	1	1	1	1	2	2	2	2	–	–	–	–	–	–	–	c													
14+13															1	1	1	2	2	2	–	–	–	–	–	–	c													
middle column 2	0	–	3	1	1	1	1	1	1	1	1	1	1	1	1	1	2	1	2	2	2	1	–	–	–	–	c													
14+13															1	–	1	1	2	2	1	–	–	–	–	–	c													
Tilapia sparrmanii CUMV 91199	0	2	1	1	1	1	1	1	1	1	1	1	1	1	1	1	1	2	1	3	1	–	–	–	–	–	c													
14+13															2	–	1	2	2	2	1	–	–	–	–	–	c													
NHMUK 1859.5.7.85 lectotype	0	2	1	1	1	1	1	1	1	1	1	1	1	1	1	1	2	2	2	1	–	–	–	–	–	c														
13+13														1	1	2	1	2	2	2	–	–	–	–	–	c														
NHMUK 1859.5.7.46	0	2	1	1	1	1	1	1	1	1	1	1	1	1	1	1	2	1	2	2	1	–	–	–	–	–	c													
14+13															1	1	2	1	2	3	–	–	–	–	–	–	c													
	0	2	1	1	1	1	1	1	1	1	1	1	1	1	1	1	2	1	2	2	1	–	–	–	–	–	c													
14+13															1	1	2	1	2	2	1	–	–	–	–	–	c													
**Lake Barombi Mbo**																																								
**Oreochromini**																																								
Konia eisentrauti CUMV 93584	0	2	1	1	1	1	1	1	1	1	1	1	1	1	1	1	1	1	2	2	2	1	–	–	–	–	–	–	c											
14+15															1	–	1	2	1	2	2	–	–	–	–	–	–	–	c											
UMMZ 220255 1	0	2	1	1	1	1	1	1	1	1	1	1	1	1	1	1	1	2	1	2	2	1	–	–	–	–	–	–	c											
14+15															1	–	2	1	2	2	2	–	–	–	–	–	–	–	c											
2	0	2	1	1	1	1	1	1	1	1	1	1	1	1	1	1	1	1	2	2	2	1	–	–	–	–	–	–	c											
14+15															1	–	1	1	2	2	2	–	–	–	–	–	–	–	c											
3	0	2	1	1	1	1	1	1	1	1	1	1	1	1	1	1	1	1	2	2	2	1	–	–	–	–	–	–	c											
14+15															1	–	1	2	2	2	1	–	–	–	–	–	–	–	c											
4	0	2	1	1	1	1	1	1	1	1	1	1	1	1	1	1	1	2	1	2	2	2	–	–	–	–	–	c												
14+14															1	–	1	2	1	2	3	–	–	–	–	–	–	c												
Myaka myaka AMNH 271183	0	2	1	1	1	1	1	1	1	1	1	1	1	1	1	1	1	2	2	2	2	–	–	–	–	–	–	–	c											
14+15															1	1	1	2	2	2	2	–	–	–	–	–	–	–	c											
	0	2	1	1	1	1	1	1	1	1	1	1	1	1	1	1	1	2	1	3	2	1	–	–	–	–	–	–	c											
14+15															2	–	1	1	2	2	2	–	–	–	–	–	–	–	c											
	0	2	1	1	1	1	1	1	1	1	1	1	1	1	1	1	1	2	1	3	2	–	–	–	–	–	–	–	c											
14+15															1	–	2	1	2	2	2	–	–	–	–	–	–	–	c											
	0	2	1	1	1	1	1	1	1	1	1	1	1	1	1	1	1	2	2	2	2	–	–	–	–	–	–	–	c											
14+15															1	1	1	2	2	2	1	–	–	–	–	–	–	–	c											
Pungu maclareni CUMV 93585	0	2	1	1	1	1	1	1	1	1	1	1	1	1	1	1	1	1	2	2	2	1	–	–	–	–	–	–	c											
15+14																1	1	1	1	2	3	1	–	–	–	–	–	–	c											
NHMUK 1959.8.18.177 holotype	0	2	1	1	1	1	1	1	1	1	1	1	1	1	1	1	1	1	2	1	2	2	–	–	–	–	–	–	c											
15+14																1	1	1	1	2	2	2	–	–	–	–	–	–	c											
Stomatepia mariae CUMV 93588	0	2	1	1	1	1	1	1	1	1	1	1	1	1	1	1	2	2	2	2	–	–	–	–	–	–	–	c												
13+15														1	–	1	2	2	2	2	–	–	–	–	–	–	–	c												

Species, Museum, Catalog number / ID Vertebral count	1	2	3	4	5	6	7	8	9	10	11	12	13	14	15	16	17	18	19	20	21	22	23	24	25	26	27	28	29	30	31	32	33	34	35	36	37	38	39	40
Stomatepia pindu UMMZ 220257 1	0	2	1	1	1	1	1	1	1	1	1	1	1	1	1	1	1	2	2	2	2	–	–	–	–	–	–	c												
14+14															1	1	1	2	2	3	1	–	–	–	–	–	–	c												
2	0	2	1	1	1	1	1	1	1	1	1	1	1	1	1	1	1	2	2	3	–	–	–	–	–	–	–	c												
13+15														1	–	1	2	2	2	2	–	–	–	–	–	–	–	c												
3	0	2	1	1	1	1	1	1	1	1	1	1	1	1	1	1	2	2	2	1	–	–	–	–	–	–	c													
13+14														1	–	2	1	2	3	1	–	–	–	–	–	–	c													
4	0	2	1	1	1	1	1	1	1	1	1	1	1	1	1	1	2	1	3	2	–	–	–	–	–	–	–	c												
14+14															1	2	1	2	2	2	–	–	–	–	–	–	–	c												
**Lake Fwa**																																								
**Pseudocrenilabrini**																																								
Cyclopharynx schwetzi AMNH 247750 left 1	0	2	1	1	1	1	1	1	1	1	1	1	1	1	1	1	1	1	1	2	2	2	–	–	–	–	–	–	–	c										
14+16															1	–	1	1	2	2	2	–	–	–	–	–	–	–	–	c										
left 2	0	2	1	1	1	1	1	1	1	1	1	1	1	1	1	1	1	1	2	1	2	2	–	–	–	–	–	–	c											
14+15															2	–	–	2	1	2	2	–	–	–	–	–	–	–	c											
left 3	0	2	1	1	1	1	1	1	1	1	1	1	1	1	1	1	1	1	1	2	2	2	–	–	–	–	–	–	c											
14+15															1	–	2	–	2	2	2	–	–	–	–	–	–	–	c											
left 4	0	2	1	1	1	1	1	1	1	1	1	1	1	1	1	1	1	1	1	2	2	2	–	–	–	–	–	–	c											
14+15															1	–	1	1	2	2	2	–	–	–	–	–	–	–	c											
left 5	0	1	2	1	1	1	1	1	1	1	1	1	1	1	1	1	1	1	1	2	1	3	1	–	–	–	–	–	c											
15+14																2	–	1	1	2	3	1	–	–	–	–	–	–	c											
left 6	0	2	1	1	1	1	1	1	1	1	1	1	1	1	1	1	1	1	1	2	1	3	–	–	–	–	–	–	–	c										
15+15																1	1	1	1	2	2	1	–	–	–	–	–	–	–	c										
left 7	0	2	1	1	1	1	1	1	1	1	1	1	1	1	1	1	1	1	2	1	3	–	–	–	–	–	–	–	c											
14+15															1	1	1	1	2	2	1	–	–	–	–	–	–	–	c											
right 1	0	2	1	1	1	1	1	1	1	1	1	1	1	1	1	1	1	1	1	2	2	2	–	–	–	–	–	–	c											
14+15															1	–	1	1	2	2	2	–	–	–	–	–	–	–	c											
right 2	0	2	1	1	1	1	1	1	1	1	1	1	1	1	1	1	1	1	2	1	3	–	–	–	–	–	–	–	c											
14+15															2	1	–	1	2	2	1	–	–	–	–	–	–	–	c											
right 3	0	2	1	1	1	1	1	1	1	1	1	1	1	1	1	1	1	1	1	2	3	1	–	–	–	–	–	–	c											
14+15															1	–	1	2	1	2	2	–	–	–	–	–	–	–	c											
right 4	0	2	1	1	1	1	1	1	1	1	1	1	1	1	1	1	1	1	1	2	2	2	–	–	–	–	–	–	c											
14+15															1	–	1	1	2	2	2	–	–	–	–	–	–	–	c											
right 5	0	2	1	1	1	1	1	1	1	1	1	1	1	1	1	1	1	1	1	2	2	2	–	–	–	–	–	–	c											
14+15															2	–	–	1	2	2	2	–	–	–	–	–	–	–	c											
right 6	0	2	1	1	1	1	1	1	1	1	1	1	1	1	1	1	1	1	2	1	2	1	–	–	–	–	–	–	c											
14+15															1	–	2	1	1	2	2	–	–	–	–	–	–	–	c											
right 7	0	2	1	1	1	1	1	1	1	1	1	1	1	1	1	1	1	1	2	1	2	1	–	–	–	–	–	–	c											
14+15															1	2	–	1	2	2	1	–	–	–	–	–	–	–	c											
Schwetzochromis neodon AMNH 247754	0	2	1	1	1	1	1	1	1	1	1	1	1	1	1	1	1	1	1	1	2	1	2	2	–	–	–	–	–	–	–	c								
14+18															1	–	1	1	1	1	1	2	2	–	–	–	–	–	–	–	–	c								
AMNH 261775	0	2	1	1	1	1	1	1	1	1	1	1	1	1	1	1	1	1	1	1	2	1	2	2	–	–	–	–	–	–	–	c								
14+18															1	–	1	1	1	1	1	2	1	1	–	–	–	–	–	–	–	c								
**Lake Albert**																																								
**Pseudocrenilabrini**																																								
Haplochromis avium NHMUK 1929.2.15.4-8 top R	0	2	1	1	1	1	1	1	1	1	1	1	1	1	1	1	1	1	2	1	2	–	–	–	–	–	–	–	c											
paratypes 13+16														1	1	–	1	2	2	1	–	–	–	–	–	–	–	–	c											
bottom L	0	2	1	1	1	1	1	1	1	1	1	1	1	1	1	1	1	1	2	1	–	–	–	–	–	–	–	–	c											
12+17													2	–	–	1	1	1	2	1	–	–	–	–	–	–	–	–	c											
bottom R	0	2	1	1	1	1	1	1	1	1	1	1	1	1	1	1	1	1	2	2?	–	–	–	–	–	–	–	–	c											
12+17													2	1	–	1	1	2	1	–?	–	–	–	–	–	–	–	–	c											
Haplochromis loati NHMUK 1909.7.27.42	0	2	1	1	1	1	1	1	1	1	1	1	1	1	1	1	1	1	2	1	2	–	–	–	–	–	–	–	c											
13+16														1	–	1	1	2	1	2	2	–	–	–	–	–	–	–	c											
**Lake Edward–George system**																																								
**Pseudocrenilabrini**																																								
Haplochromis akika NHMUK 1972.6.2.784 holotype	0	2	1	1	1	1	1	1	1	1	1	1	1	1	1	1	1	1	2	2	1	–	–	–	–	–	–	–	–	c										
13+17														2	–	1	1	1	2	1	2	–	–	–	–	–	–	–	–	c										
NHMUK 1972.6.2.785-787 paratypes top	0	2	1	1	1	1	1	1	1	1	1	1	1	1	1	1	1	1	2	1	2	–	–	–	–	–	–	–	–	c										
13+17														2	–	1	1	1	2	1	2	–	–	–	–	–	–	–	–	c										
second	0	2	1	1	1	1	1	1	1	1	1	1	1	1	1	1	1	1	1	2	2	1	–	–	–	–	–	–	–	c										
13+17														1	–	1	1	2	1	1	2	2	–	–	–	–	–	–	–	c										
third	0	2	1	1	1	1	1	1	1	1	1	1	1	1	1	1	1	1	2	1	2	1	–	–	–	–	–	–	–	c										
13+17														2	–	–	1	2	1	2	2	–	–	–	–	–	–	–	–	c										
Haplochromis aquila RMCA 2018.008.P.0355 holotype	0	2	1	1	1	1	1	1	1	1	1	1	1	1	1	1	1	1	2	1	3	–	–	–	–	–	–	–	–	–	c									
13+18														1	1	1	–	2	1	2	2	–	–	–	–	–	–	–	–	–	c									
Haplochromis aureus RMCA 2019.002.P.0136 holotype	0	2	1	1	1	1	1	1	1	1	1	1	1	1	1	1	1	1	1	2	2	1	–	–	–	–	–	–	–	–	–	c								
14+18															2	–	1	1	2	1	2	1	–	–	–	–	–	–	–	–	–	c								
Haplochromis curvidens RMCA 2016.035.P.0219 holotype	0	2	1	1	1	1	1	1	1	1	1	1	1	1	1	1	1	1	2	1	3	–	–	–	–	–	–	–	–	c										
13+17														2	–	–	1	2	1	2	2	–	–	–	–	–	–	–	–	c										

Species, Museum, Catalog number / ID<br>Vertebral count	1	2	3	4	5	6	7	8	9	10	11	12	13	14	15	16	17	18	19	20	21	22	23	24	25	26	27	28	29	30	31	32	33	34	35	36	37	38	39	40
Haplochromis falcatus RMCA 2018.008.P.0401 holotype	0	2	1	1	1	1	1	1	1	1	1	1	1	1	1	1	1	1	2	2	1	–	–	–	–	–	–	–	–	c										
13+17														2	–	1	2	1	2	1	2	–	–	–	–	–	–	–	–	c										
Haplochromis fuscus NHMUK 1925.8.7.11 paratype	0	2	1	1	1	1	1	1	1	1	1	1	1	1	1	1	1	1	2	1	2	1	–	–	–	–	–	–	–	c										
13+17														1	–	1	1	1	1	2	1	2	–	–	–	–	–	–	–	c										
Haplochromis glaucus RMCA 2019.002.P.0016 holotype	0	2	1	1	1	1	1	1	1	1	1	1	2	–	1	1	1	1	1	2	2	–	–	–	–	–	–	–	–	c										
13+17														1	–	1	1	1	2	2	2	–	–	–	–	–	–	–	–	c										
Haplochromis gracilifur RMCA 2017.006.P.0004 holotype	0	2	1	1	1	1	1	1	1	1	1	1	1	1	1	1	1	1	2	1	2	–	–	–	–	–	–	–	–	c										
13+17														1	–	1	1	2	1	2	1	–	–	–	–	–	–	–	–	c										
Haplochromis kimondo RMCA 2019.002.P.0015 holotype	0	2	1	1	1	1	1	1	1	1	1	1	1	1	1	1	1	1	1	2	1	2	–	–	–	–	–	–	–	–	c									
13+18														1	–	1	1	1	2	2	1	1	–	–	–	–	–	–	–	–	c									
Haplochromis labiatus NHMUK 1972.6.2.809	0	2	1	1	1	1	1	1	1	1	1	1	1	1	1	1	1	1	1	2	1	2	–	–	–	–	–	–	–	c										
13+17														1	–	1	1	1	2	1	2	1	–	–	–	–	–	–	–	c										
Haplochromis latifrons RMCA 2018.008.P.0330 holotype	0	2	1	1	1	1	1	1	1	1	1	1	1	1	1	1	1	1	2	2	1	–	–	–	–	–	–	–	–	–	c									
14+17															1	1	1	2	1	2	1	–	–	–	–	–	–	–	–	–	c									
Haplochromis limax NHMUK 1972.6.2.136–139 1	0	2	1	1	1	1	1	1	1	1	1	1	1	1	1	1	1	1	2	2	2	–	–	–	–	–	–	–	c											
13+16														1	1	1	1	1	2	1	2	–	–	–	–	–	–	–	c											
2	0	2	1	1	1	1	1	1	1	1	1	1	1	1	1	1	1	2	1	2	1	–	–	–	–	–	–	–	c											
13+16														2	–	1	1	1	2	2	1	–	–	–	–	–	–	–	c											
3	0	2	1	1	1	1	1	1	1	1	1	1	1	1	1	1	1	1	2	1	2	1	–	–	–	–	–	–	c											
13+16														1	1	1	1	1	2	2	2	–	–	–	–	–	–	–	c											
4	0	1	2	1	1	1	1	1	1	1	1	1	1	1	1	1	1	1	1	2	2	1	–	–	–	–	–	–	–	c										
13+17														1	–	1	1	1	2	1	2	1	–	–	–	–	–	–	–	c										
Haplochromis mentatus MCZ 31523 holotype	0	2	1	1	1	1	1	1	1	1	1	1	1	1	1	1	1	1	1	2	1	2	–	–	–	–	–	–	–	–	c									
14+17															1	1	1	1	1	2	2	1	–	–	–	–	–	–	–	–	c									
Haplochromis molossus RMCA 2016.035.P.0046 holotype	0	2	1	1	1	1	1	1	1	1	1	1	1	1	1	1	1	1	2	1	2	–	–	–	–	–	–	–	–	c										
14+16															1	1	1	1	2	1	2	–	–	–	–	–	–	–	–	c										
Haplochromis pappenheimi ZMB 19110 lectotype	0	2	1	1	1	1	1	1	1	1	1	1	1	1	1	1	1	1	2	1	2	–	–	–	–	–	–	–	–	–	c									
14+17															1	1	1	1	2	1	2	–	–	–	–	–	–	–	–	–	c									
Haplochromis paradoxus RMCA 2017.006.P.0011 holotype	0	1	2	1	1	1	1	1	1	1	1	1	1	1	1	1	1	1	1	2	2	1	–	–	–	–	–	–	–	c										
14+16															1	1	1	1	1	2	2	–	–	–	–	–	–	–	–	c										
Haplochromis pardus RMCA 2016.035.P.0202 holotype	0	2	1	1	1	1	1	1	1	1	1	1	1	1	1	1	1	1	2	1	2	1	–	–	–	–	–	–	–	c										
13+17														1	1	–	2	1	1	2	2	–	–	–	–	–	–	–	–	c										
Haplochromis pelagicus RMCA 2019.002.P.0138 holotype	0	2	1	1	1	1	1	1	1	1	1	1	1	1	1	1	1	1	1	1	2	1	2	1	–	–	–	–	–	–	–	c								
15+17																1	1	1	2	1	2	2	–	–	–	–	–	–	–	–	–	c								
Haplochromis pharyngalis RMCA 65724 lectotype	0	2	1	1	1	1	1	1	1	1	1	1	1	1	1	1	1	1	1	2	1	2	–	–	–	–	–	–	–	–	c									
14+17															1	1	1	–	2	1	2	2	–	–	–	–	–	–	–	–	c									
Haplochromis quasimodo RMCA 2018.008.P.0336 holotype	0	2	1	1	1	1	1	1	1	1	1	1	1	1	1	1	1	1	1	2	2	1	–	–	–	–	–	–	–	–	c									
14+17															1	1	1	1	2	1	3	–	–	–	–	–	–	–	–	–	c									
Haplochromis relictidens RMCA 2017.006.P.0007 holotype	0	2	1	1	1	1	1	1	1	1	1	1	1	1	1	1	1	1	2	2	1	–	–	–	–	–	–	–	–	c										
13+17														1	2	–	1	1	1	2	1	–	–	–	–	–	–	–	–	c										
Haplochromis rex RMCA 2018.008.P.0345 holotype	0	2	1	1	1	1	1	1	1	1	1	1	1	1	1	1	1	1	2	1	2	1	–	–	–	–	–	–	–	c										
13+17														1	1	1	1	1	2	1	2	–	–	–	–	–	–	–	–	c										
Haplochromis simba RMCA 2016.035.P.0225 holotype	0	2	1	1	1	1	1	1	1	1	1	1	1	1	1	1	1	1	2	1	2	1?	–	–	–	–	–	–	–	–	–	c								
14+18															1	1	1	1	1	2	2	1	–	–	–	–	–	–	–	–	–	c								
Haplochromis squamipinnis NHMUK 1914.4.8.32 holotype	0	2	1	1	1	1	1	1	1	1	1	1	1	1	1	1	1	1	1	2	2	1	–	–	–	–	–	–	–	–	c									
13+18														1	–	1	1	2	1	1	2	1	–	–	–	–	–	–	–	–	c									
Haplochromis taurinus NHMUK 1933.2.23.406 holotype	0	2	1	1	1	1	1	1	1	1	1	1	1	1	1	1	1	1	1	2	1	2	–	–	–	–	–	–	–	–	c									
14+17															2	–	1	1	1	2	1	2	–	–	–	–	–	–	–	–	c									
Schubotzia eduardiana NHMUK 1987.2.25.28–30	0	2	1	1	1	1	1	1	1	1	1	1	1	1	1	1	1	1	2	1	2	1	–	–	–	–	–	–	–	c										
13+17														1	1	–	1	2	1	2	2	–	–	–	–	–	–	–	–	c										
	0	2	1	1	1	1	1	1	1	1	1	1	1	1	1	1	1	1	2	1	2	–	–	–	–	–	–	–	–	c										
13+17														2	–	–	1	2	1	2	1	–	–	–	–	–	–	–	–	c										
	0	2	1	1	1	1	1	1	1	1	1	1	1	1	1	1	1	1	1	2	2	1	–	–	–	–	–	–	–	c										
14+16															1	1	1	1	1	1	2	1	–	–	–	–	–	–	–	c										
NHMUK 1987.2.25.381–387 L1	0	2	1	1	1	1	1	1	1	1	1	1	1	1	1	1	1	1	1	2	2	–	–	–	–	–	–	–	–	c										
13+17														1	–	1	1	1	2	1	2	–	–	–	–	–	–	–	–	c										
L2	0	2	1	1	1	1	1	1	1	1	1	1	1	1	1	1	1	1	2	1	2	–	–	–	–	–	–	–	–	c										
13+17														1	–	1	1	1	2	2	1	–	–	–	–	–	–	–	–	c										
L3	0	2	1	1	1	1	1	1	1	1	1	1	1	1	1	1	1	1	1	2	2	1	–	–	–	–	–	–	–	–	c									
13+18														2	–	–	1	2	1	1	2	1	–	–	–	–	–	–	–	–	c									
L4	0	2	1	1	1	1	1	1	1	1	1	1	1	1	1	1	1	1	1	2	2	1	–	–	–	–	–	–	–	c										
13+17														1	–	1	1	2	1	2	2	–	–	–	–	–	–	–	–	c										
R1	0	2	1	1	1	1	1	1	1	1	1	1	1	1	1	1	1	1	2	1	2	–	–	–	–	–	–	–	–	c										
13+17														1	–	1	1	2	1	2	2	–	–	–	–	–	–	–	–	c										
R2	0	2	1	1	1	1	1	1	1	1	1	1	1	1	1	1	1	1	2	1	2	1	–	–	–	–	–	–	–	c										
13+17														1	–	1	1	2	1	2	2	–	–	–	–	–	–	–	–	c										
R3	0	2	1	1	1	1	1	1	1	1	1	1	1	1	1	1	1	1	2	1	2	–	–	–	–	–	–	–	–	c										
13+17														1	–	1	1	2	1	2	1	–	–	–	–	–	–	–	–	c										

Species, Museum, Catalog number / ID<br>Vertebral count	1	2	3	4	5	6	7	8	9	10	11	12	13	14	15	16	17	18	19	20	21	22	23	24	25	26	27	28	29	30	31	32	33	34	35	36	37	38	39	40
Lake Victoria & satellites																																								
**Pseudocrenilabrini**																																								
Allochromis welcommei MCZ 152827	0	2	1	1	1	1	1	1	1	1	1	1	1	1	1	1	1	1	1	2	2	1	–	–	–	–	–	–	–	–	c									
13+18														2	–	–	1	1	2	1	2	2	–	–	–	–	–	–	–	–	c									
Astatoreochromis alluaudi YPM 003830	0	2	1	1	1	1	1	1	1	1	1	1	1	1	1	1	1	1	1	2	1	3	–	–	–	–	–	–	–	–	c									
14+17															1	1	1	1	2	2	1	2	–	–	–	–	–	–	–	–	c									
Haplochromis chlorochrous Paratypes top center	0	2	1	1	1	1	1	1	1	1	1	1	1	1	1	1	1	2	1	1	2	–	–	–	–	–	–	–	–	–	c									
NHMUK 1968.8.30.311-328 13+18														2	–	1	2	1	1	2?	–	–	–	–	–	–	–	–	–	–	c									
top right	0	2	1	1	1	1	1	1	1	1	1	1	1	1	1	1	1	1	2	1	2	–	–	–	–	–	–	–	–	c										
13+17														1	1	1	1	1	2	2	1?	–	–	–	–	–	–	–	–	c										
Right column 2nd	0	1	2	1	1	1	1	1	1	1	1	1	1	1	1	1	1	1	2	2	1	–	–	–	–	–	–	–	–	–	c									
13+18														1	1	1	1	1	1	2	1	–	–	–	–	–	–	–	–	–	c									
Right column 3rd	0	2	1	1	1	1	1	1	1	1	1	1	1	1	1	1	1	2	1	2	–	–	–	–	–	–	–	–	–	–	c									
13+18														1	1	1	1	1	2	2	–	–	–	–	–	–	–	–	–	–	c									
Haplochromis cryptogramma NHMUK 1968.8.30.198-214	0	2	1	1	1	1	1	1	1	1	1	1	1	1	1	1	1	1	2	1	1	–	–	–	–	–	–	–	–	–	–	c								
paratypes L column 1 13+19														2	–	1	1	2	1	2	–	–	–	–	–	–	–	–	–	–	–	c								
L 2	0	2	1	1	1	1	1	1	1	1	1	1	1	1	1	1	1	2	1	2	1	–	–	–	–	–	–	–	–	–	c									
12+19													1	–	1	1	1	2	1	2	–	–	–	–	–	–	–	–	–	–	c									
L3	0	2	1	1	1	1	1	1	1	1	1	1	1	1	1	1	1	2	1	2	1	–	–	–	–	–	–	–	–	–	c									
13+18														2	–	1	1	1	2	2	–	–	–	–	–	–	–	–	–	–	c									
L4	0	2	1	1	1	1	1	1	1	1	1	1	1	1	1	1	1	2	2	1?	–	–	–	–	–	–	–	–	–	–	c									
13+18														1	1	1	2	1	1	2?	–	–	–	–	–	–	–	–	–	–	c									
middle column 1	0	2	1	1	1	1	1	1	1	1	1	1	1	1	1	1	1	2	1	2	1	–	–	–	–	–	–	–	–	–	c									
13+18														1	1	1	1	1	2	2	–	–	–	–	–	–	–	–	–	–	c									
mid 2	0	2	1	1	1	1	1	1	1	1	1	1	1	1	1	1	1	1	2	2	1	–	–	–	–	–	–	–	–	–	c									
13+18														1	–	1	1	2	1	2	1	–	–	–	–	–	–	–	–	–	c									
mid 3	0	2	1	1	1	1	1	1	1	1	1	1	1	1	1	1	1	2	1	2	–	–	–	–	–	–	–	–	–	c										
13+17														1	1	1	1	2	2	1	–	–	–	–	–	–	–	–	–	c										
R1	0	–	2	1	1	1	1	1	1	1	1	1	1	1	1	1	1	1	2	2	–	–	–	–	–	–	–	–	–	–	c									
14+17															1	1	1	1	2	2	1	–	–	–	–	–	–	–	–	–	c									
R2	0	2	1	1	1	1	1	1	1	1	1	1	1	1	1	1	1	1	2	2	–	–	–	–	–	–	–	–	–	–	c									
13+18														1	1	1	1	1	2	2	–	–	–	–	–	–	–	–	–	–	c									
R3	0	2	1	1	1	1	1	1	1	1	1	1	1	1	1	1	1	1	2	2	1	–	–	–	–	–	–	–	–	–	c									
13+18														1	1	–	1	1	2	2	1?	–	–	–	–	–	–	–	–	–	c									
Haplochromis latifasciatus NHMUK 1929.1.24.335-339 L1	0	2	1	1	1	1	1	1	1	1	1	1	1	1	1	1	1	1	2	1	2	–	–	–	–	–	–	–	–	c										
13+17														1	–	1	1	2	1	2	2	–	–	–	–	–	–	–	–	c										
L2	0	2	1	1	1	1	1	1	1	1	1	1	1	1	1	1	1	1	2	1	2	–	–	–	–	–	–	–	–	c										
13+17														1	1	–	1	1	2	1	2	–	–	–	–	–	–	–	–	c										
L3	0	2	1	1	1	1	1	1	1	1	1	1	1	1	1	1	1	1	2	1	2	1	–	–	–	–	–	–	–	c										
13+17														1	–	1	1	1	2	2	2	–	–	–	–	–	–	–	–	c										
R1	0	–	2	1	1	1	1	1	1	1	1	1	1	1	1	1	1	1	2	1	2	1	–	–	–	–	–	–	–	–	c									
14+17															1	1	1	1	1	2	1	2	–	–	–	–	–	–	–	–	c									
R2	0	2	1	1	1	1	1	1	1	1	1	1	1	1	1	1	1	1	2	1	2	1	–	–	–	–	–	–	–	c										
13+17														1	–	1	1	1	2	2	2	–	–	–	–	–	–	–	–	c										
R3	0	2	1	1	1	1	1	1	1	1	1	1	1	1	1	1	1	1	2	1	2	1	–	–	–	–	–	–	–	c										
14+16															1	1	1	1	2	1	2	1	–	–	–	–	–	–	–	c										
Haplochromis lividus AMNH 20484	0	2	1	1	1	1	1	1	1	1	1	1	1	1	1	1	1	1	2	1	2	1?	–	–	–	–	–	–	–	c										
13+17														1	–	1	1	1	2	2	2	–	–	–	–	–	–	–	–	c										
	0	2	1	1	1	1	1	1	1	1	1	1	1	1	1	1	1	1	1	1	2	2	–	–	–	–	–	–	–	–	c									
14+17															2	–	–	1	1	2	2	2	–	–	–	–	–	–	–	–	c									
Haplochromis nubilus AMNH 13747	0	2	1	1	1	1	1	1	1	1	1	1	1	1	1	1	1	1	1	2	2	1	–	–	–	–	–	–	–	c										
13+17														2	–	–	1	1	2	2	2	–	–	–	–	–	–	–	–	c										
	0	2	1	1	1	1	1	1	1	1	1	1	1	1	1	1	1	1	1	2	1	2	–	–	–	–	–	–	–	c										
14+16															2	–	1	1	1	2	1	2	–	–	–	–	–	–	–	c										
NHMUK 1906.5.30.490 Lectotype	0	2	1	1	1	1	1	1	1	1	1	1	1	1	1	1	1	1	2	1	2	1	–	–	–	–	–	–	–	c										
13+17														1	1	1	1	1	2	2	2	–	–	–	–	–	–	–	–	c										
Haplochromis plutonius NHMUK 1977.1.10.40-48 1	0	2	1	1	1	1	1	1	1	1	1	1	1	1	1	1	1	1	2	3	–	–	–	–	–	–	–	–	–	–	c									
paratypes 12+19													1	–	1	1	1	1	2	2	–	–	–	–	–	–	–	–	–	–	c									
2	0	2	1	1	1	1	1	1	1	1	1	1	1	1	1	1	1	1	1	2	2	1	–	–	–	–	–	–	–	–	c									
13+18														1	–	1	1	1	2	2	2	–	–	–	–	–	–	–	–	–	c									
3	0	2	1	1	1	1	1	1	1	1	1	1	1	1	1	1	1	1	1	2	3	–	–	–	–	–	–	–	–	–	c									
14+17															1	1	1	1	2	2	2	–	–	–	–	–	–	–	–	–	c									
4	0	2	1	1	1	1	1	1	1	1	1	1	1	1	1	1	1	1	2	1	2	–	–	–	–	–	–	–	–	–	c									
13+18														1	1	1	1	1	2	2	1	–	–	–	–	–	–	–	–	–	c									
5	0	2	1	1	1	1	1	1	1	1	1	1	1	1	1	1	1	2	1	2	1	–	–	–	–	–	–	–	–	–	c									
13+18														2	–	1	1	1	2	2	–	–	–	–	–	–	–	–	–	–	c									
Lithochromis rubripinnis RMNH 33417	0	1	2	1	1	1	1	1	1	1	1	1	1	1	1	1	1	1	1	2	1	2	–	–	–	–	–	–	–	–	c									
13+18														1	–	1	1	1	1	2	2	1	–	–	–	–	–	–	–	–	c									

Species, Museum, Catalog number / ID<br>Vertebral count	1	2	3	4	5	6	7	8	9	10	11	12	13	14	15	16	17	18	19	20	21	22	23	24	25	26	27	28	29	30	31	32	33	34	35	36	37	38	39	40
RMNH 33418	0	2	1	1	1	1	1	1	1	1	1	1	1	1	1	1	1	1	1	2	1	2	–	–	–	–	–	–	–	c										
14+16															1	1	1	1	2	1	2	1	–	–	–	–	–	–	–	c										
RMNH 33420	0	2	1	1	1	1	1	1	1	1	1	1	1	1	1	1	1	1	1	2	1	2	–	–	–	–	–	–	–	c										
14+16															1	1	1	1	2	1	2	2	–	–	–	–	–	–	–	c										
RMNH 33423	0	2	1	1	1	1	1	1	1	1	1	1	1	1	1	1	1	1	1	2	2	1	–	–	–	–	–	–	–	–	c									
14+17															1	1	1	1	1	2	2	1	–	–	–	–	–	–	–	–	c									
Lithochromis xanthopteryx RMNH 33435	0	2	1	1	1	1	1	1	1	1	1	1	1	1	1	1	1	1	1	2	1	2	–	–	–	–	–	–	–	–	c									
14+17															1	1	1	1	1	2	1	2	–	–	–	–	–	–	–	–	c									
RMNH 33436	0	2	1	1	1	1	1	1	1	1	1	1	1	1	1	1	1	1	2	1	2	1?	–	–	–	–	–	–	–	–	c									
14+17															1	1	1	1	1	1	2	1	–	–	–	–	–	–	–	–	c									
RMNH 33437	0	2	1	1	1	1	1	1	1	1	1	1	1	1	1	1	1	1	2	1	2	1	–	–	–	–	–	–	–	–	c									
13+18														1	1	–	2	1	1	2	2	–	–	–	–	–	–	–	–	–	c									
RMNH 33438	0	1	2	1	1	1	1	1	1	1	1	1	1	1	1	1	1	1	1	2	1	2	1	–	–	–	–	–	–	–	c									
14+17															2	–	1	1	1	1	2	2	1	–	–	–	–	–	–	–	c									
Mbipia mbipi MCZ 137947	0	2	1	1	1	1	1	1	1	1	1	1	1	1	1	1	1	1	1	2	2	1	–	–	–	–	–	–	–	c										
14+16															1	1	1	1	2	1	1	1	–	–	–	–	–	–	–	c										
Neochromis nigricans AMNH 13743	0	2	1	1	1	1	1	1	1	1	1	1	1	1	1	1	1	1	1	2	1	2	1	–	–	–	–	–	–	c										
13+17														1	–	1	1	1	1	2	2	1	–	–	–	–	–	–	–	c										
Paralabidochromis victoriae AMNH 13746 det PHG	0	2	1	1	1	1	1	1	1	1	1	1	1	1	1	1	1	1	2	1	2	–	–	–	–	–	–	–	–	c										
13+17														2	–	–	1	2	1	2	2	–	–	–	–	–	–	–	–	c										
MCZ 152956	0	2	1	1	1	1	1	1	1	1	1	1	1	1	1	1	1	1	1	2	1	2?	–	–	–	–	–	–	–	–	c									
13+18														2	–	1	1	1	1	2	1	1?	–	–	–	–	–	–	–	–	c									
Pundamilia igneopinnis RMNH 33339-33340	0	2	1	1	1	1	1	1	1	1	1	1	1	1	1	1	1	1	1	1	2	2	1	–	–	–	–	–	–	–	–	c								
14+18															2	–	–	1	1	2	1	2	1	–	–	–	–	–	–	–	–	c								
	0	2	1	1	1	1	1	1	1	1	1	1	1	1	1	1	1	1	2	1	3	–	–	–	–	–	–	–	–	–	c									
14+17															1	1	1	2	1	2	2	–	–	–	–	–	–	–	–	–	c									
Pundamilia pundamilia RMNH 33380-33383	0	2	1	1	1	1	1	1	1	1	1	1	1	1	1	1	1	1	1	2	2	1	–	–	–	–	–	–	–	c										
13+17														1	–	1	1	1	1	2	2	1	–	–	–	–	–	–	–	c										
	0	2	1	1	1	1	1	1	1	1	1	1	1	1	1	1	1	1	1	2	1	2	–	–	–	–	–	–	–	–	c									
13+18														1	–	1	1	1	1	2	1	2	–	–	–	–	–	–	–	–	c									
	0	2	1	1	1	1	1	1	1	1	1	1	1	1	1	1	1	1	1	2	2	1	–	–	–	–	–	–	–	c										
13+17														1	–	1	1	1	2	1	2	1	–	–	–	–	–	–	–	c										
	0	2	1	1	1	1	1	1	1	1	1	1	1	1	1	1	1	1	2	1	2	–	–	–	–	–	–	–	c											
13+16														2	–	–	1	2	1	2	1	–	–	–	–	–	–	–	c											
Pyxichromis parorthostoma NHMUK 1966.3.9.253 paratype	0	2	1	1	1	1	1	1	1	1	1	1	1	1	1	1	1	1	2	2	2	–	–	–	–	–	–	–	c											
13+16														3	–	–	1	1	1	2	1	–	–	–	–	–	–	–	c											
Yssichromis fusiformis MCZ 136638	0	2	1	1	1	1	1	1	1	1	1	1	1	1	1	1	1	1	1	1	2	2	1	–	–	–	–	–	–	–	–	–	c							
15+18																1	1	1	1	2	2	2	–	–	–	–	–	–	–	–	–	–	c							
**Lake Kivu**																																								
**Pseudocrenilabrini**																																								
Haplochromis astatodon NHMUK 1906.9.6.125 Lectotype	0	2	1	1	1	1	1	1	1	1	1	1	1	1	1	1	1	1	2	1	2	–	–	–	–	–	–	–	–	c										
13+17														1	–	1	1	2	1	2	1	1	–	–	–	–	–	–	–	c										
NHMUK 1906.9.6.126-129 paralectotypes L1	0	2	1	1	1	1	1	1	1	1	1	1	1	1	1	1	1	1	2	1	2	–	–	–	–	–	–	–	–	c										
14+16															1	1	1	2	1	2	2	–	–	–	–	–	–	–	–	c										
L2	0	2	1	1	1	1	1	1	1	1	1	1	1	1	1	1	1	2	1	3	–	–	–	–	–	–	–	–	c											
13+16														2	–	1	1	2	1	2	–	–	–	–	–	–	–	–	c											
R1	0	2	1	1	1	1	1	1	1	1	1	1	1	1	1	1	1	1	2	2	1	–	–	–	–	–	–	–	c											
13+16														1	1	1	1	1	2	2	–	–	–	–	–	–	–	–	c											
R2	0	2	1	1	1	1	1	1	1	1	1	1	1	1	1	1	1	2	1	2	1	–	–	–	–	–	–	–	c											
13+16														1	1	1	1	1	2	2	1?	–	–	–	–	–	–	–	c											
R3	0	2	1	1	1	1	1	1	1	1	1	1	1	1	1	1	1	1	1	2	2	–	–	–	–	–	–	–	–	c										
14+16															1	1	1	1	2	2	2	–	–	–	–	–	–	–	–	c										
Haplochromis paucidens NHMUK 1906.9.6.72-73 syntypes	0	2	1	1	1	1	1	1	1	1	1	1	1	1	1	1	1	1	1	2	2	1	–	–	–	–	–	–	–	c										
14+16															1	1	1	1	2	2	1	–	–	–	–	–	–	–	–	c										
	0	2	1	1	1	1	1	1	1	1	1	1	1	1	1	1	1	1	2	1	2	1	–	–	–	–	–	–	c											
14+15															1	1	1	1	1	2	2	–	–	–	–	–	–	–	c											
**Lake Turkana**																																								
**Pseudocrenilabrini**																																								
Haplochromis rudolfianus NHMUK 1933.2.23.167	0	2	1	1	1	1	1	1	1	1	1	1	1	1	1	1	1	1	2	1	2	–	–	–	–	–	–	–	–	c										
13+17														2	–	–	1	1	2	2	2	–	–	–	–	–	–	–	–	c										
	0	2	1	1	1	1	1	1	1	1	1	1	1	1	1	1	1	1	1	2	1	2	–	–	–	–	–	–	–	–	c									
13+18														2	–	–	1	1	2	1	2	1	–	–	–	–	–	–	–	–	c									
NHMUK 1965.12.10.16–27 R1	0	2	1	1	1	1	1	1	1	1	1	1	1	1	1	1	1	1	1	2	2	1	–	–	–	–	–	–	–	c										
13+17														1	1	1	1	1	2	1	2	–	–	–	–	–	–	–	–	c										
R2	0	2	1	1	1	1	1	1	1	1	1	1	1	1	1	1	1	1	2	1	3	–	–	–	–	–	–	–	–	c										
14+16															1	1	1	1	2	2	2	–	–	–	–	–	–	–	–	c										
R3	0	2	1	1	1	1	1	1	1	1	1	1	1	1	1	1	1	1	2	1	2	–	–	–	–	–	–	–	–	c										
13+17														1	1	1	1	1	1	2	2	–	–	–	–	–	–	–	–	c										

Species, Museum, Catalog number / ID<br>Vertebral count	1	2	3	4	5	6	7	8	9	10	11	12	13	14	15	16	17	18	19	20	21	22	23	24	25	26	27	28	29	30	31	32	33	34	35	36	37	38	39	40
L1	0	2	1	1	1	1	1	1	1	1	1	1	1	1	1	1	1	1	1	2	2	–	–	–	–	–	–	–	–	c										
13+17														1	1	–	1	2	1	2	2	–	–	–	–	–	–	–	–	c										
L2	0	2	1	1	1	1	1	1	1	1	1	1	1	1	1	1	1	1	2	2	–	–	–	–	–	–	–	–	c											
13+16														1	1	1	1	2	1	2	1	–	–	–	–	–	–	–	c											
L3 large (below 2 small)	0	2	1	1	1	1	1	1	1	1	1	1	1	1	1	1	1	1	2	2	1	–	–	–	–	–	–	–	c											
13+16														1	1	1	1	1	1	2	2	–	–	–	–	–	–	–	c											
Haplochromis turkanae NHMUK 1973.11.20.2-4 1	0	2	1	1	1	1	1	1	1	1	1	1	1	1	1	1	1	1	2	2	–	–	–	–	–	–	–	–	c											
13+16														1	1	1	1	1	2	2	–	–	–	–	–	–	–	–	c											
2	0	2	1	1	1	1	1	1	1	1	1	1	1	1	1	1	1	1	2	2	–	–	–	–	–	–	–	–	c											
13+16?														2	–	–	1	2	1	2	–	–	–	–	–	–	–	–	c											
3	0	2	1	1	1	1	1	1	1	1	1	1	1	1	1	1	1	1	2	1	1	–	–	–	–	–	–	–	c											
13+16														1	–	2	1	1	1	2	1	–	–	–	–	–	–	–	c											
**Lake Tanganyika**																																								
**Bathybatini**																																								
Bathybates fasciatus UNIBAS GPB3	–	1	2	1	1	1	1	1	1	1	1	1	1	1	1	1	1	1	1	2	1	2	1	2	2	1	2	–	–	–	–	–	–	–	–	–	–	–	–	c
18+22																			1	2	2	2	2	2	2	2	2	1	–	–	–	–	–	–	–	–	–	–	–	c
UNIBAS ITH8	–	1	2	1	1	1	1	1	1	1	1	1	1	1	1	1	1	1	2	1	1	2	2	1	2	2	–?	–	–	–	–	–	–	–	–	–	–	–	c	
18+21																			1	2	3	1	2	2	2	2	2?	1?	–	–	–	–	–	–	–	–	–	–	c	
UNIBAS JEG4	–	1	2	1	1	1	1	1	1	1	1	1	1	1	1	1	1	1	2	1	1	2	1	2	2	1	1	–	–	–	–	–	–	–	–	–	–	–	–	c
18+22																			1	3	2	2	1	2	2	2	2	1	–	–	–	–	–	–	–	–	–	–	–	c
UNIBAS JYF6	–	1	2	1	1	1	1	1	1	1	1	1	1	1	1	1	1	1	1	2	1	1	2	1	2	2	2	–	–	–	–	–	–	–	–	–	–	–	–	c
18+22																			1	2	2	2	2	2	2	1	2	2	–	–	–	–	–	–	–	–	–	–	–	c
UNIBAS KYD1	–	1	2	1	1	1	1	1	1	1	1	1	1	1	1	1	1	1	1	2	1	1	2	2	1	2	2	–	–	–	–	–	–	–	–	–	–	–	–	c
18+22																			1	2	3	2	1	2	2	2	2	1	–	–	–	–	–	–	–	–	–	–	–	c
Bathybates ferox UNIBAS LCD6	0	1	2	1	1	1	1	1	1	1	1	1	1	1	1	1	2	1	1	2	1	2	1	2	1	–	–	–	–	–	–	–	–	–	c					
15+20																1	2	2	2	1	2	2	1	2	2	–	–	–	–	–	–	–	–	–	c					
UNIBAS LCD7	0	1	2	1	1	1	1	1	1	1	1	1	1	1	1	1	1	2	1	1	2	2	1	2	–	–	–	–	–	–	–	–	–	–	c					
15+20																1	1	3	1	2	2	2	1	2	2	–	–	–	–	–	–	–	–	–	c					
UNIBAS LCD8	0	1	2	1	1	1	1	1	1	1	1	1	1	1	1	1	2	1	1	2	1	2	2	1	–	–	–	–	–	–	–	–	–	–	c					
15+20																1	2	2	2	1	2	2	2	1	2	–	–	–	–	–	–	–	–	–	c					
UNIBAS LCD9	0	1	2	1	1	1	1	1	1	1	1	1	1	1	1	1	1	2	1	1	2	1	2	2	–	–	–	–	–	–	–	–	–	–	c					
15+20																1	2	2	1	2	2	1	2	2	2	–	–	–	–	–	–	–	–	–	c					
UNIBAS LCE1	0	1	2	1	1	1	1	1	1	1	1	1	1	1	1	1	1	2	1	2	1	2	2	1	–	–	–	–	–	–	–	–	–	–	c					
15+20																1	1	3	2	1	2	2	2	1	2	–	–	–	–	–	–	–	–	–	c					
UNIBAS LCE2	0	1	2	1	1	1	1	1	1	1	1	1	1	1	1	1	1	2	1	2	1	2	1	2	1	–	–	–	–	–	–	–	–	–	–	c				
15+21																1	1	3	1	2	2	2	1	2	2	1	–	–	–	–	–	–	–	–	–	c				
UNIBAS LCE3	0	1	2	1	1	1	1	1	1	1	1	1	1	1	1	1	1	2	1	1	2	1	2	2	–	–	–	–	–	–	–	–	–	–	c					
15+20																1	1	3	1	2	2	2	1	2	2	–	–	–	–	–	–	–	–	–	c					
UNIBAS LCE4	0	1	2	1	1	1	1	1	1	1	1	1	1	1	1	1	1	2	1	1	2	1	2	2	1	–	–	–	–	–	–	–	–	–	c					
15+20																1	1	2	2	2	2	1	2	2	2	–	–	–	–	–	–	–	–	–	c					
UNIBAS LCE5	0	1	2	1	1	1	1	1	1	1	1	1	1	1	1	1	1	2	1	2	1	2	1	2	–	–	–	–	–	–	–	–	–	–	c					
15+20																1	1	3	2	1	2	2	2	1	2	–	–	–	–	–	–	–	–	–	c					
UNIBAS LCE6	–	1	2	1	1	1	1	1	1	1	1	1	1	1	1	1	1	1	2	1	2	1	2	2	–	–	–	–	–	–	–	–	–	–	c					
15+20																1	1	3	2	1	2	1	2	2	2	–	–	–	–	–	–	–	–	–	c					
Bathybates graueri UNIBAS IUH6	0	1	2	1	1	1	1	1	1	1	1	1	1	1	1	1	1	2	1	2	1	2	2	–	–	–	–	–	–	–	–	–	–	–	c					
15+20																1	2	2	1	2	1	2	2	1	–	–	–	–	–	–	–	–	–	–	c					
UNIBAS IUH8	0	1	2	1	1	1	1	1	1	1	1	1	1	1	1	1	2	1	1	2	2	1	1	–	–	–	–	–	–	–	–	–	–	c						
15+19																1	3	2	1	2	1	2	2	1	–	–	–	–	–	–	–	–	–	c						
UNIBAS IUH9	0	1	2	1	1	1	1	1	1	1	1	1	1	1	1	1	2	1	2	1	2	1	1	–	–	–	–	–	–	–	–	–	–	c						
15+19																1	3	2	1	2	2	1	2	1	–	–	–	–	–	–	–	–	–	c						
UNIBAS IXG2	0	1	2	1	1	1	1	1	1	1	1	1	1	1	1	1	2	1	1	2	2	1	1	–	–	–	–	–	–	–	–	–	–	c						
15+19																1	3	2	1	2	2	1	2	1	–	–	–	–	–	–	–	–	–	c						
UNIBAS IXG3	0	1	2	1	1	1	1	1	1	1	1	1	1	1	1	1	2	1	2	1	2	1	1	–	–	–	–	–	–	–	–	–	–	c						
15+19																1	2	2	2	1	2	2	1	2	–	–	–	–	–	–	–	–	–	c						
Bathybates hornii UNIBAS MOD4	–	1	2	1	1	1	1	1	1	1	1	1	1	1	1	2	1	1	1	1	2	2	2	1	–	–	–	–	–	–	–	–	–	–	c					
15+20																1	3	2	1	2	2	1	2	2	–	–	–	–	–	–	–	–	–	–	c					
Bathybates leo UNIBAS KCB8	–	1	2	1	1	1	1	1	1	1	1	1	1	1	1	1	1	1	1	2	1	2	1	2	2	–	–	–	–	–	–	–	–	–	–	c				
16+20																	2	1	2	1	2	2	1	2	2	1	–	–	–	–	–	–	–	–	–	c				
UNIBAS KCB9	–	1	2	1	1	1	1	1	1	1	1	1	1	1	1	1	1	2	1	1	2	1	2	1	1	–	–	–	–	–	–	–	–	–	–	c				
16+20																	1	3	2	1	2	1	2	2	2	–	–	–	–	–	–	–	–	–	–	c				
UNIBAS KCC1	–	1	2	1	1	1	1	1	1	1	1	1	1	1	1	1	1	2	1	1	1	2	2	1	1	–	–	–	–	–	–	–	–	–	–	c				
16+20																	2	2	2	1	2	1	2	2	1	2	–	–	–	–	–	–	–	–	–	c				
UNIBAS KCC2	–	1	2	1	1	1	1	1	1	1	1	1	1	1	1	1	1	2	1	1	2	1	2	2	–	–	–	–	–	–	–	–	–	–	–	c				
16+20																	1	3	1	2	2	1	2	2	1	2	–	–	–	–	–	–	–	–	–	c				
UNIBAS KCC5	–	1	2	1	1	1	1	1	1	1	1	1	1	1	1	1	1	1	2	1	1	2	1	2	2	–	–	–	–	–	–	–	–	–	–	c				
16+20																	1	3	1	2	2	1	2	2	2	1	–	–	–	–	–	–	–	–	–	c				
Bathybates minor UNIBAS JEF6	–	1	2	1	1	1	1	1	1	1	1	1	1	1	1	1	2	1	2	1	2	2	1	–	–	–	–	–	–	–	–	–	–	–	–	c				
15+21																1	3	1	2	2	1	2	2	2	2	–	–	–	–	–	–	–	–	–	–	c				

Species, Museum, Catalog number / ID<br>Vertebral count	1	2	3	4	5	6	7	8	9	10	11	12	13	14	15	16	17	18	19	20	21	22	23	24	25	26	27	28	29	30	31	32	33	34	35	36	37	38	39	40
UNIBAS JEF7	–	1	2	1	1	1	1	1	1	1	1	1	1	1	1	2	1	2	1	2	1	2	1	–	–	–	–	–	–	–	–	–	–	–	–	c				
15+21																1	2	2	2	2	2	2	2	1	1	–	–	–	–	–	–	–	–	–	–	c				
UNIBAS JEF8	–	1	2	1	1	1	1	1	1	1	1	1	1	1	1	1	2	1	2	1	2	2	1	–	–	–	–	–	–	–	–	–	–	–	–	c				
15+21																1	2	2	2	2	1	2	2	2	2	–	–	–	–	–	–	–	–	–	–	c				
UNIBAS JEF9	–	1	2	1	1	1	1	1	1	1	1	1	1	1	1	2	1	1	2	2	1	2	1	–	–	–	–	–	–	–	–	–	–	–	–	c				
15+21																1	2	2	2	1	2	2	2	2	1	1	–	–	–	–	–	–	–	–	–	c				
UNIBAS JEG2	–	1	2	1	1	1	1	1	1	1	1	1	1	1	1	2	1	1	2	1	2	1	2	1	–	–	–	–	–	–	–	–	–	–	–	–	c			
15+22																1	2	2	2	1	2	2	2	1	2	1	–	–	–	–	–	–	–	–	–	–	c			
Bathybates vittatus UNIBAS LNH9	0?	1	2	1	1	1	1	1	1	1	1	1	1	1	1	1	1	2	1	1	2	2	1	2	–	–	–	–	–	–	–	–	–	–	–	c				
16+20																	1	3	2	1	2	2	1	2	2	–	–	–	–	–	–	–	–	–	–	c				
Hemibates stenosoma UNIBAS IXC5	0	1	2	1	1	1	1	1	1	1	1	1	1	1	1	1	1	1	1	2	1	2	2	1	–	–	–	–	–	–	–	–	–	–	c					
16+19																	1	2	2	1	2	2	2	1	–	–	–	–	–	–	–	–	–	–	c					
UNIBAS IXC6	0	1	2	1	1	1	1	1	1	1	1	1	1	1	1	1	1	1	1	1	2	2	2	2	–	–	–	–	–	–	–	–	–	–	c					
16+19																	1	2	1	2	2	2	2	2	–	–	–	–	–	–	–	–	–	–	c					
UNIBAS IXC7	0	1	2	1	1	1	1	1	1	1	1	1	1	1	1	1	1	1	1	2	1	2	2	1	–	–	–	–	–	–	–	–	–	–	c					
16+19																	1	2	2	1	2	2	2	2	–	–	–	–	–	–	–	–	–	–	c					
UNIBAS IXC8	0	1	2	1	1	1	1	1	1	1	1	1	1	1	1	1	1	1	1	2	1	2	2	1	–	–	–	–	–	–	–	–	–	–	c					
16+19																	1	2	2	1	2	2	2	2	–	–	–	–	–	–	–	–	–	–	c					
Trematocara unimaculatum UNIBAS IXF2	0	1	2	1	1	1	1	1	1	1	1	1	1	1	1	1	1	2	1	2	–	–	–	–	–	–	–	–	–	c										
12+18													1	1	1	1	2	1	2	1	–	–	–	–	–	–	–	–	–	c										
UNIBAS IXF5	0	1	2	1	1	1	1	1	1	1	1	1	1	1	1	1	1	2	1	2	–	–	–	–	–	–	–	–	–	c										
11+19												1	–	1	1	2	1	1	2	1	–	–	–	–	–	–	–	–	–	c										
UNIBAS IXF6	0	1	2	1	1	1	1	1	1	1	1	1	1	1	1	1	1	1	2	1	1	–	–	–	–	–	–	–	–	c										
12+18													1	1	1	1	1	2	2	1	1	–	–	–	–	–	–	–	–	c										
UNIBAS IXF7	0	1	2	1	1	1	1	1	1	1	1	1	1	1	1	1	1	1	2	1	1	–	–	–	–	–	–	–	–	c										
12+18													1	1	1	1	1	2	2	1	1	–	–	–	–	–	–	–	–	c										
UNIBAS IXG1	0	1	2	1	1	1	1	1	1	1	1	1	1	1	1	1	1	2	1	2	–	–	–	–	–	–	–	–	–	c										
11+19												1	–	1	1	2	1	2	1	1	–	–	–	–	–	–	–	–	–	c										
Trematocara zebra UNIBAS LFE7	0	1	2	1	1	1	1	1	1	1	1	1	1	1	1	1	1	2	1	2	–	–	–	–	–	–	–	–	–	c										
12+18													1	1	2	1	2	1	1	–	–	–	–	–	–	–	–	–	–	c										
UNIBAS LFE8	0	1	2	1	1	1	1	1	1	1	1	1	1	1	1	1	1	1	2	–	–	–	–	–	–	–	–	–	–	c										
12+18													1	2	1	2	1	2	–	–	–	–	–	–	–	–	–	–	–	c										
UNIBAS LFE9	0	1	2	1	1	1	1	1	1	1	1	1	1	1	1	1	1	2	1	–	–	–	–	–	–	–	–	–	–	c										
12+18													1	2	1	1	1	2	–	–	–	–	–	–	–	–	–	–	–	c										
UNIBAS LFF2	0	1	2	1	1	1	1	1	1	1	1	1	1	1	1	1	1	2	1	–	–	–	–	–	–	–	–	–	–	c										
12+18													1	2	2	1	2	1	–	–	–	–	–	–	–	–	–	–	–	c										
UNIBAS LFF3	0	1	2	1	1	1	1	1	1	1	1	1	1	1	1	1	1	2	1	1	–	–	–	–	–	–	–	–	–	c										
12+18													1	2	1	2	1	2	–	–	–	–	–	–	–	–	–	–	–	c										
**Benthochromini**																																								
Benthochromis tricoti UNIBAS LDB4	0	2	1	1	1	1	1	1	1	1	1	1	1	1	1	1	1	1	1	1	1	1	1	1	2	1	1	1	–	–	–	–	–	–	–	–	c			
18+19																			1	1	1	1	1	2	1	2	1	–	–	–	–	–	–	–	–	–	c			
UNIBAS LDB5	0	1	1	2	–	1	1	1	1	1	1	1	1	1	1	1	1	1	1	1	1	1	1	1	2	2	1	–	–	–	–	–	–	–	–	–	c			
18+19																			1	1	1	1	2	1	2	1	1	–	–	–	–	–	–	–	–	–	c			
UNIBAS LDB6	0	2	1	1	1	1	1	1	1	1	1	1	1	1	1	1	1	1	1	1	1	1	1	1	2	1	–	–	–	–	–	–	–	–	c					
17+18																		1	1	1	1	1	2	2	1	1	–	–	–	–	–	–	–	–	c					
UNIBAS LDB7	0	2	1	1	1	1	1	1	1	1	1	1	1	1	1	1	1	1	1	1	1	1	1	1	1	2	1	–	–	–	–	–	–	–	–	c				
18+18																			1	1	1	1	1	2	1	2	–	–	–	–	–	–	–	–	–	c				
UNIBAS LDB8	0	2	1	1	1	1	1	1	1	1	1	1	1	1	1	1	1	1	1	1	1	1	1	1	1	2	1	–	–	–	–	–	–	–	–	c				
17+19																		1	1	1	1	1	1	2	2	1	–	–	–	–	–	–	–	–	–	c				
**Boulengerochromini**																																								
Boulengerochromis microlepis AMNH 11727	0	2	1	1	1	1	1	1	1	1	1	1	1	1	1	1	1	2	1	2	2	2	2	2	–	–	–	–	–	–	–	–	–	c						
16+18																	1	1	1	2	1	2	2	1	–	–	–	–	–	–	–	–	–	c						
	0	2	1	1	1	1	1	1	1	1	1	1	1	1	1	1	1	1	2	1	2	2	2	2	1	–	–	–	–	–	–	–	–	–	c					
16+19																	1	–	1	2	1	2	2	2	–	–	–	–	–	–	–	–	–	–	c					
UNIBAS ILI6	0	2	1	1	1	1	1	1	1	1	1	1	1	1	1	1	1	2	1	2	2	2	2	2	–	–	–	–	–	–	–	–	–	c						
16+18																	1	1	1	2	2	2	2	–	–	–	–	–	–	–	–	–	–	c						
UNIBAS ITH6	0	2	1	1	1	1	1	1	1	1	1	1	1	1	1	1	1	2	1	2	2	2	2	1	–	–	–	–	–	–	–	–	–	c						
16+18																	1	1	1	2	2	1	2	–	–	–	–	–	–	–	–	–	–	c						
UNIBAS ITH7	0	2	1	1	1	1	1	1	1	1	1	1	1	1	1	1	1	2	1	2	2	1	2	3	–	–	–	–	–	–	–	–	–	c						
16+18																	1	1	1	2	1	2	3	–	–	–	–	–	–	–	–	–	–	c						
UNIBAS IZB6	0	2	1	1	1	1	1	1	1	1	1	1	1	1	1	1	1	2	1	2	2	2	2	2	–	–	–	–	–	–	–	–	–	c						
16+18																	1	1	1	1	2	2	2	–	–	–	–	–	–	–	–	–	–	c						
UNIBAS JBI8	0	2	1	1	1	1	1	1	1	1	1	1	1	1	1	1	1	2	1	2	1	2	2	3	–	–	–	–	–	–	–	–	–	c						
16+18																	1	1	1	1	2	2	2	–	–	–	–	–	–	–	–	–	–	c						
**Cyphotilapiini**																																								
Cyphotilapia frontosa UNIBAS LEI5	0	2	1	1	1	1	1	1	1	1	1	1	1	1	1	1	1	1	1	1	1	2	2	2	–	–	–	–	–	–	–	c								
16+16																	1	–	1	1	1	2	2	–	–	–	–	–	–	–	–	c								

**Species, Museum, Catalog number / ID** **Vertebral count**	1	2	3	4	5	6	7	8	9	10	11	12	13	14	15	16	17	18	19	20	21	22	23	24	25	26	27	28	29	30	31	32	33	34	35	36	37	38	39	40
UNIBAS LEI6	0	2	1	1	1	1	1	1	1	1	1	1	1	1	1	1	1	1	1	1	2	1	2	1	–	–	–	–	–	–	–	c								
16+16																	2	–	1	1	1	2	2	–	–	–	–	–	–	–	–	c								
UNIBAS LEI7	0	2	1	1	1	1	1	1	1	1	1	1	1	1	1	1	1	1	1	1	2	1	2	2	–	–	–	–	–	–	–	c								
16+16																	2	–	–	1	2	1	2	–	–	–	–	–	–	–	–	c								
UNIBAS LEI8	0	2	1	1	1	1	1	1	1	1	1	1	1	1	1	1	1	1	1	1	1	2	1	2	1	–	–	–	–	–	–	c								
16+16																	1	–	1	1	1	2	2	–	–	–	–	–	–	–	–	c								
UNIBAS LEI9	0	2	2	1	–	1	1	1	1	1	1	1	1	1	1	1	1	1	1	1	1	2	2	1	–	–	–	–	–	–	–	c								
15+17																1	1	–	1	–	2	1	2	–	–	–	–	–	–	–	–	c								
Cyphotilapia gibberosa UNIBAS JEA9	0	2	1	1	1	1	1	1	1	1	1	1	1	1	1	1	1	1	1	1	1	1	2	2	1	–	–	–	–	–	–	c								
17+15																		1	1	1	1	2	2	1	–	–	–	–	–	–	–	c								
UNIBAS JBG1	0	2	1	1	1	1	1	1	1	1	1	1	1	1	1	1	1	1	1	1	1	2	2	1	–	–	–	–	–	–	–	c								
16+16																	2	–	–	1	2	1	2	–	–	–	–	–	–	–	–	c								
UNIBAS JEA8	0	2	1	1	1	1	1	1	1	1	1	1	1	1	1	1	1	1	1	1	1	2	1	2	1	–	–	–	–	–	–	c								
16+16																	2	–	–	1	1	2	2	1	–	–	–	–	–	–	–	c								
UNIBAS JEB1	0	2	1	1	1	1	1	1	1	1	1	1	1	1	1	1	1	1	1	1	1	2	1	2	–	–	–	–	–	–	–	c								
16+16																	2	–	–	1	1	2	2	–	–	–	–	–	–	–	–	c								
UNIBAS JED6	0	2	1	1	1	1	1	1	1	1	1	1	1	1	1	1	1	1	1	1	1	2	1	3	–	–	–	–	–	–	–	c								
16+16																	2	–	–	1	1	2	2	1	–	–	–	–	–	–	–	c								
**Cyprichromini**																																								
Cyprichromis coloratus UNIBAS ITF7	0	1	2	1	1	1	1	1	1	1	1	1	1	1	1	1	1	1	1	1	1	1	1	1	1	1	2	1	–	–	–	–	–	–	–	–	–	–	c	
20+19														1	2	–	1	–	1	1	–	1	1	2	1	2	–	–	–	–	–	–	–	–	–	–	–	–	c	
UNIBAS JEC7	0	1	2	1	1	1	1	1	1	1	1	1	1	1	1	1	1	1	1	1	1	1	1	1	1	2	1	1	–	–	–	–	–	–	–	–	–	–	c	
21+18															2	1	1	1	–	1	1	–	1	2	1	2	–	–	–	–	–	–	–	–	–	–	–	–	c	
UNIBAS JEC8	0	1	2	1	1	1	1	1	1	1	1	1	1	1	1	1	1	1	1	1	1	1	1	1	2	1	2	–	–	–	–	–	–	–	–	–	–	–	c	
20+19														2	1	1	–	1	1	–	1	1	1	1	2	1	–	–	–	–	–	–	–	–	–	–	–	–	c	
UNIBAS JEC9	0	1	2	1	1	1	1	1	1	1	1	1	1	1	1	1	1	1	1	1	1	1	1	1	2	1	1	–	–	–	–	–	–	–	–	–	–	c		
20+18															2	1	1	–	1	1	–	1	2	1	2	–	–	–	–	–	–	–	–	–	–	–	–	c		
UNIBAS JED1	0	1	2	1	1	1	1	1	1	1	1	1	1	1	1	1	1	1	1	1	1	1	1	1	2	1	1	–	–	–	–	–	–	–	–	–	–	c		
20+18														2	1	1	–	1	–	1	–	2	1	1	2	–	–	–	–	–	–	–	–	–	–	–	–	c		
Cyprichromis leptosoma UNIBAS ITC7	0	1	2	1	1	1	1	1	1	1	1	1	1	1	1	1	1	1	1	1	1	1	1	2	1	1	–	–	–	–	–	–	–	–	–	–	c			
18+19														2	1	1	1	–	1	–	1	2	1	2	1	–	–	–	–	–	–	–	–	–	–	–	c			
UNIBAS ITE6	0	1	2	1	1	1	1	1	1	1	1	1	1	1	1	1	1	1	1	1	1	2	1	1	2	–	–	–	–	–	–	–	–	–	–	c				
17+19														2	1	1	–	1	–	1	2	1	2	1	–	–	–	–	–	–	–	–	–	–	–	c				
UNIBAS ITE8	0	1	2	1	1	1	1	1	1	1	1	1	1	1	1	1	1	1	1	1	1	1	2	1	2	–	–	–	–	–	–	–	–	–	–	c				
18+18														2	1	1	1	–	1	–	1	2	1	2	–	–	–	–	–	–	–	–	–	–	–	c				
UNIBAS ITE9	0	1	2	1	1	1	1	1	1	1	1	1	1	1	1	1	1	1	1	1	1	1	2	1	1	1	–	–	–	–	–	–	–	–	–	–	c			
19+18														2	1	1	1	1	1	–	–	2	1	2	1	–	–	–	–	–	–	–	–	–	–	–	c			
UNIBAS ITF1	0	1	2	1	1	1	1	1	1	1	1	1	1	1	1	1	1	1	1	1	1	1	2	1	1	1	–	–	–	–	–	–	–	–	–	–	c			
18+19														2	1	1	1	–	1	1	–	2	1	2	–	–	–	–	–	–	–	–	–	–	–	–	c			
Cyprichromis microlepidotus UNIBAS JVE9	0	1	2	1	1	1	1	1	1	1	1	1	1	1	1	1	1	1	1	1	1	1	1	2	1	2	–	–	–	–	–	–	–	–	–	–	c			
19+18													1	1	1	1	–	1	1	–	1	2	1	2	1	–	–	–	–	–	–	–	–	–	–	–	c			
UNIBAS JVF1	0	1	2	1	1	1	1	1	1	1	1	1	1	1	1	1	1	1	1	1	1	1	1	2	1	2	–	–	–	–	–	–	–	–	–	–	c			
19+18														2	1	1	1	1	–	1	–	2	1	2	1	1	–	–	–	–	–	–	–	–	–	–	c			
UNIBAS JVF5	0	1	2	1	1	1	1	1	1	1	1	1	1	1	1	1	1	1	1	1	1	1	1	2	1	2	–	–	–	–	–	–	–	–	–	c				
18+18														2	1	1	1	1	1	–	1	1	1	2	1	–	–	–	–	–	–	–	–	–	–	c				
UNIBAS KDG3	0	1	1	2	1	1	1	1	1	1	1	1	1	1	1	1	1	1	1	1	1	1	1	2	2	–	–	–	–	–	–	–	–	–	–	c				
18+18														2	1	1	1	–	1	–	1	2	1	2	–	–	–	–	–	–	–	–	–	–	–	c				
UNIBAS KDG4	0	1	2	1	1	1	1	1	1	1	1	1	1	1	1	1	1	1	1	1	1	1	2	1	1	2	–	–	–	–	–	–	–	–	–	c				
19+17														2	1	1	–	1	1	–	1	2	1	2	1	–	–	–	–	–	–	–	–	–	–	c				
Cyprichromis pavo UNIBAS JEC1	0	1	2	1	1	1	1	1	1	1	1	1	1	1	1	1	1	1	1	1	1	1	1	1	1	1	2	1	–	–	–	–	–	–	–	–	–	–	c	
21+18															2	1	–	1	1	–	1	–	1	2	1	2	1	–	–	–	–	–	–	–	–	–	–	–	c	
UNIBAS JEC2	0	1	2	1	1	1	1	1	1	1	1	1	1	1	1	1	1	1	1	1	1	1	1	1	2	1	1	1	–	–	–	–	–	–	–	–	–	–	c	
20+19														1	2	1	–	1	1	1	–	–	1	2	2	1	–	–	–	–	–	–	–	–	–	–	–	–	c	
UNIBAS JEC4	0	1	2	1	1	1	1	1	1	1	1	1	1	1	1	1	1	1	1	1	1	1	1	1	2	1	1	1	–	–	–	–	–	–	–	–	–	–	c	
21+18														1	2	1	–	1	1	1	–	–	1	2	1	2	1	–	–	–	–	–	–	–	–	–	–	–	c	
UNIBAS JEC5	0	–	3	1	1	1	1	1	1	1	1	1	1	1	1	1	1	1	1	1	1	1	1	1	1	2	1	1	1	–	–	–	–	–	–	–	–	–	–	c
21+19															1	2	–	1	1	1	–	1	–	2	1	2	1	–	–	–	–	–	–	–	–	–	–	–	–	c
UNIBAS JEC6	0	–	2	2	1	1	1	1	1	1	1	1	1	1	1	1	1	1	1	1	1	1	1	1	1	2	1	2	–	–	–	–	–	–	–	–	–	–	c	
20+19															2	1	1	1	1	1	–	–	1	2	1	2	1	–	–	–	–	–	–	–	–	–	–	–	c	
Cyprichromis zonatus UNIBAS LBI8	0	1	2	1	1	1	1	1	1	1	1	1	1	1	1	1	1	1	1	1	1	1	1	1	2	1	1	–	–	–	–	–	–	–	–	–	–	c		
19+19															1	2	1	–	1	1	–	1	1	2	2	1	–	–	–	–	–	–	–	–	–	–	–	c		
UNIBAS LBI9	0	1	2	1	1	1	1	1	1	1	1	1	1	1	1	1	1	1	1	1	1	1	1	1	2	1	2	–	–	–	–	–	–	–	–	–	–	c		
19+19															2	1	1	1	–	1	–	1	1	2	2	1	–	–	–	–	–	–	–	–	–	–	–	c		
UNIBAS LDA1	0	1	2	1	1	1	1	1	1	1	1	1	1	1	1	1	1	1	1	1	1	1	1	1	2	1	2	–	–	–	–	–	–	–	–	–	–	c		
19+19														1	2	1	1	1	–	1	–	1	2	1	3	–	–	–	–	–	–	–	–	–	–	–	–	c		
UNIBAS LDA2	0	2	1	1	1	1	1	1	1	1	1	1	1	1	1	1	1	1	1	1	1	1	1	2	1	1	–	–	–	–	–	–	–	–	–	–	c			
19+18														1	1	1	1	–	1	–	1	1	2	3	–	–	–	–	–	–	–	–	–	–	–	–	c			

Species, Museum, Catalog number / ID<br>Vertebral count	1	2	3	4	5	6	7	8	9	10	11	12	13	14	15	16	17	18	19	20	21	22	23	24	25	26	27	28	29	30	31	32	33	34	35	36	37	38	39	40
UNIBAS LDA3	0	1	2	1	1	1	1	1	1	1	1	1	1	1	1	1	1	1	1	1	1	1	1	2	1	2	–	–	–	–	–	–	–	–	–	–	c			
19+18															2	1	1	1	–	1	–	1	2	1	2	–	–	–	–	–	–	–	–	–	–	–	c			
Cyprichromis sp. "dwarf jumbo" UNIBAS KFA5	0	1	2	1	1	1	1	1	1	1	1	1	1	1	1	1	1	1	1	1	1	1	1	2	1	1	2	–	–	–	–	–	–	–	–	–	–	c		
20+18														2	1	1	1	1	1	–	–	2	1	1	2	1	–	–	–	–	–	–	–	–	–	–	–	c		
UNIBAS KFB2	0	1	2	1	1	1	1	1	1	1	1	1	1	1	1	1	1	1	1	1	1	1	1	2	1	1	1	–	–	–	–	–	–	–	–	–	c			
18+19													1	2	1	–	1	–	1	–	1	1	2	1	2	–	–	–	–	–	–	–	–	–	–	–	c			
UNIBAS KFB3	0	1	2	1	1	1	1	1	1	1	1	1	1	1	1	1	1	1	1	1	1	1	1	2	1	1	1	–	–	–	–	–	–	–	–	–	c			
18+19														2	1	1	1	–	1	–	1	1	2	1	1	–	–	–	–	–	–	–	–	–	–	–	c			
UNIBAS KFB4	0	1	2	1	1	1	1	1	1	1	1	1	1	1	1	1	1	1	1	1	1	1	1	1	2	1	1	–	–	–	–	–	–	–	–	–	c			
20+17														2	1	1	1	–	1	1	–	1	2	1	1	–	–	–	–	–	–	–	–	–	–	–	c			
UNIBAS KFB5	0	1	2	1	1	1	1	1	1	1	1	1	1	1	1	1	1	1	1	1	1	1	1	2	1	2	–	–	–	–	–	–	–	–	–	–	–	c		
20+18														1	2	1	1	1	–	1	–	1	2	1	2	–	–	–	–	–	–	–	–	–	–	–	–	c		
Cyprichromis sp. "jumbo" UNIBAS INH5	0	1	2	1	1	1	1	1	1	1	1	1	1	1	1	1	1	1	1	1	1	1	1	1	2	1	1	–	–	–	–	–	–	–	–	–	–	c		
20+18														2	1	1	–	1	1	1	–	1	2	1	2	–	–	–	–	–	–	–	–	–	–	–	–	c		
UNIBAS MOD7	0	1	2	1	1	1	1	1	1	1	1	1	1	1	1	1	1	1	1	1	1	1	1	1	2	1	1	1	–	–	–	–	–	–	–	–	–	–	c	
20+19															2	1	1	1	1	1	–	–	2	1	1	2	–	–	–	–	–	–	–	–	–	–	–	–	c	
UNIBAS MOD8	0	1	2	1	1	1	1	1	1	1	1	1	1	1	1	1	1	1	1	1	1	1	1	2	1	1	1	–	–	–	–	–	–	–	–	–	–	c		
19+19															2	1	1	1	–	–	2	1	1	2	1	–	–	–	–	–	–	–	–	–	–	–	–	c		
UNIBAS MOD9	0	1	2	1	1	1	1	1	1	1	1	1	1	1	1	1	1	1	1	1	1	1	1	1	1	2	1	1	–	–	–	–	–	–	–	–	–	–	c	
20+19															2	1	1	1	1	–	–	1	2	1	1	1	–	–	–	–	–	–	–	–	–	–	–	–	c	
UNIBAS MOE1	0	1	2	1	1	1	1	1	1	1	1	1	1	1	1	1	1	1	1	1	1	1	1	1	2	1	1	1	–	–	–	–	–	–	–	–	–	–	c	
20+19														1	1	1	1	1	–	1	–	2	1	1	1	1	–	–	–	–	–	–	–	–	–	–	–	–	c	
Paracyprichromis brieni UNIBAS JXI7	0	2	1	1	1	1	1	1	1	1	1	1	1	1	1	1	1	1	1	1	1	1	2	1	2	–	–	–	–	–	–	–	–	–	–	c				
17+19																		3	1	1	1	2	1	2	–	–	–	–	–	–	–	–	–	–	–	c				
UNIBAS JXI8	0	2	1	1	1	1	1	1	1	1	1	1	1	1	1	1	1	1	1	1	1	1	2	1	2	–	–	–	–	–	–	–	–	–	–	c				
16+20																	3	–	1	1	1	2	1	1	–	–	–	–	–	–	–	–	–	–	–	c				
UNIBAS JYA1	0	2	1	1	1	1	1	1	1	1	1	1	1	1	1	1	1	1	1	1	1	1	2	1	2	–	–	–	–	–	–	–	–	–	–	c				
17+19																		3	1	1	1	2	2	–	–	–	–	–	–	–	–	–	–	–	–	c				
UNIBAS JYA2	0	2	1	1	1	1	1	1	1	1	1	1	1	1	1	1	1	1	1	1	1	1	2	1	1	–	–	–	–	–	–	–	–	–	–	c				
16+20																	–	3	1	2	1	2	2	–	–	–	–	–	–	–	–	–	–	–	–	c				
UNIBAS JYA3	0	2	1	1	1	1	1	1	1	1	1	1	1	1	1	1	1	1	1	1	1	1	2	1	2	1	–	–	–	–	–	–	–	–	–	c				
16+20																	–	3	1	1	1	2	1	2	–	–	–	–	–	–	–	–	–	–	–	c				
Paracyprichromis nigripinnis UNIBAS GPD8	0	2	1	1	1	1	1	1	1	1	1	1	1	1	1	1	1	1	1	1	1	1	2	1	1	–	–	–	–	–	–	–	–	–	c					
16+19																	3	1	–	1	1	2	1	–	–	–	–	–	–	–	–	–	–	–	c					
UNIBAS GPD9	0	2	1	1	1	1	1	1	1	1	1	1	1	1	1	1	1	1	1	1	1	2	1	2	–	–	–	–	–	–	–	–	–	–	c					
16+19																	3	–	1	1	1	2	1	–	–	–	–	–	–	–	–	–	–	–	c					
UNIBAS GPE3	0	2	1	1	1	1	1	1	1	1	1	1	1	1	1	1	1	1	1	1	1	1	2	1	2	–	–	–	–	–	–	–	–	–	c					
17+18																		3	–	1	1	1	1	1	–	–	–	–	–	–	–	–	–	–	c					
UNIBAS GPE5	0	2	1	1	1	1	1	1	1	1	1	1	1	1	1	1	1	1	1	1	1	2	1	1	1	–	–	–	–	–	–	–	–	–	–	c				
17+19																		3	–	1	1	2	1	–	–	–	–	–	–	–	–	–	–	–	–	c				
Paracyprichromis sp. "brieni south" UNIBAS IQE6	0	2	1	1	1	1	1	1	1	1	1	1	1	1	1	1	1	1	1	1	1	1	1	2	2	–	–	–	–	–	–	–	–	–	–	c				
17+19																		2	2	1	1	1	2	1	–	–	–	–	–	–	–	–	–	–	–	c				
UNIBAS ITD8	0	2	1	1	1	1	1	1	1	1	1	1	1	1	1	1	1	1	1	1	1	1	2	1	2	–	–	–	–	–	–	–	–	–	–	c				
17+19																		3	1	1	1	1	2	1	–	–	–	–	–	–	–	–	–	–	–	c				
UNIBAS ITD9	0	1	2	1	1	1	1	1	1	1	1	1	1	1	1	1	1	1	1	1	1	2	1	2	–	–	–	–	–	–	–	–	–	–	c					
17+18																		3	1	1	2	1	2	–	–	–	–	–	–	–	–	–	–	–	c					
UNIBAS ITE1	0	2	1	1	1	1	1	1	1	1	1	1	1	1	1	1	1	1	1	1	1	2	1	1	2	–	–	–	–	–	–	–	–	–	–	c				
17+19																		2	1	2	1	1	2	1	–	–	–	–	–	–	–	–	–	–	–	c				
UNIBAS ITE2	0	2	1	1	1	1	1	1	1	1	1	1	1	1	1	1	1	1	1	1	1	1	2	1	2	–	–	–	–	–	–	–	–	–	c					
17+18																		2	1	2	1	2	1	1	–	–	–	–	–	–	–	–	–	–	c					
**Ectodini**																																								
Asprotilapia leptura UNIBAS INF8	0	1	2	1	1	1	1	1	1	1	1	1	1	1	1	1	1	1	1	1	1	1	1	1	1	2	1	–	–	–	–	–	–	–	–	–	c			
16+21																	2	1	1	2	1	1	1	–	–	–	–	–	–	–	–	–	–	–	–	–	c			
UNIBAS ING1	0	1	2	1	1	1	1	1	1	1	1	1	1	1	1	1	1	1	1	1	1	1	1	1	1	2	1	–	–	–	–	–	–	–	–	–	c			
15+22																1	1	1	1	2	1	1	1	–	–	–	–	–	–	–	–	–	–	–	–	–	c			
UNIBAS ING2	0	2	1	1	1	1	1	1	1	1	1	1	1	1	1	1	1	1	1	1	1	1	1	1	1	2	–	–	–	–	–	–	–	–	–	–	c			
15+22																2	–	2	–	2	1	1	1	–	–	–	–	–	–	–	–	–	–	–	–	–	c			
UNIBAS ING3	0	2	1	1	1	1	1	1	1	1	1	1	1	1	1	1	1	1	1	1	1	1	1	1	1	1	2	–	–	–	–	–	–	–	–	–	c			
16+21																	3	1	1	1	1	1	2	–	–	–	–	–	–	–	–	–	–	–	–	–	c			
UNIBAS JDC9	0	2	1	1	1	1	1	1	1	1	1	1	1	1	1	1	1	1	1	1	1	1	1	1	1	2	1	–	–	–	–	–	–	–	–	c				
16+20																	2	1	1	1	1	1	2	–	–	–	–	–	–	–	–	–	–	–	–	c				
Aulonocranus dewindti UNIBAS IPH5	0	2	1	1	1	1	1	1	1	1	1	1	1	1	1	1	1	1	1	1	1	2	1	–	–	–	–	–	–	–	–	c								
14+18															1	1	1	2	1	2	1	–	–	–	–	–	–	–	–	–	–	c								
UNIBAS IYH5	0	2	1	1	1	1	1	1	1	1	1	1	1	1	1	1	1	1	1	1	1	2	1	–	–	–	–	–	–	–	–	c								
15+17																2	1	1	1	2	1	2	–	–	–	–	–	–	–	–	–	c								
UNIBAS IYI1	0	2	1	1	1	1	1	1	1	1	1	1	1	1	1	1	1	1	1	1	1	2	1	–	–	–	–	–	–	–	–	c								
14+18															1	–	2	1	1	2	1	2	–	–	–	–	–	–	–	–	–	c								

Species, Museum, Catalog number / ID<br>Vertebral count	1	2	3	4	5	6	7	8	9	10	11	12	13	14	15	16	17	18	19	20	21	22	23	24	25	26	27	28	29	30	31	32	33	34	35	36	37	38	39	40
UNIBAS IYI2	0	2	1	1	1	1	1	1	1	1	1	1	1	1	1	1	1	1	1	1	1	2	1	1?	–	–	–	–	–	–	–	c								
15+17																1	2	1	1	1	2	2	–	–	–	–	–	–	–	–	–	c								
UNIBAS IYI6	0	2	1	1	1	1	1	1	1	1	1	1	1	1	1	1	1	1	1	1	1	1	2	–?	–	–	–	–	–	–	–	c								
15+17																1	2	1	1	2	2	1	–?	–	–	–	–	–	–	–	–	c								
Callochromis macrops UNIBAS JCH5	0	2	1	1	1	1	1	1	1	1	1	1	1	1	1	1	1	1	1	1	1	1	1	2	1	–	–	–	–	–	–	–	c							
14+19															1	–	1	1	1	1	2	1	–	–	–	–	–	–	–	–	–	–	c							
UNIBAS JCI1	0	2	1	1	1	1	1	1	1	1	1	1	1	1	1	1	1	1	1	1	1	1	1	2	1	–	–	–	–	–	–	–	c							
14+19															1	–	1	1	1	1	2	2	–	–	–	–	–	–	–	–	–	–	c							
UNIBAS JCI4	0	2	1	1	1	1	1	1	1	1	1	1	1	1	1	1	1	1	1	1	1	1	1	1	2	–	–	–	–	–	–	–	c							
14+19															1	–	1	1	1	1	2	1	–	–	–	–	–	–	–	–	–	–	c							
UNIBAS JCI5	0	2	1	1	1	1	1	1	1	1	1	1	1	1	1	1	1	1	1	1	1	1	1	2	1	–	–	–	–	–	–	–	c							
14+19															1	–	1	1	1	1	1	2	–	–	–	–	–	–	–	–	–	–	c							
UNIBAS JCI6	0	2	1	1	1	1	1	1	1	1	1	1	1	1	1	1	1	1	1	1	1	1	1	2	1	–	–	–	–	–	–	c								
14+18															1	–	1	1	1	1	2	1	–	–	–	–	–	–	–	–	–	c								
Cardiopharynx schoutedeni UNIBAS KCA7	0	2	1	1	1	1	1	1	1	1	1	1	1	1	1	1	1	1	1	1	1	1	1	1	2	–	–	–	–	–	–	–	–	c						
16+18																	1	1	1	1	2	1	1	2	–	–	–	–	–	–	–	–	–	c						
UNIBAS KCA8	0	2	1	1	1	1	1	1	1	1	1	1	1	1	1	1	1	1	1	1	1	1	1	1	2	1	–	–	–	–	–	–	–	–	c					
16+19																	1	1	1	2	1	1	2	1	–	–	–	–	–	–	–	–	–	–	c					
UNIBAS KCB1	0	2	1	1	1	1	1	1	1	1	1	1	1	1	1	1	1	1	1	1	1	1	1	2	1	–	–	–	–	–	–	–	–	c						
16+18																	1	1	1	2	1	1	2	1	–	–	–	–	–	–	–	–	–	c						
UNIBAS KCB2	0	2	1	1	1	1	1	1	1	1	1	1	1	1	1	1	1	1	1	1	1	1	1	1	2	1	–	–	–	–	–	–	–	–	c					
17+18																		1	2	1	1	2	1	1	2	–	–	–	–	–	–	–	–	–	c					
UNIBAS KCB4	0	2	1	1	1	1	1	1	1	1	1	1	1	1	1	1	1	1	1	1	1	1	1	1	2	1	–	–	–	–	–	–	–	–	c					
16+19																	2	–	1	1	2	1	1	2	–	–	–	–	–	–	–	–	–	–	c					
Cunningtonia longiventralis UNIBAS IWE5	0	2	1	1	1	1	1	1	1	1	1	1	1	1	1	1	1	1	1	1	1	1	1	1	1	2	–	–	–	–	–	–	–	–	c					
17+18																		2	1	1	1	1	2	1	1	–	–	–	–	–	–	–	–	–	c					
UNIBAS IWE6	0	2	1	1	1	1	1	1	1	1	1	1	1	1	1	1	1	1	1	1	1	1	1	1	2	–	–	–	–	–	–	–	–	–	c					
16+19																	2	–	1	1	1	2	1	2	–	–	–	–	–	–	–	–	–	–	c					
UNIBAS IWE7	0	2	1	1	1	1	1	1	1	1	1	1	1	1	1	1	1	1	1	1	1	1	1	2	1	–	–	–	–	–	–	–	–	–	c					
16+19																	1	1	1	1	1	2	1	1	–	–	–	–	–	–	–	–	–	–	c					
UNIBAS IWE8	0	2	1	1	1	1	1	1	1	1	1	1	1	1	1	1	1	1	1	1	1	1	1	1	2	–	–	–	–	–	–	–	–	–	c					
16+19																	2	–	1	1	2	1	2	1	–	–	–	–	–	–	–	–	–	–	c					
UNIBAS IWE9	0	2	1	1	1	1	1	1	1	1	1	1	1	1	1	1	1	1	1	1	1	1	1	1	2	1	–	–	–	–	–	–	–	–	–	c				
16+20																	2	–	1	1	1	1	2	1	1	–	–	–	–	–	–	–	–	–	–	c				
Cyathopharynx furcifer UNIBAS IQI1	0	2	1	1	1	1	1	1	1	1	1	1	1	1	1	1	1	1	1	1	1	1	1	2	1	–	–	–	–	–	–	–	–	c						
16+18																	1	1	1	1	1	2	1	2	–	–	–	–	–	–	–	–	–	c						
UNIBAS ISF4	0	2	1	1	1	1	1	1	1	1	1	1	1	1	1	1	1	1	1	1	1	1	1	2	2	–	–	–	–	–	–	–	–	c						
16+18																	1	1	1	1	2	1	2	1	–	–	–	–	–	–	–	–	–	c						
UNIBAS ISF5	0	2	1	1	1	1	1	1	1	1	1	1	1	1	1	1	1	1	1	1	1	1	2	1	1	–	–	–	–	–	–	–	–	c						
16+18																	1	1	1	1	2	1	2	1	–	–	–	–	–	–	–	–	–	c						
UNIBAS IUH1	0	2	1	1	1	1	1	1	1	1	1	1	1	1	1	1	1	1	1	1	1	1	1	2	1	–	–	–	–	–	–	–	c							
16+17																	1	1	2	1	1	1	2	1	–	–	–	–	–	–	–	–	c							
UNIBAS IUH2	0	2	1	1	1	1	1	1	1	1	1	1	1	1	1	1	1	1	1	1	1	1	2	1	–	–	–	–	–	–	–	–	c							
16+17																	1	2	1	1	1	2	1	2	–	–	–	–	–	–	–	–	c							
Ectodus descampsii UNIBAS IRE9	0	2	1	1	1	1	1	1	1	1	1	1	1	1	1	1	1	1	1	1	1	1	2	1	–	–	–	–	–	–	–	c								
15+17																1	1	1	1	1	2	1	2	–	–	–	–	–	–	–	–	c								
UNIBAS IRF1	0	2	1	1	1	1	1	1	1	1	1	1	1	1	1	1	1	1	1	1	1	1	2	1	–	–	–	–	–	–	–	–	c							
15+18																2	–	1	2	1	1	2	1	–	–	–	–	–	–	–	–	–	c							
UNIBAS IRF2	0	2	1	1	1	1	1	1	1	1	1	1	1	1	1	1	1	1	1	1	1	1	2	1	1	–	–	–	–	–	–	–	c							
16+17																	2	1	1	1	1	2	2	–	–	–	–	–	–	–	–	–	c							
UNIBAS IRF3	0	2	1	1	1	1	1	1	1	1	1	1	1	1	1	1	1	1	1	1	1	1	2	1	–	–	–	–	–	–	–	–	c							
15+18																2	–	1	1	1	2	1	1	–	–	–	–	–	–	–	–	–	c							
UNIBAS IRF5	0	2	1	1	1	1	1	1	1	1	1	1	1	1	1	1	1	1	1	1	1	2	1	1	–	–	–	–	–	–	–	c								
15+17																2	1	1	1	1	2	1	–	–	–	–	–	–	–	–	–	c								
Enantiopus melanogenys UNIBAS IVB2	0	2	1	1	1	1	1	1	1	1	1	1	1	1	1	1	1	1	1	1	1	1	1	1	1	1	1	1	2	–	–	–	–	–	–	–	–	–	c	
14+25															3	–	1	1	1	1	1	1	1	2	1	1	1	1	2	–	–	–	–	–	–	–	–	–	c	
UNIBAS JCB9	0	2	1	1	1	1	1	1	1	1	1	1	1	1	1	1	1	1	1	1	1	1	1	1	1	1	1	1	2	–	–	–	–	–	–	–	–	–	c	
14+25															3	–	1	1	1	1	1	1	1	2	1	1	1	2	1	–	–	–	–	–	–	–	–	–	c	
UNIBAS JCC1	0	2	1	1	1	1	1	1	1	1	1	1	1	1	1	1	1	1	1	1	1	1	1	1	1	1	1	1	2	–	–	–	–	–	–	–	–	–	c	
14+25															3	–	1	1	1	1	1	2	1	1	1	1	2	1	1	–	–	–	–	–	–	–	–	–	c	
UNIBAS JCC2	0	2	1	1	1	1	1	1	1	1	1	1	1	1	1	1	1	1	1	1	1	1	1	1	1	1	1	2	1	–	–	–	–	–	–	–	–	c		
14+24															3	–	–	1	1	1	1	2	1	1	1	1	1	2	1	–	–	–	–	–	–	–	–	c		
UNIBAS JCC3	0	2	1	1	1	1	1	1	1	1	1	1	1	1	1	1	1	1	1	1	1	1	1	1	1	1	1	1	2	1	–	–	–	–	–	–	–	c		
14+24															3	–	1	1	1	1	1	1	1	2	1	1	1	2	1	–	–	–	–	–	–	–	–	c		
Grammatotria lemairii UNIBAS IOD3	0	2	1	1	1	1	1	1	1	1	1	1	1	1	1	1	1	1	1	1	1	1	1	1	1	2	–	–	–	–	–	–	–	–	–	–	c			
14+23															2	–	1	1	1	1	1	1	1	1	2	–	–	–	–	–	–	–	–	–	–	–	c			
UNIBAS IUG9	0	2	1	1	1	1	1	1	1	1	1	1	1	1	1	1	1	1	1	1	1	1	1	1	1	2	–	–	–	–	–	–	–	–	–	–	c			
14+23															2	–	1	1	1	1	1	1	1	2	–	–	–	–	–	–	–	–	–	–	–	–	c			

Species, Museum, Catalog number / ID<br>Vertebral count	1	2	3	4	5	6	7	8	9	10	11	12	13	14	15	16	17	18	19	20	21	22	23	24	25	26	27	28	29	30	31	32	33	34	35	36	37	38	39	40
UNIBAS IWG6	0	2	1	1	1	1	1	1	1	1	1	1	1	1	1	1	1	1	1	1	1	1	1	1	1	1	2	–	–	–	–	–	–	–	–	–	c			
15+22																3	–	1	1	1	1	1	1	1	2	–	–	–	–	–	–	–	–	–	–	–	c			
UNIBAS JBC5	0	2	1	1	1	1	1	1	1	1	1	1	1	1	1	1	1	1	1	1	1	1	1	1	1	1	1	–	–	–	–	–	–	–	–	–	c			
14+23															2	–	1	1	1	1	1	1	1	1	2	–	–	–	–	–	–	–	–	–	–	–	c			
UNIBAS JBC6	0	2	1	1	1	1	1	1	1	1	1	1	1	1	1	1	1	1	1	1	1	1	1	1	1	1	2	–	–	–	–	–	–	–	–	–	c			
14+23															2	–	1	1	1	1	1	1	1	1	2	–	–	–	–	–	–	–	–	–	–	–	c			
Lestradea perspicax UNIBAS IOI1	0	2	1	1	1	1	1	1	1	1	1	1	1	1	1	1	1	1	1	1	1	1	1	1	1	2	1	–	–	–	–	–	–	–	–	c				
17+19																		2	1	1	1	1	1	2	2	–	–	–	–	–	–	–	–	–	–	c				
UNIBAS IOI3	0	2	1	1	1	1	1	1	1	1	1	1	1	1	1	1	1	1	1	1	1	1	1	1	2	1	–	–	–	–	–	–	–	–	–	c				
16+20																	2	–	2	1	1	1	1	2	1	–	–	–	–	–	–	–	–	–	–	c				
UNIBAS IOI5	0	2	1	1	1	1	1	1	1	1	1	1	1	1	1	1	1	1	1	1	1	1	1	1	1	2	1	–	–	–	–	–	–	–	–	c				
17+19																		2	1	2	1	1	1	2	1	–	–	–	–	–	–	–	–	–	–	c				
UNIBAS IOI6	0	2	1	1	1	1	1	1	1	1	1	1	1	1	1	1	1	1	1	1	1	1	1	1	1	2	1	–	–	–	–	–	–	–	–	c				
16+20																	2	–	2	1	1	1	2	2	–	–	–	–	–	–	–	–	–	–	–	c				
UNIBAS IOI7	0	2	1	1	1	1	1	1	1	1	1	1	1	1	1	1	1	1	1	1	1	1	1	1	2	2	–	–	–	–	–	–	–	–	–	c				
17+19																		3	–	1	1	1	2	1	2	–	–	–	–	–	–	–	–	–	–	c				
UNIBAS IOI8	0	2	1	1	1	1	1	1	1	1	1	1	1	1	1	1	1	1	1	1	1	1	1	1	2	1	–	–	–	–	–	–	–	–	c					
16+19																	2	–	1	1	2	1	1	2	–	–	–	–	–	–	–	–	–	–	c					
Microdontochromis tenuidentatus UNIBAS LHH4	0	2	1	1	1	1	1	1	1	1	1	1	1	1	1	1	1	1	1	1	1	1	1	1	2	–	–	–	–	–	–	–	–	–	–	c				
14+22															2	1	1	1	1	1	1	1	2	1	–	–	–	–	–	–	–	–	–	–	–	c				
UNIBAS LHH5	0	2	1	1	1	1	1	1	1	1	1	1	1	1	1	1	1	1	1	1	1	1	1	1	1	1	–	–	–	–	–	–	–	–	c					
14+21															1	1	1	1	1	1	1	1	1	2	–	–	–	–	–	–	–	–	–	–	c					
UNIBAS LHH6	0	1	2	1	1	1	1	1	1	1	1	1	1	1	1	1	1	1	1	1	1	1	1	1	2	–	–	–	–	–	–	–	–	–	c					
14+21															1	1	1	1	1	1	1	1	2	–	–	–	–	–	–	–	–	–	–	–	c					
UNIBAS LHH7	0	2	1	1	1	1	1	1	1	1	1	1	1	1	1	1	1	1	1	1	1	1	1	1	2	–	–	–	–	–	–	–	–	–	c					
14+21															1	2	1	1	1	1	1	1	1	–	–	–	–	–	–	–	–	–	–	–	c					
UNIBAS LHH8	0	2	1	1	1	1	1	1	1	1	1	1	1	1	1	1	1	1	1	1	1	1	–	1	2	–	–	–	–	–	–	–	–	–	c					
14+21															1	1	1	1	1	1	1	1	1	–	–	–	–	–	–	–	–	–	–	–	c					
Ophthalmotilapia boops UNIBAS LFI5	0	2	1	1	1	1	1	1	1	1	1	1	1	1	1	1	1	1	1	1	1	1	1	1	1	2	–	–	–	–	–	–	–	–	–	c				
17+19																		3	–	1	1	2	1	2	1	–	–	–	–	–	–	–	–	–	–	c				
UNIBAS LFI8	0	2	1	1	1	2	–	2	1	1	–	1	1	1	1	1	1	1	1	1	1	1	1	2	2	–	–	–	–	–	–	–	–	–	c					
16+19																	2	–	2	1	1	1	1	3	–	–	–	–	–	–	–	–	–	–	c					
UNIBAS LFI9	0	2	1	1	1	1	1	1	1	1	1	1	1	1	1	1	1	1	1	1	1	1	1	2	1	–	–	–	–	–	–	–	–	–	c					
16+19																	2	1	1	1	1	2	2	1	–	–	–	–	–	–	–	–	–	–	c					
UNIBAS LGA2	0	2	1	1	1	1	1	1	1	1	1	1	1	1	1	1	1	1	1	1	1	1	1	1	2	1	–	–	–	–	–	–	–	–	c					
16+19																	1	1	1	1	1	2	1	3	–	–	–	–	–	–	–	–	–	–	c					
UNIBAS LGA3	0	2	1	1	1	1	1	1	1	1	1	1	1	1	1	1	1	1	1	1	1	1	1	2	1	1	–	–	–	–	–	–	–	–	c					
16+19																	3	–	1	1	1	1	2	2	–	–	–	–	–	–	–	–	–	–	c					
Xenotilapia sima UNIBAS LBE2	0	2	1	1	1	1	1	1	1	1	1	1	1	1	1	1	1	1	1	1	1	1	1	1	2	1	–	–	–	–	–	–	–	–	c					
13+22														1	1	1	1	1	1	1	1	1	1	2	–	–	–	–	–	–	–	–	–	–	c					
UNIBAS LBE5	0	2	1	1	1	1	1	1	1	1	1	1	1	1	1	1	1	1	1	1	1	1	1	1	1	2	–	–	–	–	–	–	–	–	c					
13+22														1	1	1	1	1	1	1	1	1	1	2	–	–	–	–	–	–	–	–	–	–	c					
UNIBAS LBE9	0	2	1	1	1	1	1	1	1	1	1	1	1	1	1	1	1	1	1	1	1	1	1	1	1	1	–	–	–	–	–	–	–	–	c					
13+22														1	1	1	1	1	1	1	1	1	1	1	–	–	–	–	–	–	–	–	–	–	c					
UNIBAS LBF1	0	2	1	1	1	1	1	1	1	1	1	1	1	1	1	1	1	1	1	1	1	1	1	1	1	2	–	–	–	–	–	–	–	–	c					
13+22														1	1	1	1	1	1	1	1	1	1	2	–	–	–	–	–	–	–	–	–	–	c					
UNIBAS LBF2	0	2	1	1	1	1	1	1	1	1	1	1	1	1	1	1	1	1	1	1	1	1	1	1	1	2	–	–	–	–	–	–	–	–	c					
13+22														1	1	1	1	1	1	1	1	1	1	1	–	–	–	–	–	–	–	–	–	–	c					
**Eretmodini**																																								
Eretmodus cyanostictus UNIBAS JDA5	0	2	1	1	1	1	1	1	1	1	1	1	1	1	1	1	1	1	1	1	1	1	1	2	1	1	–	–	–	c										
14+16															2	–	–	1	1	2	1	2	–	–	–	–	–	–	–	c										
UNIBAS JDA7	0	2	1	1	1	1	1	1	1	1	1	1	1	1	1	1	1	1	1	1	1	2	1	1	2	–	–	–	–	c										
14+16															1	–	1	1	1	1	2	1	–	–	–	–	–	–	–	c										
UNIBAS JDA8	0	2	1	1	1	1	1	1	1	1	1	1	1	1	1	1	1	1	1	1	1	1	2	1	2	–	–	–	–	c										
14+16															1	1	–	1	1	2	2	1	–	–	–	–	–	–	–	c										
UNIBAS JDA9	0	2	1	1	1	1	1	1	1	1	1	1	1	1	1	1	1	1	1	1	1	1	2	1	2	–	–	–	–	c										
14+16															1	–	1	1	1	1	2	2	–	–	–	–	–	–	–	c										
UNIBAS JDB1	0	2	1	1	1	1	1	1	1	1	1	1	1	1	1	1	1	1	1	1	1	1	2	1	1	–	–	–	c											
13+16														1	–	1	1	1	1	1	2	–	–	–	–	–	–	–	c											
Eretmodus marksmithi UNIBAS JXF7	0	2	1	1	1	1	1	1	1	1	1	1	1	1	1	1	1	1	1	1	1	2	1	1	2	–	–	–	–	c										
14+16															1	1	1	1	1	2	1	–	–	–	–	–	–	–	–	c										
UNIBAS JXF9	0	2	1	1	1	1	1	1	1	1	1	1	1	1	1	1	1	1	1	1	1	1	2	1	2	–	–	–	–	c										
13+17														1	–	1	1	1	1	2	1	–	–	–	–	–	–	–	–	c										
UNIBAS JXG1	0	2	1	1	1	1	1	1	1	1	1	1	1	1	1	1	1	1	1	1	1	1	2	1	2	–	–	–	–	c										
14+16															1	1	–	1	1	2	2	–	–	–	–	–	–	–	–	c										
UNIBAS JXG3	0	2	1	1	1	1	1	1	1	1	1	1	1	1	1	1	1	1	1	1	1	1	2	1	2	–	–	–	–	c										
13+17														1	–	1	1	1	1	2	1	–	–	–	–	–	–	–	–	c										

Species, Museum, Catalog number / ID<br>Vertebral count	1	2	3	4	5	6	7	8	9	10	11	12	13	14	15	16	17	18	19	20	21	22	23	24	25	26	27	28	29	30	31	32	33	34	35	36	37	38	39	40
UNIBAS JXG4	0	2	1	1	1	1	1	1	1	1	1	1	1	1	1	1	1	1	1	1	1	2	1	1	2	–	–	–	–	c										
13+17														1	–	1	1	1	1	2	1	–	–	–	–	–	–	–	–	c										
Spathodus erythrodon UNIBAS JUC5	0	2	1	1	1	1	1	1	1	1	1	1	1	1	1	1	1	1	1	1	1	1	2	1	2	–	–	–	–	c										
13+17														1	–	1	1	1	1	2	1	–	–	–	–	–	–	–	–	c										
UNIBAS JUC6	0	2	1	1	1	1	1	1	1	1	1	1	1	1	1	1	1	1	1	1	1	1	2	1	2	–	–	–	–	c										
13+17														1	–	1	1	1	1	2	1	–	–	–	–	–	–	–	–	c										
UNIBAS JUC8	0	2	1	1	1	1	1	1	1	1	1	1	1	1	1	1	1	1	1	1	1	2	1	1	2	–	–	–	–	c										
13+17														1	–	1	1	1	1	2	1	–	–	–	–	–	–	–	–	c										
UNIBAS JUC9	0	2	1	1	1	1	1	1	1	1	1	1	1	1	1	1	1	1	1	1	1	1	2	1	2	–	–	–	–	c										
13+17														1	–	1	1	1	1	2	1	–	–	–	–	–	–	–	–	c										
UNIBAS JUD1	0	2	1	1	1	1	1	1	1	1	1	1	1	1	1	1	1	1	1	1	1	1	2	1	2	–	–	–	–	c										
13+17														1	–	1	1	1	1	1	2	–	–	–	–	–	–	–	–	c										
Tanganicodus irsacae UNIBAS JYI1	0	2	1	1	1	1	1	1	1	1	1	1	1	1	1	1	1	1	1	1	1	1	1	2	1	1	–	–	–	c										
13+17														1	–	1	1	1	1	2	1	–	–	–	–	–	–	–	–	c										
UNIBAS JYI3	0	2	1	1	1	1	1	1	1	1	1	1	1	1	1	1	1	1	1	1	1	1	1	2	1	1	–	–	–	c										
13+17														1	–	1	1	1	1	2	1	–	–	–	–	–	–	–	–	c										
UNIBAS JYI4	0	2	1	1	1	1	1	1	1	1	1	1	1	1	1	1	1	1	1	1	1	1	1	2	1	1	–	–	–	c										
13+17														1	–	1	1	1	1	2	2	–	–	–	–	–	–	–	–	c										
UNIBAS JYI5	0	2	1	1	1	1	1	1	1	1	1	1	1	1	1	1	1	1	1	1	1	1	2	1	2	–	–	–	–	c										
13+17														1	–	1	1	1	1	1	2	–	–	–	–	–	–	–	–	c										
UNIBAS JYI6	0	2	1	1	1	1	1	1	1	1	1	1	1	1	1	1	1	1	1	1	1	1	2	1	2	–	–	–	–	c										
13+17														1	–	1	1	1	2	1	1	–	–	–	–	–	–	–	–	c										
**Lamprologini**																																								
Altolamprologus calvus UNIBAS IOB6	0	2	1	1	1	1	1	1	1	1	1	1	1	1	1	1	1	1	1	1	1	2	1	2	–	–	–	–	–	–	c									
13+18														4	1	1	1	1	1	1	1	2	1	1	–	–	–	–	–	–	c									
UNIBAS IOC3	0	2	1	1	1	1	1	1	1	1	1	1	1	1	1	1	1	1	1	1	1	2	1	2	1	–	–	–	–	–	c									
13+18														4	1	1	1	1	1	1	1	1	2	1	–	–	–	–	–	–	c									
UNIBAS IOC5	0	2	1	1	1	1	1	1	1	1	1	1	1	1	1	1	1	1	1	1	1	1	2	2	1	–	–	–	–	–	c									
13+18														4	1	1	1	1	1	1	1	1	1	2	–	–	–	–	–	–	c									
UNIBAS IOE4	0	2	1	1	1	1	1	1	1	1	1	1	1	1	1	1	1	1	1	1	1	1	2	2	1	–	–	–	–	–	c									
13+18														4	1	1	1	1	1	1	1	1	2	2	–	–	–	–	–	–	c									
UNIBAS IOE5	0	2	1	1	1	1	1	1	1	1	1	1	1	1	1	1	1	1	1	1	1	1	2	2	1	–	–	–	–	–	c									
13+18														4	1	1	1	1	1	1	1	1	1	2	1	–	–	–	–	–	c									
Altolamprologus compressiceps UNIBAS IYC9	0	2	1	1	1	1	1	1	1	1	1	1	1	1	1	1	1	1	1	1	1	1	2	2	1	–	–	–	–	–	c									
13+18														3	1	1	1	1	1	1	1	2	2	–	–	–	–	–	–	–	c									
UNIBAS IYE7	0	2	1	1	1	1	1	1	1	1	1	1	1	1	1	1	1	1	1	1	1	1	2	2	–	–	–	–	–	–	c									
13+18														3	1	1	1	1	1	1	1	2	1	1	–	–	–	–	–	–	c									
UNIBAS IYE8	0	2	1	1	1	1	1	1	1	1	1	1	1	1	1	1	1	1	1	1	1	1	2	2	–	–	–	–	–	–	c									
13+18														3	1	1	1	1	1	1	1	2	2	–	–	–	–	–	–	–	c									
UNIBAS JYD1	0	2	1	1	1	1	1	1	1	1	1	1	1	1	1	1	1	1	1	1	1	2	2	–	–	–	–	–	–	c										
13+17														3	1	1	1	1	1	1	1	2	1	–	–	–	–	–	–	c										
UNIBAS JYD2	0	2	1	1	1	1	1	1	1	1	1	1	1	1	1	1	1	1	1	1	2	1	2	–	–	–	–	–	–	c										
13+17														3	1	1	1	1	1	1	1	2	1	–	–	–	–	–	–	c										
Altolamprologus 'compressiceps shell' UNIBAS IRI2	0	2	1	1	1	1	1	1	1	1	1	1	1	1	1	1	1	1	1	1	1	2	2	–	–	–	–	–	–	c										
12+18													2	2	1	1	1	1	1	1	2	2	–	–	–	–	–	–	–	c										
UNIBAS IRI3	0	2	1	1	1	1	1	1	1	1	1	1	1	1	1	1	1	1	1	1	1	2	2	–	–	–	–	–	–	c										
12+18													2	1	1	1	1	1	1	1	1	2	1	–	–	–	–	–	–	c										
UNIBAS IRI5	0	2	1	1	1	1	1	1	1	1	1	1	1	1	1	1	1	1	1	1	1	2	2	–	–	–	–	–	–	c										
12+18													2	1	1	1	1	1	1	1	1	2	1	–	–	–	–	–	–	c										
UNIBAS IRI6	0	2	1	1	1	1	1	1	1	1	1	1	1	1	1	1	1	1	1	1	2	1	2	–	–	–	–	–	c											
12+17													1	2	1	1	1	1	1	1	1	2	–	–	–	–	–	–	c											
UNIBAS IRI7	0	2	1	1	1	1	1	1	1	1	1	1	1	1	1	1	1	1	1	1	1	2	2	–	–	–	–	–	–	c										
12+18													3	1	1	1	1	1	1	1	1	3	–	–	–	–	–	–	–	c										
Chalinochromis brichardi UNIBAS IQF6	0	1	2	1	1	1	1	1	1	1	1	1	1	1	1	1	1	1	1	1	1	1	1	1	2	1	2	–	–	–	–	–	–	c						
17+17																		2	1	1	1	1	1	2	1	1	–	–	–	–	–	–	–	c						
UNIBAS IQF7	0	1	2	1	1	1	1	1	1	1	1	1	1	1	1	1	1	1	1	1	1	1	1	1	2	1	2	–	–	–	–	–	–	c						
17+17																		2	1	1	1	1	1	1	2	1	–	–	–	–	–	–	–	c						
UNIBAS IYC5	0	2	1	1	1	1	1	1	1	1	1	1	1	1	1	1	1	1	1	1	1	1	1	1	2	1	1	–	–	–	–	–	–	c						
17+17																		2	1	1	1	1	1	2	1	–	–	–	–	–	–	–	–	c						
UNIBAS IYC6	0	2	1	1	1	1	1	1	1	1	1	1	1	1	1	1	1	1	1	1	1	1	1	1	2	1	2	–	–	–	–	–	–	c						
17+17																		3	1	1	1	1	1	2	1	1	–	–	–	–	–	–	–	c						
UNIBAS IYD7	0	2	1	1	1	1	1	1	1	1	1	1	1	1	1	1	1	1	1	1	1	1	1	1	2	1	2	–	–	–	–	–	–	c						
17+17																		2	1	1	1	1	1	1	2	1	–	–	–	–	–	–	–	c						
Chalinochromis cyanophleps UNIBAS LGC8	0	–	3	1	1	1	1	1	1	1	1	1	1	1	1	1	1	1	1	1	1	1	1	1	1	2	2	–	–	–	–	–	–	–	c					
17+18																		2	1	1	1	1	1	1	2	1	–	–	–	–	–	–	–	–	c					
UNIBAS LGG6	0	1	2	1	1	1	1	1	1	1	1	1	1	1	1	1	1	1	1	1	1	1	1	1	2	1	2	–	–	–	–	–	–	–	–	c				
17+19																		2	1	1	1	1	1	1	2	1	–	–	–	–	–	–	–	–	–	c				

Species, Museum, Catalog number / ID<br>Vertebral count	1	2	3	4	5	6	7	8	9	10	11	12	13	14	15	16	17	18	19	20	21	22	23	24	25	26	27	28	29	30	31	32	33	34	35	36	37	38	39	40
UNIBAS LGG7	0	2	1	1	1	1	1	1	1	1	1	1	1	1	1	1	1	1	1	1	1	1	1	1	2	1	2	–	–	–	–	–	–	–	–	c				
17+19																		2	1	1	1	1	2	1	1	2	–	–	–	–	–	–	–	–	–	c				
UNIBAS LGG8	0	2	1	1	1	1	1	1	1	1	1	1	1	1	1	1	1	1	1	1	1	1	1	2	1	2	–	–	–	–	–	–	–	–	c					
16+19																	2	1	1	1	1	1	2	1	1	–	–	–	–	–	–	–	–	–	c					
UNIBAS LGG9	0	1	2	1	1	1	1	1	1	1	1	1	1	1	1	1	1	1	1	1	1	1	1	1	1	2	2	–	–	–	–	–	–	–	–	c				
17+19																		2	1	1	1	1	1	1	2	1	–	–	–	–	–	–	–	–	–	c				
Julidochromis ornatus UNIBAS JCF9	0	1	2	1	1	1	1	1	1	1	1	1	1	1	1	1	1	1	1	1	1	1	1	2	1	2	–	–	–	–	–	–	c							
16+17																	2	1	1	1	1	1	1	2	1	–	–	–	–	–	–	–	c							
UNIBAS JCG1	0	2	1	1	1	1	1	1	1	1	1	1	1	1	1	1	1	1	1	1	1	1	2	1	1	2	–	–	–	–	–	–	c							
16+17																	2	1	1	1	1	1	1	2	1	–	–	–	–	–	–	–	c							
UNIBAS JCI2	0	1	2	1	1	1	1	1	1	1	1	1	1	1	1	1	1	1	1	1	1	1	1	1	2	1	1	–	–	–	–	–	c							
16+17																	2	1	1	1	1	1	1	2	1	–	–	–	–	–	–	–	c							
UNIBAS JCI3	0	2	1	1	1	1	1	1	1	1	1	1	1	1	1	1	1	1	1	1	1	1	1	1	2	1	1	–	–	–	–	–	c							
15+18																1	1	1	1	1	1	1	1	1	2	–	–	–	–	–	–	–	c							
UNIBAS JDD6	0	2	1	1	1	1	1	1	1	1	1	1	1	1	1	1	1	1	1	1	1	1	1	1	2	1	1	–	–	–	–	–	c							
16+17																	2	1	1	1	1	1	1	2	1	–	–	–	–	–	–	–	c							
Julidochromis regani UNIBAS KHF1	0	1	2	1	1	1	1	1	1	1	1	1	1	1	1	1	1	1	1	1	1	1	1	1	2	1	2	–	–	–	–	–	–	c						
16+18																	2	–	1	1	1	1	1	1	2	–	–	–	–	–	–	–	–	c						
UNIBAS KHF2	0	2	1	1	1	1	1	1	1	1	1	1	1	1	1	1	1	1	1	1	1	1	1	1	2	2	–	–	–	–	–	–	–	c						
16+18																	2	–	1	1	1	1	1	2	2	–	–	–	–	–	–	–	–	c						
UNIBAS KHF3	0	2	1	1	1	1	1	1	1	1	1	1	1	1	1	1	1	1	1	1	1	1	1	1	2	2	–	–	–	–	–	–	c							
16+17																	2	–	1	1	1	1	1	1	1	–	–	–	–	–	–	–	c							
UNIBAS KHI3	0	2	1	1	1	1	1	1	1	1	1	1	1	1	1	1	1	1	1	1	1	1	1	1	2	2	–	–	–	–	–	–	c							
16+17																	1	1	1	1	1	1	1	2	–	–	–	–	–	–	–	–	c							
UNIBAS KHI4	0	2	1	1	1	1	1	1	1	1	1	1	1	1	1	1	1	1	1	1	1	1	1	1	2	1	–	–	–	–	–	–	c							
16+17																	1	1	1	1	1	1	1	2	–	–	–	–	–	–	–	–	c							
Lamprologus callipterus UNIBAS IMF4	0	1	2	1	1	1	1	1	1	1	1	1	1	1	1	1	1	1	1	1	1	1	1	2	1	1	–	–	–	–	–	–	–	c						
14+20															3	1	1	1	1	1	1	1	2	1	1	–	–	–	–	–	–	–	–	c						
UNIBAS JAB9	0	1	2	1	1	1	1	1	1	1	1	1	1	1	1	1	1	1	1	1	1	1	2	1	2	–	–	–	–	–	–	–	–	c						
14+20															3	1	1	1	1	1	1	1	2	2	–	–	–	–	–	–	–	–	–	c						
UNIBAS JEA4	0	1	2	1	1	1	1	1	1	1	1	1	1	1	1	1	1	1	1	1	1	1	1	2	1	1	–	–	–	–	–	–	–	c						
14+20															3	1	1	1	1	1	1	1	2	1	1	–	–	–	–	–	–	–	–	c						
UNIBAS JEA5	0	1	2	1	1	1	1	1	1	1	1	1	1	1	1	1	1	1	1	1	1	1	2	1	2	–	–	–	–	–	–	–	–	c						
14+20															4	1	1	1	1	1	1	1	2	2	–	–	–	–	–	–	–	–	–	c						
UNIBAS JEA6	0	1	2	1	1	1	1	1	1	1	1	1	1	1	1	1	1	1	1	1	1	1	2	1	2	–	–	–	–	–	–	–	–	c						
14+20															3	1	1	1	1	1	1	1	1	2	1	–	–	–	–	–	–	–	–	c						
UNIBAS JEA7	0	2	1	1	1	1	1	1	1	1	1	1	1	1	1	1	1	1	1	1	1	1	1	2	1	1	–	–	–	–	–	–	–	c						
14+20															3	1	1	1	1	1	1	1	1	2	1	–	–	–	–	–	–	–	–	c						
UNIBAS JEE3	0	1	2	1	1	1	1	1	1	1	1	1	1	1	1	1	1	1	1	1	1	1	1	2	1	1	–	–	–	–	–	–	–	c						
14+20															3	1	1	1	1	1	1	1	2	1	1	–	–	–	–	–	–	–	–	c						
UNIBAS JEE4	0	1	2	1	1	1	1	1	1	1	1	1	1	1	1	1	1	1	1	1	1	1	2	1	2	–	–	–	–	–	–	–	–	c						
14+20															3	1	1	1	1	1	1	1	2	1	1	–	–	–	–	–	–	–	–	c						
UNIBAS JEE5	0	1	2	1	1	1	1	1	1	1	1	1	1	1	1	1	1	1	1	1	1	1	2	1	1	–	–	–	–	–	–	–	–	c						
14+20															3	1	1	1	1	1	1	2	1	1	–	–	–	–	–	–	–	–	–	c						
UNIBAS JEE6	0	1	2	1	1	1	1	1	1	1	1	1	1	1	1	1	1	1	1	1	1	1	1	2	1	1	–	–	–	–	–	–	–	–	c					
14+21															2	1	1	1	1	1	1	1	2	1	1	–	–	–	–	–	–	–	–	–	c					
Lamprologus 'ornatipinnis congo' UNIBAS 93-14	0	2	1	1	1	1	1	1	1	1	1	1	1	1	1	1	1	1	1	1	1	1	2	1	–	–	–	–	–	–	–	–	c							
12+21													1	1	1	1	1	1	1	1	2	1	1	–	–	–	–	–	–	–	–	–	c							
UNIBAS 93-18-a	0	2	1	1	1	1	1	1	1	1	1	1	1	1	1	1	1	1	1	1	1	2	1	1	–	–	–	–	–	–	–	–	c							
12+21													1	1	1	1	1	1	1	1	1	2	1	–	–	–	–	–	–	–	–	–	c							
UNIBAS 93-18-b	0	2	1	1	1	1	1	1	1	1	1	1	1	1	1	1	1	1	1	1	1	1	1	2	–	–	–	–	–	–	–	–	–	c						
12+22													1	1	1	1	1	1	1	1	1	2	1	1	–	–	–	–	–	–	–	–	–	c						
UNIBAS 94-51	0	2	1	1	1	1	1	1	1	1	1	1	1	1	1	1	1	1	1	1	1	1	2	1	–	–	–	–	–	–	–	–	c							
12+21													1	1	1	1	1	1	1	1	1	2	1	–	–	–	–	–	–	–	–	–	c							
UNIBAS 94-60	0	2	1	1	1	1	1	1	1	1	1	1	1	1	1	1	1	1	1	1	1	1	2	1	–	–	–	–	–	–	–	–	c							
12+21													1	1	1	1	1	1	1	1	1	2	1	–	–	–	–	–	–	–	–	–	c							
Lamprologus 'ornatipinnis zambia' UNIBAS ILD7	0	2	1	1	1	1	1	1	1	1	1	1	1	1	1	1	1	1	1	1	1	1	1	2	–	–	–	–	–	–	–	–	c							
12+21													1	1	1	1	1	1	1	1	1	2	1	–	–	–	–	–	–	–	–	–	c							
UNIBAS IVE5	0	2	1	1	1	1	1	1	1	1	1	1	1	1	1	1	1	1	1	1	1	2	2	–	–	–	–	–	–	–	–	–	c							
12+21													1	1	1	1	1	1	1	1	2	1	1	–	–	–	–	–	–	–	–	–	c							
UNIBAS IVE6	0	2	1	1	1	1	1	1	1	1	1	1	1	1	1	1	1	1	1	1	1	1	2	1	–	–	–	–	–	–	–	–	c							
12+21													1	1	1	1	1	1	1	1	1	2	1	–	–	–	–	–	–	–	–	–	c							
UNIBAS IVE7	0	2	1	1	1	1	1	1	1	1	1	1	1	1	1	1	1	1	1	1	1	2	1	1	–	–	–	–	–	–	–	c								
12+20													1	1	1	1	1	1	1	1	2	1	–	–	–	–	–	–	–	–	–	c								
UNIBAS JDH4	0	2	1	1	1	1	1	1	1	1	1	1	1	1	1	1	1	1	1	1	1	1	1	2	–	–	–	–	–	–	–	–	c							
12+21													1	1	1	1	1	1	1	1	1	2	1	–	–	–	–	–	–	–	–	–	c							
UNIBAS JDH5	0	2	1	1	1	1	1	1	1	1	1	1	1	1	1	1	1	1	1	1	1	1	2	–	–	–	–	–	–	–	–	–	c							
12+21													1	1	1	1	1	1	1	1	2	1	1	–	–	–	–	–	–	–	–	–	c							

Species, Museum, Catalog number / ID<br>Vertebral count	1	2	3	4	5	6	7	8	9	10	11	12	13	14	15	16	17	18	19	20	21	22	23	24	25	26	27	28	29	30	31	32	33	34	35	36	37	38	39	40
UNIBAS JDH6	0	2	1	1	1	1	1	1	1	1	1	1	1	1	1	1	1	1	1	1	1	1	2	1	–	–	–	–	–	–	–	–	c							
12+21													1	1	1	1	1	1	1	1	1	2	1	–	–	–	–	–	–	–	–	–	c							
UNIBAS JEE2	0	2	1	1	1	1	1	1	1	1	1	1	1	1	1	1	1	1	1	1	1	1	1	2	–	–	–	–	–	–	–	–	–	c						
12+22													1	1	1	1	1	1	1	1	1	2	1	–	–	–	–	–	–	–	–	–	–	c						
UNIBAS JEH6	0	2	1	1	1	1	1	1	1	1	1	1	1	1	1	1	1	1	1	1	1	1	2	1	–	–	–	–	–	–	–	–	–	c						
12+22													1	1	1	1	1	1	1	1	1	3	–	–	–	–	–	–	–	–	–	–	–	c						
Lamprologus speciosus UNIBAS KCG9	0	2	1	1	1	1	1	1	1	1	1	1	1	1	1	1	1	1	1	2	1	1	–	–	–	–	–	c												
11+17												2	1	1	1	1	1	1	1	2	1	–	–	–	–	–	–	c												
UNIBAS KCH1	0	2	1	1	1	1	1	1	1	1	1	1	1	1	1	1	1	1	1	1	2	1	–	–	–	–	–	c												
11+17												2	1	1	1	1	1	1	1	2	1	–	–	–	–	–	–	c												
UNIBAS KCH2	0	2	1	1	1	1	1	1	1	1	1	1	1	1	1	1	1	1	1	2	1	1	–	–	–	–	–	c												
12+16													2	1	1	1	2	1	1	1	2	–	–	–	–	–	–	c												
UNIBAS KHI5	0	2	1	1	1	1	1	1	1	1	1	1	1	1	1	1	1	1	1	2	1	1	–	–	–	–	–	c												
12+16													3	1	1	1	1	1	2	1	1	–	–	–	–	–	–	c												
UNIBAS KHI6	0	2	1	1	1	1	1	1	1	1	1	1	1	1	1	1	1	1	1	2	2	–	–	–	–	–	c													
11+16												2	1	1	1	1	1	1	2	1	–	–	–	–	–	–	c													
Lepidiolamprologus kendalli UNIBAS IOC1	0	3	1	1	1	1	1	1	1	1	1	1	1	1	1	1	1	1	1	1	1	1	2	1	2	–	–	–	–	–	–	–	–	c						
16+18																	2	1	1	1	1	1	2	2	–	–	–	–	–	–	–	–	–	c						
UNIBAS JBB6	0	2	1	1	1	1	1	1	1	1	1	1	1	1	1	1	1	1	1	1	1	1	2	1	1	2	–	–	–	–	–	–	–	c						
16+18																	2	1	1	1	1	1	2	1	2	–	–	–	–	–	–	–	–	c						
UNIBAS JBB7	0	2	1	1	1	1	1	1	1	1	1	1	1	1	1	1	1	1	1	1	1	1	1	2	2	1	–	–	–	–	–	–	–	c						
16+18																	2	1	1	1	1	1	2	1	2	–	–	–	–	–	–	–	–	c						
UNIBAS JBB8	0	2	1	1	1	1	1	1	1	1	1	1	1	1	1	1	1	1	1	1	1	1	2	1	2	1	–	–	–	–	–	–	–	c						
16+18																	2	1	1	1	1	1	2	1	2	–	–	–	–	–	–	–	–	c						
UNIBAS JEE1	0	2	1	1	1	1	1	1	1	1	1	1	1	1	1	1	1	1	1	1	1	1	1	2	1	2	–	–	–	–	–	–	–	–	c					
16+19																	3	–	1	1	1	1	1	2	2	–	–	–	–	–	–	–	–	–	c					
Lepidiolamprologus mimicus UNIBAS LDD5	0	2	1	1	1	1	1	1	1	1	1	1	1	1	1	1	1	1	1	1	1	1	2	1	2	1	–	–	–	–	–	–	–	–	–	c				
16+20																	2	1	1	1	1	1	1	2	2	–	–	–	–	–	–	–	–	–	–	c				
UNIBAS LDD6	0	2	1	1	1	1	1	1	1	1	1	1	1	1	1	1	1	1	1	1	1	1	2	1	2	1	–	–	–	–	–	–	–	–	c					
16+19																	2	1	1	1	1	1	2	2	–	–	–	–	–	–	–	–	–	–	c					
UNIBAS LDH3	0	2	1	1	1	1	1	1	1	1	1	1	1	1	1	1	1	1	1	1	1	1	2	1	2	1	–	–	–	–	–	–	–	–	c					
16+19																	2	1	1	1	1	1	2	1	2	–	–	–	–	–	–	–	–	–	c					
UNIBAS LGD5	0	2	1	1	1	1	1	1	1	1	1	1	1	1	1	1	1	1	1	1	1	1	1	2	2	1	–	–	–	–	–	–	–	–	c					
16+19																	2	1	1	1	1	1	2	1	2	–	–	–	–	–	–	–	–	–	c					
UNIBAS LGG4	0	2	1	1	1	1	1	1	1	1	1	1	1	1	1	1	1	1	1	1	1	1	2	1	2	1	–	–	–	–	–	–	–	–	c					
16+19																	3	1	1	1	1	1	2	1	1	–	–	–	–	–	–	–	–	–	c					
Lepidiolamprologus profundicola UNIBAS JED3	0	2	1	1	1	1	1	1	1	1	1	1	1	1	1	1	1	1	1	1	1	1	1	2	1	2	–	–	–	–	–	–	–	–	–	c				
16+20																	2	1	1	1	1	1	2	2	1	–	–	–	–	–	–	–	–	–	–	c				
UNIBAS KAD2	0	2	1	1	1	1	1	1	1	1	1	1	1	1	1	1	1	1	1	1	1	1	1	2	1	1	2	–	–	–	–	–	–	–	–	c				
16+20																	2	1	1	1	1	1	2	1	2	–	–	–	–	–	–	–	–	–	–	c				
UNIBAS KEB8	0	2	1	1	1	1	1	1	1	1	1	1	1	1	1	1	1	1	1	1	1	1	2	1	2	1	–	–	–	–	–	–	–	–	–	c				
16+20																	2	1	1	1	1	1	2	1	2	–	–	–	–	–	–	–	–	–	–	c				
UNIBAS KEB9	0	2	1	1	1	1	1	1	1	1	1	1	1	1	1	1	1	1	1	1	1	1	2	1	1	2	–	–	–	–	–	–	–	–	–	c				
16+20																	2	1	1	1	1	1	2	1	2	–	–	–	–	–	–	–	–	–	–	c				
UNIBAS LGD1	0	2	1	1	1	1	1	1	1	1	1	1	1	1	1	1	1	1	1	1	1	1	1	2	1	2	–	–	–	–	–	–	–	–	–	–	c			
16+21																	2	–	1	1	1	1	1	2	1	1	–	–	–	–	–	–	–	–	–	–	c			
Neolamprologus tretocephalus UNIBAS KFI8	0	2	1	1	1	1	1	1	1	1	1	1	1	1	1	1	1	1	1	1	2	1	2	2	–	–	–	–	–	–	–	c								
14+18															1	1	1	1	1	1	1	2	1	–	–	–	–	–	–	–	–	c								
UNIBAS KFI4	0	2	1	1	1	1	1	1	1	1	1	1	1	1	1	1	1	1	1	1	2	1	2	2	–	–	–	–	–	–	–	c								
14+18															1	1	1	1	1	1	2	2	–	–	–	–	–	–	–	–	–	c								
UNIBAS KFI5	0	2	1	1	1	1	1	1	1	1	1	1	1	1	1	1	1	1	1	1	2	1	2	1	–	–	–	–	–	–	–	c								
14+18															1	1	1	1	1	1	2	2	–	–	–	–	–	–	–	–	–	c								
UNIBAS KFI6	0	2	1	1	1	1	1	1	1	1	1	1	1	1	1	1	1	1	1	1	2	1	2	2	–	–	–	–	–	–	–	c								
14+18															1	1	1	1	1	1	1	2	1	–	–	–	–	–	–	–	–	c								
UNIBAS KFI7	0	2	1	1	1	1	1	1	1	1	1	1	1	1	1	1	1	1	1	1	2	1	2	1	1	–	–	–	–	–	–	–	c							
14+19															1	1	1	1	1	1	1	2	1	–	–	–	–	–	–	–	–	–	c							
Neolamprologus ventralis UNIBAS KAD4	0	2	1	1	1	1	1	1	1	1	1	1	1	1	1	1	1	1	1	1	1	1	2	1	–	–	–	–	–	–	–	c								
14+18															1	1	1	1	1	1	1	3	–	–	–	–	–	–	–	–	–	c								
UNIBAS KAG9	0	2	1	1	1	1	1	1	1	1	1	1	1	1	1	1	1	1	1	1	1	2	2	–	–	–	–	–	–	–	–	c								
14+18															2	1	1	1	1	1	2	1	–	–	–	–	–	–	–	–	–	c								
UNIBAS KDF3	0	2	1	1	1	1	1	1	1	1	1	1	1	1	1	1	1	1	1	1	1	1	2	1	–	–	–	–	–	–	–	c								
14+18															2	1	1	1	1	1	1	2	1	–	–	–	–	–	–	–	–	c								
UNIBAS KDF4	0	2	1	1	1	1	1	1	1	1	1	1	1	1	1	1	1	1	1	1	1	2	1	1	–	–	–	–	–	–	–	c								
13+19														1	1	1	1	1	1	1	1	2	–	–	–	–	–	–	–	–	–	c								
UNIBAS KDF5	0	2	1	1	1	1	1	1	1	1	1	1	1	1	1	1	1	1	1	1	1	1	2	2	–	–	–	–	–	–	–	c								
14+18															2	1	1	1	1	1	1	2	–	–	–	–	–	–	–	–	–	c								
Telmatochromis dhonti UNIBAS IZG9	0	2	1	1	1	1	1	1	1	1	1	1	1	1	1	1	1	1	1	1	1	1	1	2	1	–	–	–	–	–	–	c								
14+18															1	1	1	1	1	1	1	1	2	1	–	–	–	–	–	–	–	c								

**Species, Museum, Catalog number / ID** **Vertebral count**	1	2	3	4	5	6	7	8	9	10	11	12	13	14	15	16	17	18	19	20	21	22	23	24	25	26	27	28	29	30	31	32	33	34	35	36	37	38	39	40
UNIBAS IZH3	0	2	1	1	1	1	1	1	1	1	1	1	1	1	1	1	1	1	1	1	1	1	1	2	1	–	–	–	–	–	–	c								
14+18															1	1	1	1	1	1	1	1	2	1	–	–	–	–	–	–	–	c								
UNIBAS IZH4	–	2	1	1	1	1	1	1	1	1	1	1	1	1	1	1	1	1	1	1	1	1	1	2	1	–	–	–	–	–	–	c								
14+18															1	2	1	1	1	1	1	1	1	2	–	–	–	–	–	–	–	c								
UNIBAS IZH5	0	2	1	1	1	1	1	1	1	1	1	1	1	1	1	1	1	1	1	1	1	1	1	2	1	–	–	–	–	–	–	c								
14+18															1	1	1	1	1	1	1	1	2	1	–	–	–	–	–	–	–	c								
UNIBAS IZH6	0	2	1	1	1	1	1	1	1	1	1	1	1	1	1	1	1	1	1	1	1	1	1	2	1	1	–	–	–	–	–	–	c							
15+18																2	1	1	1	1	1	1	1	2	–	–	–	–	–	–	–	–	c							
Telmatochromis temporalis UNIBAS IND4	0	2	1	1	1	1	1	1	1	1	1	1	1	1	1	1	1	1	1	1	1	1	1	1	2	1	–	–	–	–	–	–	c							
15+18																1	1	1	1	1	1	1	1	2	1	–	–	–	–	–	–	–	c							
UNIBAS IND5	0	2	1	1	1	1	1	1	1	1	1	1	1	1	1	1	1	1	1	1	1	1	1	1	2	1	1	–	–	–	–	c								
15+17																1	1	1	1	1	1	1	1	2	1	1	–	–	–	–	–	c								
UNIBAS IQA7	0	2	1	1	1	1	1	1	1	1	1	1	1	1	1	1	1	1	1	1	1	1	1	2	1	–	–	–	–	–	–	–	c							
14+19															1	1	1	1	1	1	1	1	1	2	–	–	–	–	–	–	–	–	c							
UNIBAS IYH6	0	2	1	1	1	1	1	1	1	1	1	1	1	1	1	1	1	1	1	1	1	1	1	2	1	1	–	–	–	–	–	–	c							
15+18																1	1	1	1	1	1	1	2	1	1	–	–	–	–	–	–	–	c							
UNIBAS JAD5	0	2	1	1	1	1	1	1	1	1	1	1	1	1	1	1	1	1	1	1	1	1	1	1	2	2	–	–	–	–	–	–	c							
15+18																1	1	1	1	1	1	1	1	2	1	–	–	–	–	–	–	–	c							
Telmatochromis vittatus UNIBAS IRC1	0	2	1	1	1	1	1	1	1	1	1	1	1	1	1	1	1	1	1	1	1	1	1	2	1	1	–	–	–	–	–	–	–	c						
16+18																	2	1	1	1	1	1	1	2	1	–	–	–	–	–	–	–	–	c						
UNIBAS JBD7	0	2	1	1	1	1	1	1	1	1	1	1	1	1	1	1	1	1	1	1	1	1	1	1	1	2	1	–	–	–	–	–	–	–	c					
17+18																		2	1	1	1	1	1	1	2	1	–	–	–	–	–	–	–	–	c					
UNIBAS LPA4	–	1	2	1	1	1	1	1	1	1	1	1	1	1	1	1	1	1	1	1	1	1	1	1	2	1	2	–	–	–	–	–	–	–	–	c				
17+19																		2	1	1	1	1	1	1	2	1	–	–	–	–	–	–	–	–	–	c				
NHMUK 1898.9.9.19–20 syntypes small	–	2	1	1	1	1	1	1	1	1	1	1	1	1	1	1	1	1	1	1	1	1	1	1	2	2	–	–	–	–	–	–	–	c						
16+18																	2	1	1	1	1	1	1	2	1	–	–	–	–	–	–	–	–	c						
larger	–	2	1	1	1	1	1	1	1	1	1	1	1	1	1	1	1	1	1	1	1	1	1	1	2	1	1	–	–	–	–	–	–	–	c					
16+19																	2	1	1	1	1	1	1	1	2	–	–	–	–	–	–	–	–	–	c					
Variabilichromis moorii UNIBAS IYC2	0	2	1	1	1	1	1	1	1	1	1	1	1	1	1	1	1	1	1	1	2	1	2	1	2	–	–	–	–	–	–	c								
16+16																	4	1	1	1	2	1	1	2	–	–	–	–	–	–	–	c								
UNIBAS IYC3	0	2	1	1	1	1	1	1	1	1	1	1	1	1	1	1	1	1	1	1	1	2	1	2	2	–	–	–	–	–	–	–	c							
16+17																	4	1	1	1	1	2	1	2	–	–	–	–	–	–	–	–	c							
UNIBAS IYC4	0	2	1	1	1	1	1	1	1	1	1	1	1	1	1	1	1	1	1	1	1	2	1	2	2	–	–	–	–	–	–	c								
16+16																	4	1	1	1	1	2	1	2	1	–	–	–	–	–	–	c								
UNIBAS IYC7	0	2	1	1	1	1	1	1	1	1	1	1	1	1	1	1	1	1	1	1	2	1	1	2	2	–	–	–	–	–	–	c								
16+16																	4	1	2	1	1	1	2	1	–	–	–	–	–	–	–	c								
UNIBAS IYC8	0	2	1	1	1	1	1	1	1	1	1	1	1	1	1	1	1	1	1	1	1	2	1	2	2	–	–	–	–	–	–	–	c							
16+17																	4	1	1	2	1	1	2	1	1	–	–	–	–	–	–	–	c							
UNIBAS IYH8	0	2	1	1	1	1	1	1	1	1	1	1	1	1	1	1	1	1	1	1	1	2	1	2	2	–	–	–	–	–	–	c								
16+16																	4	1	1	1	2	1	1	2	1	–	–	–	–	–	–	c								
UNIBAS LNF5	0	2	1	1	1	1	1	1	1	1	1	1	1	1	1	1	1	1	1	1	1	2	1	2	2	–	–	–	–	–	–	c								
16+16																	4	1	1	1	2	1	1	2	1	–	–	–	–	–	–	c								
**Limnochromini**																																								
Baileychromis centropomoides UNIBAS JCC6	0	1	2	1	1	1	1	1	1	1	1	1	1	1	1	1	1	1	1	1	1	1	2	1	–	–	–	–	–	–	–	–	c							
15+18																1	2	1	–	1	1	2	1	–	–	–	–	–	–	–	–	–	c							
UNIBAS JCC9	0	2	1	1	1	1	1	1	1	1	1	1	1	1	1	1	1	1	1	1	1	1	2	1	–	–	–	–	–	–	–	–	c							
15+18																1	1	1	1	1	2	1	2?	–	–	–	–	–	–	–	–	–	c							
UNIBAS JCD1	0	1	2	1	1	1	1	1	1	1	1	1	1	1	1	1	1	1	1	1	1	1	2	1	–	–	–	–	–	–	–	–	c							
15+18																1	1	1	1	1	1	2	1	–	–	–	–	–	–	–	–	–	c							
UNIBAS LBF4	0	1	2	1	1	1	1	1	1	1	1	1	1	1	1	1	1	1	1	1	1	1	2	1	–	–	–	–	–	–	–	–	c							
15+18																1	1	–	1	2	1	1	2	–	–	–	–	–	–	–	–	–	c							
UNIBAS LBF5	0	1	2	1	1	1	1	1	1	1	1	1	1	1	1	1	1	1	1	1	1	1	2	1	1	–	–	–	–	–	–	–	–	c						
15+19																2	–	1	1	1	1	2	2	–	–	–	–	–	–	–	–	–	–	c						
Gnathochromis permaxillaris UNIBAS IXD5	0	2	1	1	1	1	1	1	1	1	1	1	1	1	1	1	1	1	1	1	1	1	2	2	–	–	–	–	–	–	–	–	c							
15+18																1	1	1	1	1	1	2	2	–	–	–	–	–	–	–	–	–	c							
UNIBAS IXD6	0	2	1	1	1	1	1	1	1	1	1	1	1	1	1	1	1	1	1	1	1	2	1	2	–	–	–	–	–	–	–	–	c							
15+18																2	–	–	1	1	2	1	2	–	–	–	–	–	–	–	–	–	c							
UNIBAS IXD7	0	2	1	1	1	1	1	1	1	1	1	1	1	1	1	1	1	1	1	1	1	2	1	2	–	–	–	–	–	–	–	–	c							
15+18																2	–	–	1	1	2	1	2	–	–	–	–	–	–	–	–	–	c							
UNIBAS IXD8	0	2	1	1	1	1	1	1	1	1	1	1	1	1	1	1	1	1	1	1	1	2	2	–	–	–	–	–	–	–	–	c								
15+17																1	1	1	1	1	1	2	–	–	–	–	–	–	–	–	–	c								
UNIBAS IXD9	0	2	1	1	1	1	1	1	1	1	1	1	1	1	1	1	1	1	1	1	1	2	1	2	–	–	–	–	–	–	–	–	c							
15+18																1	–	1	1	1	2	1	2	–	–	–	–	–	–	–	–	–	c							
Greenwoodochromis christyi UNIBAS JCB6	0	2	1	1	1	1	1	1	1	1	1	1	1	1	1	1	1	1	1	1	2	2	–	–	–	–	–	–	–	–	c									
14+17															1	1	1	1	2	1	2	–	–	–	–	–	–	–	–	–	c									
UNIBAS JCE4	0	2	1	1	1	1	1	1	1	1	1	1	1	1	1	1	1	1	1	1	2	2	1	–	–	–	–	–	–	–	–	c								
14+18															2	–	–	1	1	2	1	2	–	–	–	–	–	–	–	–	–	c								

Species, Museum, Catalog number / ID<br>Vertebral count	1	2	3	4	5	6	7	8	9	10	11	12	13	14	15	16	17	18	19	20	21	22	23	24	25	26	27	28	29	30	31	32	33	34	35	36	37	38	39	40
UNIBAS JCE6	0	1	2	1	1	1	1	1	1	1	1	1	1	1	1	1	1	1	1	2	1	2	–	–	–	–	–	–	–	–	c									
14+17															2	1	1	–	1	2	1	1	–	–	–	–	–	–	–	–	c									
UNIBAS JCE7	0	2	1	1	1	1	1	1	1	1	1	1	1	1	1	1	1	1	1	1	2	1	1	–	–	–	–	–	–	–	c									
14+17															1	1	1	1	1	2	1	1	–	–	–	–	–	–	–	–	c									
UNIBAS JCE8	0	2	1	1	1	1	1	1	1	1	1	1	1	1	1	1	1	1	1	1	2	2	1	–	–	–	–	–	–	–	–	c								
14+18															1	–	1	1	1	2	1	2	–	–	–	–	–	–	–	–	–	c								
Jabarichromis pfefferi UNIBAS ISH6	0	2	1	1	1	1	1	1	1	1	1	1	1	1	1	1	1	1	1	2	1	2	1	–	–	–	–	–	–	c										
14+16															1	1	1	1	1	2	2	–	–	–	–	–	–	–	–	c										
UNIBAS IWC7	0	2	1	1	1	1	1	1	1	1	1	1	1	1	1	1	1	1	1	2	1	3	–	–	–	–	–	–	–	c										
13+17														1	–	1	1	1	1	1	2	–	–	–	–	–	–	–	–	c										
UNIBAS IWG8	0	2	1	1	1	1	1	1	1	1	1	1	1	1	1	1	1	1	1	2	1	2	1	–	–	–	–	–	–	c										
14+16															1	1	1	1	1	1	2	1	–	–	–	–	–	–	–	c										
UNIBAS IWG9	0	2	1	1	1	1	1	1	1	1	1	1	1	1	1	1	1	1	1	2	1	2	1	–	–	–	–	–	–	c										
13+17														1	–	1	1	1	1	2	2	–	–	–	–	–	–	–	–	c										
UNIBAS IWH1	0	2	1	1	1	1	1	1	1	1	1	1	1	1	1	1	1	1	2	1	1	2	–	–	–	–	–	–	–	c										
14+16															1	1	1	1	1	2	1	–	–	–	–	–	–	–	–	c										
Limnochromis auritus MCZ 50835	0	2	1	1	1	1	1	1	1	1	1	1	1	1	1	1	1	1	1	1	1	2	2	–	–	–	–	–	–	–	–	–	c							
15+18																1	1	1	1	2	1	2	–	–	–	–	–	–	–	–	–	–	c							
UNIBAS JCA1	0	2	1	1	1	1	1	1	1	1	1	1	1	1	1	1	1	1	1	1	2	1	2	–	–	–	–	–	–	–	–	c								
14+18															1	–	1	1	1	2	2	1	–	–	–	–	–	–	–	–	–	c								
UNIBAS JCD9	0	2	1	1	1	1	1	1	1	1	1	1	1	1	1	1	1	1	1	1	1	2	1	2	–	–	–	–	–	–	–	–	c							
15+18																1	1	1	1	1	2	2	–	–	–	–	–	–	–	–	–	–	c							
UNIBAS JCE1	0	2	1	1	1	1	1	1	1	1	1	1	1	1	1	1	1	1	1	1	1	2	2	–	–	–	–	–	–	–	–	–	c							
14+19															1	–	1	1	1	2	2	1	–	–	–	–	–	–	–	–	–	–	c							
UNIBAS JEH2	0	2	1	1	1	1	1	1	1	1	1	1	1	1	1	1	1	1	1	1	1	2	1	2	–	–	–	–	–	–	–	–	c							
15+18																1	1	1	1	1	2	2	–	–	–	–	–	–	–	–	–	–	c							
Reganochromis calliurus UNIBAS IYB4	0	3	1	1	1	1	1	1	1	1	1	1	1	1	1	1	1	1	1	1	1	1	2	1	1	–	–	–	–	–	–	–	–	c						
15+19																2	–	1	1	1	1	1	2	–	–	–	–	–	–	–	–	–	–	c						
UNIBAS IZB5	0	2	1	1	1	1	1	1	1	1	1	1	1	1	1	1	1	1	1	1	1	1	1	2	1	1	–	–	–	–	–	–	–	–	c					
16+19																	2	–	1	1	1	1	2	1	–	–	–	–	–	–	–	–	–	–	c					
UNIBAS JCD3	0	2	1	1	1	1	1	1	1	1	1	1	1	1	1	1	1	1	1	1	1	1	1	1	2	1	–	–	–	–	–	–	–	–	c					
16+19																	2	–	1	1	1	2	1	1	–	–	–	–	–	–	–	–	–	–	c					
UNIBAS JCD4	0	2	1	1	1	1	1	1	1	1	1	1	1	1	1	1	1	1	1	1	1	1	1	1	2	1	1	–	–	–	–	–	–	–	c					
16+19																	2	–	1	1	1	1	2	2	–	–	–	–	–	–	–	–	–	–	c					
UNIBAS JCD5	0	2	1	1	1	1	1	1	1	1	1	1	1	1	1	1	1	1	1	1	1	1	2	1	2	–	–	–	–	–	–	–	–	c						
15+19																2	–	1	1	1	2	1	2	–	–	–	–	–	–	–	–	–	–	c						
Tangachromis dhanisi MCZ 50836	0	2	1	1	1	1	1	1	1	1	1	1	1	1	1	1	1	1	1	1	2	1	1	–	–	–	–	–	–	–	–	c								
14+18															1	1	1	1	1	1	2	–	–	–	–	–	–	–	–	–	–	c								
UNIBAS LJA7	0	2	1	1	1	1	1	1	1	1	1	1	1	1	1	1	1	1	1	2	1	–	–	–	–	–	–	–	–	c										
13+17														2	–	1	1	1	2	1	–	–	–	–	–	–	–	–	–	c										
UNIBAS LJA8	0	2	2	1	1	1	–	1	1	1	1	1	1	1	1	1	1	1	1	1	2	1	–	–	–	–	–	–	–	c										
13+17														1	1	–	1	2	1	2	–	–	–	–	–	–	–	–	–	c										
UNIBAS LJA9	0	2	1	1	1	1	1	1	1	1	1	1	1	1	1	1	1	1	1	2	2	–	–	–	–	–	–	–	–	c										
13+17														1	–	1	1	1	2	2	1	–	–	–	–	–	–	–	–	c										
Trematochromis benthicola UNIBAS DMC6	0	2	1	1	1	1	1	1	1	1	1	1	1	1	1	1	1	1	1	1	2	2	1	–	–	–	–	–	–	–	c									
14+17															2	–	–	1	1	1	2	1	–	–	–	–	–	–	–	–	c									
UNIBAS DMC7	0	2	1	1	1	1	1	1	1	1	1	1	1	1	1	1	1	1	1	1	2	2	1	–	–	–	–	–	–	–	c									
14+17															2	–	–	1	1	2	1	1	–	–	–	–	–	–	–	–	c									
UNIBAS DMC8	0	2	1	1	1	1	1	1	1	1	1	1	1	1	1	1	1	1	1	1	2	2	1	–	–	–	–	–	–	–	c									
14+17															2	–	–	1	1	1	2	1	–	–	–	–	–	–	–	–	c									
UNIBAS DMC9	0	2	1	1	1	1	1	1	1	1	1	1	1	1	1	1	1	1	1	2	2	2	–	–	–	–	–	–	–	–	c									
14+17															2	–	–	1	1	2	2	–	–	–	–	–	–	–	–	–	c									
UNIBAS DMD2	0	2	1	1	1	1	1	1	1	1	1	1	1	1	1	1	1	1	1	1	2	2	2	–	–	–	–	–	–	–	–	c								
14+18															1	–	1	1	–	2	1	**3**	–	–	–	–	–	–	–	–	–	c								
Triglachromis otostigma UNIBAS JBB3	0	2	1	1	1	1	1	1	1	1	1	1	1	1	1	1	1	1	1	1	1	1	2	–	–	–	–	–	–	–	–	c								
15+17																1	–	1	1	1	2	1	2	–	–	–	–	–	–	–	–	c								
UNIBAS JEG6	0	2	1	1	1	1	1	1	1	1	1	1	1	1	1	1	1	1	1	1	1	1	2	–	–	–	–	–	–	–	–	–	c							
15+18																1	–	1	1	1	1	2	1	1	–	–	–	–	–	–	–	–	c							
UNIBAS JEG7	0	2	1	1	1	1	1	1	1	1	1	1	1	1	1	1	1	1	1	1	1	1	1	–	–	–	–	–	–	–	–	c								
14+18															1	–	1	1	1	1	1	2	1	–	–	–	–	–	–	–	–	c								
UNIBAS KAI9	0	2	1	1	1	1	1	1	1	1	1	1	1	1	1	1	1	1	1	1	1	1	2	–	–	–	–	–	–	–	–	c								
14+18															1	–	1	1	1	1	2	1	1	–	–	–	–	–	–	–	–	c								
UNIBAS KCA1	0	2	1	1	1	1	1	1	1	1	1	1	1	1	1	1	1	1	1	1	1	2	1	–	–	–	–	–	–	–	–	c								
15+17																1	1	1	1	1	2	1	1	–	–	–	–	–	–	–	–	c								
**Oreochromini**																																								
Oreochromis tanganicae UNIBAS LGD7	0	2	1	1	1	1	1	1	1	1	1	1	1	1	1	1	1	1	1	2	2	2	2	2	–	–	–	–	–	–	c									
16+15																	1	–	2	1	2	2	2	–	–	–	–	–	–	–	c									

Species, Museum, Catalog number / ID<br>Vertebral count	1	2	3	4	5	6	7	8	9	10	11	12	13	14	15	16	17	18	19	20	21	22	23	24	25	26	27	28	29	30	31	32	33	34	35	36	37	38	39	40
UNIBAS LGD8	0	2	1	1	1	1	1	1	1	1	1	1	1	1	1	1	1	1	1	2	1	2	3	2	–	–	–	–	–	–	c									
16+15																	1	–	2	1	2	2	2	–	–	–	–	–	–	–	c									
UNIBAS LGD9	0	2	1	1	1	1	1	1	1	1	1	1	1	1	1	1	1	1	1	2	2	2	2	1	–	–	–	–	–	–	c									
16+15																	1	–	2	1	2	2	2	1	–	–	–	–	–	–	c									
UNIBAS LIB3	0	2	1	1	1	1	1	1	1	1	1	1	1	1	1	1	1	1	1	2	2	2	2	1	–	–	–	–	–	–	c									
16+15																	2	–	1	2	1	2	2	–	–	–	–	–	–	–	c									
UNIBAS LIB4	0	2	1	1	1	1	1	1	1	1	1	1	1	1	1	1	1	1	1	2	2	1	3	1	–	–	–	–	–	–	c									
16+15																	2	–	1	2	2	2	2	–	–	–	–	–	–	–	c									
**Perissodini**																																								
Haplotaxodon microlepis UNIBAS ITG5	0	2	1	1	1	1	1	1	1	1	1	1	1	1	1	1	1	1	1	1	1	1	2	1	2	2	–	–	–	–	–	–	–	–	–	–	c			
17+20																		1	1	1	1	2	1	2	2	–	–	–	–	–	–	–	–	–	–	–	c			
UNIBAS ITG6	0	2	1	1	1	1	1	1	1	1	1	1	1	1	1	1	1	1	1	1	1	1	1	2	1	2	1	–	–	–	–	–	–	–	–	c				
17+19																		1	1	1	1	1	2	2	1	–	–	–	–	–	–	–	–	–	–	c				
UNIBAS JEA3	0	2	1	1	1	1	1	1	1	1	1	1	1	1	1	1	1	1	1	1	1	1	2	1	2	2	–	–	–	–	–	–	–	–	–	c				
17+19																		2	–	1	1	1	1	2	2	–	–	–	–	–	–	–	–	–	–	c				
UNIBAS JED7	0	2	1	1	1	1	1	1	1	1	1	1	1	1	1	1	1	1	1	1	1	1	2	1	2	1	–	–	–	–	–	–	–	–	–	c				
17+19																		1	1	1	1	2	1	2	1	–	–	–	–	–	–	–	–	–	–	c				
UNIBAS JED8	0	2	1	1	1	1	1	1	1	1	1	1	1	1	1	1	1	1	1	1	1	1	2	1	2	1	–	–	–	–	–	–	–	–	–	c				
17+19																		1	1	1	1	2	2	2	–	–	–	–	–	–	–	–	–	–	–	c				
Perissodus microlepis UNIBAS IPE9	0	1	2	1	1	1	1	1	1	1	1	1	1	1	1	1	1	1	1	1	1	1	2	2	2	–	–	–	–	–	–	–	–	c						
16+18																	2	–	1	1	1	2	2	2	–	–	–	–	–	–	–	–	–	c						
UNIBAS IPG1	0	1	2	1	1	1	1	1	1	1	1	1	1	1	1	1	1	1	1	1	1	2	1	2	2	–	–	–	–	–	–	–	–	c						
16+18																	1	1	1	1	1	2	1	3	–	–	–	–	–	–	–	–	–	c						
UNIBAS JAD7	0	–	3	1	1	1	1	1	1	1	1	1	1	1	1	1	1	1	1	2	2	1	2	1	2	1	–	–	–	–	–	–	–	c						
16+18																	2	–	–	1	1	1	1	2	2	–	–	–	–	–	–	–	–	c						
UNIBAS JAD8	0	–	3	1	1	1	1	1	1	1	1	1	1	1	1	1	1	1	1	1	1	1	2	1	2	2	–	–	–	–	–	–	–	–	c					
16+19																	2	–	–	2	1	1	1	2	2	–	–	–	–	–	–	–	–	–	c					
UNIBAS KAG6	0	1	2	1	1	1	1	1	1	1	1	1	1	1	1	1	1	1	1	1	2	1	2	1	2	–	–	–	–	–	–	–	–	c						
15+19																1	–	1	1	2	1	2	2	–	–	–	–	–	–	–	–	–	–	c						
Plecodus paradoxus UNIBAS KEH9	0	2	1	1	1	1	1	1	1	1	1	1	1	1	1	1	1	1	1	1	1	1	1	1	1	2	1	2	1	–	–	–	–	–	–	–	c			
17+20																		1	1	–	1	1	1	2	1	2	1	2	–	–	–	–	–	–	–	–	c			
UNIBAS KEI1	0	2	1	1	1	1	1	1	1	1	1	1	1	1	1	1	1	1	1	1	1	1	1	1	1	2	1	2	1	–	–	–	–	–	–	–	–	c		
18+20																			1	1	1	1	1	1	2	1	2	2	–	–	–	–	–	–	–	–	–	c		
UNIBAS LEA1	0	2	1	1	1	1	1	1	1	1	1	1	1	1	1	1	1	1	1	1	1	1	1	1	1	1	2	1	2	1	–	–	–	–	–	–	–	–	c	
18+21																			1	1	–	1	1	1	2	1	1	2	2	–	–	–	–	–	–	–	–	–	c	
UNIBAS LEA2	0	2	1	1	1	1	1	1	1	1	1	1	1	1	1	1	1	1	1	1	1	1	1	1	1	2	1	2	1	–	–	–	–	–	–	–	–	c		
18+20																			1	1	1	1	1	1	2	1	2	2	–	–	–	–	–	–	–	–	–	c		
UNIBAS LEC6	0	2	1	1	1	1	1	1	1	1	1	1	1	1	1	1	1	1	1	1	1	1	1	1	1	2	1	2	1	–	–	–	–	–	–	–	–	c		
18+20																			1	1	1	1	1	1	2	1	2	2	–	–	–	–	–	–	–	–	–	c		
Xenochromis hecqui UNIBAS IZC1	0	2	1	1	1	1	1	1	1	1	1	1	1	1	1	1	1	1	1	1	1	1	2	1	2	1	–	–	–	–	–	–	–	c						
15+19																1	–	1	1	1	1	1	2	2	1	–	–	–	–	–	–	–	–	c						
UNIBAS IZC2	0	2	1	1	1	1	1	1	1	1	1	1	1	1	1	1	1	1	1	1	1	1	2	2	1	–	–	–	–	–	–	–	–	c						
15+19																1	–	1	1	1	1	2	1	2	1	–	–	–	–	–	–	–	–	c						
UNIBAS JAC8	0	2	1	1	1	1	1	1	1	1	1	1	1	1	1	1	1	1	1	1	1	1	2	1	2	–	–	–	–	–	–	–	–	c						
15+19																1	–	1	1	1	1	2	1	2	1	–	–	–	–	–	–	–	–	c						
UNIBAS LBC3	0	2	1	1	1	1	1	1	1	1	1	1	1	1	1	1	1	1	1	1	1	1	1	2	2	–	–	–	–	–	–	–	–	c						
16+18																	1	1	1	1	1	1	2	2	1	–	–	–	–	–	–	–	–	c						
UNIBAS LBD4	0	2	1	1	1	1	1	1	1	1	1	1	1	1	1	1	1	1	1	1	1	1	2	1	2	–	–	–	–	–	–	–	–	c						
15+19																1	–	1	1	1	1	2	1	2	1	–	–	–	–	–	–	–	–	c						
**Pseudocrenilabrini: Tropheina**																																								
Interochromis loocki UNIBAS IYE5	0	2	1	1	1	1	1	1	1	1	1	1	1	1	1	1	1	1	1	2	1	2	2	–	–	–	–	–	–	–	c									
15+16																1	1	–	1	2	1	2	–	–	–	–	–	–	–	–	c									
UNIBAS IYF7	0	2	1	1	1	1	1	1	1	1	1	1	1	1	1	1	1	1	1	2	1	2	2	–	–	–	–	–	–	–	c									
15+16																1	1	1	1	1	2	1	–	–	–	–	–	–	–	–	c									
UNIBAS IYF8	0	2	1	1	1	1	1	1	1	1	1	1	1	1	1	1	1	1	1	1	2	1	2	2	–	–	–	–	–	–	c									
15+16																1	–	1	1	1	2	1	2	–	–	–	–	–	–	–	c									
UNIBAS IYG8	0	2	1	1	1	1	1	1	1	1	1	1	1	1	1	1	1	1	1	1	2	2	2	–	–	–	–	–	–	–	c									
15+16																1	1	–	1	2	1	2	–	–	–	–	–	–	–	–	c									
UNIBAS IYG9	0	2	1	1	1	1	1	1	1	1	1	1	1	1	1	1	1	1	1	2	1	2	2	–	–	–	–	–	–	–	c									
15+16																1	1	1	1	1	1	2	–	–	–	–	–	–	–	–	c									
Limnotilapia dardennii UNIBAS GPB6	0	2	1	1	1	1	1	1	1	1	1	1	1	1	1	1	1	1	1	1	1	2	1	2	2	–	–	–	–	–	–	–	–	c						
16+18																	1	–	1	1	1	2	1	2	–	–	–	–	–	–	–	–	–	c						
UNIBAS GPF5	0	2	1	1	1	1	1	1	1	1	1	1	1	1	1	1	1	1	1	1	1	2	1	2	1	–	–	–	–	–	–	–	–	c						
16+18																	1	1	–	1	1	2	1	1	–	–	–	–	–	–	–	–	–	c						
UNIBAS IMD6	0	2	1	1	1	1	1	1	1	1	1	1	1	1	1	1	1	1	1	1	1	2	1	2	1	–	–	–	–	–	–	–	–	c						
16+18																	1	1	1	–	2	1	2	1	–	–	–	–	–	–	–	–	–	c						
UNIBAS JBF6	0	2	1	1	1	1	1	1	1	1	1	1	1	1	1	1	1	1	1	1	1	2	1	2	1	–	–	–	–	–	–	–	–	c						
16+18																	1	–	1	1	1	2	1	2	–	–	–	–	–	–	–	–	–	c						

Species, Museum, Catalog number / ID<br>Vertebral count	1	2	3	4	5	6	7	8	9	10	11	12	13	14	15	16	17	18	19	20	21	22	23	24	25	26	27	28	29	30	31	32	33	34	35	36	37	38	39	40
Lobochilotes labiatus UNIBAS IYB9	0	2	1	1	1	1	1	1	1	1	1	1	1	1	1	1	1	1	1	2	1	2	2	1	–	–	–	–	–	–	–	c								
15+17																1	1	–	1	2	1	2	–	–	–	–	–	–	–	–	–	c								
UNIBAS IYE2	0	2	1	1	1	1	1	1	1	1	1	1	1	1	1	1	1	1	1	1	2	2	1	2	–	–	–	–	–	–	–	c								
15+17																1	–	1	1	1	2	1	1	–	–	–	–	–	–	–	–	c								
UNIBAS IYF9	0	2	1	1	1	1	1	1	1	1	1	1	1	1	1	1	1	1	1	2	1	2	2	1	–	–	–	–	–	–	–	c								
15+17																2	–	–	1	1	2	2	–	–	–	–	–	–	–	–	–	c								
UNIBAS IYG2	0	2	1	1	1	1	1	1	1	1	1	1	1	1	1	1	1	1	1	1	2	2	1	2	–	–	–	–	–	–	–	c								
15+17																1	1	–	1	1	2	2	–	–	–	–	–	–	–	–	–	c								
UNIBAS IYG3	0	2	1	1	1	1	1	1	1	1	1	1	1	1	1	1	1	1	1	1	2	2	1	2	–	–	–	–	–	–	–	c								
15+17																1	–	1	1	1	2	1	1	–	–	–	–	–	–	–	–	c								
Petrochromis horii UNIBAS IWB9	0	2	1	1	1	1	1	1	1	1	1	1	1	1	1	1	1	1	1	1	2	1	2	2	–	–	–	–	–	–	–	c								
16+16																	1	1	1	1	1	2	2	1	–	–	–	–	–	–	–	c								
UNIBAS IWC1	0	2	1	1	1	1	1	1	1	1	1	1	1	1	1	1	1	1	1	1	1	2	1	2	2	–	–	–	–	–	–	–	c							
16+17																	2	–	–	1	1	2	2	1	–	–	–	–	–	–	–	–	c							
UNIBAS IWC2	0	2	1	1	1	1	1	1	1	1	1	1	1	1	1	1	1	1	1	1	1	2	1	2	2	–	–	–	–	–	–	–	c							
16+17																	2	–	–	1	1	2	2	1	–	–	–	–	–	–	–	–	c							
UNIBAS JBF8	0	2	1	1	1	1	1	1	1	1	1	1	1	1	1	1	1	1	1	1	1	2	1	2	2	–	–	–	–	–	–	c								
16+16																	2	–	–	1	1	2	1	2	–	–	–	–	–	–	–	c								
UNIBAS JBF9	0	2	1	1	1	1	1	1	1	1	1	1	1	1	1	1	1	1	1	1	1	2	2	2	–	–	–	–	–	–	–	c								
16+16																	2	–	–	1	1	2	2	–	–	–	–	–	–	–	–	c								
Petrochromis polyodon UNIBAS IYD4	0	2	1	1	1	1	1	1	1	1	1	1	1	1	1	1	1	1	1	1	2	1	2	1	–	–	–	–	–	–	–	c								
16+16																	1	1	1	1	2	1	2	–	–	–	–	–	–	–	–	c								
UNIBAS IYD5	0	2	1	1	1	1	1	1	1	1	1	1	1	1	1	1	1	1	1	1	2	1	2	1	–	–	–	–	–	–	–	c								
15+17																2	–	–	1	2	1	2	1	–	–	–	–	–	–	–	–	c								
UNIBAS IYE9	0	2	1	1	1	1	1	1	1	1	1	1	1	1	1	1	1	1	1	1	2	1	2	1	–	–	–	–	–	–	–	c								
16+16																	1	1	1	1	2	2	1	–	–	–	–	–	–	–	–	c								
UNIBAS IYG5	0	1	2	1	1	1	1	1	1	1	1	1	1	1	1	1	1	1	1	1	2	1	2	2	–	–	–	–	–	–	–	c								
16+16																	2	–	1	1	1	2	2	–	–	–	–	–	–	–	–	c								
UNIBAS JXD2	0	2	1	1	1	1	1	1	1	1	1	1	1	1	1	1	1	1	1	1	2	1	2	1	–	–	–	–	–	–	–	c								
16+16																	1	1	1	1	2	1	1	–	–	–	–	–	–	–	–	c								
Pseudosimochromis curvifrons UNIBAS IYG4	0	2	1	1	1	1	1	1	1	1	1	1	1	1	1	1	1	1	1	1	2	1	2	1	–	–	–	–	–	–	c									
15+16																1	1	–	1	2	1	2	1	–	–	–	–	–	–	–	c									
UNIBAS JBC1	0	2	1	1	1	1	1	1	1	1	1	1	1	1	1	1	1	1	1	1	2	2	2	–	–	–	–	–	–	–	c									
15+16																1	1	1	1	1	2	1	2	–	–	–	–	–	–	–	c									
UNIBAS JBC2	0	2	1	1	1	1	1	1	1	1	1	1	1	1	1	1	1	1	1	1	1	2	2	2	–	–	–	–	–	–	–	c								
15+17																1	–	1	1	1	1	2	2	1	–	–	–	–	–	–	–	c								
UNIBAS JBC3	0	2	1	1	1	1	1	1	1	1	1	1	1	1	1	1	1	1	1	1	2	1	2	1	–	–	–	–	–	–	c									
15+16																1	1	1	1	1	1	2	1	–	–	–	–	–	–	–	c									
UNIBAS JCA2	0	2	1	1	1	1	1	1	1	1	1	1	1	1	1	1	1	1	1	1	2	1	2	1	–	–	–	–	–	–	c									
15+16																1	1	1	1	1	1	2	1	–	–	–	–	–	–	–	c									
Shuja horei UNIBAS JDB6	0	2	1	1	1	1	1	1	1	1	1	1	1	1	1	1	1	1	1	2	1	2	–	–	–	–	–	–	–	c										
13+17														1	1	–	1	1	1	2	1	–	–	–	–	–	–	–	–	c										
UNIBAS JDC8	0	2	1	1	1	1	1	1	1	1	1	1	1	1	1	1	1	1	2	1	2	–	–	–	–	–	–	–	c											
13+16														1	1	–	1	1	2	2	–	–	–	–	–	–	–	–	c											
UNIBAS JDD9	0	2	1	1	1	1	1	1	1	1	1	1	1	1	1	1	1	1	1	2	2	1	–	–	–	–	–	–	–	c										
13+17														1	–	1	1	1	1	2	1	–	–	–	–	–	–	–	–	c										
UNIBAS JDE1	0	2	1	1	1	1	1	1	1	1	1	1	1	1	1	1	1	1	1	2	1	2	1	–	–	–	–	–	–	c										
13+17														1	1	–	1	1	1	1	2	–	–	–	–	–	–	–	–	c										
UNIBAS KAE4	0	2	1	1	1	1	1	1	1	1	1	1	1	1	1	1	1	1	1	2	2	1	–	–	–	–	–	–	–	c										
13+17														1	1	1	–	1	2	1	1	–	–	–	–	–	–	–	–	c										
NHMUK 1889.1.30.13–15 (syntypes) Genner '22 a	0	2	1	1	1	1	1	1	1	1	1	1	1	1	1	1	1	1	1	2	1	2	–	–	–	–	–	–	c											
13+16														1	–	1	1	1	1	2	1	–	–	–	–	–	–	–	c											
b	0	2	1	1	1	1	1	1	1	1	1	1	1	1	1	1	1	1	1	2	2	1	–	–	–	–	–	–	–	c										
13+17														1	–	1	1	1	1	2	1	–	–	–	–	–	–	–	–	c										
c	0	2	1	1	1	1	1	1	1	1	1	1	1	1	1	1	1	1	1	2	1	2	–	–	–	–	–	–	–	c										
13+17														1	–	1	1	1	1	2	1	–	–	–	–	–	–	–	–	c										
Simochromis diagramma UNIBAS JDB7	0	2	1	2	–	1	1	1	1	1	1	1	1	1	1	1	1	1	1	1	2	1	2	2	–	–	–	–	–	–	–	c								
16+16																	1	1	1	1	1	1	2	1	–	–	–	–	–	–	–	c								
UNIBAS JDB8	0	2	1	1	1	1	1	1	1	1	1	1	1	1	1	1	1	1	1	1	1	2	1	2	1	–	–	–	–	–	–	–	c							
16+17																	1	1	1	1	1	1	2	1	–	–	–	–	–	–	–	–	c							
UNIBAS JDB9	0	2	1	1	1	1	1	1	1	1	1	1	1	1	1	1	1	1	1	1	2	1	2	2	–	–	–	–	–	–	–	c								
15+17																1	–	1	1	1	1	2	1	1	–	–	–	–	–	–	–	c								
UNIBAS JDC1	0	2	1	1	1	1	1	1	1	1	1	1	1	1	1	1	1	1	1	1	2	1	2	2	–	–	–	–	–	–	–	c								
15+17																1	–	1	1	1	1	2	2	–	–	–	–	–	–	–	–	c								
UNIBAS JDC2	0	2	1	1	1	1	1	1	1	1	1	1	1	1	1	1	1	1	1	1	2	1	2	2	1	–	–	–	–	–	–	–	c							
15+18																1	–	1	1	1	1	2	2	–	–	–	–	–	–	–	–	–	c							
Tropheus brichardi UNIBAS JZA6	0	2	1	1	1	1	1	1	1	1	1	1	1	1	1	1	1	1	1	1	2	1	2	1	–	–	–	–	–	c										
15+15																1	1	1	2	1	1	2	1	–	–	–	–	–	–	c										

Species, Museum, Catalog number / ID<br>Vertebral count	1	2	3	4	5	6	7	8	9	10	11	12	13	14	15	16	17	18	19	20	21	22	23	24	25	26	27	28	29	30	31	32	33	34	35	36	37	38	39	40
UNIBAS JZA8	0	2	1	1	1	1	1	1	1	1	1	1	1	1	1	1	1	1	1	2	1	2	1	–	–	–	–	–	c											
14+15															1	1	1	1	1	1	2	2	–	–	–	–	–	–	c											
UNIBAS JZA9	0	2	1	1	1	1	1	1	1	1	1	1	1	1	1	1	1	1	1	1	1	2	1	2	–	–	–	–	–	c										
15+15																1	1	1	1	1	2	1	2	–	–	–	–	–	–	c										
UNIBAS JZB1	0	2	1	1	1	1	1	1	1	1	1	1	1	1	1	1	1	1	1	1	2	2	1	–	–	–	–	–	c											
14+15															2	–	1	1	1	1	2	2	–	–	–	–	–	–	c											
UNIBAS JZB2	0	2	1	1	1	1	1	1	1	1	1	1	1	1	1	1	1	1	1	1	2	1	2	1	–	–	–	–	–	c										
15+15																1	1	1	1	1	2	1	2	–	–	–	–	–	–	c										
Tropheus duboisi UNIBAS KHF7	0	2	1	1	1	1	1	1	1	1	1	1	1	1	1	1	1	1	1	1	1	2	1	2	–	–	–	–	–	c										
15+15																1	1	–	1	1	2	1	2	–	–	–	–	–	–	c										
UNIBAS KHF8	0	2	1	1	1	1	1	1	1	1	1	1	1	1	1	1	1	1	1	1	1	2	1	2	–	–	–	–	–	–	c									
15+16																1	–	1	1	1	2	1	2	–	–	–	–	–	–	–	c									
UNIBAS KHF9	0	2	1	1	1	1	1	1	1	1	1	1	1	1	1	1	1	1	1	1	2	1	2	1	–	–	–	–	–	c										
15+15																1	1	1	1	1	1	2	1	–	–	–	–	–	–	c										
UNIBAS KHG1	0	2	1	1	1	1	1	1	1	1	1	1	1	1	1	1	1	1	1	1	2	1	2	1	–	–	–	–	–	c										
15+15																1	1	1	1	1	1	2	1	–	–	–	–	–	–	c										
UNIBAS KHI7	0	2	1	1	1	1	1	1	1	1	1	1	1	1	1	1	1	1	1	1	1	2	1	2	–	–	–	–	–	c										
15+15																1	1	–	1	2	1	2	1	–	–	–	–	–	–	c										
Tropheus moorii UNIBAS GPF3	0	2	1	1	1	1	1	1	1	1	1	1	1	1	1	1	1	1	1	1	1	2	1	2	1	–	–	–	–	–	c									
15+16																1	1	1	1	1	1	2	2	1	–	–	–	–	–	–	c									
UNIBAS GPF4	0	2	1	1	1	1	1	1	1	1	1	1	1	1	1	1	1	1	1	1	1	2	1	2	1	–	–	–	–	–	c									
15+16																1	1	1	1	1	1	2	2	–	–	–	–	–	–	–	c									
UNIBAS IMA9	0	2	1	1	1	1	1	1	1	1	1	1	1	1	1	1	1	1	1	1	1	2	1	2	–	–	–	–	–	–	c									
15+16																1	1	1	1	1	2	1	2	–	–	–	–	–	–	–	c									
UNIBAS IMF5	0	2	1	1	1	1	1	1	1	1	1	1	1	1	1	1	1	1	1	1	1	2	1	2	–	–	–	–	–	–	c									
15+16																1	1	1	1	1	2	1	2	–	–	–	–	–	–	–	c									
UNIBAS IMG6	0	2	1	1	1	1	1	1	1	1	1	1	1	1	1	1	1	1	1	1	2	1	2	1	–	–	–	–	–	c										
15+15																1	1	1	1	1	2	1	2	–	–	–	–	–	–	c										
UNIBAS IOC2	0	2	1	1	1	1	1	1	1	1	1	1	1	1	1	1	1	1	1	1	2	1	1	2	–	–	–	–	–	c										
15+15																1	1	1	1	1	2	1	2	–	–	–	–	–	–	c										
UNIBAS JBH6	0	2	1	1	1	1	1	1	1	1	1	1	1	1	1	1	1	1	1	1	1	2	1	2	–	–	–	–	–	–	c									
15+16																1	1	1	1	1	1	2	1	1	–	–	–	–	–	–	c									
UNIBAS JBH7	0	2	1	1	1	1	1	1	1	1	1	1	1	1	1	1	1	1	1	1	1	2	1	2	1	–	–	–	–	–	c									
15+16																1	1	1	1	1	1	2	1	1	–	–	–	–	–	–	c									
UNIBAS JBH9	0	2	1	1	1	1	1	1	1	1	1	1	1	1	1	1	1	1	1	1	1	2	1	2	–	–	–	–	–	–	c									
15+16																1	1	1	1	1	2	1	2	–	–	–	–	–	–	–	c									
UNIBAS JBI1	0	2	1	1	1	1	1	1	1	1	1	1	1	1	1	1	1	1	1	1	2	1	1	2	1	–	–	–	–	c										
15+15																1	1	1	1	1	2	1	2	1	–	–	–	–	–	c										
Tropheus polli UNIBAS LEG6	0	2	1	1	1	1	1	1	1	1	1	1	1	1	1	1	1	1	1	1	2	1	1	2	1	–	–	–	–	–	c									
16+15																	1	1	1	1	1	2	2	–	–	–	–	–	–	–	c									
UNIBAS LEG7	0	2	1	1	1	1	1	1	1	1	1	1	1	1	1	1	1	1	1	1	2	1	1	3	–	–	–	–	–	–	c									
15+16																1	–	1	1	1	2	1	2	–	–	–	–	–	–	–	c									
UNIBAS LEG8	0	2	1	1	1	1	1	1	1	1	1	1	1	1	1	1	1	1	1	1	2	1	2	1	1	–	–	–	–	–	c									
15+16																1	1	–	1	1	2	1	2	1	–	–	–	–	–	–	c									
UNIBAS LEG9	0	2	1	1	1	1	1	1	1	1	1	1	1	1	1	1	1	1	1	1	2	1	1	2	1	–	–	–	–	–	c									
15+16																1	–	1	1	1	2	1	2	1	–	–	–	–	–	–	c									
UNIBAS LEH1	0	2	1	1	1	1	1	1	1	1	1	1	1	1	1	1	1	1	1	1	2	1	1	2	1	–	–	–	–	–	c									
15+16																1	–	1	1	1	1	2	2	1	–	–	–	–	–	–	c									
**Tylochromini**																																								
Tylochromis polylepis UNIBAS GPB4	0	0-2	1	1	1	1	1	1	1	1	1	1	1	1	1	1	1	1	2	1	2	2	3	–	–	–	–	–	–	–	c									
15+16																1	1	1	1	2	2	–	–	–	–	–	–	–	–	–	c									
UNIBAS KCA2	0	0-2	1	1	1	1	1	1	1	1	1	1	1	1	1	1	1	1	2	1	2	2	3	–	–	–	–	–	–	–	c									
14+17															1	–	1	1	1	2	2	–	–	–	–	–	–	–	–	–	c									
UNIBAS KCA3	0	0-2	1	1	1	1	1	1	1	1	1	1	1	1	1	1	1	1	2	1	2	1	3	1	–	–	–	–	–	–	c									
15+16																1	1	–	1	2	2	1	–	–	–	–	–	–	–	–	c									
UNIBAS LIC8	0	0-2	1	1	1	1	1	1	1	1	1	1	1	1	1	1	1	1	2	1	2	2	2	1	–	–	–	–	–	–	c									
14+17															1	–	1	1	1	1	2	2	–	–	–	–	–	–	–	–	c									
UNIBAS LIC9	0	0-2	1	1	1	1	1	1	1	1	1	1	1	1	1	1	1	1	2	1	2	2	2	2	–	–	–	–	–	–	c									
15+16																1	1	1	1	1	2	2	–	–	–	–	–	–	–	–	c									
UNIBAS LID1	0	0-2	1	1	1	1	1	1	1	1	1	1	1	1	1	1	1	2	1	2	1	3	2	–	–	–	–	–	–	–	c									
15+16																1	1	1	2	1	3	–	–	–	–	–	–	–	–	–	c									
**Lake Malawi**																																								
**Pseudocrenilabrini: Cyrtocarina**																																								
Alticorpus mentale CUMV 86278	0	2	1	1	1	1	1	1	1	1	1	1	1	1	1	1	1	2	1	2	2	–	–	–	–	–	–	–	–	–	–	c								
13+19														1	1	1	1	2	2	3	–	–	–	–	–	–	–	–	–	–	–	c								
Alticorpus peterdaviesi USNM 210714 paratype	0	2	1	1	1	1	1	1	1	1	1	1	1	1	1	1	1	1	2	1	3	–	–	–	–	–	–	–	–	–	c									
12+19													1	1	–	1	1	2	2	2	–	–	–	–	–	–	–	–	–	–	c									

**Species, Museum, Catalog number / ID** **Vertebral count**	1	2	3	4	5	6	7	8	9	10	11	12	13	14	15	16	17	18	19	20	21	22	23	24	25	26	27	28	29	30	31	32	33	34	35	36	37	38	39	40
Aristochromis christyi USNM 329636	0	2	1	1	1	1	1	1	1	1	1	1	1	1	1	1	1	1	1	1	2	2	3	–	–	–	–	–	–	–	–	–	c							
14+19															2	–	–	1	2	1	2	3	–	–	–	–	–	–	–	–	–	–	c							
	0	2	1	1	1	1	1	1	1	1	1	1	1	1	1	1	1	1	1	1	2	2	2	1	–	–	–	–	–	–	–	–	c							
14+19															1	–	1	1	2	2	1	3	1	–	–	–	–	–	–	–	–	–	c							
Aulonocara nyassae YPM 027961	0	2	1	1	1	1	1	1	1	1	1	1	1	1	1	1	1	2	1	2	2	1	–	–	–	–	–	–	–	–	c									
13+18														1	1	–	1	1	2	2	1	–	–	–	–	–	–	–	–	–	c									
Aulonocara rostratum YPM 014345	0	2	1	1	1	1	1	1	1	1	1	1	1	1	1	1	1	1	2	1	2	2	–	–	–	–	–	–	–	–	–	c								
13+19														1	–	1	1	1	2	1	3	–	–	–	–	–	–	–	–	–	–	c								
Aulonocara stonemani USNM 210697 Holotype	0	2	1	1	1	1	1	1	1	1	1	1	1	1	1	1	1	1	2	2	2	1	–	–	–	–	–	–	–	–	c									
13+18														1	1	–	1	2	2	2	–	–	–	–	–	–	–	–	–	–	c									
Buccochromis atritaeniatus NHMUK 1921.9.6.179 Lectotype	0	2	1	1	1	1	1	1	1	1	1	1	1	1	1	1	1	1	1	2	1	4	–	–	–	–	–	–	–	–	–	c								
14+18															2	–	–	1	2	1	2	2	–	–	–	–	–	–	–	–	–	c								
NHMUK 1921.9.6.180 paralectotype	0	2	1	1	1	1	1	1	1	1	1	1	1	1	1	1	1	1	1	2	1	3	–	–	–	–	–	–	–	–	–	c								
14+18															1	1	1	–	2	2	2	1	–	–	–	–	–	–	–	–	–	c								
Buccochromis heterotaenia NHMUK 1935.6.14.1419 Lectotype	0	2	1	1	1	1	1	1	1	1	1	1	1	1	1	1	1	1	1	1	2	2	2	1	–	–	–	–	–	–	–	–	c							
15+18																1	1	1	1	1	2	2	2	–	–	–	–	–	–	–	–	–	c							
NHMUK 1935.6.14.1420 paralectotype	0	2	1	1	1	1	1	1	1	1	1	1	1	1	1	1	1	1	1	1	2	2	2	1	–	–	–	–	–	–	–	–	c							
15+18																1	1	1	1	2	1	2	2	–	–	–	–	–	–	–	–	–	c							
Buccochromis nototaenia YPM 023201	0	2	1	1	1	1	1	1	1	1	1	1	1	1	1	1	1	1	1	1	2	1	2	2	–	–	–	–	–	–	–	–	–	c						
15+19																1	1	1	1	1	1	2	2	1?	–	–	–	–	–	–	–	–	–	c						
NHMUK 1906.9.7.21 holotype	0	0-2	1	1	1	1	1	1	1	1	1	1	1	1	1	1	1	1	1	1	2	1	2	2	1	–	–	–	–	–	–	–	c							
15+18																1	1	–	1	2	1	2	3	–	–	–	–	–	–	–	–	–	c							
Buccochromis oculatus NHMUK 1935.6.14.1442-3 paralectos	0	2	1	1	1	1	1	1	1	1	1	1	1	1	1	1	1	1	1	1	2	1	3	1	–	–	–	–	–	–	–	–	–	c						
15+19																1	1	1	1	1	2	3	1	–	–	–	–	–	–	–	–	–	–	c						
	0	2	1	1	1	1	1	1	1	1	1	1	1	1	1	1	1	1	1	1	2	2	2	–	–	–	–	–	–	–	–	–	c							
15+18																1	1	1	2	1	2	3	–	–	–	–	–	–	–	–	–	–	c							
	0	2	1	1	1	1	1	1	1	1	1	1	1	1	1	1	1	1	1	2	1	3	1	–	–	–	–	–	–	–	–	–	c							
15+18																1	1	1	2	1	2	2	–	–	–	–	–	–	–	–	–	–	c							
Buccochromis spectabilis NHMUK 1935.6.14.1448-1452	0	2	1	1	1	1	1	1	1	1	1	1	1	1	1	1	1	1	1	2	1	1	2	2	1	–	–	–	–	–	–	–	–	c						
15+19																1	1	1	1	1	1	2	3	–	–	–	–	–	–	–	–	–	–	c						
Caprichromis orthognathus AMNH 31767	0	2	1	1	1	1	1	1	1	1	1	1	1	1	1	1	1	1	2	1	2	2	1	–	–	–	–	–	–	–	c									
14+17															1	1	1	1	1	2	2	1	–	–	–	–	–	–	–	–	c									
	0	2	1	1	1	1	1	1	1	1	1	1	1	1	1	1	1	1	1	1	2	1	3	1	–	–	–	–	–	–	–	c								
14+18															1	–	1	1	2	1	2	2	1	–	–	–	–	–	–	–	–	c								
	0	2	1	1	1	1	1	1	1	1	1	1	1	1	1	1	1	1	2	2	2	1	–	–	–	–	–	–	–	c										
14+16															1	1	1	1	2	2	2	–	–	–	–	–	–	–	–	c										
	0	2	2	–	1	1	1	1	1	1	1	1	1	1	1	1	1	1	2	1	2	2	–	–	–	–	–	–	–	–	c									
14+17															1	1	1	1	2	2	2	–	–	–	–	–	–	–	–	–	c									
YPM 014341	0	2	1	1	1	1	1	1	1	1	1	1	1	1	1	1	1	1	1	1	2	1	2	2	–	–	–	–	–	–	–	–	c							
15+18																1	1	1	1	1	2	1	3	–	–	–	–	–	–	–	–	–	c							
Champsochromis caeruleus NHMUK 1935.6.14.1267–1269 1	0	2	1	1	1	1	1	1	1	1	1	1	1	1	1	1	1	1	1	1	2	1	2	2	1	–	–	–	–	–	–	–	–	–	c					
15+20																1	–	1	1	2	1	2	2	1	–	–	–	–	–	–	–	–	–	–	c					
2	0	2	1	1	1	1	1	1	1	1	1	1	1	1	1	1	1	1	1	1	1	1	2	2	2	–	–	–	–	–	–	–	–	–	c					
15+20																1	–	1	1	1	2	1	2	2	–	–	–	–	–	–	–	–	–	–	c					
3	0	2	1	1	1	1	1	1	1	1	1	1	1	1	1	1	1	1	1	1	1	2	1	2	2	1	–	–	–	–	–	–	–	–	c					
16+19																	1	1	1	1	2	1	2	2	–	–	–	–	–	–	–	–	–	–	c					
4	0	0-1	2	1	1	1	1	1	1	1	1	1	1	1	1	1	1	1	1	1	1	2	1	2	3	–	–	–	–	–	–	–	–	–	c					
15+20																1	–	1	1	1	2	1	2	2	–	–	–	–	–	–	–	–	–	–	c					
Champsochromis spilorhynchus USNM 265522	0	2	1	1	1	1	1	1	1	1	1	1	1	1	1	1	1	1	1	1	1	2	1	2	2	–	–	–	–	–	–	–	–	c						
16+18																	1	1	1	1	1	1	2	2	–	–	–	–	–	–	–	–	–	c						
USNM 266872	0	2	1	1	1	1	1	1	1	1	1	1	1	1	1	1	1	1	1	1	2	1	2	2	1	–	–	–	–	–	–	–	–	c						
16+18																	1	1	1	1	1	2	2	1	–	–	–	–	–	–	–	–	–	c						
Cheilochromis euchilus NHMUK 1935.6.14:1016-17 Lectotype	0	2	1	1	1	1	1	1	1	1	1	1	1	1	1	1	1	1	2	1	2	1	–	–	–	–	–	–	–	c										
13+17														1	–	1	1	1	2	2	2	–	–	–	–	–	–	–	–	c										
paralectotype (smaller)	0	2	1	1	1	1	1	1	1	1	1	1	1	1	1	1	1	1	2	1	3	1	–	–	–	–	–	–	–	–	c									
14+17															1	1	1	1	2	2	2	–	–	–	–	–	–	–	–	–	c									
AMNH 222094	0	2	1	1	1	1	1	1	1	1	1	1	1	1	1	1	1	1	2	1	2	2	–	–	–	–	–	–	–	c										
14+16															1	1	1	1	1	2	2	1	–	–	–	–	–	–	–	c										
	0	2	1	1	1	1	1	1	1	1	1	1	1	1	1	1	1	1	1	2	1	3	–	–	–	–	–	–	–	–	c									
14+17															1	1	1	1	1	2	3	–	–	–	–	–	–	–	–	–	c									
	0	2	1	1	1	1	1	1	1	1	1	1	1	1	1	1	1	1	2	2	2	1	–	–	–	–	–	–	–	c										
13+17														1	–	1	1	1	2	2	2	–	–	–	–	–	–	–	–	c										
Chilotilapia rhoadesii AMNH 31860 a	0	2	1	1	1	1	1	1	1	1	1	1	1	1	1	1	1	1	1	2	1	2	2	–	–	–	–	–	–	–	c									
14+17															1	1	1	1	2	2	2	1	–	–	–	–	–	–	–	–	c									
b	0	2	1	1	1	1	1	1	1	1	1	1	1	1	1	1	1	1	2	1	1	3	1	–	–	–	–	–	–	–	c									
14+17															1	1	1	1	2	1	2	2	–	–	–	–	–	–	–	–	c									
AMNH 31861	0	1	2	1	1	1	1	1	1	1	1	1	1	1	1	1	1	1	2	1	2	2	–	–	–	–	–	–	–	–	c									
14+17															1	1	1	1	2	2	2	1	–	–	–	–	–	–	–	–	c									

Species, Museum, Catalog number / ID<br>Vertebral count	1	2	3	4	5	6	7	8	9	10	11	12	13	14	15	16	17	18	19	20	21	22	23	24	25	26	27	28	29	30	31	32	33	34	35	36	37	38	39	40
YPM 007852	0	2	1	1	1	1	1	1	1	1	1	1	1	1	1	1	1	1	1	2	2	2	–	–	–	–	–	–	–	–	c									
14+17															1	1	–	1	2	1	2	2	–	–	–	–	–	–	–	–	c									
Copadichromis jacksoni NHMUK 1962.10.18.83-91	0	1	2	1	1	1	1	1	1	1	1	1	1	1	1	1	1	1	1	1	1	2	2	2	1	–	–	–	–	–	–	–	c							
paratypes 15+18																1	1	1	1	1	2	2	1	3	–	–	–	–	–	–	–	–	c							
	0	2	1	1	1	1	1	1	1	1	1	1	1	1	1	1	1	1	1	1	2	1	2	2	2	–	–	–	–	–	–	–	c							
15+18																2	–	1	1	2	1	2	2	2	–	–	–	–	–	–	–	–	c							
	0	2	1	1	1	1	1	1	1	1	1	1	1	1	1	1	1	1	1	1	2	1	2	2	2	–	–	–	–	–	–	–	c							
15+18																1	1	1	1	2	1	2	1	3	–	–	–	–	–	–	–	–	c							
Copadichromis quadrimaculatus YPM 014347	0	2	1	1	1	1	1	1	1	1	1	1	1	1	1	1	1	1	1	1	1	2	1	3	–	–	–	–	–	–	–	c								
15+17																2	–	1	1	1	2	1	2	2	–	–	–	–	–	–	–	c								
Corematodus taeniatus USNM 330794 1	0	2	1	1	1	1	1	1	1	1	1	1	1	1	1	1	1	1	2	1	3	1	–	–	–	–	–	–	–	–	c									
14+17															1	1	2	1	1	3	1	–	–	–	–	–	–	–	–	–	c									
2	0	2	1	1	1	1	1	1	1	1	1	1	1	1	1	1	1	1	1	2	2	2	1?	–	–	–	–	–	–	–	c									
14+17															1	1	1	2	1	2	3	–	–	–	–	–	–	–	–	–	c									
3	0	2	1	1	1	1	1	1	1	1	1	1	1	1	1	1	1	1	2	1	2	2	–	–	–	–	–	–	–	–	c									
13+18														2	–	–	1	1	2	2	2	1	–	–	–	–	–	–	–	–	c									
4	0	2	1	1	1	1	1	1	1	1	1	1	1	1	1	1	1	1	2	1	2	2	–	–	–	–	–	–	–	–	c									
13+18														2	–	1	1	1	2	1	3	–	–	–	–	–	–	–	–	–	c									
5	0	2	1	1	1	1	1	1	1	1	1	1	1	1	1	1	1	1	2	1	2	2	–	–	–	–	–	–	–	–	c									
13+18														2	–	1	1	1	1	2	2	–	–	–	–	–	–	–	–	–	c									
6	0	2	1	1	1	1	1	1	1	1	1	1	1	1	1	1	1	1	1	2	2	2	–	–	–	–	–	–	–	–	c									
13+18														1	–	2	–	2	1	2	2	1	–	–	–	–	–	–	–	–	c									
7	0	2	1	1	1	1	1	1	1	1	1	1	1	1	1	1	1	1	1	2	2	2	–	–	–	–	–	–	–	–	c									
14+17															1	1	1	2	1	2	3	–	–	–	–	–	–	–	–	–	c									
Ctenopharynx nitidus NHMUK1935.6.14:1760-62 Paralectos	0	2	1	1	1	1	1	1	1	1	1	1	1	1	1	1	1	1	1	2	1	2	3*	–	–	–	–	–	–	–	–	c								
15+17																1	1	1	1	2	1	3	–	–	–	–	–	–	–	–	–	c								
	0	2	1	1	1	1	1	1	1	1	1	1	1	1	1	1	1	1	1	2	1	2	3	–	–	–	–	–	–	–	–	c								
15+17																1	1	1	1	2	1	3	–	–	–	–	–	–	–	–	–	c								
	0	2	1	1	1	1	1	1	1	1	1	1	1	1	1	1	1	1	1	1	2	1	2	2	–	–	–	–	–	–	–	c								
15+17																1	1	1	1	2	1	3	–	–	–	–	–	–	–	–	–	c								
AMNH 31784 a	0	2	1	1	1	1	1	1	1	1	1	1	1	1	1	1	1	1	1	2	1	2	3	–	–	–	–	–	–	–	–	c								
14+18															1	1	–	1	2	1	2	2	–	–	–	–	–	–	–	–	–	c								
b	0	2	1	1	1	1	1	1	1	1	1	1	1	1	1	1	1	1	1	2	1	2	3	–	–	–	–	–	–	–	–	c								
14+18															1	1	–	1	2	1	2	2	–	–	–	–	–	–	–	–	–	c								
c	0	2	1	1	1	1	1	1	1	1	1	1	1	1	1	1	1	1	1	1	1	2	2	2	1	–	–	–	–	–	–	–	c							
15+18																1	1	1	1	1	1	2	3*	–	–	–	–	–	–	–	–	–	c							
d	0	2	1	1	1	1	1	1	1	1	1	1	1	1	1	1	1	1	1	1	2	1	2	2	–	–	–	–	–	–	–	c								
15+17																2	–	1	1	2	1	2	2	–	–	–	–	–	–	–	–	c								
e	0	2	1	1	1	1	1	1	1	1	1	1	1	1	1	1	1	1	1	1	2	1	2	2	–	–	–	–	–	–	–	c								
15+17																1	1	1	1	1	2	1	2	–	–	–	–	–	–	–	–	c								
f	0	2	1	1	1	1	1	1	1	1	1	1	1	1	1	1	1	1	1	2	1	2	2	2	–	–	–	–	–	–	–	c								
14+18															2	–	1	2	1	2	1	2	–	–	–	–	–	–	–	–	–	c								
AMNH 31790	0	2	1	1	1	1	1	1	1	1	1	1	1	1	1	1	1	1	1	2	1	2	2	–	–	–	–	–	–	–	–	c								
15+17																2	–	1	1	2	1	3	–	–	–	–	–	–	–	–	–	c								
AMNH 31836 a	0	2	1	1	1	1	1	1	1	1	1	1	1	1	1	1	1	1	1	1	2	1	2	2	–	–	–	–	–	–	–	c								
15+17																1	1	1	1	2	1	2	2	–	–	–	–	–	–	–	–	c								
b	0	2	1	1	1	1	1	1	1	1	1	1	1	1	1	1	1	1	1	2	1	2	2	–	–	–	–	–	–	–	–	c								
15+17																1	1	1	1	1	2	3	–	–	–	–	–	–	–	–	–	c								
c	0	2	1	1	1	1	1	1	1	1	1	1	1	1	1	1	1	1	1	2	1	2	2	1	–	–	–	–	–	–	–	c								
14+18															1	1	1	1	1	1	2	2	1	–	–	–	–	–	–	–	–	c								
d	0	2	1	1	1	1	1	1	1	1	1	1	1	1	1	1	1	1	1	2	1	2	2	1	–	–	–	–	–	–	–	c								
15+17																1	1	1	1	2	1	2	1	–	–	–	–	–	–	–	–	c								
e	0	2	1	1	1	1	1	1	1	1	1	1	1	1	1	1	1	1	1	2	1	2	2	1	–	–	–	–	–	–	–	c								
15+17																1	1	1	2	1	2	2	–	–	–	–	–	–	–	–	–	c								
f	0	2	1	1	1	1	1	1	1	1	1	1	1	1	1	1	1	1	1	2	1	2	2	1	–	–	–	–	–	–	–	c								
14+18															1	1	1	1	1	1	2	2	–	–	–	–	–	–	–	–	–	c								
Ctenopharynx pictus NHMUK	0	2	1	1	1	1	1	1	1	1	1	1	1	1	1	1	1	1	2	1	2	2	1	–	–	–	–	–	–	–	c									
14+17															1	1	1	–	2	1	2	2	–	–	–	–	–	–	–	–	c									
Cyrtocara moorii NHMUK 1906.9.7:26 Holotype	0	2	1	1	1	1	1	1	1	1	1	1	1	1	1	1	1	1	1	1	2	1	2	2	–	–	–	–	–	–	–	–	c							
14+19															2	–	–	1	1	2	1	2	2	–	–	–	–	–	–	–	–	–	c							
AMNH 31772	0	1	2	1	1	1	1	1	1	1	1	1	1	1	1	1	1	1	1	2	1	2	1	–	–	–	–	–	–	–	–	c								
14+18															1	1	–	1	1	2	1	2	2	–	–	–	–	–	–	–	–	c								
AMNH 31781 a	0	2	1	1	1	1	1	1	1	1	1	1	1	1	1	1	1	1	1	1	2	1	2	2	–	–	–	–	–	–	–	c								
14+18															2	–	–	1	1	2	1	2	2	–	–	–	–	–	–	–	–	c								
b	0	2	1	1	1	1	1	1	1	1	1	1	1	1	1	1	1	1	1	2	1	2	2	–	–	–	–	–	–	–	–	c								
14+18															1	1	1	1	1	1	2	2	–	–	–	–	–	–	–	–	–	c								
c	0	2	1	1	1	1	1	1	1	1	1	1	1	1	1	1	1	1	1	2	1	2	1	1	–	–	–	–	–	–	–	c								
14+18															1	1	1	1	1	2	1	2	1	–	–	–	–	–	–	–	–	c								

Species, Museum, Catalog number / ID<br>Vertebral count	1	2	3	4	5	6	7	8	9	10	11	12	13	14	15	16	17	18	19	20	21	22	23	24	25	26	27	28	29	30	31	32	33	34	35	36	37	38	39	40
AMNH 31831	0	2	1	1	1	1	1	1	1	1	1	1	1	1	1	1	1	1	1	2	1	2	1	–	–	–	–	–	–	–	c									
14+17															1	1	1	1	1	1	2	2	–	–	–	–	–	–	–	–	c									
Dimidiochromis compressiceps AMNH 11722 c	0	2	1	1	1	1	1	1	1	1	1	1	1	1	1	1	1	1	2	1	2	2	1	–	–	–	–	–	–	–	–	c								
14+18															1	1	1	2	2	1	2	2	–	–	–	–	–	–	–	–	–	c								
AMNH 11737 a	0	2	1	1	1	1	1	1	1	1	1	1	1	1	1	1	1	1	2	1	1	2	2	–	–	–	–	–	–	–	–	c								
13+19														1	–	1	1	2	1	2	2	2	–	–	–	–	–	–	–	–	–	c								
AMNH 17826	0	2	1	1	1	1	1	1	1	1	1	1	1	1	1	1	1	1	2	1	2	2	–	–	–	–	–	–	–	–	c									
13+18														1	1	1	1	1	2	2	2	1	–	–	–	–	–	–	–	–	c									
AMNH 31783 a	0	2	1	1	1	1	1	1	1	1	1	1	1	1	1	1	1	1	1	2	2	2	–	–	–	–	–	–	–	–	–	c								
14+18															1	1	1	1	2	1	2	2	–	–	–	–	–	–	–	–	–	c								
b	0	2	1	1	1	1	1	1	1	1	1	1	1	1	1	1	1	1	1	2	1	2	2	–	–	–	–	–	–	–	–	c								
14+18															1	1	1	2	1	2	1	3	–	–	–	–	–	–	–	–	–	c								
c	0	2	1	1	1	1	1	1	1	1	1	1	1	1	1	1	1	1	2	1	2	2	–	–	–	–	–	–	–	–	–	c								
13+19														1	–	1	1	1	1	2	2	2	–	–	–	–	–	–	–	–	–	c								
d	0	2	1	1	1	1	1	1	1	1	1	1	1	1	1	1	1	1	1	2	1	3	–	–	–	–	–	–	–	–	–	c								
14+18															1	1	1	2	1	2	2	1	–	–	–	–	–	–	–	–	–	c								
AMNH 31785 a	0	2	1	1	1	1	1	1	1	1	1	1	1	1	1	1	1	1	1	1	2	2	2	–	–	–	–	–	–	–	–	c								
14+18															1	1	1	1	2	2	1	3	–	–	–	–	–	–	–	–	–	c								
b	0	2	1	1	1	1	1	1	1	1	1	1	1	1	1	1	1	1	2	1	2	2	–	–	–	–	–	–	–	–	–	c								
13+19														2	–	–	2	1	2	1	2	2	–	–	–	–	–	–	–	–	–	c								
c	0	2	1	1	1	1	1	1	1	1	1	1	1	1	1	1	1	1	1	2	1	2	2	–	–	–	–	–	–	–	–	c								
14+18															1	1	1	1	2	2	1	3	1	–	–	–	–	–	–	–	–	c								
d	0	2	1	1	1	1	1	1	1	1	1	1	1	1	1	1	1	1	1	2	1	2	2	–	–	–	–	–	–	–	–	c								
14+18															1	2	–	1	2	1	2	3	–	–	–	–	–	–	–	–	–	c								
AMNH 31835 b	0	2	1	1	1	1	1	1	1	1	1	1	1	1	1	1	1	1	1	1	2	2	2	–	–	–	–	–	–	–	–	–	c							
14+19															1	1	1	1	2	1	2	2	1	–	–	–	–	–	–	–	–	–	c							
Dimidiochromis kiwinge NHMUK 1935.6.14.1079–1084	0	2	1	1	1	1	1	1	1	1	1	1	1	1	1	1	1	1	1	1	2	1	2	2	–	–	–	–	–	–	–	–	c							
15+18																1	1	1	1	2	2	2	2	–	–	–	–	–	–	–	–	–	c							
	0	2	1	1	1	1	1	1	1	1	1	1	1	1	1	1	1	1	1	1	2	1	2	2	–	–	–	–	–	–	–	–	c							
15+18																1	1	1	2	1	2	2	1	–	–	–	–	–	–	–	–	–	c							
	0	0-1	1	1	1	1	1	1	1	1	1	1	1	1	1	1	1	1	1	2	1	2	2	1	–	–	–	–	–	–	–	–	c							
15+18																			4*	–*	2	1	2	3?	1?	–	–	–	–	–	–	–	c							
Docimodus evelynae NHMUK 1975.3.10.1 holotype	0	1	2	1	1	1	1	1	1	1	1	1	1	1	1	1	1	1	1	1	2	1	2	2	–	–	–	–	–	–	–	–	c							
15+18																1	1	1	1	2	1	2	2	–	–	–	–	–	–	–	–	–	c							
USNM 216603 paratype	0	2	1	1	1	1	1	1	1	1	1	1	1	1	1	1	1	1	1	1	1	2	2	1	2	–	–	–	–	–	–	–	c							
15+18																1	1	1	1	1	1	2	2	1	–	–	–	–	–	–	–	–	c							
Docimodus johnstoni USNM 330496	0	2	1	1	1	1	1	1	1	1	1	1	1	1	1	1	1	1	2	1	2	2	1	–	–	–	–	–	–	–	c									
14+18															1	1	1	1	1	2	2	1	–	–	–	–	–	–	–	–	c									
	0	0-2	1	1	1	1	1	1	1	1	1	1	1	1	1	1	1	1	2	1	2	2	–	–	–	–	–	–	–	–	c									
14+18															1	1	1	1	2	1	2	2	–	–	–	–	–	–	–	–	c									
CUMV 86290	0	2	1	1	1	1	1	1	1	1	1	1	1	1	1	1	1	1	1	1	2	1	2	1	–	–	–	–	–	–	–	–	c							
14+19															1	1	–	1	2	1	2	2	–	–	–	–	–	–	–	–	–	–	c							
Exochochromis anagenys PSU 13375 fish 1	0	2	1	1	1	1	1	1	1	1	1	1	1	1	1	1	1	1	1	2	1	2	2	–	–	–	–	–	–	–	–	c								
14+18															2	–	1	1	1	2	2	2	–	–	–	–	–	–	–	–	–	c								
fish 2	0	1	2	1	1	1	1	1	1	1	1	1	1	1	1	1	1	1	1	2	1	2	2	–	–	–	–	–	–	–	–	c								
14+18															1	1	1	1	1	2	2	2	–	–	–	–	–	–	–	–	–	c								
PSU 13376 fish 1	0	2	2	–	2	1	–	1	1	1	1	1	1	1	1	1	1	1	1	2	1	2	3	–	–	–	–	–	–	–	–	c								
14+18															1	1	1	1	1	2	1	3	–	–	–	–	–	–	–	–	–	c								
fish 2	0	2	1	1	1	1	1	1	1	1	1	1	1	1	1	1	1	1	2	1	2	2	1	–	–	–	–	–	–	–	–	c								
14+18															1	1	1	1	1	1	2	3	–	–	–	–	–	–	–	–	–	c								
USNM 304657 Holotype	0	2	1	1	1	1	1	1	1	1	1	1	1	1	1	1	1	1	1	1	2	2	2	1	–	–	–	–	–	–	–	–	c							
15+18																1	1	1	1	2	1	3	–	–	–	–	–	–	–	–	–	–	c							
USNM 304659 Paratype	0	2	1	1	1	1	1	1	1	1	1	1	1	1	1	1	1	1	1	2	1	2	3	–	–	–	–	–	–	–	–	c								
14+18															2	–	–	2	1	1	2	3	–	–	–	–	–	–	–	–	–	c								
YPM 023205	0	2	1	1	1	1	1	1	1	1	1	1	1	1	1	1	1	1	1	2	2	2	2	–	–	–	–	–	–	–	–	c								
13+19														1	–	1	1	1	2	2	2	1	–	–	–	–	–	–	–	–	–	c								
Fossorochromis rostratus YPM 027097	0	2	1	1	1	1	1	1	1	1	1	1	1	1	1	1	1	1	1	2	1	2	2	–	–	–	–	–	–	–	–	c								
14+18															2	–	1	–	2	1	2	1	2	–	–	–	–	–	–	–	–	c								
Hemitaeniochromis urotaenia YPM 007803	0	2	1	1	1	1	1	1	1	1	1	1	1	1	1	1	1	1	2	1	2	2	–	–	–	–	–	–	–	–	c									
14+17															1	1	1	1	1	2	2	1	–	–	–	–	–	–	–	–	c									
Hemitilapia oxyrhynchus AMNH 31880 a	0	2	1	1	1	1	1	1	1	1	1	1	1	1	1	1	1	1	1	2	1	2	2	–	–	–	–	–	–	–	c									
14+17															1	1	1	1	1	2	2	2	–	–	–	–	–	–	–	–	c									
b	0	2	1	1	1	1	1	1	1	1	1	1	1	1	1	1	1	1	2	1	2	3	–	–	–	–	–	–	–	–	c									
14+17															2	–	1	1	2	1	2	2	–	–	–	–	–	–	–	–	c									
c	0	2	1	1	1	1	1	1	1	1	1	1	1	1	1	1	1	1	1	2	1	2	2	–	–	–	–	–	–	–	c									
14+17															1	–	1	1	1	2	2	2	–	–	–	–	–	–	–	–	c									
d	0	2	1	1	1	1	1	1	1	1	1	1	1	1	1	1	1	1	2	1	2	2	1	–	–	–	–	–	–	–	c									
14+17															1	1	1	1	1	2	2	2	–	–	–	–	–	–	–	–	c									

Species, Museum, Catalog number / ID<br>Vertebral count	1	2	3	4	5	6	7	8	9	10	11	12	13	14	15	16	17	18	19	20	21	22	23	24	25	26	27	28	29	30	31	32	33	34	35	36	37	38	39	40
e	0	2	1	1	1	1	1	1	1	1	1	1	1	1	1	1	1	1	2	1	1	2	2	1	–	–	–	–	–	–	–	c								
15+17																1	1	1	1	2	1	2	2	–	–	–	–	–	–	–	–	c								
f	0	2	1	1	1	1	1	1	1	1	1	1	1	1	1	1	1	1	1	2	1	2	2	–	–	–	–	–	–	–	c									
14+17															1	–	1	1	1	2	2	1	2	–	–	–	–	–	–	–	c									
NHMUK 1972.12.18:45-48 a	0	2	1	1	1	1	1	1	1	1	1	1	1	1	1	1	1	2	1	2	2	1	–	–	–	–	–	–	–	c										
14+16															1	1	1	1	2	2	2	1	–	–	–	–	–	–	–	c										
b	0	2	1	1	1	1	1	1	1	1	1	1	1	1	1	1	1	1	1	2	1	3	–	–	–	–	–	–	–	–	c									
14+17															1	1	–	1	2	2	1	3	–	–	–	–	–	–	–	–	c									
c	0	2	1	1	1	1	1	1	1	1	1	1	1	1	1	1	1	1	2	1	2	2	1	–	–	–	–	–	–	–	c									
14+17															1	2	–	1	1	2	1	3	–	–	–	–	–	–	–	–	c									
d	0	2	1	1	1	1	1	1	1	1	1	1	1	1	1	1	1	1	1	2	1	2	2	–	–	–	–	–	–	–	–	c								
15+17																2	–	1	1	2	2	2	–	–	–	–	–	–	–	–	–	c								
Lethrinops gossei USNM 210694 Holotype	0	2	1	1	1	1	1	1	1	1	1	1	1	1	1	1	1	1	1	2	2	2	–	–	–	–	–	–	–	–	c									
13+18														2	–	–	1	2	1	2	2	–	–	–	–	–	–	–	–	–	c									
USNM 210715 Paratype	0	2	1	1	1	1	1	1	1	1	1	1	1	1	1	1	1	2	1	1	3	1	–	–	–	–	–	–	–	–	–	c								
14+18															1	1	1	1	1	2	2	1	–	–	–	–	–	–	–	–	–	c								
Lethrinops lethrinus AMNH 58000	0	2	1	1	1	1	1	1	1	1	1	1	1	1	1	1	1	2	1	1	2	2	–	–	–	–	–	–	–	c										
13+17														1	1	–	1	2	1	2	2	–	–	–	–	–	–	–	–	c										
	0	2	1	1	1	1	1	1	1	1	1	1	1	1	1	1	1	2	1	2	2	–	–	–	–	–	–	–	–	c										
13+17														1	1	1	1	1	2	2	–	–	–	–	–	–	–	–	–	c										
YPM 014443	0	2	1	1	1	1	1	1	1	1	1	1	1	1	1	1	1	2	1	2	2	–	–	–	–	–	–	–	c											
13+16														2	–	1	1	2	2	2	–	–	–	–	–	–	–	–	c											
Lethrinops polli USNM 210695 Holotype	0	2	1	1	1	1	1	1	1	1	1	1	1	1	1	1	1	1	2	1	3	1	–	–	–	–	–	–	–	–	c									
13+18														1	–	1	1	2	1	2	2	–	–	–	–	–	–	–	–	–	c									
Lichnochromis acuticeps YPM 014590	0	2	1	1	1	1	1	1	1	1	1	1	1	1	1	1	1	2	1	2	1	2	–	–	–	–	–	–	–	–	c									
14+17															2	1	–	1	1	2	2	1	–	–	–	–	–	–	–	–	c									
Mchenga cyclicos USNM 324569 a Paratype	0	2	1	1	1	1	1	1	1	1	1	1	1	1	1	1	1	1	1	2	1	2	2	–	–	–	–	–	–	–	–	–	c							
15+18																1	1	1	1	1	2	2	–	–	–	–	–	–	–	–	–	–	c							
Mchenga inornata NHMUK 1908.10.27.101 Lectotype	0	2	1	1	1	1	1	1	1	1	1	1	1	1	1	1	1	1	1	2	1	1	2	3	–	–	–	–	–	–	–	–	c							
14+19															1	–	1	1	1	1	1	2	2	–	–	–	–	–	–	–	–	–	c							
NHMUK 1908.10.27.102 paralectotype	0	2	1	1	1	1	1	1	1	1	1	1	1	1	1	1	1	1	1	2	1	2	1	2	–	–	–	–	–	–	–	–	c							
15+18																1	1	1	1	1	2	1	2	–	–	–	–	–	–	–	–	–	c							
Mchenga sp. AMNH 31855 a	0	2	1	1	1	1	1	1	1	1	1	1	1	1	1	1	1	1	1	2	1	2	1	2	–	–	–	–	–	–	–	–	c							
15+18																1	1	1	1	1	1	2	2	–	–	–	–	–	–	–	–	–	c							
b	0	1	2	1	1	1	1	1	1	1	1	1	1	1	1	1	1	1	1	1	2	1	3	1	–	–	–	–	–	–	–	–	c							
15+18																1	1	1	1	1	2	2	2	–	–	–	–	–	–	–	–	–	c							
c	0	1	2	1	1	1	1	1	1	1	1	1	1	1	1	1	1	1	1	1	2	1	2	2	–	–	–	–	–	–	–	–	–	c						
16+18																	2	1	1	1	1	2	2	–	–	–	–	–	–	–	–	–	–	c						
d	0	2	1	1	1	1	1	1	1	1	1	1	1	1	1	1	1	1	1	1	2	1	2	2	–	–	–	–	–	–	–	–	c							
16+17																	2	1	1	2	1	2	2	–	–	–	–	–	–	–	–	–	c							
e	0	2	1	1	1	1	1	1	1	1	1	1	1	1	1	1	1	1	1	2	1	1	2	2	–	–	–	–	–	–	–	–	–	c						
16+18																	2	1	1	1	1	2	2	1	–	–	–	–	–	–	–	–	–	c						
f	0	2	1	1	1	1	1	1	1	1	1	1	1	1	1	1	1	1	1	1	2	1	3	1	–	–	–	–	–	–	–	–	c							
16+17																	2	1	1	1	2	1	3	–	–	–	–	–	–	–	–	–	c							
g	0	1	2	1	1	1	1	1	1	1	1	1	1	1	1	1	1	1	1	2	1	2	2	1	–	–	–	–	–	–	–	–	c							
16+17																	2	1	1	1	2	2	2*	1*	–	–	–	–	–	–	–	–	c							
h	0	2	1	1	1	1	1	1	1	1	1	1	1	1	1	1	1	1	1	2	1	2	2	2	–	–	–	–	–	–	–	–	c							
15+18																1	1	1	1	1	2	1	3	–	–	–	–	–	–	–	–	–	c							
i	0	1	2	1	1	1	1	1	1	1	1	1	1	1	1	1	1	1	1	1	2	1	2	2	–	–	–	–	–	–	–	–	c							
16+17																	2	1	1	1	1	2	3	–	–	–	–	–	–	–	–	–	c							
j	0	2	1	1	1	1	1	1	1	1	1	1	1	1	1	1	1	1	1	1	2	1	2	2	–	–	–	–	–	–	–	–	–	c						
16+18																	2	1	1	1	2	1	2	2	–	–	–	–	–	–	–	–	–	c						
k	0	2	1	1	1	1	1	1	1	1	1	1	1	1	1	1	1	1	1	1	2	1	1	3	1	–	–	–	–	–	–	–	–	c						
16+18																	2	–	1	1	2	1	2	2	–	–	–	–	–	–	–	–	–	c						
l	0	2	1	1	1	1	1	1	1	1	1	1	1	1	1	1	1	1	1	1	2	1	2	2	–	–	–	–	–	–	–	–	–	c						
16+18																	2	1	–	2	1	2	3	–	–	–	–	–	–	–	–	–	–	c						
m	0	2	1	1	1	1	1	1	1	1	1	1	1	1	1	1	1	1	1	1	2	1	2	2	1	–	–	–	–	–	–	–	–	c						
16+18																	2	–	1	1	2	1	2	2	–	–	–	–	–	–	–	–	–	c						
n	0	2	1	1	1	1	1	1	1	1	1	1	1	1	1	1	1	1	1	1	2	1	2	2	–	–	–	–	–	–	–	–	–	c						
16+18																	2	1	1	1	2	2	2	–	–	–	–	–	–	–	–	–	–	c						
o	0	2	1	1	1	1	1	1	1	1	1	1	1	1	1	1	1	1	1	1	2	1	2	2	–	–	–	–	–	–	–	–	–	c						
16+18																	2	–	1	2	1	2	2	1	–	–	–	–	–	–	–	–	–	c						
p	0	2	1	1	1	1	1	1	1	1	1	1	1	1	1	1	1	1	1	1	2	1	2	2	1	–	–	–	–	–	–	–	c							
16+17																	2	–	1	2	1	2	2	1	–	–	–	–	–	–	–	–	c							
q	0	2	1	1	1	1	1	1	1	1	1	1	1	1	1	1	1	1	1	1	2	1	2	2	–	–	–	–	–	–	–	–	c							
16+17																	2	1	1	1	2	2	2	1	–	–	–	–	–	–	–	–	c							
r	0	1	2	1	1	1	1	1	1	1	1	1	1	1	1	1	1	1	1	2	1	2	2	1	–	–	–	–	–	–	–	–	c							
16+17																	2	1	1	1	2	2	2	–	–	–	–	–	–	–	–	–	c							

Species, Museum, Catalog number / ID<br>Vertebral count	1	2	3	4	5	6	7	8	9	10	11	12	13	14	15	16	17	18	19	20	21	22	23	24	25	26	27	28	29	30	31	32	33	34	35	36	37	38	39	40
s	0	2	1	1	1	1	1	1	1	1	1	1	1	1	1	1	1	1	1	2	1	2	2	1	–	–	–	–	–	–	–	–	c							
16+17																	2	1	1	1	2	2	2	–	–	–	–	–	–	–	–	–	c							
t	0	2	1	1	1	1	1	1	1	1	1	1	1	1	1	1	1	1	1	1	1	2	2	2	–	–	–	–	–	–	–	–	–	c						
16+18																	2	1	1	1	2	1	3	–	–	–	–	–	–	–	–	–	–	c						
u	0	1	2	1	1	1	1	1	1	1	1	1	1	1	1	1	1	1	1	1	2	1	2	2	–	–	–	–	–	–	–	–	c							
16+17																	1	1	1	1	2	2	2	–	–	–	–	–	–	–	–	–	c							
v	0	2	1	1	1	1	1	1	1	1	1	1	1	1	1	1	1	1	1	1	2	1	2	2	–	–	–	–	–	–	–	c								
16+16																	2	1	1	1	2	2	2	1	–	–	–	–	–	–	–	c								
w	0	1	2	1	1	1	1	1	1	1	1	1	1	1	1	1	1	1	1	1	2	1	2	2	–	–	–	–	–	–	–	–	c							
16+17																	2	1	–	2	1	2	2	1	–	–	–	–	–	–	–	–	c							
Mylochromis formosus NHMUK 1935.6.14:1454-55 Syntypes	0	2	1	1	1	1	1	1	1	1	1	1	1	1	1	1	1	1	1	1	2	1	1	2	2	–	–	–	–	–	–	–	c							
larger = paralectotype 16+17																	1	1	1	1	1	2	1	2	–	–	–	–	–	–	–	–	c							
smaller = Lectotype	0	2	1	1	1	1	1	1	1	1	1	1	1	1	1	1	1	1	1	1	2	1	2	2	1	–	–	–	–	–	–	–	–	c						
15+19																2	–	–	1	1	2	1	2	1	–	–	–	–	–	–	–	–	–	c						
Mylochromis gracilis NHMUK 1935.6.14:1456-58	0	2	1	1	1	1	1	1	1	1	1	1	1	1	1	1	1	1	1	1	2	1	2	2	1	–	–	–	–	–	–	–	–	c						
Smallest=Lectotype 15+19																2	–	–	1	1	2	2	1	2	–	–	–	–	–	–	–	–	–	c						
	0	2	1	1	1	1	1	1	1	1	1	1	1	1	1	1	1	1	1	1	1	2	2	1	–	–	–	–	–	–	–	–	–	–	c					
Paralectotype 15+20																1	–	1	1	1	1	2	1	3	–	–	–	–	–	–	–	–	–	–	c					
	0	2	1	1	1	1	1	1	1	1	1	1	1	1	1	1	1	1	1	1	1	3	1	–	–	–	–	–	–	–	–	–	–	c						
Paralectotype 15+19																1	1	–	1	1	2	1	2	2	–	–	–	–	–	–	–	–	–	c						
Mylochromis guentheri AMNH 31808	0	1	2	1	1	1	1	1	1	1	1	1	1	1	1	1	1	1	1	1	2	2	2	1	–	–	–	–	–	–	–	c								
15+17																1	1	1	1	2	1	2	–	–	–	–	–	–	–	–	–	c								
	0	2	1	1	1	1	1	1	1	1	1	1	1	1	1	1	1	1	1	2	1	2	2	–	–	–	–	–	–	–	c									
15+16																1	1	1	1	2	1	3	–	–	–	–	–	–	–	–	c									
Mylochromis lateristriga AMNH 31770	0	2	1	1	1	1	1	1	1	1	1	1	1	1	1	1	1	1	2	1	2	3*	–	–	–	–	–	–	–	–	c									
14+17															1	1	1	1	2	1	2	2	–	–	–	–	–	–	–	–	c									
	0	2	1	1	1	1	1	1	1	1	1	1	1	1	1	1	1	1	2	1	2	2	1	–	–	–	–	–	–	–	c									
14+17															2	–	1	1	1	2	2	2	–	–	–	–	–	–	–	–	c									
AMNH 31842	0	2	1	1	1	1	1	1	1	1	1	1	1	1	1	1	1	1	2	1	2	2	1	–	–	–	–	–	–	–	c									
14+17															1	1	1	1	1	2	2	2	–	–	–	–	–	–	–	–	c									
	0	2	1	1	1	1	1	1	1	1	1	1	1	1	1	1	1	1	2	1	2	2	1	–	–	–	–	–	–	–	c									
14+17															1	1	1	1	1	2	2	1	–	–	–	–	–	–	–	–	c									
Mylochromis spilostichus NHMUK 1935.6.14:1459 Holotype	0	2	1	1	1	1	1	1	1	1	1	1	1	1	1	1	1	1	1	1	2	1	1	2	2	1	–	–	–	–	–	–	–	–	c					
16+19																	1	1	1	1	1	2	2	2	–	–	–	–	–	–	–	–	–	–	c					
NHMUK 1969.3.11:6	0	2	1	1	1	1	1	1	1	1	1	1	1	1	1	1	1	1	1	1	2	1	2	2	2	–	–	–	–	–	–	–	–	–	c					
15+20																2	–	–	1	1	2	1	2	2	–	–	–	–	–	–	–	–	–	–	c					
AMNH 221934 a	0	2	1	1	1	1	1	1	1	1	1	1	1	1	1	1	1	1	1	1	1	2	1	2	2	–	–	–	–	–	–	–	–	c						
15+19																1	–	1	1	1	2	1	2	2	–	–	–	–	–	–	–	–	–	c						
b	0	2	1	1	1	1	1	1	1	1	1	1	1	1	1	1	1	1	1	1	2	1	2	2	1	–	–	–	–	–	–	–	–	c						
15+19																1	1	–	1	1	1	2	2	2	–	–	–	–	–	–	–	–	–	c						
c	0	2	1	1	1	1	1	1	1	1	1	1	1	1	1	1	1	1	1	1	2	1	2	1	2	–	–	–	–	–	–	–	–	c						
15+19																1*	1*	–	1	1	2	1	2	2	–	–	–	–	–	–	–	–	–	c						
d	0	2	1	1	1	1	1	1	1	1	1	1	1	1	1	1	1	1	1	1	1	2	1	2	2	1	–	–	–	–	–	–	–	c						
15+19																1	–	1	1	1	2	1	1	3	–	–	–	–	–	–	–	–	–	c						
e	0	2	1	1	1	1	1	1	1	1	1	1	1	1	1	1	1	1	1	1	1	2	1	2	2	1	–	–	–	–	–	–	–	–	c					
16+19																	1	1	1	1	1	2	1	3	–	–	–	–	–	–	–	–	–	–	c					
f	0	2	1	1	1	1	1	1	1	1	1	1	1	1	1	1	1	1	1	1	2	1	2	1	2	–	–	–	–	–	–	–	–	–	c					
16+19																	1	1	1	1	1	1	2	2	1	–	–	–	–	–	–	–	–	–	c					
Mylochromis subocularis USNM 330657 vertical on film	0	2	1	1	1	1	1	1	1	1	1	1	1	1	1	1	1	1	2	1	2	2	1	–	–	–	–	–	–	–	–	c								
14+18															1	–	1	1	1	1	2	2	–	–	–	–	–	–	–	–	–	c								
inverted on film	0	2	1	1	1	1	1	1	1	1	1	1	1	1	1	1	1	1	2	1	2	2	–	–	–	–	–	–	–	–	c									
14+17															1	1	1	1	1	1	2	1	–	–	–	–	–	–	–	–	c									
Naevochromis chrysogaster AMNH 31782	0	2	1	1	1	1	1	1	1	1	1	1	1	1	1	1	1	1	1	2	2	2	–	–	–	–	–	–	–	–	c									
14+17															1	1	1	1	2	2	2	–	–	–	–	–	–	–	–	–	c									
AMNH 31793	0	2	1	1	1	1	1	1	1	1	1	1	1	1	1	1	1	1	1	2	1	3	–	–	–	–	–	–	–	–	c									
14+17															1	1	1	1	1	2	2	1	–	–	–	–	–	–	–	–	c									
NHMUK 1935.6.14:1640-1	0	2	1	1	1	1	1	1	1	1	1	1	1	1	1	1	1	1	1	2	2	2	–	–	–	–	–	–	–	–	c									
Larger=Lectotype 14+17															1	1	1	1	2	1	3	–	–	–	–	–	–	–	–	–	c									
	0	2	1	1	1	1	1	1	1	1	1	1	1	1	1	1	1	1	1	2	1	2	2	–	–	–	–	–	–	–	c									
paralecto 14+17															1	1	1	1	1	2	1	3	–	–	–	–	–	–	–	–	c									
NHMUK 1935.6.14:1642	0	2	1	1	1	1	1	1	1	1	1	1	1	1	1	1	1	1	1	2	1	2	2*	–	–	–	–	–	–	–	c									
paralecto 14+17															2?	–?	–	1	2	1	2	2	1?	–	–	–	–	–	–	–	c									
Nimbochromis fuscotaeniatus AMNH 31805	0	2	1	1	1	1	1	1	1	1	1	1	1	1	1	1	1	1	1	2	1	2	2	–	–	–	–	–	–	–	–	c								
14+18															1	1	1	1	1	2	1	3	–	–	–	–	–	–	–	–	–	c								
AMNH 31856	0	1	2	1	1	1	1	1	1	1	1	1	1	1	1	1	1	1	2	1	2	2	–	–	–	–	–	–	–	c										
14+16															1	1	1	1	2	2	2	–	–	–	–	–	–	–	–	c										
Nimbochromis livingstonii AMNH 31777	0	2	1	1	1	1	1	1	1	1	1	1	1	1	1	1	1	1	1	2	1	3	1	–	–	–	–	–	–	–	c									
14+17															1	2	–	1	2	1	2	2	–	–	–	–	–	–	–	–	c									

Species, Museum, Catalog number / ID Vertebral count	1	2	3	4	5	6	7	8	9	10	11	12	13	14	15	16	17	18	19	20	21	22	23	24	25	26	27	28	29	30	31	32	33	34	35	36	37	38	39	40
AMNH 31803 a	0	2	1	1	1	1	1	1	1	1	1	1	1	1	1	1	1	1	1	2	1	3	–	–	–	–	–	–	–	–	c									
14+17															1	1	1	1	2	1	3	–	–	–	–	–	–	–	–	–	c									
b	0	2	1	1	1	1	1	1	1	1	1	1	1	1	1	1	1	1	1	2	2	2	–	–	–	–	–	–	–	–	c									
14+17															1	1	1	1	2	1	3	–	–	–	–	–	–	–	–	–	c									
c	0	2	1	1	1	1	1	1	1	1	1	1	1	1	1	1	1	1	1	2	2	2	–	–	–	–	–	–	–	–	c									
14+17															1	1	1	1	2	1	2	2	–	–	–	–	–	–	–	–	c									
d	0	2	1	1	1	1	1	1	1	1	1	1	1	1	1	1	1	1	1	1	2	2	2	–	–	–	–	–	–	–	–	c								
15+17																1	1	1	2	1	2	2	–	–	–	–	–	–	–	–	–	c								
e	0	2	1	1	1	1	1	1	1	1	1	1	1	1	1	1	1	1	1	2	1	3	–	–	–	–	–	–	–	–	c									
14+17															1	1	1	1	2	2	2	–	–	–	–	–	–	–	–	–	c									
Nimbochromis venustus AMNH 221885 a	0	2	1	1	1	1	1	1	1	1	1	1	1	1	1	1	1	1	1	2	2	2	–	–	–	–	–	–	–	–	c									
14+17															1	1	1	1	2	2	2	1	–	–	–	–	–	–	–	–	c									
b	0	2	1	1	1	1	1	1	1	1	1	1	1	1	1	1	1	1	1	2	1	2	2	–	–	–	–	–	–	–	c									
14+17															2	–	–	1	1	2	2	2*	1*	–	–	–	–	–	–	–	c									
c	0	2	1	1	1	1	1	1	1	1	1	1	1	1	1	1	1	1	1	2	1	2	2	–	–	–	–	–	–	–	c									
14+17															1	1	1	1	2	1	2	2	–	–	–	–	–	–	–	–	c									
d	0	1	2	1	1	1	1	1	1	1	1	1	1	1	1	1	1	1	1	2	2	2	–	–	–	–	–	–	–	–	c									
14+17															1	1	1	1	1	2	2	2	–	–	–	–	–	–	–	–	c									
Nyassachromis leuciscus NHMUK 1921.9.6.188 Lectotype	0	2	1	1	1	1	1	1	1	1	1	1	1	1	1	1	1	1	1	2	1	2	2	1	–	–	–	–	–	–	–	c								
14+18															1	–	1	1	1	1	2	2	1	–	–	–	–	–	–	–	–	c								
USNM 266852 1	0	2	1	1	1	1	1	1	1	1	1	1	1	1	1	1	1	1	1	2	1	2	2	–	–	–	–	–	–	–	–	c								
14+18															1	1	–	1	2	1	2	2	–	–	–	–	–	–	–	–	–	c								
2	0	2	1	1	1	1	1	1	1	1	1	1	1	1	1	1	1	1	1	2	1	3	1	–	–	–	–	–	–	–	–	c								
14+18															1	1	1	1	1	2	2	2	–	–	–	–	–	–	–	–	–	c								
3	0	2	1	1	1	1	1	1	1	1	1	1	1	1	1	1	1	1	2	1	2	1	2	–	–	–	–	–	–	–	–	c								
14+18															1	1	1	1	1	1	2	3	–	–	–	–	–	–	–	–	–	c								
4	0	2	1	1	1	1	1	1	1	1	1	1	1	1	1	1	1	1	2	1	2	1	2	–	–	–	–	–	–	–	–	c								
14+18															1	1	1	1	1	2	2	2	–	–	–	–	–	–	–	–	–	c								
5	0	2	1	1	1	1	1	1	1	1	1	1	1	1	1	1	1	1	1	2	1	1	2	2	–	–	–	–	–	–	–	–	c							
14+19															1	1	1	–	2	1	2	1	2	–	–	–	–	–	–	–	–	–	c							
Nyassachromis microcephalus NHMUK 1935.6.14.873-4	0	2	1	1	1	1	1	1	1	1	1	1	1	1	1	1	1	1	1	1	2	1	2	2	1	–	–	–	–	–	–	–	c							
Lectotype & paralectotype 16+17																	1	1	1	1	1	2	2	2	–	–	–	–	–	–	–	–	c							
	0	2	1	1	1	1	1	1	1	1	1	1	1	1	1	1	1	1	1	2	1	2	2	1	–	–	–	–	–	–	–	–	c							
(lower, larger) 15+18																1	1	–	2	1	1	2	2	–	–	–	–	–	–	–	–	–	c							
Nyassachromis nigritaeniatus USNM 266863 1	0	2	1	1	1	1	1	1	1	1	1	1	1	1	1	1	1	1	1	1	2	1	2	2	–	–	–	–	–	–	–	–	c							
16+17																	2	–	1	1	2	1	3	–	–	–	–	–	–	–	–	–	c							
2	0	2	1	1	1	1	1	1	1	1	1	1	1	1	1	1	1	1	1	1	2	1	4	–	–	–	–	–	–	–	–	–	c							
15+18																2	–	1	1	1	2	2	1	–	–	–	–	–	–	–	–	–	c							
3	0	2	1	1	1	1	1	1	1	1	1	1	1	1	1	1	1	1	1	2	1	2	3	–	–	–	–	–	–	–	–	–	c							
15+18																1	1	1	1	1	1	2	2	–	–	–	–	–	–	–	–	–	c							
4	0	2	1	1	1	1	1	1	1	1	1	1	1	1	1	1	1	1	1	2	1	2	3	–	–	–	–	–	–	–	–	c								
15+17																1	1	1	1	2	1	2	1	–	–	–	–	–	–	–	–	c								
Nyassachromis purpurans NHMUK1935.6.14.895-902 syntype	0	2	1	1	1	1	1	1	1	1	1	1	1	1	1	1	1	1	1	2	1	2	2	1	–	–	–	–	–	–	–	–	c							
15+18																1	1	1	1	1	1	2	2	–	–	–	–	–	–	–	–	–	c							
Otopharynx argyrosoma NHMUK 1908.10.27:99 Holotype	0	2	1	1	1	1	1	1	1	1	1	1	1	1	1	1	1	1	1	2	1	2	2	–	–	–	–	–	–	–	–	c								
15+17																1	1	1	1	1	2	2	1	–	–	–	–	–	–	–	–	c								
uncat.? [cat # TBD, not AMNH, YPM, USNM]	0	2	1	1	1	1	1	1	1	1	1	1	1	1	1	1	1	1	1	2	1	2	3	–	–	–	–	–	–	–	–	–	c							
15+18																1	1	1	1	1	1	2	2	–	–	–	–	–	–	–	–	–	c							
	0	2	1	1	1	1	1	1	1	1	1	1	1	1	1	1	1	1	1	1	2	1	2	2	–	–	–	–	–	–	–	–	c							
15+18																1	1	1	1	1	2	1	2	–	–	–	–	–	–	–	–	–	c							
Otopharynx decorus NHMUK 1935.6.14:1651 Lectotype	0	2	1	1	1	1	1	1	1	1	1	1	1	1	1	1	1	1	1	1	1	2	1	2	3	–	–	–	–	–	–	–	–	–	c					
16+19																	1	1	1	1	1	1	2	2	1	–	–	–	–	–	–	–	–	–	c					
NHMUK 1935.6.14:1652-54 Paralectotypes	0	2	1	1	1	1	1	1	1	1	1	1	1	1	1	1	1	1	1	1	1	2	1	2	2	–	–	–	–	–	–	–	–	c						
16+18																	1	1	1	1	1	2	1	2	–	–	–	–	–	–	–	–	–	c						
	0	2	1	1	1	1	1	1	1	1	1	1	1	1	1	1	1	1	1	1	2	1	1	2	2	–	–	–	–	–	–	–	–	c						
16+18																	1	1	1	1	1	2	1	3	–	–	–	–	–	–	–	–	–	c						
	0	2	1	1	1	1	1	1	1	1	1	1	1	1	1	1	1	1	1	2	1	1	2	2	1	–	–	–	–	–	–	–	–	c						
16+18																	2	–	1	1	2	1	2	1	–	–	–	–	–	–	–	–	–	c						
NHMUK 1935.6.14:1655-1656 Paralectotypes	0	2	1	1	1	1	1	1	1	1	1	1	1	1	1	1	1	1	1	1	2	1	2	1	3	–	–	–	–	–	–	–	–	–	c					
16+19																	1	1	1	1	1	2	1	2*	1*	–	–	–	–	–	–	–	–	–	c					
	0	2	1	1	1	1	1	1	1	1	1	1	1	1	1	1	1	1	1	1	1	2	1	2	2?	–	–	–	–	–	–	–	–	–	c					
16+19																	1	1	1	1	1	1	2	2	–?	–	–	–	–	–	–	–	–	–	c					
Otopharynx cf. *heterodon* AMNH 31886 a	0	2	1	1	1	1	1	1	1	1	1	1	1	1	1	1	1	1	1	2	1	2	2	–	–	–	–	–	–	–	c									
14+17															2	–	–	1	1	2	2	2	–	–	–	–	–	–	–	–	c									
b	0	2	1	1	1	1	1	1	1	1	1	1	1	1	1	1	1	1	1	2	1	2	2	–	–	–	–	–	–	–	c									
14+17															1	–	1	1	1	2	1	3	–	–	–	–	–	–	–	–	c									
c	0	2	1	1	1	1	1	1	1	1	1	1	1	1	1	1	1	1	1	2	1	2	2	–	–	–	–	–	–	–	c									
14+17															1	1	–	1	2	1	2	1	–	–	–	–	–	–	–	–	c									

Species, Museum, Catalog number / ID<br>Vertebral count	1	2	3	4	5	6	7	8	9	10	11	12	13	14	15	16	17	18	19	20	21	22	23	24	25	26	27	28	29	30	31	32	33	34	35	36	37	38	39	40
d	0	2	1	1	1	1	1	1	1	1	1	1	1	1	1	1	1	1	1	2	1	2	2	–	–	–	–	–	–	–	c									
14+17															1	1	–	1	1	2	1	3	–	–	–	–	–	–	–	–	c									
e	0	2	1	1	1	1	1	1	1	1	1	1	1	1	1	1	1	1	1	1	2	1	3	1	–	–	–	–	–	–	–	c								
15+17																1	1	–	1	2	1	2	1	–	–	–	–	–	–	–	–	c								
f	0	2	1	1	1	1	1	1	1	1	1	1	1	1	1	1	1	1	1	2	1	2	2	–	–	–	–	–	–	–	c									
14+17															1	–	1	1	–	2	1	3	–	–	–	–	–	–	–	–	c									
g	0	2	1	1	1	1	1	1	1	1	1	1	1	1	1	1	1	1	2	1	1	2	2	–	–	–	–	–	–	–	c									
14+17															2	–	–	1	1	1	2	2	–	–	–	–	–	–	–	–	c									
h	0	2	1	1	1	1	1	1	1	1	1	1	1	1	1	1	1	1	1	1	2	2	2	–	–	–	–	–	–	–	c									
14+17															2	–	–	1	1	1	2	2	–	–	–	–	–	–	–	–	c									
i	0	2	1	1	1	1	1	1	1	1	1	1	1	1	1	1	1	1	1	2	1	3	1	–	–	–	–	–	–	–	c									
14+17															1	–	1	1	1	2	2	1	–	–	–	–	–	–	–	–	c									
j	0	2	1	1	1	1	1	1	1	1	1	1	1	1	1	1	1	1	1	1	2	2	2	–	–	–	–	–	–	–	c									
14+17															1	1	–	1	1	1	2	2	1	–	–	–	–	–	–	–	c									
k	0	2	1	1	1	1	1	1	1	1	1	1	1	1	1	1	1	1	1	2	1	2	2	–	–	–	–	–	–	–	c									
14+17															2	–	–	1	2	1	2	1	–	–	–	–	–	–	–	–	c									
l	0	2	1	1	1	1	1	1	1	1	1	1	1	1	1	1	1	1	1	2	1	2	3	–	–	–	–	–	–	–	–	c								
14+18															1	–	1	1	1	1	2	3	–	–	–	–	–	–	–	–	–	c								
m	0	2	1	1	1	1	1	1	1	1	1	1	1	1	1	1	1	1	1	2	2	3	–	–	–	–	–	–	–	–	c									
14+17															1	1	1	1	1	1	2	1	–	–	–	–	–	–	–	–	c									
n	0	2	1	1	1	1	1	1	1	1	1	1	1	1	1	1	1	1	1	2	1	2	3	–	–	–	–	–	–	–	c									
14+17															1	–	1	1	1	1	2	3	–	–	–	–	–	–	–	–	c									
o	0	2	1	1	1	1	1	1	1	1	1	1	1	1	1	1	1	1	1	2	1	2	2	–	–	–	–	–	–	–	c									
14+17															2	–	–	1	1	2	2	1	–	–	–	–	–	–	–	–	c									
p	0	2	1	1	1	1	1	1	1	1	1	1	1	1	1	1	1	1	2	1	2	3	–	–	–	–	–	–	–	–	c									
14+17															1	–	1	1	1	2	2	1	–	–	–	–	–	–	–	–	c									
q	0	2	1	1	1	1	1	1	1	1	1	1	1	1	1	1	1	1	1	2	1	2	2	–	–	–	–	–	–	–	c									
14+17															1	–	1	1	1	1	2	2	–	–	–	–	–	–	–	–	c									
r	0	2	1	1	1	1	1	1	1	1	1	1	1	1	1	1	1	1	1	2	1	2	2	–	–	–	–	–	–	–	c									
14+17															1	1	–	1	1	2	2	2	–	–	–	–	–	–	–	–	c									
s	0	2	1	1	1	1	1	1	1	1	1	1	1	1	1	1	1	1	1	2	1	2	3	–	–	–	–	–	–	–	c									
14+17															1	–	1	1	1	1	2	2	1	–	–	–	–	–	–	–	c									
Otopharynx heterodon NHMUK 1935.6.14:1584-5 Syntypes	0	2	1	1	1	1	1	1	1	1	1	1	1	1	1	1	1	1	1	1	2	1	3	2	–	–	–	–	–	–	c									
15+16																1	1	1	1	1	2	2	1	–	–	–	–	–	–	–	c									
Register number of correct lectotype still TBD	0	2	1	1	1	1	1	1	1	1	1	1	1	1	1	1	1	1	1	2	1	2	3	–	–	–	–	–	–	–	c									
15+16																1	1	1	1	1	2	2	1	–	–	–	–	–	–	–	c									
NHMUK 1935.6.14:1586 Syntype	0	2	1	1	1	1	1	1	1	1	1	1	1	1	1	1	1	1	1	1	2	2	2	–	–	–	–	–	–	–	–	c								
15+17																1	1	–	1	2	1	2	1	–	–	–	–	–	–	–	–	c								
NHMUK 1935.6.14:1587-89 Syntypes	0	2	1	1	1	1	1	1	1	1	1	1	1	1	1	1	1	1	1	2	1	2	2	–	–	–	–	–	–	–	c									
15+16																1	1	1	1	1	2	2	–	–	–	–	–	–	–	–	c									
	0	2	1	1	1	1	1	1	1	1	1	1	1	1	1	1	1	1	1	2	1	2	2	–	–	–	–	–	–	c										
15+15																2	–	1	1	2	1	2	–	–	–	–	–	–	–	c										
	0	2	1	1	1	1	1	1	1	1	1	1	1	1	1	1	1	1	1	2	1	2	2	–	–	–	–	–	–	–	c									
15+16																1	1	1	1	1	2	2	–	–	–	–	–	–	–	–	c									
	0	2	1	1	1	1	1	1	1	1	1	1	1	1	1	1	1	1	1	2	1	2	2	–	–	–	–	–	–	–	c									
15+16																1	1	1	–	2	1	3	–	–	–	–	–	–	–	–	c									
	0	2	1	1	1	1	1	1	1	1	1	1	1	1	1	1	1	1	2	1	2	2	1	–	–	–	–	–	–	–	c									
15+16																1	1	1	1	1	2	2	–	–	–	–	–	–	–	–	c									
Otopharynx lithobates NHMUK 1974.7.5:1 Holotype a	0	2	1	1	1	1	1	1	1	1	1	1	1	1	1	1	1	1	1	2	1	2	2	–	–	–	–	–	–	–	c									
14+17															2	–	–	1	1	1	2	2	–	–	–	–	–	–	–	–	c									
NHMUK 1974.7.5:2-3 Paratypes c	0	2	1	1	1	1	1	1	1	1	1	1	1	1	1	1	1	1	2	1	2	1	2	–	–	–	–	–	–	c										
NOTE: Lots in different order on x-ray 14+16															1	1	–	1	2	1	2	1	–	–	–	–	–	–	–	c										
d	0	2	1	1	1	1	1	1	1	1	1	1	1	1	1	1	1	1	1	2	1	2	2	–	–	–	–	–	–	–	c									
14+17															2	–	–	1	2	1	2	2	–	–	–	–	–	–	–	–	c									
MKO71-VIII-10 YPM? not USNM Paratype? b	0	2	1	1	1	1	1	1	1	1	1	1	1	1	1	1	1	1	1	2	1	2	2	–	–	–	–	–	–	–	c									
14+17															1	1	–	1	2	1	2	1	–	–	–	–	–	–	–	–	c									
MKO71-VI-5a YPM? not USNM Paratype? e	0	2	1	1	1	1	1	1	1	1	1	1	1	1	1	1	1	1	1	2	1	2	2	–	–	–	–	–	–	–	c									
14+17															2	–	–	1	1	1	2	2	–	–	–	–	–	–	–	–	c									
Otopharynx ovatus AMNH 31826	0	1	1	1	1	1	1	1	1	1	1	1	1	1	1	1	1	1	1	2	1	2	2	2	–	–	–	–	–	–	–	–	c							
15+18																1*	1*	1	1	1	2	2	2	–	–	–	–	–	–	–	–	–	c							
AMNH 31832	0	2	1	1	1	1	1	1	1	1	1	1	1	1	1	1	1	1	1	2	1	2	2	2	–	–	–	–	–	–	–	c								
15+17																1	1	1	1	1	1	2	2	–	–	–	–	–	–	–	–	c								
NHMUK 1935.6.14.1487–1489 Lectotype	0	2	1	1	1	1	1	1	1	1	1	1	1	1	1	1	1	1	1	1	2	1	3	1	–	–	–	–	–	–	–	c								
15+17																1	1	1	1	2	2	2	1	–	–	–	–	–	–	–	–	c								
paralectotypes	0	2	1	1	1	1	1	1	1	1	1	1	1	1	1	1	1	1	1	2	1	2	2	1	–	–	–	–	–	–	–	c								
14+18															2	–	1	–	1	2	2	3	–	–	–	–	–	–	–	–	–	c								
	0	2	1	1	1	1	1	1	1	1	1	1	1	1	1	1	1	1	1	1	2	1	3	1	–	–	–	–	–	–	–	c								
15+17																1	1	1	1	2	2	2	1	–	–	–	–	–	–	–	–	c								

Species, Museum, Catalog number / ID<br>Vertebral count	1	2	3	4	5	6	7	8	9	10	11	12	13	14	15	16	17	18	19	20	21	22	23	24	25	26	27	28	29	30	31	32	33	34	35	36	37	38	39	40
Otopharynx selenurus NHMUK 1935.6.14:1671-75 top↘bot	0	2	1	1	1	1	1	1	1	1	1	1	1	1	1	1	1	1	1	1	2	1	2	2	–	–	–	–	–	–	–	–	c							
15+18																1	1	1	1	2	1	3	1	–	–	–	–	–	–	–	–	–	c							
	0	2	1	1	1	1	1	1	1	1	1	1	1	1	1	1	1	1	1	2	1	1	2	2	–	–	–	–	–	–	–	–	c							
15+18																1	1	1	1	1	2	1	2*	1*	–	–	–	–	–	–	–	–	c							
	0	2	1	1	1	1	1	1	1	1	1	1	1	1	1	1	1	1	1	2	2	1	3	–	–	–	–	–	–	–	–	–	c							
14+19															1	1	–	1	1	2	2	1	2	–	–	–	–	–	–	–	–	–	c							
	0	2	1	1	1	1	1	1	1	1	1	1	1	1	1	1	1	1	1	1	2	1	2	2	–	–	–	–	–	–	–	–	c							
15+18																1	1	1	1	1	2	2	2	–	–	–	–	–	–	–	–	–	c							
	0	1	2	1	1	1	1	1	1	1	1	1	1	1	1	1	1	1	1	1	2	1	2	2	–	–	–	–	–	–	–	–	–	c						
15+19																1	1	–	1	1	2	2	2	–	–	–	–	–	–	–	–	–	–	c						
	0	2	1	1	1	1	1	1	1	1	1	1	1	1	1	1	1	1	1	2	1	1	3	–	–	–	–	–	–	–	–	–	c							
15+18																1	1	1	1	1	2	2	1	–	–	–	–	–	–	–	–	–	c							
Otopharynx speciosus AMNH 31791 L to R	0	2	1	1	1	1	1	1	1	1	1	1	1	1	1	1	1	1	2	1	2	2	1	–	–	–	–	–	–	–	–	–	c							
15+18																1	1	1	1	2	2	2	–	–	–	–	–	–	–	–	–	–	c							
	0	2	1	1	1	1	1	1	1	1	1	1	1	1	1	1	1	1	2	1	2	2	–	–	–	–	–	–	–	–	–	c								
14+18															1	1	1	1	1	2	2	1	–	–	–	–	–	–	–	–	–	c								
	0	2	1	1	1	1	1	1	1	1	1	1	1	1	1	1	1	1	2	1	2	2	1	–	–	–	–	–	–	–	–	c								
14+18															1	1	1	1	1	2	2	1	–	–	–	–	–	–	–	–	–	c								
NHMUK 1956.6.12:15-16	0	2	1	1	1	1	1	1	1	1	1	1	1	1	1	1	1	1	2	1	2	2	–	–	–	–	–	–	–	c										
13+17														1	–	1	1	2	1	2	2	–	–	–	–	–	–	–	–	c										
	0	2	1	1	1	1	1	1	1	1	1	1	1	1	1	1	1	1	2	1	2	2	–	–	–	–	–	–	–	c										
13+17														1	–	1	1	1	2	1	3	1	–	–	–	–	–	–	–	c										
Otopharynx tetraspilus AMNH 31802 L to R	0	2	1	1	1	1	1	1	1	1	1	1	1	1	1	1	1	1	1	2	1	2	2	–	–	–	–	–	–	–	–	c								
15+17																1	1	1	1	2	1	2	2	–	–	–	–	–	–	–	–	c								
	0	2	1	1	1	1	1	1	1	1	1	1	1	1	1	1	1	1	1	2	2	1	2	–	–	–	–	–	–	–	c									
15+16																1	1	1	2	1	2	2	1	–	–	–	–	–	–	–	c									
	0	2	1	1	1	1	1	1	1	1	1	1	1	1	1	1	1	1	1	2	1	2	2	–	–	–	–	–	–	–	c									
15+16																1	1	1	2	1	2	2	1	–	–	–	–	–	–	–	c									
YPM 014153 (4), AMNH 221783 SW (1) a	0	2	1	1	1	1	1	1	1	1	1	1	1	1	1	1	1	1	2	1	2	2	–	–	–	–	–	–	–	c										
14+16															2*	–	1	1	1	2	2	1	–	–	–	–	–	–	–	c										
b	0	2	1	1	1	1	1	1	1	1	1	1	1	1	1	1	1	1	1	2	2	2	–	–	–	–	–	–	–	c										
14+16															2	–	–	1	1	2	2	2	–	–	–	–	–	–	–	c										
c	0	2	1	1	1	1	1	1	1	1	1	1	1	1	1	1	1	1	2	1	2	2	–	–	–	–	–	–	–	c										
14+16															2	–	1	1	2	2	2	–	–	–	–	–	–	–	–	c										
d	0	2	1	1	1	1	1	1	1	1	1	1	1	1	1	1	1	1	1	2	1	2	2	–	–	–	–	–	–	–	c									
15+16																2	–	1	2	1	2	2	1	–	–	–	–	–	–	–	c									
e	0	2	1	1	1	1	1	1	1	1	1	1	1	1	1	1	1	1	1	2	1	2	2	–	–	–	–	–	–	–	c									
15+16																2	–	1	2	1	2	2	–	–	–	–	–	–	–	–	c									
Otopharynx tetrastigma NHMUK 1893.11.15:34-37	0	2	1	1	1	1	1	1	1	1	1	1	1	1	1	1	1	1	2	1	2	2	–	–	–	–	–	–	–	c										
Lectotype 13+17														2	–	–	1	2	1	2	2	–	–	–	–	–	–	–	–	c										
	0	2	2	–	1	1	1	1	1	1	1	1	1	1	1	1	1	2	1	2	1?	–	–	–	–	–	–	–	c											
1 of 3 paralectotypes 13+16														1	1	1	1	2	2	2	–	–	–	–	–	–	–	–	c											
NHMUK 1935.6.14:1567-77 39a	0	2	1	1	1	1	1	1	1	1	1	1	1	1	1	1	1	1	2	1	2	2	–	–	–	–	–	–	–	c										
13+17														1	1	–	1	2	1	2	2	–	–	–	–	–	–	–	–	c										
b	0	2	1	1	1	1	1	1	1	1	1	1	1	1	1	1	1	1	2	1	2	1	–	–	–	–	–	–	–	c										
13+17														1	1	–	1	2	1	2	2	–	–	–	–	–	–	–	–	c										
c	0	2	1	1	1	1	1	1	1	1	1	1	1	1	1	1	1	1	1	2	1	3	–	–	–	–	–	–	–	–	c									
14+17															1	1	1	1	1	2	2	1	–	–	–	–	–	–	–	–	c									
d	0	2	1	1	1	1	1	1	1	1	1	1	1	1	1	1	1	1	2	1	2	2	–	–	–	–	–	–	–	c										
13+17														1	1	1	–	2	1	2	2	1	–	–	–	–	–	–	–	c										
e	0	2	1	1	1	1	1	1	1	1	1	1	1	1	1	1	1	1	1	2	1	2	1	–	–	–	–	–	–	–	c									
14+17															1	1	1	1	1	2	2	1	–	–	–	–	–	–	–	–	c									
f	0	2	1	1	1	1	1	1	1	1	1	1	1	1	1	1	1	1	1	2	1	3	–	–	–	–	–	–	–	–	c									
13+18														1	1	1	1	2	1	2	2	–	–	–	–	–	–	–	–	–	c									
g	0	2	1	1	1	1	1	1	1	1	1	1	1	1	1	1	1	1	2	2	1	2	–	–	–	–	–	–	–	c										
13+17														1	1	–	1	2	1	2	3	–	–	–	–	–	–	–	–	c										
h	0	2	1	1	1	1	1	1	1	1	1	1	1	1	1	1	1	2	1	2	2	–	–	–	–	–	–	–	–	c										
13+17														1	1	1	1	1	2	2	1	–	–	–	–	–	–	–	–	c										
40a	0	2	1	1	1	1	1	1	1	1	1	1	1	1	1	1	1	1	1	2	1	2	1	–	–	–	–	–	–	–	c									
14+17															1	1	1	1	1	2	2	2	–	–	–	–	–	–	–	–	c									
b	0	2	1	1	1	1	1	1	1	1	1	1	1	1	1	1	1	1	1	2	1	3	1	–	–	–	–	–	–	–	c									
14+17															1	1	1	1	1	2	1	2	–	–	–	–	–	–	–	–	c									
c	0	2	1	1	1	1	1	1	1	1	1	1	1	1	1	1	1	1	2	1	2	3	–	–	–	–	–	–	–	c										
14+16															1	1	1	1	2	1	3	–	–	–	–	–	–	–	–	c										
d	0	2	1	1	1	1	1	1	1	1	1	1	1	1	1	1	1	1	2	1	2	1	–	–	–	–	–	–	–	c										
14+16															2	–	1	2	1	2	2	–	–	–	–	–	–	–	–	c										
e	0	2	1	1	1	1	1	1	1	1	1	1	1	1	1	1	1	1	1	2	1	2	2	–	–	–	–	–	–	–	c									
14+17															1	1	1	1	1	2	2	2	–	–	–	–	–	–	–	–	c									

Species, Museum, Catalog number / ID<br>Vertebral count	1	2	3	4	5	6	7	8	9	10	11	12	13	14	15	16	17	18	19	20	21	22	23	24	25	26	27	28	29	30	31	32	33	34	35	36	37	38	39	40
f	0	2	1	1	1	1	1	1	1	1	1	1	1	1	1	1	1	1	2	1	2	2*	1	–	–	–	–	–	–	c										
14+16																2	1	1	2	1	2	3	–	–	–	–	–	–	–	–	–	–	–	c						
Placidochromis hennydaviesae USNM 210698 Holotype	0	2	1	1	1	1	1	1	1	1	1	1	1	1	1	1	1	2	1	2	2	–	–	–	–	–	–	–	–	c										
13+17														1	1	1	1	1	2	2	–	–	–	–	–	–	–	–	–	c										
Placidochromis johnstoni AMNH 31775 out of x-ray order	0	1	2	1	1	1	1	1	1	1	1	1	1	1	1	1	1	1	1	2	1	2	2	–	–	–	–	–	–	–	c									
14+17															1	1	1	1	1	2	2	2	–	–	–	–	–	–	–	–	c									
	0	2	1	1	1	1	1	1	1	1	1	1	1	1	1	1	1	1	2	2	2	1	–	–	–	–	–	–	–	–	c									
13+18														1	–	1	1	2	1	2	3	–	–	–	–	–	–	–	–	–	c									
AMNH 31776	0	2	1	1	1	1	1	1	1	1	1	1	1	1	1	1	1	1	2	1	2	2	–	–	–	–	–	–	–	–	c									
13+18														1	–	1	1	1	2	1	2	1	–	–	–	–	–	–	–	–	c									
AMNH 222058	0	2	1	1	1	1	1	1	1	1	1	1	1	1	1	1	1	1	1	2	3	–	–	–	–	–	–	–	–	c										
14+16															1	1	1	1	2	2	2	–	–	–	–	–	–	–	–	c										
Placidochromis longimanus USNM 330603 L. Malombe a	0	2	1	1	1	1	1	1	1	1	1	1	1	1	1	1	1	1	1	1	2	1	3	–	–	–	–	–	–	–	–	c								
15+17																1	1	1	1	2	2	2	–	–	–	–	–	–	–	–	–	c								
b	0	2	1	1	1	1	1	1	1	1	1	1	1	1	1	1	1	1	1	1	2	2	2	–	–	–	–	–	–	–	–	c								
15+17																1	1	1	1	2	2	2	–	–	–	–	–	–	–	–	–	c								
c	0	2	1	1	1	1	1	1	1	1	1	1	1	1	1	1	1	1	1	2	1	2	2	–	–	–	–	–	–	–	–	c								
15+17																1	1	1	1	2	2	2	–	–	–	–	–	–	–	–	–	c								
d	0	2	1	1	1	1	1	1	1	1	1	1	1	1	1	1	1	1	1	1	2	2	1	–	–	–	–	–	–	–	–	c								
15+17																1	1	1	1	1	2	3	–	–	–	–	–	–	–	–	–	c								
e	0	2	1	1	1	1	1	1	1	1	1	1	1	1	1	1	1	1	1	1	2	2	2	–	–	–	–	–	–	–	–	c								
15+17																1	1	1	1	2	1	3	–	–	–	–	–	–	–	–	–	c								
f	0	2	1	1	1	1	1	1	1	1	1	1	1	1	1	1	1	1	1	2	1	2	2	–	–	–	–	–	–	–	–	c								
15+17																1	1	1	2	1	1	3	–	–	–	–	–	–	–	–	–	c								
g	0	2	1	1	1	1	1	1	1	1	1	1	1	1	1	1	1	1	2	1	2	2	–	–	–	–	–	–	–	–	–	c								
15+17																1	1	1	2	1	3	–	–	–	–	–	–	–	–	–	–	c								
h	0	2	1	1	1	1	1	1	1	1	1	1	1	1	1	1	1	1	1	2	1	3	1	–	–	–	–	–	–	–	–	c								
15+17																1	1	1	2	1	2	2	–	–	–	–	–	–	–	–	–	c								
I	0	2	1	1	1	1	1	1	1	1	1	1	1	1	1	1	1	1	1	2	1	2	2	–	–	–	–	–	–	–	–	c								
15+17																1	1	1	2	1	2	2	–	–	–	–	–	–	–	–	–	c								
j	0	2	1	1	1	1	1	1	1	1	1	1	1	1	1	1	1	1	1	2	1	2	2	–	–	–	–	–	–	–	–	c								
15+17																1	1	1	1	2	2	2	–	–	–	–	–	–	–	–	–	c								
k	0	2	1	1	1	1	1	1	1	1	1	1	1	1	1	1	1	1	1	1	2	2	1	–	–	–	–	–	–	–	–	c								
15+17																1	1	1	1	2	2	1	–	–	–	–	–	–	–	–	–	c								
l	0	2	1	1	1	1	1	1	1	1	1	1	1	1	1	1	1	1	1	1	2	2	2	–	–	–	–	–	–	–	–	c								
15+17																2	–	1	1	1	2	2	–	–	–	–	–	–	–	–	–	c								
m	0	2	1	1	1	1	1	1	1	1	1	1	1	1	1	1	1	1	1	2	1	3	–	–	–	–	–	–	–	–	c									
14+17															1	1	1	1	1	2	2	1	–	–	–	–	–	–	–	–	c									
35a	0	2	1	1	1	1	1	1	1	1	1	1	1	1	1	1	1	1	1	2	1	2	2	–	–	–	–	–	–	–	–	c								
15+17																1	1	1	1	1	2	2	–	–	–	–	–	–	–	–	–	c								
b	0	2	1	1	1	1	1	1	1	1	1	1	1	1	1	1	1	1	1	2	1	3	–	–	–	–	–	–	–	–	c									
14+17															1	–	1	1	2	1	3	–	–	–	–	–	–	–	–	–	c									
c	0	2	1	1	1	1	1	1	1	1	1	1	1	1	1	1	1	1	2	1	2	2	–	–	–	–	–	–	–	–	–	c								
14+18															2	1	–	1	1	2	2	–	–	–	–	–	–	–	–	–	–	c								
d	0	1	2	1	1	1	1	1	1	1	1	1	1	1	1	1	1	1	1	2	1	2	1	–	–	–	–	–	–	–	–	c								
15+17																1	1	1	1	2	2	2	–	–	–	–	–	–	–	–	–	c								
e	0	2	1	1	1	1	1	1	1	1	1	1	1	1	1	1	1	1	1	2	1	2	2	–	–	–	–	–	–	–	–	c								
15+17																1	1	1	1	2	1	2	–	–	–	–	–	–	–	–	–	c								
f	0	2	1	1	1	1	1	1	1	1	1	1	1	1	1	1	1	1	1	1	2	1	3	–	–	–	–	–	–	–	–	c								
15+17																1	1	1	1	1	2	2	–	–	–	–	–	–	–	–	–	c								
g	0	2	1	1	1	1	1	1	1	1	1	1	1	1	1	1	1	1	1	1	2	1	3	–	–	–	–	–	–	–	–	c								
15+17																1	1	1	2	1	1	3	–	–	–	–	–	–	–	–	–	c								
h	0	2	1	1	1	1	1	1	1	1	1	1	1	1	1	1	1	1	1	1	2	1	3	–	–	–	–	–	–	–	–	c								
15+17																1	1	1	1	1	2	2	1	–	–	–	–	–	–	–	–	c								
I	0	2	1	1	1	1	1	1	1	1	1	1	1	1	1	1	1	1	1	2	1	3	–	–	–	–	–	–	–	–	–	c								
15+17																2*	–	1	2	1	2	2	–	–	–	–	–	–	–	–	–	c								
j	0	2	1	1	1	1	1	1	1	1	1	1	1	1	1	1	1	1	1	2	1	3	–	–	–	–	–	–	–	–	c									
14+17															1	1	1	1	1	1	3	–	–	–	–	–	–	–	–	–	c									
k	0	2	1	1	1	1	1	1	1	1	1	1	1	1	1	1	1	1	2	1	1	3	–	–	–	–	–	–	–	–	–	c								
15+17																1	1	1	2	1	2	1	–	–	–	–	–	–	–	–	–	c								
l	0	2	1	1	1	1	1	1	1	1	1	1	1	1	1	1	1	1	1	1	2	1	3	–	–	–	–	–	–	–	c									
15+16																1	1	1	1	2	1	3	–	–	–	–	–	–	–	–	c									
m	0	2	1	1	1	1	1	1	1	1	1	1	1	1	1	1	1	1	1	2	1	3	–	–	–	–	–	–	–	–	c									
15+16																2	–	1	2	1	2	2	–	–	–	–	–	–	–	–	c									
n	0	2	1	1	1	1	1	1	1	1	1	1	1	1	1	1	1	1	1	2	1	3	–	–	–	–	–	–	–	–	–	c								
15+17																1	1	1	2	1	2	2	–	–	–	–	–	–	–	–	–	c								
o	0	2	1	1	1	1	1	1	1	1	1	1	1	1	1	1	1	1	1	2	1	2	1	–	–	–	–	–	–	–	–	c								
15+17																1	1	1	2	1	2	2	–	–	–	–	–	–	–	–	–	c								

Species, Museum, Catalog number / ID<br>Vertebral count	1	2	3	4	5	6	7	8	9	10	11	12	13	14	15	16	17	18	19	20	21	22	23	24	25	26	27	28	29	30	31	32	33	34	35	36	37	38	39	40
Placidochromis macrognathus uncat? MKO71-VII-15	0	2	1	1	1	1	1	1	1	1	1	1	1	1	1	1	1	1	2	1	2	2	–	–	–	–	–	–	–	–	c									
13+18														2	–	1	1	1	2	1	2	–	–	–	–	–	–	–	–	–	c									
	0	1	2	1	1	1	1	1	1	1	1	1	1	1	1	1	1	2	1	1	2	2	–	–	–	–	–	–	–	–	c									
14+17															1	1	1	1	2	2	2	–	–	–	–	–	–	–	–	–	c									
Placidochromis milomo AMNH 92694	0	2	1	1	1	1	1	1	1	1	1	1	1	1	1	1	1	1	1	2	1	2	3	–	–	–	–	–	–	–	c									
15+16																1	1	1	1	2	2	2	1	–	–	–	–	–	–	–	c									
	0	1	2	1	1	1	1	1	1	1	1	1	1	1	1	1	1	1	1	2	1	3	2	–	–	–	–	–	–	c										
15+15																2	–	1	2	1	2	3	–	–	–	–	–	–	–	c										
	0	2	1	1	1	1	1	1	1	1	1	1	1	1	1	1	1	1	2	1	2	3	–	–	–	–	–	–	–	c										
14+16															1	1	–	2	1	1	2	2	–	–	–	–	–	–	–	c										
USNM 265487	0	2	1	1	1	1	1	1	1	1	1	1	1	1	1	1	1	1	2	1	2	3	–	–	–	–	–	–	–	c										
14+16															1	1	1	1	1	1	3	1	–	–	–	–	–	–	–	c										
Protomelas annectens NHMUK 1935.6.14.847–852	0	2	1	1	1	1	1	1	1	1	1	1	1	1	1	1	1	1	1	2	1	2	2	–	–	–	–	–	–	–	–	–	c							
15+18																1	1	1	1	1	2	2	1	–	–	–	–	–	–	–	–	–	c							
	0	2	1	1	1	1	1	1	1	1	1	1	1	1	1	1	1	1	1	1	2	1	2	2	–	–	–	–	–	–	–	–	c							
15+18																1	1	1	–	2	1	1	3	–	–	–	–	–	–	–	–	–	c							
Protomelas fenestratus AMNH 221891 a	0	2	1	1	1	1	1	1	1	1	1	1	1	1	1	1	1	1	1	2	1	1	3	–	–	–	–	–	–	–	c									
15+16																1	1	1	1	1	2	1	2	–	–	–	–	–	–	–	c									
b	0	2	1	1	1	1	1	1	1	1	1	1	1	1	1	1	1	1	1	2	1	1	3	1	–	–	–	–	–	–	c									
15+16																–	1	1	1	1	2	2	2	–	–	–	–	–	–	–	c									
c	0	2	1	1	1	1	1	1	1	1	1	1	1	1	1	1	1	1	1	2	1	2	2	1	–	–	–	–	–	–	c									
15+16																1	1	1	1	1	2	2	1	–	–	–	–	–	–	–	c									
d	0	2	1	1	1	1	1	1	1	1	1	1	1	1	1	1	1	1	1	2	1	2	2	–	–	–	–	–	–	–	c									
14+17															2	–	–	1	1	2	1	2	1	–	–	–	–	–	–	–	c									
e	0	2	1	1	1	1	1	1	1	1	1	1	1	1	1	1	1	1	1	2	1	2	2	–	–	–	–	–	–	–	c									
14+17															1	–	1	1	1	2	1	3	–	–	–	–	–	–	–	–	c									
f	0	2	1	1	1	1	1	1	1	1	1	1	1	1	1	1	1	1	1	2	1	2	2	–	–	–	–	–	–	c										
14+16															1	–	1	1	1	2	1	3	–	–	–	–	–	–	–	c										
g	0	2	1	1	1	1	1	1	1	1	1	1	1	1	1	1	1	1	1	2	1	2	2	–	–	–	–	–	–	–	c									
15+16																1	1	1	1	1	2	2	1	–	–	–	–	–	–	–	c									
h	0	2	1	1	1	1	1	1	1	1	1	1	1	1	1	1	1	1	2	1	2	2	1	–	–	–	–	–	–	–	c									
14+17															1	–	1	1	1	2	1	2	–	–	–	–	–	–	–	–	c									
i	0	2	1	1	1	1	1	1	1	1	1	1	1	1	1	1	1	1	1	2	1	3	1	–	–	–	–	–	–	–	c									
14+17															2	–	–	1	1	2	1	3	–	–	–	–	–	–	–	–	c									
j	0	2	1	1	1	1	1	1	1	1	1	1	1	1	1	1	1	1	1	1	2	2	2	1?	–	–	–	–	–	–	c									
15+16																1	1	–	1	2	1	2	2?	–	–	–	–	–	–	–	c									
AMNH 222022 (14 fluid + 1 c&s of these 26) a	0	2	1	1	1	1	1	1	1	1	1	1	1	1	1	1	1	1	2	1	2	2	–	–	–	–	–	–	–	–	c									
14+17															1	1	–	1	2	1	2	2	–	–	–	–	–	–	–	–	c									
b	0	2	1	1	1	1	1	1	1	1	1	1	1	1	1	1	1	1	1	2	1	3	1	–	–	–	–	–	–	–	c									
14+17															1	1	–	1	2	1	2	2	–	–	–	–	–	–	–	–	c									
c	0	2	1	1	1	1	1	1	1	1	1	1	1	1	1	1	1	1	1	2	2	2	–	–	–	–	–	–	–	c										
14+16															1	1	1	1	2	1	2	2	–	–	–	–	–	–	–	c										
d	0	2	1	1	2	1	1	1	1	1	1	1	1	1	1	1	1	2	1	2	2	–	–	–	–	–	–	–	–	c										
13+17*														1	–	1	1	1	2	1	3	–	–	–	–	–	–	–	–	c										
e	0	2	1	1	1	1	1	1	1	1	1	1	1	1	1	1	1	1	2	1	2	2	–	–	–	–	–	–	–	–	c									
14+17															1	1	–	1	2	1	2	2	–	–	–	–	–	–	–	–	c									
f	0	2	1	1	1	1	1	1	1	1	1	1	1	1	1	1	1	1	2	1	2	2	–	–	–	–	–	–	–	–	c									
14+17															1	1	1	1	1	2	2	1	–	–	–	–	–	–	–	–	c									
g	0	2	1	1	2	–	1	1	1	1	1	1	1	1	1	1	1	1	2	1	2	2	–	–	–	–	–	–	–	–	c									
14+17															2	–	–	1	2	1	2	2	–	–	–	–	–	–	–	–	c									
h	0	2	1	1	1	1	1	1	1	1	1	1	1	1	1	1	1	1	1	2	1	2	1	–	–	–	–	–	–	–	c									
14+17															1	1	1	1	1	2	1	2	–	–	–	–	–	–	–	–	c									
i	0	2	1	1	1	1	1	1	1	1	1	1	1	1	1	1	1	1	1	2	1	3	1	–	–	–	–	–	–	–	c									
14+17															1	1	1	1	1	2	1	2	–	–	–	–	–	–	–	–	c									
j	0	2	1	1	1	1	1	1	1	1	1	1	1	1	1	1	1	1	2	1	2	2	–	–	–	–	–	–	–	c										
14+16															1	1	1	1	1	2	2	1	–	–	–	–	–	–	–	c										
k	0	2	1	1	1	1	1	1	1	1	1	1	1	1	1	1	1	1	2	1	2	2	–	–	–	–	–	–	c											
13+16														2	–	–	1	1	2	1	2	–	–	–	–	–	–	–	c											
l	0	2	1	1	1	1	1	1	1	1	1	1	1	1	1	1	1	1	2	1	2	3	–	–	–	–	–	–	–	–	c									
14+17															1	1	–	1	2	1	1	3	–	–	–	–	–	–	–	–	c									
m	0	2	1	1	1	1	1	1	1	1	1	1	1	1	1	1	1	1	2	1	2	3	–	–	–	–	–	–	–	–	c									
14+17															1	1	–	1	2	1	2	2	–	–	–	–	–	–	–	–	c									
n	0	2	1	1	1	1	1	1	1	1	1	1	1	1	1	1	1	1	2	2	2	1	–	–	–	–	–	–	–	c										
13+17														1	1	–	1	2	1	2	2	–	–	–	–	–	–	–	–	c										
o	0	2	1	1	1	1	1	1	1	1	1	1	1	1	1	1	1	2	1	2	2	1	–	–	–	–	–	–	–	c										
13+17														1	–	1	1	1	1	2	3	–	–	–	–	–	–	–	–	c										
p	0	2	1	1	1	1	1	1	1	1	1	1	1	1	1	1	1	1	1	2	2	2	–	–	–	–	–	–	–	c										
14+16															1	1	1	1	1	2	2	–	–	–	–	–	–	–	–	c										

Species, Museum, Catalog number / ID<br>Vertebral count	1	2	3	4	5	6	7	8	9	10	11	12	13	14	15	16	17	18	19	20	21	22	23	24	25	26	27	28	29	30	31	32	33	34	35	36	37	38	39	40
q	0	2	1	1	1	1	1	1	1	1	1	1	1	1	1	1	1	1	2	1	2	2	–	–	–	–	–	–	–	–	c									
14+17															1	1	1	1	1	2	2	1	–	–	–	–	–	–	–	–	c									
r	0	2	1	1	1	1	1	1	1	1	1	1	1	1	1	1	1	1	2	1	2	2	–	–	–	–	–	–	–	c										
14+16															1	1	1	1	1	2	3	–	–	–	–	–	–	–	–	c										
s	0	2	1	1	1	1	1	1	1	1	1	1	1	1	1	1	1	1	2	1	2	3	–	–	–	–	–	–	–	–	c									
14+17															1	1	–	1	2	1	2	2	–	–	–	–	–	–	–	–	c									
t	0	2	1	1	1	1	1	1	1	1	1	1	1	1	1	1	1	1	1	2	2	2	1	–	–	–	–	–	–	–	c									
14+17															1	1	1	–	2	1	2	2	–	–	–	–	–	–	–	–	c									
u	0	2	1	1	1	1	1	1	1	1	1	1	1	1	1	1	1	1	2	1	1	3	–	–	–	–	–	–	–	–	c									
14+17															1	1	1	1	1	2	2	1	–	–	–	–	–	–	–	–	c									
v	0	2	1	1	1	1	1	1	1	1	1	1	1	1	1	1	1	1	2	1	2	3	–	–	–	–	–	–	–	–	c									
14+17															1	1	1	1	2	1	3	–	–	–	–	–	–	–	–	–	c									
w	0	1	2	1	1	1	1	1	1	1	1	1	1	1	1	1	1	1	1	1	2	1	3	–	–	–	–	–	–	–	c									
14+17															1	1	–	1	1	2	1	3	–	–	–	–	–	–	–	–	c									
x	0	2	1	1	1	1	1	1	1	1	1	1	1	1	1	1	1	1	2	1	2	3	–	–	–	–	–	–	–	–	c									
14+17															1	1	1	1	1	2	2	1	–	–	–	–	–	–	–	–	c									
y	0	2	1	1	1	1	1	1	1	1	1	1	1	1	1	1	1	1	2	1	2	2	–	–	–	–	–	–	–	c										
14+16															1	1	1	1	2	1	3	–	–	–	–	–	–	–	–	c										
z	0	2	1	1	1	1	1	1	1	1	1	1	1	1	1	1	1	1	1	2	1	2	2	–	–	–	–	–	–	–	c									
14+17															1	1	1	1	1	1	2	2	–	–	–	–	–	–	–	–	c									
Protomelas insignis NHMUK 1935.6.14.839-843 syntypes	0	2	1	1	1	1	1	1	1	1	1	1	1	1	1	1	1	1	1	2	1	2	2	1	–	–	–	–	–	–	–	c								
15+17																1	1	1	1	1	2	2	1	–	–	–	–	–	–	–	–	c								
	0	2	1	1	1	1	1	1	1	1	1	1	1	1	1	1	1	1	1	2	1	2	2	2	–	–	–	–	–	–	–	c								
15+17																2	–	–	1	2	1	2	3	–	–	–	–	–	–	–	–	c								
Protomelas kirkii YPM 024312	0	2	1	1	1	1	1	1	1	1	1	1	1	1	1	1	1	1	2	1	2	1	–	–	–	–	–	–	–	c										
14+16															1	1	1	1	2	2	1	–	–	–	–	–	–	–	–	c										
NHMUK 1893.11.15.7 Lectotype	0	2	1	1	1	1	1	1	1	1	1	1	1	1	1	1	1	1	2	1	2	1	–	–	–	–	–	–	–	c										
13+17														1	1	1	–	2	1	2	1	–	–	–	–	–	–	–	–	c										
NHMUK 1893.11.15.8-11 paralectotype	0	2	1	1	1	1	1	1	1	1	1	1	1	1	1	1	1	2	1	2	2	1	–	–	–	–	–	–	–	c										
13+17														1	1	–	1	2	1	2	2	–	–	–	–	–	–	–	–	c										
Protomelas labridens YPM 014519	0	2	1	1	1	1	1	1	1	1	1	1	1	1	1	1	1	1	1	2	1	2	1	–	–	–	–	–	–	–	c									
14+17															1	1	1	1	1	2	1	2	1	–	–	–	–	–	–	–	c									
Protomelas ornatus NHMUK 1921.9.6.112 Holotype	0	2	1	1	1	1	1	1	1	1	1	1	1	1	1	1	1	1	2	1	2	2	–	–	–	–	–	–	–	c										
13+17														1	–	1	1	1	2	1	2	1	–	–	–	–	–	–	–	c										
NHMUK 1935.6.14.1013	0	2	1	1	1	1	1	1	1	1	1	1	1	1	1	1	1	2	1	2	2	1	–	–	–	–	–	–	–	c										
13+17														2	–	–	1	1	2	2	2	–	–	–	–	–	–	–	–	c										
Protomelas spilopterus AMNH 31882	0	2	1	1	1	1	1	1	1	1	1	1	1	1	1	1	1	1	1	2	1	2	2	–	–	–	–	–	–	–	c									
14+17															1	1	1	1	1	2	2	2	–	–	–	–	–	–	–	–	c									
AMNH 31761	0	2	1	1	1	1	1	1	1	1	1	1	1	1	1	1	1	1	1	2	1	2	2	–	–	–	–	–	–	–	c									
15+16																1	1	1	2	1	2	2	–	–	–	–	–	–	–	–	c									
AMNH 31846	0	2	1	1	1	1	1	1	1	1	1	1	1	1	1	1	1	1	1	2	2	2	–	–	–	–	–	–	–	c										
14+16															1	1	1	1	2	2	2	–	–	–	–	–	–	–	–	c										
AMNH 31884	0	2	1	1	1	1	1	1	1	1	1	1	1	1	1	1	1	1	1	1	2	1	3	–	–	–	–	–	–	–	–	c								
15+17																1	1	1	1	2	2	2	–	–	–	–	–	–	–	–	–	c								
NHMUK 1935.6.14.644-647 paralectotypes	0	2	1	1	1	1	1	1	1	1	1	1	1	1	1	1	1	1	1	2	2	2	1	–	–	–	–	–	–	c										
14+16															1	1	1	1	2	1	4	–	–	–	–	–	–	–	–	c										
	0	2	1	1	1	1	1	1	1	1	1	1	1	1	1	1	1	1	2	1	2	1	–	–	–	–	–	–	–	c										
13+17														2	–	–	2	1	2	2	1	–	–	–	–	–	–	–	–	c										
Protomelas taeniolatus NHMUK 1961.12.1.355	0	2	1	1	1	1	1	1	1	1	1	1	1	1	1	1	1	1	1	2	1	2	2	–	–	–	–	–	–	–	c									
14+17															2	–	–	1	2	1	2	2	–	–	–	–	–	–	–	–	c									
Protomelas triaenodon AMNH 31778 a	0	2	1	1	1	1	1	1	1	1	1	1	1	1	1	1	1	1	1	2	1	2	2	–	–	–	–	–	–	–	c									
14+17															1	–	1	1	1	2	2	2	–	–	–	–	–	–	–	–	c									
b	0	2	1	1	1	1	1	1	1	1	1	1	1	1	1	1	1	1	1	2	1	2	2	–	–	–	–	–	–	–	–	c								
14+18															2	–	–	1	2	2	1	3	–	–	–	–	–	–	–	–	–	c								
c	0	2	1	1	1	1	1	1	1	1	1	1	1	1	1	1	1	1	1	2	2	2	1	–	–	–	–	–	–	–	c									
14+17																		1	1	1	2	1	2	3	–	–	–	–	–	–	–	–	–	–	–	–	c			
Sciaenochromis ahli NHMUK 1935.6.14:1460-68 a	0	2	1	1	1	1	1	1	1	1	1	1	1	1	1	1	1	1	2	1	2	2	–	–	–	–	–	–	–	–	c									
13+18														1	–	1	1	1	2	2	2	–	–	–	–	–	–	–	–	–	c									
b	0	2	1	1	1	1	1	1	1	1	1	1	1	1	1	1	1	1	2	1	2	2	–	–	–	–	–	–	–	–	c									
13+18														1	–	1	1	1	2	1	3	–	–	–	–	–	–	–	–	–	c									
c	0	2	1	1	1	1	1	1	1	1	1	1	1	1	1	1	1	1	1	2	2	2	–	–	–	–	–	–	–	–	c									
13+18														1	–	1	1	1	2	2	2	–	–	–	–	–	–	–	–	–	c									
d	0	2	1	1	1	1	1	1	1	1	1	1	1	1	1	1	1	1	1	2	1	3	–	–	–	–	–	–	–	–	c									
14+17															1	1	1	1	1	2	2	1	–	–	–	–	–	–	–	–	c									
e	0	2	1	1	1	1	1	1	1	1	1	1	1	1	1	1	1	1	1	2	1	3	1	–	–	–	–	–	–	–	c									
13+18														1	–	1	1	1	2	1	3	–	–	–	–	–	–	–	–	–	c									
f	0	2	1	1	1	1	1	1	1	1	1	1	1	1	1	1	1	2	1	2	2	1	–	–	–	–	–	–	–	–	c									
13+18														1	–	1	1	2	1	2	2	–	–	–	–	–	–	–	–	–	c									

Species, Museum, Catalog number / ID<br>Vertebral count	1	2	3	4	5	6	7	8	9	10	11	12	13	14	15	16	17	18	19	20	21	22	23	24	25	26	27	28	29	30	31	32	33	34	35	36	37	38	39	40
g	0	2	1	1	1	1	1	1	1	1	1	1	1	1	1	1	1	1	2	1	2	1	–	–	–	–	–	–	–	–	c									
13+18														1	–	1	1	1	2	2	2	–	–	–	–	–	–	–	–	–	c									
h	0	1	2	1	1	1	1	1	1	1	1	1	1	1	1	1	1	1	1	2	2	2	–	–	–	–	–	–	–	–	c									
13+18														1	–	1	1	2	1	2	2	–	–	–	–	–	–	–	–	–	c									
i	0	2	1	1	1	1	1	1	1	1	1	1	1	1	1	1	1	1	1	2	2	2	–	–	–	–	–	–	–	–	c									
13+18														1	–	1	1	1	2	1	2	2	–	–	–	–	–	–	–	–	c									
j	0	2	1	1	1	1	1	1	1	1	1	1	1	1	1	1	1	1	1	2	2	2	–	–	–	–	–	–	–	–	c									
13+18														1	–	1	1	2	1	2	2	–	–	–	–	–	–	–	–	–	c									
NHMUK 1935.6.14:1469-1471	0	2	1	1	1	1	1	1	1	1	1	1	1	1	1	1	1	1	1	2	2	2	–	–	–	–	–	–	–	–	c									
14+17															1	1	1	1	1	2	2	1	–	–	–	–	–	–	–	–	c									
	0	1	2	1	1	1	1	1	1	1	1	1	1	1	1	1	1	1	2	1	2	2	–	–	–	–	–	–	–	–	c									
14+17															1	1	1	1	2	1	3	–	–	–	–	–	–	–	–	–	c									
	–	2	1	1	1	1	1	1	1	1	1	1	1	1	1	1	1	1	2	1	2	2	–	–	–	–	–	–	–	–	c									
13+18														1	–	1	1	1	2	1	3	–	–	–	–	–	–	–	–	–	c									
NHMUK 1935.6.14:1472-1473	0	2	1	1	1	1	1	1	1	1	1	1	1	1	1	1	1	1	2	1	2	2	–	–	–	–	–	–	–	–	c									
13+18														1	1	1	1	2	1	3	–	–	–	–	–	–	–	–	–	–	c									
	0	2	1	1	1	1	1	1	1	1	1	1	1	1	1	1	1	1	2	1	2	2	–	–	–	–	–	–	–	–	c									
14+17															1	1	1	1	2	1	3	–	–	–	–	–	–	–	–	–	c									
	0	2	1	1	1	1	1	1	1	1	1	1	1	1	1	1	1	1	1	2	2	2	1	–	–	–	–	–	–	–	–	c								
14+18															1	1	1	1	1	2	2	1	–	–	–	–	–	–	–	–	–	c								
Stigmatochromis modestus AMNH 31792	0	2	1	1	1	1	1	1	1	1	1	1	1	1	1	1	1	1	1	2	1	2	2	–	–	–	–	–	–	–	c									
14+17															1	1	1	1	1	1	2	2	–	–	–	–	–	–	–	–	c									
AMNH 31794	0	2	1	1	1	1	1	1	1	1	1	1	1	1	1	1	1	1	1	2	2	2	1	–	–	–	–	–	–	–	c									
13+18														1	–	1	1	1	1	2	2	2	–	–	–	–	–	–	–	–	c									
AMNH 31828	0	2	1	1	1	1	1	1	1	1	1	1	1	1	1	1	1	1	2	1	2	3	–	–	–	–	–	–	–	c										
13+17														1	–	1	1	1	1	2	2	2	–	–	–	–	–	–	–	c										
NHMUK 1893.1.17:5 Holotype	0	2	1	1	1	1	1	1	1	1	1	1	1	1	1	1	1	1	2	1	2	2	1	–	–	–	–	–	–	c										
13+17														2	–	–	1	1	2	1	2	2	–	–	–	–	–	–	–	c										
NHMUK 1969.3.11:4	0	2	1	1	1	1	1	1	1	1	1	1	1	1	1	1	1	1	2	1	2	2	–	–	–	–	–	–	–	c										
13+17														1	–	1	1	1	2	1	3	–	–	–	–	–	–	–	–	c										
St. pholidophorus NHMUK 1935.6.14:1544 Holotype	0	2	1	1	1	1	1	1	1	1	1	1	1	1	1	1	1	1	2	1	2	3	1	–	–	–	–	–	–	–	c									
13+18														1	–	1	1	1	2	1	2	3	–	–	–	–	–	–	–	–	c									
NHMUK 1969.10.10:1-2	0	2	1	1	1	1	1	1	1	1	1	1	1	1	1	1	1	1	2	1	2	2	1	–	–	–	–	–	–	–	c									
13+18														2?	–?	–?	1	1	2	2	2	1	–	–	–	–	–	–	–	–	c									
	0	2	1	1	1	1	1	1	1	1	1	1	1	1	1	1	1	1	2	1	2	3	–	–	–	–	–	–	–	–	c									
14+17															1	1	1	1	2	2	1	2	–	–	–	–	–	–	–	–	c									
NHMUK unregistered as of 1972	0	2	1	1	1	1	1	1	1	1	1	1	1	1	1	1	1	1	2	1	2	3	–	–	–	–	–	–	–	–	–	c								
14+18															1	1	1	1	1	2	2	1	–	–	–	–	–	–	–	–	–	c								
	0	2	1	1	1	1	1	1	1	1	1	1	1	1	1	1	1	1	2	1	2	3	–	–	–	–	–	–	–	–	c									
14+17															1	1	1	1	1	2	3	–	–	–	–	–	–	–	–	–	c									
St. pleurospilus NHMUK 1935.6.14:1475 Holotype	0	2	1	1	1	1	1	1	1	1	1	1	1	1	1	1	1	1	2?	?	?	?	–	–	–	–	–	–	–	–	c									
13+18														1	–	1	1	1	2	1	?	?	–	–	–	–	–	–	–	–	c									
St. woodi NHMUK 1921.9.6:139-144 Paralectos a	0	2	1	1	1	1	1	1	1	1	1	1	1	1	1	1	1	1	2	1	2	2	–	–	–	–	–	–	–	–	c									
13+18														1	–	1	1	1	2	1	3	–	–	–	–	–	–	–	–	–	c									
b	0	2	1	1	1	1	1	1	1	1	1	1	1	1	1	1	1	1	1	2	2	2	–	–	–	–	–	–	–	–	c									
13+18														1	–	1	1	1	2	1	3	–	–	–	–	–	–	–	–	–	c									
c	0	2	1	1	1	1	1	1	1	1	1	1	1	1	1	1	1	1	1	2	2	2	–	–	–	–	–	–	–	–	c									
13+18														1	–	1	1	1	1	2	2	2?	–	–	–	–	–	–	–	–	c									
Lectotype **d**	0	2	1	1	1	1	1	1	1	1	1	1	1	1	1	1	1	1	1	2	2	2	–	–	–	–	–	–	–	–	c									
14+17															1	1	1	1	1	2	1	3	–	–	–	–	–	–	–	–	c									
e	0	2	1	1	1	1	1	1	1	1	1	1	1	1	1	1	1	1	2	1	2	2	–	–	–	–	–	–	–	c										
13+17														1	–	1	1	1	2	2	2	–	–	–	–	–	–	–	–	c										
NHMUK 1935.6.14:1541-1542	0	2	1	1	1	1	1	1	1	1	1	1	1	1	1	1	1	1	1	2	2	1?	?	–	–	–	–	–	–	–	c									
14+17															1	–	1	1	1	1	2	1	2?	–	–	–	–	–	–	–	c									
	0	2	1	1	1	1	1	1	1	1	1	1	1	1	1	1	1	?	?	?	?	?	?	–	–	–	–	–	–	–	–	c								
13+19														1	–	1	1	1	?	?	?	?	?	–	–	–	–	–	–	–	–	c								
Taeniochromis holotaenia YPM 007827	0	1	2	1	1	1	1	1	1	1	1	1	1	1	1	1	1	1	1	1	1	2	1	2	2	–	–	–	–	–	c									
15+18																1	1	1	1	1	1	2	2	1	–	–	–	–	–	–	c									
Taeniolethrinops praeorbitalis YPM 014420	0	2	1	1	1	1	1	1	1	1	1	1	1	1	1	1	1	1	2	1	2	2	1	–	–	–	–	–	–	–	–	–	c							
15+18																2	–	1	2	1	2	2	–	–	–	–	–	–	–	–	–	–	c							
Tramitichromis brevis YPM 007847	0	2	1	1	1	1	1	1	1	1	1	1	1	1	1	1	1	1	2	1	2	2	–	–	–	–	–	–	–	–	c									
14+17															1	1	1	1	2	1	3	–	–	–	–	–	–	–	–	–	c									
Trematocranus labifer NHMUK 1935.6.14:1643-44 Paralectos	0	2	1	1	1	1	1	1	1	1	1	1	1	1	1	1	1	1	2	1	2	2	–	–	–	–	–	–	–	–	c									
"3" (2 fish) on x-ray 14+17															1	1	1	1	1	2	2	2	–	–	–	–	–	–	–	–	c									
	0	2	1	1	1	1	1	1	1	1	1	1	1	1	1	1	1	1	2	1	2	2	–	–	–	–	–	–	–	–	c									
14+17															1	1	1	1	2	2	2	–	–	–	–	–	–	–	–	–	c									
NHMUK 1935.6.14:1647 Paralecto	0	2	1	1	1	1	1	1	1	1	1	1	1	1	1	1	1	1	2	1	2	2	–	–	–	–	–	–	–	–	c									
"1" on x-ray 14+17															1	1	1	2	1	2	2	–	–	–	–	–	–	–	–	–	c									

Species, Museum, Catalog number / ID<br>Vertebral count	1	2	3	4	5	6	7	8	9	10	11	12	13	14	15	16	17	18	19	20	21	22	23	24	25	26	27	28	29	30	31	32	33	34	35	36	37	38	39	40
NHMUK 1935.6.14:1648 Paralecto	0	2	1	1	1	1	1	1	1	1	1	1	1	1	1	1	1	1	1	2	2	2	–	–	–	–	–	–	–	–	c									
"2" on x-ray 14+17															1	1	1	1	1	2	2	1	–	–	–	–	–	–	–	–	c									
Trematocranus microstoma USNM 227923 a	0	2	1	1	1	1	1	1	1	1	1	1	1	1	1	1	1	1	1	2	1	2	2	1	–	–	–	–	–	–	–	–	c							
14+19															2	–	–	1	1	2	1	2	2	–	–	–	–	–	–	–	–	–	c							
b	0	1	2	1	1	1	1	1	1	1	1	1	1	1	1	1	1	1	1	2	1	2	2	1	–	–	–	–	–	–	–	–	c							
15+18																1	1	1	1	2	1	2	2	–	–	–	–	–	–	–	–	–	c							
c	0	2	1	1	1	1	1	1	1	1	1	1	1	1	1	1	1	1	2	1	2	2	1	–	–	–	–	–	–	–	c									
13+18														1	–	1	1	1	1	2	2	1	–	–	–	–	–	–	–	–	c									
d	0	0-2	1	1	1	1	1	1	1	1	1	1	1	1	1	1	1	1	1	2	1	2	3	–	–	–	–	–	–	–	–	c								
14+18															1	1	–	1	1	2	2	1	2	–	–	–	–	–	–	–	–	c								
AMNH 226033 a	0	2	1	1	1	1	1	1	1	1	1	1	1	1	1	1	1	1	1	2	1	3	–	–	–	–	–	–	–	–	c									
13+18														1	–	1	1	1	2	1	2	2	–	–	–	–	–	–	–	–	c									
b	0	2	1	1	1	1	1	1	1	1	1	1	1	1	1	1	1	1	2	1	2	2	–	–	–	–	–	–	–	–	c									
13+18														1	–	1	1	1	2	2	2	1	–	–	–	–	–	–	–	–	c									
c	0	2	1	1	1	1	1	1	1	1	1	1	1	1	1	1	1	1	1	2	2	2	–	–	–	–	–	–	–	–	c									
13+18														1	–	1	1	1	2	1	3	–	–	–	–	–	–	–	–	–	c									
d	0	2	1	1	1	1	1	1	1	1	1	1	1	1	1	1	1	1	1	2	2	2	–	–	–	–	–	–	–	–	c									
13+18														1	1	–	1	1	2	2	2	1	–	–	–	–	–	–	–	–	c									
e	0	2	1	1	1	1	1	1	1	1	1	1	1	1	1	1	1	1	2	1	2	2	–	–	–	–	–	–	–	c										
14+16															1	1	1	1	2	2	2	–	–	–	–	–	–	–	–	c										
Trematocranus placodon USNM 227921 a	0	2	1	1	1	1	1	1	1	1	1	1	1	1	1	1	1	1	2	1	2	2	–	–	–	–	–	–	–	c										
13+17														1	–	1	1	1	2	1	2	1	–	–	–	–	–	–	–	c										
b	0	2	1	1	1	1	1	1	1	1	1	1	1	1	1	1	1	1	2	1	2	2	1	–	–	–	–	–	–	–	c									
13+18														1	–	1	1	1	2	1	2	1	–	–	–	–	–	–	–	–	c									
c	0	2	1	1	1	1	1	1	1	1	1	1	1	1	1	1	1	1	2	1	2	2	–	–	–	–	–	–	–	–	c									
13+18														1	–	1	1	1	1	2	2	–	–	–	–	–	–	–	–	–	c									
Tyrannochromis macrostoma YPM 014282	0	2	1	1	1	1	1	1	1	1	1	1	1	1	1	1	1	1	1	2	1	2	1	1	–	–	–	–	–	–	–	c								
14+18															1	1	1	1	1	2	2	1	–	–	–	–	–	–	–	–	–	c								
Tyrannochromis nigriventer NHMUK 1956.6.4.6 holotype	0	2	1	1	1	1	1	1	1	1	1	1	1	1	1	1	1	1	1	2	1	2	2	–	–	–	–	–	–	–	–	c								
14+18																																								
**Pseudocrenilabrini: Pseudotropheina**																																								
Abactochromis labrosus NHMUK 1935.6.14:321 Holotype	0	2	1	1	1	1	1	1	1	1	1	1	1	1	1	1	1	1	1	2	2	–?	–	–	–	–	–	–	c											
13+16														1	–	1	1	1	1	2	1?	–	–	–	–	–	–	–	c											
NHMUK unregistered as of 1971	0	2	1	1	1	1	1	1	1	1	1	1	1	1	1	1	1	1	2	2	1	–	–	–	–	–	–	c												
13+15														1	–	1	1	1	1	3	–	–	–	–	–	–	–	c												
YPM 21602	0	2	1	1	1	1	1	1	1	1	1	1	1	1	1	1	1	1	2	1	2	–	–	–	–	–	–	–	c											
13+16														1	1	–	1	1	1	3	–	–	–	–	–	–	–	–	c											
RMCA 164900 on AMNH1	0	2	1	1	1	1	1	1	1	1	1	1	1	1	1	1	1	1	1	2	2	1?	–	–	–	–	–	–	c											
13+16														1	–	1	1	1	1	1	2	–	–	–	–	–	–	–	c											
RMCA 99-041-P-1323 on AMNH1	0	2	1	1	1	1	1	1	1	1	1	1	1	1	1	1	1	1	1	2	2	–	–	–	–	–	–	–	c											
13+16														1	–	1	1	1	2	2	–?	–	–	–	–	–	–	–	c											
RMCA 99-041-P-1364 on AMNH1	0	2	1	1	1	1	1	1	1	1	1	1	1	1	1	1	1	1	1	2	2	1	–	–	–	–	–	–	c											
13+16														1	–	1	–	1	2	1	3	–	–	–	–	–	–	–	c											
Chindongo bellicosus AMNH 31897	0	2	1	1	1	1	1	1	1	1	1	1	1	1	1	1	1	1	1	1	2	1	2	1	–	–	–	–	–	–	–	c								
16+16																	1	1	1	1	1	2	2	–	–	–	–	–	–	–	–	c								
	0	2	1	1	1	1	1	1	1	1	1	1	1	1	1	1	1	1	1	1	1	2	2	1	–	–	–	–	–	–	c									
15+16																2	–	–	1	1	1	2	1	–	–	–	–	–	–	–	c									
YPM 007857	0	2	1	1	1	1	1	1	1	1	1	1	1	1	1	1	1	1	1	1	1	2	2	2	–	–	–	–	–	–	c									
15+16																1	–	1	1	1	1	2	1	–	–	–	–	–	–	–	c									
MKO80-93 Domwe, uncat. ex C&S scan 1	0	2	1	1	1	1	1	1	1	1	1	1	1	1	1	1	1	1	1	1	1	2	2	1	–	–	–	–	–	–	–	c								
15+17																2	–	–	1	1	2	1	2	–	–	–	–	–	–	–	–	c								
2	0	2	1	1	1	1	1	1	1	1	1	1	1	1	1	1	1	1	1	1	1	2	1	3	–	–	–	–	–	–	–	c								
15+17																1	–	1	1	1	1	1	2	2	–	–	–	–	–	–	–	c								
3	0	2	1	1	1	1	1	1	1	1	1	1	1	1	1	1	1	1	1	1	1	2	1	3	–	–	–	–	–	–	–	c								
16+16																	2	–	1	1	1	2	2	–	–	–	–	–	–	–	–	c								
Chindongo minutus AMNH 31900	0	2	1	1	1	1	1	1	1	1	1	1	1	1	1	1	1	1	1	2	2	2	–	–	–	–	–	–	c											
14+15															2	–	–	1	1	2	2	1	–	–	–	–	–	–	c											
Cyathochromis obliquidens AMNH 233482	0	2	1	1	1	1	1	1	1	1	1	1	1	1	1	1	1	1	1	2	1	2	1	–	–	–	–	–	–	–	c									
1 of 2 on x-ray AMNH-5 14+17															1	1	1	–	2	1	2	1?	–	–	–	–	–	–	–	–	c									
	0	2	1	1	1	1	1	1	1	1	1	1	1	1	1	1	1	1	2	2	1?	1?	–	–	–	–	–	–	–	c										
2 of 2 13+17														1	–	1	1	1	1	2	2?	–	–	–	–	–	–	–	–	c										
MKO68-7-31 Monkey Bay, uncat. ex C&S scan	0	2	1	1	1	1	1	1	1	1	1	1	1	1	1	1	1	1	2	1	2	1	–	–	–	–	–	–	–	c										
14+16															1	1	1	1	1	2	2	–	–	–	–	–	–	–	–	c										
YPM 024973	0	2	1	1	1	1	1	1	1	1	1	1	1	1	1	1	1	1	1	2	1	2	–	–	–	–	–	–	–	c										
14+16															1	1	1	1	1	2	1	1	–	–	–	–	–	–	–	c										
Cynotilapia afra USNM 270440	0	2	1	1	1	1	1	1	1	1	1	1	1	1	1	1	1	1	1	1	2	2	1	–	–	–	–	–	–	–	c									
15+16																1	1	1	1	1	2	2	–	–	–	–	–	–	–	–	c									

**Species, Museum, Catalog number / ID**<br>**Vertebral count**	1	2	3	4	5	6	7	8	9	10	11	12	13	14	15	16	17	18	19	20	21	22	23	24	25	26	27	28	29	30	31	32	33	34	35	36	37	38	39	40
Cynotilapia sp. AMNH 31892 1	0	2	1	1	1	1	1	1	1	1	1	1	1	1	1	1	1	1	1	1	2	2	1	–	–	–	–	–	–	c										
15+15																1	1	1	1	1	2	2	–	–	–	–	–	–	–	c										
2	0	2	1	1	1	1	1	1	1	1	1	1	1	1	1	1	1	1	1	1	2	2	1	–	–	–	–	–	–	c										
15+15																1	1	1	1	1	2	2	1	–	–	–	–	–	–	c										
3	0	2	1	1	1	1	1	1	1	1	1	1	1	1	1	1	1	1	1	2	1	3	–	–	–	–	–	–	–	c										
14+16															2	–	–	1	1	2	2	1	–	–	–	–	–	–	–	c										
4	0	2	1	1	1	1	1	1	1	1	1	1	1	1	1	1	1	1	1	2	1	2	–	–	–	–	–	–	c											
14+15															1	1	1	1	1	2	2	–	–	–	–	–	–	–	c											
Genyochromis mento AMNH 31903	0	2	1	1	1	1	1	1	1	1	1	1	1	1	1	1	1	1	1	1	2	1	2	1	–	–	–	–	–	–	c									
15+16																1	1	1	1	1	2	2	–	–	–	–	–	–	–	–	c									
AMNH 220337	0	2	1	1	1	1	1	1	1	1	1	1	1	1	1	1	1	1	1	1	2	2	1	–	–	–	–	–	–	–	c									
14+17															1	1	–	1	1	2	1	2	–	–	–	–	–	–	–	–	c									
AMNH 233496 1	0	2	1	1	1	1	1	1	1	1	1	1	1	1	1	1	1	1	1	2	1	2	1	–	–	–	–	–	–	c										
14+16															1	1	1	1	1	2	1	1	–	–	–	–	–	–	–	c										
2	0	2	1	1	1	1	1	1	1	1	1	1	1	1	1	1	1	1	1	1	2	1	2	–	–	–	–	–	–	–	c									
14+17															1	1	–	1	1	2	1	1	–	–	–	–	–	–	–	–	c									
3	0	2	1	1	1	1	1	1	1	1	1	1	1	1	1	1	1	1	1	1	2	2	1	–	–	–	–	–	–	–	c									
14+17															1	1	–	1	2	1	2	1	–	–	–	–	–	–	–	–	c									
4	0	2	1	1	1	1	1	1	1	1	1	1	1	1	1	1	1	1	1	1	2	2	1	–	–	–	–	–	–	–	c									
14+17															1	1	1	–	2	1	2	1	–	–	–	–	–	–	–	–	c									
5	0	2	1	1	1	1	1	1	1	1	1	1	1	1	1	1	1	1	1	1	2	1	2?	–	–	–	–	–	–	–	c									
14+17															1	1	–	1	1	2	2	1?	–	–	–	–	–	–	–	–	c									
6	0	2	1	1	1	1	1	1	1	1	1	1	1	1	1	1	1	1	1	1	2	1	3	–	–	–	–	–	–	–	c									
14+17															1	1	–	1	1	2	1	2	–	–	–	–	–	–	–	–	c									
MKO80-70 Nkhata B., uncat. ex C&S scan	0	1	2	1	1	1	1	1	1	1	1	1	1	1	1	1	1	1	1	1	1	2	2	1	–	–	–	–	–	–	–	c								
15+17																2	–	1	–	2	1	2	1	–	–	–	–	–	–	–	–	c								
2-part fish	0	2	1	1	1	1	1	1	1	1	1	1	1	1	1	1	1	1	1	1	3	1	1	–	–	–	–	–	–	–	c									
14+17															2	–	–	1	1	2	1	2	–	–	–	–	–	–	–	–	c									
Gephyrochromis lawsi AMNH 31863	0	2	1	1	1	1	1	1	1	1	1	1	1	1	1	1	1	1	1	1	1	2	2	1	–	–	–	–	–	–	c									
15+16																1	1	–	1	1	2	2	1	–	–	–	–	–	–	–	c									
Gephyrochromis moorii MKO80-40 exC&S scan 1	0	2	1	1	1	1	1	1	1	1	1	1	1	1	1	1	1	1	1	1	2	1	3	–	–	–	–	–	–	–	c									
14+17															2	–	–	1	1	2	1	2	–	–	–	–	–	–	–	–	c									
2	0	2	1	1	1	1	1	1	1	1	1	1	1	1	1	1	1	1	1	1	2	1	2	1	–	–	–	–	–	–	–	c								
15+17																1	1	–	1	1	2	1	2	–	–	–	–	–	–	–	–	c								
3	0	2	1	1	1	1	1	1	1	1	1	1	1	1	1	1	1	1	1	1	2	1	2	1	–	–	–	–	–	–	c									
14+17															1	–	1	–	1	2	1	2	1	–	–	–	–	–	–	–	c									
YPM 028230	0	2	1	1	1	1	1	1	1	1	1	1	1	1	1	1	1	1	1	1	2	1	3	–	–	–	–	–	–	–	c									
15+16																1	1	1	1	1	2	2	–	–	–	–	–	–	–	–	c									
Iodotropheus sprengerae USNM 207012 Paratype	0	2	1	1	1	1	1	1	1	1	1	1	1	1	1	1	1	1	1	1	2	1	1	–	–	–	–	–	–	c										
14+16															2	–	–	1	1	1	2	1	–	–	–	–	–	–	–	c										
USNM 207013 Paratype	0	2	1	1	1	1	1	1	1	1	1	1	1	1	1	1	1	1	1	1	2	2	–	–	–	–	–	–	–	c										
14+16															1	–	1	1	1	2	2	–	–	–	–	–	–	–	–	c										
USNM 207014 Paratype	0	2	1	1	1	1	1	1	1	1	1	1	1	1	1	1	1	1	1	1	2	2	1	–	–	–	–	–	–	c										
14+16															1	–	1	1	1	1	2	1	–	–	–	–	–	–	–	c										
USNM 207015 Paratype	0	2	1	1	1	1	1	1	1	1	1	1	1	1	1	1	1	1	1	1	2	2	1	–	–	–	–	–	–	c										
14+16															1	1	–	1	–	1	2	2	–	–	–	–	–	–	–	c										
wild captive via Finley 1972, ex C&S scan	0	2	1	1	1	1	1	1	1	1	1	1	1	1	1	1	1	1	1	2	2	1	–	–	–	–	–	–	c											
14+15															1	1	1	1	1	1	2	2	–	–	–	–	–	–	c											
MCZ 48015	0	2	1	1	1	1	1	1	1	1	1	1	1	1	1	1	1	1	1	2	2	–	–	–	–	–	–	–	c											
14+15															1	1	1	1	1	1	2	1	–	–	–	–	–	–	c											
Labeotropheus fuelleborni AMNH 19128 a	?	2	1	1	1	1	1	1	1	1	1	1	1	1	1	1	1	1	1	1	1	2	2	–	–	–	–	–	–	–	c									
15+16																1	–	1	1	1	2	1	1	–	–	–	–	–	–	–	c									
b	0	2	1	1	1	1	1	1	1	1	1	1	1	1	1	1	1	1	1	1	2	2	1	–	–	–	–	–	–	–	c									
15+16																1	1	1	1	1	2	2	–	–	–	–	–	–	–	–	c									
c	0-1	2	–	1	1	1	1	1	1	1	1	1	1	1	1	1	1	1	1	1	2	2	1	–	–	–	–	–	–	–	c									
15+16																1	1	1	1	1	2	2	–	–	–	–	–	–	–	–	c									
d	0	2	1	1	2*	–	1	1	1	1	1	1	1	1	1	1	1	1	1	1	2	1	2	–	–	–	–	–	–	–	c									
15+16																1	1	1	1	1	1	3	–	–	–	–	–	–	–	–	c									
e	0	2	1	1	1	1	1	1	1	1	1	1	1	1	1	1	1	1	1	1	2	2	1	–	–	–	–	–	–	–	c									
15+16																1	1	1	1	1	2	2	–	–	–	–	–	–	–	–	c									
f	0	2	1	1	1	1	1	1	1	1	1	1	1	1	1	1	1	1	1	1	2	1	2	1	–	–	–	–	–	–	c									
15+16																2	–	–	1	1	2	2	–	–	–	–	–	–	–	–	c									
g	0	2	1	1	1	1	1	1	1	1	1	1	1	1	1	1	1	1	1	1	2	1	2	–	–	–	–	–	–	c										
14+16															1	–	1	1	1	2	1	2	–	–	–	–	–	–	–	c										
AMNH 31894 a	0	2	1	1	1	1	1	1	1	1	1	1	1	1	1	1	1	1	1	1	2	2	2	–	–	–	–	–	–	c										
15+15																1	1	1	1	1	2	2	–	–	–	–	–	–	–	c										
b	0	2	1	1	1	1	1	1	1	1	1	1	1	1	1	1	1	1	1	1	2	2	1	–	–	–	–	–	–	c										
15+15																1	1	1	1	1	2	2	–	–	–	–	–	–	–	c										

Species, Museum, Catalog number / ID<br>Vertebral count	1	2	3	4	5	6	7	8	9	10	11	12	13	14	15	16	17	18	19	20	21	22	23	24	25	26	27	28	29	30	31	32	33	34	35	36	37	38	39	40
c	0	2	1	1	1	1	1	1	1	1	1	1	1	1	1	1	1	1	1	1	2	2	2	–	–	–	–	–	–	–	c									
14+17															1	–	1	1	1	1	2	2	–	–	–	–	–	–	–	–	c									
d	0	2	1	1	1	1	1	1	1	1	1	1	1	1	1	1	1	1	1	2	2	2	–	–	–	–	–	–	–	c										
14+16															1	–	1	1	1	2	2	–	–	–	–	–	–	–	–	c										
AMNH 31906	0	2	1	1	1	1	1	1	1	1	1	1	1	1	1	1	1	1	1	2	1	2	1	–	–	–	–	–	–	c										
15+15																1	1	1	1	1	2	2	–	–	–	–	–	–	–	c										
	0	2	1	1	1	1	1	1	1	1	1	1	1	1	1	1	1	1	1	1	2	2	1	–	–	–	–	–	–	c										
14+16															1	–	1	1	1	1	2	2	–	–	–	–	–	–	–	c										
	0	2	1	1	1	1	1	1	1	1	1	1	1	1	1	1	1	1	1	2	1	2	–	–	–	–	–	–	–	–	c									
14+17															1	–	1	1	1	1	2	2	–	–	–	–	–	–	–	–	c									
	0	2	1	1	1	1	1	1	1	1	1	1	1	1	1	1	1	1	1	1	1	2	2?	–	–	–	–	–	–	–	c									
15+16																2	–	1	1	1	1	2	–	–	–	–	–	–	–	–	c									
Labeotropheus trewavasae AMNH 31890	0	2	1	1	1	1	1	1	1	1	1	1	1	1	1	1	1	1	1	1	1	2	2	1	–	–	–	–	–	–	–	c								
bottom left on xray 2 16+16																	1	1	1	1	1	2	1	–	–	–	–	–	–	–	–	c								
AMNH 31893	0	2	1	1	1	1	1	1	1	1	1	1	1	1	1	1	1	1	1	1	1	2	2	1	–	–	–	–	–	–	–	–	c							
bottom right on xray 2 16+17																	1	1	1	1	1	2	2	–	–	–	–	–	–	–	–	–	c							
AMNH 31899	0	1	2	1	1	1	1	1	1	1	1	1	1	1	1	1	1	1	1	1	1	2	1	2	–	–	–	–	–	–	–	c								
15+17																1	–	1	1	1	1	2	2	–	–	–	–	–	–	–	–	c								
Labidochromis caeruleus	0	2	1	1	1	1	1	1	1	1	1	1	1	1	1	1	1	1	1	1	2	2	1	–	–	–	–	–	–	c										
ex Monkey Bay Fisheries stn colln 14+16															1	–	1	1	1	1	2	1	–	–	–	–	–	–	–	c										
Labidochromis freibergi AMNH 33466 Holotype	0	2	1	1	1	1	1	1	1	1	1	1	1	1	1	1	1	1	1	1	2	2	–	–	–	–	–	–	–	c										
14+16															2	–	–	1	1	2	1	1	–	–	–	–	–	–	–	c										
Labidochromis joanjohnsonae AMNH 33464 Holotype	0	2	1	1	1	1	1	1	1	1	1	1	1	1	1	1	1	1	1	2	2	2	–	–	–	–	–	–	–	c										
13+17														2	–	–	1	1	2	1	2	–	–	–	–	–	–	–	–	c										
Labidochromis pallidus YPM 014259	0	2	1	1	1	1	1	1	1	1	1	1	1	1	1	1	1	1	1	2	2	1	–	–	–	–	–	–	c											
14+15															1	1	–	1	2	1	2	–	–	–	–	–	–	–	c											
8 of these 12 are YPM 014137 L column	0	2	1	1	1	1	1	1	1	1	1	1	1	1	1	1	1	1	1	1	2	2	–	–	–	–	–	–	–	c										
14+16															1	1	1	–	2	1	2	–	–	–	–	–	–	–	–	c										
	0	2	1	1	1	1	1	1	1	1	1	1	1	1	1	1	1	1	1	2	2	1	–	–	–	–	–	–	–	c										
14+16															1	1	–	1	2	1	2	–	–	–	–	–	–	–	–	c										
	0	2	1	1	1	1	1	1	1	1	1	1	1	1	1	1	1	1	1	2	1	3	–	–	–	–	–	–	–	c										
13+17														1	–	1	1	1	1	2	2	–	–	–	–	–	–	–	–	c										
	0	2	1	1	1	1	1	1	1	1	1	1	1	1	1	1	1	1	1	2	2	1	–	–	–	–	–	–	c											
14+15															1	1	1	1	1	1	2	–	–	–	–	–	–	–	c											
	0	2	1	1	1	1	1	1	1	1	1	1	1	1	1	1	1	1	1	2	2	1	–	–	–	–	–	–	c											
13+16														1	–	1	1	1	1	2	1	–	–	–	–	–	–	–	c											
	0	2	1	1	1	1	1	1	1	1	1	1	1	1	1	1	1	1	1	2	1	2	–	–	–	–	–	–	–	c										
14+16															1	1	–	1	2	1	2	–	–	–	–	–	–	–	–	c										
	0	2	1	1	1	1	1	1	1	1	1	1	1	1	1	1	1	1	1	2	2	1	–	–	–	–	–	–	–	c										
14+16															1	1	1	1	1	1	2	–	–	–	–	–	–	–	–	c										
Right column	0	2	1	1	1	1	1	1	1	1	1	1	1	1	1	1	1	1	1	1	2	2	–	–	–	–	–	–	–	c										
14+16															1	–	1	1	1	1	2	1	–	–	–	–	–	–	–	c										
	0	2	1	1	1	1	1	1	1	1	1	1	1	1	1	1	1	1	1	1	2	2	1	–	–	–	–	–	–	c										
14+16															1	–	1	1	1	2	2	–	–	–	–	–	–	–	–	c										
	0	2	1	1	1	1	1	1	1	1	1	1	1	1	1	1	1	1	1	2	1	3	–	–	–	–	–	–	–	c										
14+16															2	–	–	1	1	2	2	–	–	–	–	–	–	–	–	c										
	0	2	1	1	1	1	1	1	1	1	1	1	1	1	1	1	1	1	1	2	2	1	–	–	–	–	–	–	c											
13+16														1	–	1	1	1	1	2	1	–	–	–	–	–	–	–	c											
	0	2	1	1	1	1	1	1	1	1	1	1	1	1	1	1	1	1	1	1	2	2	1?	–	–	–	–	–	–	c										
14+16															1	–	1	1	1	2	1	2	–	–	–	–	–	–	–	c										
uncat. Parle on Plate 6	0	2	1	1	1	1	1	1	1	1	1	1	1	1	1	1	1	1	1	2	1	2	–	–	–	–	–	–	c											
#1 14+15															1	1	–	1	2	1	2	–	–	–	–	–	–	–	c											
	0	2	1	1	1	1	1	1	1	1	1	1	1	1	1	1	1	1	1	1	2	2	1	–	–	–	–	–	–	c										
#2 14+16															1	–	1	1	1	1	2	1	–	–	–	–	–	–	–	c										
	0	2	1	1	1	1	1	1	1	1	1	1	1	1	1	1	1	1	1	2	1	2	–	–	–	–	–	–	c											
#3 14+15															1	1	1	1	–	2	2	–	–	–	–	–	–	–	c											
	0	2	1	1	1	1	1	1	1	1	1	1	1	1	1	1	1	1	1	2	1	2	–	–	–	–	–	–	c											
#4 14+15															1	1	–	1	2	1	2	–	–	–	–	–	–	–	c											
	0	2	1	1	1	1	1	1	1	1	1	1	1	1	1	–	1	1	1	2	2	–	–	–	–	–	–	–	c											
#5 13+16														2	–	–	1	1	2	2	–	–	–	–	–	–	–	–	c											
	0	2	1	1	1	1	1	1	1	1	1	1	1	1	1	1	1	1	1	2	1	3	–	–	–	–	–	–	–	c										
#6 14+16															1	–	1	1	1	2	2	–	–	–	–	–	–	–	–	c										
	0	2	1	1	1	1	1	1	1	1	1	1	1	1	1	1	1	1	1	1	2	2	1	–	–	–	–	–	–	c										
#7 14+16															1	–	1	1	1	2	1	1	–	–	–	–	–	–	–	c										
	0	2	1	1	1	1	1	1	1	1	1	1	1	1	1	1	1	1	1	2	2	1	–	–	–	–	–	–	c											
#8 13+16														2	–	–	1	1	2	1	2	–	–	–	–	–	–	–	c											
	0	2	1	1	1	1	1	1	1	1	1	1	1	1	1	1	1	1	1	2	1	2	–	–	–	–	–	–	–	c										
#9 14+16															1	1	–	1	2	1	2	–	–	–	–	–	–	–	–	c										

Species, Museum, Catalog number / ID<br>Vertebral count	1	2	3	4	5	6	7	8	9	10	11	12	13	14	15	16	17	18	19	20	21	22	23	24	25	26	27	28	29	30	31	32	33	34	35	36	37	38	39	40
	0	2	1	1	1	1	1	1	1	1	1	1	1	1	1	1	1	1	1	1	2	2	–	–	–	–	–	–	–	c										
#10 14+16															1	1	–	1	1	2	1	1	–	–	–	–	–	–	–	c										
Labidochromis shiranus NHMUK 1935.6.14:335	0	2	1	1	1	1	1	1	1	1	1	1	1	1	1	1	1	1	1	1	2	2	1	–	–	–	–	–	–	c										
13+17														1	–	1	1	1	1	2	2	–	–	–	–	–	–	–	–	c										
Labidochromis textilis NHMUK 1975.5.27:9 Holotype	0	2	1	1	1	1	1	1	1	1	1	1	1	1	1	1	1	1	1	2	1	2	–	–	–	–	–	–	–	c										
14+16															2	–	–	1	2	1	2	–	–	–	–	–	–	–	–	c										
AMNH 33465 Paratype	0	2	1	1	1	1	1	1	1	1	1	1	1	1	1	1	1	1	1	1	2	1	2	–	–	–	–	–	–	–	c									
14+17															1	–	1	1	1	1	2	2	–	–	–	–	–	–	–	–	c									
Labidochromis vellicans NHMUK 1935.6.14:326-335 Syntypes	0	2	1	1	1	1	1	1	1	1	1	1	1	1	1	1	1	1	1	1	2	2	1	–	–	–	–	–	–	c										
is revision: some of these are L. maculicauda 14+16															2	–	–	1	1	2	1	2	–	–	–	–	–	–	–	c										
	0	2	1	1	1	1	1	1	1	1	1	1	1	1	1	1	1	1	1	2	1	2	–	–	–	–	–	–	–	c										
14+16															1	1	–	1	1	2	2	–	–	–	–	–	–	–	–	c										
	0	2	1	1	1	1	1	1	1	1	1	1	1	1	1	1	1	1	1	1	2	1	2	–	–	–	–	–	–	c										
14+16															1	–	1	1	1	1	2	1	1	–	–	–	–	–	–	c										
	0	2	1	1	1	1	1	1	1	1	1	1	1	1	1	1	1	1	1	1	2	2	–	–	–	–	–	–	c											
14+15															2	–	1	–	2	1	3	–	–	–	–	–	–	–	c											
	0	2	1	1	1	1	1	1	1	1	1	1	1	1	1	1	1	1	1	1	1	2	2	–	–	–	–	–	–	c										
14+16															1	–	1	1	1	1	2	2	–	–	–	–	–	–	–	c										
	0	2	1	1	1	1	1	1	1	1	1	1	1	1	1	1	1	1	1	1	2	2	1	–	–	–	–	–	–	c										
14+16															1	1	–	1	1	2	1	1	–	–	–	–	–	–	–	c										
NHMUK 1965.10.26:14-21	0	2	1	1	1	1	1	1	1	1	1	1	1	1	1	1	1	1	1	1	2	2	1	–	–	–	–	–	–	c										
14+16															1	–	1	1	1	1	2	1	–	–	–	–	–	–	–	c										
	0	2	1	1	1	1	1	1	1	1	1	1	1	1	1	1	1	1	1	1	2	2	1	–	–	–	–	–	–	c										
14+16															1	1	–	1	1	2	1	2	–	–	–	–	–	–	–	c										
	0	2	1	1	1	1	1	1	1	1	1	1	1	1	1	1	1	1	1	1	2	1	2	–	–	–	–	–	–	c										
14+16															2	–	–	1	1	1	2	2	–	–	–	–	–	–	–	c										
	0	2	1	1	1	1	1	1	1	1	1	1	1	1	1	1	1	1	1	1	2	2	1	–	–	–	–	–	–	–	c									
14+17															1	–	1	1	1	1	1	3	–	–	–	–	–	–	–	–	c									
	0	2	1	1	1	1	1	1	1	1	1	1	1	1	1	1	1	1	1	1	2	1	2	–	–	–	–	–	–	c										
14+16															1	1	–	1	1	2	1	2	–	–	–	–	–	–	–	c										
	0	2	1	1	1	1	1	1	1	1	1	1	1	1	1	1	1	1	1	1	2	2	1	–	–	–	–	–	–	c										
14+16															1	1	–	1	1	1	2	2	–	–	–	–	–	–	–	c										
	0	2	1	1	1	1	1	1	1	1	1	1	1	1	1	1	1	1	1	1	1	2	2	–	–	–	–	–	–	–	c									
14+17															2	–	–	1	1	1	1	2	–	–	–	–	–	–	–	–	c									
	0	2	1	1	1	1	1	1	1	1	1	1	1	1	1	1	1	1	1	1	2	?	?	–	–	–	–	–	–	c										
14+16															1	1	–	1	1	1	2	–?	–	–	–	–	–	–	–	c										
YPM 014268 1	0	2	1	1	1	1	1	1	1	1	1	1	1	1	1	1	1	1	1	2	1	2	–	–	–	–	–	–	–	c										
14+16															1	1	–	1	1	2	2	1	–	–	–	–	–	–	–	c										
2	0	2	1	1	1	1	1	1	1	1	1	1	1	1	1	1	1	1	1	2	2	1	–	–	–	–	–	–	c											
13+16														1	–	1	1	1	1	1	3	–	–	–	–	–	–	–	c											
3	0	2	1	1	1	1	1	1	1	1	1	1	1	1	1	1	1	1	1	1	2	2	1	–	–	–	–	–	–	c										
14+16															1	1	–	1	1	2	2	–	–	–	–	–	–	–	–	c										
4	0	2	1	1	1	1	1	1	1	1	1	1	1	1	1	1	1	1	1	2	1	2	1	–	–	–	–	–	–	c										
14+16															1	1	–	1	2	1	2	1	–	–	–	–	–	–	–	c										
5	0	**3**	1	1	1	1	1	1	1	1	1	1	1	1	1	1	1	1	1	1	2	2	1	–	–	–	–	–	–	c										
14+16															1	1	–	1	1	2	1	2	–	–	–	–	–	–	–	c										
6	0	2	1	1	1	1	1	1	1	1	1	1	1	1	1	1	1	1	1	1	2	2	1	–	–	–	–	–	–	c										
14+16															2	–	–	1	1	1	2	2	–	–	–	–	–	–	–	c										
7	0	2	1	1	1	1	1	1	1	1	1	1	1	1	1	1	1	1	1	1	2	2	1	–	–	–	–	–	–	c										
14+16															1	1	–	1	1	2	1	2	–	–	–	–	–	–	–	c										
8	0	2	1	1	1	1	1	1	1	1	1	1	1	1	1	1	1	1	1	1	2	2	–	–	–	–	–	–	c											
14+15															1	1	–	1	2	1	2	1	–	–	–	–	–	–	c											
9	0	2	1	1	1	1	1	1	1	1	1	1	1	1	1	1	1	1	1	2	1	2	–	–	–	–	–	–	–	c										
13+17														1	–	1	1	1	1	1	3	–	–	–	–	–	–	–	–	c										
10	0	2	1	1	1	1	1	1	1	1	1	1	1	1	1	1	1	1	1	2	1	2	–	–	–	–	–	–	c											
13+16														1	–	1	1	1	1	2	2	–	–	–	–	–	–	–	c											
11	0	2	1	1	1	1	1	1	1	1	1	1	1	1	1	1	1	1	1	2	1	2	1	–	–	–	–	–	–	c										
14+16															2	–	–	1	1	2	2	1	–	–	–	–	–	–	–	c										
12	0	2	1	1	1	1	1	1	1	1	1	1	1	1	1	1	1	1	1	2	2	1	–	–	–	–	–	–	c											
13+16														1	–	1	1	1	1	2	2	–	–	–	–	–	–	–	c											
13	0	2	1	1	1	1	1	1	1	1	1	1	1	1	1	1	1	1	1	2	1	3	–	–	–	–	–	–	–	c										
14+16															1	1	1	1	1	1	2	1	–	–	–	–	–	–	–	c										
14	0	2	1	1	1	1	1	1	1	1	1	1	1	1	1	1	1	1	1	1	2	2	–	–	–	–	–	–	–	c										
14+16															1	1	–	2	1	1	2	–	–	–	–	–	–	–	–	c										
15	0	2	1	1	1	1	1	1	1	1	1	1	1	1	1	1	1	1	1	2	1	2	1	–	–	–	–	–	–	c										
14+16															1	1	–	1	2	1	2	1	–	–	–	–	–	–	–	c										
16	0	2	1	1	1	1	1	1	1	1	1	1	1	1	1	1	1	1	1	2	2	1	–	–	–	–	–	–	c											
13+16														1	–	1	1	1	1	2	1	–	–	–	–	–	–	–	c											

Table 10 (continued). Raw data: Vertebral count and supraneural & dorsal and anal pterygiophore insertion patterns of each specimen

**Species, Museum, Catalog number / ID**<br>**Vertebral count**	1	2	3	4	5	6	7	8	9	10	11	12	13	14	15	16	17	18	19	20	21	22	23	24	25	26	27	28	29	30	31	32	33	34	35	36	37	38	39	40
Maylandia aurora USNM 215292 Holotype	0	2	1	1	1	1	1	1	1	1	1	1	1	1	1	1	1	1	1	1	2	2	1	–	–	–	–	–	–	–	c									
14+17															1	–	1	1	1	1	2	2	–	–	–	–	–	–	–	–	c									
Maylandia koningsi PSU 12891	0	2	1	1	1	1	1	1	1	1	1	1	1	1	1	1	1	1	1	1	2	2	–	–	–	–	–	–	–	c										
15+15																1	1	1	1	1	2	1	–	–	–	–	–	–	–	c										
Maylandia lanisticola USNM 216266 Holotype	0	2	1	1	1	1	1	1	1	1	1	1	1	1	1	1	1	1	1	2	1	2	1	–	–	–	–	–	–	c										
14+16															1	1	–	1	1	2	2	1	–	–	–	–	–	–	–	c										
Maylandia zebra AMNH 19127 7 c1	0	2	1	1	1	1	1	1	1	1	1	1	1	1	1	1	1	1	1	1	2	2	1	–	–	–	–	–	–	c										
15+15																**3**	–	–	1	1	1	3	–	–	–	–	–	–	–	c										
c2	0	2	1	1	1	1	1	1	1	1	1	1	1	1	1	1	1	1	1	1	2	1	2	–	–	–	–	–	–	c										
15+15																1	1	–	1	2	1	2	–	–	–	–	–	–	–	c										
c3	0	2	1	1	1	1	1	1	1	1	1	1	1	1	1	1	1	1	1	1	2	2	1	–	–	–	–	–	–	c										
15+15																1	1	1	1	1	2	2	–	–	–	–	–	–	–	c										
AMNH 31891 7 b	0	2	1	1	1	1	1	1	1	1	1	1	1	1	1	1	1	1	1	1	1	2	2	1	–	–	–	–	–	–	c									
16+15																	1	1	1	1	2	2	1	–	–	–	–	–	–	–	c									
AMNH 31907 7 a	0	2	1	1	1	1	1	1	1	1	1	1	1	1	1	1	1	1	1	1	2	1	3	–	–	–	–	–	–	–	c									
15+16																2	–	–	1	2	1	2	1	–	–	–	–	–	–	–	c									
BB, MKO80-93 Domwe I., uncat. ex C&S scan	0	2	1	1	1	1	1	1	1	1	1	1	1	1	1	1	1	1	1	1	1	2	3	–	–	–	–	–	–	–	c									
15+16																1	–	1	1	1	2	1	2	–	–	–	–	–	–	–	c									
	0	2	1	1	1	1	1	1	1	1	1	1	1	1	1	1	1	1	1	1	2	1	3	–	–	–	–	–	–	–	c									
15+16																2	–	1	1	1	1	2	1	–	–	–	–	–	–	–	c									
	0	2	1	1	1	1	1	1	1	1	1	1	1	1	1	1	1	1	1	1	2	1	2	1	–	–	–	–	–	–	c									
15+16																1	–	1	1	1	2	1	2	–	–	–	–	–	–	–	c									
	0	2	1	1	1	1	1	1	1	1	1	1	1	1	1	1	1	1	1	1	1	1	2	3	–	–	–	–	–	–	–	c								
16+16																	1	1	1	1	1	1	2	1	–	–	–	–	–	–	–	c								
Melanochromis auratus AMNH 31901	0	2	1	1	1	1	1	1	1	1	1	1	1	1	1	1	1	1	1	1	1	2	2	1	–	–	–	–	–	–	c									
15+16																1	1	–	1	2	1	3	–	–	–	–	–	–	–	–	c									
	0	1	2	1	1	1	1	1	1	1	1	1	1	1	1	1	1	1	1	1	2	1	3	–	–	–	–	–	–	–	c									
14+17															1	–	1	1	1	1	2	2	–	–	–	–	–	–	–	–	c									
AMNH 31904	0	2	1	1	1	1	1	1	1	1	1	1	1	1	1	1	1	1	1	1	1	2	3	–	–	–	–	–	–	–	c									
15+16																1	1	1	–	1	2	2	–	–	–	–	–	–	–	–	c									
MKO80-93 Domwe I., uncat. ex C&S scan	0	2	1	1	1	1	1	1	1	1	1	1	1	1	1	1	1	1	1	1	1	2	2	–	–	–	–	–	–	–	c									
14+17															1	–	1	1	1	1	1	3	–	–	–	–	–	–	–	–	c									
	0	2	1	1	1	1	1	1	1	1	1	1	1	1	1	1	1	1	1	1	1	2	2	1	–	–	–	–	–	–	c									
15+16																1	–	1	1	1	1	2	1	–	–	–	–	–	–	–	c									
NHMUK 1896.12.1.7 holotype	0	2	1	1	1	1	1	1	1	1	1	1	1	1	1	1	1	1	1	1	1	2	2	1	–	–	–	–	–	–	–	c								
15+17																1	1	–	1	1	2	2	–	–	–	–	–	–	–	–	–	c								
Melanochromis chipokae USNM 214173 holotype	0	2	1	1	1	1	1	1	1	1	1	1	1	1	1	1	1	1	1	1	1	2	2	–	–	–	–	–	–	–	c									
15+16																1	1	1	1	1	1	2	1	–	–	–	–	–	–	–	c									
Melanochromis loriae USNM 214175 holotype	0	2	1	1	1	1	1	1	1	1	1	1	1	1	1	1	1	1	1	1	1	2	2	1	–	–	–	–	–	–	c									
15+16																1	–	1	1	1	2	1	2	–	–	–	–	–	–	–	c									
USNM 214176 paratype	0	2	1	1	1	1	1	1	1	1	1	1	1	1	1	1	1	1	1	1	2	1	3	–	–	–	–	–	–	–	c									
15+16																1	1	–	1	1	2	2	–	–	–	–	–	–	–	–	c									
USNM 215029	0	2	1	1	1	1	1	1	1	1	1	1	1	1	1	1	1	1	1	1	2	1	2	2?	–	–	–	–	–	–	c									
15+16																1	1	–	1	1	2	2	1?	–	–	–	–	–	–	–	c									
USNM 215030	0	2	1	1	1	1	1	1	1	1	1	1	1	1	1	1	1	1	1	1	2	1	3	–	–	–	–	–	–	–	c									
15+16																1	1	1	–	2	1	2	–	–	–	–	–	–	–	–	c									
Melanochromis melanopterus AMNH 31889	0	2	1	1	1	1	1	1	1	1	1	1	1	1	1	1	1	1	1	1	1	2	2	1	–	–	–	–	–	–	–	c								
left 15+17																1	–	1	1	1	1	2	2	–	–	–	–	–	–	–	–	c								
	0	2	1	1	1	1	1	1	1	1	1	1	1	1	1	1	1	1	1	1	1	2	2?	1?	–	–	–	–	–	–	–	c								
right 15+17																1	–	1	1	1	1	2?	1?	–?	–	–	–	–	–	–	–	c								
	0	1	2	1	1	1	1	1	1	1	1	1	1	1	1	1	1	1	1	1	2	1	2	1	–	–	–	–	–	–	c									
big 15+16																2	–	–	1	1	2	1	2	–	–	–	–	–	–	–	c									
AMNH 226068	0	2	1	1	1	1	1	1	1	1	1	1	1	1	1	1	1	1	1	1	1	2	2	1	–	–	–	–	–	–	–	c								
1 of 2 on AMNH-5 15+17																1	–	1	1	1	1	2	2	–	–	–	–	–	–	–	–	c								
	0	2	1	1	1	1	1	1	1	1	1	1	1	1	1	1	1	1	1	1	2	2	1?	–	–	–	–	–	–	–	c									
2 of 2 15+16																1	1	1	–	2	1	2	1?	–	–	–	–	–	–	–	c									
RMCA 99-41-P-1345-353	0	2	1	1	1	1	1	1	1	1	1	1	1	1	1	1	1	1	1	1	1	2	3	–	–	–	–	–	–	–	–	c								
15+17																1	–	1	1	1	2	1	1	–	–	–	–	–	–	–	–	c								
	0	2	1	1	1	1	1	1	1	1	1	1	1	1	1	1	1	1	1	1	2	2	1	–	–	–	–	–	–	–	c									
15+16																1	1	–	1	2	1	2	–	–	–	–	–	–	–	–	c									
MKO80-93 Domwe I., uncat. ex C&S scan 1	0	1	2	1	1	1	1	1	1	1	1	1	1	1	1	1	1	1	1	1	1	2	2	1	–	–	–	–	–	–	–	c								
15+17																1	–	1	1	1	1	2	3	–	–	–	–	–	–	–	–	c								
2	0	2	1	1	1	1	1	1	1	1	1	1	1	1	1	1	1	1	1	1	2	1	2	1	–	–	–	–	–	–	c									
15+16																1	1	–	1	1	2	1	2	–	–	–	–	–	–	–	c									
3	0	2	1	1	1	1	1	1	1	1	1	1	1	1	1	1	1	1	1	1	1	2	3	1	–	–	–	–	–	–	–	c								
15+17																2	–	–	1	1	1	1	2	1	–	–	–	–	–	–	–	c								
YPM 014310	0	2	1	1	1	1	1	1	1	1	1	1	1	1	1	1	1	1	1	1	1	2	2	–	–	–	–	–	–	–	c									
15+16																1	1	–	1	1	2	2	–	–	–	–	–	–	–	–	c									

Species, Museum, Catalog number / ID Vertebral count	1	2	3	4	5	6	7	8	9	10	11	12	13	14	15	16	17	18	19	20	21	22	23	24	25	26	27	28	29	30	31	32	33	34	35	36	37	38	39	40
Melanochromis simulans USNM 210701 Paratype	0	2	1	1	1	1	1	1	1	1	1	1	1	1	1	1	1	1	1	1	1	2	2	–?	–	–	–	–	–	–	c									
15+16																1	1	1	–	2	1	2	1	–	–	–	–	–	–	–	c									
Melanochromis cf. *vermivorus* AMNH 31896	0	2	1	1	1	1	1	1	1	1	1	1	1	1	1	1	1	1	1	1	2	2?	1?	–	–	–	–	–	–	–	c									
15+16																2	–	–	1	1	2?	1?	–	–	–	–	–	–	–	–	c									
	0	1	2	1	1	1	1	1	1	1	1	1	1	1	1	1	1	1	1	1	2	1	2	–	–	–	–	–	–	–	c									
14+17															1	–	1	1	1	1	2	2	–	–	–	–	–	–	–	–	c									
Petrotilapia genalutea YPM 014173	0	2	1	1	1	1	1	1	1	1	1	1	1	1	1	1	1	1	1	1	1	2	2	1	–	–	–	–	–	–	c									
15+16																1	1	–	1	1	2	1	2	–	–	–	–	–	–	–	c									
Petrotilapia nigra YPM 014591	0	2	1	1	1	1	1	1	1	1	1	1	1	1	1	1	1	1	1	1	2	2	1	–	–	–	–	–	–	–	c									
14+17															1	–	1	1	1	1	2	2	–	–	–	–	–	–	–	–	c									
Petrotilapia tridentiger YPM 007854	0	2	1	1	1	1	1	1	1	1	1	1	1	1	1	1	1	1	1	1	2	1	3	–	–	–	–	–	–	–	c									
15+16																1	1	1	1	1	2	2	–	–	–	–	–	–	–	–	c									
Petrotilapia sp. AMNH 19131	0	2	1	1	1	1	1	1	1	1	1	1	1	1	1	1	1	1	1	1	2	1	3	–	–	–	–	–	–	–	c									
15+16																1	1	–	1	2	1	2	1	–	–	–	–	–	–	–	c									
AMNH 31888	0	2	1	1	1	1	1	1	1	1	1	1	1	1	1	1	1	1	1	1	1	2	2	1	–	–	–	–	–	–	c									
15+16																1	–	1	1	1	2	1	2	–	–	–	–	–	–	–	c									
AMNH 31895	0	2	1	1	1	1	1	1	1	1	1	1	1	1	1	1	1	1	1	2	1	3	–	–	–	–	–	–	–	c										
15+15																1	1	1	1	2	1	2	–	–	–	–	–	–	–	c										
AMNH 31905	0	2	1	1	1	1	1	1	1	1	1	1	1	1	1	1	1	1	1	1	1	2	2	1	–	–	–	–	–	–	c									
15+16																1	–	1	1	1	2	1	2	–	–	–	–	–	–	–	c									
Pseudotropheus elegans NHMUK 1935.6.14.127 holotype	0	2	1	1	1	1	1	1	1	1	1	1	1	1	1	1	1	1	1	1	2	1	2	1	–	–	–	–	–	–	c									
14+17															1	–	1	1	1	1	2	2	–	–	–	–	–	–	–	–	c									
Pseudotropheus interruptus USNM 214221 holotype	0	2	1	1	1	1	1	1	1	1	1	1	1	1	1	1	1	1	1	1	2	1	2	1	–	–	–	–	–	–	c									
15+16																1	1	–	1	1	2	2	–	–	–	–	–	–	–	–	c									
USNM 214222 paratype	0	2	1	1	1	1	1	1	1	1	1	1	1	1	1	1	1	1	1	1	1	2	2	–	–	–	–	–	–	–	c									
14+17															1	–	1	1	1	1	2	2	–	–	–	–	–	–	–	–	c									
Pseudotropheus johannii USNM 210702 Holotype	0	2	1	1	1	1	1	1	1	1	1	1	1	1	1	1	1	1	1	1	1	2	1	2	–	–	–	–	–	–	–	c								
16+16																	1	1	1	1	1	2	2	–	–	–	–	–	–	–	–	c								
USNM 210701 Paratype	0	2	1	1	1	1	1	1	1	1	1	1	1	1	1	1	1	1	1	1	1	2	1	2	–	–	–	–	–	–	c									
16+15																	2	–	1	1	1	2	2	–	–	–	–	–	–	–	c									
AMNH 215563 1	0	2	1	1	1	1	1	1	1	1	1	1	1	1	1	1	1	1	1	1	2	1	3	–	–	–	–	–	–	c										
14+16															1	–	1	1	–	2	1	2	1	–	–	–	–	–	–	c										
2	0	2	1	1	1	1	1	1	1	1	1	1	1	1	1	1	1	1	1	1	2	1	3	–	–	–	–	–	–	c										
14+16															1	–	1	1	1	1	2	2	–	–	–	–	–	–	–	c										
3	0	2	1	1	1	1	1	1	1	1	1	1	1	1	1	1	1	1	1	1	1	2	2	1	–	–	–	–	–	–	c									
15+16																2	–	–	1	1	2	1	2	–	–	–	–	–	–	–	c									
4	0	2	1	1	1	1	1	1	1	1	1	1	1	1	1	1	1	1	1	1	2	1	2	1	–	–	–	–	–	–	c									
15+16																1	1	1	1	1	1	2	1	–	–	–	–	–	–	–	c									
5	0	2	1	1	1	1	1	1	1	1	1	1	1	1	1	1	1	1	1	1	1	2	1	3	–	–	–	–	–	–	c									
15+16																1	–	1	1	1	1	1	3	–	–	–	–	–	–	–	c									
6	0	2	1	1	1	1	1	1	1	1	1	1	1	1	1	1	1	1	1	1	2	1	2	–	–	–	–	–	–	–	c									
15+16																1	1	1	1	1	2	2	–	–	–	–	–	–	–	–	c									
7	0	2	1	1	1	1	1	1	1	1	1	1	1	1	1	1	1	1	1	1	1	2	2	–	–	–	–	–	–	–	c									
15+16																1	1	1	1	1	2	1	1	–	–	–	–	–	–	–	c									
8	0	2	1	1	1	1	1	1	1	1	1	1	1	1	1	1	1	1	1	1	1	2	1	2	–	–	–	–	–	–	–	c								
15+17																1	–	1	1	1	1	1	3	–	–	–	–	–	–	–	–	c								
9	0	2	1	1	1	1	1	1	1	1	1	1	1	1	1	1	1	1	1	1	1	2	2	1	–	–	–	–	–	–	c									
15+16																1	–	1	1	1	2	2	1	–	–	–	–	–	–	–	c									
10	0	2	1	1	1	1	1	1	1	1	1	1	1	1	1	1	1	1	1	1	1	2	2	1	–	–	–	–	–	–	c									
15+16																1	–	1	1	1	1	2	2	–	–	–	–	–	–	–	c									
11	0	2	1	1	1	1	1	1	1	1	1	1	1	1	1	1	1	1	1	1	1	2	2	1	–	–	–	–	–	c										
15+15																1	1	–	1	2	1	2	1	–	–	–	–	–	–	c										
12	0	2	1	1	1	1	1	1	1	1	1	1	1	1	1	1	1	1	1	1	1	2	2	–	–	–	–	–	–	–	c									
15+16																1	1	1	–	2	1	2	1	–	–	–	–	–	–	–	c									
13	0	2	1	1	1	1	1	1	1	1	1	1	1	1	1	1	1	1	1	1	1	2	2	1	–	–	–	–	–	–	c									
14+17															1	–	1	1	1	1	1	2	1	–	–	–	–	–	–	–	c									
14	0	2	1	1	1	1	1	1	1	1	1	1	1	1	1	1	1	1	1	1	1	2	2	1	–	–	–	–	–	–	c									
14+17															1	–	1	1	1	1	1	2	2	–	–	–	–	–	–	–	c									
15	0	2	1	1	1	1	1	1	1	1	1	1	1	1	1	1	1	1	1	1	1	2	1	3	–	–	–	–	–	–	c									
15+16																2	–	–	1	1	1	1	3	–	–	–	–	–	–	–	c									
16	0	2	1	1	1	1	1	1	1	1	1	1	1	1	1	1	1	1	1	1	1	2	2	1	–	–	–	–	–	–	c									
15+16																1	1	–	1	1	2	2	–	–	–	–	–	–	–	–	c									
17	0	2	1	1	1	1	1	1	1	1	1	1	1	1	1	1	1	1	1	1	2	1	2	1	–	–	–	–	–	–	c									
15+16																1	–	1	1	1	2	1	2	–	–	–	–	–	–	–	c									
18	0	1	1	1	1	1	1	1	1	1	1	1	1	1	1	1	1	1	1	1	1	1	2	2	1	–	–	–	–	–	–	c								
16+16																	1	1	1	–	2	1	2	1	–	–	–	–	–	–	–	c								

Species, Museum, Catalog number / ID<br>Vertebral count	1	2	3	4	5	6	7	8	9	10	11	12	13	14	15	16	17	18	19	20	21	22	23	24	25	26	27	28	29	30	31	32	33	34	35	36	37	38	39	40
19	0	2	1	1	1	1	1	1	1	1	1	1	1	1	1	1	1	1	1	1	2	1	2	1	–	–	–	–	–	–	c									
14+17															1	–	1	1	1	1	2	1	1	–	–	–	–	–	–	–	c									
20	0	2	1	1	1	1	1	1	1	1	1	1	1	1	1	1	1	1	1	1	1	2	2	1	–	–	–	–	–	–	c									
15+16																1	1	–	1	1	2	1	2	–	–	–	–	–	–	–	c									
21	0	2	1	1	1	1	1	1	1	1	1	1	1	1	1	1	1	1	1	1	1	2	1	2	–	–	–	–	–	–	c									
15+16																1	1	1	1	1	1	2	1	–	–	–	–	–	–	–	c									
22	0	2	1	1	1	1	1	1	1	1	1	1	1	1	1	1	1	1	1	1	1	2	2	1	–	–	–	–	–	–	c									
15+16																2	–	–	1	1	1	2	2	–	–	–	–	–	–	–	c									
23	0	2	1	1	1	1	1	1	1	1	1	1	1	1	1	1	1	1	1	1	1	2	1	2	–	–	–	–	–	–	c									
16+15																1	1	1	1	1	1	3	–	–	–	–	–	–	–	–	c									
24	0	2	1	1	1	1	1	1	1	1	1	1	1	1	1	1	1	1	1	1	1	2	2	–	–	–	–	–	–	–	c									
15+16																1	–	1	1	1	2	2	–	–	–	–	–	–	–	–	c									
25	0	2	1	1	1	1	1	1	1	1	1	1	1	1	1	1	1	1	1	1	1	2	1	2	–	–	–	–	–	–	c									
15+16																1	–	1	1	1	1	2	2	–	–	–	–	–	–	–	c									
26	0	2	1	1	1	1	1	1	1	1	1	1	1	1	1	1	1	1	1	1	1	2	2	1	–	–	–	–	–	–	–	c								
15+17																1	1	–	1	1	2	1	2	–	–	–	–	–	–	–	–	c								
27	0	2	1	1	1	1	1	1	1	1	1	1	1	1	1	1	1	1	1	1	2	1	3	–	–	–	–	–	–	c										
14+16															1	–	1	1	1	1	2	2	–	–	–	–	–	–	–	c										
28	0	2	1	1	1	1	1	1	1	1	1	1	1	1	1	1	1	1	1	1	1	2	1	2	–	–	–	–	–	–	c									
15+16																1	1	–	1	1	1	2	2	–	–	–	–	–	–	–	c									
29	0	2	1	1	1	1	1	1	1	1	1	1	1	1	1	1	1	1	1	1	2	1	3	–	–	–	–	–	–	–	c									
14+17															2	–	–	1	1	1	1	2	–	–	–	–	–	–	–	–	c									
30	0	2	1	1	1	1	1	1	1	1	1	1	1	1	1	1	1	1	1	1	1	2	2	1	–	–	–	–	–	c										
15+15																1	1	–	1	2	1	2	1	–	–	–	–	–	–	c										
31	0	2	1	1	1	1	1	1	1	1	1	1	1	1	1	1	1	1	1	1	1	2	2	1	–	–	–	–	–	–	c									
15+16																1	1	–	1	2	1	2	1	–	–	–	–	–	–	–	c									
32	0	2	1	1	1	1	1	1	1	1	1	1	1	1	1	1	1	1	1	1	2	3	–	–	–	–	–	–	–	c										
14+16															2	–	–	1	1	2	1	2	–	–	–	–	–	–	–	c										
dark male 33	0	2	1	1	1	1	1	1	1	1	1	1	1	1	1	1	1	1	1	1	2	1	3	–	–	–	–	–	–	–	c									
14+17															1	–	1	1	–	2	1	3	–	–	–	–	–	–	–	–	c									
dark male 34	0	2	1	1	1	1	1	1	1	1	1	1	1	1	1	1	1	1	1	1	1	2	2	1	–	–	–	–	–	–	c									
15+16																1	1	1	–	1	2	2	1	–	–	–	–	–	–	–	c									
dark male 35	0	2	1	1	1	1	1	1	1	1	1	1	1	1	1	1	1	1	1	1	1	2	2	–	–	–	–	–	–	c										
15+15																1	1	1	1	1	2	2	–	–	–	–	–	–	–	c										
Pseudotropheus livingstonii AMNH 226057	0	2	1	1	1	1	1	1	1	1	1	1	1	1	1	1	1	1	1	1	2	1	3	–	–	–	–	–	–	–	c									
14+17															1	1	–	1	1	2	1	2	–	–	–	–	–	–	–	–	c									
	0	2	1	1	1	1	1	1	1	1	1	1	1	1	1	1	1	1	1	1	2	1	2	2	–	–	–	–	–	–	–	c								
15+17																1	1	1	–	2	1	2	2	–	–	–	–	–	–	–	–	c								
AMNH 221238 At left	0	2	1	1	1	1	1	1	1	1	1	1	1	1	1	1	1	1	1	1	2	2	1	–	–	–	–	–	–	–	c									
14+17															1	–	1	1	1	1	2	2	–	–	–	–	–	–	–	–	c									
Top of column of three	0	2	1	1	1	1	1	1	1	1	1	1	1	1	1	1	1	1	1	1	2	2	1	–	–	–	–	–	–	–	c									
14+17															1	–	1	1	1	2	2	1	–	–	–	–	–	–	–	–	c									
Mid of column of three	0	2	1	1	1	1	1	1	1	1	1	1	1	1	1	1	1	1	1	1	2	2	1	–	–	–	–	–	–	–	c									
14+16															1	–	1	1	1	1	2	2	–	–	–	–	–	–	–	–	c									
Bottom of column of three	0	2	1	1	1	1	1	1	1	1	1	1	1	1	1	1	1	1	1	2	1	2?	–	–	–	–	–	–	–	–	c									
14+17															1	1	–	1	1	2	1	2	–	–	–	–	–	–	–	–	c									
NHMUK 1863.11.12.22 holotype	0	2	1	1	1	1	1	1	1	1	1	1	1	1	1	1	1	1	1	2	1	2	1	–	–	–	–	–	–	–	c									
14+17															2	–	–	1	1	1	2	2?	–	–	–	–	–	–	–	–	c									
Pseudotropheus lucerna AMNH 31898	0	2	1	1	1	1	1	1	1	1	1	1	1	1	1	1	1	1	1	1	2	1	3	–	–	–	–	–	–	–	–	c								
15+17																1	1	–	1	1	2	2	–	–	–	–	–	–	–	–	–	c								
Tropheops tropheops CUMV 89948 on AMNH-3	0	2	1	1	1	1	1	1	1	1	1	1	1	1	1	1	1	1	1	1	1	2	2	1	–	–	–	–	–	c										
#117 15+15 ↗																1	1	–	1	1	2	3	–	–	–	–	–	–	–	c										
#43 15+16	0	2	1	1	1	1	1	1	1	1	1	1	1	1	1	1	1	1	1	1	1	2	2	2	–	–	–	–	–	–	c									
																1	–	1	1	1	1	2	1	1	–	–	–	–	–	–	c									
#4 15+16	0	2	1	1	1	1	1	1	1	1	1	1	1	1	1	1	1	1	1	1	2	1	3	–	–	–	–	–	–	–	c									
																1	1	1	–	2	1	2	1	–	–	–	–	–	–	–	c									
#82 15+16	0	2	1	1	1	1	1	1	1	1	1	1	1	1	1	1	1	1	1	1	1	2	3	–	–	–	–	–	–	–	c									
																1	1	–	1	1	2	2	1	–	–	–	–	–	–	–	c									
#131 16+15	0	2	1	1	1	1	1	1	1	1	1	1	1	1	1	1	1	1	1	1	1	2	1	3	–	–	–	–	–	–	c									
																	1	1	1	–	2	1	2	1	–	–	–	–	–	–	c									
#93 15+16	0	2	1	1	1	1	1	1	1	1	1	1	1	1	1	1	1	1	1	1	1	2	2	1	–	–	–	–	–	–	c									
																1	–	1	1	1	1	2	2	–	–	–	–	–	–	–	c									
#55 16+15	0	2	1	1	1	1	1	1	1	1	1	1	1	1	1	1	1	1	1	1	2	1	2	1	–	–	–	–	–	–	c									
																	1	1	1	1	1	1	3	–	–	–	–	–	–	–	c									

Species, Museum, Catalog number / ID<br>Vertebral count	1	2	3	4	5	6	7	8	9	10	11	12	13	14	15	16	17	18	19	20	21	22	23	24	25	26	27	28	29	30	31	32	33	34	35	36	37	38	39	40
**Pseudocrenilabrini: Rhamphochromina**																1	1	1	1	2	2	1	2	–	–	–	–	–	–	–	–	–	c							
Diplotaxodon argenteus AMNH 221945	0	2	1	1	1	1	1	1	1	1	1	1	1	1	1	1	1	1	2	1	2	2	1	–	–	–	–	–	–	–	–	–	–	c						
17+17																		3	–	1	2	2	1	3	–	–	–	–	–	–	–	–	–	c						
	0	2	1	1	1	1	1	1	1	1	1	1	1	1	1	1	1	1	2	1	2	2	1	–	–	–	–	–	–	–	–	–	–	c						
17+17																		3	–	1	2	2	2	3	–	–	–	–	–	–	–	–	–	c						
	0	1	2	1	1	1	1	1	1	1	1	1	1	1	1	1	1	1	1	1	2	1	2	2	1	–	–	–	–	–	–	–	–	–	–	c				
18+18																			3	2	1	2	1	2	3	–	–	–	–	–	–	–	–	–	–	c				
	0	1	2	1	1	1	1	1	1	1	1	1	1	1	1	1	1	1	1	2	1	2	2	–	–	–	–	–	–	–	–	–	–	c						
17+17																		3	1	2	2	2	2	–	–	–	–	–	–	–	–	–	–	c						
AMNH 222088 a	0	–	3	1	1	1	1	1	1	1	1	1	1	1	1	1	1	1	1	2	1	2	2	1	–	–	–	–	–	–	–	–	–	c						
17+17																		3	1	1	2	2	3	–	–	–	–	–	–	–	–	–	–	c						
b	0	1	2	1	1	1	1	1	1	1	1	1	1	1	1	1	1	1	1	1	2	1	2	2	–	–	–	–	–	–	–	–	–	–	–	c				
18+18																			2	2	1	2	2	2	2	–	–	–	–	–	–	–	–	–	–	c				
c	0	1	1	2	1	1	1	1	1	1	1	1	1	1	1	1	1	1	1	2	1	1	2	1	2	–	–	–	–	–	–	–	–	–	–	c				
18+18																			3	1	1	2	2	2	1	–	–	–	–	–	–	–	–	–	–	c				
d	0	–	3	1	1	1	1	1	1	1	1	1	1	1	1	1	1	1	1	2	1	2	2	1	–	–	–	–	–	–	–	–	–	–	–	c				
18+18																			3	1	2	2	2	2	–	–	–	–	–	–	–	–	–	–	–	c				
NHMUK 1935.6.14.2281–2282 Lectotype	0	2	1	1	1	1	1	1	1	1	1	1	1	1	1	1	1	1	2	1	2	2	1	–	–	–	–	–	–	–	–	–	–	c						
16+18																	3	–	2	2	1	2	2	–	–	–	–	–	–	–	–	–	–	c						
paralectotype	0	1	2	1	1	1	1	1	1	1	1	1	1	1	1	1	1	1	2	1	2	3	–	–	–	–	–	–	–	–	–	–	–	c						
16+18																	3	–	2	1	2	2	2	–	–	–	–	–	–	–	–	–	–	c						
Diplotaxodon ecclesi USNM 210696 Holotype	0	2	1	1	1	1	1	1	1	1	1	1	1	1	1	1	1	1	2	1	2	2	1	–	–	–	–	–	–	–	–	–	–	c						
16+18																	3	1	1	2	2	2	2	–	–	–	–	–	–	–	–	–	–	c						
Diplotaxodon greenwoodi USNM 270847 Holotype	0	2	1	1	1	1	1	1	1	1	1	1	1	1	1	1	1	2	1	2	2	1	–	–	–	–	–	–	–	–	–	–	c							
15+18																3	1	1	2	1	2	2	–	–	–	–	–	–	–	–	–	–	c							
Diplotaxodon limnothrissa YPM 26569 1	0	1	2	1	1	1	1	1	1	1	1	1	1	1	1	1	1	1	1	2	1	1	2	2	1	–	–	–	–	–	–	–	–	–	–	c				
top↘bot L, top↘bot R 18+18																			3	1	1	2	1	2	2	–	–	–	–	–	–	–	–	–	–	c				
2	0	1	2	1	1	1	1	1	1	1	1	1	1	1	1	1	1	1	1	2	1	1	2	2	1?	–	–	–	–	–	–	–	–	–	–	c				
18+18																			3	2	1	2	2	1?	–	–	–	–	–	–	–	–	–	–	–	c				
3	0	1	1	2	1	1	1	1	1	1	1	1	1	1	1	1	1	1	1	1	2	1	2	2	1?	–	–	–	–	–	–	–	–	–	c					
18+17																			3	2	1	2	2	2	–?	–	–	–	–	–	–	–	–	–	c					
4	0	1	2	1	1	1	1	1	1	1	1	1	1	1	1	1	1	1	1	1	2	1	1	2	2	–?	–	–	–	–	–	–	–	–	–	–	c			
18+19																			1	3	1	1	2	2	3	–	–	–	–	–	–	–	–	–	–	–	c			
5	0	–	3	1	1	1	1	1	1	1	1	1	1	1	1	1	1	1	1	2	1	2	1	2?	–?	–	–	–	–	–	–	–	–	–	–	c				
17+19																		1	2	2	2	2	2	1?	–	–	–	–	–	–	–	–	–	–	–	c				
6	0	–	3	1	1	1	1	1	1	1	1	1	1	1	1	1	1	1	1	1	2	1	2	2	–	–	–	–	–	–	–	–	–	–	–	c				
18+18																			3	1	2	1	2	2	1	–	–	–	–	–	–	–	–	–	–	c				
7	0	–	2	2	1	1	1	1	1	1	1	1	1	1	1	1	1	1	1	1	2	1	2	1	2	–	–	–	–	–	–	–	–	–	–	c				
18+18																			4	–	1	2	1	2	2	–	–	–	–	–	–	–	–	–	–	c				
8	0	1	2	1	1	1	1	1	1	1	1	1	1	1	1	1	1	1	1	1	2	1	2	2	–	–	–	–	–	–	–	–	–	–	–	c				
17+19																		3	–	2	1	2	1	2	–	–	–	–	–	–	–	–	–	–	–	c				
9	0	–	3	1	1	1	1	1	1	1	1	1	1	1	1	1	1	1	1	2	1	1	2	1	2	–	–	–	–	–	–	–	–	–	–	c				
18+18																			3	1	2	1	2	2	2?	–	–	–	–	–	–	–	–	–	–	c				
10	0	1	2	1	1	1	1	1	1	1	1	1	1	1	1	1	1	1	1	1	2	1	2	2	1	–	–	–	–	–	–	–	–	–	c					
18+17															1	1	1	1	2	1	3	1	–	–	–	–	–	–	–	c										
Pallidochromis tokolosh YPM 26900 top↘bot	0	1	2	1	1	1	1	1	1	1	1	1	1	1	1	1	1	2	1	2	2	2	–	–	–	–	–	–	–	–	–	–	–	c						
15+19																2	2	1	2	2	2	1	–	–	–	–	–	–	–	–	–	–	–	c						
	0	2	1	1	1	1	1	1	1	1	1	1	1	1	1	1	2	1	2	3	–	–	–	–	–	–	–	–	–	–	–	–	c							
14+19															2	1	1	2	2	2	2	1	–	–	–	–	–	–	–	–	–	–	c							
	0	–	3	1	1	1	1	1	1	1	1	1	1	1	1	1	1	1	2	1	2	2	–	–	–	–	–	–	–	–	–	–	–	c						
15+19																2	1	2	1	2	2	2	–	–	–	–	–	–	–	–	–	–	–	c						
	0	–	3	1	1	1	1	1	1	1	1	1	1	1	1	1	1	1	2	1	2	2	1	–	–	–	–	–	–	–	–	–	–	c						
15+19															1	1	1	1	1	2	2	2	–	–	–	–	–	–	–	–	c									
Rhamphochromis brevis NHMUK 1935.6.14.2182 lectotype	0	2	1	1	1	1	1	1	1	1	1	1	1	1	1	1	1	1	1	1	1	2	1	1	2	2	–	–	–	–	–	–	–	–	–	c				
17+19																		–	2	1	1	2	1	2	2	–?	–	–	–	–	–	–	–	–	–	c				
NHMUK 1935.6.14.2183 paralectotype	0	1	2	1	1	1	1	1	1	1	1	1	1	1	1	1	1	1	1	1	2	1	2	2	1	–	–	–	–	–	–	–	–	–	–	c				
16+20																	2	–	1	1	2	2	1	3	1	–	–	–	–	–	–	–	–	–	–	c				
Rhamphochromis esox AMNH 233506	0	1	2	1	1	1	1	1	1	1	1	1	1	1	1	1	1	1	1	1	1	2	1	1	2	2	1	–	–	–	–	–	–	–	–	–	–	–	c	
18+21																			2	–	1	2	1	2	2	1	–	–	–	–	–	–	–	–	–	–	–	–	c	
	–	2	1	1	1	1	1	1	1	1	1	1	1	1	1	1	1	1	1	1	1	1	1	2	1	2	2	1	–	–	–	–	–	–	–	–	–	–	–	c
18+22																			2	–	1	1	2	1	2	2	1	–	–	–	–	–	–	–	–	–	–	–	–	c
Rhamphochromis woodi YPM 032044	0	–	3	1	1	1	1	1	1	1	1	1	1	1	1	1	1	1	1	1	2	1?	1?	2?	2?	–	–	–	–	–	–	–	–	–	–	–	c			
17+20																		1	1	1	2	1	2	3	–	–	–	–	–	–	–	–	–	–	–	–	c			
