# Supplementary material for: African cichlid fishes: morphological data and taxonomic insights from a genus-level survey of supraneurals, pterygiophores, and vertebral counts (Ovalentaria, Blenniiformes, Cichlidae, Pseudocrenilabrinae)
Source: Biodivers Data J. 2024 Oct 18;12:e130707. doi: 10.3897/BDJ.12.e130707 (PMC11512106; doi:10.3897/BDJ.12.e130707)
Supplement: Supplementary material 12 — Specimens examined and information sources [file bdj-12-e130707-s012.pdf]

Specimens examined and information sources

Note: Locality, collection date, and collector(s) are given where available from my records or online sources. Species collected from a lake but occurring also in rivers (or with synonymous species in rivers) are listed as riverine. Radiographs listed without a published or online source are either images digitally provided to me by the institutions in the Acknowledgments, or old physical films currently in my possession. Cichlid species are listed by geographic area as described in *Suppl. material 1*.

Abbreviations: 2D, two-dimensional; 3D, three-dimensional; AMNH, American Museum of Natural History, New York; ANSP; The Academy of Natural Sciences of Drexel University, Philadelphia; BMNH, The Natural History Museum, London; c+s, cleared and stained; CUMV, Cornell University Museum of Vertebrates, Ithaca; det., determined; DRC, Democratic Republic of the Congo; EAFRO, East African Fisheries Research Organisation; EAFFRO, East African Freshwater Fisheries Research Organisation; FMNH, Field Museum of Natural History, Chicago; IBP, International Biological Programme; LMTS, Lake Malawi Trawling Survey; LSUMZ, Louisiana State Museum of Natural History, Baton Rouge; MCZ, Museum of Comparative Zoology, Harvard University, Cambridge, Massachusetts; MFRU, Malawi Fisheries Research Unit collection, Monkey Bay; PSU, Pennsylvania State University, University Park; RMCA, Royal Museum for Central Africa, Tervuren; RMNH, Naturalis Biodiversity Center, Leiden; TCWC, Biodiversity Research and Teaching Collections, Department of Wildlife and Fisheries Sciences, Texas A&M University, College Station; UAMZ, University of Alberta Museum of Zoology, Edmonton; UF, University of Florida ichthyology collections, Gainesville; UMMZ, University of Michigan Museum of Zoology, Ann Arbor; UNIBAS, University of Basel Zoological Institute, Basel: Lake Tanganyika Cichlid Collection; USNM, U.S. National Museum of Natural History, Washington, D.C.; UWFC, University of Washington, Burke Museum of Natural History and Culture, Seattle; YPM, Peabody Museum of Natural History, Yale University, New Haven; ZSM, Zoologische Staatssammlung München.

PHOLIDICHTHYIDAE

Pholidichthys leucotaenia Bleeker, 1856: YPM 026766, 1 (3D CT image series, MorphoSource, available at http://n2t.net/ark:/87602/m4/426003); captive. — USNM 348992, 2 (2D radiographs available at http://n2t.net/ark:/65665/3ccf92b71-377b-4382-81fc-e8fbe9d411fa and http://n2t.net/ark:/65665/39b299bf3-7c85-4055-99e1-a749edf7f0db); Solomon Islands: Florida Island: Tanavula, in 4 m; T. Alburn, 20-Apr-1998. — USNM 212163, 1 (2D radiograph available at http://n2t.net/ark:/65665/3eb66e10f-6b19-42c3-8404-c373533b4672); Japan: Ryukyu Islands: Okinawa (?); captive. (I examined these images of *Pholidichthys* but, given the extreme elongation and many autapomorphies of this highly derived species, I recorded no data from them for this study.)

POLYCENTRIDAE

Afronandus sheljuzhkoi (Meinken, 1954): USNM 372183, 1 (2D radiograph available at http://n2t.net/ark:/65665/38f5d20c3-be58-4339-a02f-2015b0abf5fb); Ghana; U. Schliewen.

Monocirrhus polyacanthus Heckel, 1840: UF 120003, 1 (3D CT image series, MorphoSource, available at http://n2t.net/ark:/87602/m4/M169968); Venezuela: Amazonas: Cano Galipero, small stream 21 km N of Puerto Ayacucho, just SE of bridge on highway between Puerto Ayacucho & Caicara; L. Nico, H. Jelks, K. Winemiller, et al., 3-Jan-1999. — USNM 103840, 1 (2D radiographs available at http://n2t.net/ark:/65665/3b1ca9d61-e1d6-4a16-9db8-4eca7af5e529); captive.

Polycentropsis abbreviata Boulenger, 1901: CUMV 93208, 1 (3D CT image series, MorphoSource, available at http://n2t.net/ark:/87602/m4/440836); Cameroon: Sud Province: Fifinda village, about 35 km N of Kribi; D. Reid, 6-Jan-2006. — USNM 302514, 1 (2D radiograph available at http://n2t.net/ark:/65665/3321d4263-2b0a-4cb7-ab75-58b5be742c20); Cameroon: Littoral Province: lower Sanaga River: Riviere Mboli about ½ mile upstream from Mouanko (Mouangko); T.R. Roberts, 29-Apr-1971.

CICHLIDAE

Etroplinae

India

Etroplus suratensis (Bloch, 1790): TCWC 11237.06, 1 (3D CT image series, MorphoSource, available at http://n2t.net/ark:/87602/m4/M70704); India: Tamil Nadu: Manavalakurichy; 9-Aug-2001.

Pseudetroplus maculatus (Bloch, 1795): FMNH 17028, 6 (2D radiograph); captive. — MCZ 4311, 1 (2D radiograph); India: Kerala: Malabar Coast: Canara (Kannur); F. Day, 1852–1887.

Madagascar

Paretroplus polyactis Bleeker, 1878: FMNH 6156, 1 (2D radiograph); Madagascar.

Ptychochrominae

Madagascar

Katria katria (Reinthal & Stiassny, 1997): UMMZ 250284, 1 (3D CT image series, MorphoSource http://n2t.net/ark:/87602/m4/M133871); Madagascar.

Oxylapia polli Kiener & Maugé, 1966: UMMZ 250283, 1 (3D CT image series, MorphoSource http://n2t.net/ark:/87602/m4/M126199); Madagascar: Nosivolo River, near village of Marolambo, Mangoro drainage.

Paratilapia polleni, Bleeker 1868: AMNH 11687, 5 (2D radiograph); Madagascar: Lac Iotry; R. Archbold & A.L. Rand, 26-Dec-1929. — AMNH 11689, 3 (2D radiograph); Madagascar; A.L. Rand & P.A. Dumont, 5-Oct-1931. — AMNH 18842, 1 (2D radiograph); no data. — AMNH 11709, 2 (2D radiograph); Madagascar; H.C. Raven, 9-Mar-1931.

Ptychochromis oligacanthus, (Bleeker 1868): YPM 023177, 1 (3D CT image series, MorphoSource http://n2t.net/ark:/87602/m4/M80207); captive. — UMMZ 237497, 11 (10 used; 2D radiograph); Madagascar: Nosy Be: Lake Andjavibe; 13-Jul-1996.

Ptychochromoides betsileanus, (Boulenger 1899): UMMZ 238114, 1 (3D CT image series, MorphoSource http://n2t.net/ark:/87602/m4/M123960); same lot, 1 (another specimen, 2D radiograph); both, Madagascar: Ilanana River, S of Isalo National Park; P.N. Reinthal.

Cichlinae

Neotropics

Astronotini

Astronotus ocellatus, (Agassiz 1831): FMNH 85660, 1 (2D radiograph); Venezuela: Amazonas: Rio Orinoco, 8.6 km S toward Puerto Ayacucho from Puerto Nuevo, small cano under road; J. Thomerson, D. Hicks, D.C. Taphorn, & H. Lopez, 14-Jan1975. — FMNH 105301, 2 (2D radiograph); Venezuela: Barinas: Rio Orinoco, Cano Sioca on W side of Rio Suripa, ca. 40 min by boat from Hato Las Mercedes pumping station; B. Chernoff, A. Machado-Allison, R. Royero, & F. Gil, 11-Jan-1991.

Chaetobranchini

Chaetobranchopsis orbicularis (Steindachner, 1875): UF 188930, 1 (3D CT image series, MorphoSource http://n2t.net/ark:/87602/m4/M159667); Brazil: Para: Santarém, Lago do Santana, Marimarituba community, at island located in main channel of rio Amazonas ca. 50 km upstream of Santarém; O. Bogota-Gregory & S. Oliveira, 10-Oct-2014.

Chaetobranchus flavescens Heckel, 1840: FMNH 101872, 2 (2D radiograph); Ecuador: Napo, Rio Yasuni, Quebrada to Rio Jantuncocha, ca. 1 km upstream from Laguna Jatuncocha; D.J. Stewart, M.C. Ibarra, R. Barriga, & C. Uquillas, 24-Oct-1981. — FMNH 111427, 2 (2D radiograph); Peru: Loreto: Cano Abico tributary to Rio Samiria, ca. 6–7 km from mouth in Rio Marañon; B. Chernoff, J.A. Wheeler, R.A. Klocek, B. DuVall, Onate, & H. Blicksilver, 1-Sep-1988.

Cichlasomatini

Aequidens tetramerus (Heckel, 1840): CAS SU 17302, 1 (3D CT image series, MorphoSource http://n2t.net/ark:/87602/m4/M100023); Peru: Loreto: Rio Ampiyacu near Pebas; W.G. Scherer, 28-Nov-1940.

Andinoacara pulcher (Gill, 1858): ANSP 195366, 1 (3D CT image series, MorphoSource http://n2t.net/ark:/87602/m4/M97817); Venezuela: Portuguesa: Guanare, caño volcan (tucupido dr.), dirt road crossing 14.5 km southwest of Guanare, ca. 1.25 km west of route 5; N.K. Lujan, D.C. Werneke, S.V. Meza V., & O. León Mata, 15-Mar-2010.

Bujurquina vittata (Heckel, 1840): ANSP 124129, 1 (3D CT image series, MorphoSource http://n2t.net/ark:/87602/m4/M97520); Paraguay: Alto Paraguay: Rio Tapiricuay; E.J. Phillips, 6-Jan-1960.

Cichlasoma dimerus (Heckel, 1840): ANSP 124090, 1 (3D CT image series, MorphoSource http://n2t.net/ark:/87602/m4/M97789) ; Paraguay: Alto Paraguay: Primavera; E.J. Phillips, 10-Sep-1959.

Cichlasoma taenia (Bennett, 1831): ANSP 76395, 1 (3D CT image series, MorphoSource http://n2t.net/ark:/87602/m4/M97559); Trinidad: Tumpuna River; L. Wehekind, 14-Jun-1930.

Krobia guianensis (Regan, 1905): ANSP 179496, 1 (3D CT image series, MorphoSource http://n2t.net/ark:/87602/m4/M97601); Guyana: Cuyuni-Mazaruni: Whitewater Creek, small blackwater creek tributary to Mazaruni R. (Essequibo drainage), 6.8 km SW Bartica; M.H. Sabaj, J.W. Armbruster, M.R. Thomas, D.C. Werneke, C.L. Allison, C.J. Chin, & D. Arjoon, 12-Nov-2002.

Cichlini

Cichla ocellaris Bloch & Schneider, 1801: UF 244243, 1 (3D CT image series, MorphoSource http://n2t.net/ark:/87602/m4/438443); USA: Florida: Tamiami Canal (C-4) just east of SW 127th Avenue bridge; L. Nico, D. Ruessler, J. Herod, & G. Hill, 24-Mar-1999.

Cichla orinocensis Humboldt, 1821: FMNH 103505, 8 (3 used; 2D radiograph); Venezuela: Amazonas: Rio Orinoco, rocks in Rio Atabapo at shore and inlet of Isla de Sapo, ca. 1.2 hr. above San Fernando de Atabapo; B. Chernoff, A. Machado-Allison, & J.A. Wheeler, 28-Jan-1991.

Geophagini

Biotodoma cupido (Heckel, 1840): UF 100668, 1 (3D CT image series, MorphoSource http://n2t.net/ark:/87602/m4/M159661); Brazil: Rondonia: Jaciparana River, ca 3 km upstream from Town Jaciparana; J. Viana, 3-Sep-1994. — FMNH 53843, 3 (2D radiograph); Guyana: Lama stop-off; C.H. Eigenmann, 1908.

Geophagus crassilabris Steindachner, 1876: UF 240894, 1 (3D CT image series, MorphoSource http://n2t.net/ark:/87602/m4/M81876); Panama: Rio Frijoles on Pipeline Road near Gamboa (Canal Zone); H. Loftin, 1963.

Guianacara owroewefi Kullander & Nijssen, 1989: FMNH 117023, 5 (4 used; 2D radiograph); Suriname: Coppename River, Rechter Coppename at rocks & sand beach; J.H. Mol, B. Chernoff, P.W. Willink, & M. Cooperman, 23-Feb-2004. — FMNH 117029, 4 (2D radiograph); Coppename River, unnamed creek, Linker Coppename, just downstream from unnamed creek 1; J.H. Mol, B. Chernoff, P.W. Willink, & M. Cooperman, 29-Feb-2004.

Saxatilia frenata (Gill, 1858): USNM 1111, 1 (holotype; 2D radiograph available at http://n2t.net/ark:/65665/m37eb364b0-ac01-4128-981b-834d1f46a48c); West Indies: Trinidad Island; T.N. Gill.

Saxatilia lepidota (Heckel, 1840): BMNH 1935.6.4.416–419, 5 (2 used; paratypes of *Crenicichla edithae* = *S. lepidota*; 2D radiograph available at https://data.nhm.ac.uk/object/8fd61af3-e70c-4c25-90ce-42262cd2aac6); Paraguay: Asunción: West Asunción; G. Schouten.

Heroini

Amphilophus citrinellus (Günther, 1864): USNM 44180, 1 (2D radiograph available at http://n2t.net/ark:/65665/39f96f501-1fd0-4fdf-87c9-c1304d59c487); Nicaragua: L. Nicaragua; C.W. Richmond, 20-Jul-1892.

Hypselecara coryphaenoides (Heckel, 1840): USNM 206097, 1 (holotype of *Chuco axelrodi* = *H. coryphaenoides*; 2D radiograph); Venezuela: Aguaro River; H.R. Axelrod & A. Fernandez–Yepez, 6-May-1971. — USNM 206098, 4 (paratypes of *Chuco axelrodi*; 2D radiograph); other data same as USNM 206097.

Retroculini

Retroculus lapidifer (Castelnau, 1855): USNM 152111, 1 (holotype of *R. boulengeri* = *R. lapidifer*; 2D radiograph available at http://n2t.net/ark:/65665/3bf2ad876-904f-4eb4-866d-2d488a9b740a); "Brazil"; C.F. Hartt.

Pseudocrenilabrinae

Middle East

Oreochromini

Iranocichla hormuzensis Coad, 1982: BMNH 1981.1.12.1–2, 2 (paratypes; 2D radiograph available at https://data.nhm.ac.uk/object/6a0b1403-d7e7-4a45-a7a9-e653cd368a95); Iran: Hormozgan: Mehran River; B.W. Coad, 18-Mar-1978.

Tristramella sacra (Günther, 1865): UMMZ 213421, 1 (3D CT image series, MorphoSource http://n2t.net/ark:/87602/m4/M123954); Israel: Lake Kinneret (=Lake Tiberias); 3-Nov-1962.

Tristramella simonis (Günther, 1864): AMNH 225653, 4 (2D radiographs available at https://emu-prod.amnh.org/db/emuwebamnh/objects/media.php?irn=5081875, https://emu-prod.amnh.org/db/emuwebamnh/objects/media.php?irn=5081876, https://emu-prod.amnh.org/db/emuwebamnh/objects/media.php?irn=5081877, & https://emu-prod.amnh.org/db/emuwebamnh/objects/media.php?irn=5081878); Israel: Lake Kinneret (=Lake Tiberias); A. Ben-Tuvia, Jan-1991.

Pseudocrenilabrini

Astatotilapia flaviijosephi (Lortet, 1883): BMNH 1898.12.5.5–6, 2 (paralectotypes; 2D radiograph available at https://data.nhm.ac.uk/object/b69774c6-0f87-4353-9950-94ac2cc609c8); Syria: Ain-el-Tabigah; L. Lortet. — BMNH 1949.9.16.398, 2 (2D radiograph available at

https://data.nhm.ac.uk/object/39938fcb-993a-4275-8486-a100db3be26d); Israel: Lake Tiberias; C. Ricardo-Bertram.

African Riverine

Chromidotilapiini

Benitochromis batesii (Boulenger, 1901): BMNH 1904.2.29.57–59, 6 (2D radiograph available at https://data.nhm.ac.uk/object/a2726bfc-9afb-4990-908d-08d4efdabf7a); Cameroon: Kribi River; G. Bates.

Benitochromis finleyi (Trewavas, 1974): USNM 205627, 1 (3D CT image series, MorphoSource http://n2t.net/ark:/87602/m4/M101315); W Cameroun, small "black-water" tributary of Mungo River ½ mile E of main channel of Mungo on the Loum road; D. Blair, Jul-1970.

Chromidotilapia guntheri (Sauvage, 1882): BMNH 1959.8.18.178, 1 (holotype of *Pelmatochromis loennbergi* = *C. guntheri*; 2D radiograph available at https://data.nhm.ac.uk/object/3169e670-82ed-451d-9f62-c5e0d22e8efa); Cameroon: Lake Barombi-ba-Kotto; P.I. Maclaren, 1-Sep-1948. — BMNH 1961.10.18.6, 1 (paratype of *P. loennbergi* = *C. guntheri*; 2D radiograph available at https://data.nhm.ac.uk/object/a5596593-b263-492e-ad52-7f4251eede74); Cameroon: Lake Barombi-ba-Kotto; M. Eisentraut, 1-Jan-1958.

Chromidotilapia kingsleyae Boulenger, 1898: CUMV 96466, 1 (3D CT image series, MorphoSource, http://n2t.net/ark:/87602/m4/M62174); Gabon: Ngounie: Loétsi: Loétsi River at rocks below Bongolo Falls; M. Arnegard, B. Carlson, & J. Gallant, 4-Aug-2009. — BMNH 1867.5.3.1, 1 (paralectotype; 2D radiograph available at https://data.nhm.ac.uk/object/01fb5724-fc61-4fb4-9a0e-14c95caa56e4); Gabon; Higgins. — BMNH 1912.4.1.517–525, 8 (2D radiograph available at https://data.nhm.ac.uk/object/c1487f59-921b-4b88-8ad8-6fb8e78d6a0a); Angola: Luali River at Lundo; W.J. Ansorge.

Congochromis robustus Lamboj, 2012: RMCA 135706, 1 (holotype; 2D radiograph in Lamboj 2012: fig. 2); DRC: Yaekama; J.P. Gosse, 1954.

Congochromis squamiceps (Boulenger, 1902): CUMV 96716, 1 (3D CT image series, MorphoSource http://n2t.net/ark:/87602/m4/440878); DRC: mouth of Lubilu River 14.5 km upriver from Yangambi; S. Lavoué, J. Thumito, & J. Sullivan, 10-Sep-2010. — BMNH 1902.4.14.11, 1 (syntype; 2D radiograph available at https://data.nhm.ac.uk/object/6965e80a-abd4-442a-a2d3-ee935a88dfea); DRC: Lindi River.

Divandu albimarginatus Lamboj & Snoeks, 2000: AMNH 232347, 4 (2D radiograph); Gabon: Lambarene; C.D. Hopkins, M. Arnegard, T. Uschold, & S. Lavoue, 19-Jul-2001.

Limbochromis robertsi (Thys van den Audenaerde & Loiselle, 1971): USNM 214090, 17 (2D radiograph); Ghana: Eastern Region: East Akim Municipal District: Birim River, Kibi, small tributary of Birim Creek 1 mile SE of Kibi Men's Training College; P.V. Loiselle, 4-Apr-1970.

Nanochromis nudiceps (Boulenger, 1899): CUMV 88260, 1 (3D CT image series, MorphoSource http://n2t.net/ark:/87602/m4/M61974); Republic of the Congo: Cuvette-Ouest: small creek flowing into Lékoli River, Odzala National Park; J. Friel, S. Lavoué, & J. Sullivan, 11-Aug-2002. — BMNH 1963.10.22.9, 1 (2D radiograph available at https://data.nhm.ac.uk/object/c34757f1-1f47-42e3-b32e-229b6327611d); Nigeria: 50 km W of Lagos (?); W. Wickler.

Nanochromis parilus Roberts & Stewart, 1976: BMNH 1977.1.11.33–34, 2 (2D radiograph available at https://data.nhm.ac.uk/object/4a3b28ce-3213-46fd-b020-bedcb899c2ce); Republic of Congo: Pool Province: Ngamaba, Stanley Pool (Pool Malebo), Regina Falls; U. Werner.

Parananochromis gabonicus (Trewavas, 1975): BMNH 1967.10.12.57, 1 (holotype; 2D radiograph available at https://data.nhm.ac.uk/object/00346032-cea3-4481-9c33-67977b7d0bd0); Gabon: pool between Mitzic and Medouneu, 15 km from Mitzic Roadside, probably in the drainage basin of the Okano River, tributary of Ogowe River; Cambridge French West Africa Expedition, 1957.

Parananochromis longirostris (Boulenger, 1903): CUMV 80766, 1 (3D CT image series, MorphoSource http://n2t.net/ark:/87602/m4/M62148); Gabon: Woleu-Ntem Province: Deghe Creek near Auberge d'Ayengbe; J. Friel, S. Lavoué, & J. Sullivan, 4-Sep-1999. — AMNH 230709, 10 (2D radiograph); Gabon: Ogooue–Ivindo: small creek mouth emptying into Ivindo River, across from IRET field station; C.D. Hopkins, J.P. Sullivan, & E. Vreven, 25-Jan-1998.

Pelmatochromis buettikoferi (Steindachner, 1894): CUMV 97836, 1 (3D CT image series, MorphoSource http://n2t.net/ark:/87602/m4/M69920); Guinea: Forecariah: Serguey Creek, near Bassia; Forécariah River - Bofon River; H. Bart, J. Armbruster, J. Friel, & party; 5-Jan-2013. — BMNH 1910.11.28.11–12, 2 (syntypes of *Paratilapia corbali* = *Pelmatochromis buettikoferi*; 2D radiograph available at https://data.nhm.ac.uk/object/dd6b6a1b-942c-402b-8f76-c650878a9f7f); Guinea–Bissau: Corbal River at Chitole; W.J. Ansorge.

Pelmatochromis ocellifer Boulenger, 1899: BMNH 1898.7.9.16, 1 (holotype; 2D radiograph available at https://data.nhm.ac.uk/object/0c27c0e2-07e0-441c-8ee6-dad13ec7c34d); DRC: Upper Congo: Monsembe; J. Weeks.

Pelvicachromis pulcher (Boulenger, 1901): YPM 009680, 1 (3D CT image series, MorphoSource http://n2t.net/ark:/87602/m4/M123976); captive.

Pelvicachromis taeniatus (Boulenger, 1901): BMNH 1901.1.28.21, 1 (holotype; 2D radiograph available at https://data.nhm.ac.uk/object/71847c76-aecf-4b4b-9d5b-25026bab5d8c); Nigeria: mouth of Ethiop River, Niger Delta (Sapele Station); W. Ansorge.

Pterochromis congicus (Boulenger, 1897): BMNH 1897.9.30.12, 1 (holotype; 2D radiograph available at https://data.nhm.ac.uk/object/10a2e3c4-29d0-44a0-bb1b-2bfbfbffe5c0); DRC: Orientale: Kisangani: Stanley Falls; W. Bentley. — BMNH 1901.12.21.77, 1 (2D radiograph available at https://data.nhm.ac.uk/object/4d4876e6-ab31-44b0-be42-6f21e117cb8b); DRC: Monsembe; J. Weeks.

Teleogramma brichardi Poll, 1959: USNM 331312, 4 (2D radiograph); Republic of the Congo: Kinshasa Province: rapids of Congo River at Kinsuka (near Kinshasa); T.R. Roberts, 20–23-Jun-1971.

Thysochromis ansorgii (Boulenger, 1901): YPM 014283, 1 (3D CT image series, MorphoSource http://n2t.net/ark:/87602/m4/M123651); captive. — BMNH 1912.2.2.10–12, 3 (syntypes of *Pelmatochromis arnoldi* = *T. ansorgii*; 2D radiograph available at https://data.nhm.ac.uk/object/1ba0e9eb-3e07-4cca-946d-27524edee7b2); Nigeria: lower Niger River; J.P. Arnold.

Wallaceochromis humilis (Boulenger, 1916): BMNH 1915.4.13.44, 1 (holotype; 2D radiograph available at https://data.nhm.ac.uk/object/990620ed-76a9-4c2f-bbaa-42ce93fa548e); Sierra Leone: North Sherbo District (rivers not far from the sea); N.W. Thomas. — AMNH 97496, 4 (2D radiograph); Sierra Leone: Southern Province: Tiwai, River Moa; M.J. Stiassny & P.N. Reinthal, 14–19-Feb-1990.

Coelotilapiini

Coelotilapia joka (Thys van den Audenaerde, 1969): BMNH 1970.1.5.1, 1 (2D radiograph available at https://data.nhm.ac.uk/object/48daf24d-85ac-4bda-a645-75b27a874cc1); Sierra Leone: forest stream near Pujehun; E. Roloff.

Coptodonini

Coptodon discolor (Günther,,1903): BMNH 1903.4.24.33–35, 3 (2 used; 2D radiograph available at https://data.nhm.ac.uk/object/e9ac3170-e141-4600-9dfb-5ada1f99c7e0); Ghana: Lake Busum-chi [Bosumtwi]; R.B.N. Walker.

Coptodon tholloni (Sauvage, 1884): BMNH 1896.5.5.33–35, 1 (syntype of *Chromis ogowensis* = *C. tholloni*; 2D radiograph available at https://data.nhm.ac.uk/object/1641c818-ac7e-4d7e-a18e-03ce25e5857e); Gabon: Ogowe River at Lambarene; M.H. Kingsley.

Coptodon zillii (Gervais, 1848): BMNH 1932.12.16.776–798, 10 (as *Tilapia melanopleura* = *C. zillii*; 2D radiograph available at https://data.nhm.ac.uk/object/55551633-2c69-4ce3-83f0-46d1a8a83096); Zambia: Luapula system: Chinsali; C.R. Pitman. — USNM 229645, 14 (2D radiograph); Nigeria: causeway bridge over Rima River, University Road; G.M. Reid, 12-Feb-1981.

Etiini

Etia nguti Schliewen & Stiassny, 2003: UMMZ 248260, 1 (3D CT image series, MorphoSource http://n2t.net/ark:/87602/m4/M126183); wild-caught captive. — ZSM-PIS-029430_20140129_121137, 1 (2D radiograph). — ZSM-PIS-029430_20140129_121735, 1 (2D radiograph). — UAMZ F8702, 3 (c+s); captive.

Gobiocichlini

Gobiocichla ethelwynnae Roberts, 1982: USNM 229454, 15 (paratypes; 11 used; 2D radiograph); Cameroon: Southwest Province: rapids in mainstream of Cross River, 4 km downstream from Mamfe; T.R. Roberts, 1–5-Mar-1980.

Gobiocichla wonderi Kanazawa, 1951: USNM 357037, 14 (2D radiograph); Nigeria: Adamawa State: Gashaka Gumpti National Park, Mayo Yim River, Bodel Village, Toungo Lga, altitude 245 m; J.T. Bell, 12-Apr-1998.

Paragobiocichla irvinei (Trewavas, 1943): BMNH 1943.7.24.1–4, 4 (syntypes; 2D radiograph available at https://data.nhm.ac.uk/object/20e6335e-0493-496a-b192-30f914f7f28b); Ghana: River Volta, rapids at Senchi; E.B. Worthington.

Hemichromini

Anomalochromis thomasi (Boulenger, 1915): CUMV 97804, 1 (3D CT image series, MorphoSource http://n2t.net/ark:/87602/m4/M59986); Guinea: Kindia: "Safa-Khoure River, at Camara-Bouyhe; Badi River - Konkouré River"; H. Bart & party, 14-Jan-2013. — BMNH 1981.6.19.104, 1 (2D radiograph available at https://data.nhm.ac.uk/object/dd7d1481-8bf5-4097-9e2f-2659b1a6bfed); Sierra Leone: Taiama, River Taia; A. Payne. — BMNH 1981.6.19.118, 1 (2D radiograph available at https://data.nhm.ac.uk/object/d1b8d6b0-457b-40f8-9052-4a99a99b2799); Sierra Leone; Southern; Moyamba; A. Payne. — BMNH 1981.6.19.124, 1 (2D radiograph available at https://data.nhm.ac.uk/object/10973db2-d1bf-40a6-bcc1-c85e64aff12b); Sierra Leone: Little Scarcies; Little Scarcies River; A. Payne.

Hemichromis elongatus (Guichenot, 1861): CUMV 92323, 4 (2D radiograph); Gabon: Ogooue–Ivindo: Ivindo River: rapids at Loa-Loa; M. Arnegard, P. McIntyre, S. Lavoué, & V. Mamonekene, 3-Jul-2006.

Hemichromis fasciatus Peters, 1857: CUMV 88248, 1 (3D CT image series, MorphoSource http://n2t.net/ark:/87602/m4/M63107); Republic of the Congo: Cuvette-Ouest: Lékoli River, Odzala National Park; J. Friel, S. Lavoué, & J. Sullivan, 24-Aug-2002.

Rubricatochromis bimaculatus (Gill, 1862): CUMV 88263, 10 (6 used; 2D radiograph); Republic of the Congo: Cuvette-Ouest: Lékoli River, Odzala National Park; J. Friel, S. Lavoué, & J. Sullivan, 8-Aug-2002.

Rubricatochromis letourneuxi Sauvage, 1880: BMNH 1898.2.15.1, 1 (syntype; 2D radiograph available at https://data.nhm.ac.uk/object/aea7e5d7-57e3-47eb-8d3c-0fa51c3b591b); Egypt: Lake Mareotis, Letourneux. — CUMV 94558, 25 (3 used; 2D radiograph); Ethiopia: Gambella: Obela Stream on road from Gambella to Gog; J. Friel, S. Lavoué; J. Sullivan, & T. Melak, 6-Oct-2008.

Heterochromini

Heterochromis multidens (Pellegrin, 1900): CUMV 88257, 8 (5 used; 2D radiograph); Republic of the Congo: Cuvette-Ouest: Pandaka River, Odzala National Park; J. Friel, S. Lavoué, & J. Sullivan, 12-Aug-2002. — CUMV 88258, 1 (3D CT image series, MorphoSource http://n2t.net/ark:/87602/m4/M63118); Republic of the Congo: Cuvette-Ouest: small channel around island in Lékoli River, Odzala National Park;

J. Friel, S. Lavoué, & J. Sullivan, 16-Aug-2002. — CUMV 89064, 3 (2D radiograph); same data as CUMV 88258. — AMNH 239640, 2 (2D radiograph available by searching catalog number 239640 at https://emu-prod.amnh.org/db/emuwebamnh/Query.php?dept=Ichthyology); Republic of the Congo: Brazzaville: Cuvette Ouest, Odzala National Forest at fishermen's camp, 00° 36' 57.6" N, 014° 54' 50.7" E; V. Mamonekene, M.E. Arnegard, P.B. McIntyre, S. Lavoué, Bossi, & E. Kinzonzi, 26-Jun-2006.

Heterotilapiini

Heterotilapia buettikoferi (Hubrecht, 1881): AMNH 59425, 5 (2D radiograph); Sierra Leone: Southern Province: Tiwai, River Moa; M.J. Stiassny & P.N. Reinthal, 16-Feb-1990.

Oreochromini

Danakilia franchettii (Vinciguerra, 1931): AMNH 223754, 12 (2D radiograph); Ethiopia: Afar: Lake Afrera, 280 km NW of Asaita; A. Getahun, 25-Nov-1996. — BMNH 1931.4.15.1–4, 5 (2 used; syntypes; 2D radiograph available at https://data.nhm.ac.uk/object/d9069444-8ef7-493a-911c-5863d47a8bf8); Ethiopia: Afar: Lake Afrera, Dancalia; D Vinciguerra.

Oreochromis jipe (Lowe, 1955): BMNH 1952.2.26.7–9, 7 (4 used; as *Tilapia girigan* = *O. jipe*; 2D radiograph available at https://data.nhm.ac.uk/object/b81b1e3c-aa6d-497a-bb9e-d3e560b184a9); Kenya: L. Jipe; R. Lowe, 29-Dec-1950.

Oreochromis niloticus (Linnaeus, 1758): BMNH 1920.5.26.121–123, 3 (2D radiograph available at https://data.nhm.ac.uk/object/e54d1108-4618-427a-b90f-6b7f2dd914e2); Zambia: Luapula River at Kasenga; L. Stappers.

Oreochromis shiranus Boulenger, 1897: AMNH 31872, 6 (2D radiograph); Malawi: L. Malawi: "possibly Nkhata Bay," D.H. Eccles, 24-Apr-1969.

Sarotherodon galilaeus (Linnaeus, 1758): BMNH 1972.1.27.4–5, 2 (as *S. sanagaensis* = *S. galilaeus;* 2D radiograph available at https://data.nhm.ac.uk/object/1844f126-6303-4e5e-b4d8-28d07b83e168); Cameroon: Lom River, affluent of Sanaga River; Bard.

Sarotherodon melanotheron Rüppell, 1852: UF 91814, 1 (3D CT image series, MorphoSource http://n2t.net/ark:/87602/m4/439827); USA: Florida: Patrick Air Force Base canal, Satellite Beach. — BMNH 1972.2.18.17–22, 5 (2D radiograph available at https://data.nhm.ac.uk/object/0cb630c3-f146-47ee-afd9-de3ccdf6ab16); Ghana: Mouri Lagoon 4 miles from Cape Coast; K. Eyeson.

Pelmatolapiini

Pelmatolapia mariae (Boulenger, 1899): USNM 304008, 7 (6 used; 2D radiograph); Cameroon: Southwest Province: Manyu district: Cross System, collecting points on main Cross River about 23 km downstream from Mamfe; G.M. Reid, 17-Feb-1988.

Pseudocrenilabrini

Astatotilapia bloyeti (Sauvage, 1883): BMNH 1898.2.15.2, 1 (syntype; 2D radiograph available at https://data.nhm.ac.uk/object/39a4151e-746e-444f-b6cf-f139e2c331a8); Tanzania: Kandoa. — BMNH 1969.2.11.372–387, 8 (7 used; 2D radiograph available at https://data.nhm.ac.uk/object/a8d5f8c0-6b82-4553-8365-d2d89508dcfe); Tanzania: lake on E. African Sisal Plantations in Myombo system, tributary of River Mukondokwa, Wami system; R. Bailey & E. Trewavas.

Astatotilapia burtoni (Günther, 1894): BMNH 1950.4.1.2176–2200, 7 (6 used; 2D radiograph available at https://data.nhm.ac.uk/object/622a5c20-5555-43a4-aa7b-b670ad5afe7b); Burundi / Tanzania / Zambia / Zaire: L. Tanganyika; C. Christy.

Astatotilapia calliptera (Günther, 1894): BMNH 1893.11.15.1–4, 2 (of which 1, the lectotype, is used; 2D radiograph available at https://data.nhm.ac.uk/object/dafb15e0-06de-44ef-aed5-dfadab88df26); Malawi: "Lake Nyasa [=Malawi] and the Upper Shire River"; H.H. Johnston. — BMNH 1893.1.17.4, 1 (a paralectotype; 2D radiograph available at https://data.nhm.ac.uk/object/56e65052-7ba6-4524-9fa1-5187f7478e98); Malawi: L. Malawi; J. Williams. — USNM 330613, 6 (2D radiograph); Malawi: Chikwawa, pond at fish farm; D.H. Eccles. — BMNH 1921.9.6.84–93, 6 (1 used; 2D radiograph available at https://data.nhm.ac.uk/object/c6eae31d-3ff8-4cec-bd0e-9158c7074a14); Malawi: Cholo: North Wadzi River, elevation 823 m; R. Wood.

Astatotilapia swynnertoni (Boulenger, 1907): BMNH 1907.7.2.21–23, 3 (syntypes, note that radiograph is correctly labeled at its upper edge but mislabeled at its lower edge; 2D radiograph available at https://data.nhm.ac.uk/object/ecb5770b-7260-49bb-9c63-a024246da320); Mozambique: Idundu River, Lower Buzi; C.F.M. Swynnerton.

Chetia flaviventris Trewavas, 1961: UMMZ 251518, 1 (2D radiograph); Botswana.

Chetia gracilis (Greenwood, 1984): BMNH 1984.2.6.147, 1 (holotype; 2D radiograph available at https://data.nhm.ac.uk/object/17d3b948-4079-4ea2-9687-33fbaa7f6584); Angola: Cutato River at Jamba bridge, Cubango; M.J. Penrith.

Chetia? sp.: AMNH 8334, 8 [+1 in another genus, excluded] (2D radiograph); equatorial Africa; A. Baudon, date not reported.

Ctenochromis pectoralis Pfeffer, 1893: BMNH 1899.2.27.1, 1 (paralectotype; 2D radiograph in *Genner et al. 2022*: fig. 8a); Tanzania: Korogwe. — BMNH 2021.7.15.1–3 part, 1 (2D radiograph in *Genner et al. 2022*: fig. 8b); Tanzania: Ruvu River.

Ctenochromis scatebra Genner, Ngatunga, & Turner, 2022: BMNH 2021.7.15.4, 1 (holotype; 2D radiograph in *Genner et al. 2022*: fig. 8c); Tanzania: Chemka Springs; 17-Aug-2015.

Haplochromis demeusii (Boulenger, 1899): BMNH 1899.6.28.25, 1 (holotype; 2D radiograph available at https://data.nhm.ac.uk/object/0548b02b-ee76-4e3b-9c8a-b41eb859e807); Republic of Congo: Upper Congo: Bangala county; M. Meuse.

Haplochromis fasciatus (Perugia, 1892): BMNH 1898.12.12.1–6, 6 (syntypes; 2D radiograph available at https://data.nhm.ac.uk/object/b1b6d4ca-e8db-48e9-bd01-03f823946fea); DRC: lower Congo: Vivi.

Haplochromis humilis (Steindachner, 1866): BMNH 1937.4.22.110–111, 2 (2D radiograph available at https://data.nhm.ac.uk/object/355349cc-ca66-48fc-9eab-b45c7c1c4283); Zambia: upper Zambezi River at Balovale; T. Jones. — BMNH 1937.4.22.109, 1 (2D radiograph available at https://data.nhm.ac.uk/object/381aad17-64f5-4231-9737-69574cafa486); Zambia: Mombezi River; T. Jones.

Haplochromis moeruensis (Boulenger, 1899): BMNH 1920.5.26.148–153, 4 (2D radiograph available at https://data.nhm.ac.uk/object/d6a9e007-45df-4a3d-840f-04b3b4f8116e); DRC: S. Katanga: L. Mweru at Lukonzolwa; L. Stappers.

Haplochromis oligacanthus Regan, 1922: BMNH 1920.7.12.48, 1 (holotype; 2D radiograph available at https://data.nhm.ac.uk/object/2f6fac82-b9c7-4134-bac2-ccfeac8ed41a); Central African Republic: Bangui; W.H. Allen.

Lufubuchromis relictus Schedel, Kupriyanov, Katongo, & Schliewen, 2020: ZSM 47494, 1 (holotype; 2D radiograph in *Schedel et al. 2020*: fig. 6C); Zambia: Northern Province: Mululwe rapids at Mululwe village, 37 km SW of Mpulungu.

Orthochromis machadoi (Poll, 1967): BMNH 1972.9.27.90–91, 2 (2D radiograph available at https://data.nhm.ac.uk/object/d5dd624e-5f1e-4ac6-98af-72ec5fe343da); Angola: Cunene River: Ondurusu Falls; M.J. Penrith.

Orthochromis malagaraziensis (David, 1937): CUMV 95549, 1 (3D CT image series, MorphoSource http://n2t.net/ark:/87602/m4/M61839); Tanzania: Kigoma: Malagarasi River above drill site; J. Friel, P. McIntyre, & R. Shelly, 19-Aug-2009. — BMNH 1937.12.16.1, 1 (syntype; 2D radiograph available at https://data.nhm.ac.uk/object/a4722bc9-0675-4d0e-b8cf-fdd5641735b6); Tanzania: Malagarasi River; L. David.

Orthochromis polyacanthus (Boulenger, 1899): BMNH 1920.5.26.137, 2 (2D radiograph available at https://data.nhm.ac.uk/object/fd7d637e-7809-466a-aaca-d75569ed3319); DRC: South Katan-ga: L. Mweru: Cape Kasengeneke; L. Stappers.

Orthochromis stormsi (Boulenger, 1902): BMNH 1977.1.11.5–24, 8 (2D radiograph available at https://data.nhm.ac.uk/object/ff9093d6-fece-435e-9fb5-71fb9768053d); DRC: Leopoldville, Regina falls at Kensuka; U. Werner. — BMNH 1975.6.20.662-663, 2 (2D radiograph available at https://data.nhm.ac.uk/object/9f5e5ffa-26b8-47c4-9178-12d61d0fc469); DRC: Lualaba River between 2 islands at Elila confluence; K. Banister, Zaire River Expedition 1974–1975. — BMNH 1992.10.9.1–2, 1 (2D radiograph available at https://data.nhm.ac.uk/object/de4d8fab-cf6a-48bf-aff4-5e2e2d6428fd); DRC: South Katanga: L. Mweru: Cape Kasengeneke; L. Stappers.

Palaeoplex palimpsest Schedel, Kupriyanov, Katongo, & Schliewen, 2020: ZSM 47492, 1 (holotype; 2D radiograph in *Schedel et al. 2020*: fig. 3C); Zambia: Luapula Province: Luongo River bridge on Kawambwa–Mansa road ~40 km [driving distance] S of Kawambwa.

Pharyngochromis acuticeps (Steindachner, 1866): BMNH 1908.12.11.5, 1 (holotype of *Pelmatochromis darlingi* = *P. acuticeps*; 2D radiograph available at https://data.nhm.ac.uk/object/794a815d-9da6-4e78-aeb0-a322243ec3e8); Zimbabwe: Makabusi River, tributary of Hunyani River, near Salisbury; J.F. Darling. — BMNH 1937.4.22.99–108, 5 (as *Pharyngochromis darlingi*; 2D radiograph available at https://data.nhm.ac.uk/object/8cb22534-8288-4d04-ac19-52ee76c20cdd); Zambia: upper Zambezi River at Balovale, altitude 1036 m.

Pseudocrenilabrus multicolor (Schöller, 1903): BMNH 1964.7.1.89–108, 11 (4 used; 2D radiograph available at https://data.nhm.ac.uk/object/f0f65457-cef7-4f33-9f74-fd8e05a38b64); Uganda: L. Nabugabo; Cambridge Nabugabo Biological Survey.

Pseudocrenilabrus philander (Weber, 1897): CUMV 91164, 1 (3D CT image series, MorphoSource http://n2t.net/ark:/87602/m4/442895); Zambia: Central Province: isolated pools SE of Luapula River bridge; R. Bills, A. Chilala, & J. Friel, 22-Sep-2005.

Sargochromis carlottae (Boulenger, 1905): BMNH 1910.5.31.65–66, 2 (syntypes of *Paratilapia gibbiceps* = *S. carlottae*; 2D radiograph available at https://data.nhm.ac.uk/object/cd402add-7b31-4513-a628-ec95baf58c35); Botswana: Okavango; R.B. Woosnam.

Sargochromis codringtonii (Boulenger, 1908): BMNH 1908.11.6.32, 2 (lectotype & paralectotype; 2D radiographs available at https://data.nhm.ac.uk/object/1adb6a15-d31d-4e42-b1af-297ff91f14b7 and https://data.nhm.ac.uk/object/4b511571-d037-496b-aec3-157ed04df8cc); Zimbabwe: Zambezi River above Victoria Falls; T. Codrington. — USNM 309604, 1 (2D radiograph); Zambia: Kafue River at junction with Lufupa River, Kafue National Park; K.O. Winemiller & L.C. Kelso-Winemiller, 18-Jun-1989.

Sargochromis giardi (Pellegrin, 1903): BMNH 1907.6.29.181–182, 2 (2D radiograph available at https://data.nhm.ac.uk/object/9f88542b-6c9f-4f02-8ea5-e336d1c88d49); Angola: Mossamedes: Ponang Kuma; W.J. Ansorge.

Sargochromis greenwoodi (Bell-Cross, 1975): BMNH 1910.5.31.53, 1 (holotype; 2D radiograph available at https://data.nhm.ac.uk/object/7ba7b0dd-8bd2-459f-ab4e-4fdcb109adc2); Botswana: Okavango River, Lake Ngami district; R.B. Woosnam. — BMNH 1910.5.31.55, 1 (paratype; 2D radiograph available at https://data.nhm.ac.uk/object/66c74cee-276e-48ae-bf97-142e6d10f57b); other data same as 1910.5.31.53).

Serranochromis angusticeps (Boulenger, 1907): BMNH 1905.11.10.27–30, 1 (2D radiograph available at https://data.nhm.ac.uk/object/fd897e43-7d09-48f6-97e1-953c7a47cf25); Zambia: Lakle Bangweulu; F. Melland & Foulon.

Serranochromis longimanus (Boulenger, 1911): BMNH 1910.5.31.62–64, 4 (lectotype & paralectotypes; 2D radiograph available at https://data.nhm.ac.uk/object/a8139bcb-5b0b-4212-a7b6-aa0de53d039f); Botswana: River Okovango; R.B. Woosnam.

Serranochromis macrocephalus (Boulenger, 1899): BMNH 1920.5.26.164, 1 (2D radiograph available at https://data.nhm.ac.uk/object/91ffe92a-6392-4d4b-9f0b-fbd9579886b3); Zambia: Luapula River: Katanga, South Katanga; L. Stappers.

Serranochromis meridianus Jubb, 1967: BMNH 1967.4.14.1, 1 (paratype; 2D radiograph available at https://data.nhm.ac.uk/object/c72a0867-6a00-4649-aa2f-798aaa117972); South Africa: Eastern Transvaal: Sand River; R. Jubb.

Serranochromis robustus (Günther, 1864): AMNH 31877, 1 (2D radiograph); Malawi: L. Malawi: Monkey Bay, Thumbi Island East; D.H. Eccles, 28-Jan-1969. — AMNH 31879, 1 (2D radiograph); Malawi: L. Malawi; D.H. Eccles, 24-Apr-1969. — BMNH 1935.6.14.2330-2337, 12 (4 used; 2D radiograph available at https://data.nhm.ac.uk/object/7b5ff4bd-4101-4b03-98be-4cf9e7d09335); Malawi: L. Malawi: Bar House; C. Christy, 1925.

Thoracochromis albolabris (Trewavas & Thys van den Audenaerde, 1969): BMNH 1972.9.27.89, 1 (2D radiograph available at https://data.nhm.ac.uk/object/639516f6-eaf5-476f-8793-08e6be92d6ac); Angola: Cunene River: Epapa Falls; M.J. Penrith.

Thoracochromis wingatii (Boulenger, 1902): MCZ 148133, 1 (3D CT image series, MorphoSource http://n2t.net/ark:/87602/m4/M55324); Uganda: L. Albert: Botiaba lagoon; L.S. Kaufman & L. Victoria Research Team, 2-Jun-1996. — UMMZ 166656, 8 (1 used; 2D radiograph); Egypt: Al Mansouria, irrigation canal; 7-Jul-1953.

Steatocranini

Steatocranus casuarius Poll, 1939: USNM 331489, 3 (2D radiograph); Republic of the Congo: Kinshasa Province: rapids of Congo River at Kinsuka (near Kinshasa); T.R. Roberts, 20–23-Jun-1971.

Tilapiini

Chilochromis duponti Boulenger, 1902: CUMV 96467, 1 (3D CT image series, MorphoSource http://n2t.net/ark:/87602/m4/M69948); Gabon: Ngounie: Loétsi: Loétsi River at rocks below Bongolo Falls; M. Arnegard, B. Carlson, & J. Gallant, 4-Aug-2009.

Congolapia bilineata (Pellegrin, 1900): AMNH 238673, 3 (2D radiograph); DRC: Brazzaville, Lefini River above confluence of la Louna River; V. Mamonekene, 18-Sep-2004.

Tilapia busumana (Günther, 1903): BMNH 1942.12.30.52–61, 15 (4 used; 2D radiograph available at https://data.nhm.ac.uk/object/d01fbf5a-576b-4f01-acdf-cbc8ba5694ac); Ghana: Ashanti: Lake Busum Twi [Bosumtwi] at Dompa; N. Junner.

Tilapia sparrmanii Smith, 1840: BMNH 1859.5.7.85, 1 (lectotype; 2D radiograph available at https://data.nhm.ac.uk/object/d4bb76fc-84a4-4669-b953-aa176cb569f3); "Ceylon" [error]; A. Smith. — BMNH 1859.5.7.46, 2 (2D radiograph available at https://data.nhm.ac.uk/object/6acb545d-a50d-423a-9063-ca2d6b20926b); no locality given; A. Smith. — CUMV 91199, 1 (3D CT image series, MorphoSource http://n2t.net/ark:/87602/m4/M62748); Zambia: Central Province: Luapula River at Luapula River bridge; R. Bills, A. Chilala, & J. Friel, 22-Sep-2005.

Lake Barombi Mbo

Oreochromini

Konia eisentrauti (Trewavas, 1962): CUMV 93584, 1 (3D CT image series, MorphoSource http://n2t.net/ark:/87602/m4/M69924); Cameroon: Sud-Ouest Province: Lake Barombi Mbo; D. Reid, 28-Sep-2006. — UMMZ 220255, 6 (4 used; 2D radiograph); Cameroon: Lake Barombi Mbo; 1-Dec-1984.

Myaka myaka Trewavas, 1972: AMNH 271183, 4 (2D radiograph); Cameroon: Sud-Ouest Province: Lake Barombi Mbo; J.S. Cutler, 15-Aug-2015.

Pungu maclareni (Trewavas, 1962): BMNH 1959.8.18.177, 1 (holotype; 2D radiograph available at https://data.nhm.ac.uk/object/8ead47a0-11c0-4841-b36c-ad40330945b2); Cameroon: Lake Barombi-ba-Kotto; P.I. Maclaren, 1-Sep-1948. — CUMV 93585, 1 (3D CT image series, MorphoSource http://n2t.net/ark:/87602/m4/M61833); Cameroon: Sud-Ouest Province: Lake Barombi Mbo; D. Reid, 28-Sep-2006.

Stomatepia mariae (Holly, 1930): CUMV 93588, 1 (3D CT image series, MorphoSource http://n2t.net/ark:/87602/m4/M69938); Cameroon: Sud-Ouest Province: Lake Barombi Mbo; D. Reid, 28-Sep-2006.

Stomatepia pindu Trewavas, 1972: UMMZ 220257, 7 (4 used; 2D radiograph); Cameroon: Lake Barombi Mbo; 1-Dec-1984.

Lake Fwa

Pseudocrenilabrini

Cyclopharynx schwetzi (Poll, 1948): AMNH 247750, 14 (2D radiograph); DRC: Kasai Occidental: Lac Fwa, Mutanga Dibele; J. Mbimbi, 06-Jul-2008.

Schwetzochromis neodon Poll, 1948: AMNH 247754, 2 (2D radiograph); DRC: Kasai Occidental: Lac Fwa, Mutanga Dibele; J. Mbimbi, 07-Jul-2008.

Lake Albert

Pseudocrenilabrini

Haplochromis avium Regan, 1929: BMNH 1929.2.15.4–8, 5 (3 used; paratypes; 2D radiograph available at https://data.nhm.ac.uk/object/1ec024ad-1b6d-4991-ba96-a9725c56a42b); Uganda: L. Albert; E.B. Worthington.

Haplochromis loati Greenwood, 1971: BMNH 1909.7.27.42, 1 (2D radiograph available at https://data.nhm.ac.uk/object/6357363b-9de5-4e7f-af03-143c36e6e690); Uganda: Lake Albert: Butiaba.

Lake Edward–Lake George system

Pseudocrenilabrini

Haplochromis akika Lippitsch, 2003: BMNH 1972.6.2.784–787, 4 (holotype & paratypes; 2D radiograph available at https://data.nhm.ac.uk/object/9b3ad2b4-4b30-4c77-8ad2-89b7c5e01bea; note that this image labeled "holotype & paratypes" does not specify which is the holotype, but the original description (*Lippitsch 2003*) gives the length of the holotype as 76 mm SL and that of the paratypes as 67.0–77 mm SL. As the lowermost of the four specimens in the image is the second-longest and only slightly shorter than the longest, it is evidently the holotype); Uganda: L. George: bay of Akika Island; Royal Society–IBP expedition, 14-Dec-1971.

Haplochromis aquila Vranken, van Steenberge, Heylen, Decru, & Snoeks, 2022: RMCA 2018.008.P.0355, 1 (holotype; 2D radiograph in *Vranken et al. 2022*: fig. 21b); Uganda: L. Edward: Kayanja offshore; HIPE3 expedition, 20-Jan-2018.

Haplochromis aureus Vranken, Van Steenberge, Mbalassa, & Snoeks, 2023: RMCA 2019.002.P.0136, 1 (holotype; 2D radiograph in *Vranken et al. 2024*: fig. 6b); Uganda: L. Edward, pelagic catch, open water near border.

Haplochromis curvidens Vranken, van Steenberge, Heylen, Decru, & Snoeks, 2022: RMCA 2016.035.P.0219, 1 (holotype; 2D radiograph in *Vranken et al. 2022*: fig. 30b); Uganda: L. Edward: bought at Rwenshama landing site; HIPE1 expedition, 9-Nov-2016.

Haplochromis falcatus Vranken, van Steenberge, Heylen, Decru, & Snoeks, 2022: RMCA 2018.008.P.0401, 1 (holotype; 2D radiograph in *Vranken et al. 2022*: fig. 27b); Uganda: L. Edward: Kayanja offshore; HIPE3 expedition, 21-Jan-2018.

Haplochromis fuscus Regan, 1925: BMNH 1925.8.7.11, 1 (paratype; 2D radiograph available at https://data.nhm.ac.uk/object/d99c9876-f4de-4c6b-a9bf-324cc68c6512); DRC or Uganda: L. Edward; T. Barbour.

Haplochromis glaucus Vranken, van Steenberge, Heylen, Decru, & Snoeks, 2022: RMCA 2019.002.P.0016, 1 (holotype; 2D radiograph in *Vranken et al. 2022*: fig. 18b); Uganda: L. Edward: Kayanja offshore; HIPE4 expedition, 21-Mar-2019.

Haplochromis gracilifur Vranken, van Steenberge, & Snoeks, 2019: RMCA 2017.006.P.0004, 1 (holotype; 2D radiograph in *Vranken et al. 2019*: fig. S6b); Uganda: L. Edward: Kayanja offshore; HIPE2 expedition, 31-Mar-2017.

Haplochromis kimondo Vranken, van Steenberge, Heylen, Decru, & Snoeks, 2022: RMCA 2019.002.P.0015, 1 (holotype; 2D radiograph in *Vranken et al. 2022*: fig. 24b); Uganda: L. Edward: Kayanja offshore; HIPE4 expedition, 21-Mar-2019.

Haplochromis labiatus Trewavas, 1933: BMNH 1972.6.2.809, 1 (2D radiograph available at https://data.nhm.ac.uk/object/870920f0-04e7-4c52-940d-7c4aa1c9c739); Uganda: Lake George: E side Akika Island; P.H. Greenwood & I Dunn.

Haplochromis latifrons Vranken, van Steenberge, Heylen, Decru, & Snoeks, 2022: RMCA 2018.008.P.0330, 1 (holotype; 2D radiograph in *Vranken et al. 2022*: fig. 6b); Uganda: L. Edward: bought at Rwenshama landing site; HIPE3 expedition, 24-Jan-2018.

Haplochromis limax Trewavas, 1933: BMNH 1972.6.2.136–139, 4 (2D radiograph available at https://data.nhm.ac.uk/object/f7f729df-4c90-48ec-917c-e8c585837cf0); Uganda: Lake George, Kankuranga Island off papyrus shore; P.H. Greenwood & I. Dunn.

Haplochromis mentatus Regan, 1925: MCZ 31523, 1 (holotype; 2D radiograph in *Vranken et al. 2022*: fig. 9b); Uganda: L. Edward; J.C. Phillips, 1924.

Haplochromis molossus Vranken, van Steenberge, & Snoeks, 2019: RMCA 2016.035.P.0046, 1 (holotype; 2D radiograph in *Vranken et al. 2019*: fig. S4b); Uganda: L. Edward: Mukutu Kihinga, rocky offshore of Mweya; HIPE1 expedition, 23-Oct-2016.

Haplochromis pappenheimi (Boulenger, 1914): ZMB 19110, 1 (lectotype; 2D radiograph in *Vranken et al. 2024*: fig. 4b); Lake Edward; H. Schubotz, 1907–1908.

Haplochromis paradoxus (Lippitsch & Kaufman, 2003): RMCA 2017.006.P.0011, 1 (2D radiograph in *Vranken et al. 2019*: fig. S10b); Uganda: Lake Edward: Katwe town; 26-Jun-1994.

Haplochromis pardus Vranken, van Steenberge, Heylen, Decru, & Snoeks, 2022: RMCA 2016.035.P.0202, 1 (holotype; 2D radiograph in *Vranken et al. 2022*: fig. 33b); Uganda: L. Edward: Mukutu Kihinga, rocky offshore of Mweya; HIPE1 expedition, 23-Oct-2016.

Haplochromis pelagicus Vranken, Van Steenberge, Mbalassa, & Snoeks, 2023: RMCA 2019.002.P.0138, 1 (holotype; 2D radiograph in *Vranken et al. 2024*: fig. 5b); Uganda: L. Edward: pelagic catch, open water near border.

Haplochromis pharyngalis Poll & Damas, 1939: RMCA 65724, 1 (lectotype; 2D radiograph in *Vranken et al. 2020*: fig. S1b); DRC: L. Edward: Bugazia; H. Damas, 1935–1936.

Haplochromis quasimodo Vranken, van Steenberge, Heylen, Decru, & Snoeks, 2022: RMCA 2018.008.P.0336, 1 (holotype; 2D radiograph in *Vranken et al. 2022*: fig. 36b); Uganda: Lake Edward: 0°21′31.7′′ S, 29°43′17.7′′ E, deep catch, open water ± 30 m deep; HIPE3 expedition, 1-Feb-2018.

Haplochromis relictidens Vranken, van Steenberge, & Snoeks, 2018: RMCA 2017.006.P.0007, 1 (holotype; 2D radiograph in *Vranken et al. 2019*: fig. S8b); Uganda: L. Edward: mouth of Kazinga Channel; HIPE2 expedition, 24-Mar-2017.

Haplochromis rex Vranken, van Steenberge, Heylen, Decru, & Snoeks, 2022: RMCA 2018.008.P.0345, 1 (holotype; 2D radiograph in *Vranken et al. 2022*: fig. 12b); Uganda: L. Edward: bought at Rwenshama landing site; HIPE3 expedition, 24-Jan-2018.

Haplochromis simba Vranken, van Steenberge, Heylen, Decru, & Snoeks, 2022: RMCA 2016.035.P.0225, 1 (holotype; 2D radiograph in *Vranken et al. 2022*: fig. 15b); Uganda: L. Edward: bought at Rwenshama landing site; HIPE1 expedition, 9-Nov-2016.

Haplochromis squamipinnis Regan, 1921: BMNH 1914.4.8.32, 1 (holotype; 2D radiograph in *Vranken et al. 2022*: fig. 39b); Democratic Republic of the Congo (probably): Lake Edward; H. Schubotz, 1907–1908.

Haplochromis taurinus Trewavas, 1933: BMNH 1933.2.23.406, 1 (holotype; 2D radiograph in *Vranken et al. 2019*: fig. S2b); Democratic Republic of the Congo/ Uganda: L. Edward; Cambridge Expedition to the East African Lakes, 1930–1931.

Schubotzia eduardiana Boulenger, 1914: BMNH 1987.2.25.28–30, 3 (2D radiograph); Uganda: ¾ mile above bridge, Kazinga Channel; IBP team, 1972. — BMNH 1987.2.25.381–387, 7 (2D radiograph); Uganda: L. George: TUFMAC Bay; I. Dunn, 7-Dec-1971.

Lake Victoria and its satellites

Pseudocrenilabrini

Allochromis welcommei (Greenwood, 1966): MCZ 152827, 1 (3D CT image series, MorphoSource http://n2t.net/ark:/87602/m4/M44843); L. Nawampasa, near L. Kyoga; L. Victoria Research Team, W. Mwanja & P. Takeyama, 25–31-Jul-1997.

Astatoreochromis alluaudi Pellegrin, 1904: YPM 003830, 1 (3D CT image series, MorphoSource http://n2t.net/ark:/87602/m4/426545); L. Victoria.

Haplochromis chlorochrous Greenwood & Gee, 1969: BMNH 1968.8.30.311–328, 10 (4 used; paratypes; 2D radiograph available at https://data.nhm.ac.uk/object/e3e8b0cf-aafb-4251-a12e-2556f13ef883); Uganda: L. Victoria: between Nsadzi Island and the mainland in 21–30 m; EAFFRO.

Haplochromis cryptogramma Greenwood & Gee, 1969: BMNH 1968.8.30.198–214, 10 (paratypes; 2D radiograph available at https://data.nhm.ac.uk/object/79191640-e448-4557-8886-a2b93dce2b05); Uganda: L. Victoria: Bulago-Tavu Bank in 15–23 m; EAFFRO.

Haplochromis fusiformis Greenwood & Gee, 1969: MCZ 136638, 1 (3D CT image series, MorphoSource http://n2t.net/ark:/87602/m4/M55748); Uganda: L. Victoria: Napoleon Gulf: Bugungu; L. Victoria Research Team, M. Chandler, 15-Apr-1995.

Haplochromis latifasciatus Regan, 1929: BMNH 1929.1.24.335–339, 6 (syntypes; 2D radiograph available at https://data.nhm.ac.uk/object/6708d5fd-a886-4f09-abb5-f18ba065a577); Uganda: L. Kyoga; E.B. Worthington.

Haplochromis lividus Greenwood, 1956: AMNH 20484, 2 (2D radiograph); L. Victoria; Lerner African Expedition, 1937.

Haplochromis nubilus (Boulenger, 1906): AMNH 13747, 2 (det. by Greenwood; 2D radiograph); L. Victoria; Lerner African Expedition, 1937. — BMNH 1906.5.30.490, 1 (lectotype; 2D radiograph available at https://data.nhm.ac.uk/object/5736a1a5-816c-4923-a3a1-26478fc082e4); Uganda: L. Victoria at Entebbe; E. Degen.

Haplochromis plutonius Greenwood & Barel, 1978: BMNH 1977.1.10.40–48, 5 (paratypes; 2D radiograph available at https://data.nhm.ac.uk/object/bfc10e70-9919-4d34-a9c7-76e31634047e; note: radiograph ambiguously labeled as paratypes of both *H. plutonius* and *H. sulphureus*); Tanzania: L. Victoria: Speke Gulf, NE of Tefu Island; C.D. Barel & G. Anker, 5-Jun-1975.

Lithochromis rubripinnis Seehausen, Lippitsch, & Bouton, 1998: RMNH 33417, 33418, 33420, & 33423, 4 (paratypes; 2D radiograph); Tanzania: Lake Victoria: Mwanza Gulf.

Lithochromis xanthopteryx Seehausen & Bouton, 1998: RMNH 33435–33438, 4 (paratypes; 2D radiograph); Tanzania: Lake Victoria: Mwanza Gulf: Anchor Island; N. Bouton, Jun–Dec 1990.

Mbipia mbipi Seehausen, Lippitsch, & Bouton, 1998: MCZ 137947, 1 (3D CT image series, MorphoSource http://n2t.net/ark:/87602/m4/M48539); Kenya: L. Victoria: Mbita Point; L. Victoria Research Team, L.S. Kaufman, 1995.

Neochromis nigricans (Boulenger, 1906): AMNH 13743, 1 (2D radiograph); L. Victoria; 1937.

Paralabidochromis victoriae Greenwood, 1956: AMNH 13746, 1 (det. by Greenwood; 2D radiograph) ; L. Victoria; Lerner African Expedition, 1937. — MCZ 152956, 1 (2D x-ray CT, MorphoSource http://n2t.net/ark:/87602/m4/M49804); Uganda: L. Nyaguo, Kyoga system; L. Victoria Research Team, W. Mwanja & P. Takeyama; 25-Jul-1997.

Pundamilia igneopinnis Seehausen & Lippitsch, 1998: RMNH 33339–33340, 2 (paratypes; 2D radiograph); Tanzania: Lake Victoria: Speke Gulf: Igombe Island; O. Seehausen, N ov–Dec 1993.

Pundamilia pundamilia Seehausen & Bouton, 1998: RMNH 33380–33383, 4 (paratypes; 2D radiograph); Tanzania: Lake Victoria: Mwanza Gulf: Python Islands; O. Seehausen, Oct–Dec 1993.

Pyxichromis parorthostoma (Greenwood, 1967): BMNH 1966.3.9.253, 1 (paratype; 2D radiograph available at https://data.nhm.ac.uk/object/8a862d76-4d5e-44ac-babc-b26687fdf298); Uganda: L. Victoria: Buvuma Channel, near Sero Island; EAFRO.

Lake Kivu

Pseudocrenilabrini

Haplochromis astatodon Regan, 1921: BMNH 1906.9.6.125, 1 (lectotype; 2D radiograph available at https://data.nhm.ac.uk/object/dc149473-edc0-420c-bc8d-57b117f17505); DRC or Rwanda: L. Kivu; J.E. Moore. — BMNH 1906.9.6.126–129, 5 (paralectotypes; 2D radiograph available at https://data.nhm.ac.uk/object/5c790a5e-e1c3-4be7-aa51-9f7bbefd6125); DRC or Rwanda: L. Kivu; J.E. Moore.

Haplochromis paucidens Regan, 1921: BMNH 1906.9.6.72–73, 2 (syntypes; 2D radiograph available at https://data.nhm.ac.uk/object/5d310b77-c95d-4959-ba38-5d75cbf8a275); DRC or Rwanda: L. Kivu; J.E. Moore.

Lake Turkana

Pseudocrenilabrini

Haplochromis rudolfianus Trewavas, 1933: BMNH 1933.2.23.167, 2 (paralectotypes; 2D radiograph available at https://data.nhm.ac.uk/object/030123bd-621b-4b5b-842c-1c4b4123ef3e); Kenya: L. Turkana; E.B. Worthington. — BMNH 1965.12.10.16–27, 12 (6 used; 2D radiograph available at https://data.nhm.ac.uk/object/fac7603e-2102-4b09-8773-6a15ce51bfe2); Kenya: L. Turkana, crater lake, central island; R. Welcomme, 18-Aug-1965.

Haplochromis turkanae Greenwood, 1974: BMNH 1973.11.20.2–4, 3 (paratypes; 2D radiograph available at https://data.nhm.ac.uk/object/c916e72e-e4f5-4e58-92a6-08b972e5ff06); Kenya: L. Turkana: 3.5 miles NW of Porr; Lake Rudolf Fishery Research Project 1972, 10-Oct-1973.

Lake Tanganyika

Bathybatini

Bathybates fasciatus Boulenger, 1901: UNIBAS GPB3, 1 (2D x-ray CT, MorphoSource http://n2t.net/ark:/87602/m4/M155692); Zambia: L. Tanganyika: Mbita Island W; F. Ronco, A. Indermaur, H. Büscher, & W. Salzburger, 2-Sep-2014. — UNIBAS ITH8, 1 (2D x-ray CT, MorphoSource http://n2t.net/ark:/87602/m4/M155694); Tanzania: L. Tanganyika: Chipwa fishermen; F. Ronco, A. Indermaur, H. Büscher, & W. Salzburger, 24-Jul-2014. — UNIBAS JEG4, 1 (2D x-ray CT, MorphoSource http://n2t.net/ark:/87602/m4/M155695); L. Tanganyika: Kasenga W fishermen; F. Ronco, A. Indermaur, H. Büscher, & W. Salzburger, 22-Aug-2014. — UNIBAS JYF6, 1 (2D x-ray CT, MorphoSource http://n2t.net/ark:/87602/m4/M155696); Burundi: L. Tanganyika: Nyaruhongoka 2; F. Ronco, A. Indermaur, H. Büscher, & W. Salzburger, 10-Jan-2015. — UNIBAS KYD1, 1 (2D x-ray CT, MorphoSource

http://n2t.net/ark:/87602/m4/M155697); Tanzania: Ujiji fishmarket; F. Ronco, A. Indermaur, H. Büscher, & W. Salzburger, 13-Jul-2015.

Bathybates ferox Boulenger, 1898: UNIBAS LCD6, 1 (2D x-ray CT, MorphoSource http://n2t.net/ark:/87602/m4/M155718); L. Tanganyika: Nganja; F. Ronco, A. Indermaur, H. Büscher, & W. Salzburger, 2-Jul-2015. — UNIBAS LCD7, 1 (2D x-ray CT, MorphoSource http://n2t.net/ark:/87602/m4/M155719); other data same as LCD6. — UNIBAS LCD8, 1 (2D x-ray CT, MorphoSource http://n2t.net/ark:/87602/m4/M155720); other data same as LCD6. — UNIBAS LCD9, 1 (2D x-ray CT, MorphoSource http://n2t.net/ark:/87602/m4/M155721); other data same as LCD6. — UNIBAS LCE1, 1 (2D x-ray CT, MorphoSource http://n2t.net/ark:/87602/m4/M155722); other data same as LCD6. — UNIBAS LCE2, 1 (2D x-ray CT, MorphoSource http://n2t.net/ark:/87602/m4/M155723); other data same as LCD6. — UNIBAS LCE3, 1 (2D x-ray CT, MorphoSource http://n2t.net/ark:/87602/m4/M155724); other data same as LCD6. — UNIBAS LCE4, 1 (2D x-ray CT, MorphoSource http://n2t.net/ark:/87602/m4/M155725); other data same as LCD6. — UNIBAS LCE5, 1 (2D x-ray CT, MorphoSource http://n2t.net/ark:/87602/m4/M155726); other data same as LCD6. — UNIBAS LCE6, 1 (2D x-ray CT, MorphoSource http://n2t.net/ark:/87602/m4/M155727); other data same as LCD6.

Bathybates graueri Steindachner, 1911: UNIBAS IUH6, 1 (2D x-ray CT, MorphoSource http://n2t.net/ark:/87602/m4/M155795); Tanzania: L. Tanganyika: Chipwa fishermen; F. Ronco, A. Indermaur, H. Büscher, & W. Salzburger, 25-Jul-2014. — UNIBAS IUH8, 1 (2D x-ray CT, MorphoSource http://n2t.net/ark:/87602/m4/M155797); Zambia: Lake Tanganyika: Kalambo (Toby's) Lodge; F. Ronco, A. Indermaur, H. Büscher, & W. Salzburger, 25-Jul-2014. — IUH9, 1 (2D x-ray CT, MorphoSource http://n2t.net/ark:/87602/m4/M155798); other data same as IUH8. — UNIBAS IXG2, 1 (2D x-ray CT, MorphoSource http://n2t.net/ark:/87602/m4/M155799); Tanzania: L. Tanganyika: Chipwa fishermen; F. Ronco, A. Indermaur, H. Büscher, & W. Salzburger, 27-Jul-2014. — UNIBAS IXG3, 1 (2D x-ray CT, MorphoSource http://n2t.net/ark:/87602/m4/M155800); other data same as IXG2.

Bathybates hornii Steindachner, 1911: UNIBAS MOD4, 1 (2D x-ray CT, MorphoSource http://n2t.net/ark:/87602/m4/M155842); Zambia: L. Tanganyika: Mpulungu fishmarket; F. Ronco, A. Indermaur, H. Büscher, & W. Salzburger, 1-Sep-2016.

Bathybates leo Poll, 1956: UNIBAS KCB8, 1 (2D x-ray CT, MorphoSource http://n2t.net/ark:/87602/m4/M155920); Burundi: L. Tanganyika: Bujumbura fishmarket; F. Ronco, A. Indermaur, H. Büscher, & W. Salzburger, 15-Jan-2015. — UNIBAS KCB9, 1 (2D x-ray CT, MorphoSource http://n2t.net/ark:/87602/m4/M155921); other data same as KCB8. — UNIBAS KCC1, 1 (2D x-ray CT, MorphoSource http://n2t.net/ark:/87602/m4/M155922); other data same as KCB8. — UNIBAS KCC2, 1 (2D x-ray CT, MorphoSource http://n2t.net/ark:/87602/m4/M155923); other data same as KCB8. — UNIBAS KCC5, 1 (2D x-ray CT, MorphoSource http://n2t.net/ark:/87602/m4/M155924); other data same as KCB8.

Bathybates minor Boulenger, 1906: UNIBAS JEF6, 1 (2D x-ray CT, MorphoSource http://n2t.net/ark:/87602/m4/M156163); L. Tanganyika: Kasenga W fishermen; F. Ronco, A. Indermaur,

H. Büscher, & W. Salzburger, 22-Aug-2014. — UNIBAS JEF7, 1 (2D x-ray CT, MorphoSource http://n2t.net/ark:/87602/m4/M156164); other data same as JEF6. — UNIBAS JEF8, 1 (2D x-ray CT, MorphoSource http://n2t.net/ark:/87602/m4/M156165); other data same as JEF6. — UNIBAS JEF9, 1 (2D x-ray CT, MorphoSource http://n2t.net/ark:/87602/m4/M156166); other data same as JEF6. — UNIBAS JEG2, 1 (2D x-ray CT, MorphoSource http://n2t.net/ark:/87602/m4/M156167); other data same as JEF6.

Bathybates vittatus Boulenger, 1914: UNIBAS LNH9, 1 (2D x-ray CT, MorphoSource http://n2t.net/ark:/87602/m4/M157226); Tanzania: L. Tanganyika: Chipwa fishermen; F. Ronco, A. Indermaur, H. Büscher, & W. Salzburger, 23-Aug-2016.

Hemibates stenosoma (Boulenger, 1901): UNIBAS IXC5, 1 (2D x-ray CT, MorphoSource http://n2t.net/ark:/87602/m4/M157096); Tanzania: L. Tanganyika: Chipwa fishermen; F. Ronco, A. Indermaur, H. Büscher, & W. Salzburger, 27-Jul-2014. — UNIBAS IXC6, 1 (2D x-ray CT, MorphoSource http://n2t.net/ark:/87602/m4/M157097); other data same as IXC5. — UNIBAS IXC7, 1 (2D x-ray CT, MorphoSource http://n2t.net/ark:/87602/m4/M157098); other data same as IXC5. — UNIBAS IXC8, 1 (2D x-ray CT, MorphoSource http://n2t.net/ark:/87602/m4/M157099); other data same as IXC5.

Trematocara unimaculatum Boulenger, 1901: UNIBAS IXF2, 1 (2D x-ray CT, MorphoSource http://n2t.net/ark:/87602/m4/M157203); Tanzania: L. Tanganyika: Chipwa fishermen; F. Ronco, A. Indermaur, H. Büscher, & W. Salzburger, 27-Jul-2014. — UNIBAS IXF5, 1 (2D x-ray CT, MorphoSource http://n2t.net/ark:/87602/m4/M157205); other data same as IXF2. — UNIBAS IXF6, 1 (2D x-ray CT, MorphoSource http://n2t.net/ark:/87602/m4/M157206); other data same as IXF2. — UNIBAS IXF7, 1 (2D x-ray CT, MorphoSource http://n2t.net/ark:/87602/m4/M157207); other data same as IXF2. — UNIBAS IXG1, 1 (2D x-ray CT, MorphoSource http://n2t.net/ark:/87602/m4/M157209); other data same as IXF2.

Trematocara zebra De Vos, Nshombo, & Thys van den Audenaerde, 1996: UNIBAS LFE7, 1 (2D x-ray CT, MorphoSource http://n2t.net/ark:/87602/m4/M157250); Tanzania: L. Tanganyika: Korongwe; F. Ronco, A. Indermaur, H. Büscher, & W. Salzburger, 15-Aug-2015. — UNIBAS LFE8, 1 (2D x-ray CT, MorphoSource http://n2t.net/ark:/87602/m4/M157251); other data same as LFE7. — UNIBAS LFE9, 1 (2D x-ray CT, MorphoSource http://n2t.net/ark:/87602/m4/M157252); other data same as LFE7. — UNIBAS LFF2, 1 (2D x-ray CT, MorphoSource http://n2t.net/ark:/87602/m4/M157253); other data same as LFE7. — UNIBAS LFF3, 1 (2D x-ray CT, MorphoSource http://n2t.net/ark:/87602/m4/M157254); other data same as LFE7.

Benthochromini

Benthochromis tricoti (Poll, 1948): UNIBAS LDB4, 1 (2D x-ray CT, MorphoSource http://n2t.net/ark:/87602/m4/M157196); Tanzania: L. Tanganyika: Chipwa fishermen; F. Ronco, A. Indermaur, H. Büscher, & W. Salzburger, 7-Aug-2015. — UNIBAS LDB5, 1 (2D x-ray CT, MorphoSource http://n2t.net/ark:/87602/m4/M157197); other data same as LDB4. — UNIBAS LDB6, 1 (2D x-ray CT, MorphoSource http://n2t.net/ark:/87602/m4/M157198); other data same as LDB4. — UNIBAS LDB7, 1 (2D x-ray CT, MorphoSource http://n2t.net/ark:/87602/m4/M157199); other data same as LDB4. —

UNIBAS LDB8, 1 (2D x-ray CT, MorphoSource http://n2t.net/ark:/87602/m4/M157200); other data same as LDB4.

Boulengerochromini

Boulengerochromis microlepis (Boulenger, 1899): AMNH 11727, 2 (2D radiograph); Tanzania: L. Tanganyika; H.C. Raven, 1929. — UNIBAS ILI6, 1 (2D x-ray CT, MorphoSource http://n2t.net/ark:/87602/m4/M156127); Zambia: L. Tanganyika: Kombe fishermen; F. Ronco, A. Indermaur, H. Büscher, & W. Salzburger, 16-Aug-2014. — UNIBAS ITH6, 1 (2D x-ray CT, MorphoSource http://n2t.net/ark:/87602/m4/M156130); Tanzania: L. Tanganyika: Chipwa fishermen; F. Ronco, A. Indermaur, H. Büscher, & W. Salzburger, 24-Jul-2014. — UNIBAS ITH7, 1 (2D x-ray CT, MorphoSource http://n2t.net/ark:/87602/m4/M156131); other data same as ITH6. — UNIBAS IZB6, 1 (2D x-ray CT, MorphoSource http://n2t.net/ark:/87602/m4/M156132); 28-Jul-2014, other data same as ITH6. — UNIBAS JBI8, 1 (2D x-ray CT, MorphoSource http://n2t.net/ark:/87602/m4/M156133); Zambia: L. Tanganyika: Mbita Island W; F. Ronco, A. Indermaur, H. Büscher, & W. Salzburger, 2-Sep-2014.

Cyphotilapiini

Cyphotilapia frontosa (Boulenger, 1906): UNIBAS LEI5, 1 (2D x-ray CT, MorphoSource http://n2t.net/ark:/87602/m4/M155752); Tanzania: L. Tanganyika: Kalalangabo; F. Ronco, A. Indermaur, H. Büscher, & W. Salzburger, 10-Jul-2015. — UNIBAS LEI6, 1 (2D x-ray CT, MorphoSource http://n2t.net/ark:/87602/m4/M155753); Tanzania: L. Tanganyika: Nondwa Point; F. Ronco, A. Indermaur, H. Büscher, & W. Salzburger, 10-Jul-2015. — UNIBAS LEI7, 1 (2D x-ray CT, MorphoSource http://n2t.net/ark:/87602/m4/M155754); other data same as LEI6. — UNIBAS LEI8, 1 (2D x-ray CT, MorphoSource http://n2t.net/ark:/87602/m4/M155755); other data same as LEI6. — UNIBAS LEI9, 1 (2D x-ray CT, MorphoSource http://n2t.net/ark:/87602/m4/M155756); other data same as LEI6.

Cyphotilapia gibberosa Takahashi & Nakaya, 2003 (considered a synonym of *C. frontosa* by some authors): UNIBAS JEA9, 1 (2D x-ray CT, MorphoSource http://n2t.net/ark:/87602/m4/M155783); L. Tanganyika: Misepa; F. Ronco, A. Indermaur, H. Büscher, & W. Salzburger, 20-Aug-2014. — UNIBAS JBG1, 1 (2D x-ray CT, MorphoSource http://n2t.net/ark:/87602/m4/M155781); Zambia: L. Tanganyika: Kanfonki; F. Ronco, A. Indermaur, H. Büscher, & W. Salzburger, 1-Sep-2014. — UNIBAS JEA8, 1 (2D x-ray CT, MorphoSource http://n2t.net/ark:/87602/m4/M155782); other data same as JEA9. — UNIBAS JEB1, 1 (2D x-ray CT, MorphoSource http://n2t.net/ark:/87602/m4/M155784); other data same as JEA9. — UNIBAS JED6, 1 (2D x-ray CT, MorphoSource http://n2t.net/ark:/87602/m4/M155785); Zambia: L. Tanganyika: Mibwebwe; F. Ronco, A. Indermaur, H. Büscher, & W. Salzburger, 21-Aug-2014.

Cyprichromini

Cyprichromis coloratus Takahashi & Hori, 2006: UNIBAS ITF7, 1 (2D x-ray CT, MorphoSource http://n2t.net/ark:/87602/m4/M155509); Zambia: Lake Tanganyika: Kalambo (Toby's) Lodge; F. Ronco, A. Indermaur, H. Büscher, & W. Salzburger, 24-Jul-2014. — UNIBAS JEC7, 1 (2D x-ray CT, MorphoSource http://n2t.net/ark:/87602/m4/M155510); Zambia: Lake Tanganyika: Chitweshiba; F. Ronco, A. Indermaur, H. Büscher, & W. Salzburger, 20-Aug-2014. — UNIBAS JEC8, 1 (2D x-ray CT, MorphoSource

http://n2t.net/ark:/87602/m4/M155511); other data same as JEC7. — UNIBAS JEC9, 1 (2D x-ray CT, MorphoSource http://n2t.net/ark:/87602/m4/M155512); other data same as JEC7. — UNIBAS JED1, 1 (2D x-ray CT, MorphoSource http://n2t.net/ark:/87602/m4/M155513); other data same as JEC7.

Cyprichromis leptosoma (Boulenger, 1898): UNIBAS ITC7, 1 (2D x-ray CT, MorphoSource http://n2t.net/ark:/87602/m4/M155930); Tanzania: L. Tanganyika: Chipwa fishermen; F. Ronco, A. Indermaur, H. Büscher, & W. Salzburger, 24-Jul-2014. — UNIBAS ITE6, 1 (2D x-ray CT, MorphoSource http://n2t.net/ark:/87602/m4/M155931); other data same as ITC7. — UNIBAS ITE8, 1 (2D x-ray CT, MorphoSource http://n2t.net/ark:/87602/m4/M155933); other data same as ITC7. — UNIBAS ITE9, 1 (2D x-ray CT, MorphoSource http://n2t.net/ark:/87602/m4/M155934); other data same as ITC7. — UNIBAS ITF1, 1 (2D x-ray CT, MorphoSource http://n2t.net/ark:/87602/m4/M155935); other data same as ITC7.

Cyprichromis microlepidotus (Poll, 1956): UNIBAS JVE9, 1 (2D x-ray CT, MorphoSource http://n2t.net/ark:/87602/m4/M156120); Burundi: L. Tanganyika: Nyaruhongoka 2; F. Ronco, A. Indermaur, H. Büscher, & W. Salzburger, 7-Jan-2015. — UNIBAS JVF1, 1 (2D x-ray CT, MorphoSource http://n2t.net/ark:/87602/m4/M156121); other data same as JVE9. — UNIBAS JVF5, 1 (2D x-ray CT, MorphoSource http://n2t.net/ark:/87602/m4/M156123); other data same as JVE9. — UNIBAS KDG3, 1 (2D x-ray CT, MorphoSource http://n2t.net/ark:/87602/m4/M156124); 24-Jan-2015, other data same as JVE9. — UNIBAS KDG4, 1 (2D x-ray CT, MorphoSource http://n2t.net/ark:/87602/m4/M156125); other data same as KDG3.

Cyprichromis pavo Büscher, 1994: UNIBAS JEC1, 1 (2D x-ray CT, MorphoSource http://n2t.net/ark:/87602/m4/M156414); L. Tanganyika: Misepa; F. Ronco, A. Indermaur, H. Büscher, & W. Salzburger, 20-Aug-2014. — UNIBAS JEC2, 1 (2D x-ray CT, MorphoSource http://n2t.net/ark:/87602/m4/M156415); other data same as JEC1. — UNIBAS JEC4, 1 (2D x-ray CT, MorphoSource http://n2t.net/ark:/87602/m4/M156417); other data same as JEC1. — UNIBAS JEC5, 1 (2D x-ray CT, MorphoSource http://n2t.net/ark:/87602/m4/M156418); other data same as JEC1. — UNIBAS JEC6, 1 (2D x-ray CT, MorphoSource http://n2t.net/ark:/87602/m4/M156419); Zambia: Lake Tanganyika: Chitweshiba; F. Ronco, A. Indermaur, H. Büscher, & W. Salzburger, 20-Aug-2014.

Cyprichromis zonatus Takahashi, Hori, & Nakaya, 2002: UNIBAS LBI8, 1 (2D x-ray CT, MorphoSource http://n2t.net/ark:/87602/m4/M157261); Zambia: L. Tanganyika: Chituta; F. Ronco, A. Indermaur, H. Büscher, & W. Salzburger, 6-Aug-2015. — UNIBAS LBI9, 1 (2D x-ray CT, MorphoSource http://n2t.net/ark:/87602/m4/M157262); other data same as LBI8. — UNIBAS LDA1, 1 (2D x-ray CT, MorphoSource http://n2t.net/ark:/87602/m4/M157263); other data same as LBI8. — UNIBAS LDA2, 1 (2D x-ray CT, MorphoSource http://n2t.net/ark:/87602/m4/M157264); other data same as LBI8. — UNIBAS LDA3, 1 (2D x-ray CT, MorphoSource http://n2t.net/ark:/87602/m4/M157265); other data same as LBI8.

Cyprichromis sp. “dwarf jumbo”: UNIBAS KFA5, 1 (2D x-ray CT, MorphoSource http://n2t.net/ark:/87602/m4/M156670); Tanzania: L. Tanganyika: cave Kigoma; F. Ronco, A. Indermaur, H. Büscher, & W. Salzburger, 20-Jun-2015. — UNIBAS KFB2, 1 (2D x-ray CT, MorphoSource

http://n2t.net/ark:/87602/m4/M156672); other data same as KFA5. — UNIBAS KFB3, 1 (2D x-ray CT, MorphoSource http://n2t.net/ark:/87602/m4/M156673); other data same as KFA5. — UNIBAS KFB4, 1 (2D x-ray CT, MorphoSource http://n2t.net/ark:/87602/m4/M156674); other data same as KFA5. — UNIBAS KFB5, 1 (2D x-ray CT, MorphoSource http://n2t.net/ark:/87602/m4/M156675); other data same as KFA5.

Cyprichromis sp. "jumbo": UNIBAS INH5, 1 (2D x-ray CT, MorphoSource http://n2t.net/ark:/87602/m4/M156680); "Kanfonki"; F. Ronco, A. Indermaur, H. Büscher, & W. Salzburger, 17-Aug-2014. — UNIBAS MOD7, 1 (2D x-ray CT, MorphoSource http://n2t.net/ark:/87602/m4/M156682); 2-Sep-2016, other data same as INH5. — UNIBAS MOD8, 1 (2D x-ray CT, MorphoSource http://n2t.net/ark:/87602/m4/M156683); other data same as MOD7. — UNIBAS MOD9, 1 (2D x-ray CT, MorphoSource http://n2t.net/ark:/87602/m4/M156684); other data same as MOD7. — UNIBAS MOE1, 1 (2D x-ray CT, MorphoSource http://n2t.net/ark:/87602/m4/M156685); other data same as MOD7.

Paracyprichromis brieni (Poll, 1981): UNIBAS JXI7, 1 (2D x-ray CT, MorphoSource http://n2t.net/ark:/87602/m4/M155381); Burundi: L. Tanganyika: Nyaruhongoka 2; F. Ronco, A. Indermaur, H. Büscher, & W. Salzburger, 9-Jan-2015. — UNIBAS JXI8, 1 (2D x-ray CT, MorphoSource http://n2t.net/ark:/87602/m4/M155382); other data same as JXI7. — UNIBAS JYA1, 1 (2D x-ray CT, MorphoSource http://n2t.net/ark:/87602/m4/M155384); other data same as JXI7. — UNIBAS JYA2, 1 (2D x-ray CT, MorphoSource http://n2t.net/ark:/87602/m4/M155385); other data same as JXI7. — UNIBAS JYA3, 1 (2D x-ray CT, MorphoSource http://n2t.net/ark:/87602/m4/M155386); other data same as JXI7.

Paracyprichromis nigripinnis (Boulenger, 1901): UNIBAS GPD8, 1 (2D x-ray CT, MorphoSource http://n2t.net/ark:/87602/m4/M156278); Zambia: L. Tanganyika: Chituta; F. Ronco, A. Indermaur, H. Büscher, & W. Salzburger, 3-Sep-2014. — UNIBAS GPD9, 1 (2D x-ray CT, MorphoSource http://n2t.net/ark:/87602/m4/M156279); other data same as GPD8. — UNIBAS GPE3, 1 (2D x-ray CT, MorphoSource http://n2t.net/ark:/87602/m4/M156282); other data same as GPD8. — UNIBAS GPE5, 1 (2D x-ray CT, MorphoSource http://n2t.net/ark:/87602/m4/M156284); other data same as GPD8.

Paracyprichromis sp. "brieni south": UNIBAS IQE6, 1 (2D x-ray CT, MorphoSource http://n2t.net/ark:/87602/m4/M156862); Zambia: Lake Tanganyika: Kalambo (Toby's) Lodge; F. Ronco, A. Indermaur, H. Büscher, & W. Salzburger, 22-Jul-2014. — UNIBAS ITD8, 1 (2D x-ray CT, MorphoSource http://n2t.net/ark:/87602/m4/M156864); Tanzania: L. Tanganyika: Chipwa fishermen; F. Ronco, A. Indermaur, H. Büscher, & W. Salzburger, 24-Jul-2014. — UNIBAS ITD9, 1 (2D x-ray CT, MorphoSource http://n2t.net/ark:/87602/m4/M156865); other data same as ITD8. — UNIBAS ITE1, 1 (2D x-ray CT, MorphoSource http://n2t.net/ark:/87602/m4/M156866); other data same as ITD8. — UNIBAS ITE2, 1 (2D x-ray CT, MorphoSource http://n2t.net/ark:/87602/m4/M156867); other data same as ITD8.

Ectodini

Asprotilapia leptura Boulenger, 1901: UNIBAS INF8, 1 (2D x-ray CT, MorphoSource http://n2t.net/ark:/87602/m4/M155940); Zambia: L. Tanganyika: Kanfonki; F. Ronco, A. Indermaur, H.

Büscher, & W. Salzburger, 17-Aug-2014. — UNIBAS ING1, 1 (2D x-ray CT, MorphoSource http://n2t.net/ark:/87602/m4/M155942); other data same as INF8. — UNIBAS ING2, 1 (2D x-ray CT, MorphoSource http://n2t.net/ark:/87602/m4/M155943); other data same as INF8. — UNIBAS ING3, 1 (2D x-ray CT, MorphoSource http://n2t.net/ark:/87602/m4/M155944); other data same as INF8. — UNIBAS JDC9, 1 (2D x-ray CT, MorphoSource http://n2t.net/ark:/87602/m4/M155945); Zambia: Lake Tanganyika: Kalambo (Toby's) Lodge; F. Ronco, A. Indermaur, H. Büscher, & W. Salzburger, 1-Aug-2014.

Aulonocranus dewindti (Boulenger, 1899): UNIBAS IPH5, 1 (2D x-ray CT, MorphoSource http://n2t.net/ark:/87602/m4/M155587); Zambia: Lake Tanganyika: Kalambo (Toby's) Lodge; F. Ronco, A. Indermaur, H. Büscher, & W. Salzburger, 21-Jul-2014. — UNIBAS IYH5, 1 (2D x-ray CT, MorphoSource http://n2t.net/ark:/87602/m4/M155590); 29-Jul-2014, other data same as IPH5. — IYI1, 1 (2D x-ray CT, MorphoSource http://n2t.net/ark:/87602/m4/M155591); other data same as IYH5. — UNIBAS IYI2, 1 (2D x-ray CT, MorphoSource http://n2t.net/ark:/87602/m4/M155592); other data same as IYH5. — UNIBAS IYI6, 1 (2D x-ray CT, MorphoSource http://n2t.net/ark:/87602/m4/M155593); other data same as IYH5.

Callochromis macrops (Boulenger, 1898): UNIBAS JCH5, 1 (2D x-ray CT, MorphoSource http://n2t.net/ark:/87602/m4/M155998); Zambia: Lake Tanganyika: Kalambo (Toby's) Lodge; F. Ronco, A. Indermaur, H. Büscher, & W. Salzburger, 1-Aug-2014. — UNIBAS JCI1, 1 (2D x-ray CT, MorphoSource http://n2t.net/ark:/87602/m4/M156001); other data same as JCH5. — UNIBAS JCI4, 1 (2D x-ray CT, MorphoSource http://n2t.net/ark:/87602/m4/M156002); other data same as JCH5. — UNIBAS JCI5, 1 (2D x-ray CT, MorphoSource http://n2t.net/ark:/87602/m4/M156003); other data same as JCH5. — UNIBAS JCI6, 1 (2D x-ray CT, MorphoSource http://n2t.net/ark:/87602/m4/M156004); other data same as JCH5.

Cardiopharynx schoutedeni Poll, 1942: UNIBAS KCA7, 1 (2D x-ray CT, MorphoSource http://n2t.net/ark:/87602/m4/M156564); Burundi: L. Tanganyika: Bujumbura fishmarket; F. Ronco, A. Indermaur, H. Büscher, & W. Salzburger, 15-Jan-2015. — UNIBAS KCA8, 1 (2D x-ray CT, MorphoSource http://n2t.net/ark:/87602/m4/M156565); other data same as KCA7. — UNIBAS KCB1, 1 (2D x-ray CT, MorphoSource http://n2t.net/ark:/87602/m4/M156567); other data same as KCA7. — UNIBAS KCB2, 1 (2D x-ray CT, MorphoSource http://n2t.net/ark:/87602/m4/M156568); other data same as KCA7. — UNIBAS KCB4, 1 (2D x-ray CT, MorphoSource http://n2t.net/ark:/87602/m4/M156570); other data same as KCA7.

Cunningtonia longiventralis Boulenger, 1906: UNIBAS IWE5, 1 (2D x-ray CT, MorphoSource http://n2t.net/ark:/87602/m4/M155971); L. Tanganyika: Ntingila; F. Ronco, A. Indermaur, H. Büscher, & W. Salzburger, 27-Aug-2014. — UNIBAS IWE6, 1 (2D x-ray CT, MorphoSource http://n2t.net/ark:/87602/m4/M155972); other data same as IWE5. — UNIBAS IWE7, 1 (2D x-ray CT, MorphoSource http://n2t.net/ark:/87602/m4/M155973); other data same as IWE5. — UNIBAS IWE8, 1 (2D x-ray CT, MorphoSource http://n2t.net/ark:/87602/m4/M155974); other data same as IWE5. — UNIBAS IWE9, 1 (2D x-ray CT, MorphoSource http://n2t.net/ark:/87602/m4/M155975); other data same as IWE5.

Cyathopharynx furcifer (Boulenger, 1898): UNIBAS IQI1, 1 (2D x-ray CT, MorphoSource http://n2t.net/ark:/87602/m4/M155761); Zambia: Lake Tanganyika: Kalambo (Toby's) Lodge; F. Ronco, A. Indermaur, H. Büscher, & W. Salzburger, 22-Jul-2014. — UNIBAS ISF4, 1 (2D x-ray CT, MorphoSource http://n2t.net/ark:/87602/m4/M155762); 23-Jul-2014, other data same as IQI1. — UNIBAS ISF5, 1 (2D x-ray CT, MorphoSource http://n2t.net/ark:/87602/m4/M155763); other data same as ISF4. — UNIBAS IUH1, 1 (2D x-ray CT, MorphoSource http://n2t.net/ark:/87602/m4/M155764); 25-Jul-2014, other data same as IQI1. — UNIBAS IUH2, 1 (2D x-ray CT, MorphoSource http://n2t.net/ark:/87602/m4/M155765); other data same as IUH1.

Ectodus descampsii Boulenger, 1898: UNIBAS IRE9, 1 (2D x-ray CT, MorphoSource http://n2t.net/ark:/87602/m4/M155578); Zambia: L. Tanganyika: Ndole Bay harbor; F. Ronco, A. Indermaur, H. Büscher, & W. Salzburger, 25-Aug-2014. — UNIBAS IRF1, 1 (2D x-ray CT, MorphoSource http://n2t.net/ark:/87602/m4/M155579); other data same as IRE9. — UNIBAS IRF2, 1 (2D x-ray CT, MorphoSource http://n2t.net/ark:/87602/m4/M155580); other data same as IRE9. — UNIBAS IRF3, 1 (2D x-ray CT, MorphoSource http://n2t.net/ark:/87602/m4/M155581); other data same as IRE9. — UNIBAS IRF5, 1 (2D x-ray CT, MorphoSource http://n2t.net/ark:/87602/m4/M155583); other data same as IRE9.

Enantiopus melanogenys (Boulenger, 1898): UNIBAS IVB2, 1 (2D x-ray CT, MorphoSource http://n2t.net/ark:/87602/m4/M156082); L. Tanganyika: Ntingila; F. Ronco, A. Indermaur, H. Büscher, & W. Salzburger, 25-Aug-2014. — UNIBAS JCB9, 1 (2D x-ray CT, MorphoSource http://n2t.net/ark:/87602/m4/M156084); Zambia: Lake Tanganyika: Kalambo (Toby's) Lodge; F. Ronco, A. Indermaur, H. Büscher, & W. Salzburger, 31-Jul-2014. — UNIBAS JCC1, 1 (2D x-ray CT, MorphoSource http://n2t.net/ark:/87602/m4/M156085); other data same as JCB9. — UNIBAS JCC2, 1 (2D x-ray CT, MorphoSource http://n2t.net/ark:/87602/m4/M156086); other data same as JCB9. — UNIBA JCC3, 1 (2D x-ray CT, MorphoSource http://n2t.net/ark:/87602/m4/M156087); other data same as JCB9.

Grammatotria lemairii Boulenger, 1899: UNIBAS IOD3, 1 (2D x-ray CT, MorphoSource http://n2t.net/ark:/87602/m4/M155906); Zambia: L. Tanganyika: Kachese; F. Ronco, A. Indermaur, H. Büscher, & W. Salzburger, 23-Aug-2014. — UNIBAS IUG9, 1 (2D x-ray CT, MorphoSource http://n2t.net/ark:/87602/m4/M155907); Zambia: Lake Tanganyika: Kalambo (Toby's) Lodge; F. Ronco, A. Indermaur, H. Büscher, & W. Salzburger, 25-Jul-2014. — UNIBAS IWG6, 1 (2D x-ray CT, MorphoSource http://n2t.net/ark:/87602/m4/M155908); Zambia: L. Tanganyika: Ndole fishermen; F. Ronco, A. Indermaur, H. Büscher, & W. Salzburger, 28-Aug-2014. — UNIBAS JBC5, 1 (2D x-ray CT, MorphoSource http://n2t.net/ark:/87602/m4/M155909); Zambia: Lake Tanganyika: Chitweshiba; F. Ronco, A. Indermaur, H. Büscher, & W. Salzburger, 31-Aug-2014. — UNIBAS JBC6, 1 (2D x-ray CT, MorphoSource http://n2t.net/ark:/87602/m4/M155910); other data same as JBC5.

Lestradea perspicax Poll, 1943: UNIBAS IOI1, 1 (2D x-ray CT, MorphoSource http://n2t.net/ark:/87602/m4/M156438); Zambia: L. Tanganyika: Chimba; F. Ronco, A. Indermaur, H. Büscher, & W. Salzburger, 24-Aug-2014. — UNIBAS IOI3, 1 (2D x-ray CT, MorphoSource http://n2t.net/ark:/87602/m4/M156440); other data same as IOI1. — UNIBAS IOI5, 1 (2D x-ray CT, MorphoSource http://n2t.net/ark:/87602/m4/M156442); other data same as IOI1. — UNIBAS IOI6, 1

(2D x-ray CT, MorphoSource http://n2t.net/ark:/87602/m4/M156443); other data same as IOI1. — UNIBAS IOI7, 1 (2D x-ray CT, MorphoSource http://n2t.net/ark:/87602/m4/M156444); other data same as IOI1.

Microdontochromis tenuidentatus (Poll, 1951): UNIBAS LHH4, 1 (2D x-ray CT, MorphoSource http://n2t.net/ark:/87602/m4/M157136); Tanzania: L. Tanganyika: Malasa Bay; F. Ronco, A. Indermaur, H. Büscher, & W. Salzburger, 23-Aug-2015. — UNIBAS LHH5, 1 (2D x-ray CT, MorphoSource http://n2t.net/ark:/87602/m4/M157137); other data same as LHH4. — LHH6, 1 (2D x-ray CT, MorphoSource http://n2t.net/ark:/87602/m4/M157138); other data same as LHH4. — UNIBAS LHH7, 1 (2D x-ray CT, MorphoSource http://n2t.net/ark:/87602/m4/M157139); other data same as LHH4. — UNIBAS LHH8, 1 (2D x-ray CT, MorphoSource http://n2t.net/ark:/87602/m4/M157140); other data same as LHH4.

Ophthalmotilapia boops (Boulenger, 1901): UNIBAS LFI5, 1 (2D x-ray CT, MorphoSource http://n2t.net/ark:/87602/m4/M155294); Tanzania: L. Tanganyika: Nkondwe; F. Ronco, A. Indermaur, H. Büscher, & W. Salzburger, 17-Aug-2015. — UNIBAS LFI8, 1 (2D x-ray CT, MorphoSource http://n2t.net/ark:/87602/m4/M155297); other data same as LFI5. — UNIBAS LFI9, 1 (2D x-ray CT, MorphoSource http://n2t.net/ark:/87602/m4/M155298); other data same as LFI5. — UNIBAS LGA2, 1 (2D x-ray CT, MorphoSource http://n2t.net/ark:/87602/m4/M155300); other data same as LFI5. — UNIBAS LGA3, 1 (2D x-ray CT, MorphoSource http://n2t.net/ark:/87602/m4/M155301); other data same as LFI5.

Xenotilapia sima Boulenger, 1899: UNIBAS LBE2, 1 (2D x-ray CT, MorphoSource http://n2t.net/ark:/87602/m4/M156593); Tanzania: L. Tanganyika: Chipwa fishermen; F. Ronco, A. Indermaur, H. Büscher, & W. Salzburger, 2-Aug-2015. — UNIBAS LBE5, 1 (2D x-ray CT, MorphoSource http://n2t.net/ark:/87602/m4/M156596); other data same as LBE2. — UNIBAS LBE9, 1 (2D x-ray CT, MorphoSource http://n2t.net/ark:/87602/m4/M156598); other data same as LBE2. — UNIBAS LBF1, 1 (2D x-ray CT, MorphoSource http://n2t.net/ark:/87602/m4/M156599); other data same as LBE2. — UNIBAS LBF2, 1 (2D x-ray CT, MorphoSource http://n2t.net/ark:/87602/m4/M156600); other data same as LBE2.

Eretmodini

Eretmodus cyanostictus Boulenger, 1898: UNIBAS JDA5, 1 (2D x-ray CT, MorphoSource http://n2t.net/ark:/87602/m4/M154707); Zambia: Lake Tanganyika: Kalambo (Toby's) Lodge; F. Ronco, A. Indermaur, H. Büscher, & W. Salzburger, 1-Aug-2014. — UNIBAS JDA7, 1 (2D x-ray CT, MorphoSource http://n2t.net/ark:/87602/m4/M154709); other data same as JDA5. — UNIBAS JDA8, 1 (2D x-ray CT, MorphoSource http://n2t.net/ark:/87602/m4/M154710); other data same as JDA5. — UNIBAS JDA9, 1 (2D x-ray CT, MorphoSource http://n2t.net/ark:/87602/m4/M154711); other data same as JDA5. — UNIBAS JDB1, 1 (2D x-ray CT, MorphoSource http://n2t.net/ark:/87602/m4/M154712); other data same as JDA5.

Eretmodus marksmithi Burgess, 2012: UNIBAS JXF7, 1 (2D x-ray CT, MorphoSource http://n2t.net/ark:/87602/m4/M154718); Burundi: L. Tanganyika: Nyaruhongoka 2; F. Ronco, A.

Indermaur, H. Büscher, & W. Salzburger, 8-Jan-2015. — UNIBAS JXF9, 1 (2D x-ray CT, MorphoSource http://n2t.net/ark:/87602/m4/M154719); other data same as JXF7. — UNIBAS JXG1, 1 (2D x-ray CT, MorphoSource http://n2t.net/ark:/87602/m4/M154720); other data same as JXF7. — UNIBAS JXG3, 1 (2D x-ray CT, MorphoSource http://n2t.net/ark:/87602/m4/M154721); other data same as JXF7. — UNIBAS JXG4, 1 (2D x-ray CT, MorphoSource http://n2t.net/ark:/87602/m4/M154722); other data same as JXF7.

Spathodus erythrodon Boulenger, 1900: UNIBAS JUC5, 1 (2D x-ray CT, MorphoSource http://n2t.net/ark:/87602/m4/M154727); Burundi: L. Tanganyika: Kitaza south; F. Ronco, A. Indermaur, H. Büscher, & W. Salzburger, 5-Jan-2015. — UNIBAS JUC6, 1 (2D x-ray CT, MorphoSource http://n2t.net/ark:/87602/m4/M154728); other data same as JUC5. — UNIBAS JUC8, 1 (2D x-ray CT, MorphoSource http://n2t.net/ark:/87602/m4/M154730); other data same as JUC5. — UNIBAS JUC9, 1 (2D x-ray CT, MorphoSource http://n2t.net/ark:/87602/m4/M154731); other data same as JUC5. — JUD1, 1 (2D x-ray CT, MorphoSource http://n2t.net/ark:/87602/m4/M154732); other data same as JUC5.

Tanganicodus irsacae Poll, 1950: UNIBAS JYI1, 1 (2D x-ray CT, MorphoSource http://n2t.net/ark:/87602/m4/M154748); Burundi: L. Tanganyika: Nyanza Lac; F. Ronco, A. Indermaur, H. Büscher, & W. Salzburger, 11-Jan-2015. — UNIBAS JYI3, 1 (2D x-ray CT, MorphoSource http://n2t.net/ark:/87602/m4/M154749); other data same as JYI1. — UNIBAS JYI4, 1 (2D x-ray CT, MorphoSource http://n2t.net/ark:/87602/m4/M154750); other data same as JYI1. — UNIBAS JYI5, 1 (2D x-ray CT, MorphoSource http://n2t.net/ark:/87602/m4/M154751); other data same as JYI1. — UNIBAS JYI6, 1 (2D x-ray CT, MorphoSource http://n2t.net/ark:/87602/m4/M154752); other data same as JYI1.

Lamprologini

Altolamprologus calvus (Poll, 1978): UNIBAS IOB6, 1 (2D x-ray CT, MorphoSource http://n2t.net/ark:/87602/m4/M155435); no locality available; F. Ronco, A. Indermaur, H. Büscher, & W. Salzburger, 23-Aug-2014. — UNIBAS IOC3, 1 (2D x-ray CT, MorphoSource http://n2t.net/ark:/87602/m4/M155436); Zambia: L. Tanganyika: Kachese; F. Ronco, A. Indermaur, H. Büscher, & W. Salzburger, 23-Aug-2014. — UNIBAS IOC5, 1 (2D x-ray CT, MorphoSource http://n2t.net/ark:/87602/m4/M155438); other data same as IOC3. — UNIBAS IOE4, 1 (2D x-ray CT, MorphoSource http://n2t.net/ark:/87602/m4/M155439); Zambia: L. Tanganyika: Chimba; F. Ronco, A. Indermaur, H. Büscher, & W. Salzburger, 24-Aug-2014. — UNIBAS IOE5, 1 (2D x-ray CT, MorphoSource http://n2t.net/ark:/87602/m4/M155440); other data same as IOE4.

Altolamprologus compressiceps (Boulenger, 1898): UNIBAS IYC9, 1 (2D x-ray CT, MorphoSource http://n2t.net/ark:/87602/m4/M155518); Zambia: Lake Tanganyika: Kalambo (Toby's) Lodge; F. Ronco, A. Indermaur, H. Büscher, & W. Salzburger, 29-Jul-2014. — UNIBAS IYE7, 1 (2D x-ray CT, MorphoSource http://n2t.net/ark:/87602/m4/M155520); other data same as IYC9. — UNIBAS IYE8, 1 (2D x-ray CT, MorphoSource http://n2t.net/ark:/87602/m4/M155521); other data same as IYC9. — UNIBAS JYD1, 1 (2D x-ray CT, MorphoSource http://n2t.net/ark:/87602/m4/M155522); Burundi: L. Tanganyika: Nyaruhongoka 2; F. Ronco, A. Indermaur, H. Büscher, & W. Salzburger, 9-Jan-2015. — UNIBAS JYD2, 1

(2D x-ray CT, MorphoSource http://n2t.net/ark:/87602/m4/M155523); 7-Jan-2015, other data same as JYD1.

Altolamprologus sp. “compressiceps shell’: UNIBAS IRI2, 1 (2D x-ray CT, MorphoSource http://n2t.net/ark:/87602/m4/M156626); Zambia: L. Tanganyika: Chibwensolo; F. Ronco, A. Indermaur, H. Büscher, & W. Salzburger, 25-Aug-2014. — UNIBAS IRI3, 1 (2D x-ray CT, MorphoSource http://n2t.net/ark:/87602/m4/M156627); other data same as IRI2. — UNIBAS IRI5, 1 (2D x-ray CT, MorphoSource http://n2t.net/ark:/87602/m4/M156628); other data same as IRI2. — UNIBAS IRI6, 1 (2D x-ray CT, MorphoSource http://n2t.net/ark:/87602/m4/M156629); other data same as IRI2. — UNIBAS IRI7, 1 (2D x-ray CT, MorphoSource http://n2t.net/ark:/87602/m4/M156630); other data same as IRI2.

Chalinochromis brichardi, Poll 1974: UNIBAS IQF6, 1 (2D x-ray CT, MorphoSource http://n2t.net/ark:/87602/m4/M155346); Zambia: Lake Tanganyika: Kalambo (Toby’s) Lodge; F. Ronco, A. Indermaur, H. Büscher, & W. Salzburger, 22-Jul-2014. — UNIBAS IQF7, 1 (2D x-ray CT, MorphoSource http://n2t.net/ark:/87602/m4/M155347); other data same as IQF6. — UNIBAS IYC5, 1 (2D x-ray CT, MorphoSource http://n2t.net/ark:/87602/m4/M155348); 29-Jul-2014, other data same as IQF6. — UNIBAS IYC6, 1 (2D x-ray CT, MorphoSource http://n2t.net/ark:/87602/m4/M155349); other data same as IYC5. — UNIBAS IYD7, 1 (2D x-ray CT, MorphoSource http://n2t.net/ark:/87602/m4/M155350); other data same as IYC5.

Chalinochromis cyanophleps Kullander, Karlsson, Karlsson, & Norén, 2014: UNIBAS LGC8, 1 (2D x-ray CT, MorphoSource http://n2t.net/ark:/87602/m4/M155554); Tanzania: L. Tanganyika: Mvuna Island; F. Ronco, A. Indermaur, H. Büscher, & W. Salzburger, 18-Aug-2015. — UNIBAS LGG6, 1 (2D x-ray CT, MorphoSource http://n2t.net/ark:/87602/m4/M155556); Tanzania: L. Tanganyika: Twiyu; F. Ronco, A. Indermaur, H. Büscher, & W. Salzburger, 19-Aug-2015. — UNIBAS LGG7, 1 (2D x-ray CT, MorphoSource http://n2t.net/ark:/87602/m4/M155557); other data same as LGG6. — UNIBAS LGG8, 1 (2D x-ray CT, MorphoSource http://n2t.net/ark:/87602/m4/M155558); other data same as LGG6. — UNIBAS LGG9, 1 (2D x-ray CT, MorphoSource http://n2t.net/ark:/87602/m4/M155559); other data same as LGG6.

Julidochromis ornatus, Boulenger 1898: UNIBAS JCF9, 1 (2D x-ray CT, MorphoSource http://n2t.net/ark:/87602/m4/M156364); Zambia: Lake Tanganyika: Kalambo (Toby’s) Lodge; F. Ronco, A. Indermaur, H. Büscher, & W. Salzburger, 1-Aug-2014. — UNIBAS JCG1, 1 (2D x-ray CT, MorphoSource http://n2t.net/ark:/87602/m4/M156365); other data same as JCF9. — UNIBAS JCI2, 1 (2D x-ray CT, MorphoSource http://n2t.net/ark:/87602/m4/M156366); other data same as JCF9. — UNIBAS JCI3, 1 (2D x-ray CT, MorphoSource http://n2t.net/ark:/87602/m4/M156367); other data same as JCF9. — UNIBAS JDD6, 1 (2D x-ray CT, MorphoSource http://n2t.net/ark:/87602/m4/M156368); other data same as JCF9.

Julidochromis regani, Poll 1942: UNIBAS KHF1, 1 (2D x-ray CT, MorphoSource http://n2t.net/ark:/87602/m4/M156536); Tanzania: L. Tanganyika: Nondwa Point; F. Ronco, A. Indermaur, H. Büscher, & W. Salzburger, 23-Jun-2015. — UNIBAS KHF2, 1 (2D x-ray CT, MorphoSource http://n2t.net/ark:/87602/m4/M156537); other data same as KHF1. — UNIBAS KHF3, 1 (2D x-ray CT, MorphoSource http://n2t.net/ark:/87602/m4/M156538); other data same as KHF1. — UNIBAS KHI3, 1

(2D x-ray CT, MorphoSource http://n2t.net/ark:/87602/m4/M156539); Zambia: L. Tanganyika: Kaku; F. Ronco, A. Indermaur, H. Büscher, & W. Salzburger, 24-Jun-2015. — UNIBAS KHI4, 1 (2D x-ray CT, MorphoSource http://n2t.net/ark:/87602/m4/M156540); other data same as KHI3.

Lamprologus callipterus, Boulenger 1906: UNIBAS IMF4, 1 (2D x-ray CT, MorphoSource http://n2t.net/ark:/87602/m4/M155401); Zambia: L. Tanganyika: Kabwensolo; F. Ronco, A. Indermaur, H. Büscher, & W. Salzburger, 19-Aug-2014. — UNIBAS JAB9, 1 (2D x-ray CT, MorphoSource http://n2t.net/ark:/87602/m4/M155402); Zambia: Lake Tanganyika: Kalambo (Toby's) Lodge; F. Ronco, A. Indermaur, H. Büscher, & W. Salzburger, 30-Jul-2014. — UNIBAS JEA4, 1 (2D x-ray CT, MorphoSource http://n2t.net/ark:/87602/m4/M155403); Zambia: Lake Tanganyika: Chitweshiba; F. Ronco, A. Indermaur, H. Büscher, & W. Salzburger, 20-Aug-2014. — UNIBAS JEA5, 1 (2D x-ray CT, MorphoSource http://n2t.net/ark:/87602/m4/M155404); other data same as JEA4. — UNIBAS JEA6, 1 (2D x-ray CT, MorphoSource http://n2t.net/ark:/87602/m4/M155405); other data same as JEA4. — UNIBAS JEA7, 1 (2D x-ray CT, MorphoSource http://n2t.net/ark:/87602/m4/M155406); other data same as JEA4. — UNIBAS JEE3, 1 (2D x-ray CT, MorphoSource http://n2t.net/ark:/87602/m4/M155407); Zambia: L. Tanganyika: Mibwebwe; F. Ronco, A. Indermaur, H. Büscher, & W. Salzburger, 21-Aug-2014. — UNIBAS JEE4, 1 (2D x-ray CT, MorphoSource http://n2t.net/ark:/87602/m4/M155408); other data same as JEE3. — UNIBAS JEE5, 1 (2D x-ray CT, MorphoSource http://n2t.net/ark:/87602/m4/M155409); other data same as JEE3. — UNIBAS JEE6, 1 (2D x-ray CT, MorphoSource http://n2t.net/ark:/87602/m4/M155410); other data same as JEE3.

Lamprologus sp. 'ornatipinnis congo': UNIBAS 93-14, 1 (2D x-ray CT, MorphoSource http://n2t.net/ark:/87602/m4/M156731); DRC: L. Tanganyika: Kisongwa; collector(s) not available, 29-Apr-1993. — UNIBAS 93-18-a, 1 (2D x-ray CT, MorphoSource http://n2t.net/ark:/87602/m4/M156732); 2-May-1993, other data same as 93-14. — UNIBAS 93-18-b, 1 (2D x-ray CT, MorphoSource http://n2t.net/ark:/87602/m4/M156733); other data same as 93-18-a. — UNIBAS 94-51, 1 (2D x-ray CT, MorphoSource http://n2t.net/ark:/87602/m4/M156734); 2-Jun-1994, other data same as 93-14. — UNIBAS 94-60, 1 (2D x-ray CT, MorphoSource http://n2t.net/ark:/87602/m4/M156735); other data same as 94-51.

Lamprologus sp. 'ornatipinnis zambia': UNIBAS ILD7, 1 (2D x-ray CT, MorphoSource http://n2t.net/ark:/87602/m4/M156736); Zambia: L. Tanganyika: Kombe; F. Ronco, A. Indermaur, H. Büscher, & W. Salzburger, 15-Aug-2014. — UNIBAS IVE5, 1 (2D x-ray CT, MorphoSource http://n2t.net/ark:/87602/m4/M156737); L. Tanganyika: Ntingila; F. Ronco, A. Indermaur, H. Büscher, & W. Salzburger, 25-Aug-2014. — UNIBAS IVE6, 1 (2D x-ray CT, MorphoSource http://n2t.net/ark:/87602/m4/M156738); other data same as IVE5. — UNIBAS IVE7, 1 (2D x-ray CT, MorphoSource http://n2t.net/ark:/87602/m4/M156739); other data same as IVE5. — UNIBAS JDH4, 1 (2D x-ray CT, MorphoSource http://n2t.net/ark:/87602/m4/M156740); Zambia: L. Tanganyika: Katoto; F. Ronco, A. Indermaur, H. Büscher, & W. Salzburger, 14-Aug-2014. — UNIBAS JDH5, 1 (2D x-ray CT, MorphoSource http://n2t.net/ark:/87602/m4/M156741); other data same as JDH4. — UNIBAS JDH6, 1 (2D x-ray CT, MorphoSource http://n2t.net/ark:/87602/m4/M156742); other data same as JDH4. — UNIBAS JEE2, 1 (2D x-ray CT, MorphoSource http://n2t.net/ark:/87602/m4/M156743); Zambia: L. Tanganyika: Kabyolwe; F. Ronco, A. Indermaur, H. Büscher, & W. Salzburger, 21-Aug-2014. — UNIBAS

JEH6, 1 (2D x-ray CT, MorphoSource http://n2t.net/ark:/87602/m4/M156744); 22-Aug-2014, other data same as JEE2.

Lamprologus speciosus, Büscher 1991: UNIBAS KCG9, 1 (2D x-ray CT, MorphoSource http://n2t.net/ark:/87602/m4/M157056); Burundi: L. Tanganyika: Nyanza Lac; F. Ronco, A. Indermaur, H. Büscher, & W. Salzburger, 16-Jan-2015. — UNIBAS KCH1, 1 (2D x-ray CT, MorphoSource http://n2t.net/ark:/87602/m4/M157057); other data same as KCH9. — UNIBAS KCH2, 1 (2D x-ray CT, MorphoSource http://n2t.net/ark:/87602/m4/M157058); other data same as KCH9. — UNIBAS KHI5, 1 (2D x-ray CT, MorphoSource http://n2t.net/ark:/87602/m4/M157059); Zambia: L. Tanganyika: Kaku; F. Ronco, A. Indermaur, H. Büscher, & W. Salzburger, 24-Jun-2015. — UNIBAS KHI6, 1 (2D x-ray CT, MorphoSource http://n2t.net/ark:/87602/m4/M157060); other data same as KHI5.

Lepidiolamprologus kendalli (Poll & Stewart, 1977): UNIBAS IOC1, 1 (2D x-ray CT, MorphoSource http://n2t.net/ark:/87602/m4/M155858); Zambia: L. Tanganyika: Kachese; F. Ronco, A. Indermaur, H. Büscher, & W. Salzburger, 23-Aug-2014. — UNIBAS JBB6, 1 (2D x-ray CT, MorphoSource http://n2t.net/ark:/87602/m4/M155859); L. Tanganyika: Misepa; F. Ronco, A. Indermaur, H. Büscher, & W. Salzburger, 30-Aug-2014. — UNIBAS JBB7, 1 (2D x-ray CT, MorphoSource http://n2t.net/ark:/87602/m4/M155860); other data same as JBB6. — UNIBAS JBB8, 1 (2D x-ray CT, MorphoSource http://n2t.net/ark:/87602/m4/M155861); other data same as JBB6. — UNIBAS JEE1, 1 (2D x-ray CT, MorphoSource http://n2t.net/ark:/87602/m4/M155862); Zambia: L. Tanganyika: Mibwebwe; F. Ronco, A. Indermaur, H. Büscher, & W. Salzburger, 21-Aug-2014.

Lepidiolamprologus mimicus Schelly, Takahashi, Bills, & Hori, 2007: UNIBAS LDD5, 1 (2D x-ray CT, MorphoSource http://n2t.net/ark:/87602/m4/M156155); Tanzania: L. Tanganyika: Fulwe; F. Ronco, A. Indermaur, H. Büscher, & W. Salzburger, 12-Aug-2015. — UNIBAS LDD6, 1 (2D x-ray CT, MorphoSource http://n2t.net/ark:/87602/m4/M156156); other data same as LDD5. — UNIBAS LDH3, 1 (2D x-ray CT, MorphoSource http://n2t.net/ark:/87602/m4/M156157); Tanzania: L. Tanganyika: Twiyu; F. Ronco, A. Indermaur, H. Büscher, & W. Salzburger, 13-Aug-2015. — UNIBAS LGD5, 1 (2D x-ray CT, MorphoSource http://n2t.net/ark:/87602/m4/M156158); Tanzania: L. Tanganyika: Mvuna Island; F. Ronco, A. Indermaur, H. Büscher, & W. Salzburger, 18-Aug-2015. — UNIBAS LGG4, 1 (2D x-ray CT, MorphoSource http://n2t.net/ark:/87602/m4/M156159); 19-Aug-2015, other data same as LDH3.

Lepidiolamprologus profundicola (Poll, 1949): UNIBAS JED3, 1 (2D x-ray CT, MorphoSource http://n2t.net/ark:/87602/m4/M156516); Zambia: Lake Tanganyika: Chitweshiba; F. Ronco, A. Indermaur, H. Büscher, & W. Salzburger, 20-Aug-2014. — UNIBAS KAD2, 1 (2D x-ray CT, MorphoSource http://n2t.net/ark:/87602/m4/M156517); Burundi: L. Tanganyika: Nyaruhongoka 2; F. Ronco, A. Indermaur, H. Büscher, & W. Salzburger, 12-Jan-2015. — UNIBAS KEB8, 1 (2D x-ray CT, MorphoSource http://n2t.net/ark:/87602/m4/M156518); Tanzania: L. Tanganyika: Cape Kabogo; F. Ronco, A. Indermaur, H. Büscher, & W. Salzburger, 25-Jun-2015. — UNIBAS KEB9, 1 (2D x-ray CT, MorphoSource http://n2t.net/ark:/87602/m4/M156519); other data same as KEB8. — UNIBAS LGD1, 1 (2D x-ray CT, MorphoSource http://n2t.net/ark:/87602/m4/M156520); Tanzania: L. Tanganyika: Mvuna Island; F. Ronco, A. Indermaur, H. Büscher, & W. Salzburger, 18-Aug-2015.

Neolamprologus tretocephalus (Boulenger, 1899): UNIBAS KFI8, 1 (2D x-ray CT, MorphoSource http://n2t.net/ark:/87602/m4/M157180); Zambia: L. Tanganyika: Kaku; F. Ronco, A. Indermaur, H. Büscher, & W. Salzburger, 21-Jun-2015. — UNIBAS KFI4, 1 (2D x-ray CT, MorphoSource http://n2t.net/ark:/87602/m4/M157176); other data same as KFI8. — UNIBAS KFI5, 1 (2D x-ray CT, MorphoSource http://n2t.net/ark:/87602/m4/M157177); other data same as KFI8. — UNIBAS KFI6, 1 (2D x-ray CT, MorphoSource http://n2t.net/ark:/87602/m4/M157178); other data same as KFI8. — UNIBAS KFI7, 1 (2D x-ray CT, MorphoSource http://n2t.net/ark:/87602/m4/M157179); other data same as KFI8.

Neolamprologus ventralis Büscher, 1995: UNIBAS KAD4, 1 (2D x-ray CT, MorphoSource http://n2t.net/ark:/87602/m4/M157210); Burundi: L. Tanganyika: Nyaruhongoka 2; F. Ronco, A. Indermaur, H. Büscher, & W. Salzburger, 14-Jan-2015. — UNIBAS KAG9, 1 (2D x-ray CT, MorphoSource http://n2t.net/ark:/87602/m4/M157212); 15-Jan-2015, other data same as KAD4. — UNIBAS KDF3, 1 (2D x-ray CT, MorphoSource http://n2t.net/ark:/87602/m4/M157213); 22-Jan-2015, other data same as KAD4. — UNIBAS KDF4, 1 (2D x-ray CT, MorphoSource http://n2t.net/ark:/87602/m4/M157214); other data same as KDF3. — UNIBAS KDF5, 1 (2D x-ray CT, MorphoSource http://n2t.net/ark:/87602/m4/M157215); other data same as KDF3.

Telmatochromis dhonti (Boulenger, 1919): UNIBAS IZG9, 1 (2D x-ray CT, MorphoSource http://n2t.net/ark:/87602/m4/M155600); "Kalambo Lake / Chipwa"; F. Ronco, A. Indermaur, H. Büscher, & W. Salzburger, 28-Jul-2014. — UNIBAS IZH3, 1 (2D x-ray CT, MorphoSource http://n2t.net/ark:/87602/m4/M155603); Tanzania: L. Tanganyika: Chipwa fishermen; F. Ronco, A. Indermaur, H. Büscher, & W. Salzburger, 28-Jul-2014. — UNIBAS IZH4, 1 (2D x-ray CT, MorphoSource http://n2t.net/ark:/87602/m4/M155604); other data same as IZH3. — UNIBAS IZH5, 1 (2D x-ray CT, MorphoSource http://n2t.net/ark:/87602/m4/M155605); other data same as IZG9. — UNIBAS IZH6, 1 (2D x-ray CT, MorphoSource http://n2t.net/ark:/87602/m4/M155606); other data same as IZG9.

Telmatochromis temporalis Boulenger, 1898: UNIBAS IND4, 1 (2D x-ray CT, MorphoSource http://n2t.net/ark:/87602/m4/M157124); L. Tanganyika: Chezi; F. Ronco, A. Indermaur, H. Büscher, & W. Salzburger, 16-Aug-2014. — UNIBAS IND5, 1 (2D x-ray CT, MorphoSource http://n2t.net/ark:/87602/m4/M157125); other data same as IND4. — UNIBAS IQA7, 1 (2D x-ray CT, MorphoSource http://n2t.net/ark:/87602/m4/M157128); Zambia: Lake Tanganyika: Kalambo (Toby's) Lodge; F. Ronco, A. Indermaur, H. Büscher, & W. Salzburger, 21-Jul-2014. — UNIBAS IYH6, 1 (2D x-ray CT, MorphoSource http://n2t.net/ark:/87602/m4/M157129); 29-Jul-2014, other data same as IQA7. — UNIBAS JAD5, 1 (2D x-ray CT, MorphoSource http://n2t.net/ark:/87602/m4/M157130); 30-Jul-2014, other data same as IQA7.

Telmatochromis vittatus Boulenger, 1898: UNIBAS IRC1, 1 (2D x-ray CT, MorphoSource http://n2t.net/ark:/87602/m4/M157228); Zambia: L. Tanganyika: Chimba; F. Ronco, A. Indermaur, H. Büscher, & W. Salzburger, 24-Aug-2014. — UNIBAS JBD7, 1 (2D x-ray CT, MorphoSource http://n2t.net/ark:/87602/m4/M157230); Zambia: Lake Tanganyika: Chitweshiba; F. Ronco, A. Indermaur, H. Büscher, & W. Salzburger, 31-Aug-2014. — UNIBAS LPA4, 1 (2D x-ray CT, MorphoSource http://n2t.net/ark:/87602/m4/M157236); Zambia: Lake Tanganyika: Kalambo (Toby's) Lodge; F. Ronco,

A. Indermaur, H. Büscher, & W. Salzburger, 23-Aug-2014. — BMNH 1898.9.9.19–20, 2 (syntypes; 2D radiograph available at https://data.nhm.ac.uk/object/792e3e6b-76d0-4ed1-aaf7-c32ee5afa529); Tanzania: L. Tanganyika: Mbity Rocks; J.E. Moore.

Variabilichromis moorii (Boulenger, 1898): UNIBAS IYC2, 1 (2D x-ray CT, MorphoSource http://n2t.net/ark:/87602/m4/M156203); Zambia: Lake Tanganyika: Kalambo (Toby's) Lodge; F. Ronco, A. Indermaur, H. Büscher, & W. Salzburger, 29-Jul-2014. — UNIBAS IYC3, 1 (2D x-ray CT, MorphoSource http://n2t.net/ark:/87602/m4/M156204); other data same as IYC2. — UNIBAS IYC4, 1 (2D x-ray CT, MorphoSource http://n2t.net/ark:/87602/m4/M156205); other data same as IYC2. — UNIBAS IYC7, 1 (2D x-ray CT, MorphoSource http://n2t.net/ark:/87602/m4/M156206); other data same as IYC2. — UNIBAS IYC8, 1 (2D x-ray CT, MorphoSource http://n2t.net/ark:/87602/m4/M156207); other data same as IYC2. — UNIBAS IYH8, 1 (2D x-ray CT, MorphoSource http://n2t.net/ark:/87602/m4/M156208); other data same as IYC2. — UNIBAS LNF5, 1 (2D x-ray CT, MorphoSource http://n2t.net/ark:/87602/m4/M156209); 22-Aug-2016, other data same as IYC2.

Limnochromini

Baileychromis centropomoides (Bailey & Stewart, 1977): UNIBAS JCC6, 1 (2D x-ray CT, MorphoSource http://n2t.net/ark:/87602/m4/M155468); Tanzania: L. Tanganyika: Chipwa fishermen; F. Ronco, A. Indermaur, H. Büscher, & W. Salzburger, 31-Jul-2014. — UNIBAS JCC9, 1 (2D x-ray CT, MorphoSource http://n2t.net/ark:/87602/m4/M155470); other data same as JCC6. — UNIBAS JCD1, 1 (2D x-ray CT, MorphoSource http://n2t.net/ark:/87602/m4/M155471); other data same as JCC6. — UNIBAS LBF4, 1 (2D x-ray CT, MorphoSource http://n2t.net/ark:/87602/m4/M155472); 4-Aug-2015, other data same as JCC6. — UNIBAS LBF5, 1 (2D x-ray CT, MorphoSource http://n2t.net/ark:/87602/m4/M155473); other data same as LBF4.

Gnathochromis permaxillaris (David, 1936): UNIBAS IXD5, 1 (2D x-ray CT, MorphoSource http://n2t.net/ark:/87602/m4/M156426; Tanzania: L. Tanganyika: Chipwa fishermen; F. Ronco, A. Indermaur, H. Büscher, & W. Salzburger, 27-Jul-2014. — UNIBAS IXD6, 1 (2D x-ray CT, MorphoSource http://n2t.net/ark:/87602/m4/M156427); other data same as IXD5. — UNIBAS IXD7, 1 (2D x-ray CT, MorphoSource http://n2t.net/ark:/87602/m4/M156428); other data same as IXD5. — UNIBAS IXD8, 1 (2D x-ray CT, MorphoSource http://n2t.net/ark:/87602/m4/M156429); other data same as IXD5. — UNIBAS IXD9, 1 (2D x-ray CT, MorphoSource http://n2t.net/ark:/87602/m4/M156430); other data same as IXD5.

Greenwoodochromis christyi (Trewavas, 1953): UNIBAS JCB6, 1 (2D x-ray CT, MorphoSource http://n2t.net/ark:/87602/m4/M155489); Tanzania: L. Tanganyika: Chipwa fishermen; F. Ronco, A. Indermaur, H. Büscher, & W. Salzburger, 31-Jul-2014. — UNIBAS JCE4, 1 (2D x-ray CT, MorphoSource http://n2t.net/ark:/87602/m4/M155490); other data same as JCB6. — UNIBAS JCE6, 1 (2D x-ray CT, MorphoSource http://n2t.net/ark:/87602/m4/M155491); other data same as JCB6. — UNIBAS JCE7, 1 (2D x-ray CT, MorphoSource http://n2t.net/ark:/87602/m4/M155492); other data same as JCB6. — UNIBAS JCE8, 1 (2D x-ray CT, MorphoSource http://n2t.net/ark:/87602/m4/M155493); other data same as JCB6.

Limnochromis auritus (Boulenger, 1901): MCZ 50835, 1 (3D CT image series, MorphoSource http://n2t.net/ark:/87602/m4/355512); Burundi: L. Tanganyika: Bujumbura; D.J. Stewart, Oct-1973. — UNIBAS JCA1, 1 (2D x-ray CT, MorphoSource http://n2t.net/ark:/87602/m4/M155223); Tanzania: L. Tanganyika: Chipwa fishermen; F. Ronco, A. Indermaur, H. Büscher, & W. Salzburger, 31-Jul-2014. — UNIBAS JCD9, 1 (2D x-ray CT, MorphoSource http://n2t.net/ark:/87602/m4/M155224); other data same as JCA1. — UNIBAS JCE1, 1 (2D x-ray CT, MorphoSource http://n2t.net/ark:/87602/m4/M155225); other data same as JCA1. — UNIBAS JEH2, 1 (2D x-ray CT, MorphoSource http://n2t.net/ark:/87602/m4/M155226); Zambia: L. Tanganyika: Kabyolwe; F. Ronco, A. Indermaur, H. Büscher, & W. Salzburger, 22-Aug-2014.

Reganochromis calliurus (Boulenger, 1901): UNIBAS IYB4, 1 (2D x-ray CT, MorphoSource http://n2t.net/ark:/87602/m4/M155424); Tanzania: L. Tanganyika: Chipwa fishermen; F. Ronco, A. Indermaur, H. Büscher, & W. Salzburger, 29-Jul-2014. — UNIBAS IZB5, 1 (2D x-ray CT, MorphoSource http://n2t.net/ark:/87602/m4/M155425); 28-Jul-2014, other data same as IYB4. — UNIBAS JCD3, 1 (2D x-ray CT, MorphoSource http://n2t.net/ark:/87602/m4/M155428); 31-Jul-2014, other data same as IYB4. — UNIBAS JCD4, 1 (2D x-ray CT, MorphoSource http://n2t.net/ark:/87602/m4/M155429); other data same as JCD3. — UNIBAS JCD5, 1 (2D x-ray CT, MorphoSource http://n2t.net/ark:/87602/m4/M155430); other data same as JCD3.

Tangachromis dhanisi (Poll, 1949): MCZ 50836, 1 (3D CT image series, MorphoSource http://n2t.net/ark:/87602/m4/M55557); Burundi: L. Tanganyika: Bujumbura; D.J. Stewart, Oct-1973. — UNIBAS LJA7, 1 (2D x-ray CT, MorphoSource http://n2t.net/ark:/87602/m4/M155594); Zambia: L. Tanganyika: Chituta; F. Ronco, A. Indermaur, H. Büscher, & W. Salzburger, 6-Nov-2015. — UNIBAS LJA8, 1 (2D x-ray CT, MorphoSource http://n2t.net/ark:/87602/m4/M155595); other data same as LJA7. — UNIBAS LJA9, 1 (2D x-ray CT, MorphoSource http://n2t.net/ark:/87602/m4/M155596); other data same as LJA7.

Trematochromis benthicola (Matthes, 1962): UNIBAS DMC6, 1 (2D x-ray CT, MorphoSource http://n2t.net/ark:/87602/m4/M155268); Zambia: L. Tanganyika: Mpulungu fishmarket; F. Ronco, A. Indermaur, H. Büscher, & W. Salzburger, 16-Sep-2011. — UNIBAS DMC7, 1 (2D x-ray CT, MorphoSource http://n2t.net/ark:/87602/m4/M155269); other data same as DMC6. — UNIBAS DMC8, 1 (2D x-ray CT, MorphoSource http://n2t.net/ark:/87602/m4/M155270); 11-Sep-2011, other data same as DMC6. — UNIBAS DMC9, 1 (2D x-ray CT, MorphoSource http://n2t.net/ark:/87602/m4/M155271); 16-Sep-2011, other data same as DMC6. — UNIBAS DMD2, 1 (2D x-ray CT, MorphoSource http://n2t.net/ark:/87602/m4/M155272); other data same as DMC9.

Triglachromis otostigma (Regan, 1920): UNIBAS JBB3, 1 (2D x-ray CT, MorphoSource http://n2t.net/ark:/87602/m4/M156383); Zambia: L. Tanganyika: Kabyolwe; F. Ronco, A. Indermaur, H. Büscher, & W. Salzburger, 30-Aug-2014. — UNIBAS JEG6, 1 (2D x-ray CT, MorphoSource http://n2t.net/ark:/87602/m4/M156385); 22-Aug-2014, other data same as JBB3. — UNIBAS JEG7, 1 (2D x-ray CT, MorphoSource http://n2t.net/ark:/87602/m4/M156386); other data same as JEG6. — UNIBAS KAI9, 1 (2D x-ray CT, MorphoSource http://n2t.net/ark:/87602/m4/M156387); Burundi: L. Tanganyika:

Bujumbura fishmarket; F. Ronco, A. Indermaur, H. Büscher, & W. Salzburger, 15-Jan-2015. — UNIBAS KCA1, 1 (2D x-ray CT, MorphoSource http://n2t.net/ark:/87602/m4/M156388); other data same as KAI9.

Oreochromini

Oreochromis tanganicae (Günther, 1894): UNIBAS LGD7, 1 (2D x-ray CT, MorphoSource http://n2t.net/ark:/87602/m4/M157116); Tanzania: L. Tanganyika: Mvuna Island; F. Ronco, A. Indermaur, H. Büscher, & W. Salzburger, 18-Aug-2015. — UNIBAS LGD8, 1 (2D x-ray CT, MorphoSource http://n2t.net/ark:/87602/m4/M157117); other data same as LGD8. — UNIBAS LGD9, 1 (2D x-ray CT, MorphoSource http://n2t.net/ark:/87602/m4/M157118); other data same as LGD8. — UNIBAS LIB3, 1 (2D x-ray CT, MorphoSource http://n2t.net/ark:/87602/m4/M157119); Zambia: L. Tanganyika: Mpulungu fishmarket; F. Ronco, A. Indermaur, H. Büscher, & W. Salzburger, 26-Aug-2015. — UNIBAS LIB4, 1 (2D x-ray CT, MorphoSource http://n2t.net/ark:/87602/m4/M157120); other data same as LIB3.

Perissodini

Haplotaxodon microlepis Boulenger, 1906: UNIBAS ITG5, 1 (2D x-ray CT, MorphoSource http://n2t.net/ark:/87602/m4/M156136); Tanzania: L. Tanganyika: Chipwa fishermen; F. Ronco, A. Indermaur, H. Büscher, & W. Salzburger, 24-Jul-2014. — UNIBAS ITG6, 1 (2D x-ray CT, MorphoSource http://n2t.net/ark:/87602/m4/M156137); other data same as ITG5. — UNIBAS JEA3, 1 (2D x-ray CT, MorphoSource http://n2t.net/ark:/87602/m4/M156138); Zambia: Lake Tanganyika: Chitweshiba; F. Ronco, A. Indermaur, H. Büscher, & W. Salzburger, 20-Aug-2014. — UNIBAS JED7, 1 (2D x-ray CT, MorphoSource http://n2t.net/ark:/87602/m4/M156139); Zambia: L. Tanganyika: Mibwebwe; F. Ronco, A. Indermaur, H. Büscher, & W. Salzburger, 21-Aug-2014. — UNIBAS JED8, 1 (2D x-ray CT, MorphoSource http://n2t.net/ark:/87602/m4/M156140); other data same as JED7.

Perissodus microlepis Boulenger, 1898: UNIBAS IPE9, 1 (2D x-ray CT, MorphoSource http://n2t.net/ark:/87602/m4/M156145); Zambia: Lake Tanganyika: Kalambo (Toby's) Lodge; F. Ronco, A. Indermaur, H. Büscher, & W. Salzburger, 21-Jul-2014. — UNIBAS IPG1, 1 (2D x-ray CT, MorphoSource http://n2t.net/ark:/87602/m4/M156146); other data same as IPE9. — UNIBAS JAD7, 1 (2D x-ray CT, MorphoSource http://n2t.net/ark:/87602/m4/M156148); 30-Jul-2014, other data same as IPE9. — UNIBAS JAD8, 1 (2D x-ray CT, MorphoSource http://n2t.net/ark:/87602/m4/M156149); other data same as JAD7. — UNIBAS KAG6, 1 (2D x-ray CT, MorphoSource http://n2t.net/ark:/87602/m4/M156150); Burundi: L. Tanganyika: Nyaruhongoka 2; F. Ronco, A. Indermaur, H. Büscher, & W. Salzburger, 15-Jan-2015.

Plecodus paradoxus (Boulenger, 1898): UNIBAS KEH9, 1 (2D x-ray CT, MorphoSource http://n2t.net/ark:/87602/m4/M156404); Tanzania: L. Tanganyika: Bulu Point; F. Ronco, A. Indermaur, H. Büscher, & W. Salzburger, 27-Jun-2015. — UNIBAS KEI1, 1 (2D x-ray CT, MorphoSource http://n2t.net/ark:/87602/m4/M156405); other data same as KEH9. — UNIBAS LEA1, 1 (2D x-ray CT, MorphoSource http://n2t.net/ark:/87602/m4/M156407); Tanzania: L. Tanganyika: Karilani Island; F. Ronco, A. Indermaur, H. Büscher, & W. Salzburger, 27-Jun-2015. — UNIBAS LEA2, 1 (2D x-ray CT, MorphoSource http://n2t.net/ark:/87602/m4/M156408); other data same as LEA1. — UNIBAS LEC6, 1 (2D x-ray CT, MorphoSource http://n2t.net/ark:/87602/m4/M156409); other data same as LEA1.

Xenochromis hecqui Boulenger, 1899: UNIBAS IZC1, 1 (2D x-ray CT, MorphoSource http://n2t.net/ark:/87602/m4/M155801); Tanzania: L. Tanganyika: Chipwa fishermen; F. Ronco, A. Indermaur, H. Büscher, & W. Salzburger, 28-Jul-2014. — UNIBAS IZC2, 1 (2D x-ray CT, MorphoSource http://n2t.net/ark:/87602/m4/M155802); other data same as IZC1. — UNIBAS JAC8, 1 (2D x-ray CT, MorphoSource http://n2t.net/ark:/87602/m4/M155803); 30-Jul-2014, other data same as IZC1. — UNIBAS LBC3, 1 (2D x-ray CT, MorphoSource http://n2t.net/ark:/87602/m4/M155804); 1-Aug-2015, other data same as IZC1. — UNIBAS LBD4, 1 (2D x-ray CT, MorphoSource http://n2t.net/ark:/87602/m4/M155805); 2-Aug-2015, other data same as IZC1.

Pseudocrenilabrini: Tropheina

Interochromis loocki (Poll, 1949): UNIBAS IYE5, 1 (2D x-ray CT, MorphoSource http://n2t.net/ark:/87602/m4/M155981); Zambia: Lake Tanganyika: Kalambo (Toby's) Lodge; F. Ronco, A. Indermaur, H. Büscher, & W. Salzburger, 29-Jul-2014. — UNIBAS IYF7, 1 (2D x-ray CT, MorphoSource http://n2t.net/ark:/87602/m4/M155982); other data same as IYE5. — UNIBAS IYF8, 1 (2D x-ray CT, MorphoSource http://n2t.net/ark:/87602/m4/M155983); other data same as IYE5. — UNIBAS IYG8, 1 (2D x-ray CT, MorphoSource http://n2t.net/ark:/87602/m4/M155984); other data same as IYE5. — UNIBAS IYG9, 1 (2D x-ray CT, MorphoSource http://n2t.net/ark:/87602/m4/M155985); other data same as IYE5.

Jabarichromis pfefferi (Boulenger, 1898): UNIBAS ISH6, 1 (2D x-ray CT, MorphoSource http://n2t.net/ark:/87602/m4/M156461); Zambia: Lake Tanganyika: Kalambo (Toby's) Lodge; F. Ronco, A. Indermaur, H. Büscher, & W. Salzburger, 23-Jul-2014. — UNIBAS IWC7, 1 (2D x-ray CT, MorphoSource http://n2t.net/ark:/87602/m4/M156462); Zambia: L. Tanganyika: Katete 3; F. Ronco, A. Indermaur, H. Büscher, & W. Salzburger, 26-Aug-2014. — UNIBAS IWG8, 1 (2D x-ray CT, MorphoSource http://n2t.net/ark:/87602/m4/M156463); Zambia: L. Tanganyika: Chimba village; F. Ronco, A. Indermaur, H. Büscher, & W. Salzburger, 28-Aug-2014. — UNIBAS IWG9, 1 (2D x-ray CT, MorphoSource http://n2t.net/ark:/87602/m4/M156464); other data same as IWG8. — UNIBAS IWH1, 1 (2D x-ray CT, MorphoSource http://n2t.net/ark:/87602/m4/M156465); other data same as IWG8.

Limnotilapia dardennii (Boulenger, 1899): UNIBAS GPB6, 1 (2D x-ray CT, MorphoSource http://n2t.net/ark:/87602/m4/M155570); Zambia: L. Tanganyika: Mpulungu fishmarket; F. Ronco, A. Indermaur, H. Büscher, & W. Salzburger, 2-Sep-2014. — UNIBAS GPF5, 1 (2D x-ray CT, MorphoSource http://n2t.net/ark:/87602/m4/M155571); Zambia: L. Tanganyika: Chituta; F. Ronco, A. Indermaur, H. Büscher, & W. Salzburger, 3-Sep-2014. — UNIBAS IMD6, 1 (2D x-ray CT, MorphoSource http://n2t.net/ark:/87602/m4/M155572); Zambia: L. Tanganyika: Kabwensolo; F. Ronco, A. Indermaur, H. Büscher, & W. Salzburger, 19-Aug-2014. — UNIBAS JBF6, 1 (2D x-ray CT, MorphoSource http://n2t.net/ark:/87602/m4/M155573); Zambia: L. Tanganyika: Nakaku; F. Ronco, A. Indermaur, H. Büscher, & W. Salzburger, 31-Aug-2014.

Lobochilotes labiata (Boulenger, 1898): UNIBAS IYB9, 1 (2D x-ray CT, MorphoSource http://n2t.net/ark:/87602/m4/M155877); Zambia: Lake Tanganyika: Kalambo (Toby's) Lodge; F. Ronco, A. Indermaur, H. Büscher, & W. Salzburger, 29-Jul-2014. — UNIBAS IYE2, 1 (2D x-ray CT, MorphoSource

http://n2t.net/ark:/87602/m4/M155878); other data same as IYB9. — UNIBAS IYF9, 1 (2D x-ray CT, MorphoSource http://n2t.net/ark:/87602/m4/M155879); other data same as IYB9. — UNIBAS IYG2, 1 (2D x-ray CT, MorphoSource http://n2t.net/ark:/87602/m4/M155880); other data same as IYB9. — UNIBAS IYG3, 1 (2D x-ray CT, MorphoSource http://n2t.net/ark:/87602/m4/M155881); other data same as IYB9.

Petrochromis horii Takahashi & Koblmüller, 2014: UNIBAS IWB9, 1 (2D x-ray CT, MorphoSource http://n2t.net/ark:/87602/m4/M155837); Zambia: L. Tanganyika: Katete 3; F. Ronco, A. Indermaur, H. Büscher, & W. Salzburger, 26-Aug-2014. — UNIBAS IWC1, 1 (2D x-ray CT, MorphoSource http://n2t.net/ark:/87602/m4/M155838); other data same as IWB9. — UNIBAS IWC2, 1 (2D x-ray CT, MorphoSource http://n2t.net/ark:/87602/m4/M155839); other data same as IWB9. — UNIBAS JBF8, 1 (2D x-ray CT, MorphoSource http://n2t.net/ark:/87602/m4/M155840); Zambia: L. Tanganyika: Kanfonki; F. Ronco, A. Indermaur, H. Büscher, & W. Salzburger, 1-Sep-2014. — UNIBAS JBF9, 1 (2D x-ray CT, MorphoSource http://n2t.net/ark:/87602/m4/M155841); other data same as JBF8.

Petrochromis polyodon Boulenger, 1898: UNIBAS IYD4, 1 (2D x-ray CT, MorphoSource http://n2t.net/ark:/87602/m4/M156500); Zambia: Lake Tanganyika: Kalambo (Toby's) Lodge; F. Ronco, A. Indermaur, H. Büscher, & W. Salzburger, 29-Jul-2014. — UNIBAS IYD5, 1 (2D x-ray CT, MorphoSource http://n2t.net/ark:/87602/m4/M156501); other data same as IYD4. — UNIBAS IYE9, 1 (2D x-ray CT, MorphoSource http://n2t.net/ark:/87602/m4/M156502); other data same as IYD4. — UNIBAS IYG5, 1 (2D x-ray CT, MorphoSource http://n2t.net/ark:/87602/m4/M156503); other data same as IYD4. — UNIBAS JXD2, 1 (2D x-ray CT, MorphoSource http://n2t.net/ark:/87602/m4/M156504); Burundi: L. Tanganyika: Nyaruhongoka 2; F. Ronco, A. Indermaur, H. Büscher, & W. Salzburger, 7-Jan-2015.

Pseudosimochromis curvifrons (Poll, 1942): UNIBAS IYG4, 1 (2D x-ray CT, MorphoSource http://n2t.net/ark:/87602/m4/M155544); Zambia: Lake Tanganyika: Kalambo (Toby's) Lodge; F. Ronco, A. Indermaur, H. Büscher, & W. Salzburger, 29-Jul-2014. — UNIBAS JBC1, 1 (2D x-ray CT, MorphoSource http://n2t.net/ark:/87602/m4/M155545); L. Tanganyika: Misepa; F. Ronco, A. Indermaur, H. Büscher, & W. Salzburger, 30-Aug-2014. — UNIBAS JBC2, 1 (2D x-ray CT, MorphoSource http://n2t.net/ark:/87602/m4/M155546); other data same as JBC1. — UNIBAS JBC3, 1 (2D x-ray CT, MorphoSource http://n2t.net/ark:/87602/m4/M155547); other data same as JBC1. — UNIBAS JCA2, 1 (2D x-ray CT, MorphoSource http://n2t.net/ark:/87602/m4/M155548); 31-Jul-2014, other data same as IYG4.

Shuja horei (Günther, 1894): BMNH 1889.1.30.13–15, 3 (syntypes: 2D radiographs in *Genner et al. 2022*: fig. 5c); Lake Tanganyika; E. Coode-Hore. — UNIBAS JDB6, 1 (2D x-ray CT, MorphoSource http://n2t.net/ark:/87602/m4/M155816); Zambia: Lake Tanganyika: Kalambo (Toby's) Lodge; F. Ronco, A. Indermaur, H. Büscher, & W. Salzburger, 1-Aug-2014. — UNIBAS JDC8, 1 (2D x-ray CT, MorphoSource, http://n2t.net/ark:/87602/m4/M155818); other data same as JDB6. — UNIBAS JDD9, 1 (2D x-ray CT, MorphoSource http://n2t.net/ark:/87602/m4/M155819); other data same as JDB6. — UNIBAS JDE1, 1 (2D x-ray CT, MorphoSource http://n2t.net/ark:/87602/m4/M155820); other data same as JDB6. — UNIBAS KAE4, 1 (2D x-ray CT, MorphoSource http://n2t.net/ark:/87602/m4/M155821); Burundi: L. Tanganyika: Nyaruhongoka 2; F. Ronco, A. Indermaur, H. Büscher, & W. Salzburger, 14-Jan-2015.

Simochromis diagramma (Günther, 1894): UNIBAS JDB7, 1 (2D x-ray CT, MorphoSource JDB7, 1 (2D x-ray CT, MorphoSource http://n2t.net/ark:/87602/m4/M155612); Zambia: Lake Tanganyika: Kalambo (Toby's) Lodge; F. Ronco, A. Indermaur, H. Büscher, & W. Salzburger, 1-Aug-2014. — UNIBAS JDB8, 1 (2D x-ray CT, MorphoSource http://n2t.net/ark:/87602/m4/M155613); other data same as JDB7. — UNIBAS JDB9, 1 (2D x-ray CT, MorphoSource http://n2t.net/ark:/87602/m4/M155614); other data same as JDB7. — UNIBAS JDC1, 1 (2D x-ray CT, MorphoSource http://n2t.net/ark:/87602/m4/M155615); other data same as JDB7. — UNIBAS JDC2, 1 (2D x-ray CT, MorphoSource http://n2t.net/ark:/87602/m4/M155616); other data same as JDB7.

Tropheus annectens Boulenger, 1900 (as *T. polli*): UNIBAS LEG6, 1 (2D x-ray CT, MorphoSource http://n2t.net/ark:/87602/m4/M156485); L. Tanganyika: Storo 1; F. Ronco, A. Indermaur, H. Büscher, & W. Salzburger, 5-Jul-2015. — UNIBAS LEG7, 1 (2D x-ray CT, MorphoSource http://n2t.net/ark:/87602/m4/M156486); other data same as LEG6. — UNIBAS LEG8, 1 (2D x-ray CT, MorphoSource http://n2t.net/ark:/87602/m4/M156487); other data same as LEG6. — UNIBAS LEG9, 1 (2D x-ray CT, MorphoSource http://n2t.net/ark:/87602/m4/M156488); other data same as LEG6. — UNIBAS LEH1, 1 (2D x-ray CT, MorphoSource http://n2t.net/ark:/87602/m4/M156489); other data same as LEG6.

Tropheus brichardi Nelissen & Thys van den Audenaerde, 1975: UNIBAS JZA6, 1 (2D x-ray CT, MorphoSource http://n2t.net/ark:/87602/m4/M155376); Burundi: L. Tanganyika: Nyanza Lac; F. Ronco, A. Indermaur, H. Büscher, & W. Salzburger, 11-Jan-2015. — UNIBAS JZA8, 1 (2D x-ray CT, MorphoSource http://n2t.net/ark:/87602/m4/M155377); other data same as JZA6. — UNIBAS JZA9, 1 (2D x-ray CT, MorphoSource http://n2t.net/ark:/87602/m4/M155378); other data same as JZA6. — UNIBAS JZB1, 1 (2D x-ray CT, MorphoSource http://n2t.net/ark:/87602/m4/M155379); other data same as JZA6. — UNIBAS JZB2, 1 (2D x-ray CT, MorphoSource http://n2t.net/ark:/87602/m4/M155380); other data same as JZA6.

Tropheus duboisi Marlier, 1959: UNIBAS KHF7, 1 (2D x-ray CT, MorphoSource http://n2t.net/ark:/87602/m4/M155632); Tanzania: L. Tanganyika: Nondwa Point; F. Ronco, A. Indermaur, H. Büscher, & W. Salzburger, 23-Jun-2015. — UNIBAS KHF8, 1 (2D x-ray CT, MorphoSource http://n2t.net/ark:/87602/m4/M155633); other data same as KHF7. — UNIBAS KHF9, 1 (2D x-ray CT, MorphoSource http://n2t.net/ark:/87602/m4/M155634); other data same as KHF7. — UNIBAS KHG1, 1 (2D x-ray CT, MorphoSource http://n2t.net/ark:/87602/m4/M155635); other data same as KHF7. — UNIBAS KHI7, 1 (2D x-ray CT, MorphoSource http://n2t.net/ark:/87602/m4/M155636); Zambia: L. Tanganyika: Kaku; F. Ronco, A. Indermaur, H. Büscher, & W. Salzburger, 24-Jun-2015.

Tropheus moorii Boulenger, 1898: UNIBAS GPF3, 1 (2D x-ray CT, MorphoSource http://n2t.net/ark:/87602/m4/M156190); Zambia: L. Tanganyika: Chituta; F. Ronco, A. Indermaur, H. Büscher, & W. Salzburger, 3-Sep-2014. — UNIBAS GPF4, 1 (2D x-ray CT, MorphoSource http://n2t.net/ark:/87602/m4/M156191); other data same as GPF3. — UNIBAS IMA9, 1 (2D x-ray CT, MorphoSource http://n2t.net/ark:/87602/m4/M156192); Zambia: L. Tanganyika: Kabwensolo; F. Ronco, A. Indermaur, H. Büscher, & W. Salzburger, 18-Aug-2014. — UNIBAS IMF5, 1 (2D x-ray CT, MorphoSource http://n2t.net/ark:/87602/m4/M156193); 19-Aug-2014, other data same as IMA9. —

UNIBAS IMG6, 1 (2D x-ray CT, MorphoSource http://n2t.net/ark:/87602/m4/M156194); Zambia: Lake Tanganyika: Chitweshiba; F. Ronco, A. Indermaur, H. Büscher, & W. Salzburger, 19-Aug-2014. — UNIBAS IOC2, 1 (2D x-ray CT, MorphoSource http://n2t.net/ark:/87602/m4/M156195); Zambia: L. Tanganyika: Kachese; F. Ronco, A. Indermaur, H. Büscher, & W. Salzburger, 23-Aug-2014. — UNIBAS JBH6, 1 (2D x-ray CT, MorphoSource http://n2t.net/ark:/87602/m4/M156196); Zambia: L. Tanganyika: Mbita Island W; F. Ronco, A. Indermaur, H. Büscher, & W. Salzburger, 2-Sep-2014. — UNIBAS JBH7, 1 (2D x-ray CT, MorphoSource http://n2t.net/ark:/87602/m4/M156197); other data same as JBH6. — UNIBAS JBH9, 1 (2D x-ray CT, MorphoSource http://n2t.net/ark:/87602/m4/M156198); other data same as JBH6. — UNIBAS JBI1, 1 (2D x-ray CT, MorphoSource http://n2t.net/ark:/87602/m4/M156199); other data same as JBH6.

Tylochromini

Tylochromis polylepis (Boulenger, 1900): UNIBAS GPB4, 1 (2D x-ray CT, MorphoSource http://n2t.net/ark:/87602/m4/M156490); Zambia: L. Tanganyika: Mpulungu fishmarket; F. Ronco, A. Indermaur, H. Büscher, & W. Salzburger, 2-Sep-2014. — UNIBAS KCA2, 1 (2D x-ray CT, MorphoSource http://n2t.net/ark:/87602/m4/M156491); Burundi: L. Tanganyika: Bujumbura fishmarket; F. Ronco, A. Indermaur, H. Büscher, & W. Salzburger, 15-Jan-2015. — UNIBAS KCA3, 1 (2D x-ray CT, MorphoSource http://n2t.net/ark:/87602/m4/M156492); other data same as KCA2. — UNIBAS LIC8, 1 (2D x-ray CT, MorphoSource http://n2t.net/ark:/87602/m4/M156493); other data same as GPB4. — UNIBAS LIC9, 1 (2D x-ray CT, MorphoSource http://n2t.net/ark:/87602/m4/M156494); other data same as GPB4. — UNIBAS LID1, 1 (2D x-ray CT, MorphoSource http://n2t.net/ark:/87602/m4/M156495); other data same as GPB4.

Lake Malawi

Pseudocrenilabrini: Cyrtocarina

Alticorpus mentale Stauffer & McKaye, 1988: CUMV 86278, 1 (3D CT image series, MorphoSource http://n2t.net/ark:/87602/m4/M67320); Malawi: L. Malawi: Karonga; M.E. Arnegard, 29-Feb-1996.

Alticorpus peterdaviesi (Burgess & Axelrod, 1973): USNM 210714, 1 (paratype; 2D radiograph); Malawi: L. Malawi: trawled off Monkey Bay in 79 m; H.R. Axelrod, 19-May-1973.

Aristochromis christyi Trewavas, 1935: USNM 329636, 2 (2D radiograph); Malawi: L. Malawi: SW arm, station Marelli I, trawled in 27 m; LMTS, 26-May-1972.

Aulonocara nyassae Regan, 1922: YPM 027961, 1 (3D CT image series, MorphoSource http://n2t.net/ark:/87602/m4/M123649); Malawi; L. Malawi: Nankumba Peninsula, 100 m S of Otter Island; M.K. Oliver, K. McKaye, & T.D. Kocher, 29-Jun-1980.

Aulonocara rostratum Trewavas, 1935: YPM 014345, 1 (3D CT image series, MorphoSource http://n2t.net/ark:/87602/m4/426757); Malawi: L. Malawi: SE arm: LMTS station Michesi I, trawled in 22 m; M.K. Oliver, J. Tarbit, & party, 15-Jun-1971.

Aulonocara stonemani (Burgess & Axelrod, 1973): USNM 210697, 1 (holotype; 2D radiograph); Malawi: L. Malawi: trawled off Monkey Bay in 79 m; H.R. Axelrod, 19-May-1973.

Buccochromis atritaeniatus (Regan, 1922): BMNH 1921.9.6.179–180, 2 (lectotype & a paralectotype; 2D radiograph available at https://data.nhm.ac.uk/object/819fe6cb-eb44-478d-803a-b919f3aced34); Malawi: L. Malawi; R.C. Wood.

Buccochromis heterotaenia (Trewavas, 1935): BMNH 1935.6.14.1419–1420, 2 (lectotype & paralectotype; 2D radiograph available at https://data.nhm.ac.uk/object/d5bc88b1-c051-4603-a4da-a0f877ab014a); Malawi: L. Malawi; C. Christy.

Buccochromis nototaenia (Boulenger, 1902): BMNH 1906.9.7.21, 1 (holotype; 2D radiograph available at https://data.nhm.ac.uk/object/4ba83952-fd77-4d8b-b969-43dc2b27a7a1); Malawi: L. Malawi; J.E. Moore. — YPM 023201, 1 (3D CT image series, MorphoSource http://n2t.net/ark:/87602/m4/M70650); captive.

Buccochromis oculatus (Trewavas, 1935): BMNH 1935.6.14.1442–1444, 3 (paralectotypes; 2D radiograph available at https://data.nhm.ac.uk/object/cb35bb75-1d1b-4246-8c54-ba10326d00af); Malawi: L. Malawi: Bar – Fort Maguire; C. Christy, 1925.

Buccochromis spectabilis (Trewavas, 1935): BMNH 1935.6.14.1448–1452, 2 (1 used; paralectotypes; 2D radiograph available at https://data.nhm.ac.uk/object/1ae4d82e-0798-4c99-9ddc-72c70810c699); Malawi: L. Malawi: north end; C. Christy, 1925.

Caprichromis orthognathus (Trewavas, 1935): AMNH 31767, 4 (2D radiograph); Malawi: L. Malawi; D.H. Eccles, 24-Apr-1969. — YPM 014341, 1 (3D CT image series, MorphoSource http://n2t.net/ark:/87602/m4/M158024); Malawi: L. Malawi: Thumbi Island West in 40–49 m; M.K. Oliver, K. McKaye, & T.D. Kocher, 6–7-Jul-1980.

Champsochromis caeruleus (Boulenger, 1908): BMNH 1935.6.14.1267–1269, 4 (2D radiograph available at https://data.nhm.ac.uk/object/68df0ab0-7b21-4804-a42f-ab38f2b8c70e); Malawi: L. Malawi: Karonga; C. Christy, Oct-1925.

Champsochromis spilorhynchus (Regan, 1922): USNM 265522, 1 (2D radiograph); Malawi: L. Malawi: midway between Thumbi Island West and Domwe Island in 45–55 m; M.K. Oliver, K. McKaye, & T.D. Kocher, 19–20-Aug-1980. — USNM 266872, 1 (2D radiograph); Malawi: L. Malawi: Thumbi Island West near SE corner in 10–20 m; M.K. Oliver, K. McKaye, & T.D. Kocher, 16–17-Jun-1980.

Cheilochromis euchilus (Trewavas, 1935): BMNH 1935.6.14:1016–1017, 2 (lectotype & paralectotype; 2D radiograph); Malawi: L. Malawi: Deep Bay; C. Christy, Aug-1925. — AMNH 222094, 3 (2D radiograph); Malawi: L. Malawi: Maleri Island, ENE shore; M.K. Oliver, A. Mbaye, D. & T.E. Davies, 2-May-1971.

Chilotilapia rhoadesii Boulenger, 1908: AMNH 31860, 2 (2D radiograph); Malawi: L. Malawi: 6 miles from southern extremity; D.H. Eccles, 1-Jul-1965. — AMNH 31861, 1 (2D radiograph); Malawi: L. Malawi:

Monkey Bay off Bweyawanyani; D.H. Eccles, 20-Sep-1967. — YPM 007852, 1 (3D CT image series, MorphoSource http://n2t.net/ark:/87602/m4/M80058); Malawi: L. Malawi: off Namiasi.

Copadichromis jacksoni (Iles, 1960): BMNH 1962.10.18.83–91, 3 (paratypes; 2D radiograph available at https://data.nhm.ac.uk/object/2bb92f4d-97eb-44bc-ae5a-414d0eeab19d); Malawi: L. Malawi: Nkata Bay; T.D. Iles.

Copadichromis quadrimaculatus (Regan, 1922): YPM 014347, 1 (3D CT image series, MorphoSource http://n2t.net/ark:/87602/m4/426269); Malawi: L. Malawi: "S end of Thumbi Island West and Zimbawe Rock"; 22-Oct-1977.

Corematodus taeniatus Trewavas, 1935: USNM 330794, 7 (2D radiograph); Malawi: L. Malawi: SE arm, trawled 2–4 miles off Namiasi in 22–27 m; D.H. Eccles, K. Stride, & party, 12-Aug-1971.

Ctenopharynx nitidus (Trewavas, 1935): BMNH 1935.6.14:1760–1762, 3 (paralectotypes; 2D radiograph); Malawi: L. Malawi: Deep Bay; C. Christy, Aug-1925. — AMNH 31784, 6 (2D radiograph); Malawi: L. Malawi: Nkhata Bay; D.H. Eccles, 24-Apr-1969. — AMNH 31790, 1 (2D radiograph); Malawi: L. Malawi: 4 miles SE of Monkey Bay; D.H. Eccles, 4-Sep-1961. — AMNH 31836, 6 (2D radiograph); Malawi: L. Malawi: Salima; D.H. Eccles, 19-Feb-1958.

Ctenopharynx pictus (Trewavas, 1935): BMNH 1935.6.14.1777–1778, 1 (paralectotype; 2D radiograph available at https://data.nhm.ac.uk/object/d327a766-fe78-440c-9f7d-b2d3350ff440); Malawi: L. Malawi: Monkey Bay; C. Christy, Jul-1925.

Cyrtocara moorii Boulenger, 1902: BMNH 1906.9.7:26, 1 (holotype; 2D radiograph); L. Malawi; J.E.S. Moore. — AMNH 31772, 1 (2D radiograph); Malawi: L. Malawi: Nkhata Bay; D.H. Eccles, 21-May-1959. — AMNH 31781, 3 (2D radiograph); Malawi: L. Malawi; D.H. Eccles, 24-Apr-1969. — AMNH 31831, 1 (2D radiograph); Malawi: L. Malawi: Nkhata Bay; D.H. Eccles, 1960.

Dimidiochromis compressiceps (Boulenger, 1908): AMNH 11722, 1 (2D radiograph); Malawi: L. Malawi: Karonga; R. & L. Boulton, 30-Jun-1929. — AMNH 11737, 1 (2D radiograph); Malawi: L. Malawi: Deep Bay; R. & L. Boulton, 4-July-1929. — AMNH 17826, 1 (2D radiograph); Malawi: L. Malawi: Deep Bay; R. & L. Boulton, 4-July-1929. — AMNH 31783, 4 (2D radiograph); Malawi: L. Malawi: Monkey Bay; W.C. Ambali, 14-Aug-1968. — AMNH 31785, 4 (2D radiograph); Malawi: L. Malawi: Nkhata Bay; D.H. Eccles, 11-Dec-1962. — AMNH 31835, 1 (2D radiograph); Malawi: L. Malawi: Nkhata Bay; D.H. Eccles, 1960.

Dimidiochromis kiwinge (Ahl, 1926): BMNH 1935.6.14.1079–1084, 3 (2D radiograph available at https://data.nhm.ac.uk/object/c9cbffe7-fce7-4cd4-a035-61dd4f44864d); Malawi: L. Malawi: Deep Bay; C. Christy, Aug-1925.

Docimodus evelynae Lewis, 1976: BMNH 1975.3.10.1, 1 (holotype; 2D radiograph available at https://data.nhm.ac.uk/object/fecd5889-8bae-4ceb-8ef9-a176a4304c2e); Malawi: L. Malawi: Nkhata Bay, mouth of south bay; D.H. Eccles, 28-Aug-1962. — USNM 216603, 1 (paratype; 2D radiograph); Malawi: L. Malawi: Monkey Bay; 16-Nov-1962.

Docimodus johnstoni Boulenger, 1897: USNM 330496, 2 (2D radiograph); Malawi: L. Malawi: SE arm, LMTS station Nkopi III, trawled in 55 m; 6-Jun-1972. — CUMV 86290, 1 (3D CT image series, MorphoSource http://n2t.net/ark:/87602/m4/458898); Malawi: L. Malawi: Karonga; M. Arnegard, 3-Mar-1996.

Exochochromis anagenys Oliver, 1989: USNM 304657, 1 (holotype; 2D radiograph); Malawi: L. Malawi: Thumbi Island West in 27–60 m; M.K. Oliver, K. McKaye, & T.D. Kocher, 3–4-Aug-1980. — USNM 304659, 1 (paratype; 2D radiograph); captive, exported from Malawi: L. Malawi; S. Grant, 1975. — PSU 13375, 2 (2D radiograph); Malawi: L. Malawi: Mazinzi Reef; J.R. Stauffer, 21-Mar-1985. — PSU 13376, 1 (2D radiograph); Malawi: L. Malawi: Mazinzi Reef; J.R. Stauffer, 17-Mar-1985. — YPM 023205, 1 (3D CT image series, MorphoSource http://n2t.net/ark:/87602/m4/M71680); captive.

Fossorochromis rostratus (Boulenger, 1899): YPM 027097, 1 (3D CT image series, MorphoSource http://n2t.net/ark:/87602/m4/426317); captive.

Hemitaeniochromis urotaenia (Regan, 1922): YPM 007803, 1 (3D CT image series, MorphoSource http://n2t.net/ark:/87602/m4/M158659); Malawi: L. Malawi: Monkey Bay; D.H. Eccles.

Hemitilapia oxyrhynchus Boulenger, 1902: AMNH 31880, 6 (2D radiograph); Malawi: L. Malawi: Nkhata Bay; D.H. Eccles, 24-Apr-1969. — BMNH 1972.12.18.45–48, 4 (2D radiograph); Malawi: L. Malawi: SE arm: Mvunguti; R.H. Lowe.

Lethrinops gossei Burgess & Axelrod, 1973: USNM 210694, 1 (holotype; 2D radiograph); Malawi: L. Malawi: off Monkey Bay in 79 m; H.R. Axelrod, May 1973. — USNM 210715, 1 (paratype; 2D radiograph); same data as holotype.

Lethrinops lethrinus (Günther, 1894): AMNH 58000, 2 (2D radiograph); Malawi: L. Malawi: SE arm off Michesi in 22 m; M.K. Oliver, J. Tarbit, H. Klokk & party, 14-Jun-1971. — YPM 014443, 1 (3D CT image series, MorphoSource http://n2t.net/ark:/87602/m4/M141115); Malawi: L. Malawi: across Mazinzi Bay in 9–10 m; M.K. Oliver, K. McKaye, & T.D. Kocher, 28-Jun-1980.

Lethrinops polli (Burgess & Axelrod 1973): USNM 210695, 1 (holotype; 2D radiograph); Malawi: L. Malawi: off Monkey Bay in 79 m; H.R. Axelrod, May 1973.

Lichnochromis acuticeps Trewavas, 1935: YPM 014590, 1 (3D CT image series, MorphoSource http://n2t.net/ark:/87602/m4/M70872); Malawi: L. Malawi: Nankumba Peninsula 300 m E of Otter Island; M.K. Oliver & T.D. Kocher, 4-Jun-1980.

Mchenga cyclicos (Stauffer, LoVullo, & McKaye, 1993): USNM 324569, 1 (paratype; 3D CT image series, MorphoSource http://n2t.net/ark:/87602/m4/M141169); Malawi: L. Malawi: Kanchedza Island; J.R. Stauffer, 31-Jan-1989.

Mchenga inornata (Boulenger, 1908): BMNH 1908.10.27.101–102, 2 (lectotype & paralectotype; 2D radiograph available at https://data.nhm.ac.uk/object/b849adb6-0455-4bb3-a7f4-7456ead7218f); Malawi: L. Malawi; E. Rhoades.

Mchenga sp.: AMNH 31855, 23 (2D radiograph); Malawi: L. Malawi; D.H. Eccles, 24-Apr-1969.

Mylochromis formosus (Trewavas, 1935): BMNH 1935.6.14.1454–1455, 2 (lectotype & paralectotype; 2D radiograph); Malawi: L. Malawi: Vua; C. Christy, Aug/Sep-1925.

Mylochromis gracilis (Trewavas, 1935): BMNH 1935.6.14.1456–1458, 3 (lectotype & paralectotypes; 2D radiograph); Malawi: L. Malawi: Monkey Bay; C. Christy, Jul-1925.

Mylochromis guentheri (Regan, 1922): AMNH 31808, 2 (2D radiograph); Malawi: L. Malawi: Nkhata Bay; D.H. Eccles, 11-Jun-1958.

Mylochromis lateristriga (Günther, 1864): AMNH 31770, 2 (2D radiograph); Malawi: upper Shire River between Fort Johnston and Lake Malombe; D.H. Eccles, 21-Dec-1966. — AMNH 31842, 2 (2D radiograph); Malawi: L. Malawi: Monkey Bay; B. Gunn, 9-Jan-1964.

Mylochromis spilostichus (Trewavas, 1935): BMNH 1935.6.14.1459, 1 (holotype); Malawi: L. Malawi: Monkey Bay; C. Christy, Jul-1925. — BMNH 1969.3.11.6, 1 (2D radiograph); Malawi: L. Malawi: just outside Monkey Bay; D.H. Eccles, 23-Feb-1968. — AMNH 221934, 6 (2D radiograph); Malawi: L. Malawi: SE arm off Mazinzi in 42 m; M.K. Oliver & party, 25-Jun-1971.

Mylochromis subocularis (Günther, 1894): USNM 330657, 2 (2D radiograph); Malawi: L. Malawi: SE arm off Nkopi in 42-55 m; LMTS, 6-Jun-1972.

Naevochromis chrysogaster (Trewavas, 1935): AMNH 31782, 1 (2D radiograph); Malawi: L. Malawi: Monkey Bay off Zambo; D.H. Eccles, 5-Feb-1965. — AMNH 31793, 1 (2D radiograph); Malawi: L. Malawi: Nankumba Peninsula off Chembe; D.H. Eccles, 8-Aug-1961. — BMNH 1935.6.14.1640–1641, 2 (lectotype & paralectotype; 2D radiograph); Malawi: L. Malawi: SW arm; C. Christy, Dec-1925/Jan-1926. — BMNH 1935.6.14.1642, 1 (paralectotype; 2D radiograph); Malawi: L. Malawi: Karonga; C. Christy, Oct-1925.

Nimbochromis fuscotaeniatus (Regan, 1922): AMNH 31805, 1 (2D radiograph); Malawi: L. Malawi: Monkey Bay; W.C. Ambali, 30-Sep-1964. — AMNH 31856, 1 (2D radiograph); Malawi: L. Malawi: Monkey Bay; D.H. Eccles, 2-Jun-1965.

Nimbochromis livingstonii (Günther, 1894): AMNH 31777, 1 (2D radiograph); Malawi: L. Malawi; D.H. Eccles, 24-Apr-1969. — AMNH 31803, 5 (2D radiograph); Malawi: L. Malawi; D.H. Eccles, 24-Apr-1969.

Nimbochromis venustus (Boulenger, 1908): AMNH 221885, 4 (2D radiograph); Malawi: L. Malawi: trawled between S end Thumbi Island West and just SW of Ilala Gap in 27 m; K. Stride, M.K. Oliver, & party, 14 July 1971.

Nyassachromis leuciscus (Regan, 1922): BMNH 1921.9.6.188, 1 (lectotype; 2D radiograph available at https://data.nhm.ac.uk/object/4f663ff6-ee36-424c-a7f4-b977b1b5a3e5); L. Malawi; R. Wood. — USNM 266852, 5 (2D radiograph); Malawi: L. Malawi: SE arm: otter trawl across Mazinzi Bay in 9–10 m; M.K. Oliver, K. McKaye, & T.D. Kocher, 28-Jun-1980.

Nyassachromis microcephalus (Trewavas, 1935): BMNH 1935.6.14.873–874, 2 (lectotype & paralectotype; 2D radiograph available at https://data.nhm.ac.uk/object/3e80480d-2e7a-4d3c-a61e-eb9e2da8524d); Malawi: L. Malawi: Monkey Bay; C. Christy, Jul-1925.

Nyassachromis nigritaeniatus (Trewavas, 1935): USNM 266863, 4 (2D radiograph); Malawi: L. Malawi: SE arm: trawled across Mazinzi Bay in 9–10 m; M.K. Oliver, K. McKaye, & T.D. Kocher, 28-Jun-1980.

Nyassachromis purpurans (Trewavas, 1935): BMNH 1935.6.14. 895–902, 1 (lectotype or paralectotype; 2D radiograph available at https://data.nhm.ac.uk/object/556519f2-ec04-4de6-9051-f594e7af91ae); Tanzania: L. Malawi: Mwaya or Mbasi River mouth; C. Christy, Oct-1925.

Otopharynx argyrosoma (Regan, 1922): BMNH 1908.10.27:99, 1 (holotype; 2D radiograph); L. Malawi; E.L. Rhoades. — Uncatalogued, MKO71-VI-26, 2 (2D radiograph); Malawi: L. Malawi: trawled Mazinzi Bay in 5–9 m; M.K. Oliver, J. Tarbit, K. Stride, & party, 26-Jun-1971.

Otopharynx decorus (Trewavas, 1935): BMNH 1935.6.14.1651, 1 (lectotype; 2D radiograph); Malawi: L. Malawi: Vua; C. Christy, Aug/Sep-1925. — BMNH 1935.6.14.1652–1654, 3 (paralectotypes; 2D radiograph); other data same as 1935.6.14.1651. — BMNH 1935.6.14.1655–1656, 2 (paralectotypes; 2D radiograph); Malawi: L. Malawi: S end; C. Christy, 1925.

Otopharynx cf. *heterodon* (Trewavas, 1935): AMNH 31886, 19 (2D radiograph); Malawi: L. Malawi: Nkhata Bay; D.H. Eccles, 16-Jul-1958.

Otopharynx heterodon (Trewavas, 1935): BMNH 1935.6.14.1584–1585, 2 (syntypes; 2D radiograph); Malawi: L. Malawi: Deep Bay; C. Christy, Aug-1925. — BMNH 1935.6.14.1586, 1 (syntype; 2D radiograph); Malawi: L. Malawi: Monkey Bay; C. Christy, Jul-1925. — BMNH 1935.6.14.1587–1589, 5 (syntypes; 2D radiograph); other data same as 1935.6.14.1586.

Otopharynx lithobates Oliver, 1989: BMNH 1974.7.5.1, 1 (holotype; 2D radiograph); Malawi: L. Malawi: N end Thumbi Island West; A. Mbaye, 24-May-1971. — BMNH 1974.7.5.2–3, 2 (paratypes; 2D radiograph); Malawi: L. Malawi: E side Thumbi Island West; M.K. Oliver, A. Mbaye, Kingsize, & D. Davies, 30-Apr-1971. — Uncatalogued, MKO71-VIII-10, 1 (2D radiograph); Malawi: L. Malawi: Monkey Bay; M.K. Oliver & D.H. Eccles, 10-Aug-1971. — Uncatalogued, MKO71-VI-5a, 1 (2D radiograph); Malawi: L. Malawi: Monkey Bay, Thumbi Island East; M.K. Oliver, R.E. Furzer, & R. Furzer, 5-Jun-1971.

Otopharynx ovatus (Trewavas, 1935): BMNH 1935.6.14.1487–1489, 3 (lectotype & para-lectotypes; 2D radiograph available at https://data.nhm.ac.uk/object/478392b8-90ad-42f6-975f-e916c5131462); Malawi: L. Malawi: south end; C. Christy, 1925. — AMNH 31826, 1 (2D radiograph); Malawi: L. Malawi: Monkey Bay; D.H. Eccles, 6-Feb-1964. — AMNH 31832, 1 (2D radiograph); Malawi: L. Malawi: submerged reef 4 miles NE of Monkey Bay; D.H. Eccles, 14-Feb-1964.

Otopharynx selenurus Regan, 1922: BMNH 1935.6.14.1671–1675, 6 (2D radiograph); Malawi: L. Malawi: Vua; C. Christy, Aug/Sep-1925.

Otopharynx speciosus (Trewavas, 1935): AMNH 31791, 3 (2D radiograph); Malawi: L. Malawi: off Chembe beach; D.H. Eccles, 26-Sep-1968. — BMNH 1956.6.12.15–16, 2 (2D radiograph); Tanzania: L. Malawi: Mbampa Bay; P.B.N. Jackson.

Otopharynx tetraspilus (Trewavas, 1935): AMNH 31802, 3 (2D radiograph); Malawi: upper Shire River between L. Malawi and L. Malombe; D.H. Eccles, 21-Dec-1966. — AMNH 221783, 1 (2D radiograph, now c+s); Malawi: L. Malawi: trawled SE arm off Namiasi in 22 m; M.K. Oliver, J. Tarbit, & party, 15-Jun-1971. — YPM 014153, 4 (2D radiograph); Malawi: L. Malawi: trawled SE arm off Namiasi in 22 m; M.K. Oliver, J. Tarbit, & party, 15-Jun-1971.

Otopharynx tetrastigma (Günther, 1894): BMNH 1893.11.15.34–37, 2 (lectotype & paralectotype; 2D radiograph); Malawi: L. Malawi and upper Shire River. — BMNH 1935.6.14.1567–1577, 14 (2D radiograph); Malawi: L. Malawi: Mwaya; C. Christy, Oct-1925.

Placidochromis hennydaviesae (Burgess & Axelrod, 1973): USNM 210698, 1 (holotype; 2D radiograph); Malawi: L. Malawi: off Monkey Bay in 79 m; H.R. Axelrod.

Placidochromis johnstoni (Günther, 1894): AMNH 31775, 2 (2D radiograph); Malawi: L. Malawi: Nkhata Bay; D.H. Eccles, 1960. — AMNH 31776, 1 (2D radiograph); Malawi: L. Malawi; D.H. Eccles, 24-Apr-1969. — AMNH 222058, 1 (2D radiograph); Malawi: L. Malawi: Monkey Bay; D.H. Eccles & M.K. Oliver, 31-Jul-1968.

Placidochromis longimanus (Trewavas, 1935): USNM 330603, 28 (2D radiograph); Malawi: L. Malombe, bottom trawled, station Malombe A; D.H. Eccles, 31-July-1972.

Placidochromis macrognathus Hanssens, 2004: Uncatalogued, 2 (2D radiograph); MKO71-VII-14a (note: incorrectly labeled as MKO71-VII-15); between N end Thumbi Island West and center of W shore Domwe Island, trawled in 64–73 m; K. Stride, M.K. Oliver, & party, 14-Jul-1971.

Placidochromis milomo Oliver, 1989: AMNH 92694, 3 (2D radiograph); Malawi: L. Malawi: Thumbi Island West, Mitande rocks; P.N. Reinthal, 30-Jul-1988. — USNM 265487, 1 (2D radiograph); Malawi: L. Malawi: Thumbi Island [West]; K.R. McKaye & J.R. Stauffer, 26–27-Aug-1983.

Protomelas annectens (Regan, 1922): BMNH 1935.6.14.847–852, 2 (2D radiograph available at https://data.nhm.ac.uk/object/1c0074ad-15b4-4e23-9427-7886ea304448); Malawi: L. Malawi: S end; C. Christy, 1925.

Protomelas fenestratus (Trewavas, 1935): AMNH 221891, 10 (2D radiograph); Malawi: L. Malawi: SE arm: Boadzulu Island; M.K. Oliver & party, 15-Aug-1971. — AMNH 222022 (2D radiograph), full sample of 26 radiographed (this catalog number now lists only 14 specimens in fluid & 1 c+s); Malawi: L. Malawi: SE arm: N end Mazinzi Bay: cobble beach at Chingubi Point; M.K. Oliver & D.H. Eccles, 1-Aug-1971.

Protomelas insignis (Trewavas, 1935): BMNH 1935.6.14.839–843, 2 (syntypes when imaged, might include lectotype; 2D radiograph available at https://data.nhm.ac.uk/object/cabd2b63-ca9e-4185-84ba-2ba3317cd15b); Malawi: L. Malawi: Monkey Bay; C. Christy, Jul-1925.

Protomelas kirkii (Günther, 1894): YPM 024312, 1 (3D CT image series, MorphoSource http://n2t.net/ark:/87602/m4/426107); Malawi: L. Malawi: Nankumba Peninsula: off Cape Maclear Research Station; M.K. Oliver, K. McKaye, & T.D. Kocher, 23-Aug-1980. — BMNH 1893.11.15.7–8, 2 (lectotype & a paralectotype; 2D radiograph available at https://data.nhm.ac.uk/object/879a000b-1c2b-44f3-b338-696d5e93ecd3); Malawi: L. Malawi and upper Shire River; H.H. Johnston.

Protomelas labridens (Trewavas, 1935): YPM 014519, 1 (3D CT image series, MorphoSource http://n2t.net/ark:/87602/m4/426309); Malawi: L. Malawi: Nankumba Peninsula: off Cape Maclear Research Station; M.K. Oliver, K. McKaye, & T.D. Kocher, 7-Aug-1980.

Protomelas ornatus (Regan, 1922): BMNH 1921.9.6.112, 1 (holotype; 2D radiograph available at https://data.nhm.ac.uk/object/e5f66474-4465-4889-a339-fc5d206fc879); L. Malawi; R. Wood. — BMNH 1935.6.14.1013, 1 (holotype of *P. festivus* = *P. ornatus*; 2D radiograph available at https://data.nhm.ac.uk/object/4d7b3a4d-ca8a-46bb-b7eb-bad4dbb3e096); Malawi: L. Malawi: N'kudzi; C. Christy, Nov-1925.

Protomelas spilopterus (Trewavas, 1935): AMNH 31882, 1 (2D radiograph); Malawi: L. Malawi: Nkhata Bay; D.H. Eccles, 24-Apr-1969. — AMNH 31761, 1 (2D radiograph); Malawi: L. Malawi; D.H. Eccles, 24-Apr-1969. — AMNH 31846, 1 (2D radiograph); Malawi: L. Malawi: Monkey Bay; D.H. Eccles, 7-Sep-1963. — AMNH 31884, 1 (2D radiograph); Malawi: L. Malawi: Monkey Bay: Zambo; D.H. Eccles, 24-Apr-1969. — BMNH 1935.6.14.644–647, 2 (paralectotypes; 2D radiograph available at https://data.nhm.ac.uk/object/a35bfe3e-e44f-4b3f-bd6a-3e736ece45d0); Malawi: L. Malawi: south end; C. Christy, 1925.

Protomelas taeniolatus (Trewavas, 1935): BMNH 1961.12.1.355, 1 (holotype of *Haplochromis cancellus* = *P. taeniolatus*; 2D radiograph available at https://data.nhm.ac.uk/object/c60bab07-ae49-4a63-9312-2ff4c8351aaf); corrected locality: L. Malawi.

Protomelas triaenodon (Trewavas, 1935): AMNH 31778, 3 (2D radiograph); Malawi: upper Shire River between Fort Johnston and Lake Malombe; D.H. Eccles, 21-Dec-1966.

Sciaenochromis ahli (Trewavas, 1935): BMNH 1935.6.14.1460–1468, 10 (2D radiograph); Malawi: L. Malawi: SW arm; C. Christy, Dec-1925/Jan-1926. — BMNH 1935.6.14.1469–1471, 3 (2D radiograph); Malawi: L. Malawi: S end; C. Christy, 1925. — BMNH 1935.6.14.1472–1473, 3 (2D radiograph); Malawi: L. Malawi: Bar – Fort Maguire; C. Christy, 1925.

Stigmatochromis modestus (Günther, 1894): AMNH 31792, 1 (2D radiograph); Malawi: L. Malawi: Monkey Bay; D.H. Eccles, 18-Nov-1966. — AMNH 31794, 1 (2D radiograph); Malawi: L. Malawi: submerged 4 miles NE of Monkey Bay; D.H. Eccles, 14-Feb-1964. — AMNH 31828, 1 (2D radiograph); Malawi: L. Malawi: Monkey Bay; D.H. Eccles, 22-Nov-1962. — BMNH 1893.1.17.5, 1 (holotype; 2D radiograph); L. Malawi; J.A. Williams. — BMNH 1969.3.11.4, 1; Malawi: L. Malawi: Zimbawe Rock; D.H. Eccles, 27-Nov-1963.

Stigmatochromis pholidophorus (Trewavas, 1935): BMNH 1935.6.14.1544, 1 (holotype; 2D radiograph); Malawi: L. Malawi: Vua; C. Christy, Aug/Sep-1925. — BMNH 1969.10.10.1–2, 2 (2D radiograph); Malawi: L. Malawi: Nkhata Bay; D.H. Eccles, 27-Jan-1960. — BMNH unregistered as of 1972, 2 (2D radiograph).

Stigmatochromis pleurospilus (Trewavas, 1935): BMNH 1935.6.14.1475, 1 (holotype; 2D radiograph); Tanzania: L. Malawi: Lupembe; C. Christy, Oct-1925.

Stigmatochromis woodi (Regan, 1922): BMNH 1921.9.6.139–144, 5 (lectotype & paralectotypes; 2D radiograph); Malawi: L. Malawi; R.C. Wood. — BMNH 1935.6.14.1541–1542, 2 (2D radiograph); Tanzania: L. Malawi: Lupembe; C. Christy, Oct-1925.

Taeniochromis holotaenia (Regan, 1922): YPM 007827, 1 (3D CT image series, MorphoSource http://n2t.net/ark:/87602/m4/426621); Malawi: L. Malawi; D.H. Eccles, 18-Nov-1972.

Taeniolethrinops praeorbitalis (Regan, 1922): YPM 014420, 1 (3D CT image series, MorphoSource http://n2t.net/ark:/87602/m4/M72215); Malawi: L. Malawi: SE arm: trawled across Mazinzi Bay in 9–10 m; M.K. Oliver, K. McKaye, & T.D. Kocher, 28-Jun-1980.

Tramitichromis brevis (Boulenger, 1908): YPM 007847, 1 (3D CT image series, MorphoSource http://n2t.net/ark:/87602/m4/426139); Malawi: L. Malawi: Monkey Bay; D.H. Eccles, 22-May-1973.

Trematocranus labifer (Trewavas, 1935): BMNH 1935.6.14.1643–1644, 2 (paralectotypes; 2D radiograph); Malawi: upper Shire River: Fort Johnston; C. Christy, 1925. — BMNH 1935.6.14.1647, 1 (paralectotype; 2D radiograph); Malawi: L. Malawi: Karonga; C. Christy, Oct-1925. — BMNH 1935.6.14.1648, 1 (paralectotype; 2D radiograph); Malawi: L. Malawi: SW arm; C. Christy, Dec-1925/Jan-1926.

Trematocranus microstoma Trewavas, 1935: USNM 227923, 4 (2D radiograph); Malawi: L. Malawi: trawled at Sungu East in 9 m; D.H. Eccles, 29-Jul-1971. — AMNH 226033, 5 (2D radiograph); Malawi: L. Malombe; E. Fleet, 1-Aug-1972.

Trematocranus placodon (Regan, 1922): USNM 227921, 3 (2D radiograph); Malawi (original label illegible); D.H. Eccles.

Tyrannochromis macrostoma (Regan, 1922): YPM 014282, 1 (3D CT image series, MorphoSource http://n2t.net/ark:/87602/m4/426761); Malawi: L. Malawi: Nankumba Peninsula: 250 m S of Otter Island; M.K. Oliver, K. McKaye, & T.D. Kocher, 8-Jul-1980.

Tyrannochromis nigriventer Eccles, 1989: BMNH 1956.6.4.6, 1 (holotype; 2D radiograph available at https://data.nhm.ac.uk/object/644ff398-2fe4-4d74-91eb-872675eb24de); Malawi: L. Malawi: Nkhata Bay; G. Fryer, 21-Jan-1955.

Pseudocrenilabrini: Pseudotropheina

Abactochromis labrosus (Trewavas, 1935): BMNH 1935.6.14.321, 1 (Holotype; 2D radiograph); Malawi: L. Malawi: Deep Bay; C. Christy, Aug-1925. — BMNH unregistered, 1 (2D radiograph); Malawi: L. Malawi.

— YPM 021602, 1 (2D radiograph); Malawi: L. Malawi: Likoma Island; T.E. Davies, Aug-1973. — RMCA 164900, 1 (2D radiograph); Mozambique: L. Nyassa: Vila Cabral environs; M. Costa, Feb-1956. — RMCA 99-041-P-1323, 1 (2D radiograph); Mozambique: L. Nyassa: Metangula: Tchulutcha Reef; SADC/GEF Project, 25-May-1998. — RMCA 99-041-P-1364, 1 (2D radiograph); Mozambique: L. Nyassa: Likwanje Reef; SADC/GEF Project, 25-May-1998.

Chindongo bellicosus Li, Konings, & Stauffer, 2016: AMNH 31897, 2 (2D radiograph); Malawi: L. Malawi: Monkey Bay; D.H. Eccles, 20-Aug-1966. — YPM 007857, 1 (3D CT image series, MorphoSource http://n2t.net/ark:/87602/m4/M70668); Malawi: L. Malawi: Monkey Bay; Sep-1973. — Uncatalogued, MKO80-93, 3 (c+s); Malawi: L. Malawi: Domwe Island, W shore; M.K. Oliver, K. McKaye, & T.D. Kocher, 3-Aug-1980.

Chindongo minutus (Fryer, 1956): AMNH 31900, 1 (2D radiograph); Malawi: L. Malawi: Nkhata Bay; D.H. Eccles, May-1955.

Cyathochromis obliquidens Trewavas, 1935: AMNH 233482, 2 (2D radiograph); Malawi: L. Malawi: Monkey Bay; M.K. Oliver, 31-Jul-1968. — Uncatalogued, MKO68-7-31, 1 (c+s); Malawi: L. Malawi: Monkey Bay; M.K. Oliver, 31-Jul-1968. — YPM 024973, 1 (3D CT image series, MorphoSource http://n2t.net/ark:/87602/m4/426191); Malawi: L. Malawi: Nankumba Peninsula, off Cape Maclear Research Station; M.K. Oliver, K. McKaye, & T.D. Kocher, 7-Aug-1980.

Cynotilapia afra (Günther, 1894): USNM 270440, 1 (3D CT image series, MorphoSource http://n2t.net/ark:/87602/m4/M104054) ; Malawi: L. Malawi: Thumbi Island West, S end; P.N. Reinthal, 7-Nov-1984.

Cynotilapia sp.: AMNH 31892, 4 (2D radiograph); Malawi: L. Malawi: Nkhata Bay; D.H. Eccles, 1955.

Genyochromis mento Trewavas, 1935: AMNH 31903, 1 (2D radiograph); Malawi: L. Malawi: Monkey Bay; D.H. Eccles, 14-Jan-1969. — AMNH 220337, 1 (2D radiograph); Malawi: L. Malawi: Jalo Reef N of Nkhotakota; A. Konings, 27-Oct-1996. — AMNH 233496, 6 (2D radiograph); Malawi: L. Malawi: Maleri Island; M.K. Oliver, A. Mbaye, T.E. Davies, & T. Davies, 2-May-1971. — Uncatalogued, MKO80-70, 2 (c+s); Malawi: L. Malawi: Nkhata Bay; M.K. Oliver, K. McKaye, & T.D. Kocher, 21-Jul-1980.

Gephyrochromis lawsi Fryer, 1957: AMNH 31863, 1 (2D radiograph); Malawi: L. Malawi: Nkhata Bay; D.H. Eccles, 24-Apr-1969.

Gephyrochromis moorii Boulenger, 1901: Uncatalogued, MKO80-40, 3 (c+s); Malawi: L. Malawi: trawled across Mazinzi Bay in 9–10 m; M.K. Oliver, K. McKaye, & T.D. Kocher. — YPM 028230, 1 (3D CT image series, MorphoSource http://n2t.net/ark:/87602/m4/M70666); captive.

Iodotropheus sprengerae Oliver & Loiselle, 1972: USNM 207012, 1 (paratype; 2D radiograph); Malawi: L. Malawi: Boadzulu Island (presumed); T.E. Davies to aquarium trade. — USNM 207013, 1 (paratype; 2D radiograph); Malawi: L. Malawi: Boadzulu Island (presumed); T.E. Davies to aquarium trade, Feb. 1971. — USNM 207014, 1 (paratype; 2D radiograph); Malawi: L. Malawi: Boadzulu Island (presumed); T.E. Davies to aquarium trade. — USNM 207015, 1 (paratype; 2-D radiograph); Malawi: L. Malawi: Boadzulu

Island (presumed); T.E. Davies to aquarium trade. — MCZ 48015, 1 (3D CT image series, MorphoSource http://n2t.net/ark:/87602/m4/M88242) ; Malawi: L. Malawi (presumed). — Uncatalagued, 1 (c+s); wild captive via L. Finley, 1972.

Labeotropheus fuelleborni Ahl, 1926: AMNH 19128, 7 (2D radiograph); Malawi: L. Malawi: Deep Bay; R. & L. Boulton, 4-Jul-1929. — AMNH 31894, 4 (2D radiograph); Malawi: L. Malawi: Monkey Bay; D.H. Eccles, 16-Nov-1962. — AMNH 31906, 4 (2D radiograph); Malawi: L. Malawi: Monkey Bay: Thumbi Island East; D.H. Eccles, 20-Aug-1966.

Labeotropheus trewavasae Fryer, 1956: AMNH 31890, 1 (2D radiograph); Malawi: L. Malawi: Nkhata Bay; D.H. Eccles, 30-Apr-1959. — AMNH 31893, 1 (paratype; 2D radiograph); Malawi: L. Malawi: Nkhata Bay; D.H. Eccles, 21-Oct-1955. — AMNH 31899, 1 (2D radiograph); Malawi: L. Malawi: Nkhata Bay; D.H. Eccles, 24-Apr-1969.

Labidochromis caeruleus Fryer, 1956: MFRU uncatalogued, 1 (paratype *fide* Eccles; 2D radiograph); no data available.

Labidochromis freibergi Johnson, 1974: AMNH 33466, 1 (holotype; 2D radiograph); Malawi: L. Malawi: off Likoma Island; T.E. Davies & party, 10-Jul-1976.

Labidochromis joanjohnsonae Johnson, 1974: AMNH 33464, 1 (holotype; 2D radiograph); Malawi: L. Malawi: off Likoma Island; T.E. Davies & party, 10-Jul-1976.

Labidochromis pallidus Lewis, 1982: YPM 014259, 1 (2D radiograph); Malawi: L. Malawi: Maleri Island, E shore; T.E. Davies, 28-Apr-1971. — YPM 014137, 12 (2D radiograph); Malawi: L. Malawi: Maleri Island, E shore; M.K. Oliver, 28-Apr-1971. — AMNH 232148, 10 (2D radiograph); Malawi: L. Malawi.

Labidochromis shiranus Lewis, 1982: BMNH 1935.6.14:335, 1 (2D radiograph); Malawi: L. Malawi: SE arm: Nkudzi Bay; C. Christy, Nov-1925.

Labidochromis textilis Oliver, 1975: BMNH 1975.5.27:9, 1 (holotype; 2D radiograph); Malawi: L. Malawi: Likoma Island (presumed); T.E. Davies & party. — AMNH 33465, 1 (paratype; 2-D radiograph; Note: Lewis (1982) reassigned some paratypes to other, newly recognized species but attributed this specimen to *L. textilis*); Mozambique: L. Nyasa: probably vicinity of Meponda; T.E. Davies & party.

Labidochromis vellicans Trewavas, 1935: BMNH 1935.6.14:326–335, 6 (syntypes; 2D radiograph; note: Lewis (1982) reassigned one specimen in this series to *L. maculicauda* but it is unclear which one on the radiograph this was); Malawi: L. Malawi: SE arm: Nkudzi Bay; C. Christy, Nov-1925. — BMNH 1965.10.26:14–21, 8 (2D radiograph); Malawi: L. Malawi: Nkhata Bay. — YPM 014268, 16 (2D radiograph); Malawi: L. Malawi: Maleri Island, ENE shore; M.K. Oliver, 2-May-1971.

Maylandia aurora (Burgess, 1976): USNM 215292, 1 (holotype; 2D radiograph available at http://n2t.net/ark:/65665/3b5e26112-8396-4926-a5bf-f928aff715f1); Malawi: L. Malawi: “probably Likoma Island;” J. Freiberg.

Maylandia koningsi (Stauffer, 2018): PSU 12891, 1 (holotype; 3D CT image series, MorphoSource http://n2t.net/ark:/87602/m4/M39556); Malawi: Lake Malawi: Likoma Island: Membe Point; J.R. Stauffer, 5-Aug-2005.

Maylandia lanisticola (Burgess, 1976): USNM 216266, 1 (holotype; 2D radiograph available at http://n2t.net/ark:/65665/3524539ff-8ea3-471a-9b80-af919c608225); Malawi: L. Malawi: off Cape Maclear in 6–15 m; W.E. Burgess & J. Pindani.

Maylandia zebra (Boulenger, 1899): AMNH 19127, 3 (2D radiograph); Malawi: L. Malawi: Deep Bay; R. & L. Boulton, 4-Jul-1929. — AMNH 31891, 1 (2D radiograph); Malawi: L. Malawi: Monkey Bay; D.H. Eccles, 16-Nov-1962. — AMNH 31907, 1 (2D radiograph); Malawi: L. Malawi; D.H. Eccles, 24-Apr-1969. — Uncatalagued, 4 (c+s), MKO80-93; Malawi: L. Malawi: Domwe Island; M.K. Oliver, K. McKaye, T.D. Kocher, & A. Grace, 3-Aug-1980.

Melanochromis auratus (Boulenger, 1897): BMNH 1896.12.1.7, 1 (holotype; 2D radiograph available at https://data.nhm.ac.uk/object/7d7d5d1e-eccd-41dd-9adb-41ea8570d099); Malawi: L. Malawi: Monkey Bay; G. Pigott. — AMNH 31901, 2 (2D radiograph); Malawi: L. Malawi; W.C. Ambali, 23-Mar-1969. — AMNH 31904, 1 (2D radiograph); Malawi: L. Malawi: Monkey Bay, 12-Dec-1966. — Uncatalagued, 2 (c+s); MKO80-93; Malawi: L. Malawi: Domwe Island; M.K. Oliver, K. McKaye, T.D. Kocher, A. Grace, 3-Aug-1980.

Melanochromis chipokae Johnson, 1975: USNM 214173, 1 (holotype; 2D radiograph available at http://n2t.net/http://n2t.net/ark:/65665/m3eecc57b0-c050-48c6-af2f-1c836a038b71); Malawi: Central region: off Chipoka Island; T.E. Davies & party.

Melanochromis loriae Johnson, 1975: USNM 214175, 1 (holotype; 2D radiograph available at http://n2t.net/ark:/65665/3dcf85529-f27d-4990-9960-a058eb7993b3); Malawi: L. Malawi: off Chipoka Island; T.E. Davies & party. — USNM 214176, 1 (paratype; 2D radiograph available at http://n2t.net/ark:/65665/342990f51-a434-4bec-80cd-639ad3a41b14); other data same as USNM 214175. — USNM 215029, 1 (holotype of *M. parallelus* = *M. loriae*; 2D radiograph available at http://n2t.net/ark:/65665/3a413654f-7d0d-4feb-a66d-0fcf4e80b45d); Malawi: "Lake Malawi"; H.R. Axelrod, Oct-1974. — USNM 215030, 1 (paratype of *M. parallelus* = *M. loriae*; 2D radiograph available at http://n2t.net/ark:/65665/3b70fdabd-140b-4f72-afec-a5fa0a6819f8); other data same as USNM 215029.

Melanochromis melanopterus Trewavas, 1935: AMNH 31889, 3 (2D radiograph); Malawi: L. Malawi: Nkhata Bay; D.H. Eccles, 2-Oct-1954. — AMNH 226068, 2 (2D radiograph); Malawi: L. Malawi, captive, exported 1975. — RMCA 99-41-P-1345-353, 2 (2D radiograph); L. Malawi. — Uncatalagued, 3 (c+s); MKO80-93; Malawi: L. Malawi: Domwe Island; M.K. Oliver, K. McKaye, T.D. Kocher, A. Grace, 3-Aug-1980. — YPM 014310, 1 (3D CT image series, MorphoSource http://n2t.net/ark:/87602/m4/M123825); Malawi: L. Malawi: Maleri Island, ENE side; M.K. Oliver, 2-May-1971.

Melanochromis simulans Eccles, 1973: USNM 210700, 1 (holotype; 2D radiograph available at http://n2t.net/ark:/65665/39c29b050-ff7c-42b0-b8d9-285748f8ea42); Malawi: L. Malawi: S of Cape Ngombo on E coast about 20 miles N of Monkey Bay; T.E. Davies.

Melanochromis cf. *vermivorus* Trewavas, 1935: AMNH 31896, 2 (2D radiograph); Malawi: L. Malawi: Monkey Bay, Thumbi Island East; D.H. Eccles, 27-Aug-1967.

Petrotilapia genalutea Marsh, 1983: YPM 014173, 1 (3D CT image series, MorphoSource http://n2t.net/ark:/87602/m4/426131); Malawi: L. Malawi: SE arm: W side Boadzulu Island; M.K. Oliver & party, 15-Aug-1971.

Petrotilapia nigra Marsh, 1983: YPM 014591, 1 (3D CT image series, MorphoSource http://n2t.net/ark:/87602/m4/426255); Malawi: L. Malawi: Nankumba Peninsula 300 m E of Otter Island; M.K. Oliver, K. McKaye, & T.D. Kocher, 4-Jun-1980.

Petrotilapia tridentiger Trewavas, 1935: YPM 007854, 1 (3D CT image series, MorphoSource http://n2t.net/ark:/87602/m4/426297); Malawi: L. Malawi: Monkey Bay.

Petrotilapia sp.: AMNH 19131, 1 (2D radiograph); Malawi: L. Malawi: Deep Bay; R. & L. Boulton, 4-Jul-1929. — AMNH 31888, 1 (2D radiograph); Malawi: L. Malawi: Monkey Bay; D.H. Eccles, 16-Nov-1962. — AMNH 31895, 1 (2D radiograph); Malawi: L. Malawi: Monkey Bay; D.H. Eccles, 25-Oct-1962. — AMNH 31905, 1 (2D radiograph); Malawi: L. Malawi: Monkey Bay, Thumbi Island East; D.H. Eccles, 23-Aug-1966.

Pseudotropheus elegans Trewavas, 1935: BMNH 1935.6.14.127, 1 (holotype; 2D radiograph available at https://data.nhm.ac.uk/object/2bbd66c4-f176-4e84-8854-c0f06b703daa); Malawi: L. Malawi: Deep Bay; C. Christy, Aug-1925.

Pseudotropheus interruptus (Johnson, 1975): USNM 214221, 1 (holotype; 2D radiograph available at http://n2t.net/ark:/65665/3c22cf31c-ad60-4134-bd72-bc95a813d3f9); Malawi: L. Malawi: off Likoma Island; T.E. Davies & party. — USNM 214222, 1 (paratype; 2D radiograph available at http://n2t.net/ark:/65665/313ce1c12-e66b-4af7-84eb-cddd486a38e9); same data as holotype.

Pseudotropheus johannii Eccles, 1973: USNM 210702, 1 (holotype; 2D radiograph); Malawi: L. Malawi: S of Cape Ngombo on E coast about 20 miles N of Monkey Bay; T.E. Davies. — USNM 210701, 1 (paratype; 2D radiograph); same data as holotype. — AMNH 215563, 35 (2D radiograph); captive, wild reportedly collected from Malawi: L. Malawi, near Malawi/Mozambique border.

Pseudotropheus livingstonii (Boulenger, 1899): BMNH 1863.11.12.22, 1 (holotype; 2D radiograph available at https://data.nhm.ac.uk/object/40204ab9-9047-4980-8983-0723a678274e); Lake Malawi; D. Livingstone Zambezi Expedition. — AMNH 221238, 4 (2D radiograph); Malawi: L. Malawi: SE arm: Crocodile Rocks; A. Konings, 3-Dec-1989. — AMNH 226057, 2 (2D radiograph); Mozambique: L. Malawi: Metangula; D.H. Eccles, 10-Dec-1960.

Pseudotropheus lucerna Trewavas, 1935: AMNH 31898, 1 (2D radiograph); Malawi: L. Malawi: Nkhata Bay; D.H. Eccles, 20-Jul-1962.

Tropheops tropheops (Regan, 1922): CUMV 89948, 7 (2D radiograph); Malawi: L. Malawi: Monkey Bay, Harbour Island; M.E. Arnegard & A. Chambala, 25-Jun-1996.

Pseudocrenilabrini: Rhamphochromina

Diplotaxodon argenteus Trewavas, 1935: AMNH 221945, 4 (2D radiograph); Malawi: L. Malawi: SE arm, off Michesi between Namiasi and Boadzulu Island in 27 m; M.K. Oliver & party, 14-Aug-1971. — AMNH 222088, 4 (2D radiograph); Malawi: L. Malawi: SW arm, LMTS station Malembo III, 55-64 m depth; K. Stride, J. Tarbit, & M.K. Oliver, 19-Jul-1971. — BMNH 1935.6.14.2281–2282, 2 (lectotype and paralectotype; 2D radiograph available at); Malawi: L. Malawi: Bar House; C. Christy, 1925.

Diplotaxodon ecclesi Burgess & Axelrod, 1973: USNM 210696, 1 (holotype; 2D radiograph); Malawi: L. Malawi: off Monkey Bay in 79 m; H.R. Axelrod, 19-May-1973.

Diplotaxodon greenwoodi Stauffer & McKaye, 1986: USNM 270847, 1 (holotype; 2D radiograph available at http://n2t.net/ark:/65665/3266d4bef-28a4-4410-b02d-818e062cb27c); Malawi: L. Malawi: 8 km S of Mumbo Island in 86 m depth; K.R. McKaye & J.R. Stauffer, 17-Apr-1984.

Diplotaxodon limnothrissa Turner, 1994: YPM 26569, 10 (2D radiograph); Malawi: L. Malawi: Nkhata Bay area; M.K. Oliver, K. McKaye, & T.D. Kocher, purchased from purse seine catch of *Orion*, 21–22-Jul-1980.

Pallidochromis tokolosh Turner, 1994: YPM 26900, 4 (2D radiograph); Malawi: L. Malawi: SE arm, trawled, F/V *Crystal Lake*; 1991.

Rhamphochromis brevis Trewavas, 1935: BMNH 1935.6.14.2182–2183, 2 (lectotype & paralectotype; 2D radiograph available at https://data.nhm.ac.uk/object/4a0b5fba-37ec-487c-8d8e-a01844a775c8); Malawi: L. Malawi: Fort Johnston to Fort Maguire; C. Christy, Nov–Dec-1925.

Rhamphochromis esox (Boulenger, 1908): AMNH 233506, 2 (as *R. leptosoma*; 2D radiograph); Malawi: L. Malawi: Monkey Bay; M.K. Oliver, J. Tarbit, & party, 6-May-1971.

Rhamphochromis woodi Regan, 1922: YPM 032044, 1 (2D radiograph); Malawi: L. Malawi: Nankumba Peninsula: S of Otter Island; M.K. Oliver, K. McKaye, T.D. Kocher, 20-Jul-1980.
